# Author Correction: Proteogenomic analysis reveals RNA as a source for tumor-agnostic neoantigen identification

**DOI:** 10.1038/s41467-024-46724-8

**Published:** 2024-03-15

**Authors:** Celina Tretter, Niklas de Andrade Krätzig, Matteo Pecoraro, Sebastian Lange, Philipp Seifert, Clara von Frankenberg, Johannes Untch, Gabriela Zuleger, Mathias Wilhelm, Daniel P. Zolg, Florian S. Dreyer, Eva Bräunlein, Thomas Engleitner, Sebastian Uhrig, Melanie Boxberg, Katja Steiger, Julia Slotta-Huspenina, Sebastian Ochsenreither, Nikolas von Bubnoff, Sebastian Bauer, Melanie Boerries, Philipp J. Jost, Kristina Schenck, Iska Dresing, Florian Bassermann, Helmut Friess, Daniel Reim, Konrad Grützmann, Katrin Pfütze, Barbara Klink, Evelin Schröck, Bernhard Haller, Bernhard Kuster, Matthias Mann, Wilko Weichert, Stefan Fröhling, Roland Rad, Michael Hiltensperger, Angela M. Krackhardt

**Affiliations:** 1 German Cancer Consortium (DKTK), partner site Munich and German Cancer Research Center (DKFZ), Heidelberg, Germany; 2 Technical University of Munich, TUM School of Medicine, Klinikum rechts der Isar, IIIrd Medical Department, Munich, Germany; 3 Technical University of Munich, TUM School of Medicine, Klinikum rechts der Isar, IInd Medical Department, Munich, Germany; 4 Technical University of Munich, TUM School of Medicine, Center for Translational Cancer Research (TranslaTUM), Munich, Germany; 5 Technical University of Munich, TUM School of Medicine, Institute of Molecular Oncology and Functional Genomics, Munich, Germany; 6 Department of Proteomics and Signal Transduction, Max Plank Institute of Biochemistry, Munich, Germany; 7 Institute for Research in Biomedicine, Università della Svizzera italiana, Bellinzona, Switzerland; 8 Technical University of Munich, TUM School of Life Sciences, Chair of Proteomics and Bioanalytics, Freising, Germany; 9 Technical University of Munich, TUM School of Life Sciences, Computational Mass Spectrometry, Freising, Germany; 10 German Cancer Consortium (DKTK), partner site Heidelberg and German Cancer Research Center (DKFZ), Heidelberg, Germany; 11 Molecular Precision Oncology Program, NCT Heidelberg, Heidelberg, Germany; 12 Technical University of Munich, TUM School of Medicine, Klinikum rechts der Isar, Institute of Pathology, Munich, Germany; 13 German Cancer Consortium (DKTK), partner site Berlin and German Cancer Research Center (DKFZ), Heidelberg, Germany; 14 Charité Comprehensive Cancer Center, Charité – Universitätsmedizin Berlin, Berlin, Germany; 15 Department of Hematology, Oncology and Tumor Immunology, Charité – Universitätsmedizin Berlin, Berlin, Germany; 16 German Cancer Consortium (DKTK), partner site Freiburg and German Cancer Research Center (DKFZ), Heidelberg, Germany; 17 Institute of Medical Bioinformatics and Systems Medicine (IBSM), Medical Center - University of Freiburg, Faculty of Medicine, University of Freiburg, Freiburg, Germany; 18 Department of Hematology and Oncology, Medical Center, University of Schleswig Holstein, Campus Lübeck, Lübeck, Germany; 19 German Cancer Consortium (DKTK), partner site Essen and German Cancer Research Center (DKFZ), Heidelberg, Germany; 20 Department of Medical Oncology and Sarcoma Center, West German Cancer Center, University Hospital Essen, Essen, Germany; 21 Clinical Division of Oncology, Department of Internal Medicine, Medical University of Graz, Graz, Austria; 22 University Comprehensive Cancer Center Graz, Medical University of Graz, Graz, Austria; 23 Technical University of Munich, TUM School of Medicine, Klinikum rechts der Isar, Department of Surgery, Munich, Germany; 24 German Cancer Consortium (DKTK), partner site Dresden and German Cancer Research Center (DKFZ), Heidelberg, Germany; 25 Core Unit Molecular Tumor Diagnostics (CMTD), NCT Dresden, Dresden, Germany; 26 Institute for Medical Informatics and Biometry, Faculty of Medicine, TU Dresden, Dresden, Germany; 27 Institute for Clinical Genetics, University Hospital Carl Gustav Carus at the Technische Universität Dresden, Dresden, Germany; 28 ERN GENTURIS, Hereditary Cancer Syndrome Center Dresden, Dresden, Germany; 29 National Center for Tumor Diseases Dresden (NCT/UCC), Dresden, Germany; 30 Faculty of Medicine and University Hospital Carl Gustav Carus, Technische Universität Dresden, Dresden, Germany; 31 Helmholtz-Zentrum Dresden-Rossendorf (HZDR), Dresden, Germany; 32 Max Planck Institute of Molecular Cell Biology and Genetics, Dresden, Germany; 33 Technical University of Munich, TUM School of Medicine, Klinikum rechts der Isar, Institute of AI and Informatics in Medicine, Munich, Germany; 34 Technical University of Munich, TUM School of Life Sciences, Bavarian Biomolecular Mass Spectrometry Center (BayBioMS), Freising, Germany; 35 Division of Translational Medical Oncology, National Center for Tumor Diseases (NCT) Heidelberg, German Cancer Research Center (DKFZ), Heidelberg, Germany; 36 Malteser Krankenhaus St. Franziskus-Hospital, Flensburg, Germany

**Keywords:** Tumour immunology, Tumour immunology, Translational research, Cancer immunotherapy

Correction to: *Nature Communications* 10.1038/s41467-023-39570-7, published online 02 August 2023

With respect to the original “Data availability” statement, since the publication of this work, to ensure patient data privacy protection per request of the authors’ institute *Technical University of Munich*, the RNA-seq dataset from sorted CD8+ TILs originally deposited to the European Nucleotide Archive (ENA) with study accession number PRJEB61429 has been transferred to the European Genome-phenome Archive (EGA) under restricted access with the study identifier EGAS00001006706 (along with the other sequencing datasets generated in the study) and the dataset identifier EGAD50000000193. The “Data availability” section of the Article file (PDF and HTML versions) and the Data section of the Reporting Summary have been updated to reflect these changes.

