# Supplementary material for: Proteogenomic analysis reveals RNA as a source for tumor-agnostic neoantigen identification
Source: Nat Commun. 2023 Aug 2;14:4632. doi: 10.1038/s41467-023-39570-7 (PMC10397250; doi:10.1038/s41467-023-39570-7)
Supplement: Supplementary file 1 — Supplementary Information [file 41467_2023_39570_MOESM1_ESM.pdf]

Supplementary information

Includes Supplementary Figures 1 to 15 and Supplementary Tables 1 to 4

Proteogenomic analysis reveals RNA as a source for tumor-agnostic neoantigen identification

Celina Tretter*, Niklas de Andrade Krätzig*, Matteo Pecoraro, Sebastian Lange, Philipp Seifert, Clara von Frankenberg, Johannes Untch, Gabriela Zuleger, Mathias Wilhelm, Daniel P Zolg, Florian S Dreyer, Eva Bräunlein, Thomas Engleitner, Sebastian Uhrig, Melanie Boxberg, Katja Steiger, Julia Slotta-Huspenina, Sebastian Ochsenreither, Nikolas von Bubnoff, Sebastian Bauer, Melanie Boerries, Philipp J Jost, Kristina Schenck, Iska Dresing, Florian Bassermann, Helmut Friess, Daniel Reim, Konrad Grützmann, Katrin Pfütze, Barbara Klink, Evelin Schröck, Bernhard Haller, Bernhard Kuster, Matthias Mann, Wilko Weichert, Stefan Fröhling, Roland Rad, Michael Hiltensperger[#] and Angela M Krackhardt[#]

* These authors contributed equally to this work

[#] These authors jointly supervised this work

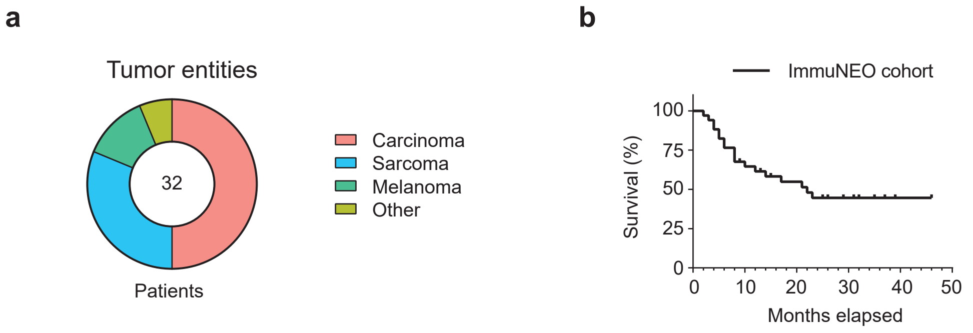

Supplementary Figure 1 | ImmuNEO MASTER cohort. a Distribution of the major groups of tumor entities of patients included in the ImmuNEO MASTER cohort. **b** Overall survival of ImmuNEO MASTER patients since tumor resection in months. **a**, **b**, n = 32 patients (see Supplementary Table 1). Source data are provided as a Source Data file.

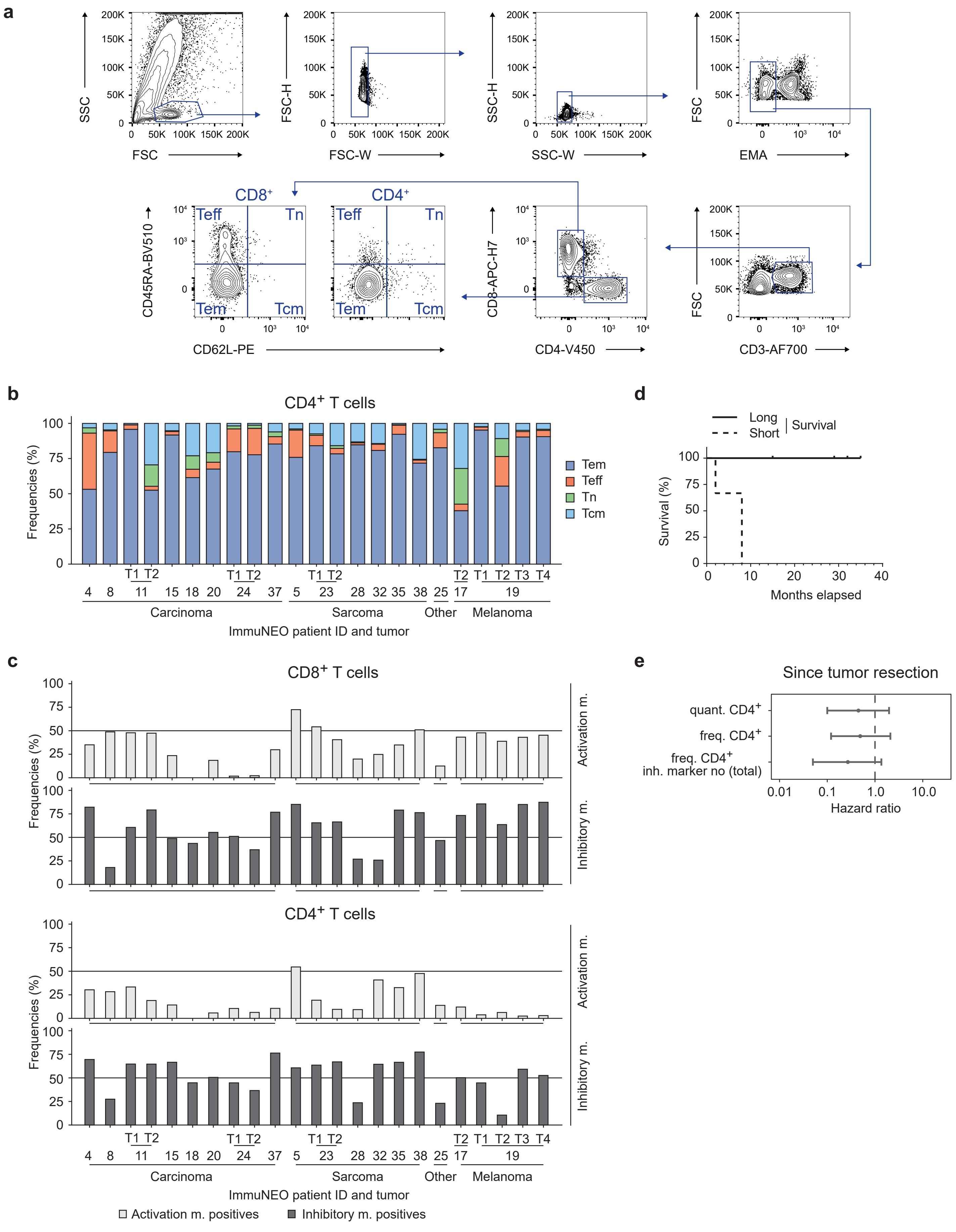

Supplementary Figure 2

Supplementary Figure 2 | Analysis of the tumor microenvironment. a Flow cytometric gating strategy for $CD4^+$ and $CD8^+$ T cell subsets. **b** Frequencies of different $CD4^+$ T cell subsets of all identified tumor-infiltrating $CD4^+$ T cells per patient and grouped by tumor entity. **c** Frequencies of $CD4^+$ (bottom) and $CD8^+$ T cells (top) per patient and grouped by tumor entity, expressing at least one activation marker (HLA-DR, CD103) or inhibitory marker (PD-1, TIM-3, LAG-3). **d** Kaplan-Meier survival estimation since tumor resection of patients with short survival (below 1 year, n = 3; ImmuNEO patient 20, 23, 32) and long survival (above 1 year, n = 5; ImmuNEO patient 11, 15, 19, 24, 25). **e** Forest plot showing the hazard ratio calculated by log rank test and Cox's proportional hazards model of several phenotypic parameters for the survival of patients since tumor resection (n = 17). For statistical analysis only one representative tumor sample per patient was used (see core cohort Supplementary Table 1). Data are shown as hazard ratio (dot) and 95% confidence intervals (lines). **a-c**, n = 23 tumor samples from n = 17 patients (see Supplementary Table 1). freq., frequency; m., marker; NK, natural killer; quant., quantified cells per gram tumor; T, tumor; Tcm, central memory T cells; Teff, effector T cells; Tem, effector memory T cells; Tn, naïve T cells. Source data are provided as a Source Data file.

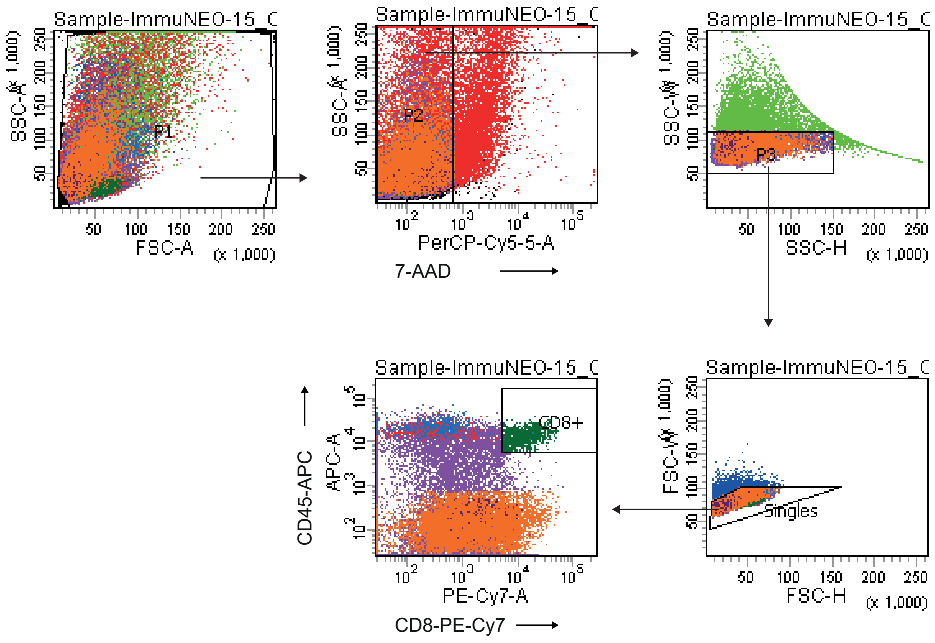

Tube: ImmuNEO-15_OP1

Supplementary Figure 3 | Sorting strategy of CD8^{+} T cells in the tumor microenvironment. Sort report of CD8^{+} T cells from the tumor sample of patient ImmuNEO-15.

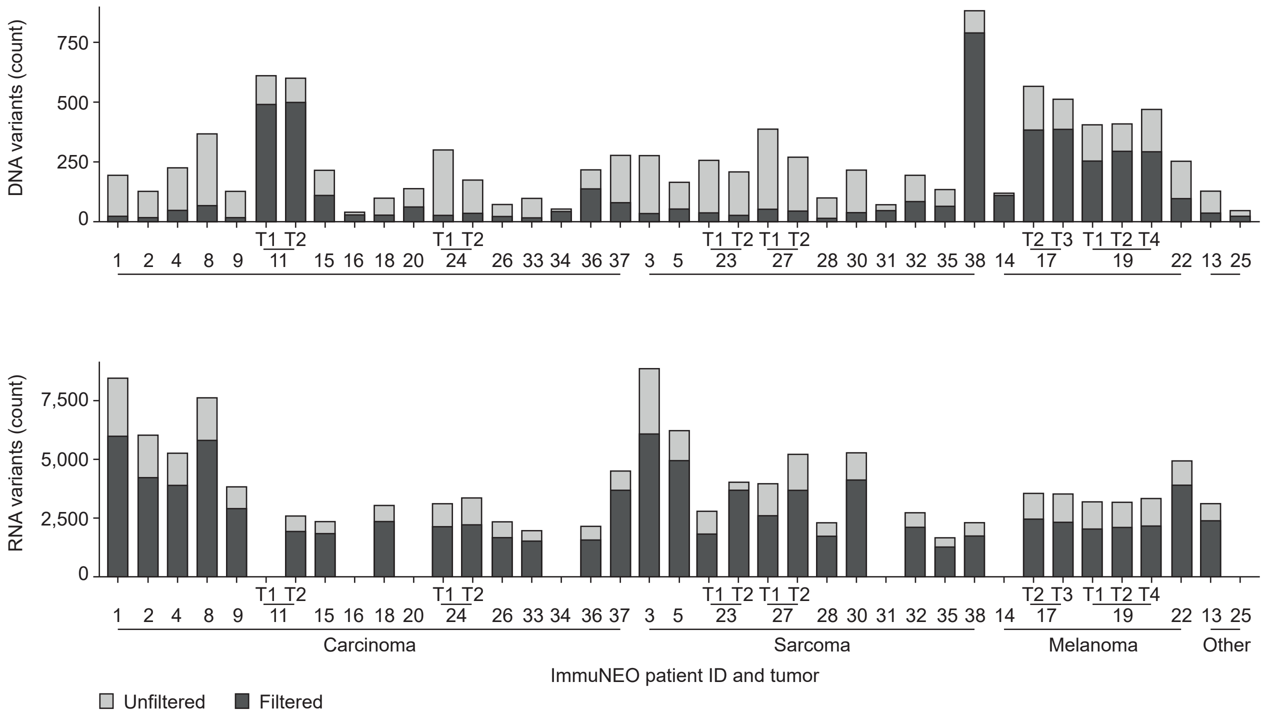

Supplementary Figure 4 | Quality assessment of genetic variants identified at the DNA and RNA level. Total unfiltered genetic variants identified by MuTect2 (v4.1.0.0) from whole exome (WES)/whole genome sequencing (WGS) data (DNA variants; upper panel) and by Strelka2 (v2.9.10) from RNA sequencing (RNA-seq) data (RNA variants; lower panel) are shown per tumor sample and grouped by tumor entity. Variants passed filtering for quality assessment only if they showed at least a coverage of 5 reads, a variant frequency of 5%, and 2 mutated reads within the tumor as well as not more than 1 mutated read within normal control tissue. n = 39 tumor samples from n = 32 patients for WES/WGS data; n = 32 tumor samples from n = 26 patients for RNA-seq data (see Supplementary Table 1). T, tumor. Source data are provided as a Source Data file.

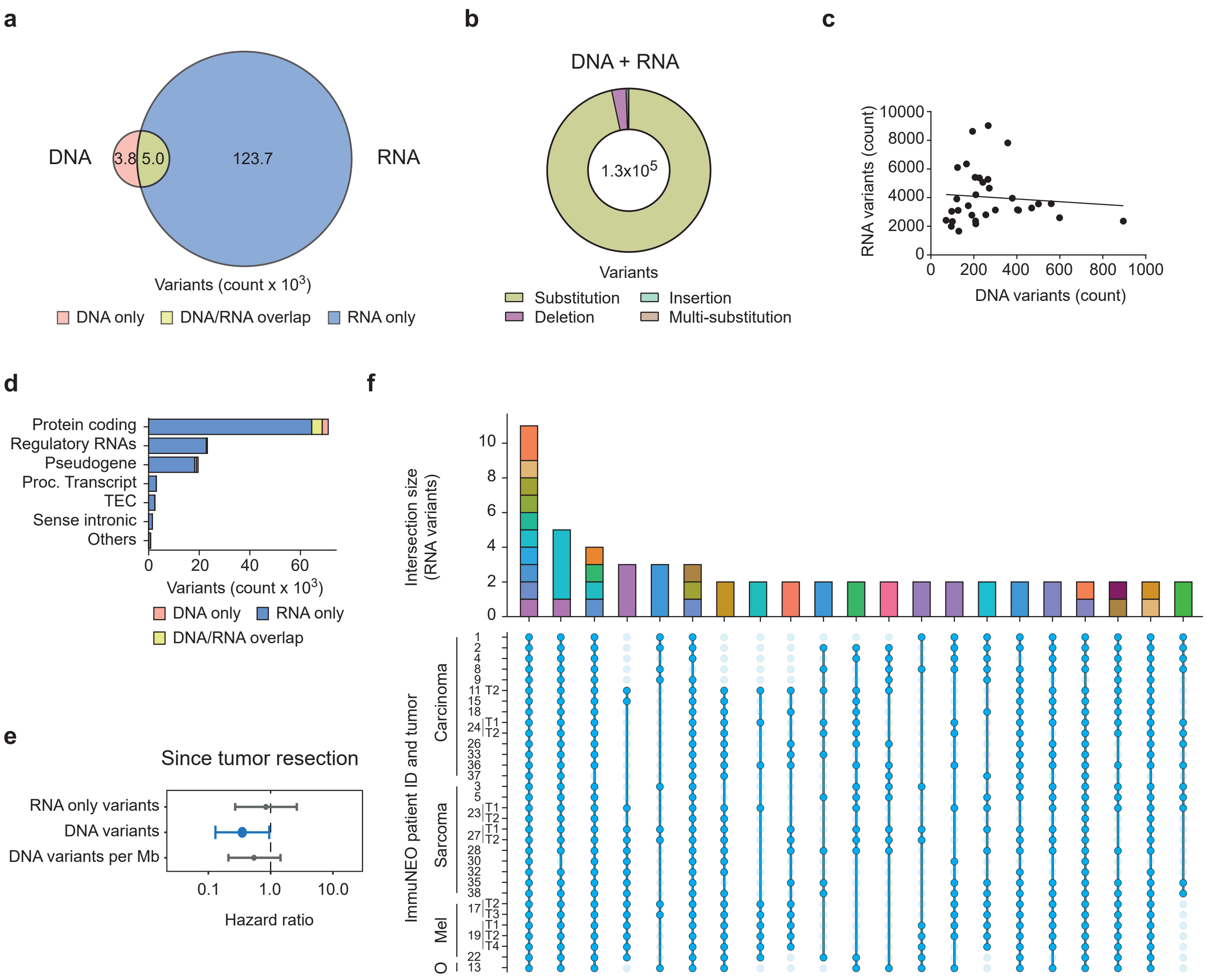

Supplementary Figure 5 | Genetic variants identified at the DNA and RNA level in tumor tissue from different cancer entities. a Venn diagram showing the overlap between all variants identified from whole exome (WES)/whole genome sequencing (WGS) data (DNA variants) and from RNA sequencing (RNA-seq) data (RNA variants). **b** Distribution of each mutation type for all identified genetic variants regardless of the sequencing origin (WES/WGS and RNA-seq combined). **c** Correlation of DNA variants with RNA variants identified from tumor samples where matching WES/WGS and RNA-seq data was available (n = 32 tumor samples). Symbols depict individual tumor samples; Spearman's rank correlation analysis, $\rho = 0.1578$; line depicts linear regression, $R^2=0.008$. **d** Bar graph showing the number of variants found in each genetic biotype and the originating dataset. **e** Forest plot showing the hazard ratio calculated by log rank test and Cox's proportional hazards model of several genetic parameters for the survival of patients since tumor resection (DNA variants n = 32 patients, RNA variants n = 26 patients). Significant results ($p \leq 0.05$) are highlighted in blue. For statistical analysis only one representative tumor sample per patient was used (see core cohort Supplementary Table 1). Data are shown as hazard ratio (dot) and 95% confidence intervals (lines). **f** Upset plot showing the overlap of at least two RNA variants between at least ten tumor samples. The bar graph shows the number of unique variants present in a shared subset of tumors defined as intersection size, dots indicate the tumor samples where the subset is present, and lines connect tumor samples within the same subset. The different genes harbouring the defined genetic variants are coloured in the intersection bar graph. **a**, **b**, **d**, **f**, n = 32 tumor samples from n = 26 patients for WES/WGS data and for RNA-seq data (see Supplementary Table 1). Mel, melanoma; Mb, mega base; O, other; OS, overall survival; Proc., processed; T, tumor; TEC, to be experimentally confirmed. Source data are provided as a Source Data file.

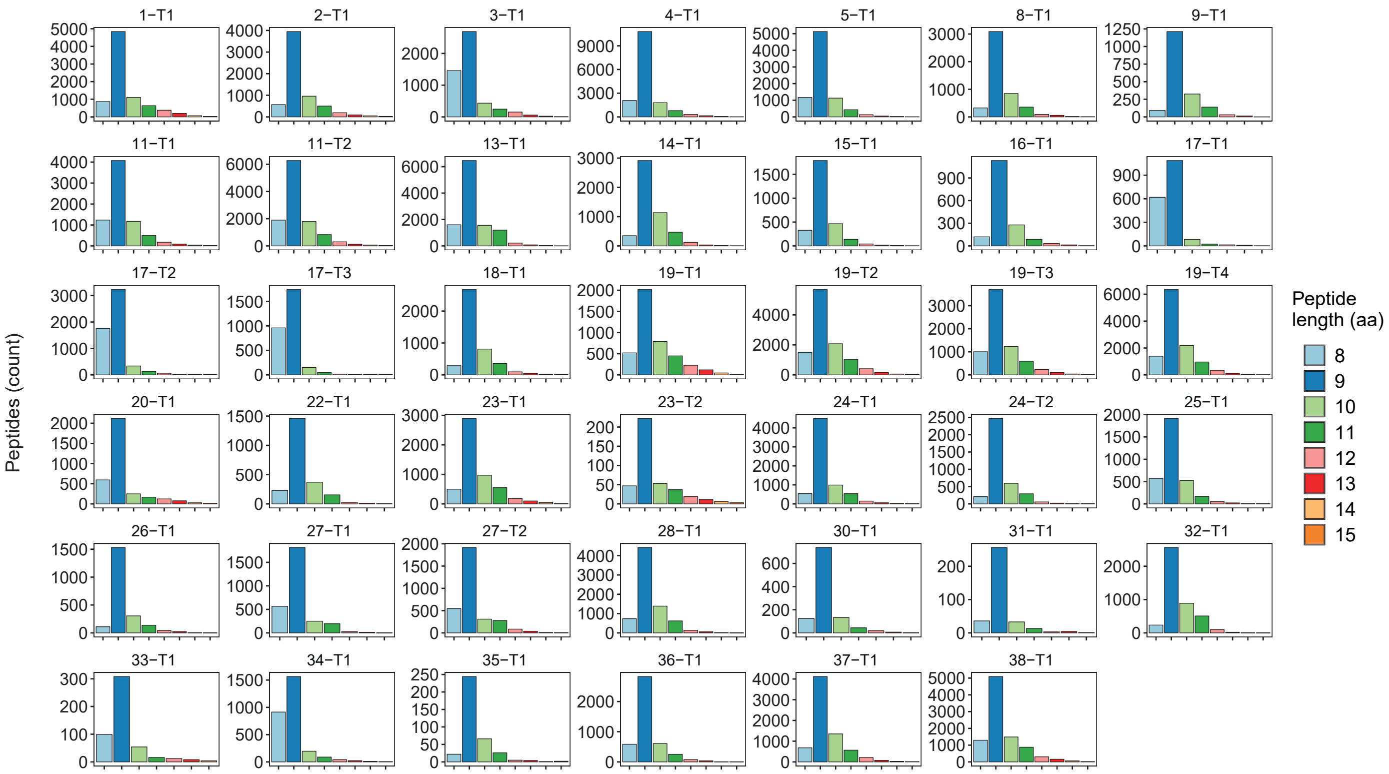

Supplementary Figure 6 | Length distribution of HLA class I peptides identified by mass spectrometry. Bar graph showing the number of unique peptides per peptide length in amino acids for every analysed tumor sample. Peptides bound to HLA class I molecules on the surface of the tumor cells have been isolated by immunoprecipitation and sequenced by liquid chromatography with tandem mass spectrometry (LC-MS/MS). Peptide sequences were then mapped with 1% FDR to the Ensemble92 protein database using pFind (v3.1.5) and unique sequences have been filtered. n = 41 tumor samples from n = 32 patients (see Supplementary Table 1). aa, amino acids; T, tumor.

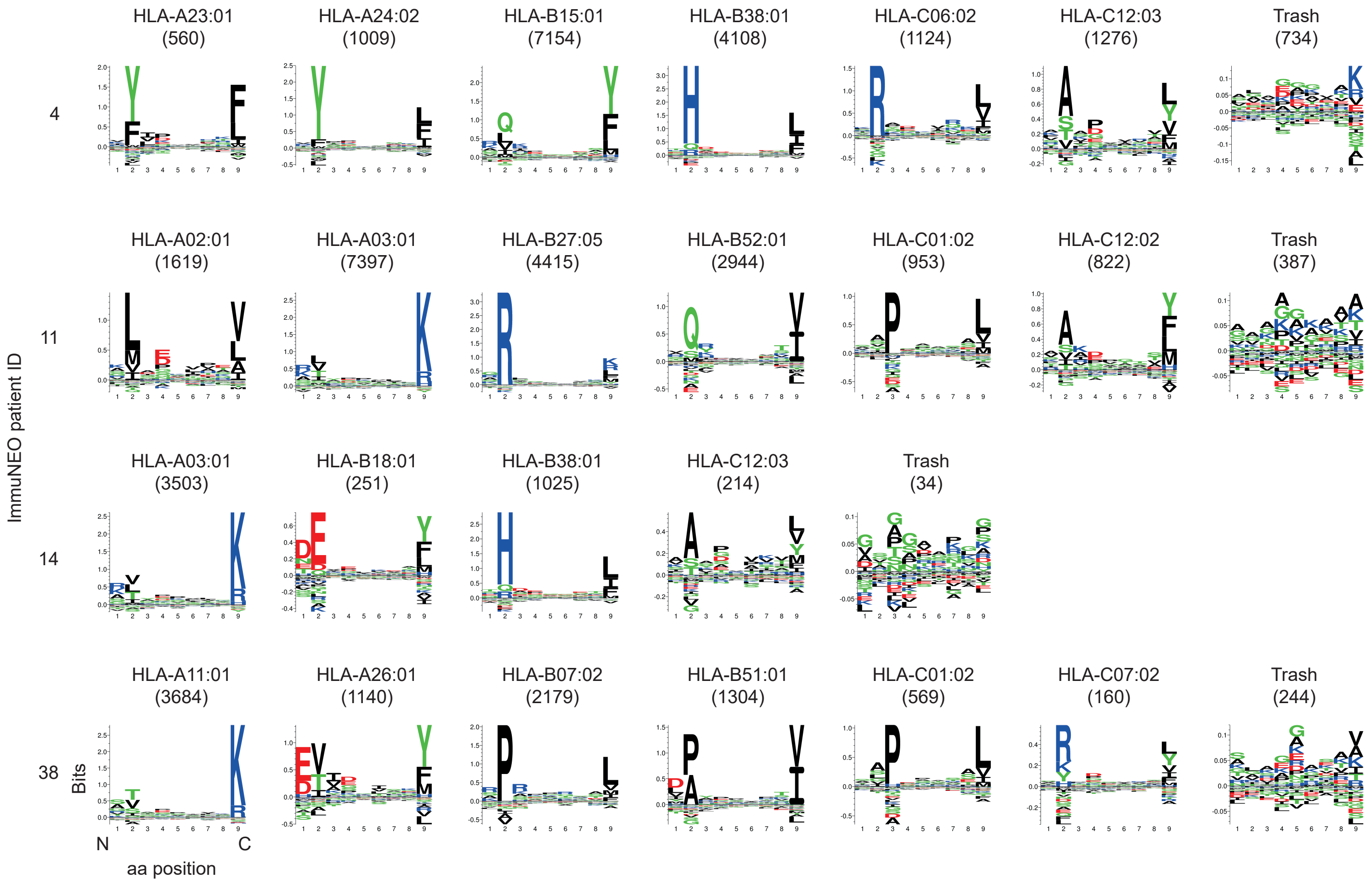

Supplementary Figure 7 | Peptide HLA class I binding motifs within the immunopeptidome. MHCMotifDecon (v1.0) has been used to match all isolated HLA class I peptides with lengths from 8-15 amino acids to the patients' HLA class I alleles according to their binding motifs and anchor residues for each tumor sample using standard settings. Binding motives of four representative tumor samples for each HLA class I allele are displayed with the total number of matched peptide sequences in brackets. Peptides not matching any HLA class I allele of the respective patient are displayed in the trash subgraph. aa, amino acid; HLA, human leukocyte antigen.

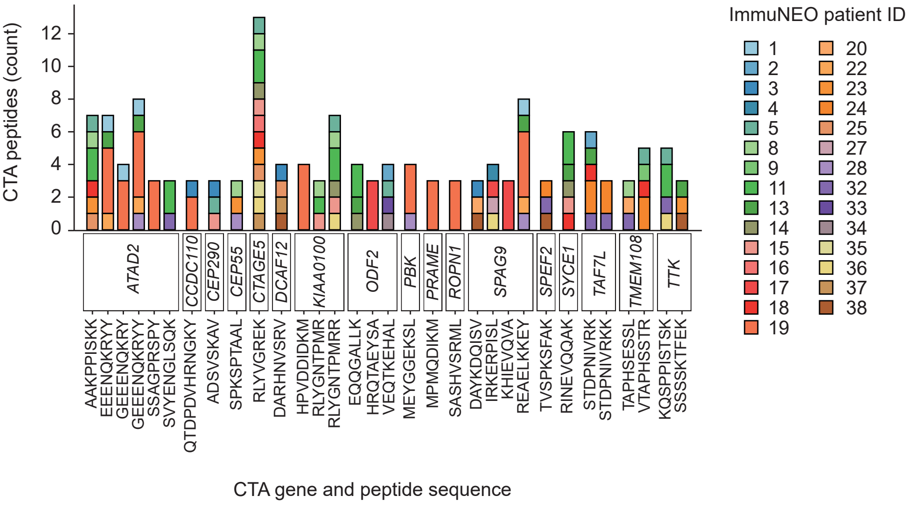

Supplementary Figure 8 | Shared CTA peptides in the HLA class I immunopeptidomes. Bar graph showing peptides origination from the annotated cancer testis antigen (CTA) genes, their sequence, and the patients where they have been found. n = 41 tumor samples from n = 32 patients (see Supplementary Table 1). CTA, cancer testis antigen.

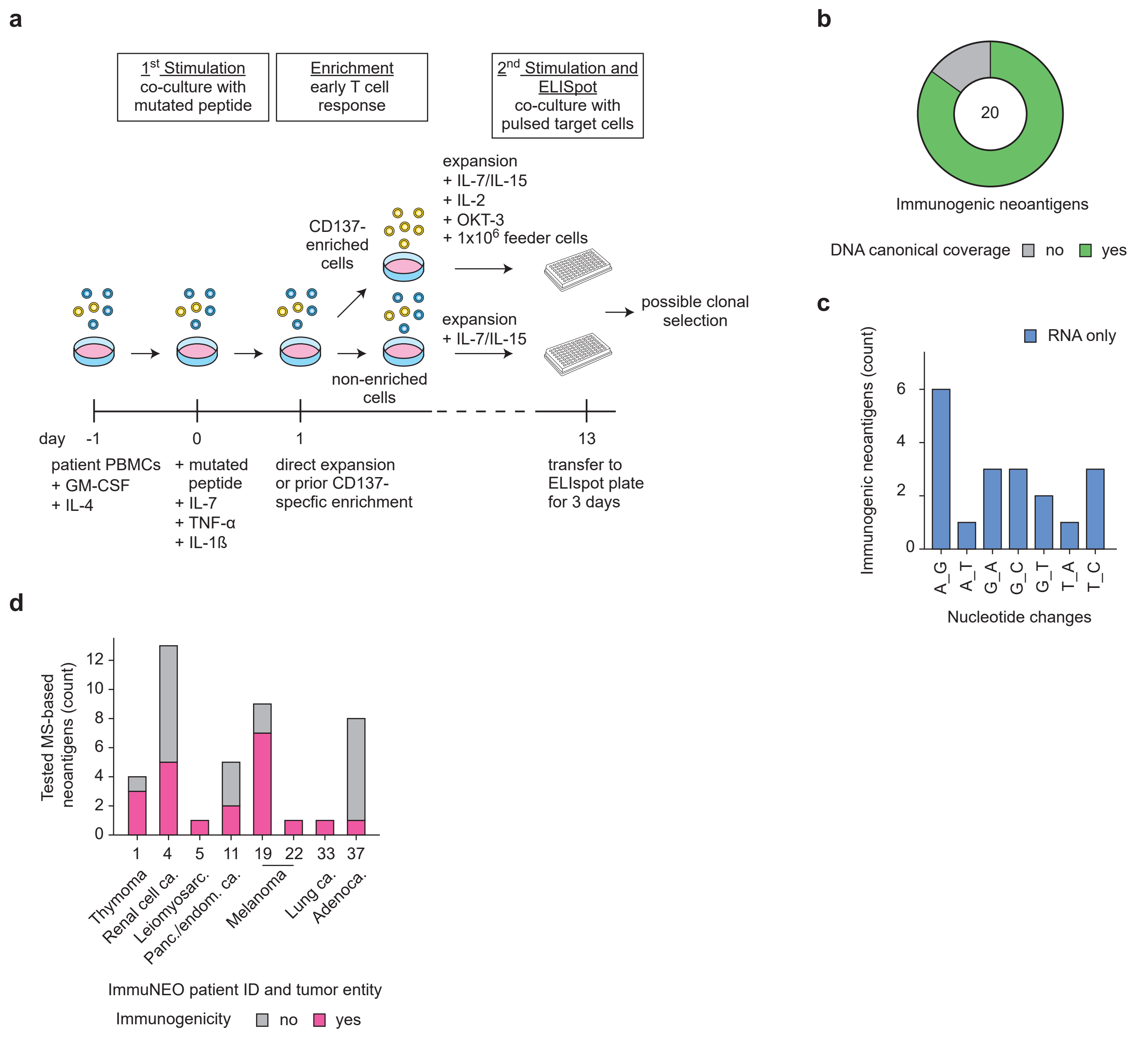

Supplementary Figure 9 | Characterization and immunogenicity assessment of neoantigen candidates. **a** Schematic overview of the immunogenicity assessment by modified accelerated co-cultured dendritic cell (acDC) assay using non-enriched or $CD137^{+}$-enriched T cells (PBMCs or TILs) for subsequent IFN-γ ELISpot readout. **b** Pie chart depicting the proportion of immunogenic neoantigens identified only from RNA sequencing (RNA-seq) data where the respective canonical sequence was identified at the DNA level with a coverage of ≥ 3 reads (green) or the respective region was not covered at the DNA level (grey, < 3 reads). **c** Distribution of the nucleotide exchange pattern of all single nucleotide variants that yield immunogenic neoantigen candidates identified only from RNA-seq data (n = 20). **d** For those patients with immunogenic neoantigens, the total number of tested neoantigen candidates is depicted including immunogenic and non-immunogenic ones. **b-d**, n = 78 neoantigen candidates from n = 24 patients were analysed in total; n = 8 patients harboured n = 21 immunogenic neoantigens; n = 1 immunogenic neoantigens from DNA and RNA variants; n = 20 neoantigen candidates from RNA variants. ca., carcinoma; endom., endometrium; GM-CSF, granulocyte macrophage-colony stimulating factor; IL, interleukin; OKT-3, Muromonab-CD3; Panc., pancreas; TNF-α, tumor necrosis factor-α. Source Data are provided as a Source Data file.

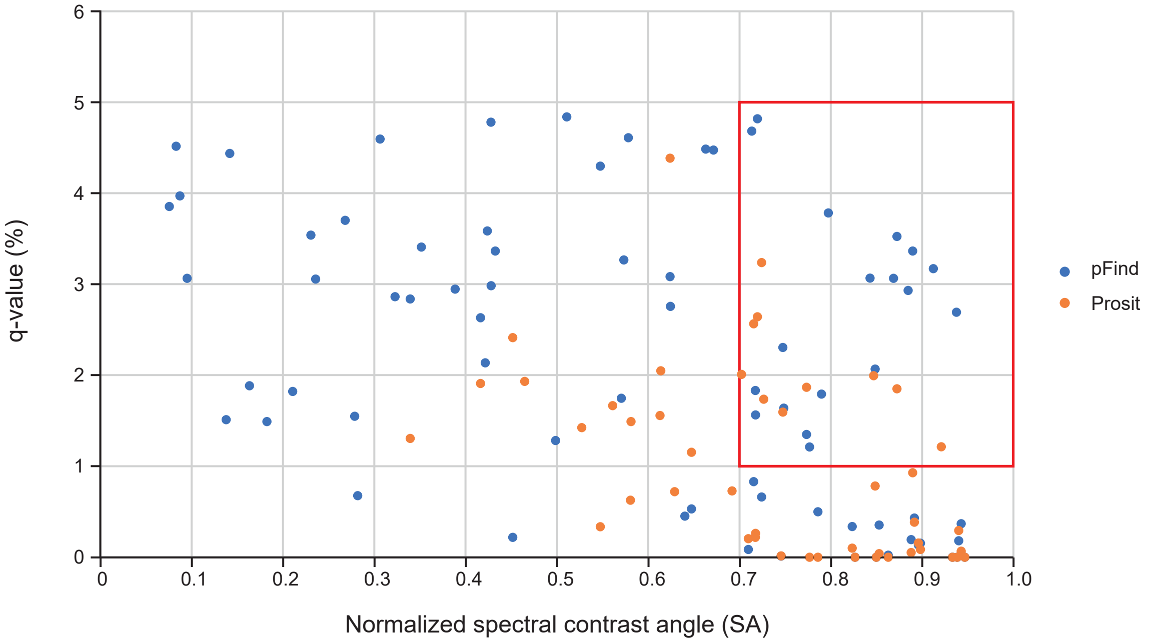

Supplementary Figure 10 | Verification of FDR for MS analysis. For all neoantigen candidates (n = 88) the q-values for their identification in the tumor is plotted against the respective best normalized spectral contrast angle (SA) between the measured and the synthetic or predicted spectra. Peptides identified with pFind are shown as blue dots, peptides identified with Prosit are shown as orange dots. SA, spectral contrast angle. Source data are provided as a Source Data file.

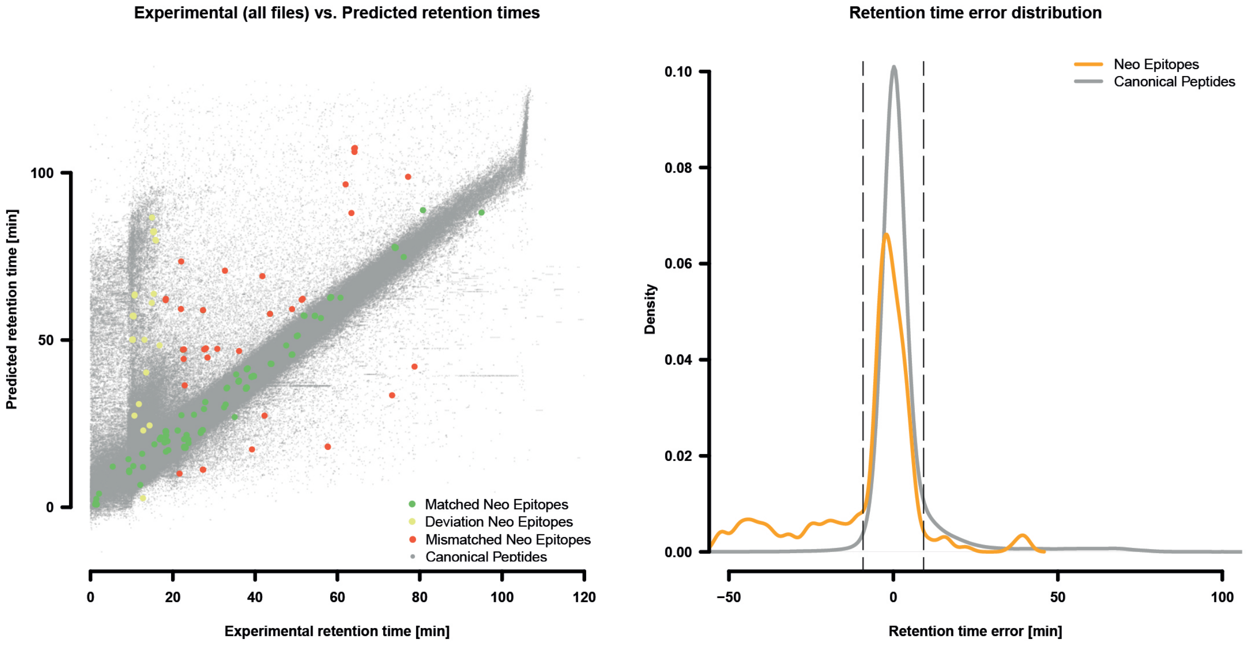

Supplementary Figure 11 | Peptide verification with predicted retention times. Retention times (RT) for each peptide were predicted using Prosit and compared to the respective experimental retention time (left). The error distribution between experimental and predicted RTs is shown in the right graph and all neoantigen candidates (n = 88) were considered matching (green dots, n = 45) or mismatching (red dots, n = 26) according to the extreme of the upper whisker of the absolute error boxplot for all datapoints (+/- 8.56 min). For some peptides (between the experimental RT range of 9 to 17 min) no accurate RT error could be predicted according to the distribution of canonical peptides and these were considered deviations (yellow dots, n = 17). Source data are provided as a Source Data file.

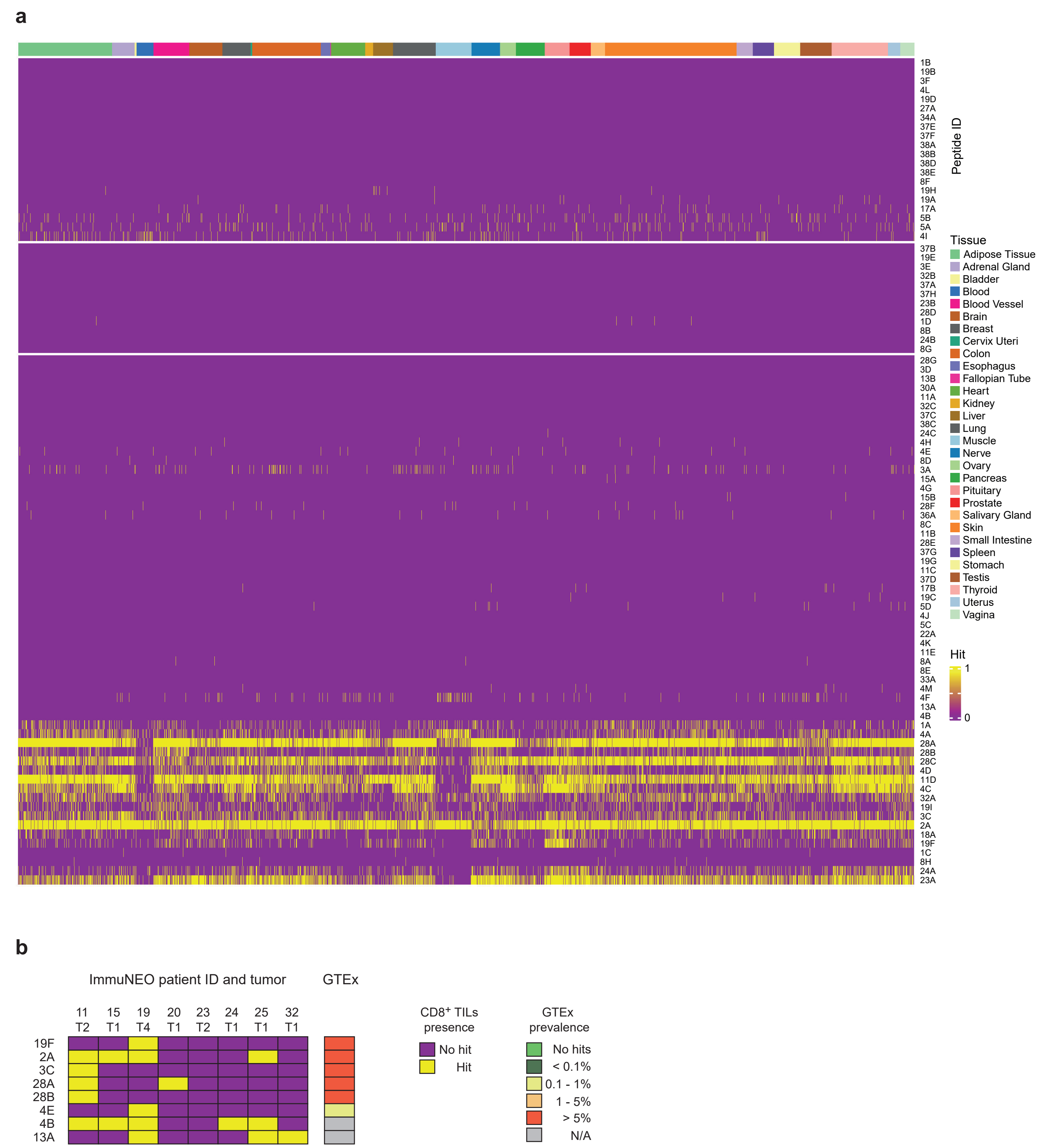

Supplementary Figure 12 | Prevalence of neoantigen candidate variants in healthy tissue. a Heatmap representation of the prevalence (at least 1 read) of each neoantigen candidate variant in RNA expression data of 10,269 samples from 30 different normal tissues from the Genotype-Tissue Expression (GTEx) project. **b** Heatmap representation of the presence of neoantigen candidate variants (left annotation) within total RNA sequencing data of sorted CD8$^+$ T cells from the tumor of ImmuNEO patients (upper annotation). The prevalence found in the GTEx data set is annotated on the right. Source data are provided as a Source Data file.

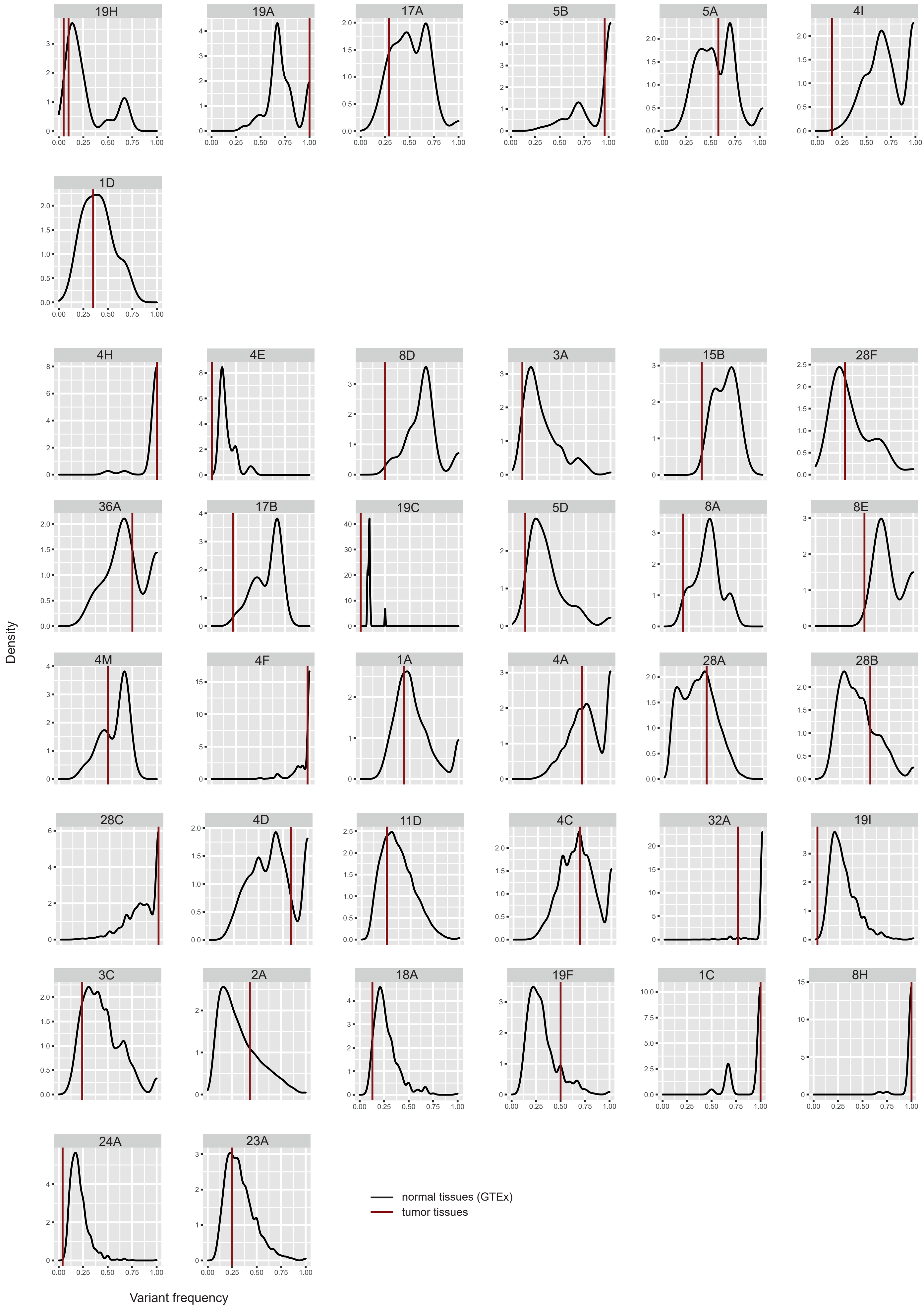

Supplementary Figure 13

Supplementary Figure 13 | Tumor and heathy tissue variant frequency distribution of all neoantigen candidate variants. For each neoantigen candidate variant the variant frequency found within the GTEx data set (10,269 samples from 30 different tissues) is plotted over all analyzed samples and the variant frequency found within the patient tumor sample is annotated with a red line.

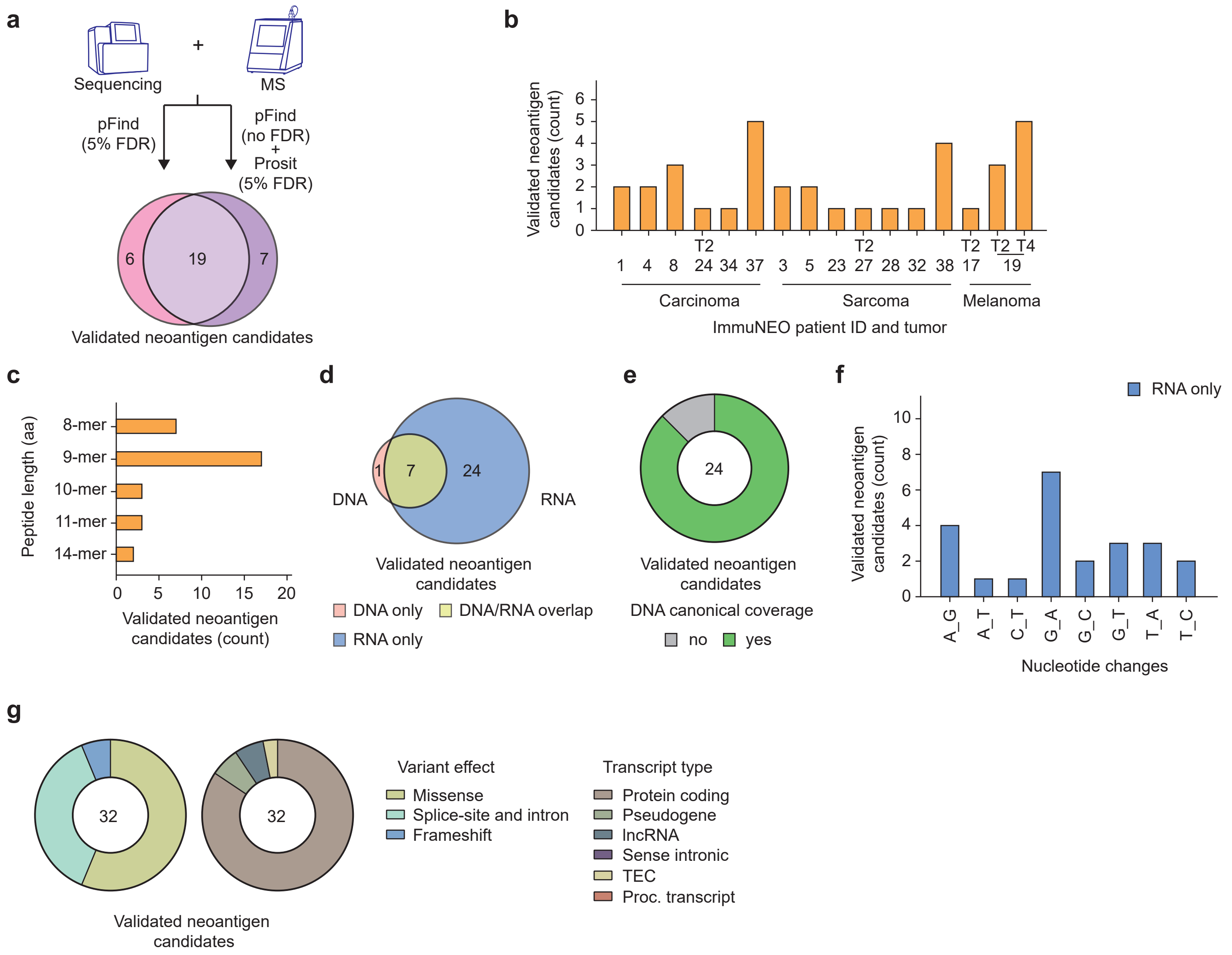

Supplementary Figure 14 | Proteogenomic identification of validated neoantigen candidates. a, b Number of identified validated neoantigen candidates based on the bioinformatics tool that they were identified with (**a**) and per tumor sample and grouped by tumor entity (**b**). pFind (v3.1.5) was used at 5% FDR on spectral level for the identification of non-canonical 8-15mer neoantigen candidates. The machine learning tool Prosit was integrated in addition for rescoring of the peptide spectra matching to the patient-specific personalized database using unfiltered pFind data as input. n = 39 tumor samples from n = 32 patients were analysed in total; n = 16 tumor samples from n = 15 patients harboured n = 32 validated neoantigen candidates. **c** Bar graph showing the length distribution of all identified neoantigen candidates in amino acids (aa). **d** Source (DNA or RNA data) of the variants that the identified validated neoantigen candidates were derived from. **e** Pie chart depicting the proportion of validated neoantigen candidates identified only from RNA sequencing (RNA-seq) data where the respective canonical sequence was identified at the DNA level with a coverage of ≥ 3 reads (green) or the respective region was not covered at the DNA level (grey, < 3 reads). **f** Distribution of the nucleotide exchange pattern of all variants that yield validated neoantigen candidates identified only from RNA-seq data. **g** Distribution of each mutation type (left) and biotype (right) of all variants that yield validated neoantigen candidates. **a-g**, n = 39 tumor samples from n = 32 patients were analysed in total; n = 16 tumor samples from n = 15 patients harboured n = 32 validated neoantigen candidates; n = 1 validated neoantigen candidates from DNA variants; n = 7 validated neoantigen candidates from DNA and RNA variants; n = 24 validated neoantigen candidates from RNA variants. aa, amino acids; MS, mass spectrometry; Proc., processed; T, tumor; TEC, to be experimentally confirmed. Source data are provided as a Source Data file.

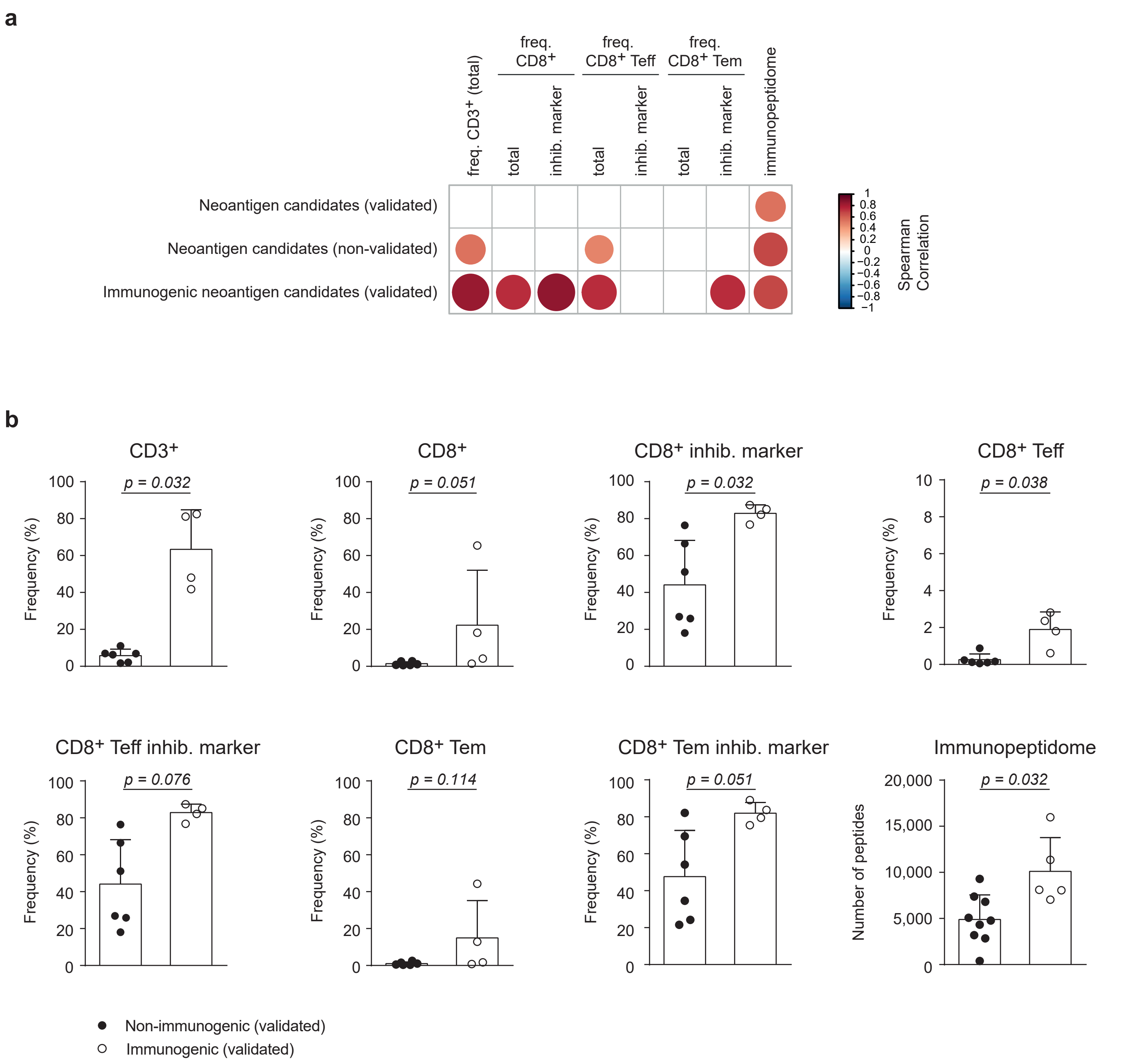

Supplementary Figure 15 | Correlation of immunogenic validated neoantigen candidates with features of the tumor microenvironment. a Correlation matrix summarizing significant ($p \leq 0.05$) Spearman correlations for multiple phenotypic parameters and the size of the immunopeptidome with the number of validated and non-validated neoantigen candidates as well as immunogenic validated neoantigen candidates. Spearman correlation coefficient Rho is labeled in color and size. For statistical analysis only one representative tumor sample per patient was used. **b** Bar graphs showing the frequencies of several immune cell subset and the size of the immunopeptidome for patients with and without validated immunogenic neoantigen candidates. Differences between both groups were assessed using a two-sided Mann-Whitney U test with Benjamini-Hochberg procedure for multiple testing and p values are shown for each graph. Data are shown as mean + s.d.. **a** Phenotypic correlations with validated (n = 19 patients), non-validated (n = 19 patients) and immunogenic validated (n = 10 patients) neoantigen candidates. Immunopeptidome correlations with validated (n = 32 patients), non-validated (n = 32 patients) and immunogenic validated (n = 14 patients) neoantigen candidates. **b** Phenotypic comparison with immunogenic (n = 4 patients) and non-immunogenic (n = 6 patients) validated neoantigen candidates. Immunopeptidome correlation with immunogenic (n = 5 patients) and non-immunogenic (n = 9 patients) validated neoantigen candidates. Freq., frequency; inhib., inhibitory; Teff, T effector cells; Tem, T effector memory cells. Source data are provided as a Source Data file.

General information					Subset	IME analysis		MS	Mutation Analysis		Survival data [months]					Immunotherapy Checkpoint Therapy					
Patient ID:	Tumor entity	Metastatic site	Staging at admission	Cohort	Core Samples	Pheno-typing	Sort & RNAseq	MS	WES (*WGS)	RNAseq tumor	Survival status	since ID	since MD	since MASTER	MD - MASTER	Received general	Response	Received prior admission	Response prior	Received post admission	Response post
**ImmuNEO-1.1**	Thymoma	lung	Stage Ivb	MASTER	Yes	x	x	Yes	Yes	Yes	alive	51	51	46	5	Yes	2	x	-	Yes	2
ImmuNEO-1.2		lung pericardium		-	x	x	x	x	x	x											
**ImmuNEO-2**	Mamma-Ca	primary	Stage IIb	MASTER	Yes	x	x	Yes	Yes	Yes	alive	41	41	37	4	x	-	x	-	x	-
**ImmuNEO-3**	Sarcoma (DSRCT)	primary	Stage IV	MASTER	Yes	x	x	Yes	Yes	Yes	deceased	29	27	23	4	x	-	x	-	x	-
**ImmuNEO-4**	Renal-cell-Ca	LN	Stage IV	MASTER	Yes	Yes	x	Yes	Yes	Yes	deceased	35	35	12	23	Yes	2	Yes	2	Yes	1
**ImmuNEO-5**	Leiomyosarcoma	lung	Stage IV	MASTER	Yes	Yes	x	Yes	Yes	Yes	deceased	17	17	4	13	x	-	x	-	x	-
**ImmuNEO-8**	Ovarian-Ca (neuroendocrine)	subcutaneous hypogastrium	Stage IV	MASTER	Yes	Yes	x	Yes	Yes	Yes	deceased	65	65	21	44	Yes	0	x	-	Yes	0
**ImmuNEO-9**	Thyroid-Ca	LN	Stage IV	MASTER	Yes	x	x	Yes	Yes	Yes	alive	39	39	39	0	x	-	x	-	x	-
ImmuNEO-11.1	Endometrium-Ca	primary	*n.a.*	MASTER	x	Yes	Yes	Yes	Yes	x	alive	63	63	32	31	x	-	x	-	x	-
**ImmuNEO-11.2**	Pancreas-Ca	LN	Stage IV	MASTER	Yes	Yes	Yes	Yes	Yes	Yes											
**ImmuNEO-13**	Testicle-Ca	LN	Stage IIIb	MASTER	Yes	x	x	Yes	Yes	Yes	deceased	21	21	10	10	x	-	x	-	x	-
**ImmuNEO-14**	Melanoma	subcutaneous abdominal wall	Stage IV	MASTER	Yes	x	x	Yes	Yes	x	deceased	127	11	6	5	Yes	0	Yes	0	Yes	0
**ImmuNEO-15**	Testicle-Ca	lung	Stage IIICA	MASTER	Yes	Yes	Yes	Yes	Yes	Yes	alive	128	124	35	89	Yes	0	x	-	Yes	0
**ImmuNEO-16**	Adeno-Ca (Gl. sublingualis)	primary	Stage IV	MASTER	Yes	x	x	Yes	Yes	x	deceased	24	24	17	7	Yes	0	x	-	Yes	0
ImmuNEO-17.1	Melanoma	LN	Stage IV	IN Plus	x	x	x	Yes	x	x	alive	213	68	31	37	Yes	1	Yes	1	x	-
**ImmuNEO-17.2**		LN		IN Plus	Yes	Yes	x	Yes	Yes	Yes											
ImmuNEO-17.3		LN		IN Plus	x	x	x	Yes	Yes	Yes											
**ImmuNEO-18**	Mamma-Ca	ovar	Stage IV	IN Plus	Yes	Yes	x	Yes	Yes	Yes	deceased	62	62	22	40	x	-	x	-	x	-
ImmuNEO-19.1	Melanoma	LN colon	Stage IV	IN Plus	x	Yes	Yes	Yes	Yes	Yes	alive	30	30	29	1	Yes	2	x	-	Yes	2
ImmuNEO-19.2		colon		IN Plus	x	Yes	Yes	Yes	Yes	Yes											
ImmuNEO-19.3		colon		IN Plus	x	Yes	Yes	Yes	x	x											
**ImmuNEO-19.4**		liver		IN Plus	Yes	Yes	Yes	Yes	Yes	Yes											
**ImmuNEO-20**	Testicle-Ca	LN	Stage IIIB	MASTER	Yes	Yes	Yes	Yes	Yes	x	deceased	90	15	8	7	x	-	x	-	x	-
**ImmuNEO-22**	Melanoma	abdominal wall	Stage IV	MASTER	Yes	x	x	Yes	Yes*	Yes	alive	67	43	31	12	Yes	1	Yes	1	Yes	1
ImmuNEO-23.1	Sarcoma (MPNST)	LN	Stage IV	IN Plus	x	Yes	x	Yes	Yes	Yes	deceased	12	12	8	4	x	-	x	-	x	-
**ImmuNEO-23.2**		primary (thorax)		MASTER	Yes	Yes	Yes	Yes	Yes	Yes											
**ImmuNEO-24.1**	Adrenocortical-Ca	liver	Stage IV	IN Plus	Yes	Yes	Yes	Yes	Yes	Yes	alive	33	33	32	1	x	-	x	-	x	-
ImmuNEO-24.2		primary (kidney)		MASTER	x	Yes	Yes	Yes	Yes	Yes											
**ImmuNEO-25**	Sarcoma (GIST)	primary (intestine)	Stage IV	MASTER	Yes	Yes	Yes	Yes	Yes	x	alive	139	139	15	124	x	-	x	-	x	-
**ImmuNEO-26**	Adeno-Ca (mucoepidermoid)	primary	Stage IVA	MASTER	Yes	x	x	Yes	Yes	Yes	deceased	9	9	5	3	Yes	1	x	-	Yes	1
ImmuNEO-27.1	Fibrosarcoma (epitheloid)	primary	Stage IV	IN Plus	x	x	x	Yes	Yes	Yes	alive	35	27	26	1	x	-	x	-	x	-
**ImmuNEO-27.2**		lung		MASTER	Yes	x	x	Yes	Yes	Yes											
**ImmuNEO-28**	Clear cell sarcoma	primary	Stage IV	MASTER	Yes	Yes	x	Yes	Yes	Yes	alive	27	27	25	2	x	-	x	-	x	-
**ImmuNEO-30**	Synovial sarcoma	primary	Stage IV	MASTER	Yes	x	x	Yes	Yes*	Yes	alive	34	34	26	8	x	-	x	-	x	-
**ImmuNEO-31**	Rhabdomyosarcoma	primary	Stage IV	MASTER	Yes	x	x	Yes	Yes	x	deceased	15	15	14	1	x	-	x	-	x	-
**ImmuNEO-32**	Osteosarcoma	brain	Stage V-VI	MASTER	Yes	Yes	Yes	Yes	Yes	Yes	deceased	17	2	2	0	x	-	x	-	x	-
**ImmuNEO-33**	atypical carcinoid of the lung	asubcut. thorax	Stage IV	MASTER	Yes	x	x	Yes	Yes	Yes	deceased	18	18	5	13	x	-	x	-	x	-
**ImmuNEO-34**	Adeno-Ca (mucinous, appendix)	primary	Stage IV	MASTER	Yes	x	x	Yes	Yes	x	deceased	6	6	3	3	x	-	x	-	x	-
**ImmuNEO-35**	Fibrosarcoma (prostate)	*n.a.*	Stage IV	MASTER	Yes	Yes	x	Yes	Yes	Yes	alive	34	13	9	4	x	-	x	-	x	-
**ImmuNEO-36**	Adeno-Ca (Barret-Ca)	LN	AEG I; G3	MASTER	Yes	x	x	Yes	Yes	Yes	deceased	6	6	6	0	Yes	0	x	-	Yes	0
**ImmuNEO-37**	Adeno-Ca (appendix)	primary	Stage IVc	MASTER	Yes	Yes	x	Yes	Yes	Yes	deceased	16	16	4	12	x	-	x	-	x	-
**ImmuNEO-38**	Sarcoma (MPNST)	colon	Stage IV	MASTER	Yes	Yes	x	Yes	Yes	Yes	alive	64	34	13	21	x	-	x	-	x	-

Supplementary Table 1 | Overview of the ImmuNEO patient cohort. Detailed information on every tumor sample of the ImmuNEO cohort including entity, metastatic site (or primary), stage at admission and primary sampling cohort. Core samples used for statistical analysis (subset) are labelled. Tumor samples used for the immune phenotyping of the tumor microenvironment (TME) by flow cytometric assessment and RNA sequencing (RNA-seq) of sorted $CD8^+$ T cells are marked. Samples where whole exome sequencing (WES) and bulk tumor RNA-seq was performed are annotated; samples analysed via whole genome sequencing (WGS) are marked with an asterisk. The survival status as well as the survival times in months are displayed for several periods since initial diagnosis (ID), diagnosis of metastatic disease (MD) and since admission to MASTER/tumor resection (MASTER). The time difference since MD and MASTER is shown in months. Furthermore, information is given on patients receiving immune checkpoint blockade in general, prior to and after study admission and the respective response with no response (0), mixed response (1) and good response (2). Ca, carcinoma; DSRCT, desmoplastic small round cell tumor; MPNST, malignant peripheral nerve sheath tumor; GIST, gastrointestinal stromal tumor; LN, lymph node; IN, ImmuNEO; MS, mass spectometry; WES, whole exome sequencing; WGS, whole genome sequencing; RNAseq, RNA sequencing; IME, immune microenvironment.

	Therapy prior to sample extraction					Therapy after sample extraction				
ImmuNEO-ID	OP	x-Ray	Chemo	Targeted Therapy	Checkpoint Therapy	OP	x-Ray	Chemo	Targeted Therapy	Checkpoint Therapy
IN-01	0	0	1	0	0	0	0	0	0	1
IN-02	1	1	1	0	0	0	0	0	1	0
IN-03	1	0	1	1	0	0	0	1	1	0
IN-04	0	0	0	1	1	0	1	0	1	1
IN-05	1	0	1	0	0	0	0	0	0	0
IN-08	1	1	1	0	0	0	1	1	0	1
IN-09	0	0	0	0	0	0	1	0	1	0
IN-11	1	0	0	0	0	1	0	0	0	0
IN-13	1	0	1	0	0	0	0	1	0	0
IN-14	1	1	0	1	1	0	0	1	0	1
IN-15	1	1	1	0	0	1	0	1	1	1
IN-16	0	0	1	0	0	0	1	0	0	1
IN-17	0	1	0	0	1	0	1	0	0	0
IN-18	0	1	1	0	0	0	1	0	0	0
IN-19	0	0	0	0	0	1	0	0	0	1
IN-20	1	0	1	0	0	0	1	1	0	0
IN-22	1	1	0	0	1	0	0	1	1	1
IN-23	1	0	1	0	0	1	1	1	0	0
IN-24	0	0	1	0	0	1	0	1	1	0
IN-25	1	0	0	1	0	0	0	0	0	0
IN-26	1	1	1	0	0	0	0	1	0	1
IN-27	1	0	0	0	0	1	0	1	1	0
IN-28	0	0	0	0	0	0	0	0	0	0
IN-30	1	1	1	0	0	1	0	1	0	0
IN-31	0	0	1	0	0	0	1	1	0	0
IN-32	1	0	1	0	0	0	0	1	0	0
IN-33	1	1	1	0	0	0	0	0	0	0
IN-34	0	0	1	0	0	0	0	0	0	0
IN-35	1	1	1	1	0	0	0	1	1	0
IN-36	1	0	0	0	0	0	0	1	0	1
IN-37	1	0	1	1	0	1	0	1	1	0
IN-38	1	1	0	0	0	0	0	0	0	0

Supplementary Table 2 | Therapy overview ImmuNEO cohort. Information about applied therapies for every ImmuNEO patient prior to and after tumor resection. 1 = therapy applied, 0 = therapy not applied. IN, ImmuNEO; OP, operation; Chemo, chemotherapy.

Patient_ID:	HLA-A		HLA-B		HLA-C	
	(1)	(2)	(1)	(2)	(1)	(2)
ImmuNEO-01*	HLA-A0201	HLA-A2402	HLA-B4402	HLA-B4402	HLA-C0501	HLA-C0704
ImmuNEO-02	HLA-A0201	HLA-A1101	HLA-B3501	HLA-B4002	HLA-C0202	HLA-C0401
ImmuNEO-03	HLA-A0101	HLA-A2403	HLA-B3701	HLA-B5101	HLA-C0602	HLA-C1402
ImmuNEO-04*	HLA-A2301	HLA-A2402	HLA-B1501	HLA-B3801	HLA-C0602	HLA-C1203
ImmuNEO-05	HLA-A0301	HLA-A6601	HLA-B3701	HLA-B4102	HLA-C0602	HLA-C1703
ImmuNEO-08	HLA-A0301	HLA-A2601	HLA-B0702	HLA-B2705	HLA-C0102	HLA-C0702
ImmuNEO-09	HLA-A1101	HLA-A3201	HLA-B3503	HLA-B4006	HLA-C0401	HLA-C1502
ImmuNEO-11	HLA-A0201	HLA-A0301	HLA-B2705	HLA-B5201	HLA-C0102	HLA-C1202
ImmuNEO-13	HLA-A1101	HLA-A3101	HLA-B0801	HLA-B4402	HLA-C0501	HLA-C0701
ImmuNEO-14	HLA-A0301	HLA-A0301	HLA-B1801	HLA-B3801	HLA-C1203	HLA-C1203
ImmuNEO-15	HLA-A0201	HLA-A0301	HLA-B1501	HLA-B3701	HLA-C0401	HLA-C0602
ImmuNEO-16	HLA-A0301	HLA-A2301	HLA-B4001	HLA-B4901	HLA-C0304	HLA-C0701
ImmuNEO-17	HLA-A0201	HLA-A6802	HLA-B1402	HLA-B3906	HLA-C0702	HLA-C0802
ImmuNEO-18	HLA-A0301	HLA-A1101	HLA-B3501	HLA-B5701	HLA-C0401	HLA-C0602
ImmuNEO-19*	HLA-A0101	HLA-A2902	HLA-B3502	HLA-B4403	HLA-C0401	HLA-C1601
ImmuNEO-20	HLA-A0201	HLA-A0201	HLA-B5101	HLA-B5701	HLA-C0102	HLA-C0602
ImmuNEO-22*	HLA-A2902	HLA-A3201	HLA-B4402	HLA-B4403	HLA-C0501	HLA-C1601
ImmuNEO-23	HLA-A0301	HLA-A1101	HLA-B0702	HLA-B1803	HLA-C0701	HLA-C0702
ImmuNEO-24	HLA-A0206	HLA-A1101	HLA-B1525	HLA-B2704	HLA-C0702	HLA-C1202
ImmuNEO-25	HLA-A0101	HLA-A0301	HLA-B1801	HLA-B5101	HLA-C0701	HLA-C0602
ImmuNEO-26	HLA-A0201	HLA-A0201	HLA-B2705	HLA-B4402	HLA-C0202	HLA-C0501
ImmuNEO-27	HLA-A0101	HLA-A3301	HLA-B1402	HLA-B4002	HLA-C0202	HLA-C0802
ImmuNEO-28	HLA-A2301	HLA-A3001	HLA-B0702	HLA-B4403	HLA-C0401	HLA-C1203
ImmuNEO-30	HLA-A0101	HLA-A2601	N/A	HLA-B3701	N/A	HLA-C0602
ImmuNEO-31	HLA-A2902	HLA-A3201	HLA-B4002	HLA-B4501	HLA-C0202	HLA-C0602
ImmuNEO-32	HLA-A0101	HLA-A1101	HLA-B0702	HLA-B5601	HLA-C0102	HLA-C0702
ImmuNEO-33	HLA-A0201	HLA-A2601	HLA-B4102	HLA-B5201	HLA-C1202	HLA-C1701
ImmuNEO-34	HLA-A0101	HLA-A3201	HLA-B0801	HLA-B4002	HLA-C0202	HLA-C0701
ImmuNEO-35	HLA-A0201	HLA-A0301	HLA-B0702	HLA-B4001	HLA-C0304	HLA-C0702
ImmuNEO-36	HLA-A0201	HLA-A0301	HLA-B1402	HLA-B3503	HLA-C0401	HLA-C0802
ImmuNEO-37	HLA-A0301	HLA-A6801	HLA-B1801	HLA-B5101	HLA-C0701	HLA-C1504
ImmuNEO-38	HLA-A1101	HLA-A2601	HLA-B0702	HLA-B5101	HLA-C0102	HLA-C0702

Supplementary Table 3 | Overview of HLA class I alleles of ImmuNEO patients. Table providing information on HLA class I alleles identified for each patient from whole exome (WES)/whole genome sequencing (WGS) data using the combination of the algorithms xHLA, BWAKit, and OptiType. For ImmuNEO-1, -4, -19 and -22 (*) the alleles were confirmed using targeted NGS (Zentrum für Humangenetik und Laboratoriumsdiagnostik, Martinsried, Germany). HLA, human leukocyte antigen; N/A, not available.

	number of peptides assigned to respective HLA binding motif													
Patient ID	HLA-A allele 1	number of assigned peptides	HLA-A allele 2	number of assigned peptides	HLA-B allele 1	number of assigned peptides	HLA-B allele 2	number of assigned peptides	HLA-C allele 1	number of assigned peptides	HLA-C allele 2	number of assigned peptides	total assigened peptides	number of peptides not assigned	total assigend peptides [%]
IN-01	HLA-A0201	1422	HLA-A2402	780	HLA-B4402	3505	-	-	HLA-C0501	941	HLA-C0704	177	6825	1291	84.1%
IN-02	HLA-A0201	392	HLA-A1101	3578	HLA-B3501	470	HLA-B4002	1369	HLA-C0202	206	HLA-C0401	78	6093	238	96.2%
IN-03	HLA-A0101	1066	HLA-A2403	334	HLA-B3701	1274	HLA-B5101	1272	HLA-C0602	362	HLA-C1402	447	4755	312	93.8%
IN-04	HLA-A2301	560	HLA-A2402	1009	HLA-B1501	7156	HLA-B3801	4108	HLA-C0602	1125	HLA-C1203	1277	15235	748	95.3%
IN-05	HLA-A0301	1958	HLA-A6601	2610	HLA-B3701	843	HLA-B4102	2214	HLA-C0602	192	HLA-C1703	113	7930	135	98.3%
IN-08	HLA-A0301	1791	HLA-A2601	433	HLA-B0702	1463	HLA-B2705	765	HLA-C0102	172	HLA-C0702	51	4675	107	97.8%
IN-09	HLA-A1101	1383	HLA-A3201	13	HLA-B3503	154	HLA-B4006	200	HLA-C0401	n/a	HLA-C1502	33	1783	20	98.9%
IN-11	HLA-A0201	1619	HLA-A0301	7403	HLA-B2705	4416	HLA-B5201	2944	HLA-C0102	960	HLA-C1202	822	18164	411	97.8%
IN-13	HLA-A1101	4145	HLA-A3101	3395	HLA-B0801	1630	HLA-B4402	1457	HLA-C0501	342	HLA-C0701	60	11029	102	99.1%
IN-14	HLA-A0301	3505	-	-	HLA-B1801	251	HLA-B3801	1025	HLA-C1203	214	-	-	4995	35	99.3%
IN-15	HLA-A0201	93	HLA-A0301	1752	HLA-B1501	479	HLA-B3701	309	HLA-C0401	16	HLA-C0602	95	2744	40	98.6%
IN-16	HLA-A0301	906	HLA-A2301	20	HLA-B4001	200	HLA-B4901	437	HLA-C0304	54	HLA-C0701	11	1628	33	98.0%
IN-17	HLA-A0201	695	HLA-A6802	1689	HLA-B1402	2297	HLA-B3906	4584	HLA-C0702	119	HLA-C0802	704	10088	235	97.7%
IN-18	HLA-A0301	1138	HLA-A1101	2513	HLA-B3501	221	HLA-B5701	108	HLA-C0401	28	HLA-C0602	131	4139	147	96.6%
IN-19	HLA-A0101	5241	HLA-A2902	3512	HLA-B3502	5493	HLA-B4403	13533	HLA-C0401	942	HLA-C1601	3098	31819	1596	95.2%
IN-20	HLA-A0201	1034	-	-	HLA-B5101	791	HLA-B5701	481	HLA-C0102	325	HLA-C0602	307	2938	430	87.2%
IN-22	HLA-A2902	138	HLA-A3201	116	HLA-B4402	992	HLA-B4403	508	HLA-C0501	170	HLA-C1601	282	2206	45	98.0%
IN-23	HLA-A0301	1365	HLA-A1101	2346	HLA-B0702	1259	HLA-B1803	255	HLA-C0701	67	HLA-C0702	82	5374	270	95.2%
IN-24	HLA-A0206	603	HLA-A1101	6606	HLA-B1525	1625	HLA-B2704	682	HLA-C0702	123	HLA-C1202	547	10186	270	97.4%
IN-25	HLA-A0101	425	HLA-A0301	1888	HLA-B1801	337	HLA-B5101	427	HLA-C0701	132	HLA-C0602	21	3230	23	99.3%
IN-26	HLA-A0201	350	-	-	HLA-B2705	1142	HLA-B4402	399	HLA-C0202	59	HLA-C0501	124	2074	78	96.4%
IN-27	HLA-A0101	649	HLA-A3301	2375	HLA-B1402	987	HLA-B4002	1588	HLA-C0202	85	HLA-C0802	209	5893	152	97.5%
IN-28	HLA-A2301	390	HLA-A3001	1536	HLA-B0702	2428	HLA-B4403	2420	HLA-C0401	174	HLA-C1203	336	7284	84	98.9%
IN-30	HLA-A0101	368	HLA-A2601	319	HLA-B3701	245	-	-	HLA-C0602	99	-	-	1031	35	96.7%
IN-31	HLA-A2902	29	HLA-A3201	13	HLA-B4002	58	HLA-B4501	188	HLA-C0202	n/a	HLA-C0602	21	309	29	91.4%
IN-32	HLA-A0101	194	HLA-A1101	2833	HLA-B0702	344	HLA-B5601	774	HLA-C0102	146	HLA-C0702	n/a	4291	18	99.6%
IN-33	HLA-A0201	46	HLA-A2601	111	HLA-B4102	191	HLA-B5201	62	HLA-C1202	35	HLA-C1701	n/a	445	48	90.3%
IN-34	HLA-A0101	405	HLA-A3201	178	HLA-B0801	1055	HLA-B4002	975	HLA-C0202	91	HLA-C0701	45	2749	92	96.8%
IN-35	HLA-A0201	24	HLA-A0301	176	HLA-B0702	64	HLA-B4001	69	HLA-C0304	26	HLA-C0702	n/a	359	10	97.3%
IN-36	HLA-A0201	345	HLA-A0301	2353	HLA-B1402	992	HLA-B3503	441	HLA-C0401	40	HLA-C0802	158	4329	62	98.6%
IN-37	HLA-A0301	2214	HLA-A6801	3534	HLA-B1801	402	HLA-B5101	503	HLA-C0701	45	HLA-C1504	256	6954	85	98.8%
IN-38	HLA-A1101	3689	HLA-A2601	1140	HLA-B0702	2181	HLA-B5101	1304	HLA-C0102	571	HLA-C0702	160	9045	254	97.3%

Supplementary Table 4 | Number of peptides assigned to each HLA class I binding motifs within the immunopeptidome. MHCMotifDecon (v1.0) has been used to match all isolated HLA class I peptides with lengths from 8-15 amino acids to the patients' HLA class I alleles (see Supplementary Table 3) according to their binding motifs and anchor residues for each tumor sample using standard settings. The total number of peptides assigned to each HLA allele is shown, as well as the number peptides not matching any HLA class I allele of the respective patient. The percentage of assigned peptides over all analysed peptides is given for each patient. HLA, human leukocyte antigen; n/a, not available.
