# Supplementary material for: Proteogenomic analysis reveals RNA as a source for tumor-agnostic neoantigen identification
Source: Nat Commun. 2023 Aug 2;14:4632. doi: 10.1038/s41467-023-39570-7 (PMC10397250; doi:10.1038/s41467-023-39570-7)
Supplement: Supplementary file 3 — Description of Additional Supplementary Files [file 41467_2023_39570_MOESM3_ESM.pdf]

Description of Additional Supplementary Files

Supplementary Data 1:
Shared somatic variants. Table showing the somatic variants present in min. 4 patients. Variants were called by MuTect2 (v4.1.0.0) from whole exome (WES)/whole genome sequencing (WGS) data and by Strelka2 (v2.9.10) from RNA sequencing (RNA-seq) data. SNP-filtering has been performed using the dbSNP-all data base. No RNA data was available for patients IN-11-T1, IN-14, IN-16, IN-20, IN-25, IN-31, IN-34. For every variant (Mutation_ID consist of chromosome, position, reference base and alternative base) the affected gene and the gene biotype are shown. The samples and patients where the variant is present is shown and counted (upper table). In the lower table all information for each variant within each samples is listed. The number of wt reads (TumorRD) and mutated reads (TumorAD) in the tumor, the wt reads (NormalRD) and mutated reads (NormalAD) in the normal control, the variant frequency within the tumor (TumorVF) and the coverage of each mutation within the tumor (TumorCoverage) is included. IN, ImmuNEO.

Supplementary Data 2:
Shared RNA alterations. Table showing the RNA alterations present in min. 10 patients. Variants were called by MuTect2 (v4.1.0.0) from whole exome (WES)/whole genome sequencing (WGS) data and by Strelka2 (v2.9.10) from RNA sequencing (RNA-seq) data. SNP-filtering has been performed using the dbSNP-all data base. No RNA data was available for patients IN-11-T1, IN-14, IN-16, IN-20, IN-25, IN-31, IN-34. For every variant (Mutation_ID consist of chromosome, position, reference base and alternative base) the affected gene and the gene biotype are shown. The samples and patients where the variant is present is shown and counted (upper table). In the lower table all information for each variant within each samples is listed. The number of wt reads (TumorRD) and mutated reads (TumorAD) in the tumor, the wt reads (NormalRD) and mutated reads (NormalAD) in the normal control, the variant frequency within the tumor (TumorVF) and the coverage of each mutation within the tumor (TumorCoverage) is included. IN, ImmuNEO.

Supplementary Data 3:
Shared RNA alterations by groups shown in Supplementary Figure 5f. Table showing the RNA alterations (min. 2 in each group) present in min. 10 unique samples. Variants were called by MuTect2 (v4.1.0.0) from whole exome (WES)/whole genome sequencing (WGS) data and by Strelka2 (v2.9.10) from RNA sequencing (RNA-seq) data. SNP-filtering has been performed using the dbSNP-all data base. No RNA data was available for patients IN-11-T1, IN-14, IN-16, IN-20, IN-25, IN-31, IN-34. For every variant (Mutation_ID consist of chromosome, position, reference base and alternative base) the affected gene and the gene biotype are shown. The samples and patients where the variant is present is shown and counted (upper table). In the lower table all information for each variant within each samples is listed. The number of wt reads (TumorRD) and mutated reads (TumorAD) in the tumor, the wt reads (NormalRD) and mutated reads (NormalAD) in the normal control, the variant frequency within the tumor (TumorVF) and the coverage of each mutation within the tumor (TumorCoverage) is included. IN, ImmuNEO.

Supplementary Data 4:
Detailed information on all neoantigen candidates. By combining genomic mutational data with mass-spectrometry (MS)-based immunopeptidomic data for each patient sample, neoantigen candidates have been identified. pFIND (v3.1.5) was used at 5% FDR on spectral level for the identification of non-canonical 8-15mer neoantigen candidates. The machine learning tool Prosit was additionally integrated to rescore and rematch the peptide spectra using unfiltered pFIND data as input. n = 39 tumor samples from n = 32 patients were analysed in total; n = 27 tumor samples from n = 24 patients harboured n = 90 neoantigen candidates. Using netMHC4.0 and MHCFlurry, binding predictions for each peptide towards the patients six HLA class I alleles (see Suppl. Table S1a) has been performed and for each algorithm the best binding allele by affinity and by rank are shown. Mutated amino acids are marked with two asterisks within the sequence and the variant location is annotated in 5' to 3' direction. Additional information for each peptide and variant are given such as the result from BLAT analysis, GTEx prevalence of the variant, peptide verification data (SA and RT errors) as well as the immunogenicity of the peptide defined by acDC (see Figure 6). a.a, amino acid; Alt, alternative; BA, binding affinity; CA, carcinoma; Chrom, chromosome; del, deletion; dup, duplication; HD, healthy donor; HLA, human leukocyte antigen; ins, insertion; n.a./NA, not applicable; nM nanomole; PBMC, peripheral blood mononbuclear cells; Pos, position; PSM, peptide-spectrum match; Ref, reference; RT, retention time; SA, spectral contrast angle; Seq, sequence; T, tumor; VF, variant frequency.

Supplementary Data 5:
Mirror plot visualization of the matching endogenous MS2 spectra, synthetic MS2 spectra and predicted fragment ions per peptide sequence.
