# Supplementary material for: Proteogenomic analysis reveals RNA as a source for tumor-agnostic neoantigen identification
Source: Nat Commun. 2023 Aug 2;14:4632. doi: 10.1038/s41467-023-39570-7 (PMC10397250; doi:10.1038/s41467-023-39570-7)
Supplement: Supplementary file 8 — Supplementary Data 5 [file 41467_2023_39570_MOESM8_ESM.pdf]

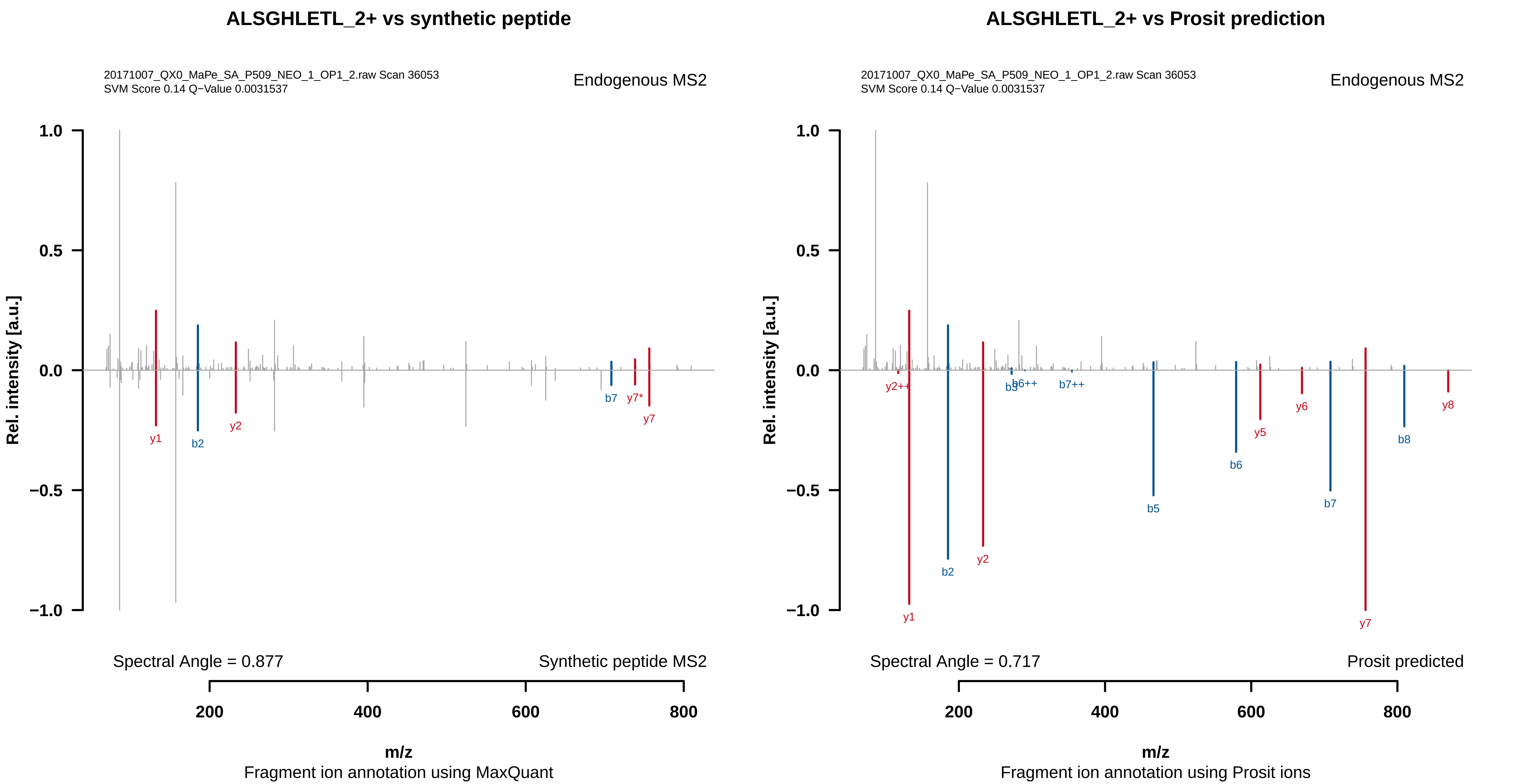
ALSGHLETL_2+ vs synthetic peptide
20171007_QX0_MaPe_SA_P509_NEO_1_OP1_2.raw Scan 36053
SVM Score 0.14 Q−Value 0.0031537
Endogenous MS2
1.0
0.5
0.0
−0.5
−1.0
Rel. intensity [a.u.]
y1
b2
y2
b7
y7*
y7
Spectral Angle = 0.877
Synthetic peptide MS2
200
400
600
800
m/z
Fragment ion annotation using MaxQuant
ALSGHLETL_2+ vs Prosit prediction
20171007_QX0_MaPe_SA_P509_NEO_1_OP1_2.raw Scan 36053
SVM Score 0.14 Q−Value 0.0031537
Endogenous MS2
1.0
0.5
0.0
−0.5
−1.0
Rel. intensity [a.u.]
y2++
b3
b6++
b7++
y1
b2
y2
b5
b6
y5
y6
b7
y7
b8
y8
Spectral Angle = 0.717
Prosit predicted
200
400
600
800
m/z
Fragment ion annotation using Prosit ions

ALSGHLETL_2+ vs synthetic peptide

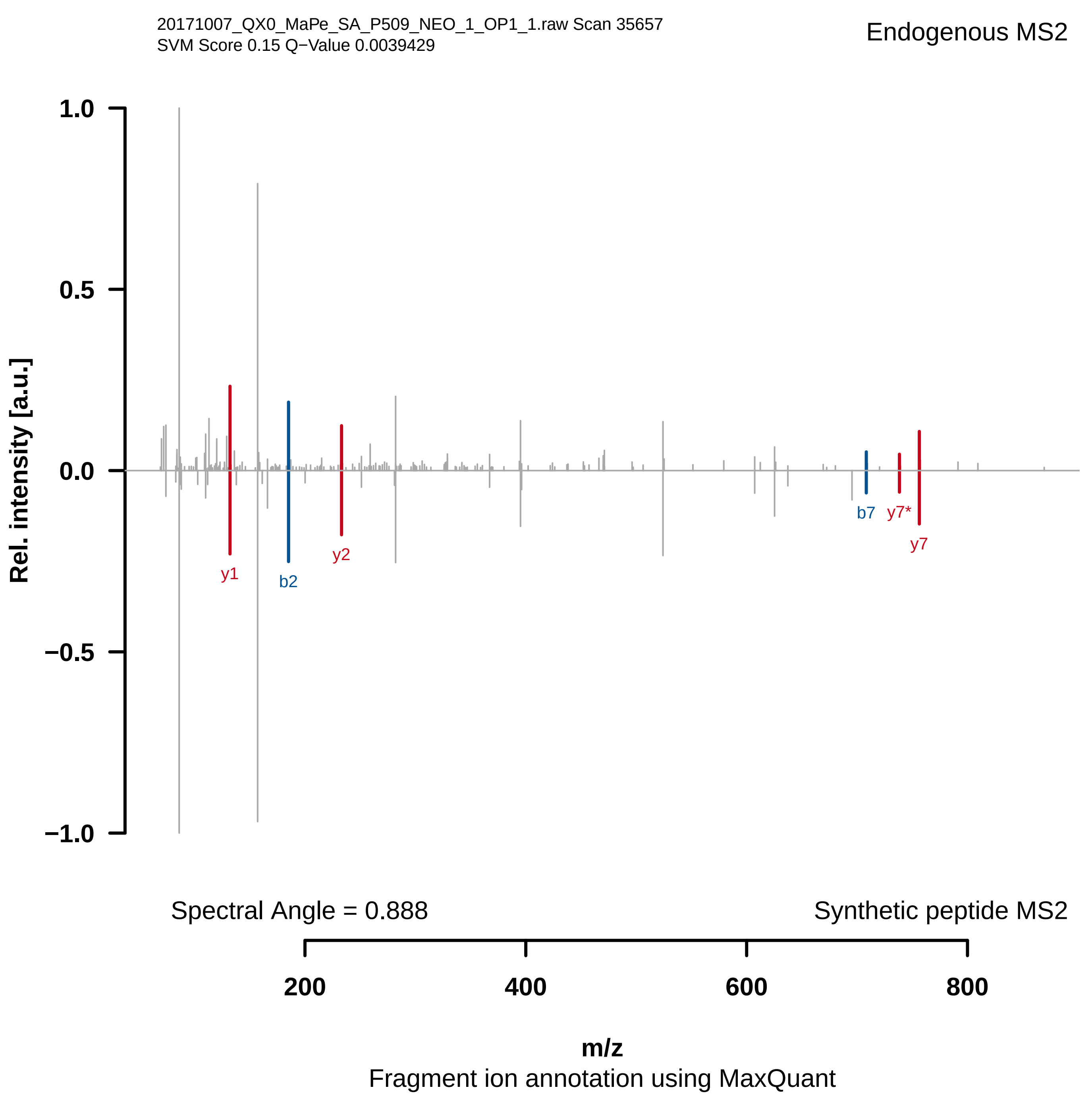

ALSGHLETL_2+ vs Prosit prediction

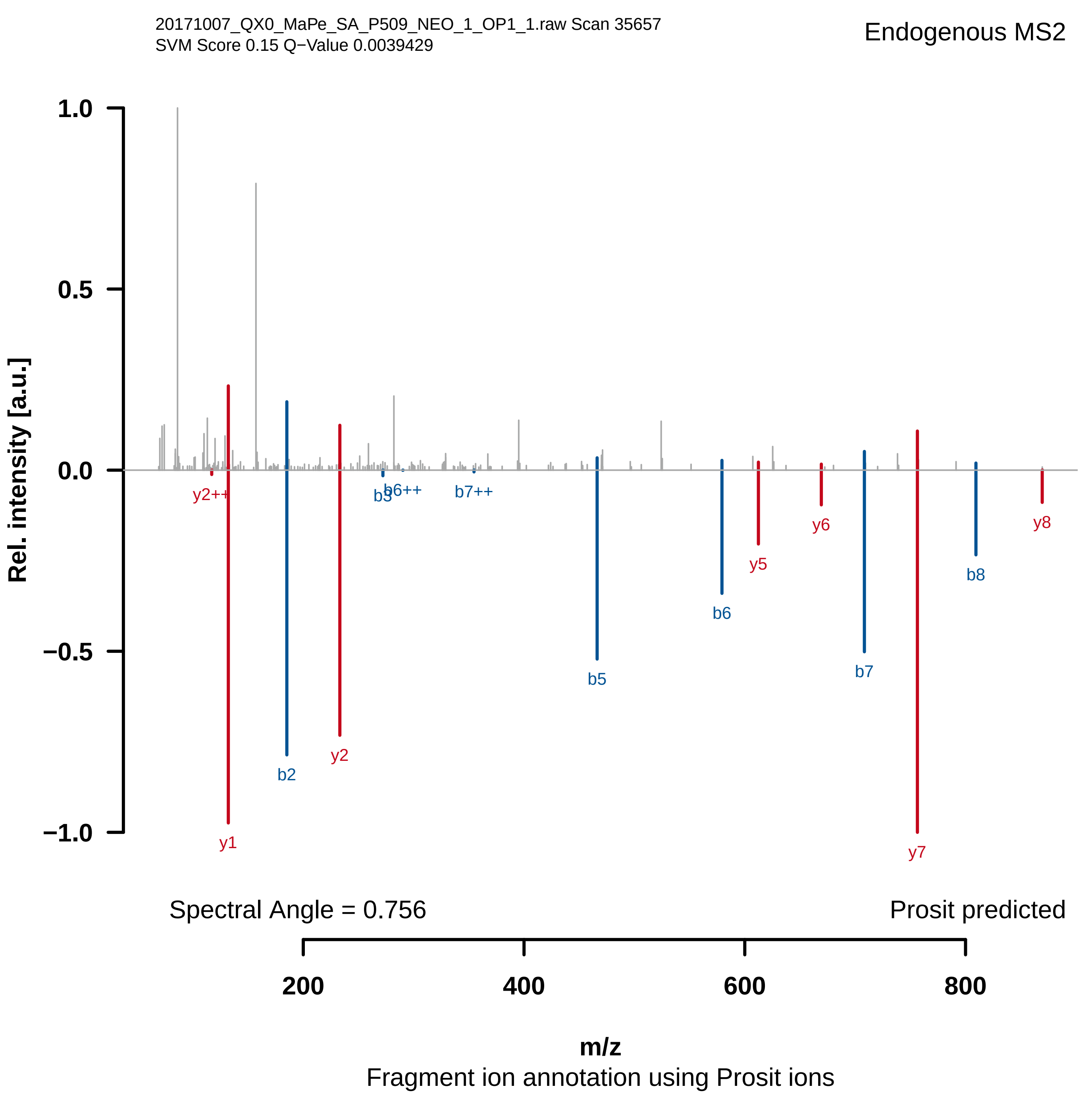

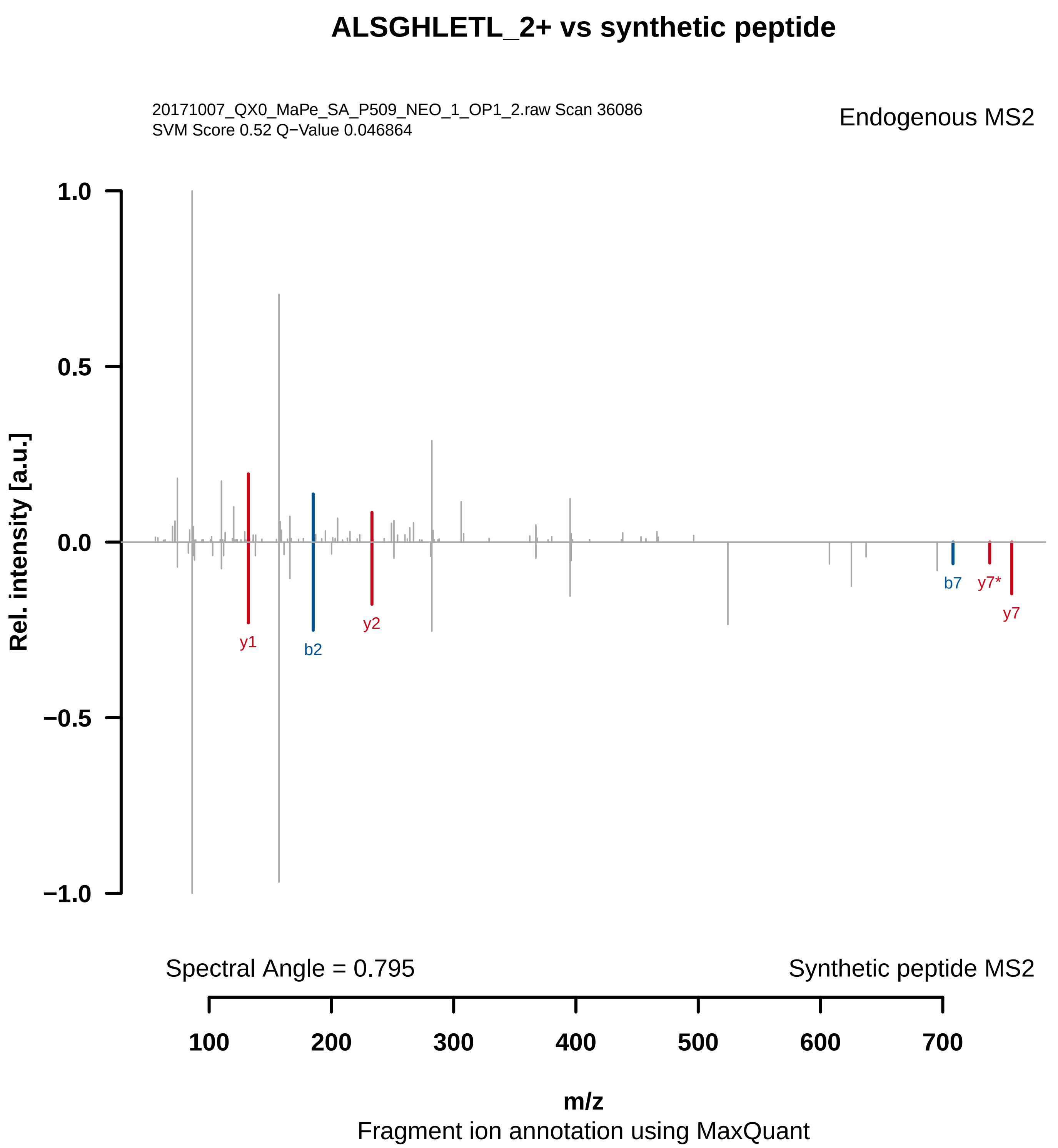

ALSGHLETL_2+ vs synthetic peptide
20171007_QX0_MaPe_SA_P509_NEO_1_OP1_2.raw Scan 36086
SVM Score 0.52 Q−Value 0.046864
Endogenous MS2
Rel. intensity [a.u.]
1.0
0.5
0.0
−0.5
−1.0
y1
b2
y2
b7
y7*
y7
Spectral Angle = 0.795
Synthetic peptide MS2
100
200
300
400
500
600
700
m/z
Fragment ion annotation using MaxQuant

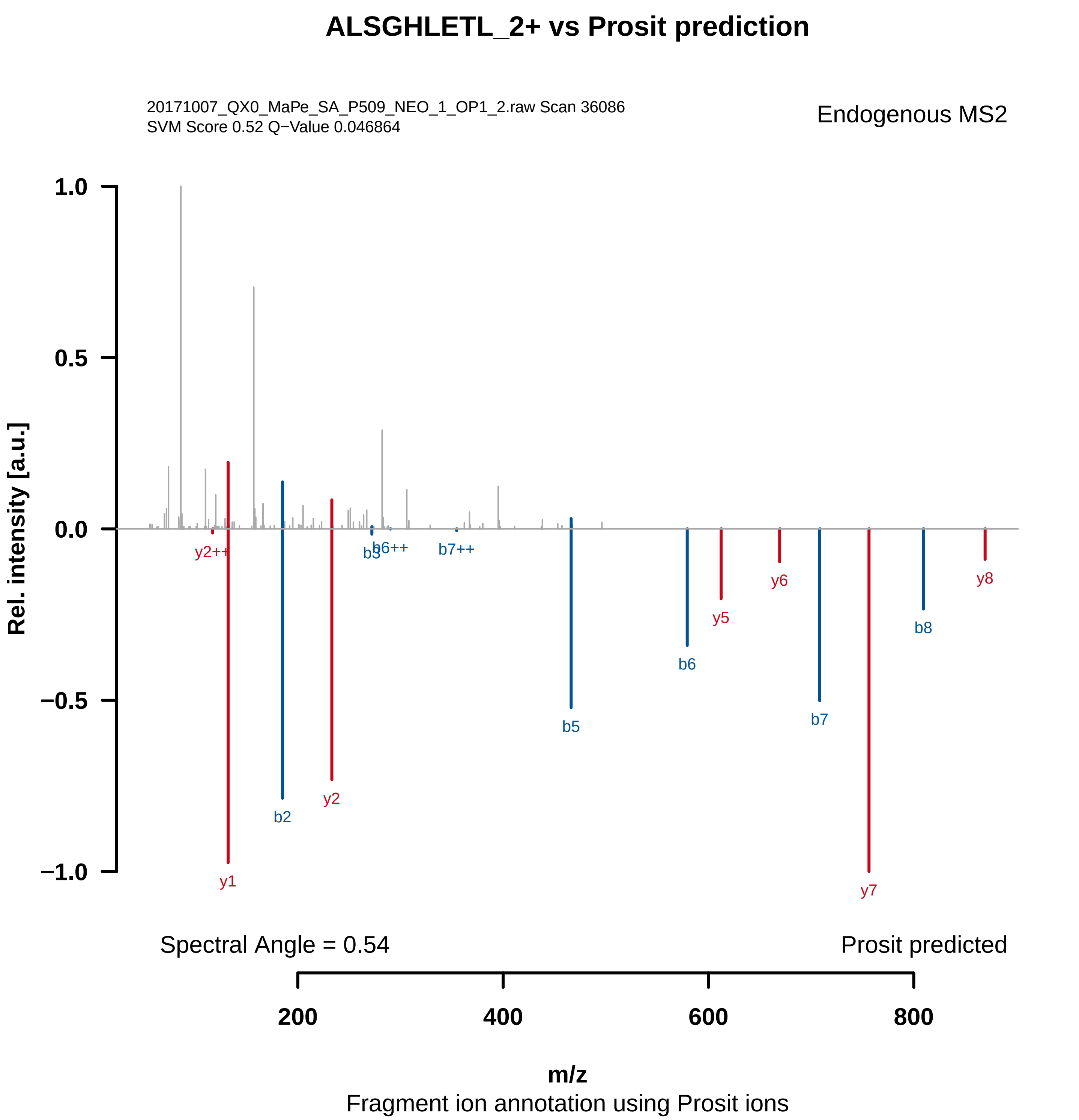

ALSGHLETL_2+ vs Prosit prediction
20171007_QX0_MaPe_SA_P509_NEO_1_OP1_2.raw Scan 36086
SVM Score 0.52 Q−Value 0.046864
Endogenous MS2
Rel. intensity [a.u.]
1.0
0.5
0.0
−0.5
−1.0
y2++
b3
b6++
b7++
y1
b2
y2
b5
b6
y5
y6
b7
y7
b8
y8
Spectral Angle = 0.54
Prosit predicted
200
400
600
800
m/z
Fragment ion annotation using Prosit ions

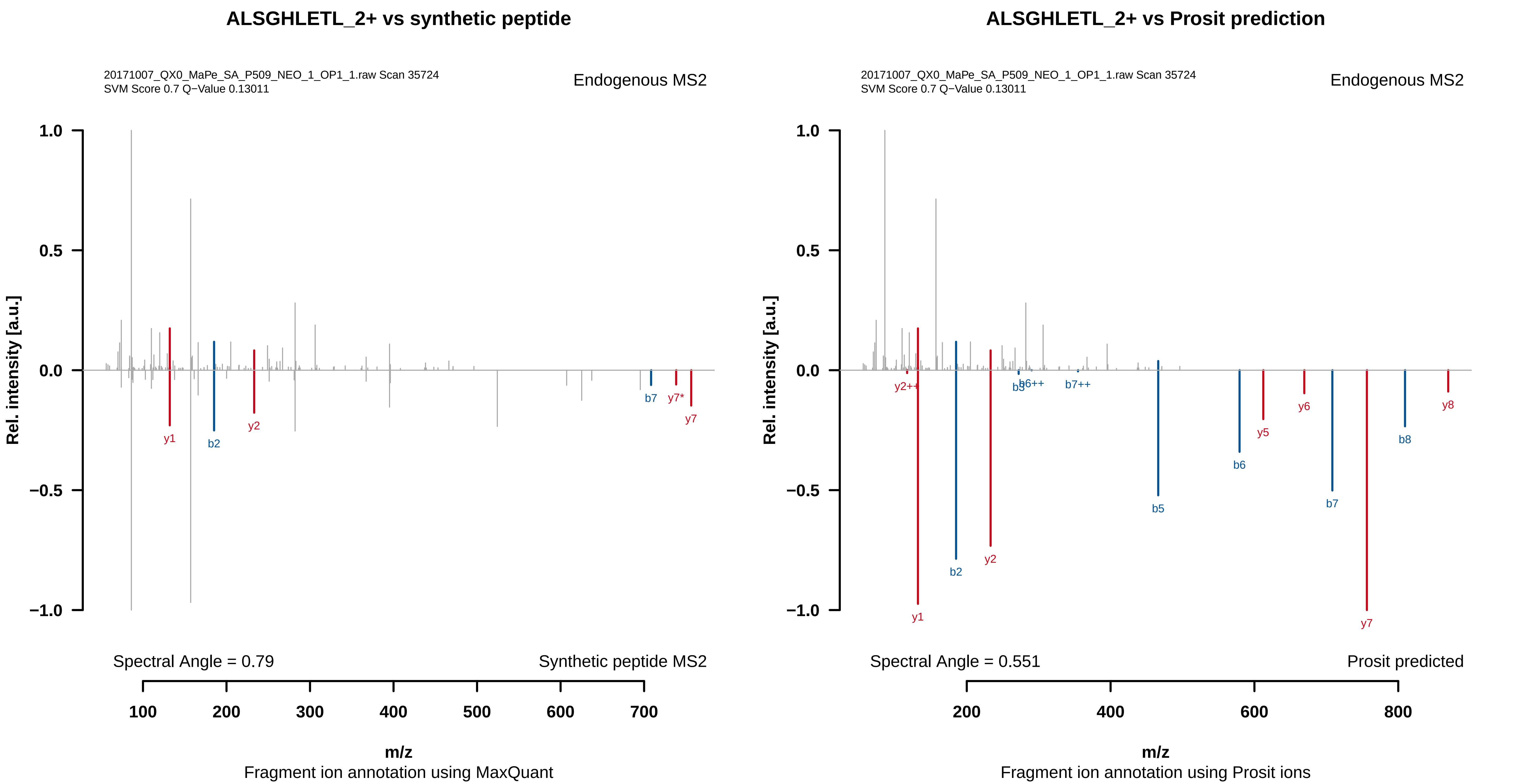

ALSGHLETL_2+ vs synthetic peptide
20171007_QX0_MaPe_SA_P509_NEO_1_OP1_1.raw Scan 35724
SVM Score 0.7 Q−Value 0.13011
Endogenous MS2
Rel. intensity [a.u.]
1.0
0.5
0.0
−0.5
−1.0
y1
b2
y2
b7
y7*
y7
Spectral Angle = 0.79
Synthetic peptide MS2
100
200
300
400
500
600
700
m/z
Fragment ion annotation using MaxQuant
ALSGHLETL_2+ vs Prosit prediction
20171007_QX0_MaPe_SA_P509_NEO_1_OP1_1.raw Scan 35724
SVM Score 0.7 Q−Value 0.13011
Endogenous MS2
Rel. intensity [a.u.]
1.0
0.5
0.0
−0.5
−1.0
y2++
b3
b6++
b7++
y1
b2
y2
b5
b6
y5
y6
b7
y7
b8
y8
Spectral Angle = 0.551
Prosit predicted
200
400
600
800
m/z
Fragment ion annotation using Prosit ions

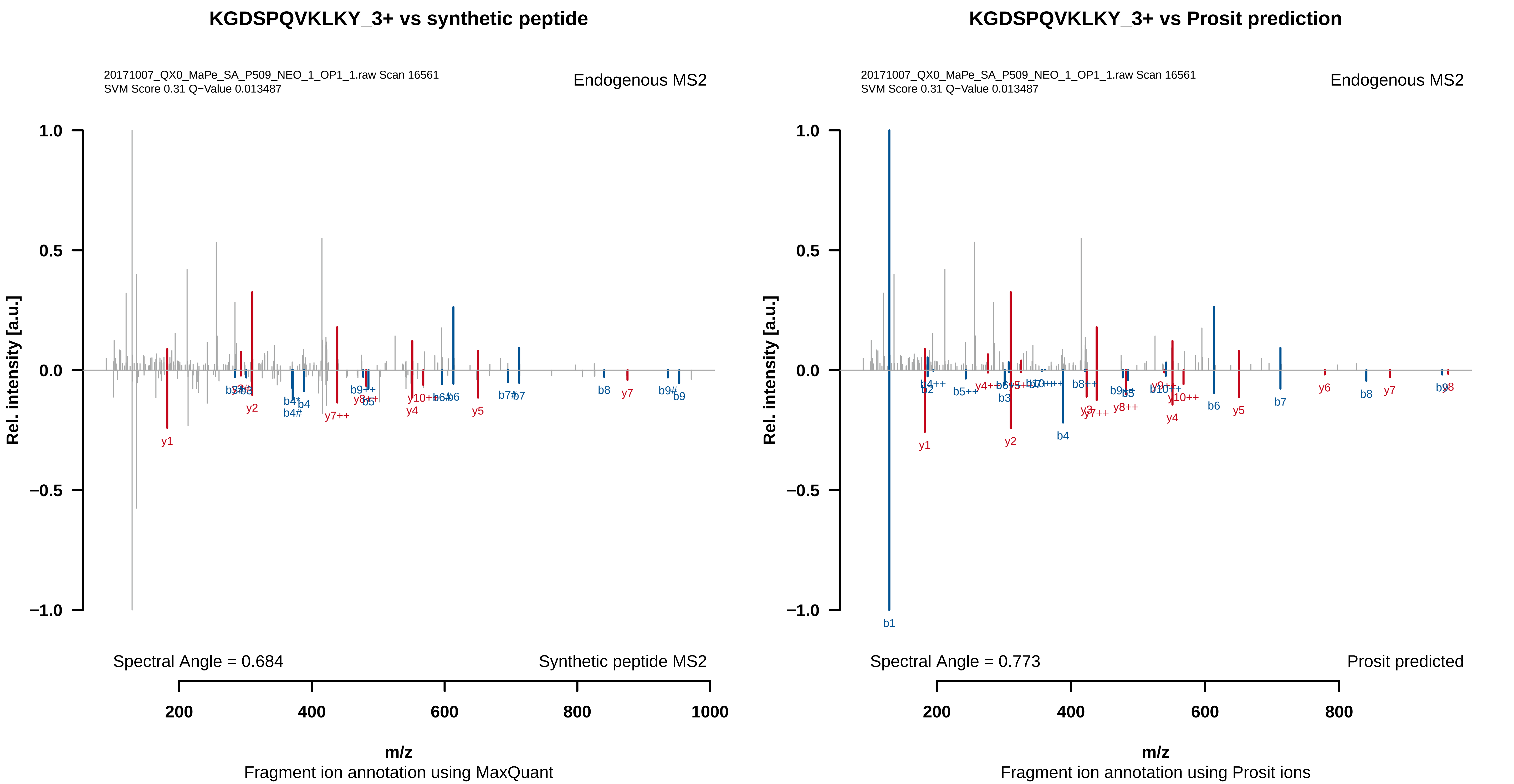
KGDSPQVKLKY_3+ vs synthetic peptide
20171007_QX0_MaPe_SA_P509_NEO_1_OP1_1.raw Scan 16561
SVM Score 0.31 Q−Value 0.013487
Endogenous MS2
Rel. intensity [a.u.]
Spectral Angle = 0.684
Synthetic peptide MS2
m/z
Fragment ion annotation using MaxQuant
KGDSPQVKLKY_3+ vs Prosit prediction
20171007_QX0_MaPe_SA_P509_NEO_1_OP1_1.raw Scan 16561
SVM Score 0.31 Q−Value 0.013487
Endogenous MS2
Rel. intensity [a.u.]
Spectral Angle = 0.773
Prosit predicted
m/z
Fragment ion annotation using Prosit ions

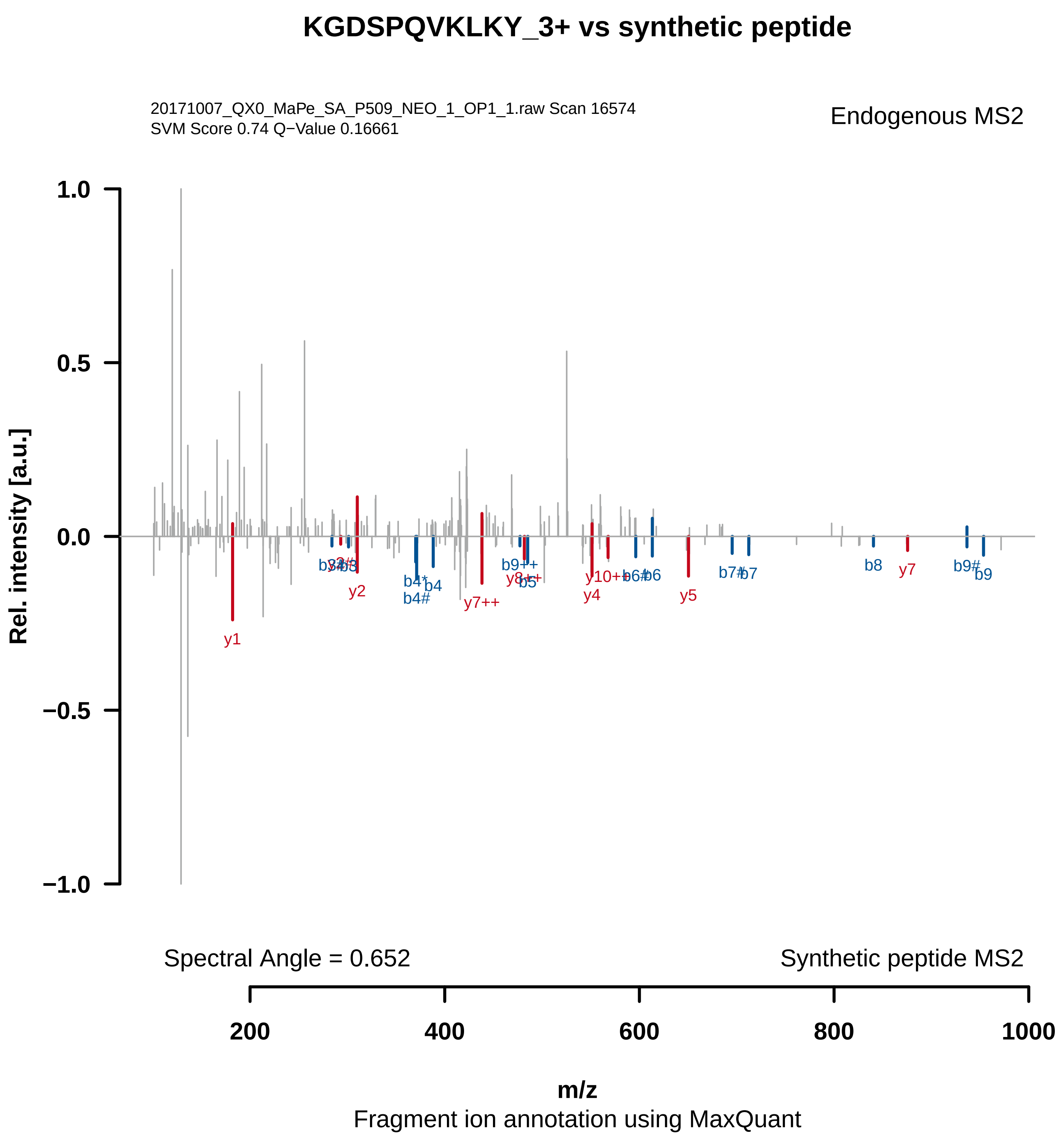
KGDSPQVKLKY_3+ vs synthetic peptide
20171007_QX0_MaPe_SA_P509_NEO_1_OP1_1.raw Scan 16574
SVM Score 0.74 Q−Value 0.16661
Endogenous MS2
Rel. intensity [a.u.]
1.0
0.5
0.0
−0.5
−1.0
Spectral Angle = 0.652
Synthetic peptide MS2
200
400
600
800
1000
m/z
Fragment ion annotation using MaxQuant

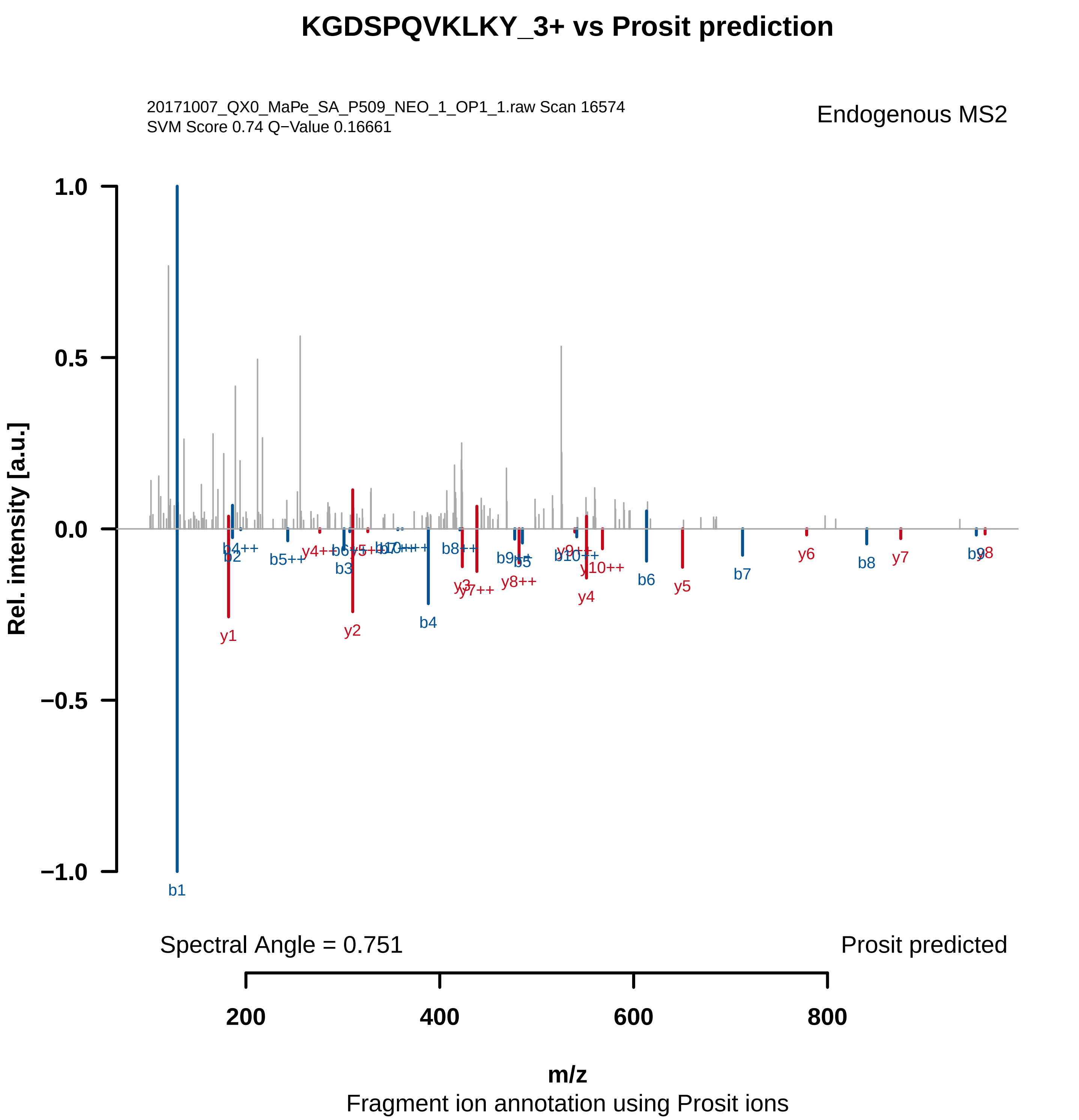
KGDSPQVKLKY_3+ vs Prosit prediction
20171007_QX0_MaPe_SA_P509_NEO_1_OP1_1.raw Scan 16574
SVM Score 0.74 Q−Value 0.16661
Endogenous MS2
Rel. intensity [a.u.]
1.0
0.5
0.0
−0.5
−1.0
Spectral Angle = 0.751
Prosit predicted
200
400
600
800
m/z
Fragment ion annotation using Prosit ions

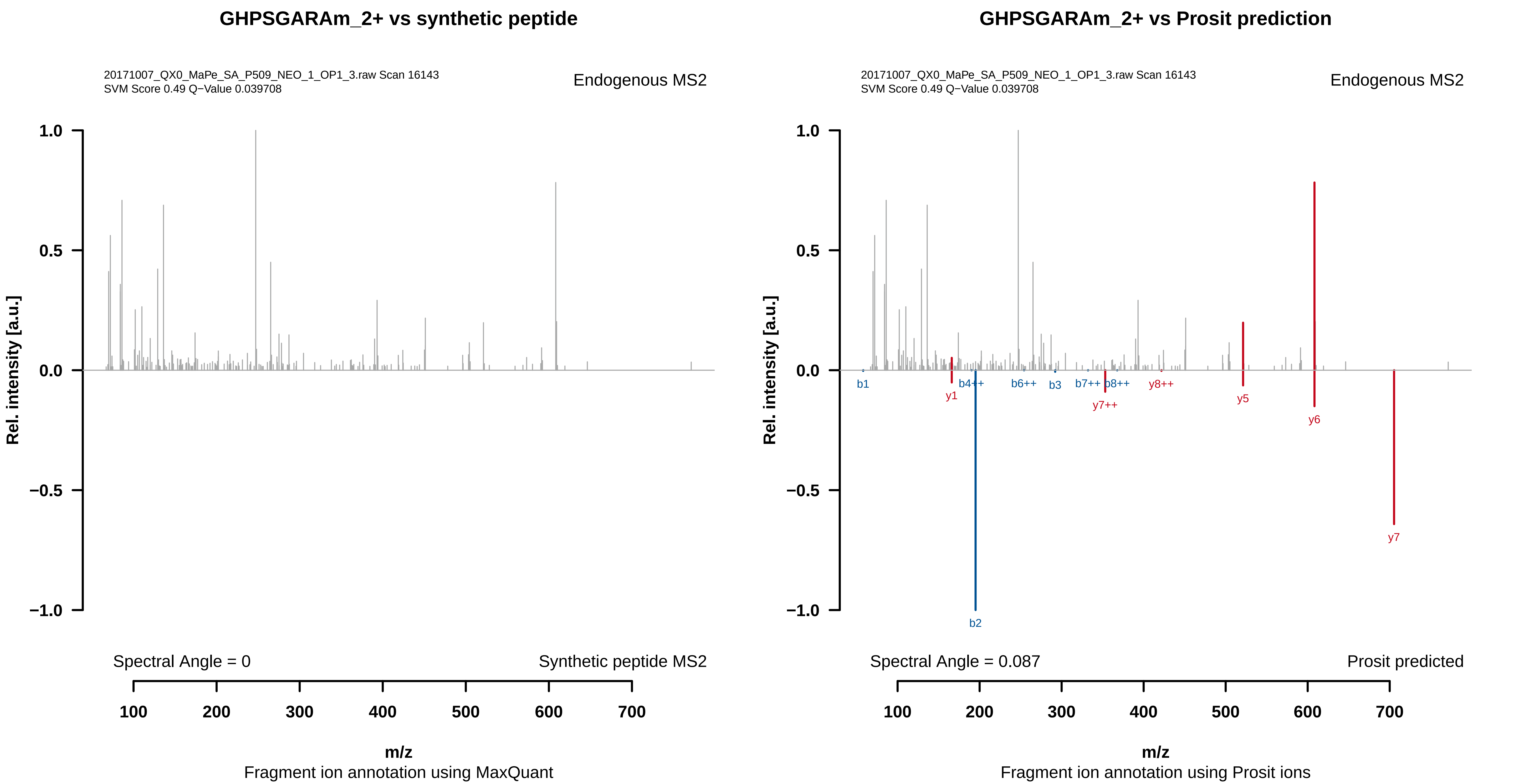

GHPSGARAm_2+ vs synthetic peptide
20171007_QX0_MaPe_SA_P509_NEO_1_OP1_3.raw Scan 16143
SVM Score 0.49 Q−Value 0.039708
Endogenous MS2
Rel. intensity [a.u.]
1.0
0.5
0.0
−0.5
−1.0
Spectral Angle = 0
Synthetic peptide MS2
100
200
300
400
500
600
700
m/z
Fragment ion annotation using MaxQuant
GHPSGARAm_2+ vs Prosit prediction
20171007_QX0_MaPe_SA_P509_NEO_1_OP1_3.raw Scan 16143
SVM Score 0.49 Q−Value 0.039708
Endogenous MS2
Rel. intensity [a.u.]
1.0
0.5
0.0
−0.5
−1.0
b1
y1
b4++
b6++
b3
b7++
b8++
y7++
y8++
y5
y6
y7
b2
Spectral Angle = 0.087
Prosit predicted
100
200
300
400
500
600
700
m/z
Fragment ion annotation using Prosit ions

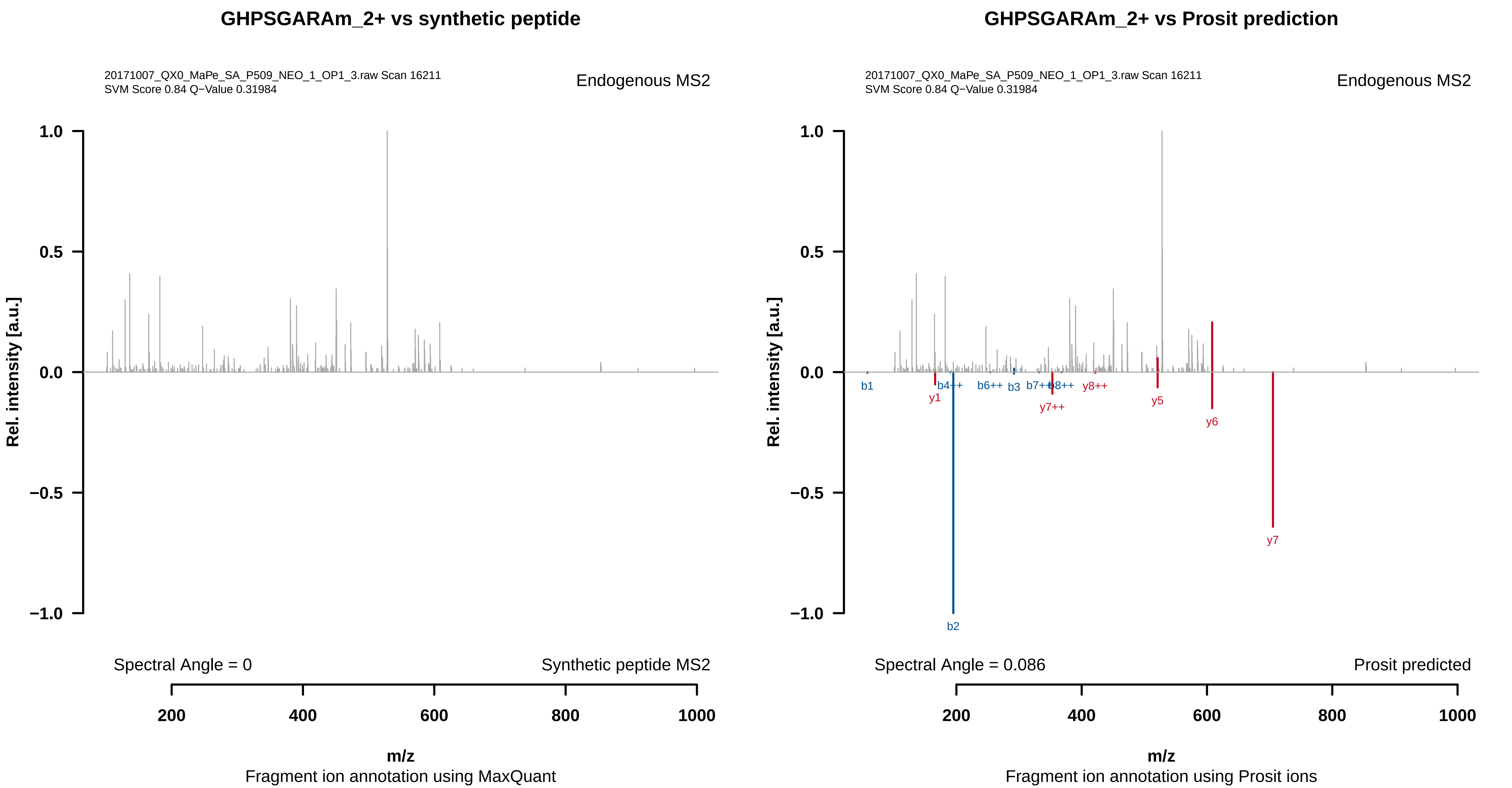
GHPSGARAm_2+ vs synthetic peptide
20171007_QX0_MaPe_SA_P509_NEO_1_OP1_3.raw Scan 16211
SVM Score 0.84 Q−Value 0.31984
Endogenous MS2
Rel. intensity [a.u.]
1.0
0.5
0.0
−0.5
−1.0
Spectral Angle = 0
Synthetic peptide MS2
200
400
600
800
1000
m/z
Fragment ion annotation using MaxQuant
GHPSGARAm_2+ vs Prosit prediction
20171007_QX0_MaPe_SA_P509_NEO_1_OP1_3.raw Scan 16211
SVM Score 0.84 Q−Value 0.31984
Endogenous MS2
Rel. intensity [a.u.]
1.0
0.5
0.0
−0.5
−1.0
b1
y1
b4++
b6++
b3
b7++
b8++
y8++
y7++
y5
y6
y7
b2
Spectral Angle = 0.086
Prosit predicted
200
400
600
800
1000
m/z
Fragment ion annotation using Prosit ions

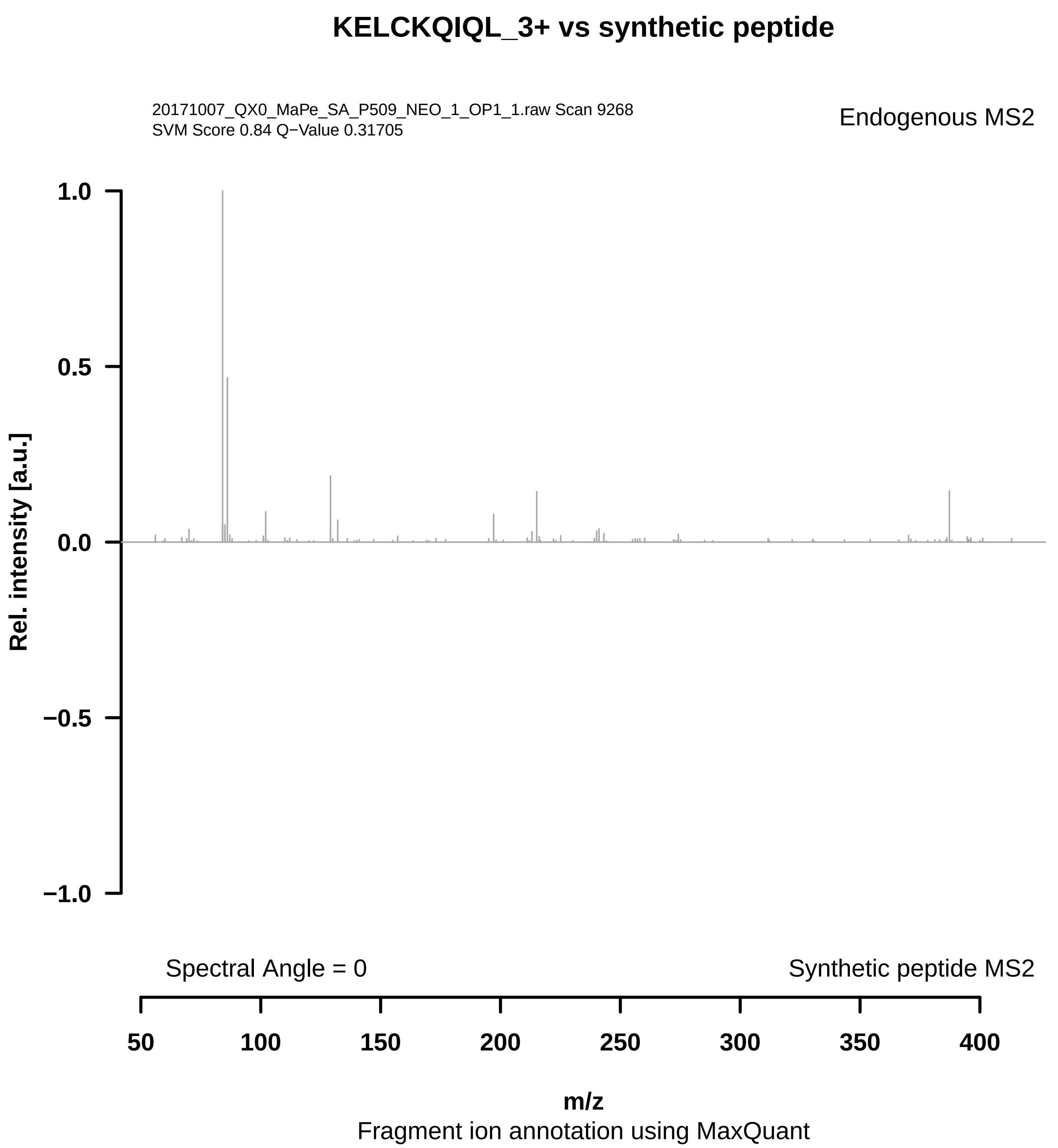
KELCKQIQL_3+ vs synthetic peptide
20171007_QX0_MaPe_SA_P509_NEO_1_OP1_1.raw Scan 9268
SVM Score 0.84 Q−Value 0.31705
Endogenous MS2
Rel. intensity [a.u.]
1.0
0.5
0.0
−0.5
−1.0
Spectral Angle = 0
Synthetic peptide MS2
50
100
150
200
250
300
350
400
m/z
Fragment ion annotation using MaxQuant

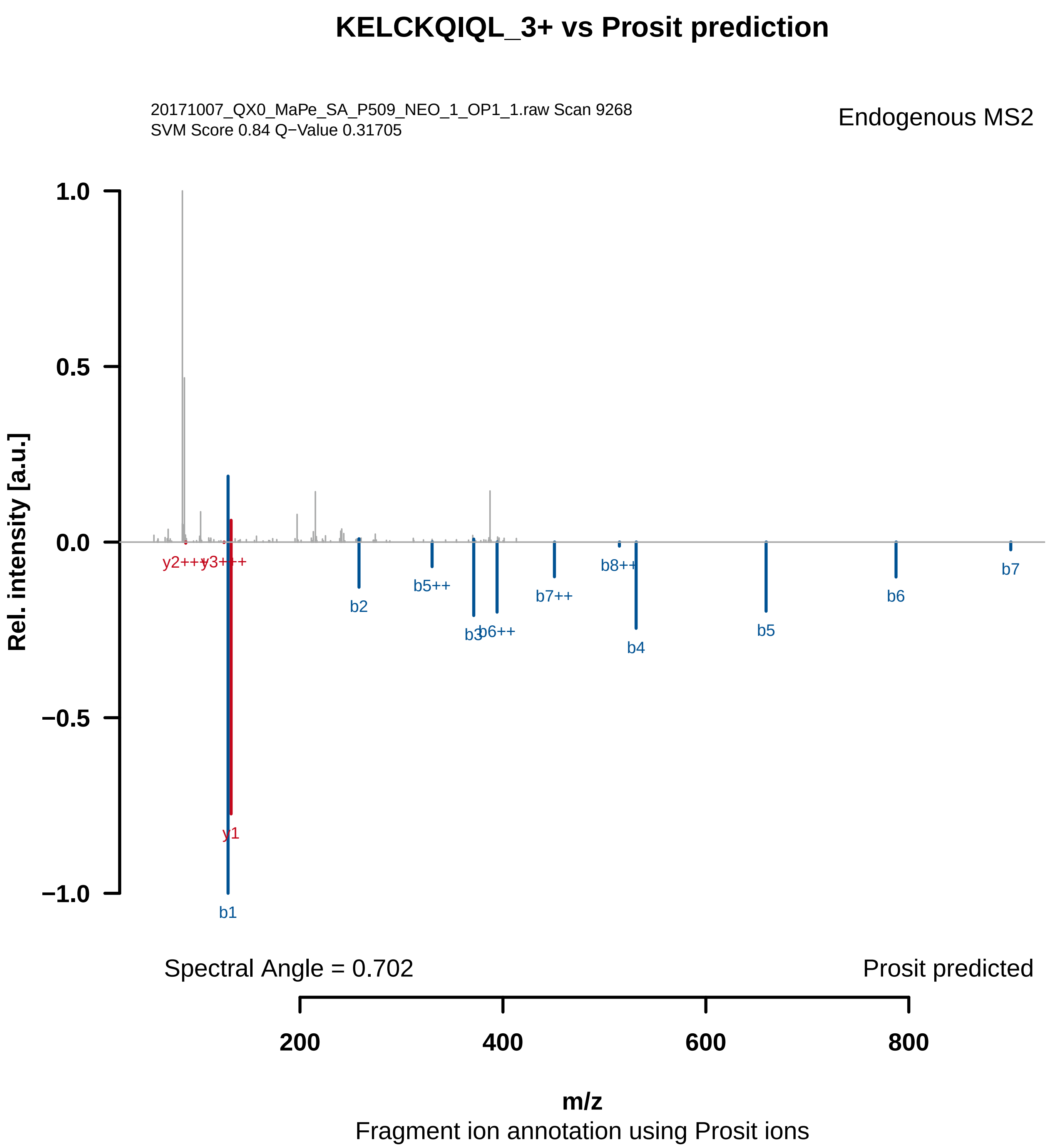
KELCKQIQL_3+ vs Prosit prediction
20171007_QX0_MaPe_SA_P509_NEO_1_OP1_1.raw Scan 9268
SVM Score 0.84 Q−Value 0.31705
Endogenous MS2
Rel. intensity [a.u.]
1.0
0.5
0.0
−0.5
−1.0
y2++
y3+++
y1
b1
b2
b5++
b3
b6++
b7++
b8++
b4
b5
b6
b7
Spectral Angle = 0.702
Prosit predicted
200
400
600
800
m/z
Fragment ion annotation using Prosit ions

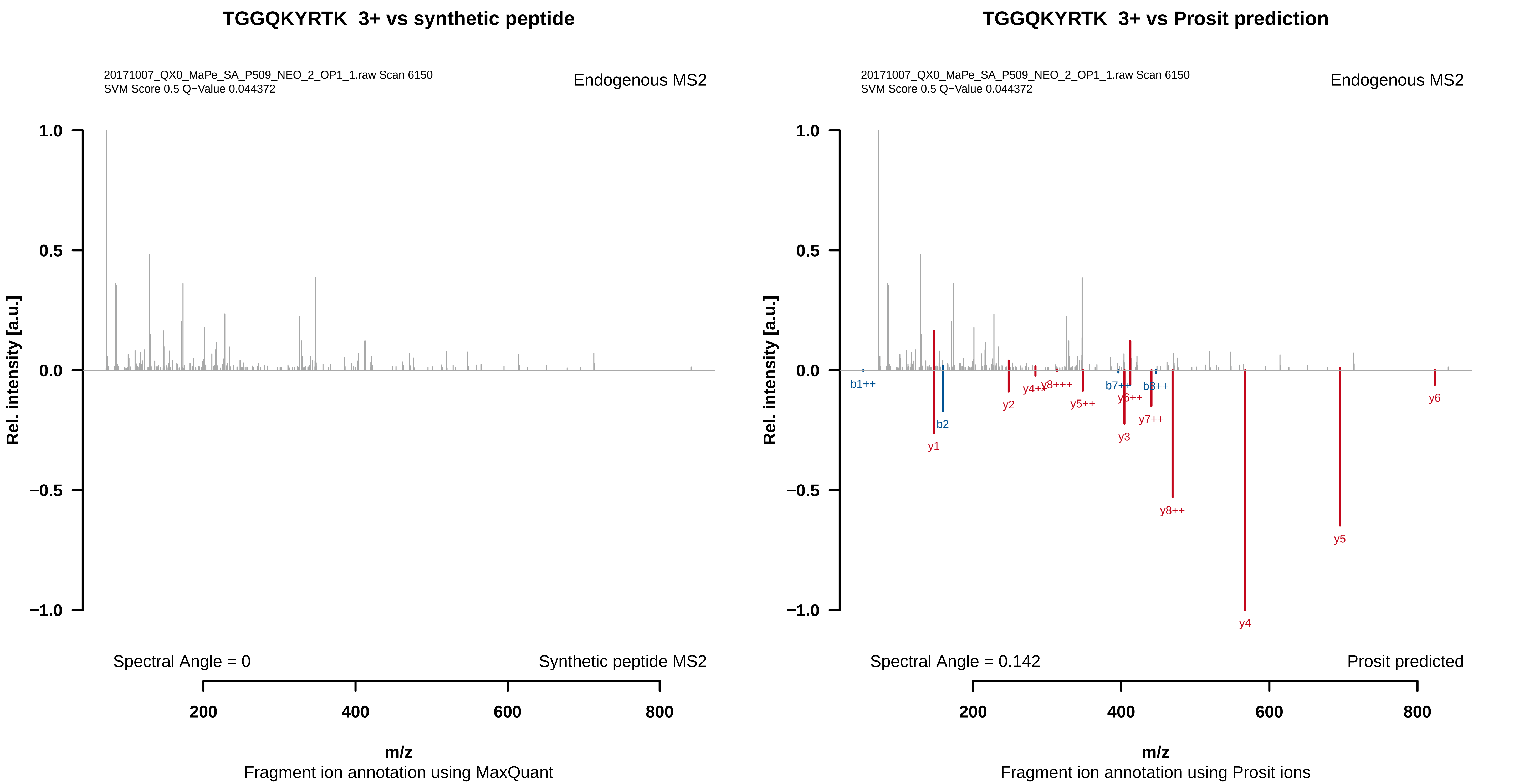
TGGQKYRTK_3+ vs synthetic peptide
20171007_QX0_MaPe_SA_P509_NEO_2_OP1_1.raw Scan 6150
SVM Score 0.5 Q−Value 0.044372
Endogenous MS2
Rel. intensity [a.u.]
1.0
0.5
0.0
−0.5
−1.0
Spectral Angle = 0
Synthetic peptide MS2
200
400
600
800
m/z
Fragment ion annotation using MaxQuant
TGGQKYRTK_3+ vs Prosit prediction
20171007_QX0_MaPe_SA_P509_NEO_2_OP1_1.raw Scan 6150
SVM Score 0.5 Q−Value 0.044372
Endogenous MS2
Rel. intensity [a.u.]
1.0
0.5
0.0
−0.5
−1.0
b1++
y1
b2
y2
y4++
y8+++
y5++
b7++
y6++
b8++
y3
y7++
y8++
y4
y5
y6
Spectral Angle = 0.142
Prosit predicted
200
400
600
800
m/z
Fragment ion annotation using Prosit ions

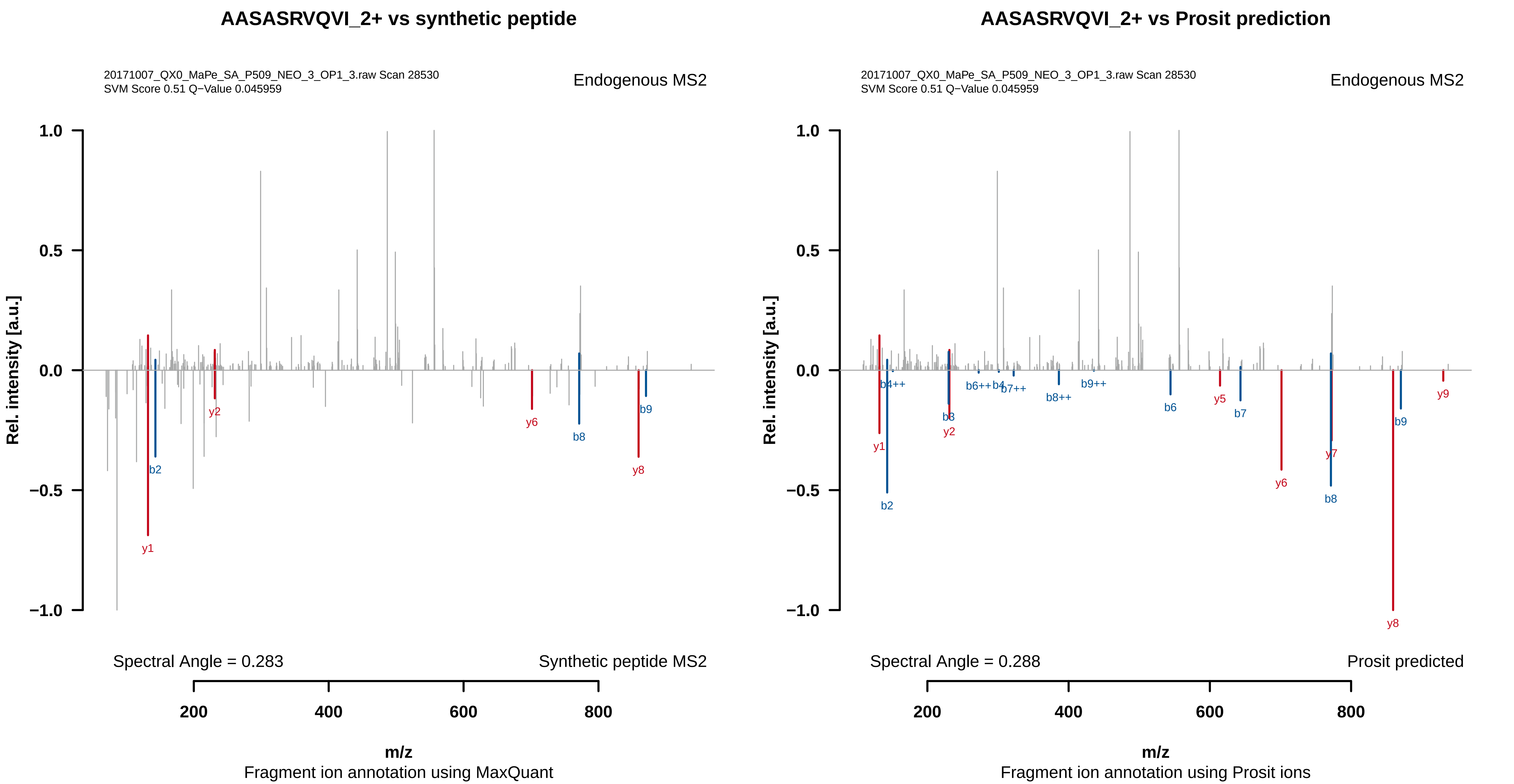

AASASRVQVI_2+ vs synthetic peptide
20171007_QX0_MaPe_SA_P509_NEO_3_OP1_3.raw Scan 28530
SVM Score 0.51 Q−Value 0.045959
Endogenous MS2
Rel. intensity [a.u.]
1.0
0.5
0.0
−0.5
−1.0
y1
b2
y2
y6
b8
y8
b9
Spectral Angle = 0.283
Synthetic peptide MS2
200
400
600
800
m/z
Fragment ion annotation using MaxQuant
AASASRVQVI_2+ vs Prosit prediction
20171007_QX0_MaPe_SA_P509_NEO_3_OP1_3.raw Scan 28530
SVM Score 0.51 Q−Value 0.045959
Endogenous MS2
Rel. intensity [a.u.]
1.0
0.5
0.0
−0.5
−1.0
b4++
b6++
b4
b7++
b8++
b9++
y1
b2
b3
y2
b6
y5
b7
y6
y7
b8
b9
y9
y8
Spectral Angle = 0.288
Prosit predicted
200
400
600
800
m/z
Fragment ion annotation using Prosit ions

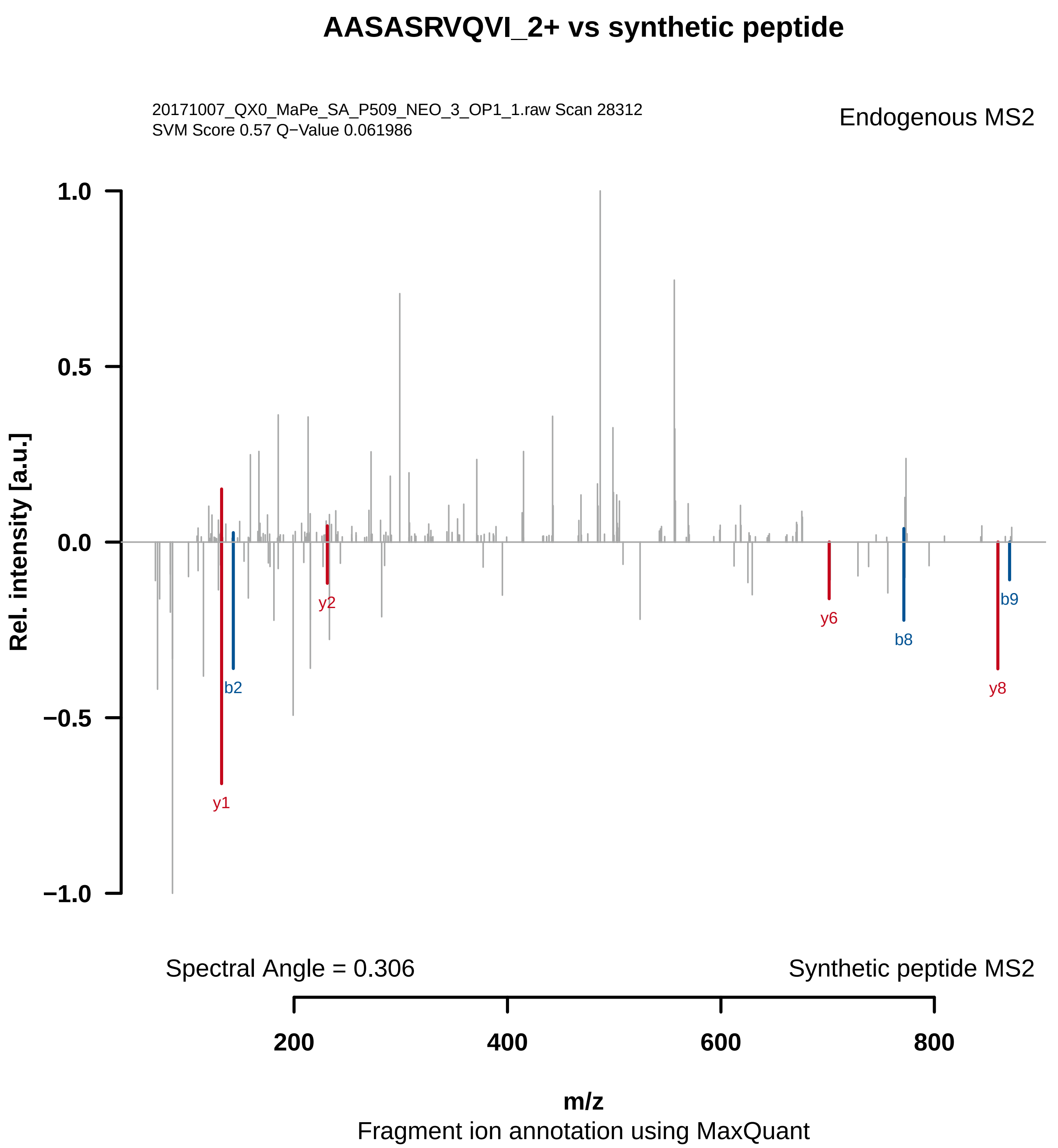
AASASRVQVI_2+ vs synthetic peptide
20171007_QX0_MaPe_SA_P509_NEO_3_OP1_1.raw Scan 28312
SVM Score 0.57 Q−Value 0.061986
Endogenous MS2
Rel. intensity [a.u.]
1.0
0.5
0.0
−0.5
−1.0
y1
b2
y2
y6
b8
y8
b9
Spectral Angle = 0.306
Synthetic peptide MS2
200
400
600
800
m/z
Fragment ion annotation using MaxQuant

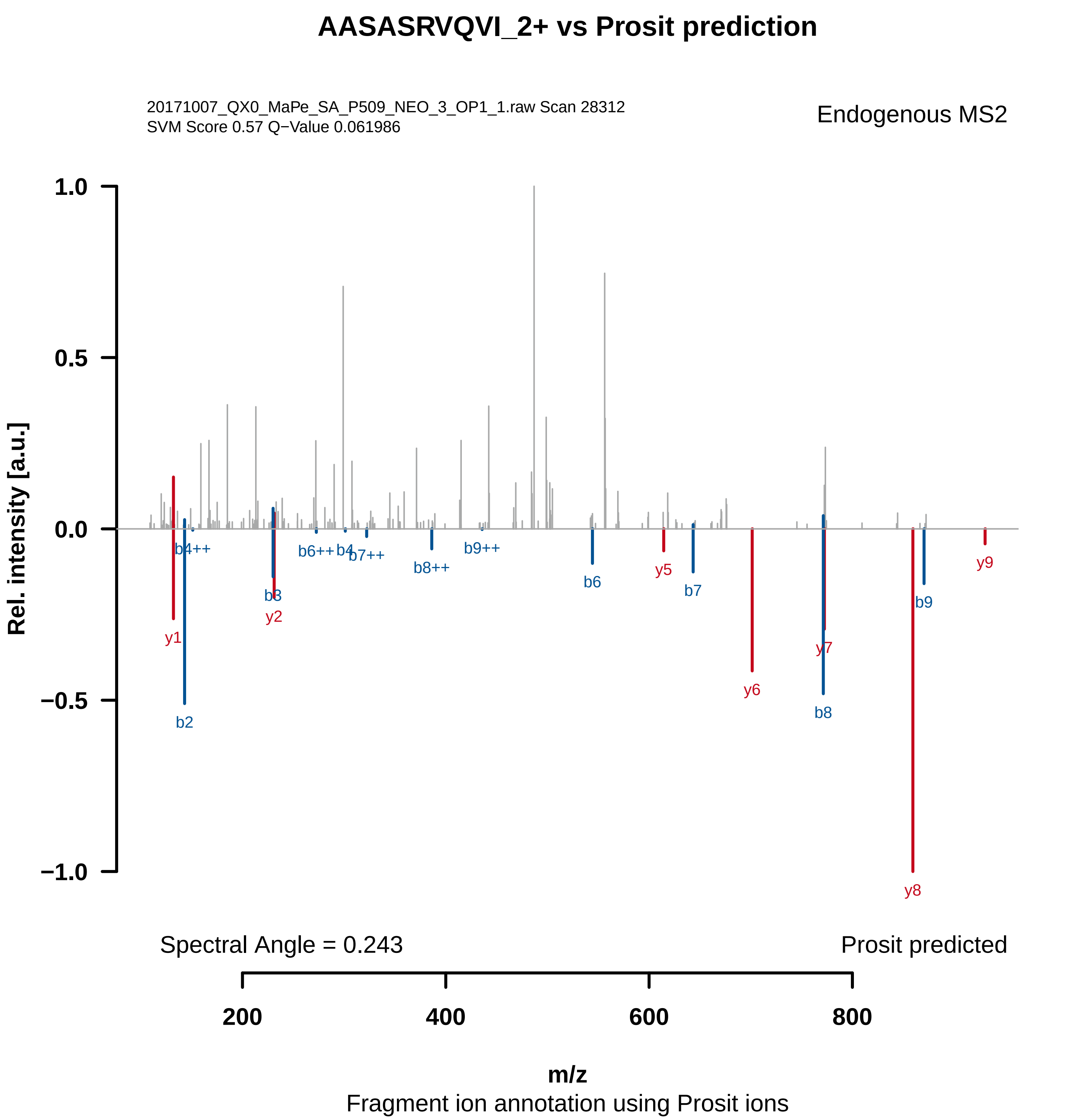
AASASRVQVI_2+ vs Prosit prediction
20171007_QX0_MaPe_SA_P509_NEO_3_OP1_1.raw Scan 28312
SVM Score 0.57 Q−Value 0.061986
Endogenous MS2
Rel. intensity [a.u.]
1.0
0.5
0.0
−0.5
−1.0
b4++
b6++
b4
b7++
b8++
b9++
b3
y2
y1
b2
b6
y5
b7
y7
y6
b8
b9
y9
y8
Spectral Angle = 0.243
Prosit predicted
200
400
600
800
m/z
Fragment ion annotation using Prosit ions

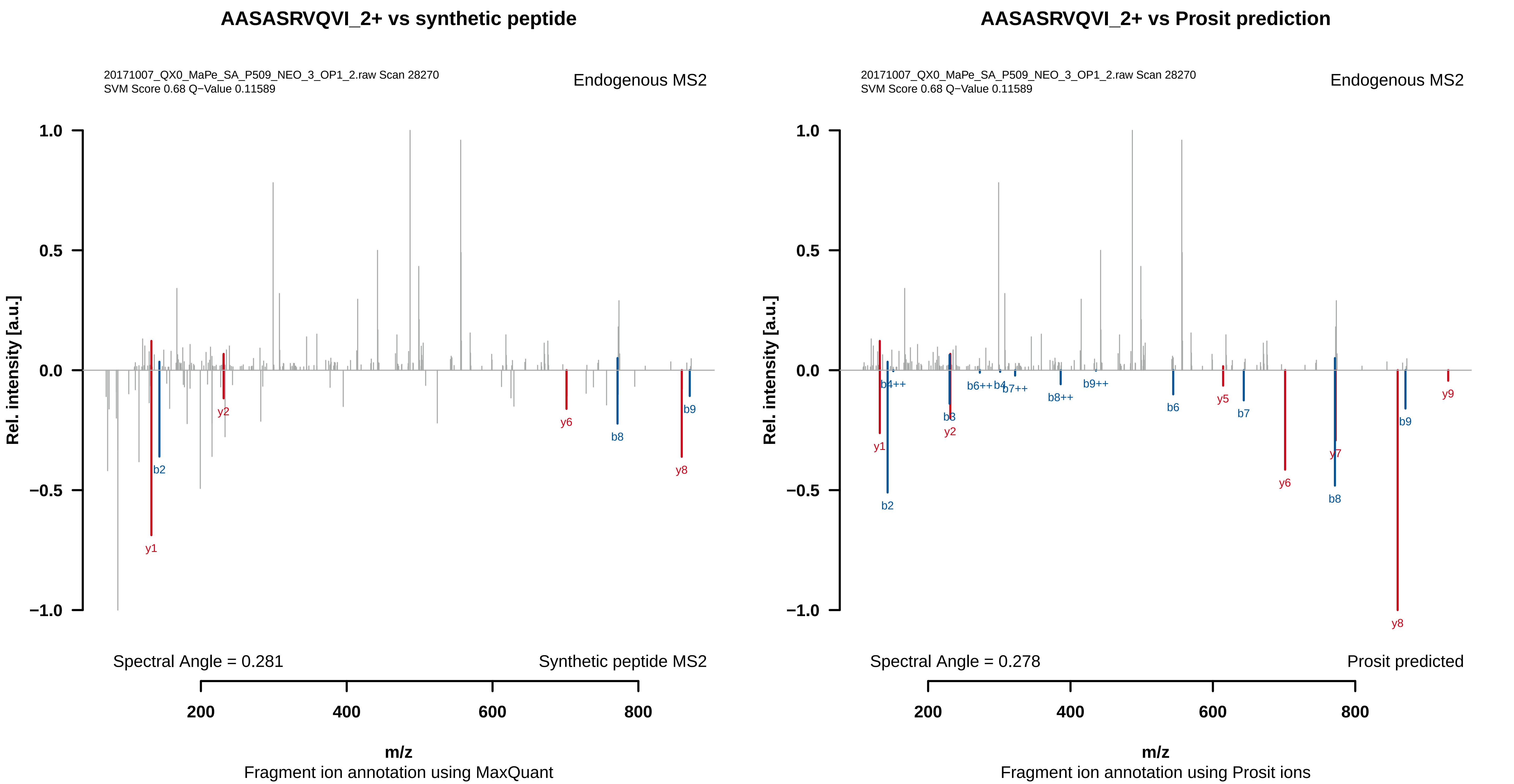
AASASRVQVI_2+ vs synthetic peptide
20171007_QX0_MaPe_SA_P509_NEO_3_OP1_2.raw Scan 28270
SVM Score 0.68 Q−Value 0.11589
Endogenous MS2
Rel. intensity [a.u.]
1.0
0.5
0.0
−0.5
−1.0
y1
b2
y2
y6
b8
y8
b9
Spectral Angle = 0.281
Synthetic peptide MS2
200
400
600
800
m/z
Fragment ion annotation using MaxQuant
AASASRVQVI_2+ vs Prosit prediction
20171007_QX0_MaPe_SA_P509_NEO_3_OP1_2.raw Scan 28270
SVM Score 0.68 Q−Value 0.11589
Endogenous MS2
Rel. intensity [a.u.]
1.0
0.5
0.0
−0.5
−1.0
b4++
b6++
b4
b7++
b8++
b9++
b3
y2
y1
b2
b6
y5
b7
y9
b9
y7
y6
b8
y8
Spectral Angle = 0.278
Prosit predicted
200
400
600
800
m/z
Fragment ion annotation using Prosit ions

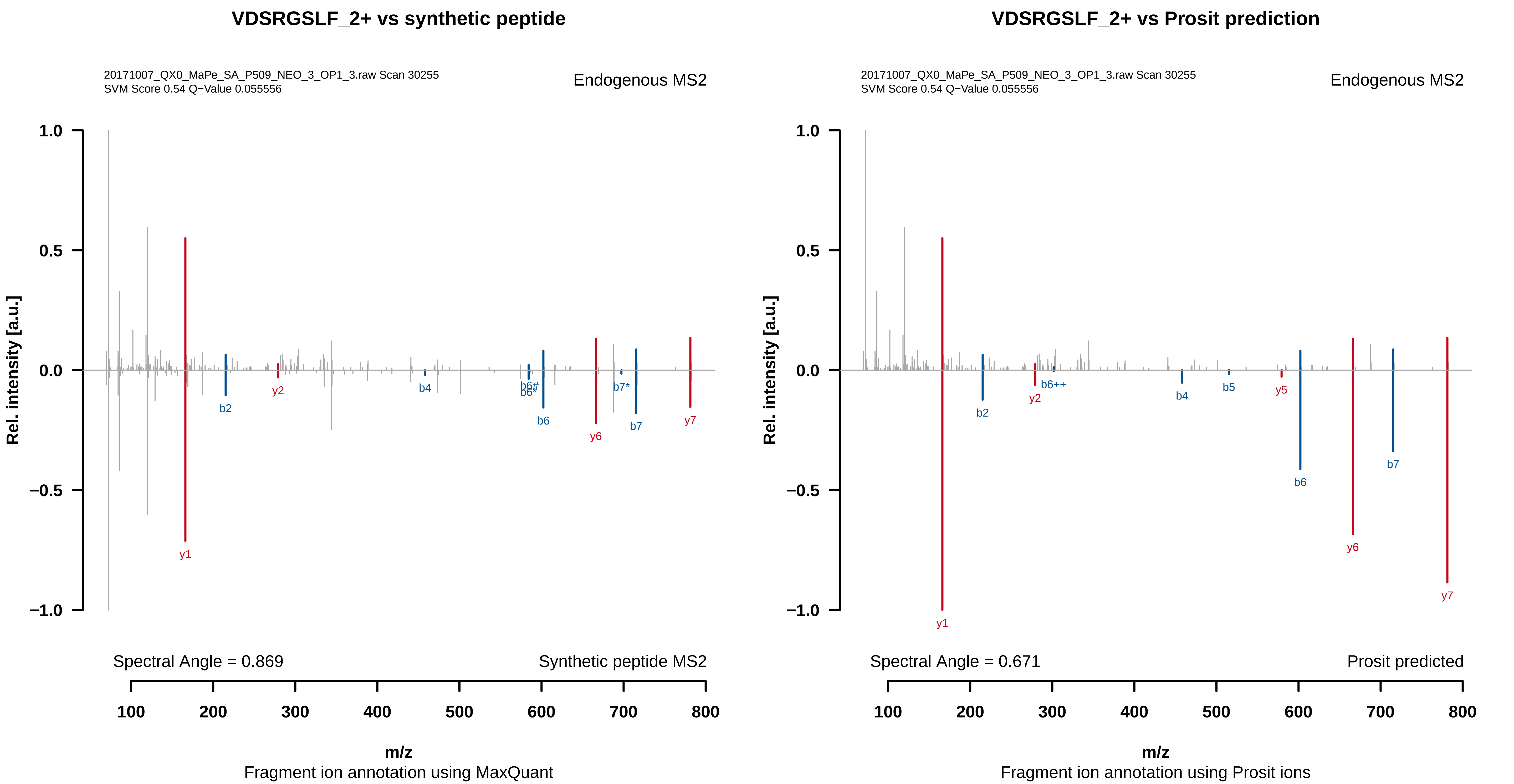

VDRSGSLF_2+ vs synthetic peptide
20171007_QX0_MaPe_SA_P509_NEO_3_OP1_3.raw Scan 30255
SVM Score 0.54 Q−Value 0.055556
Endogenous MS2
Rel. intensity [a.u.]
1.0
0.5
0.0
−0.5
−1.0
y1
b2
y2
b4
b6#
b6*
b6
y6
b7*
b7
y7
Spectral Angle = 0.869
Synthetic peptide MS2
100
200
300
400
500
600
700
800
m/z
Fragment ion annotation using MaxQuant
VDSRGSLF_2+ vs Prosit prediction
20171007_QX0_MaPe_SA_P509_NEO_3_OP1_3.raw Scan 30255
SVM Score 0.54 Q−Value 0.055556
Endogenous MS2
Rel. intensity [a.u.]
1.0
0.5
0.0
−0.5
−1.0
y1
b2
y2
b6++
b4
b5
y5
b6
y6
b7
y7
Spectral Angle = 0.671
Prosit predicted
100
200
300
400
500
600
700
800
m/z
Fragment ion annotation using Prosit ions

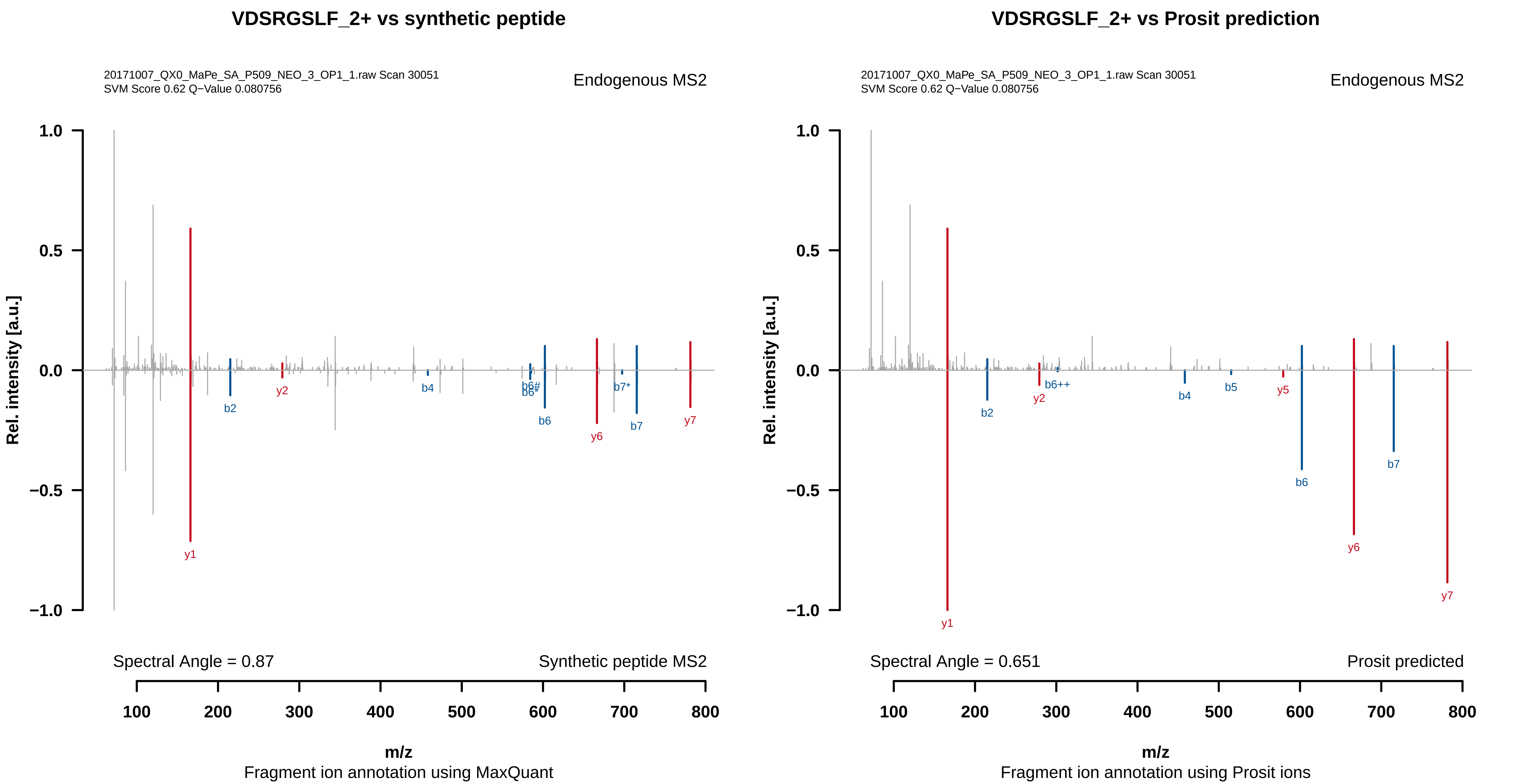
VDSRGSLF_2+ vs synthetic peptide
20171007_QX0_MaPe_SA_P509_NEO_3_OP1_1.raw Scan 30051
SVM Score 0.62 Q−Value 0.080756
Endogenous MS2
Rel. intensity [a.u.]
1.0
0.5
0.0
−0.5
−1.0
y1
b2
y2
b4
b6#
b6*
b6
y6
b7*
b7
y7
Spectral Angle = 0.87
Synthetic peptide MS2
100
200
300
400
500
600
700
800
m/z
Fragment ion annotation using MaxQuant
VDSRGSLF_2+ vs Prosit prediction
20171007_QX0_MaPe_SA_P509_NEO_3_OP1_1.raw Scan 30051
SVM Score 0.62 Q−Value 0.080756
Endogenous MS2
Rel. intensity [a.u.]
1.0
0.5
0.0
−0.5
−1.0
y1
b2
y2
b6++
b4
b5
y5
b6
y6
b7
y7
Spectral Angle = 0.651
Prosit predicted
100
200
300
400
500
600
700
800
m/z
Fragment ion annotation using Prosit ions

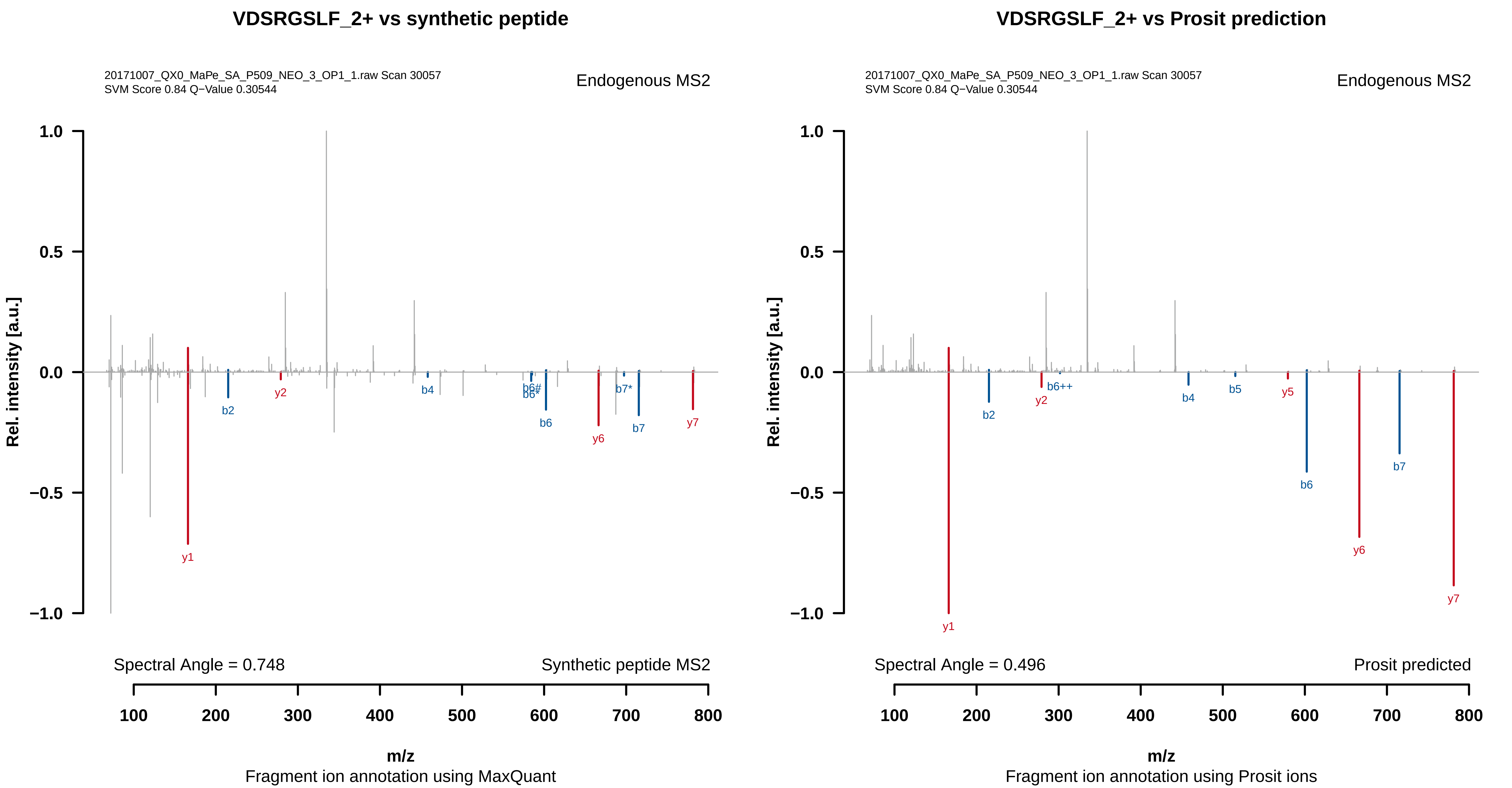

VDSRGSLF_2+ vs synthetic peptide
20171007_QX0_MaPe_SA_P509_NEO_3_OP1_1.raw Scan 30057
SVM Score 0.84 Q−Value 0.30544
Endogenous MS2
Rel. intensity [a.u.]
1.0
0.5
0.0
−0.5
−1.0
y1
b2
y2
b4
b6#
b6*
b6
y6
b7*
b7
y7
Spectral Angle = 0.748
Synthetic peptide MS2
100
200
300
400
500
600
700
800
m/z
Fragment ion annotation using MaxQuant
VDSRGSLF_2+ vs Prosit prediction
20171007_QX0_MaPe_SA_P509_NEO_3_OP1_1.raw Scan 30057
SVM Score 0.84 Q−Value 0.30544
Endogenous MS2
Rel. intensity [a.u.]
1.0
0.5
0.0
−0.5
−1.0
y1
b2
y2
b6++
b4
b5
y5
b6
y6
b7
y7
Spectral Angle = 0.496
Prosit predicted
100
200
300
400
500
600
700
800
m/z
Fragment ion annotation using Prosit ions

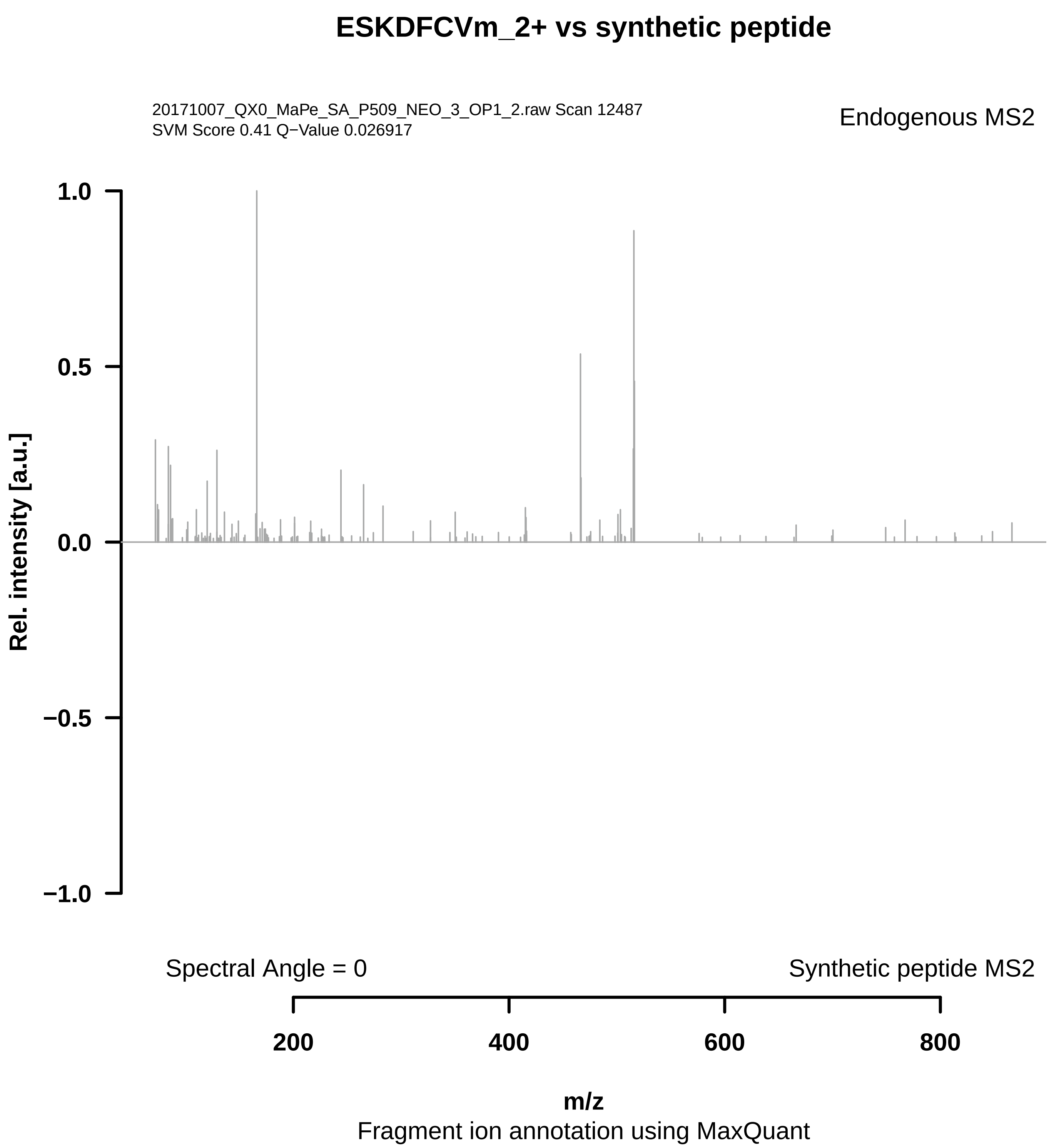
ESKDFCVm_2+ vs synthetic peptide
20171007_QX0_MaPe_SA_P509_NEO_3_OP1_2.raw Scan 12487
SVM Score 0.41 Q−Value 0.026917
Endogenous MS2
1.0
0.5
0.0
−0.5
−1.0
Rel. intensity [a.u.]
Spectral Angle = 0
Synthetic peptide MS2
200
400
600
800
m/z
Fragment ion annotation using MaxQuant

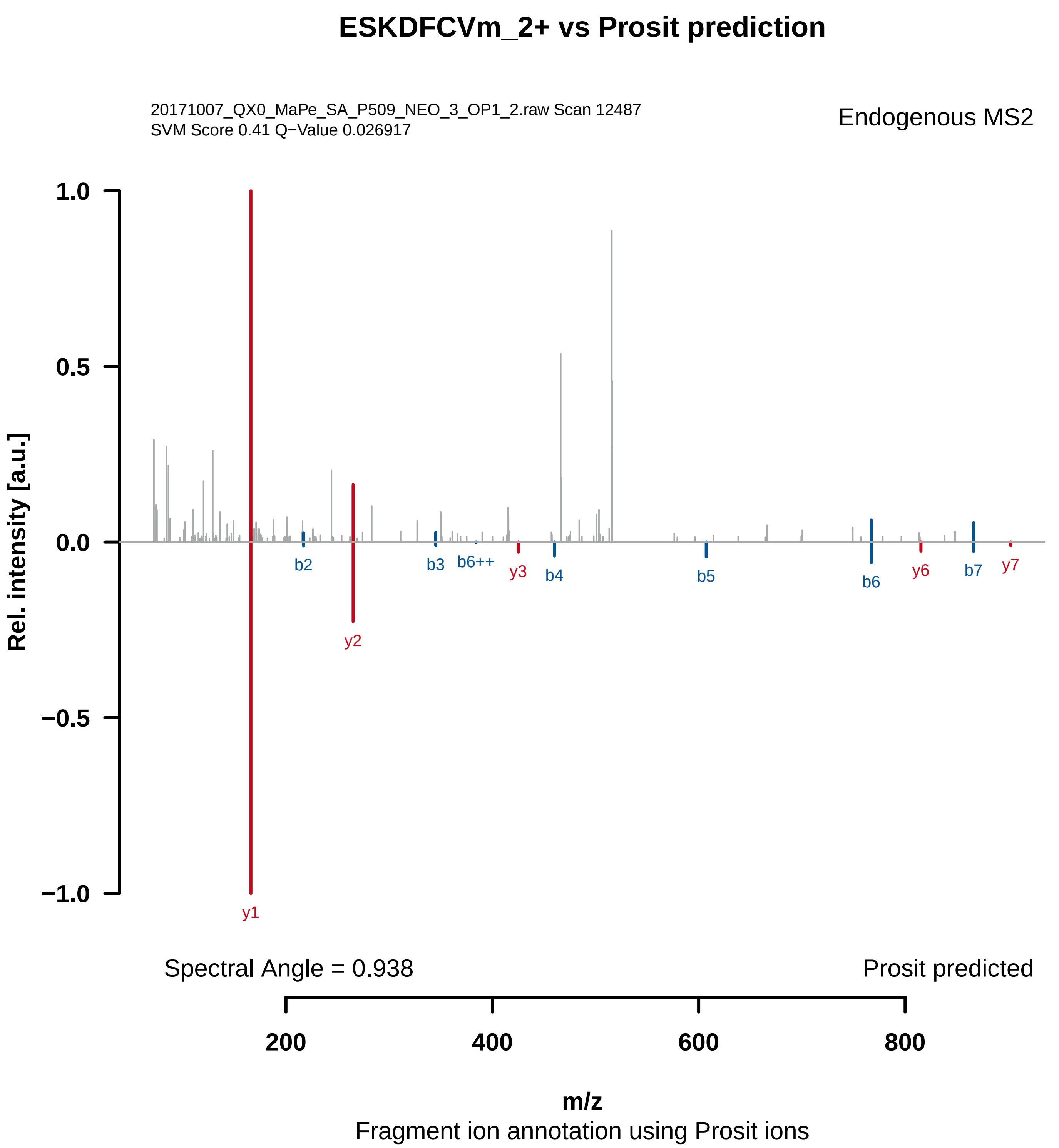
ESKDFCVm_2+ vs Prosit prediction
20171007_QX0_MaPe_SA_P509_NEO_3_OP1_2.raw Scan 12487
SVM Score 0.41 Q−Value 0.026917
Endogenous MS2
1.0
0.5
0.0
−0.5
−1.0
Rel. intensity [a.u.]
b2
b3
b6++
y3
b4
b5
b6
y6
b7
y7
y2
y1
Spectral Angle = 0.938
Prosit predicted
200
400
600
800
m/z
Fragment ion annotation using Prosit ions

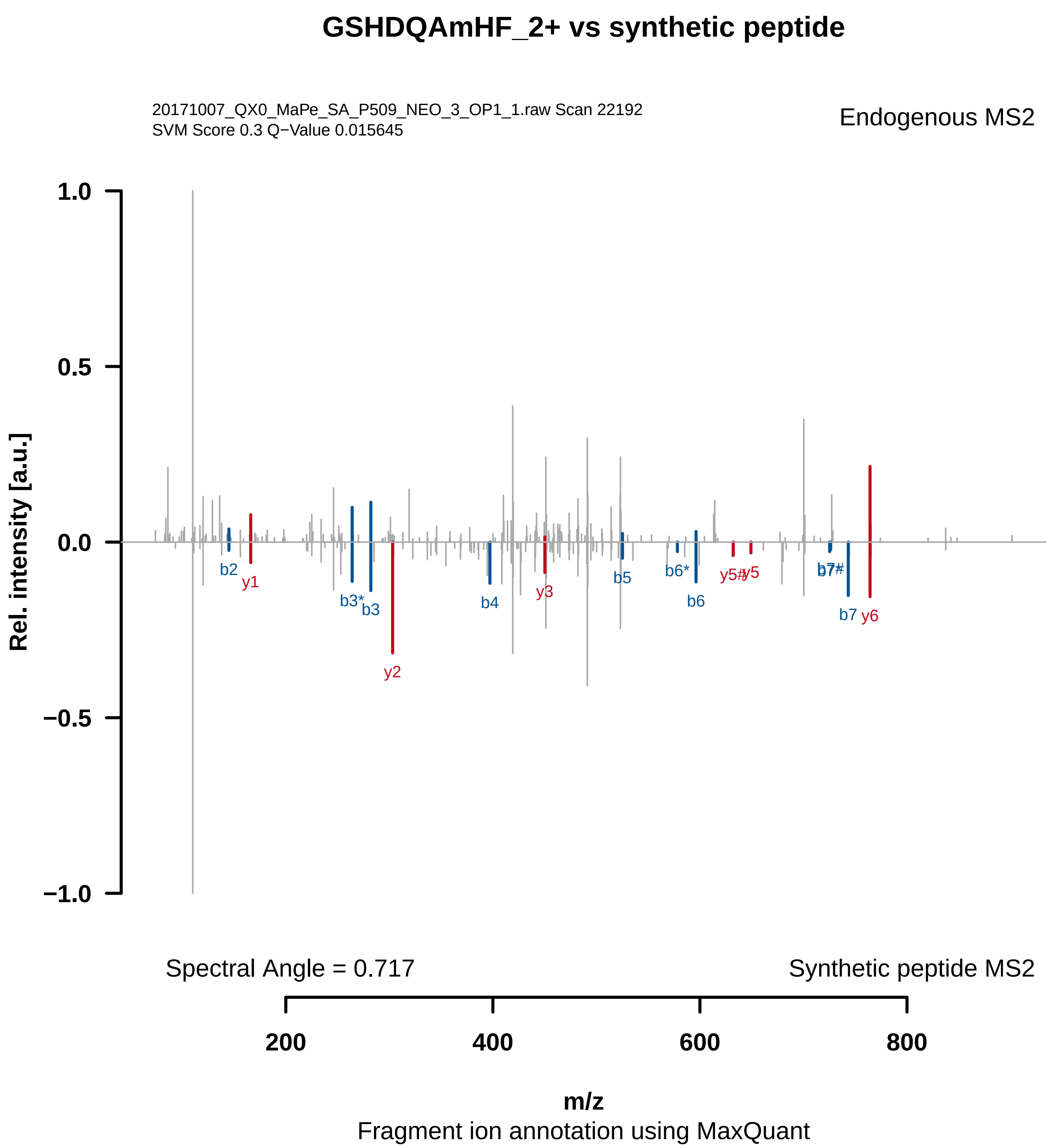

GSHDQAmHF_2+ vs synthetic peptide
20171007_QX0_MaPe_SA_P509_NEO_3_OP1_1.raw Scan 22192
SVM Score 0.3 Q−Value 0.015645
Endogenous MS2
Rel. intensity [a.u.]
1.0
0.5
0.0
−0.5
−1.0
b2
y1
b3*
b3
y2
b4
y3
b5
b6*
b6
y5#
y5
b7#
b7
y6
Spectral Angle = 0.717
Synthetic peptide MS2
200
400
600
800
m/z
Fragment ion annotation using MaxQuant

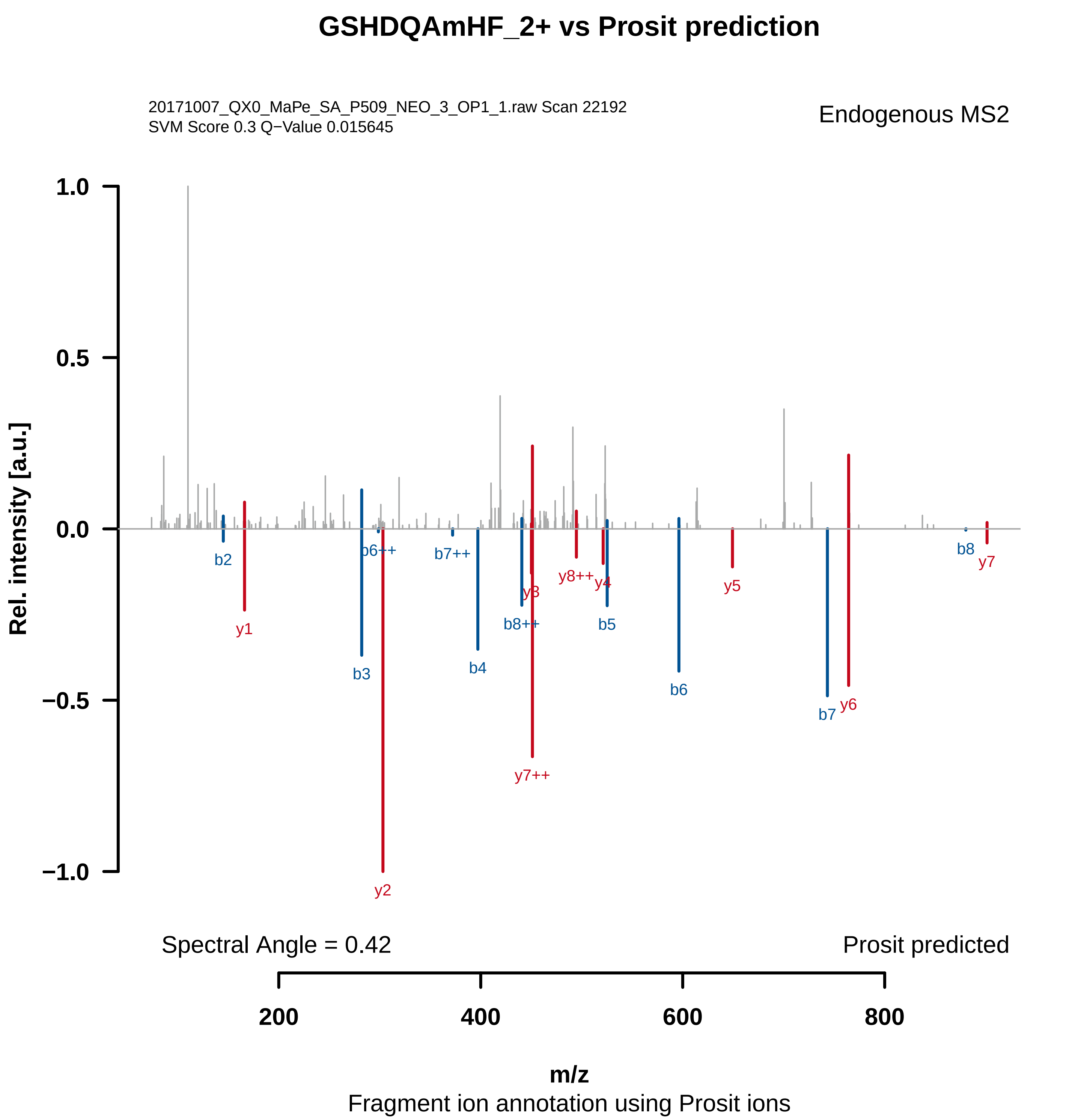

GSHDQAmHF_2+ vs Prosit prediction
20171007_QX0_MaPe_SA_P509_NEO_3_OP1_1.raw Scan 22192
SVM Score 0.3 Q−Value 0.015645
Endogenous MS2
Rel. intensity [a.u.]
1.0
0.5
0.0
−0.5
−1.0
b2
y1
b6++
b3
y2
b7++
b4
b8++
y3
y7++
y8++
y4
b5
b6
y5
b7
y6
b8
y7
Spectral Angle = 0.42
Prosit predicted
200
400
600
800
m/z
Fragment ion annotation using Prosit ions

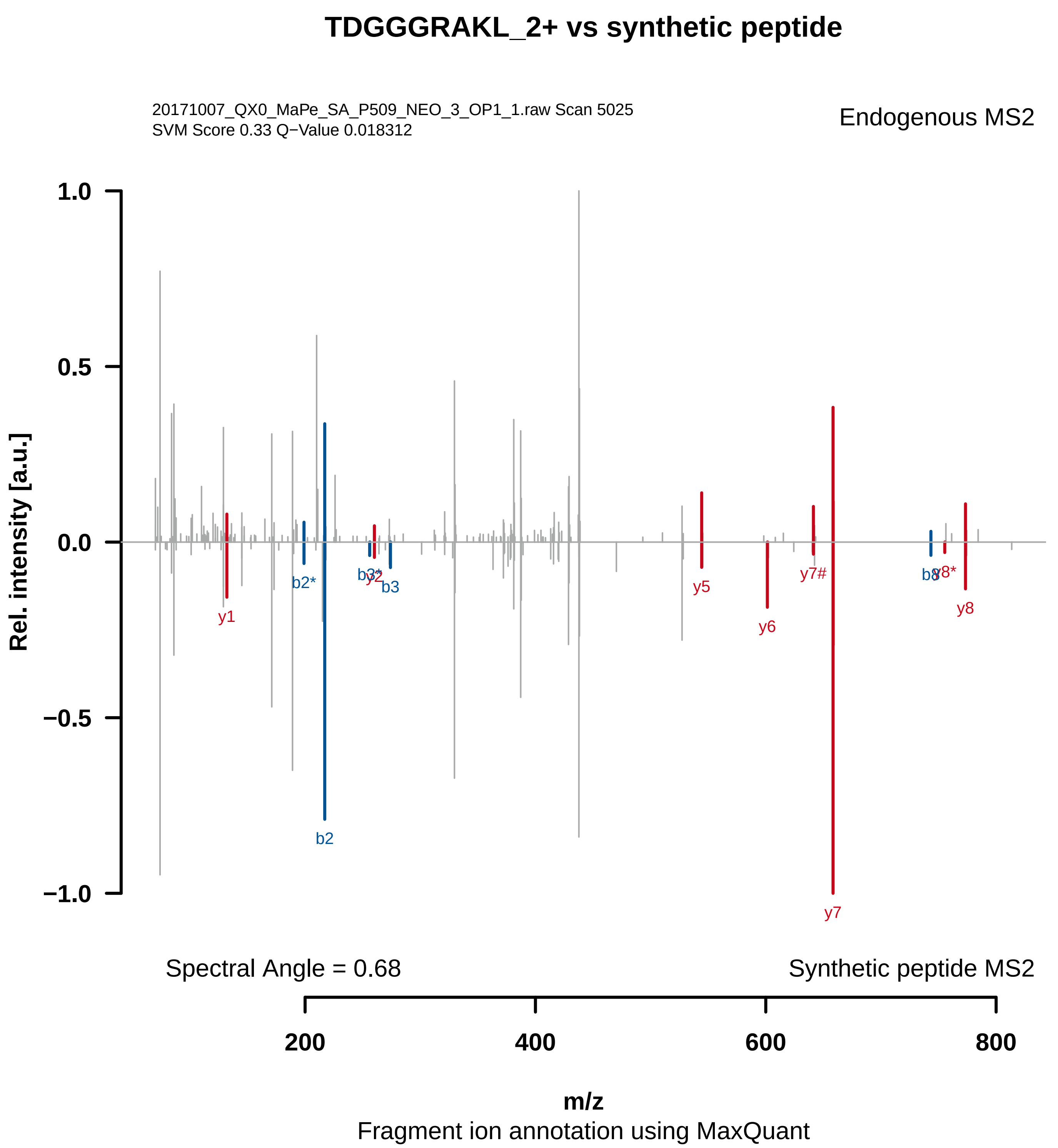
TDGGGRAKL_2+ vs synthetic peptide
20171007_QX0_MaPe_SA_P509_NEO_3_OP1_1.raw Scan 5025
SVM Score 0.33 Q−Value 0.018312
Endogenous MS2
Rel. intensity [a.u.]
1.0
0.5
0.0
−0.5
−1.0
y1
b2*
b2
b3
y5
y6
y7#
y7
y8
Spectral Angle = 0.68
Synthetic peptide MS2
200
400
600
800
m/z
Fragment ion annotation using MaxQuant

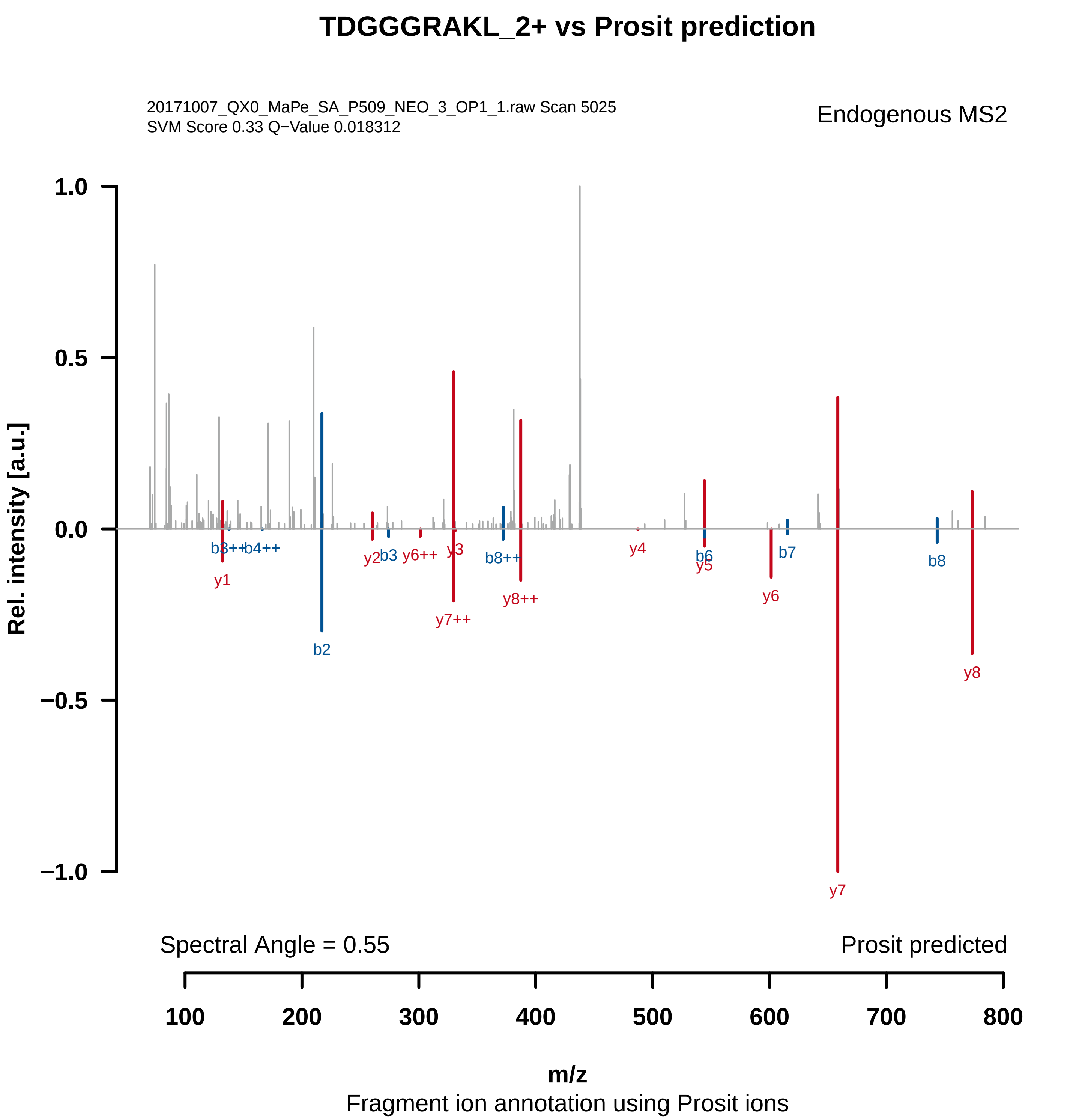
TDGGGRAKL_2+ vs Prosit prediction
20171007_QX0_MaPe_SA_P509_NEO_3_OP1_1.raw Scan 5025
SVM Score 0.33 Q−Value 0.018312
Endogenous MS2
Rel. intensity [a.u.]
1.0
0.5
0.0
−0.5
−1.0
y1
b4++
b2
b3
y6++
y3
y7++
b8++
y8++
y4
y6
b7
y7
b8
y8
Spectral Angle = 0.55
Prosit predicted
100
200
300
400
500
600
700
800
m/z
Fragment ion annotation using Prosit ions

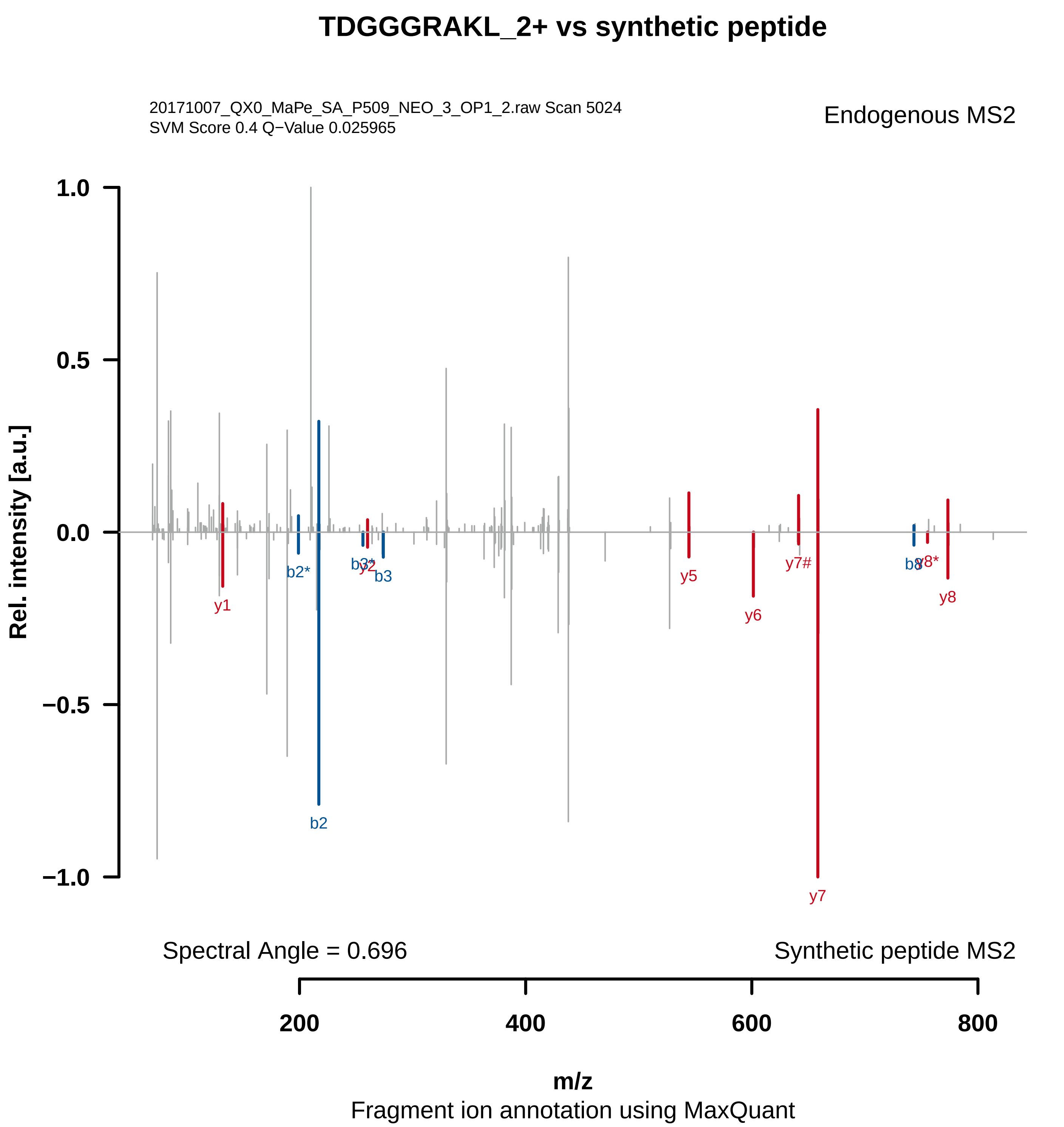
TDGGGRAKL_2+ vs synthetic peptide
20171007_QX0_MaPe_SA_P509_NEO_3_OP1_2.raw Scan 5024
SVM Score 0.4 Q−Value 0.025965
Endogenous MS2
Rel. intensity [a.u.]
1.0
0.5
0.0
−0.5
−1.0
y1
b2*
b2
b3
y2
y5
y6
y7#
y7
b8
y8*
y8
Spectral Angle = 0.696
Synthetic peptide MS2
200
400
600
800
m/z
Fragment ion annotation using MaxQuant

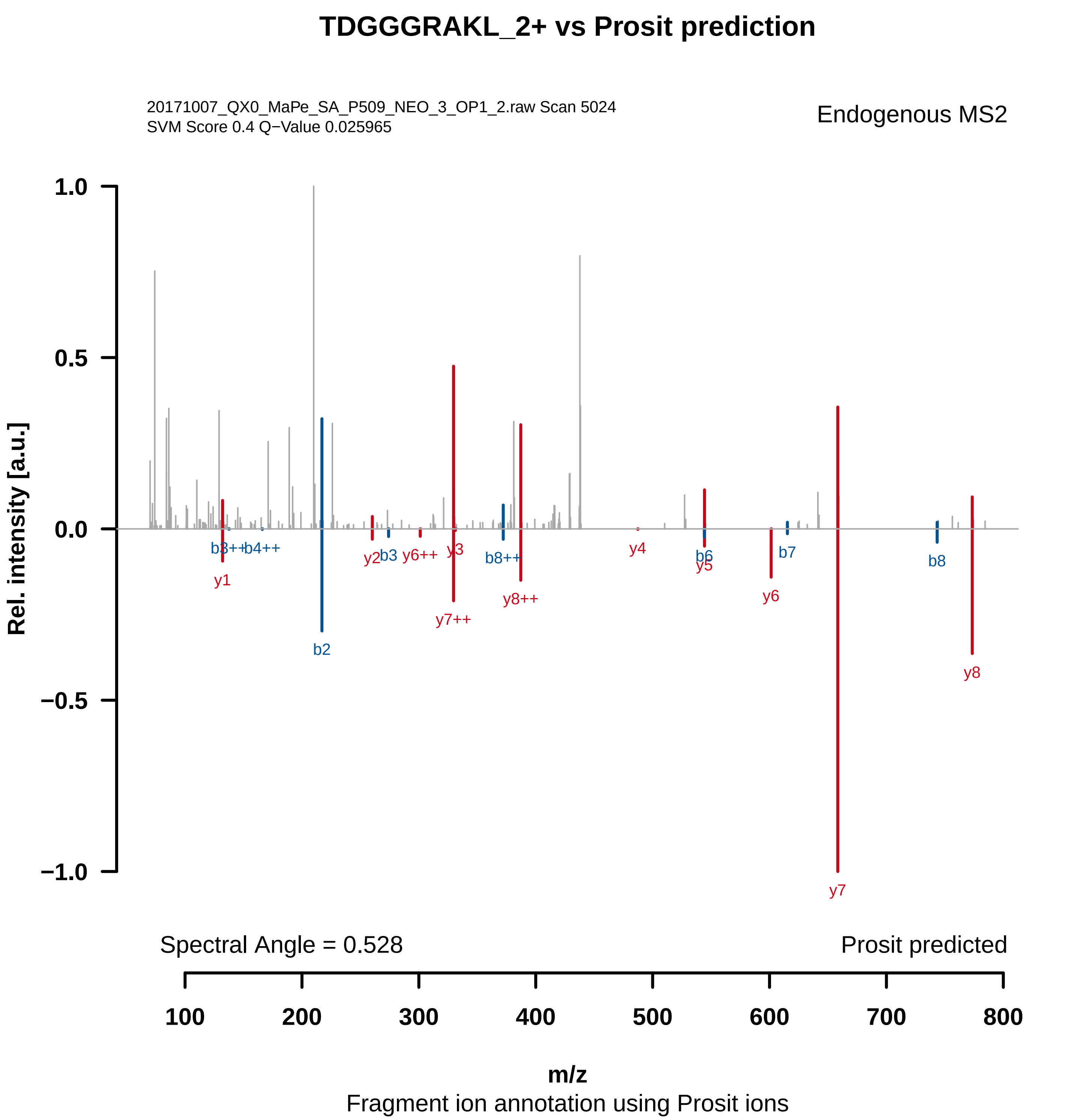
TDGGGRAKL_2+ vs Prosit prediction
20171007_QX0_MaPe_SA_P509_NEO_3_OP1_2.raw Scan 5024
SVM Score 0.4 Q−Value 0.025965
Endogenous MS2
Rel. intensity [a.u.]
1.0
0.5
0.0
−0.5
−1.0
b4++
y1
b2
y2
b3
y6++
y3
y7++
b8++
y8++
y4
b6
y5
y6
b7
y7
b8
y8
Spectral Angle = 0.528
Prosit predicted
100
200
300
400
500
600
700
800
m/z
Fragment ion annotation using Prosit ions

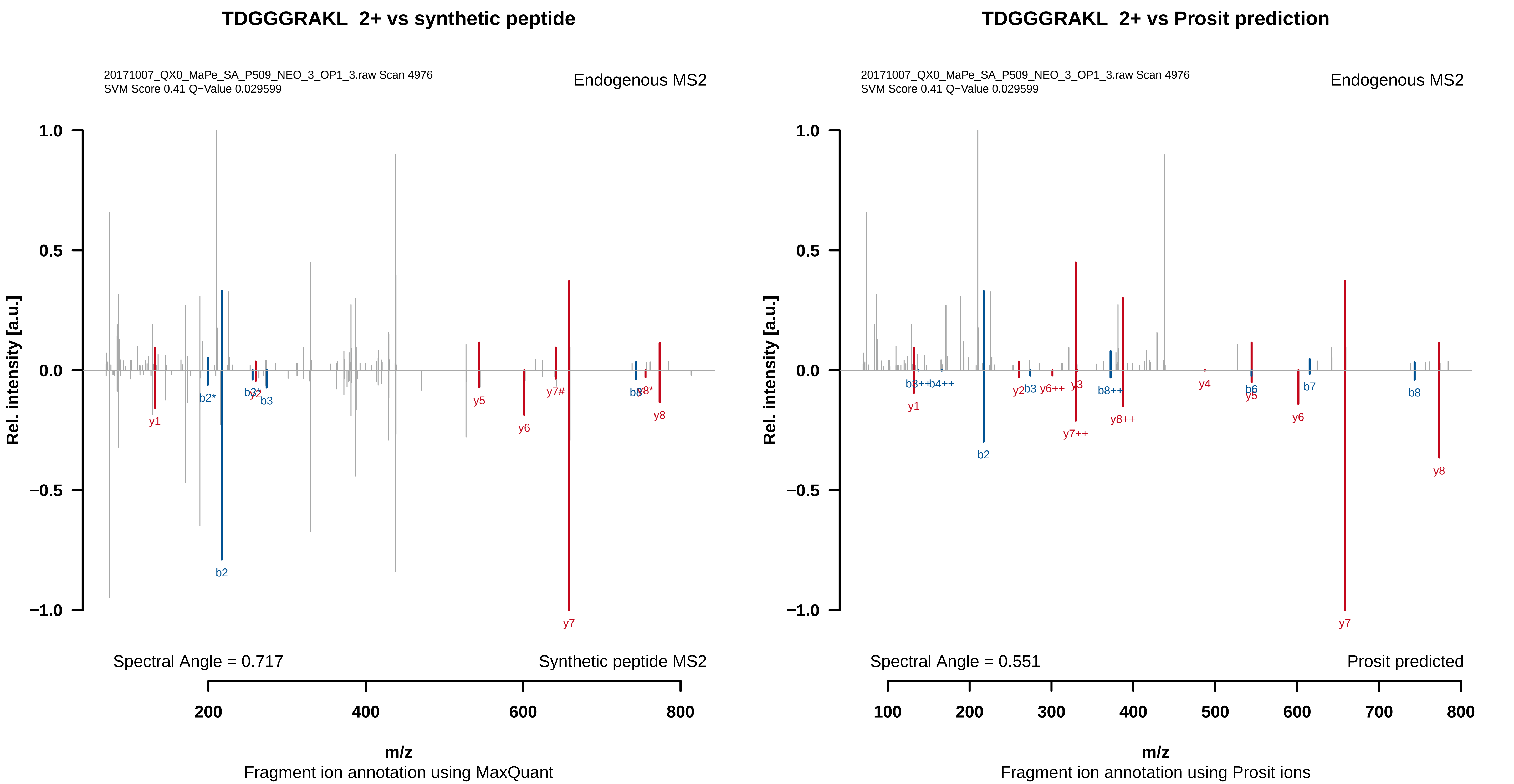

TDGGGRAKL_2+ vs synthetic peptide
20171007_QX0_MaPe_SA_P509_NEO_3_OP1_3.raw Scan 4976
SVM Score 0.41 Q−Value 0.029599
Endogenous MS2
Rel. intensity [a.u.]
1.0
0.5
0.0
−0.5
−1.0
y1
b2*
b2
b3
y2
b3
y5
y6
y7#
y7
b8
y8*
y8
Spectral Angle = 0.717
Synthetic peptide MS2
200
400
600
800
m/z
Fragment ion annotation using MaxQuant
TDGGGRAKL_2+ vs Prosit prediction
20171007_QX0_MaPe_SA_P509_NEO_3_OP1_3.raw Scan 4976
SVM Score 0.41 Q−Value 0.029599
Endogenous MS2
Rel. intensity [a.u.]
1.0
0.5
0.0
−0.5
−1.0
b3++
b4++
y1
b2
y2
b3
y6++
y3
y7++
b8++
y8++
y4
b6
y5
y6
b7
y7
b8
y8
Spectral Angle = 0.551
Prosit predicted
100
200
300
400
500
600
700
800
m/z
Fragment ion annotation using Prosit ions

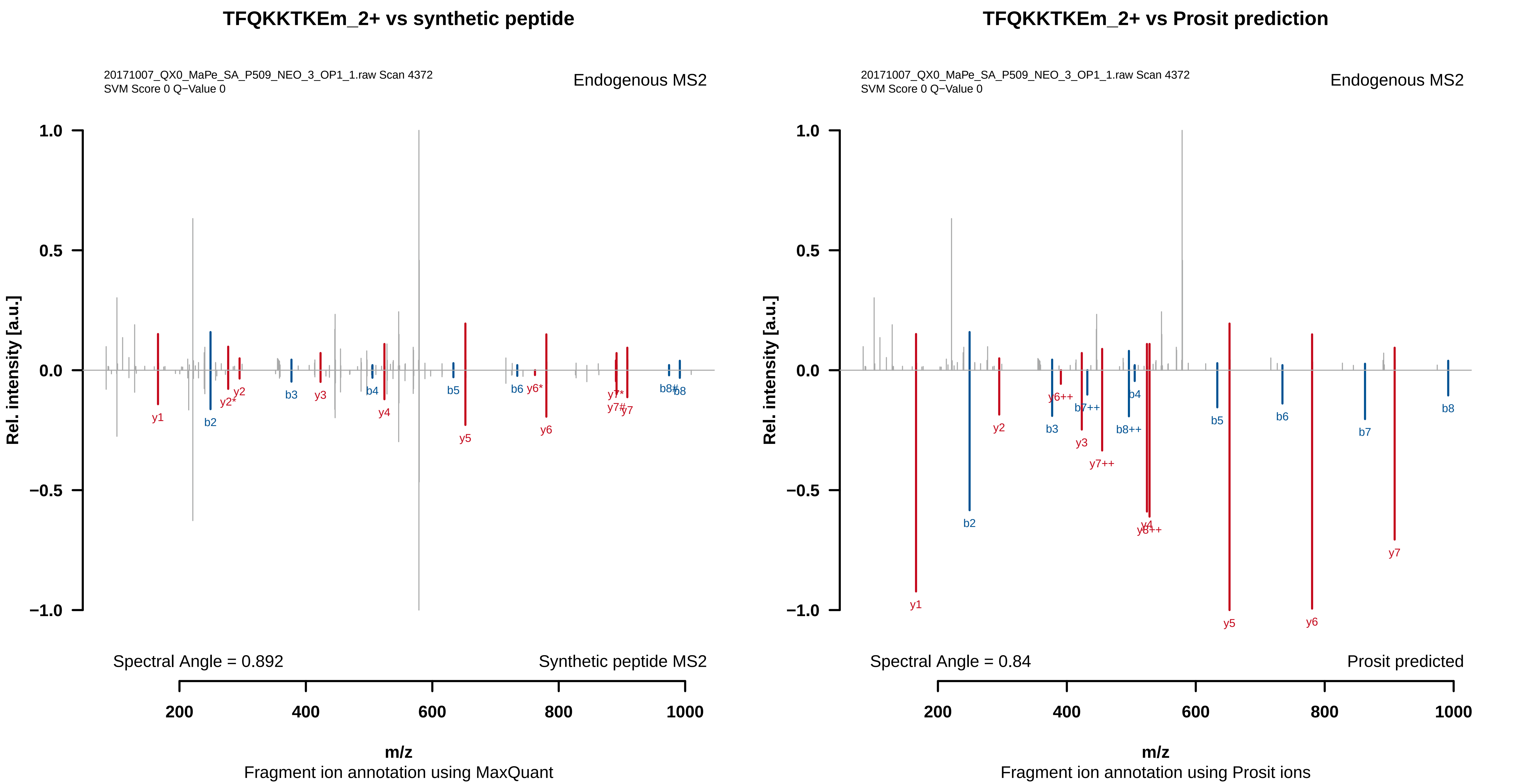

TFQKKTKEm_2+ vs synthetic peptide
20171007_QX0_MaPe_SA_P509_NEO_3_OP1_1.raw Scan 4372
SVM Score 0 Q−Value 0
Endogenous MS2
Rel. intensity [a.u.]
1.0
0.5
0.0
−0.5
−1.0
y1
b2
y2*
y2
b3
y3
b4
y4
b5
y5
b6
y6*
y6
y7*
y7#
y7
b8#
b8
Spectral Angle = 0.892
Synthetic peptide MS2
200
400
600
800
1000
m/z
Fragment ion annotation using MaxQuant
TFQKKTKEm_2+ vs Prosit prediction
20171007_QX0_MaPe_SA_P509_NEO_3_OP1_1.raw Scan 4372
SVM Score 0 Q−Value 0
Endogenous MS2
Rel. intensity [a.u.]
1.0
0.5
0.0
−0.5
−1.0
y1
b2
y2
y6++
b3
b7++
y3
y7++
b8++
b4
y4
y8++
b5
y5
b6
y6
b7
y7
b8
Spectral Angle = 0.84
Prosit predicted
200
400
600
800
1000
m/z
Fragment ion annotation using Prosit ions

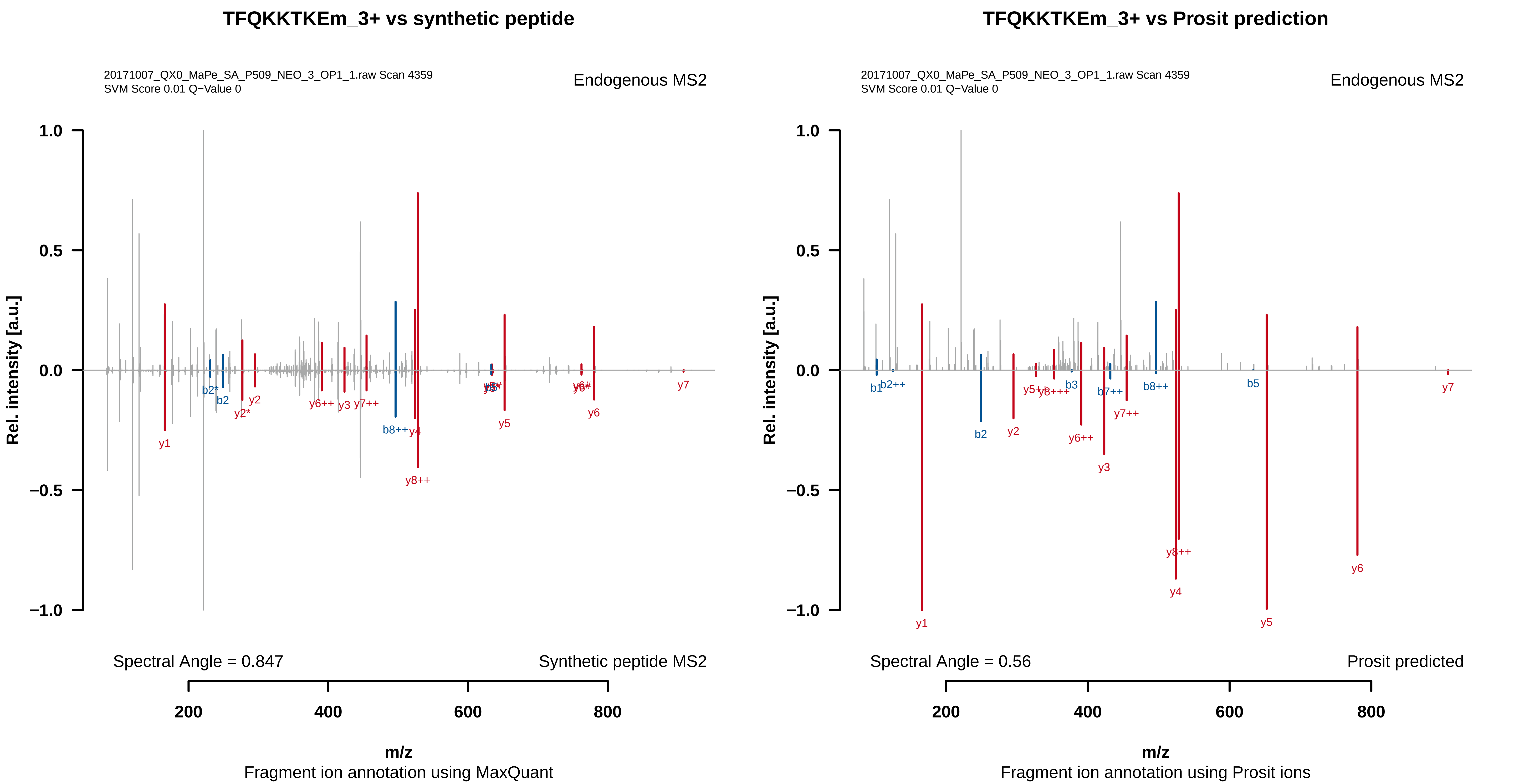
TFQKKTKEm_3+ vs synthetic peptide
20171007_QX0_MaPe_SA_P509_NEO_3_OP1_1.raw Scan 4359
SVM Score 0.01 Q−Value 0
Endogenous MS2
Rel. intensity [a.u.]
1.0
0.5
0.0
−0.5
−1.0
y1
b2*
b2
y2*
y2
y6++
y3
y7++
b8++
y4
y8++
y5
y6
y7
Spectral Angle = 0.847
Synthetic peptide MS2
200
400
600
800
m/z
Fragment ion annotation using MaxQuant
TFQKKTKEm_3+ vs Prosit prediction
20171007_QX0_MaPe_SA_P509_NEO_3_OP1_1.raw Scan 4359
SVM Score 0.01 Q−Value 0
Endogenous MS2
Rel. intensity [a.u.]
1.0
0.5
0.0
−0.5
−1.0
b1
b2++
y1
b2
y2
b3
y6++
y3
y7++
b8++
y8++
y4
b5
y5
y6
y7
Spectral Angle = 0.56
Prosit predicted
200
400
600
800
m/z
Fragment ion annotation using Prosit ions

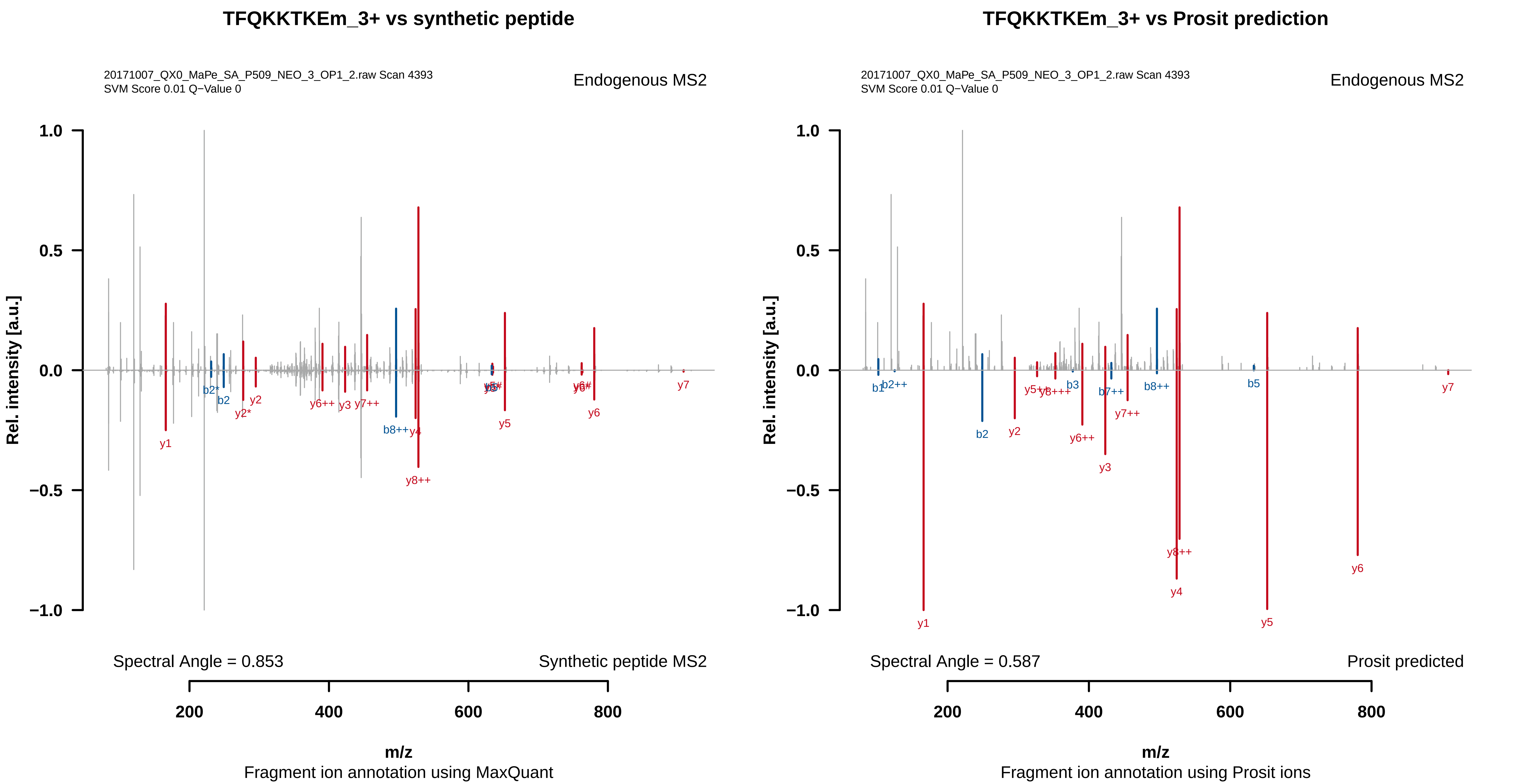

TFQKKTKEm_3+ vs synthetic peptide
20171007_QX0_MaPe_SA_P509_NEO_3_OP1_2.raw Scan 4393
SVM Score 0.01 Q−Value 0
Endogenous MS2
Rel. intensity [a.u.]
1.0
0.5
0.0
−0.5
−1.0
y1
b2*
b2
y2*
y2
y6++
y3
y7++
b8++
y4
y8++
y5
y6
y7
Spectral Angle = 0.853
Synthetic peptide MS2
200
400
600
800
m/z
Fragment ion annotation using MaxQuant
TFQKKTKEm_3+ vs Prosit prediction
20171007_QX0_MaPe_SA_P509_NEO_3_OP1_2.raw Scan 4393
SVM Score 0.01 Q−Value 0
Endogenous MS2
Rel. intensity [a.u.]
1.0
0.5
0.0
−0.5
−1.0
b1
b2++
b2
y2
b3
y6++
y3
b7++
y7++
b8++
y8++
y4
b5
y5
y6
y7
y1
Spectral Angle = 0.587
Prosit predicted
200
400
600
800
m/z
Fragment ion annotation using Prosit ions

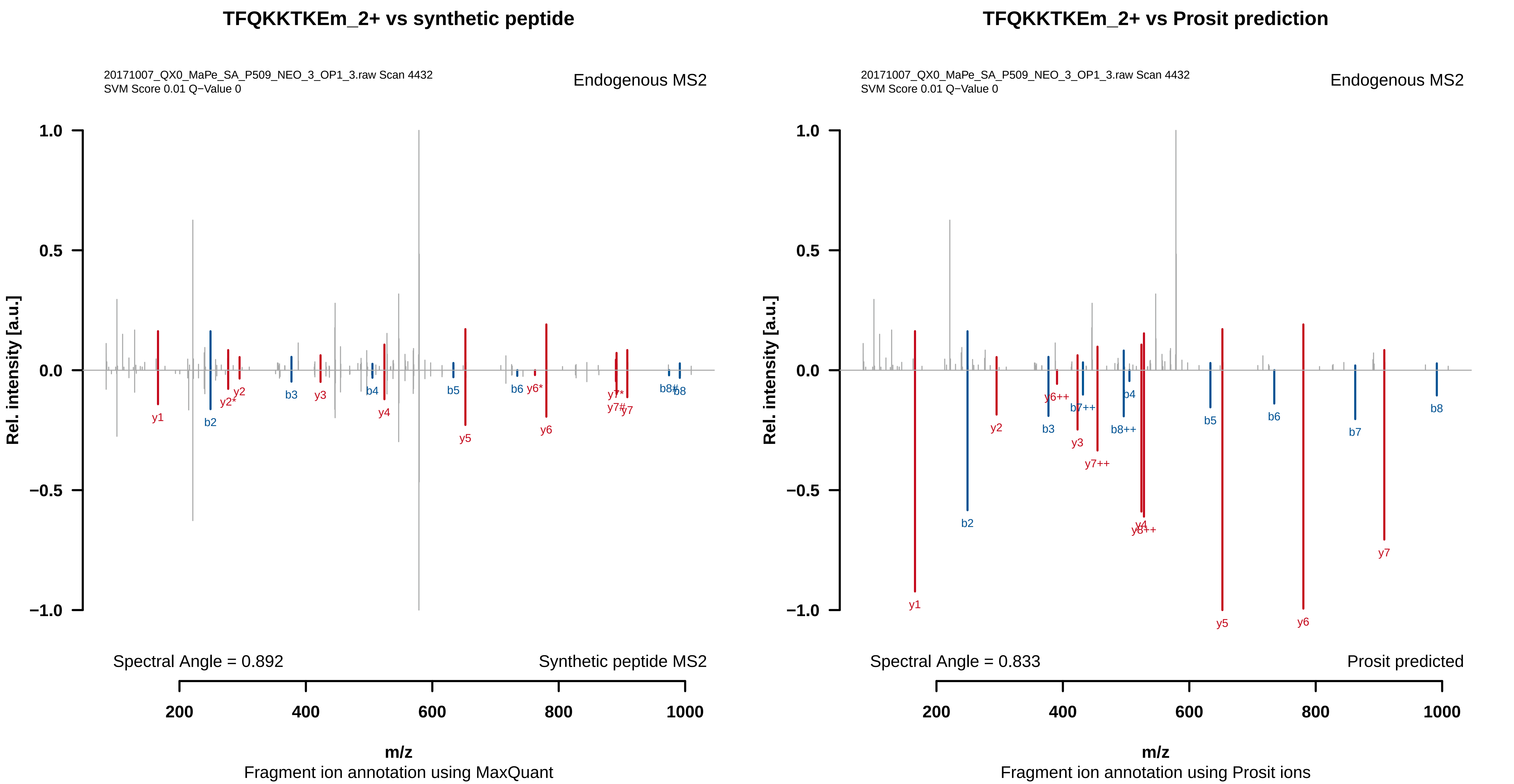
TFQKKTKEm_2+ vs synthetic peptide
20171007_QX0_MaPe_SA_P509_NEO_3_OP1_3.raw Scan 4432
SVM Score 0.01 Q−Value 0
Endogenous MS2
Rel. intensity [a.u.]
1.0
0.5
0.0
−0.5
−1.0
y1
b2
y2*
y2
b3
y3
b4
y4
b5
y5
b6
y6*
y6
y7*
y7#
y7
b8#
b8
Spectral Angle = 0.892
Synthetic peptide MS2
200
400
600
800
1000
m/z
Fragment ion annotation using MaxQuant
TFQKKTKEm_2+ vs Prosit prediction
20171007_QX0_MaPe_SA_P509_NEO_3_OP1_3.raw Scan 4432
SVM Score 0.01 Q−Value 0
Endogenous MS2
Rel. intensity [a.u.]
1.0
0.5
0.0
−0.5
−1.0
y1
b2
y2
y6++
b3
y3
b7++
y7++
b4
b8++
y4
y8++
b5
y5
b6
y6
b7
y7
b8
Spectral Angle = 0.833
Prosit predicted
200
400
600
800
1000
m/z
Fragment ion annotation using Prosit ions

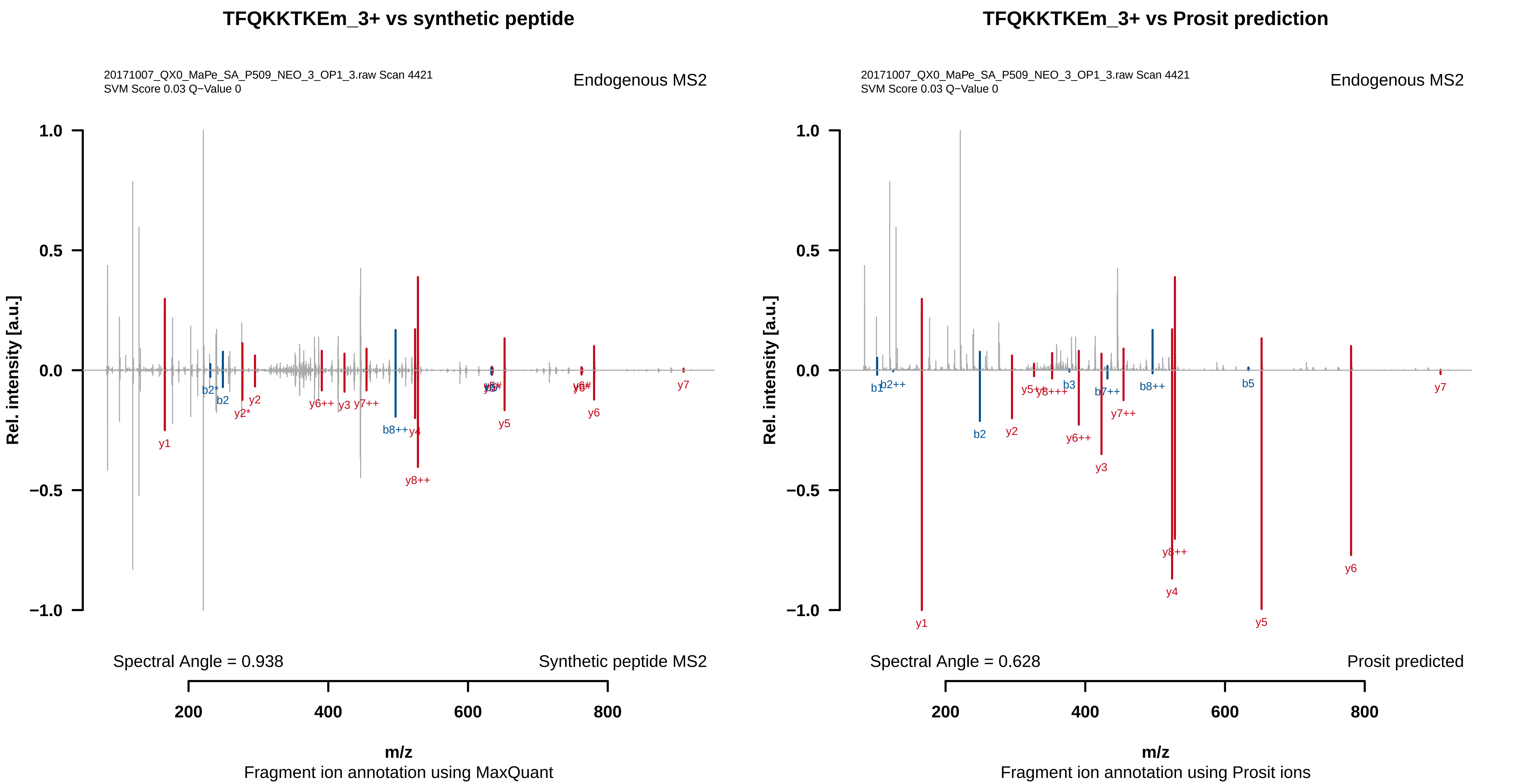

TFQKKTKEm_3+ vs synthetic peptide
20171007_QX0_MaPe_SA_P509_NEO_3_OP1_3.raw Scan 4421
SVM Score 0.03 Q−Value 0
Endogenous MS2
Rel. intensity [a.u.]
1.0
0.5
0.0
−0.5
−1.0
b2*
b2
y2*
y2
y1
y6++
y3
y7++
b8++
y4
y8++
y5
y6
y7
Spectral Angle = 0.938
Synthetic peptide MS2
200
400
600
800
m/z
Fragment ion annotation using MaxQuant
TFQKKTKEm_3+ vs Prosit prediction
20171007_QX0_MaPe_SA_P509_NEO_3_OP1_3.raw Scan 4421
SVM Score 0.03 Q−Value 0
Endogenous MS2
Rel. intensity [a.u.]
1.0
0.5
0.0
−0.5
−1.0
b1
b2++
b2
y2
b3
b7++
b8++
b5
y7
y6++
y7++
y3
y8++
y4
y6
y1
y5
Spectral Angle = 0.628
Prosit predicted
200
400
600
800
m/z
Fragment ion annotation using Prosit ions

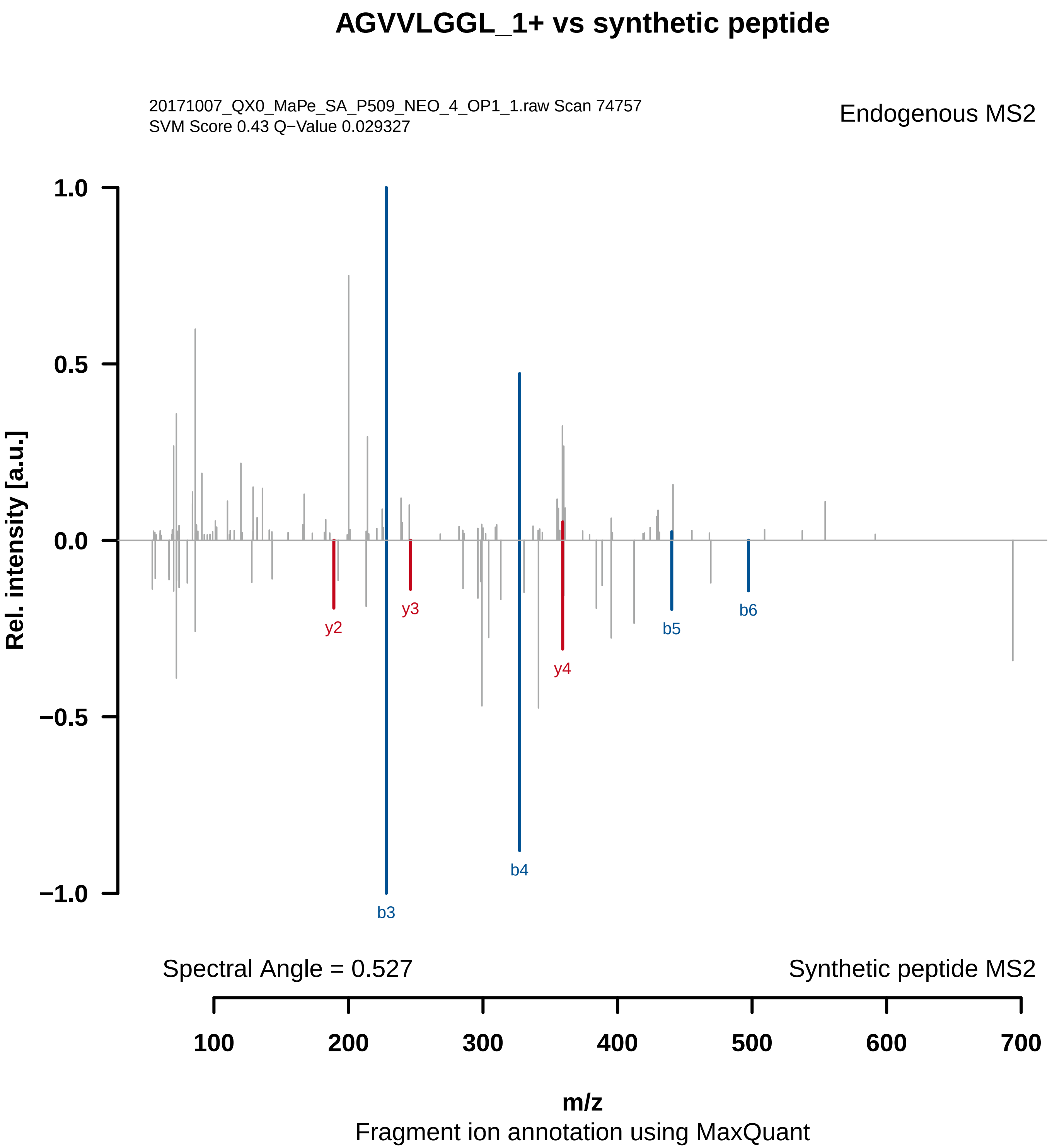
AGVVLGGL_1+ vs synthetic peptide
20171007_QX0_MaPe_SA_P509_NEO_4_OP1_1.raw Scan 74757
SVM Score 0.43 Q−Value 0.029327
Endogenous MS2
Rel. intensity [a.u.]
1.0
0.5
0.0
−0.5
−1.0
y2
y3
y4
b3
b4
b5
b6
Spectral Angle = 0.527
Synthetic peptide MS2
100
200
300
400
500
600
700
m/z
Fragment ion annotation using MaxQuant

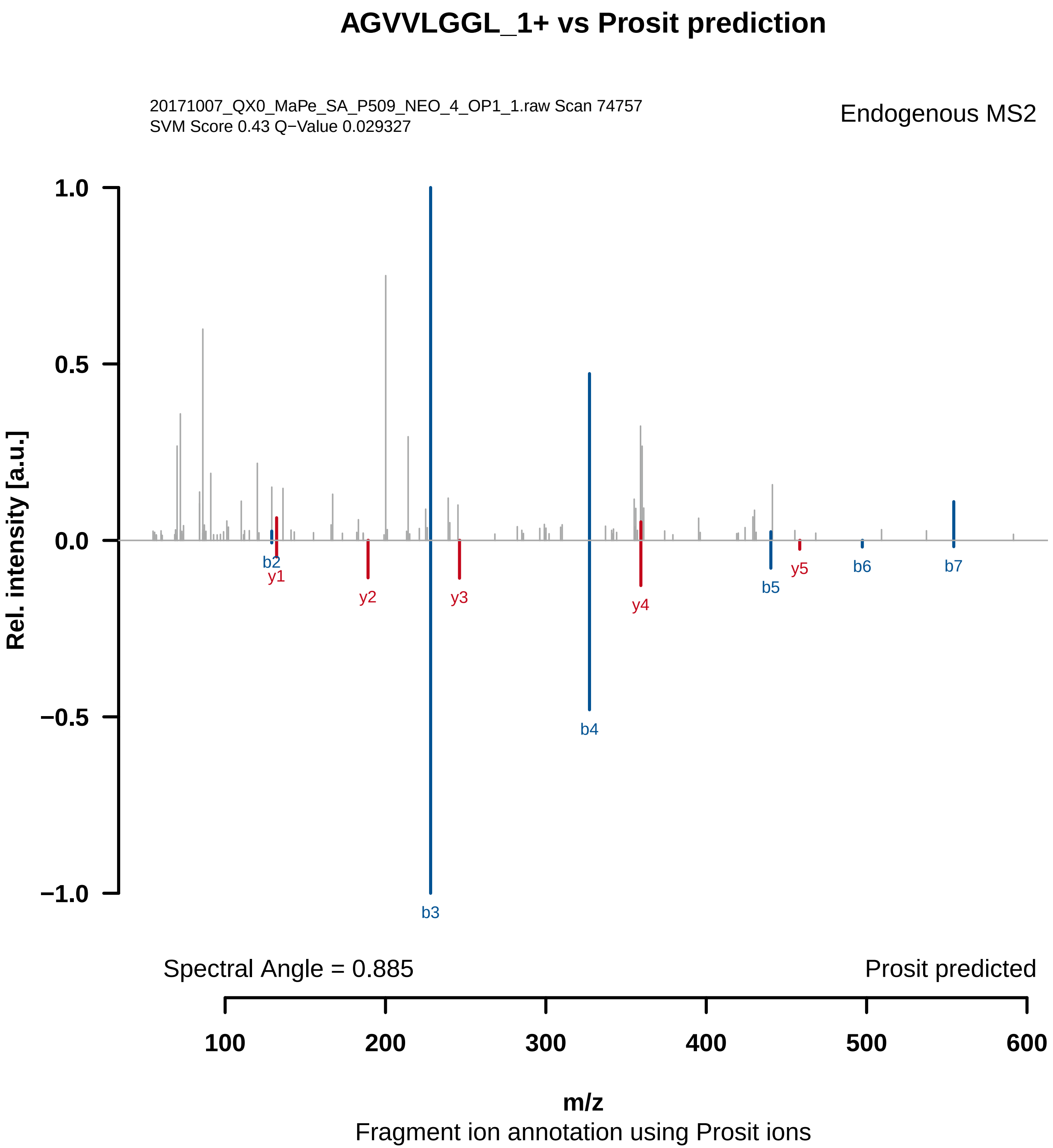
AGVVLGGL_1+ vs Prosit prediction
20171007_QX0_MaPe_SA_P509_NEO_4_OP1_1.raw Scan 74757
SVM Score 0.43 Q−Value 0.029327
Endogenous MS2
Rel. intensity [a.u.]
1.0
0.5
0.0
−0.5
−1.0
b2
y1
y2
y3
y4
b5
y5
b6
b7
b3
b4
Spectral Angle = 0.885
Prosit predicted
100
200
300
400
500
600
m/z
Fragment ion annotation using Prosit ions

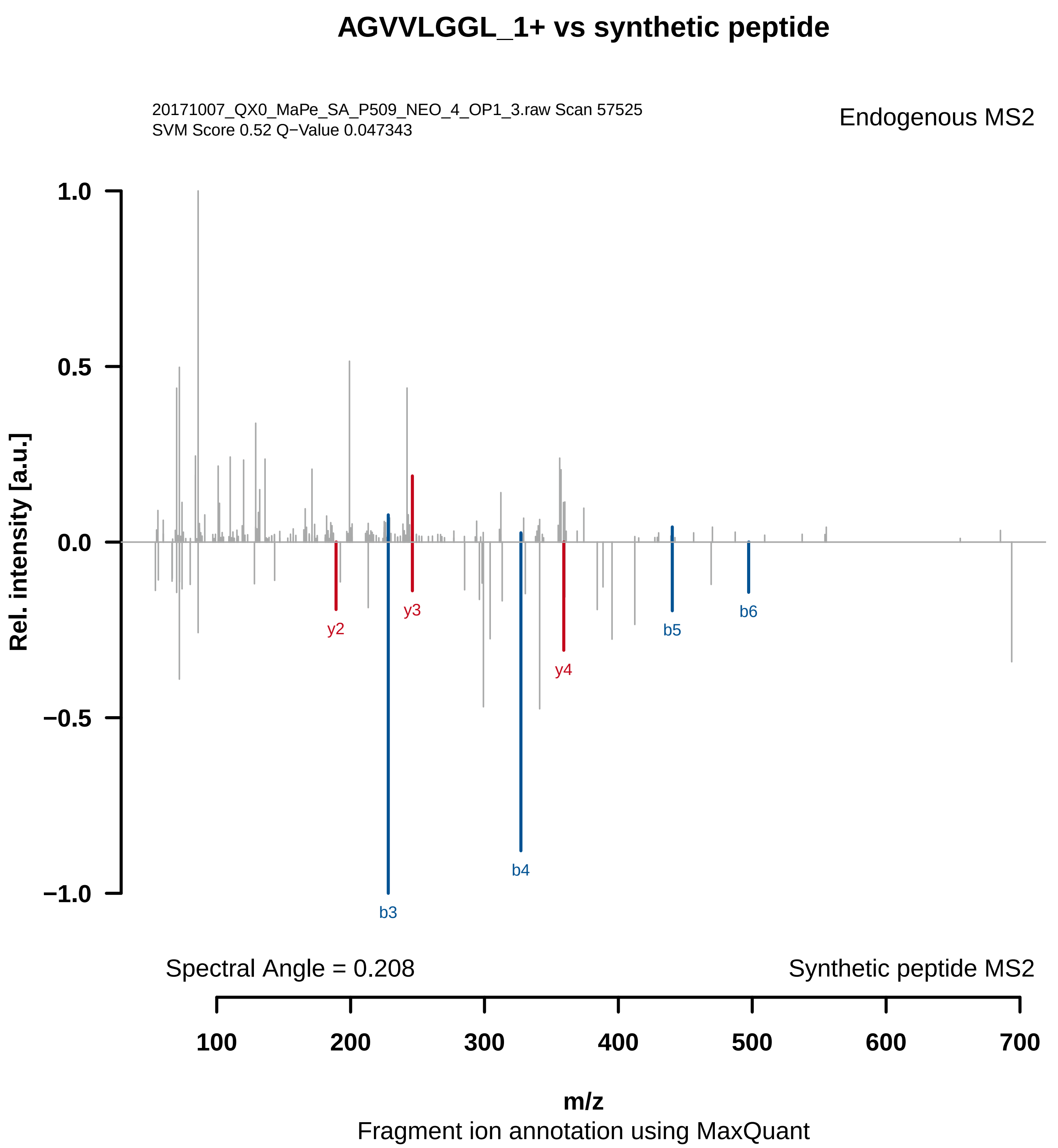
AGVVLGGL_1+ vs synthetic peptide
20171007_QX0_MaPe_SA_P509_NEO_4_OP1_3.raw Scan 57525
SVM Score 0.52 Q−Value 0.047343
Endogenous MS2
Rel. intensity [a.u.]
1.0
0.5
0.0
−0.5
−1.0
y2
y3
y4
b3
b4
b5
b6
Spectral Angle = 0.208
Synthetic peptide MS2
100
200
300
400
500
600
700
m/z
Fragment ion annotation using MaxQuant

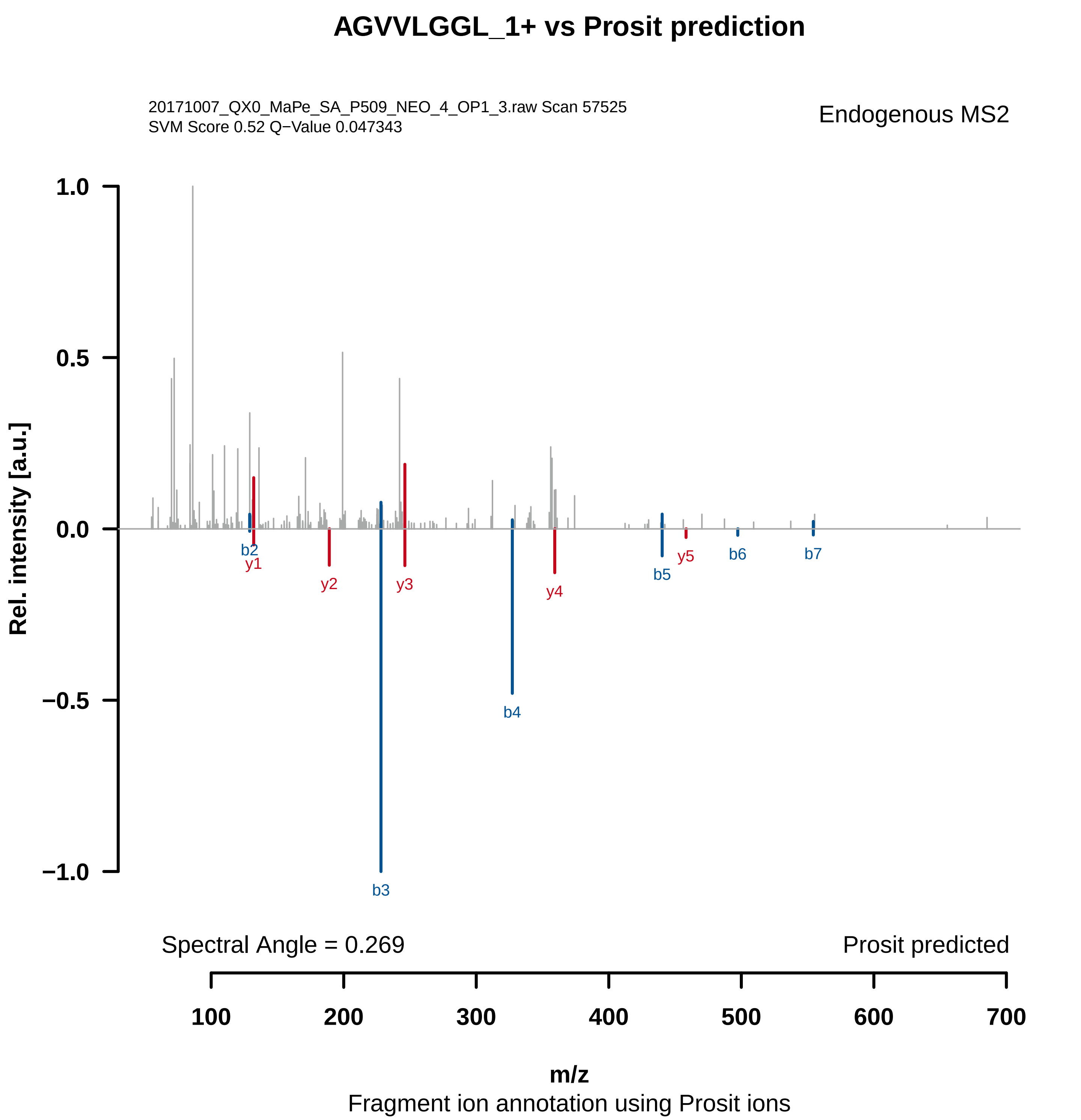
AGVVLGGL_1+ vs Prosit prediction
20171007_QX0_MaPe_SA_P509_NEO_4_OP1_3.raw Scan 57525
SVM Score 0.52 Q−Value 0.047343
Endogenous MS2
Rel. intensity [a.u.]
1.0
0.5
0.0
−0.5
−1.0
b2
y1
y2
y3
y4
y5
b3
b4
b5
b6
b7
Spectral Angle = 0.269
Prosit predicted
100
200
300
400
500
600
700
m/z
Fragment ion annotation using Prosit ions

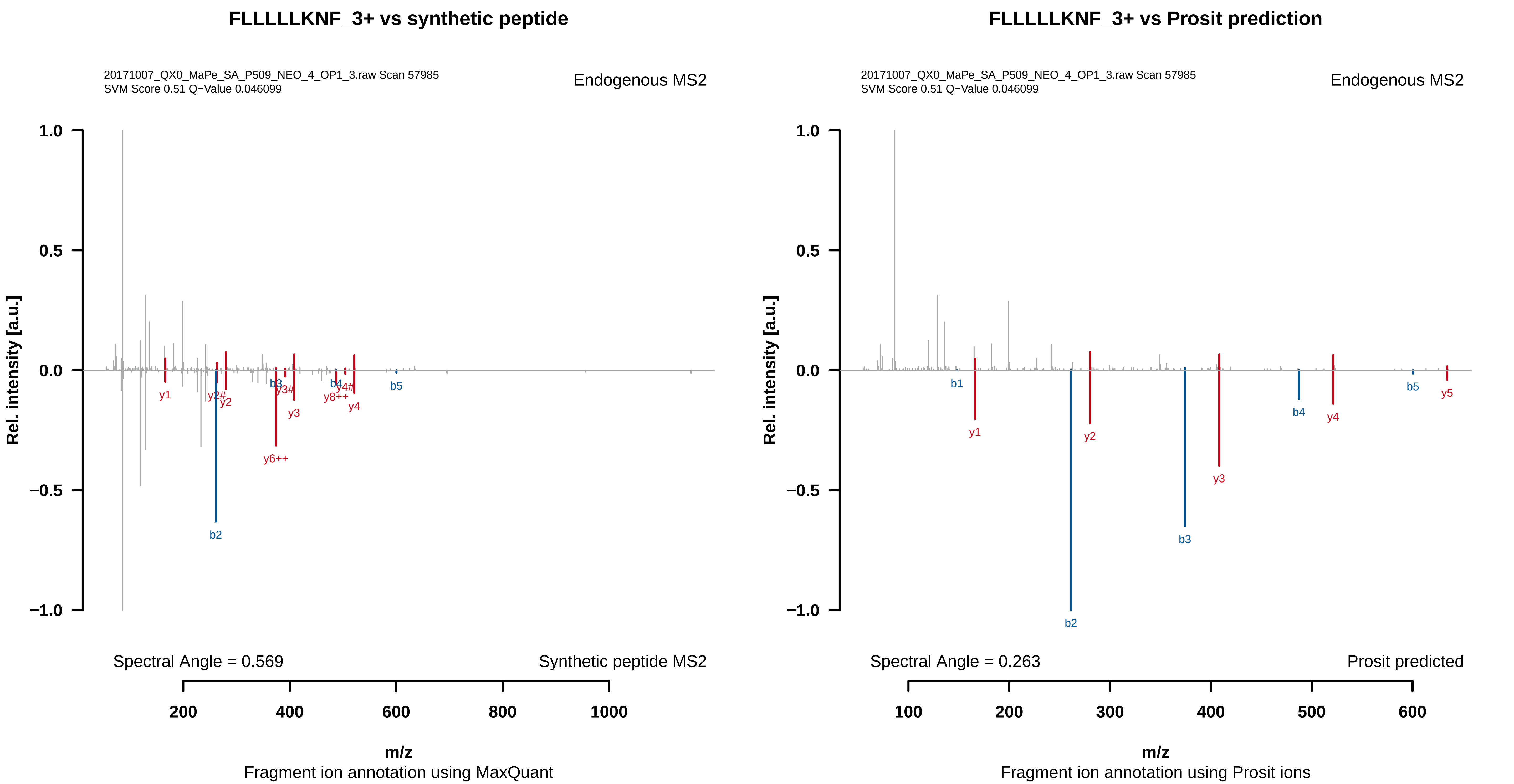
FLLLLLKNF_3+ vs synthetic peptide
20171007_QX0_MaPe_SA_P509_NEO_4_OP1_3.raw Scan 57985
SVM Score 0.51 Q−Value 0.046099
Endogenous MS2
Rel. intensity [a.u.]
1.0
0.5
0.0
−0.5
−1.0
y1
y2#
y2
b2
b3
y3#
y6++
y3
b4
y4#
y8++
y4
b5
Spectral Angle = 0.569
Synthetic peptide MS2
200
400
600
800
1000
m/z
Fragment ion annotation using MaxQuant
FLLLLLKNF_3+ vs Prosit prediction
20171007_QX0_MaPe_SA_P509_NEO_4_OP1_3.raw Scan 57985
SVM Score 0.51 Q−Value 0.046099
Endogenous MS2
Rel. intensity [a.u.]
1.0
0.5
0.0
−0.5
−1.0
b1
y1
b2
y2
b3
y3
b4
y4
b5
y5
Spectral Angle = 0.263
Prosit predicted
100
200
300
400
500
600
m/z
Fragment ion annotation using Prosit ions

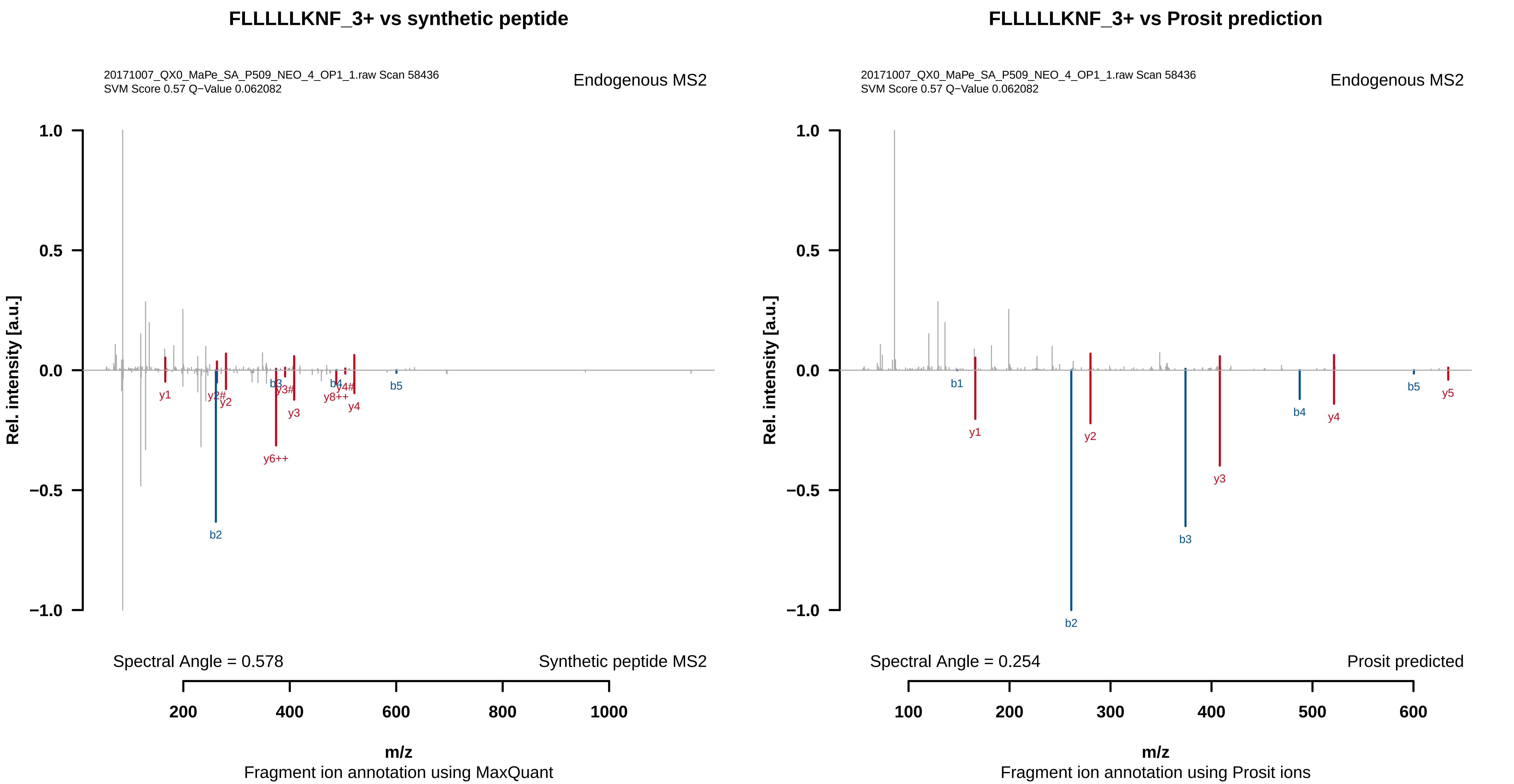
FLLLLLKNF_3+ vs synthetic peptide
20171007_QX0_MaPe_SA_P509_NEO_4_OP1_1.raw Scan 58436
SVM Score 0.57 Q−Value 0.062082
Endogenous MS2
Rel. intensity [a.u.]
1.0
0.5
0.0
−0.5
−1.0
y1
y2#
y2
b2
b3
y3#
y3
y6++
b4
y4#
y8++
y4
b5
Spectral Angle = 0.578
Synthetic peptide MS2
200
400
600
800
1000
m/z
Fragment ion annotation using MaxQuant
FLLLLLKNF_3+ vs Prosit prediction
20171007_QX0_MaPe_SA_P509_NEO_4_OP1_1.raw Scan 58436
SVM Score 0.57 Q−Value 0.062082
Endogenous MS2
Rel. intensity [a.u.]
1.0
0.5
0.0
−0.5
−1.0
b1
y1
b2
y2
b3
y3
b4
y4
b5
y5
Spectral Angle = 0.254
Prosit predicted
100
200
300
400
500
600
m/z
Fragment ion annotation using Prosit ions

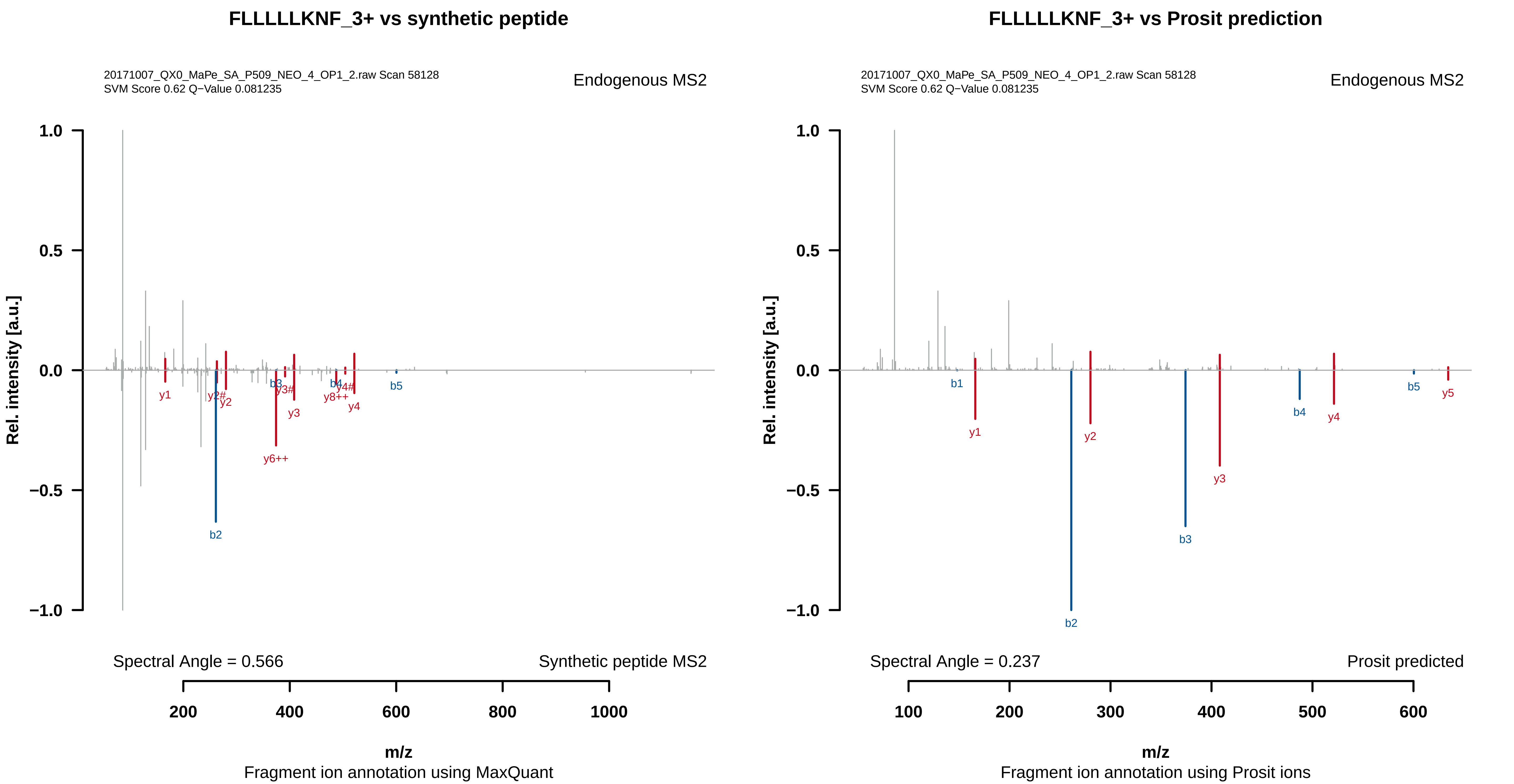

FLLLLLKNF_3+ vs synthetic peptide
20171007_QX0_MaPe_SA_P509_NEO_4_OP1_2.raw Scan 58128
SVM Score 0.62 Q−Value 0.081235
Endogenous MS2
Rel. intensity [a.u.]
1.0
0.5
0.0
−0.5
−1.0
y1
y2#
y2
b2
b3
y3#
y3
y6++
b4
y4#
y8++
y4
b5
Spectral Angle = 0.566
Synthetic peptide MS2
200
400
600
800
1000
m/z
Fragment ion annotation using MaxQuant
FLLLLLKNF_3+ vs Prosit prediction
20171007_QX0_MaPe_SA_P509_NEO_4_OP1_2.raw Scan 58128
SVM Score 0.62 Q−Value 0.081235
Endogenous MS2
Rel. intensity [a.u.]
1.0
0.5
0.0
−0.5
−1.0
b1
y1
b2
y2
b3
y3
b4
y4
b5
y5
Spectral Angle = 0.237
Prosit predicted
100
200
300
400
500
600
m/z
Fragment ion annotation using Prosit ions

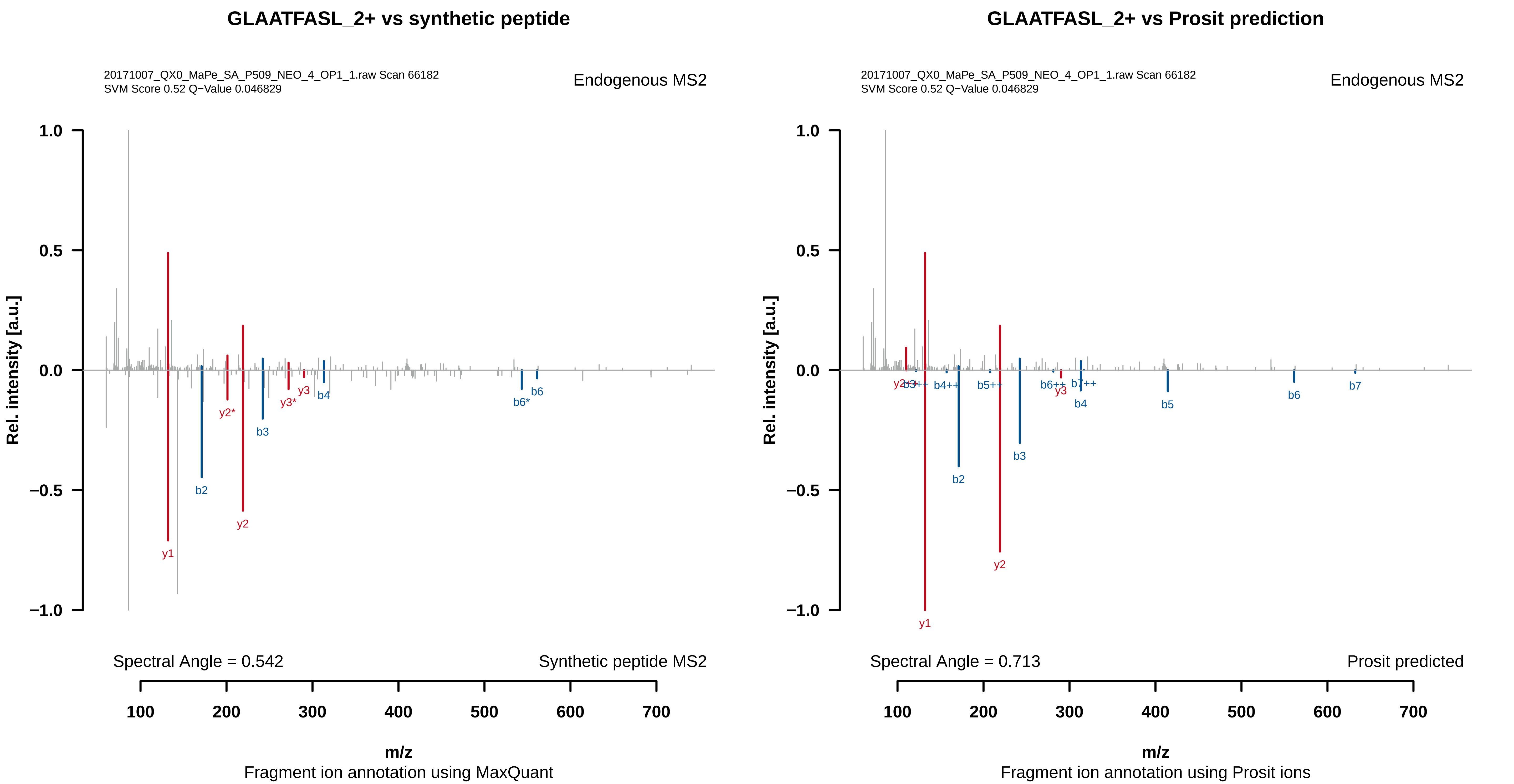
GLAATFASL_2+ vs synthetic peptide
20171007_QX0_MaPe_SA_P509_NEO_4_OP1_1.raw Scan 66182
SVM Score 0.52 Q−Value 0.046829
Endogenous MS2
Rel. intensity [a.u.]
1.0
0.5
0.0
−0.5
−1.0
y1
b2
y2*
y2
b3
y3*
y3
b4
b6*
b6
Spectral Angle = 0.542
Synthetic peptide MS2
100
200
300
400
500
600
700
m/z
Fragment ion annotation using MaxQuant
GLAATFASL_2+ vs Prosit prediction
20171007_QX0_MaPe_SA_P509_NEO_4_OP1_1.raw Scan 66182
SVM Score 0.52 Q−Value 0.046829
Endogenous MS2
Rel. intensity [a.u.]
1.0
0.5
0.0
−0.5
−1.0
y1
b2
b3
y2
b4++
b5++
b6++
b7++
y3
b4
b5
b6
b7
Spectral Angle = 0.713
Prosit predicted
100
200
300
400
500
600
700
m/z
Fragment ion annotation using Prosit ions

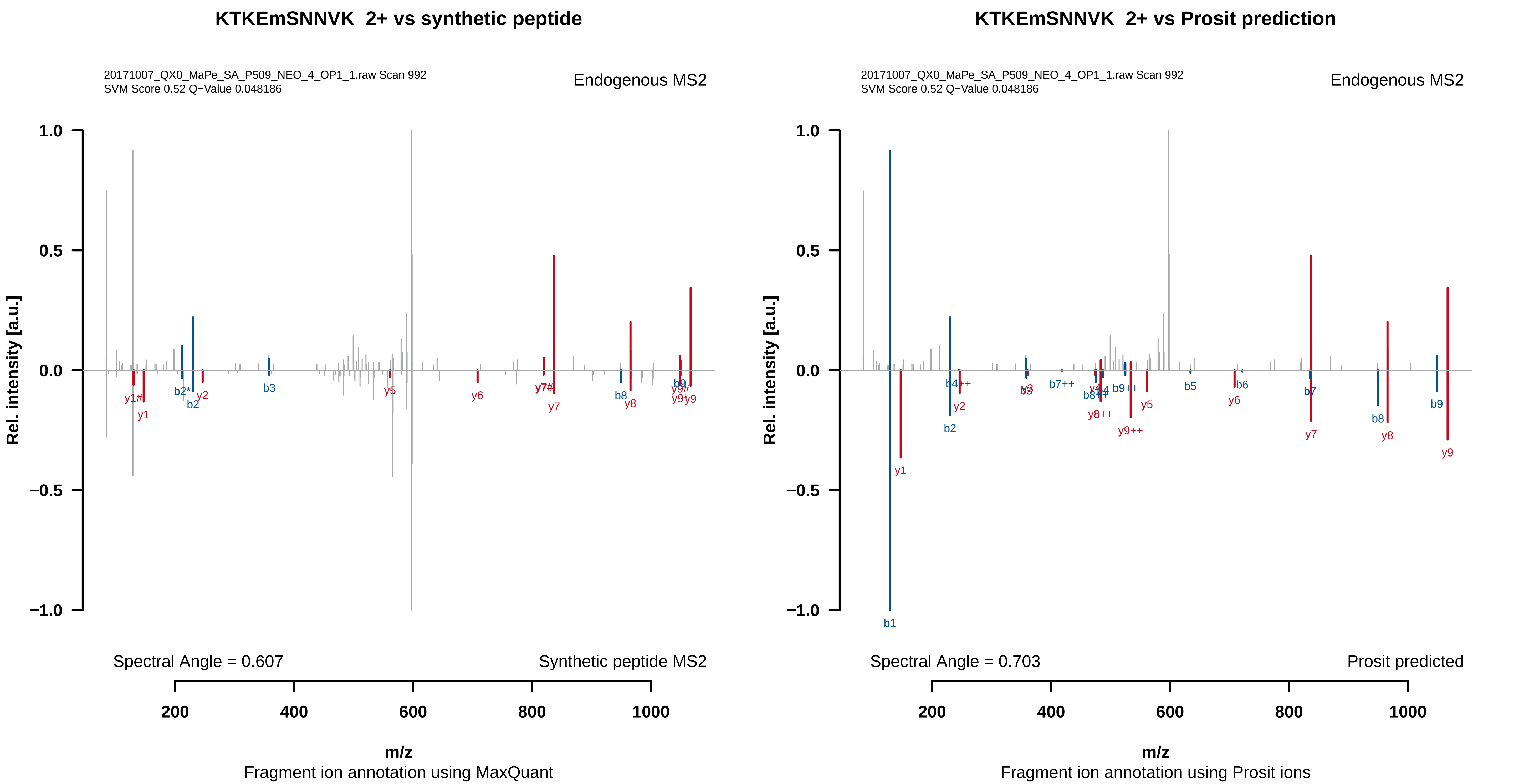
KTKEmSNNVK_2+ vs synthetic peptide
20171007_QX0_MaPe_SA_P509_NEO_4_OP1_1.raw Scan 992
SVM Score 0.52 Q−Value 0.048186
Endogenous MS2
Rel. intensity [a.u.]
1.0
0.5
0.0
−0.5
−1.0
y1#
y1
b2*
b2
y2
b3
y5
y6
y7#
y7
b8
y8
b9
y9#
y9*
y9
Spectral Angle = 0.607
Synthetic peptide MS2
200
400
600
800
1000
m/z
Fragment ion annotation using MaxQuant
KTKEmSNNVK_2+ vs Prosit prediction
20171007_QX0_MaPe_SA_P509_NEO_4_OP1_1.raw Scan 992
SVM Score 0.52 Q−Value 0.048186
Endogenous MS2
Rel. intensity [a.u.]
1.0
0.5
0.0
−0.5
−1.0
b1
y1
b2
b4++
y2
b3
b7++
y8++
y9++
b9++
y5
b5
b6
y6
b7
y7
b8
y8
b9
y9
Spectral Angle = 0.703
Prosit predicted
200
400
600
800
1000
m/z
Fragment ion annotation using Prosit ions

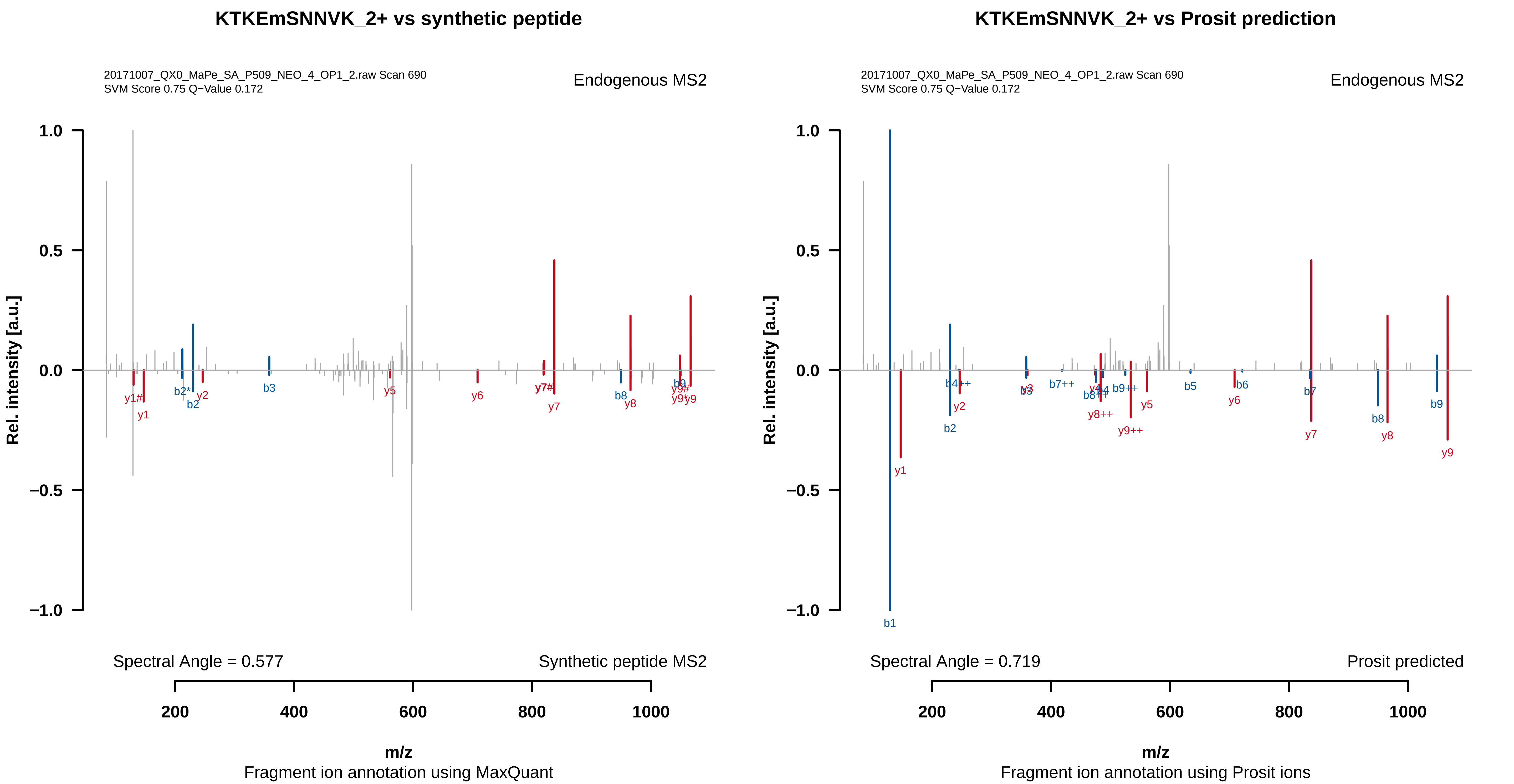

KTKEmSNNVK_2+ vs synthetic peptide
20171007_QX0_MaPe_SA_P509_NEO_4_OP1_2.raw Scan 690
SVM Score 0.75 Q−Value 0.172
Endogenous MS2
Rel. intensity [a.u.]
1.0
0.5
0.0
−0.5
−1.0
Spectral Angle = 0.577
Synthetic peptide MS2
200
400
600
800
1000
m/z
Fragment ion annotation using MaxQuant
KTKEmSNNVK_2+ vs Prosit prediction
20171007_QX0_MaPe_SA_P509_NEO_4_OP1_2.raw Scan 690
SVM Score 0.75 Q−Value 0.172
Endogenous MS2
Rel. intensity [a.u.]
Spectral Angle = 0.719
Prosit predicted
m/z
Fragment ion annotation using Prosit ions

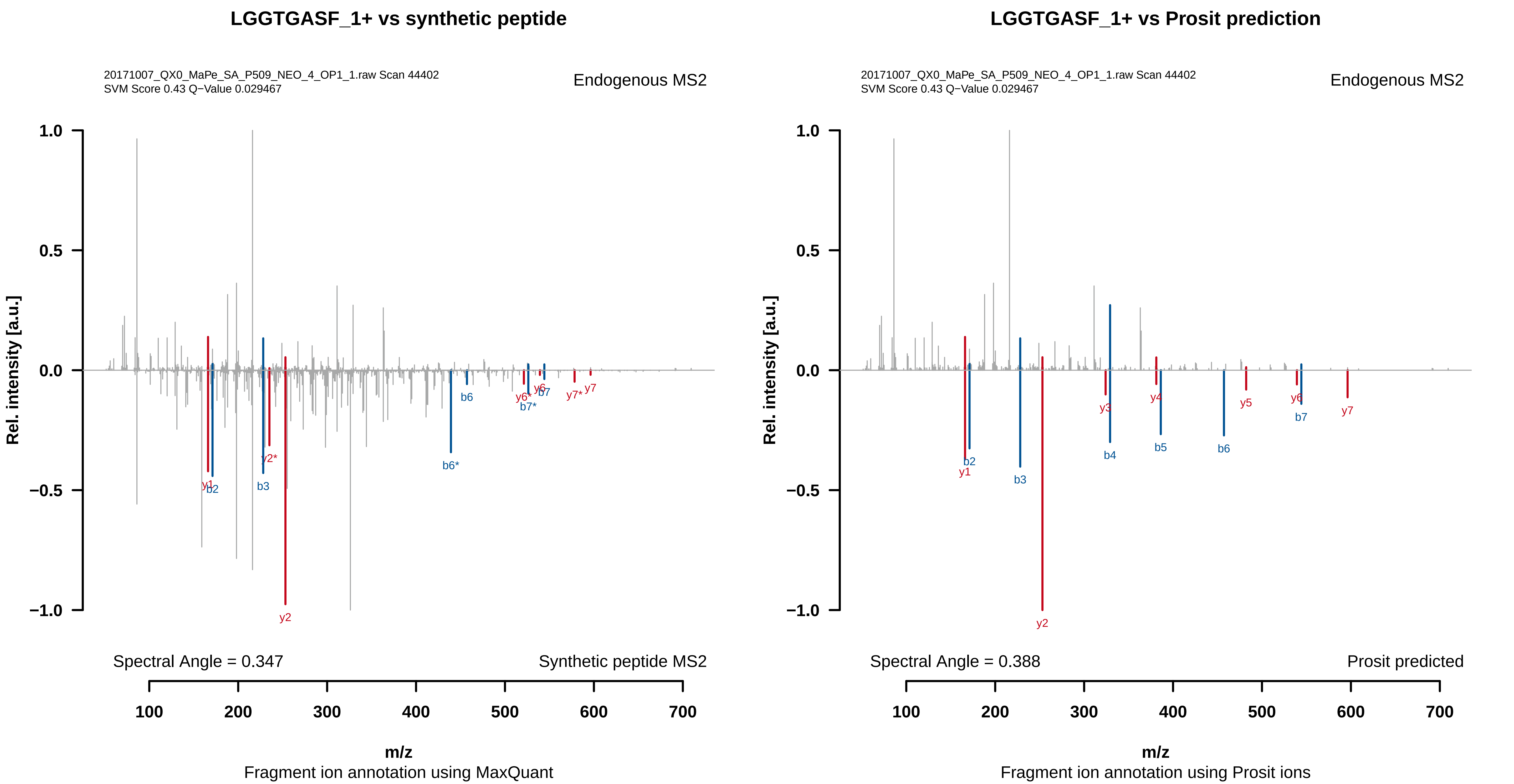

LGGTGASF_1+ vs synthetic peptide
20171007_QX0_MaPe_SA_P509_NEO_4_OP1_1.raw Scan 44402
SVM Score 0.43 Q−Value 0.029467
Endogenous MS2
Rel. intensity [a.u.]
1.0
0.5
0.0
−0.5
−1.0
y1
b2
b3
y2*
y2
b6*
b6
y6*
b7*
y6
b7
y7*
y7
Spectral Angle = 0.347
Synthetic peptide MS2
100
200
300
400
500
600
700
m/z
Fragment ion annotation using MaxQuant
LGGTGASF_1+ vs Prosit prediction
20171007_QX0_MaPe_SA_P509_NEO_4_OP1_1.raw Scan 44402
SVM Score 0.43 Q−Value 0.029467
Endogenous MS2
Rel. intensity [a.u.]
b2
y1
b3
y2
y3
b4
y4
b5
b6
y5
y6
b7
y7
Spectral Angle = 0.388
Prosit predicted
m/z
Fragment ion annotation using Prosit ions

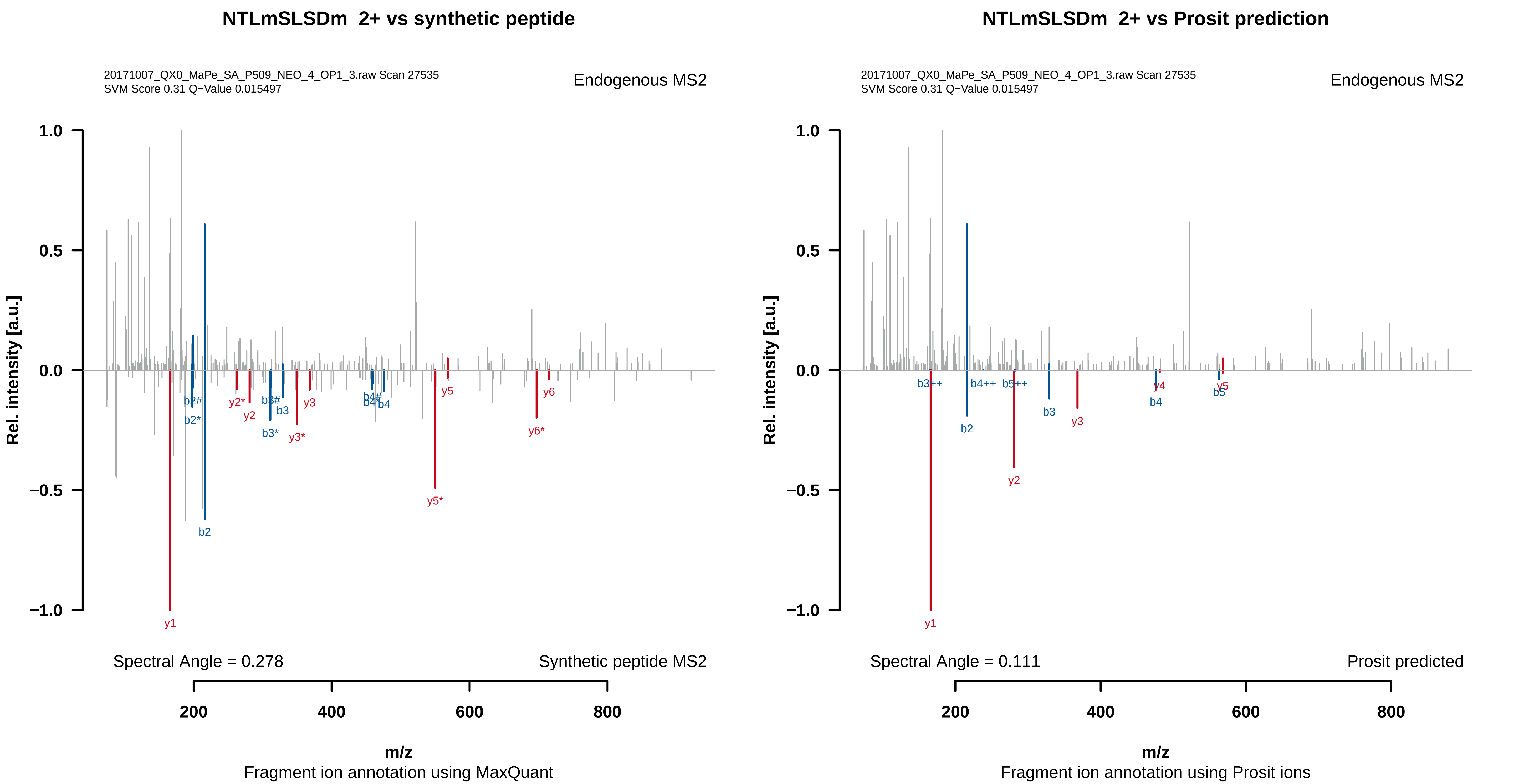
NTLmSLSDm_2+ vs synthetic peptide
20171007_QX0_MaPe_SA_P509_NEO_4_OP1_3.raw Scan 27535
SVM Score 0.31 Q−Value 0.015497
Endogenous MS2
Rel. intensity [a.u.]
Spectral Angle = 0.278
Synthetic peptide MS2
m/z
Fragment ion annotation using MaxQuant
NTLmSLSDm_2+ vs Prosit prediction
20171007_QX0_MaPe_SA_P509_NEO_4_OP1_3.raw Scan 27535
SVM Score 0.31 Q−Value 0.015497
Endogenous MS2
Rel. intensity [a.u.]
Spectral Angle = 0.111
Prosit predicted
m/z
Fragment ion annotation using Prosit ions

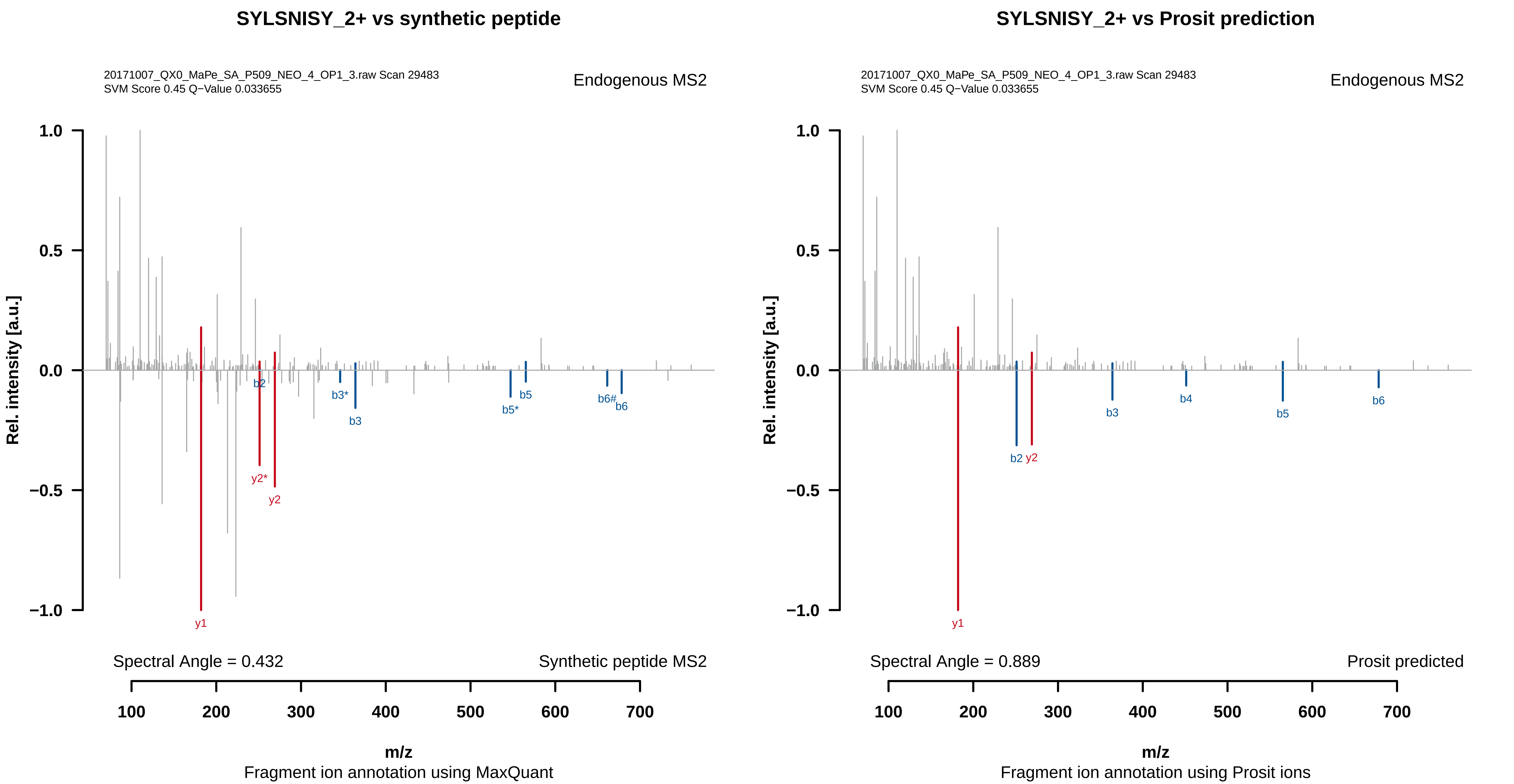

SYLSNISY_2+ vs synthetic peptide
20171007_QX0_MaPe_SA_P509_NEO_4_OP1_3.raw Scan 29483
SVM Score 0.45 Q−Value 0.033655
Endogenous MS2
Rel. intensity [a.u.]
1.0
0.5
0.0
−0.5
−1.0
b2
b3*
b3
b5
b5*
b6#
b6
y2*
y2
y1
Spectral Angle = 0.432
Synthetic peptide MS2
100
200
300
400
500
600
700
m/z
Fragment ion annotation using MaxQuant
SYLSNISY_2+ vs Prosit prediction
20171007_QX0_MaPe_SA_P509_NEO_4_OP1_3.raw Scan 29483
SVM Score 0.45 Q−Value 0.033655
Endogenous MS2
Rel. intensity [a.u.]
1.0
0.5
0.0
−0.5
−1.0
b3
b4
b5
b6
b2
y2
y1
Spectral Angle = 0.889
Prosit predicted
100
200
300
400
500
600
700
m/z
Fragment ion annotation using Prosit ions

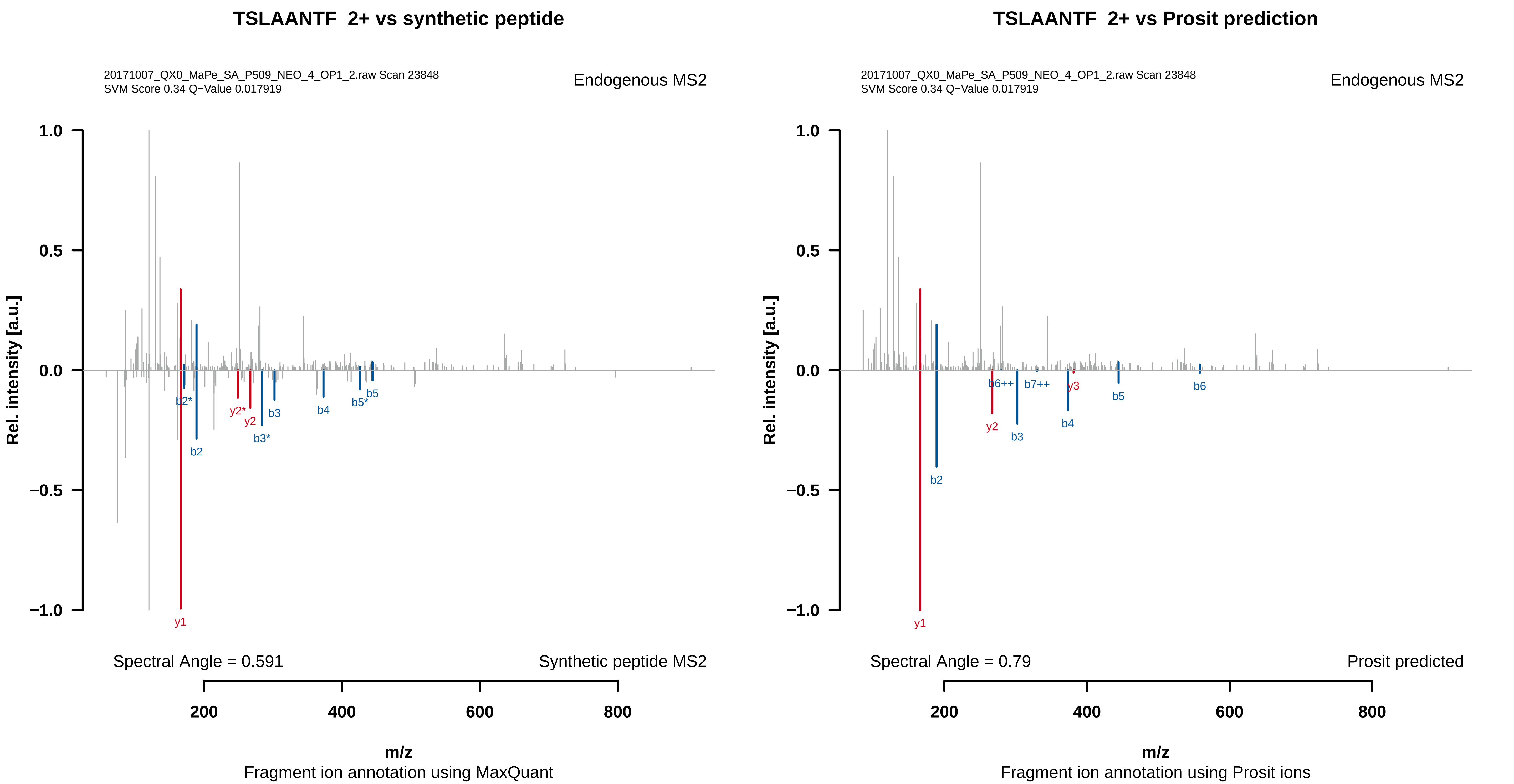

TSLAANTF_2+ vs synthetic peptide
20171007_QX0_MaPe_SA_P509_NEO_4_OP1_2.raw Scan 23848
SVM Score 0.34 Q−Value 0.017919
Endogenous MS2
Rel. intensity [a.u.]
1.0
0.5
0.0
−0.5
−1.0
b2*
b2
y2*
y2
b3
b3*
b4
b5*
b5
y1
Spectral Angle = 0.591
Synthetic peptide MS2
200
400
600
800
m/z
Fragment ion annotation using MaxQuant
TSLAANTF_2+ vs Prosit prediction
20171007_QX0_MaPe_SA_P509_NEO_4_OP1_2.raw Scan 23848
SVM Score 0.34 Q−Value 0.017919
Endogenous MS2
Rel. intensity [a.u.]
b6++
b7++
y3
b5
b6
y2
b3
b4
b2
y1
Spectral Angle = 0.79
Prosit predicted
m/z
Fragment ion annotation using Prosit ions

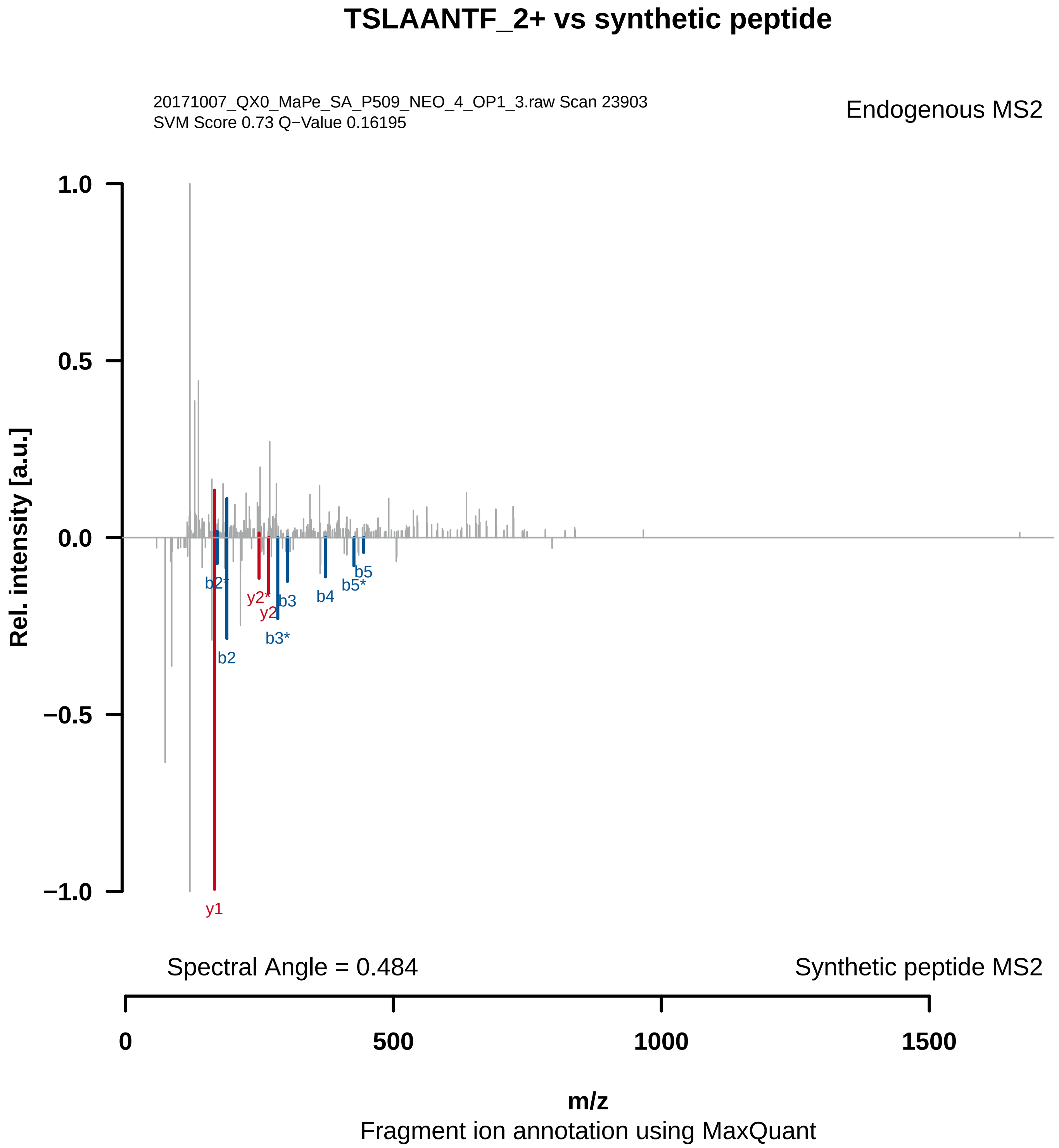

TSLAANTF_2+ vs synthetic peptide
20171007_QX0_MaPe_SA_P509_NEO_4_OP1_3.raw Scan 23903
SVM Score 0.73 Q−Value 0.16195
Endogenous MS2
Rel. intensity [a.u.]
1.0
0.5
0.0
−0.5
−1.0
b2*
b2
y2*
y2
b3
b3*
b4
b5
b5*
y1
Spectral Angle = 0.484
Synthetic peptide MS2
0
500
1000
1500
m/z
Fragment ion annotation using MaxQuant

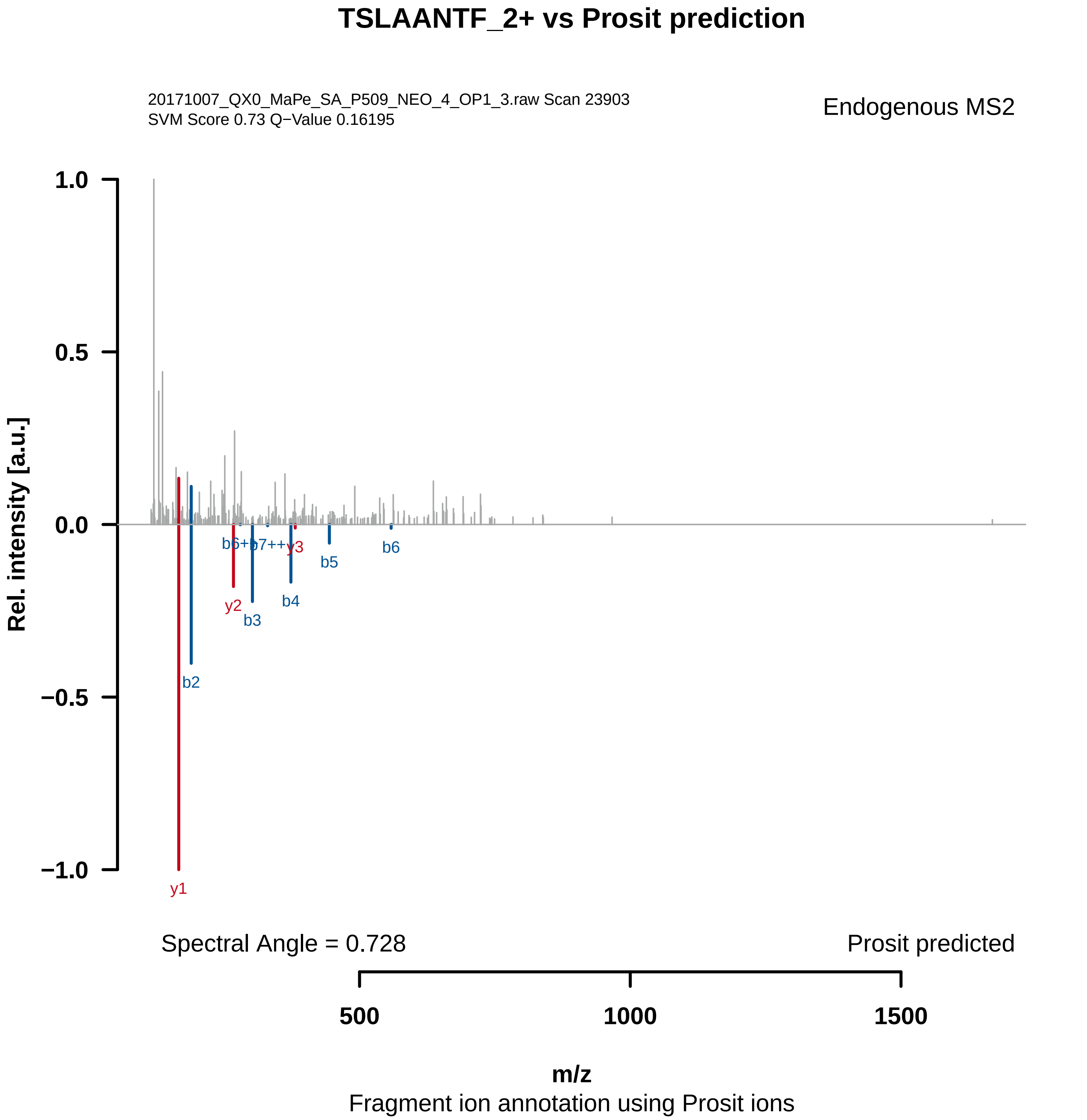

TSLAANTF_2+ vs Prosit prediction
20171007_QX0_MaPe_SA_P509_NEO_4_OP1_3.raw Scan 23903
SVM Score 0.73 Q−Value 0.16195
Endogenous MS2
Rel. intensity [a.u.]
1.0
0.5
0.0
−0.5
−1.0
b2
y2
b3
b4
b5
b6
y3
y1
Spectral Angle = 0.728
Prosit predicted
500
1000
1500
m/z
Fragment ion annotation using Prosit ions

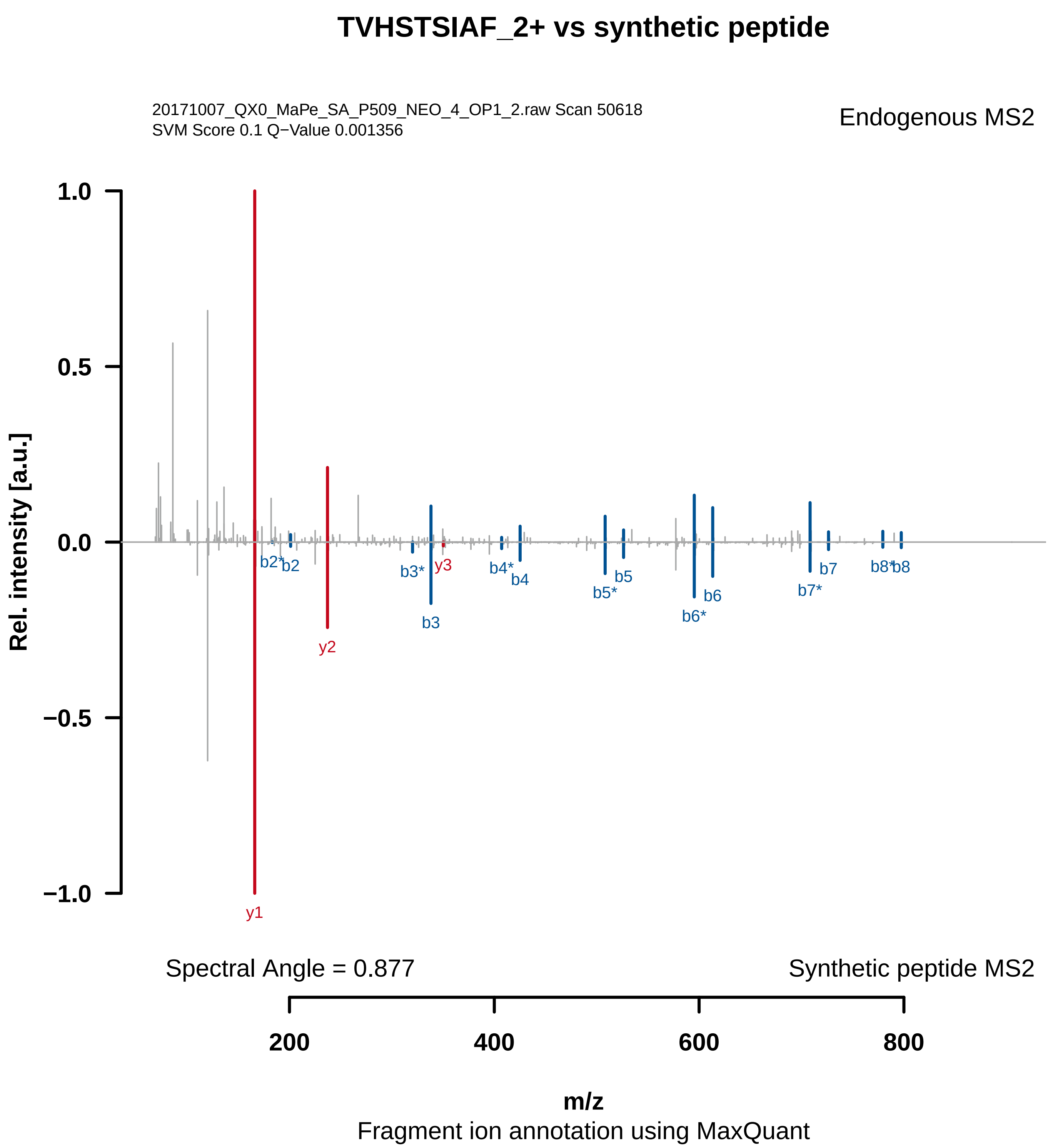
TVHSTSIAF_2+ vs synthetic peptide
20171007_QX0_MaPe_SA_P509_NEO_4_OP1_2.raw Scan 50618
SVM Score 0.1 Q−Value 0.001356
Endogenous MS2
Rel. intensity [a.u.]
1.0
0.5
0.0
−0.5
−1.0
b2*
b2
y2
b3*
y3
b3
b4*
b4
b5*
b5
b6*
b6
b7*
b7
b8*
b8
y1
Spectral Angle = 0.877
Synthetic peptide MS2
200
400
600
800
m/z
Fragment ion annotation using MaxQuant

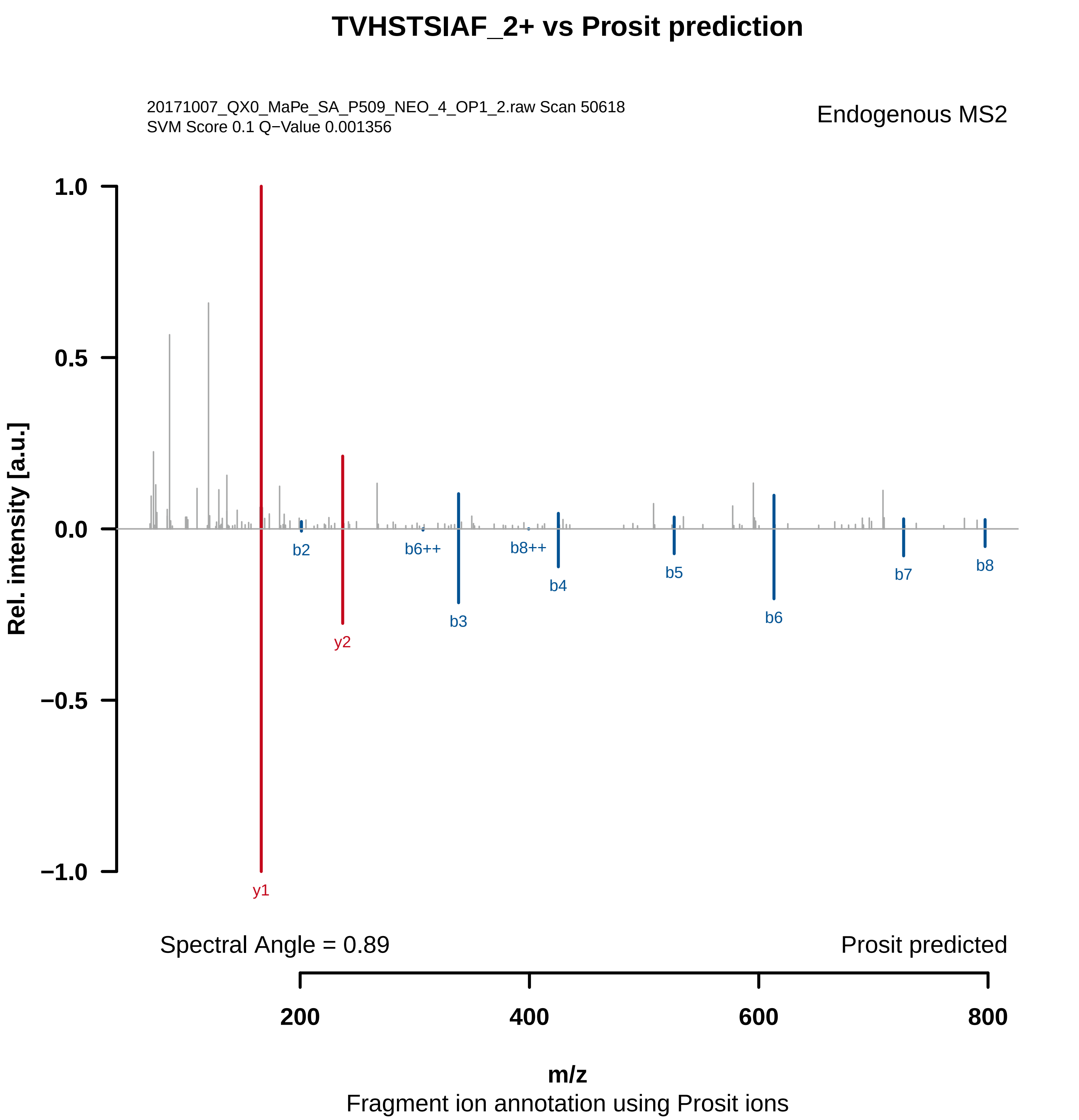
TVHSTSIAF_2+ vs Prosit prediction
20171007_QX0_MaPe_SA_P509_NEO_4_OP1_2.raw Scan 50618
SVM Score 0.1 Q−Value 0.001356
Endogenous MS2
Rel. intensity [a.u.]
1.0
0.5
0.0
−0.5
−1.0
b2
y2
b6++
b3
b8++
b4
b5
b6
b7
b8
y1
Spectral Angle = 0.89
Prosit predicted
200
400
600
800
m/z
Fragment ion annotation using Prosit ions

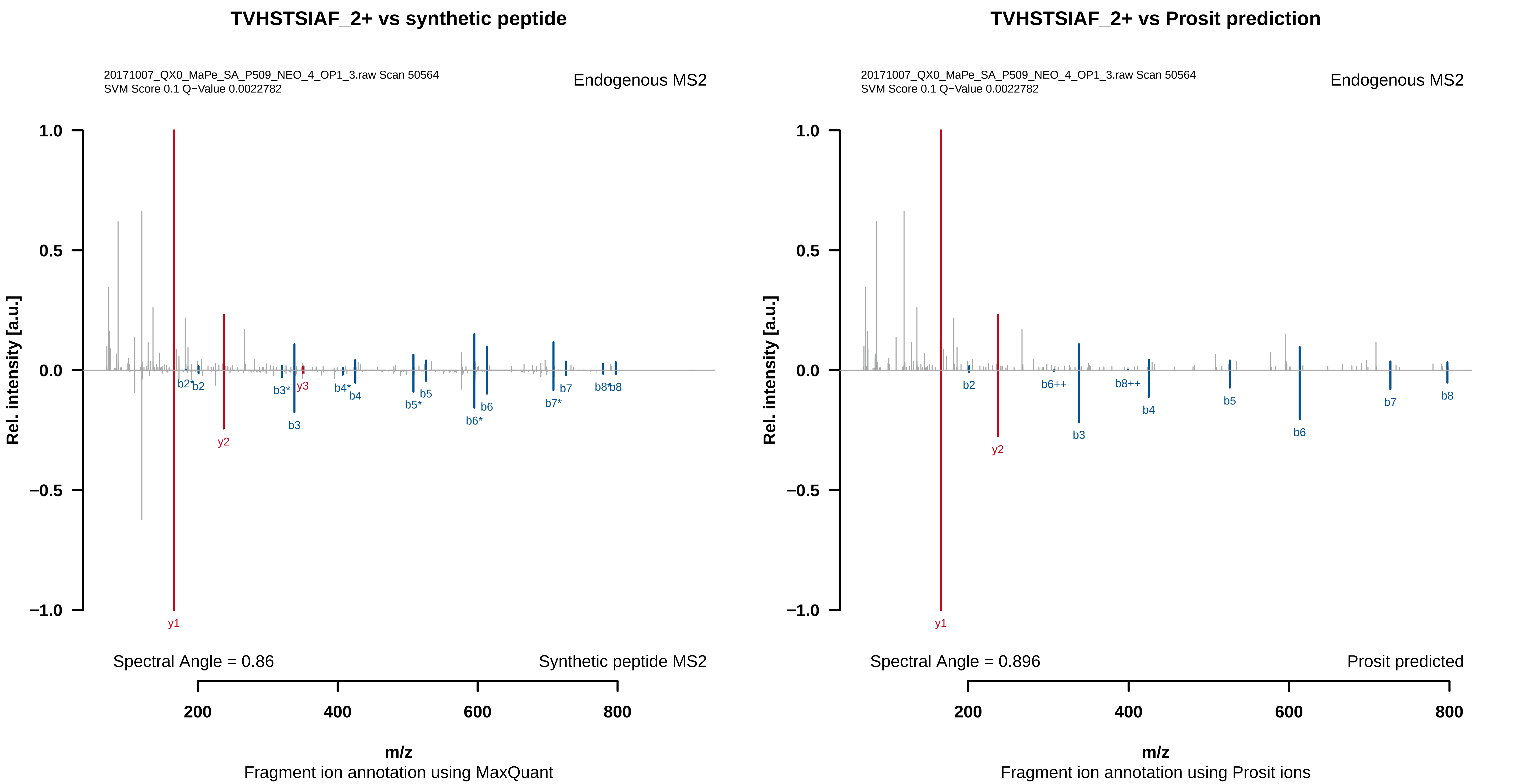

TVHSTSIAF_2+ vs synthetic peptide
20171007_QX0_MaPe_SA_P509_NEO_4_OP1_3.raw Scan 50564
SVM Score 0.1 Q−Value 0.0022782
Endogenous MS2
Rel. intensity [a.u.]
1.0
0.5
0.0
−0.5
−1.0
y1
b2*
b2
y2
b3*
b3
y3
b4*
b4
b5*
b5
b6*
b6
b7*
b7
b8*
b8
Spectral Angle = 0.86
Synthetic peptide MS2
200
400
600
800
m/z
Fragment ion annotation using MaxQuant
TVHSTSIAF_2+ vs Prosit prediction
20171007_QX0_MaPe_SA_P509_NEO_4_OP1_3.raw Scan 50564
SVM Score 0.1 Q−Value 0.0022782
Endogenous MS2
Rel. intensity [a.u.]
1.0
0.5
0.0
−0.5
−1.0
y1
b2
y2
b6++
b3
b8++
b4
b5
b6
b7
b8
Spectral Angle = 0.896
Prosit predicted
200
400
600
800
m/z
Fragment ion annotation using Prosit ions

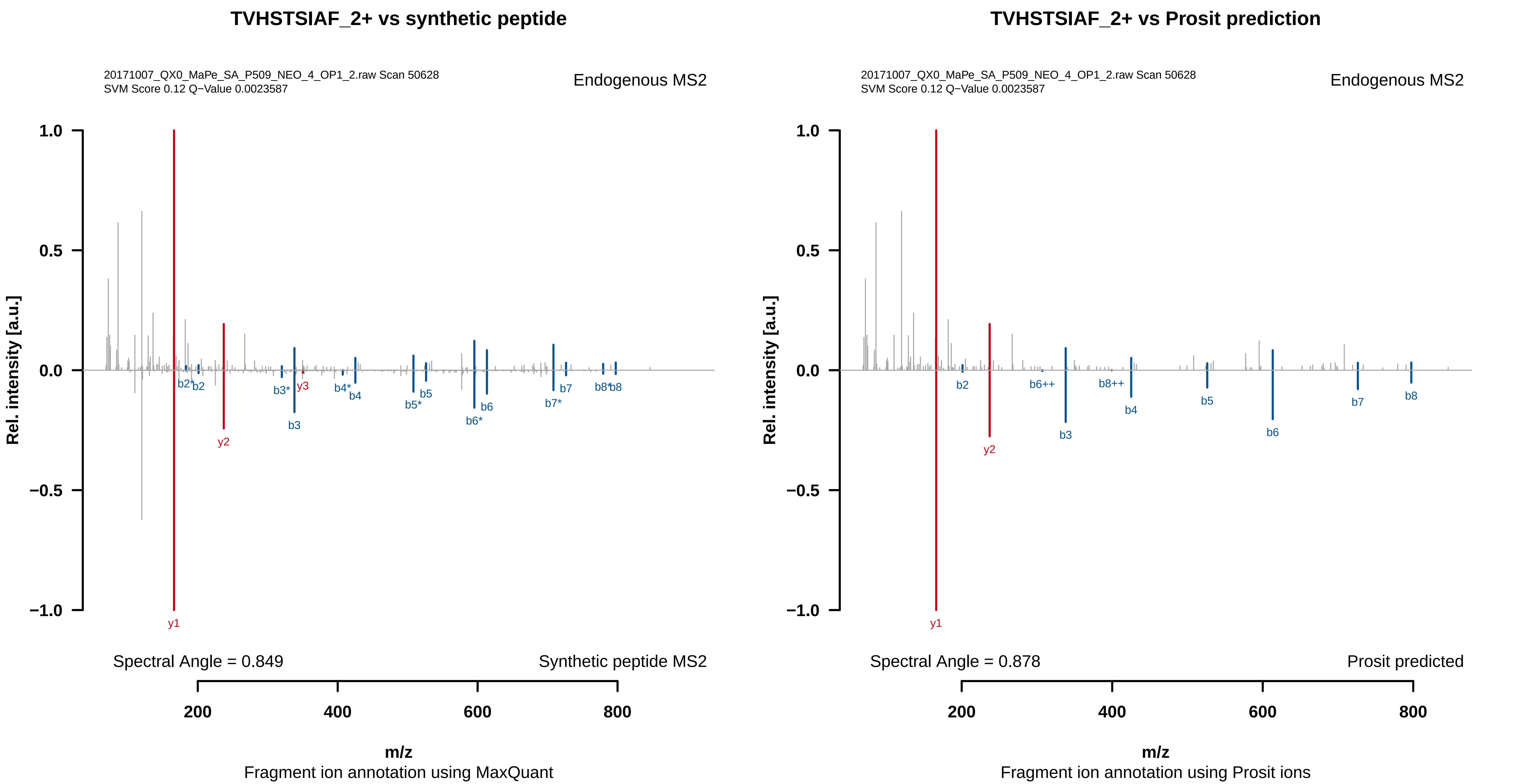

TVHSTSIAF_2+ vs synthetic peptide
20171007_QX0_MaPe_SA_P509_NEO_4_OP1_2.raw Scan 50628
SVM Score 0.12 Q−Value 0.0023587
Endogenous MS2
Rel. intensity [a.u.]
Spectral Angle = 0.849
Synthetic peptide MS2
m/z
Fragment ion annotation using MaxQuant
TVHSTSIAF_2+ vs Prosit prediction
20171007_QX0_MaPe_SA_P509_NEO_4_OP1_2.raw Scan 50628
SVM Score 0.12 Q−Value 0.0023587
Endogenous MS2
Rel. intensity [a.u.]
Spectral Angle = 0.878
Prosit predicted
m/z
Fragment ion annotation using Prosit ions

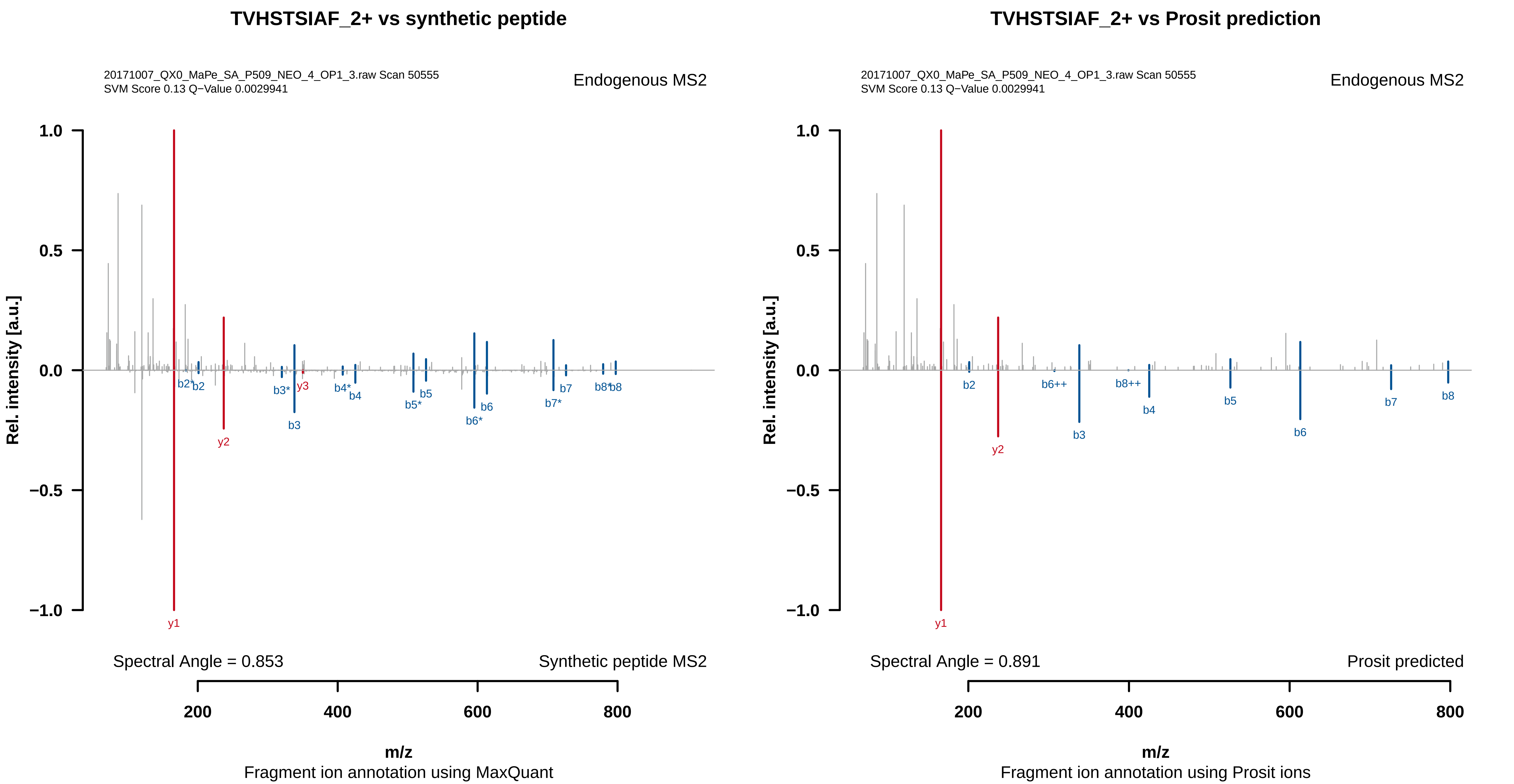

TVHSTSIAF_2+ vs synthetic peptide
20171007_QX0_MaPe_SA_P509_NEO_4_OP1_3.raw Scan 50555
SVM Score 0.13 Q−Value 0.0029941
Endogenous MS2
Rel. intensity [a.u.]
Spectral Angle = 0.853
Synthetic peptide MS2
m/z
Fragment ion annotation using MaxQuant
TVHSTSIAF_2+ vs Prosit prediction
20171007_QX0_MaPe_SA_P509_NEO_4_OP1_3.raw Scan 50555
SVM Score 0.13 Q−Value 0.0029941
Endogenous MS2
Rel. intensity [a.u.]
Spectral Angle = 0.891
Prosit predicted
m/z
Fragment ion annotation using Prosit ions

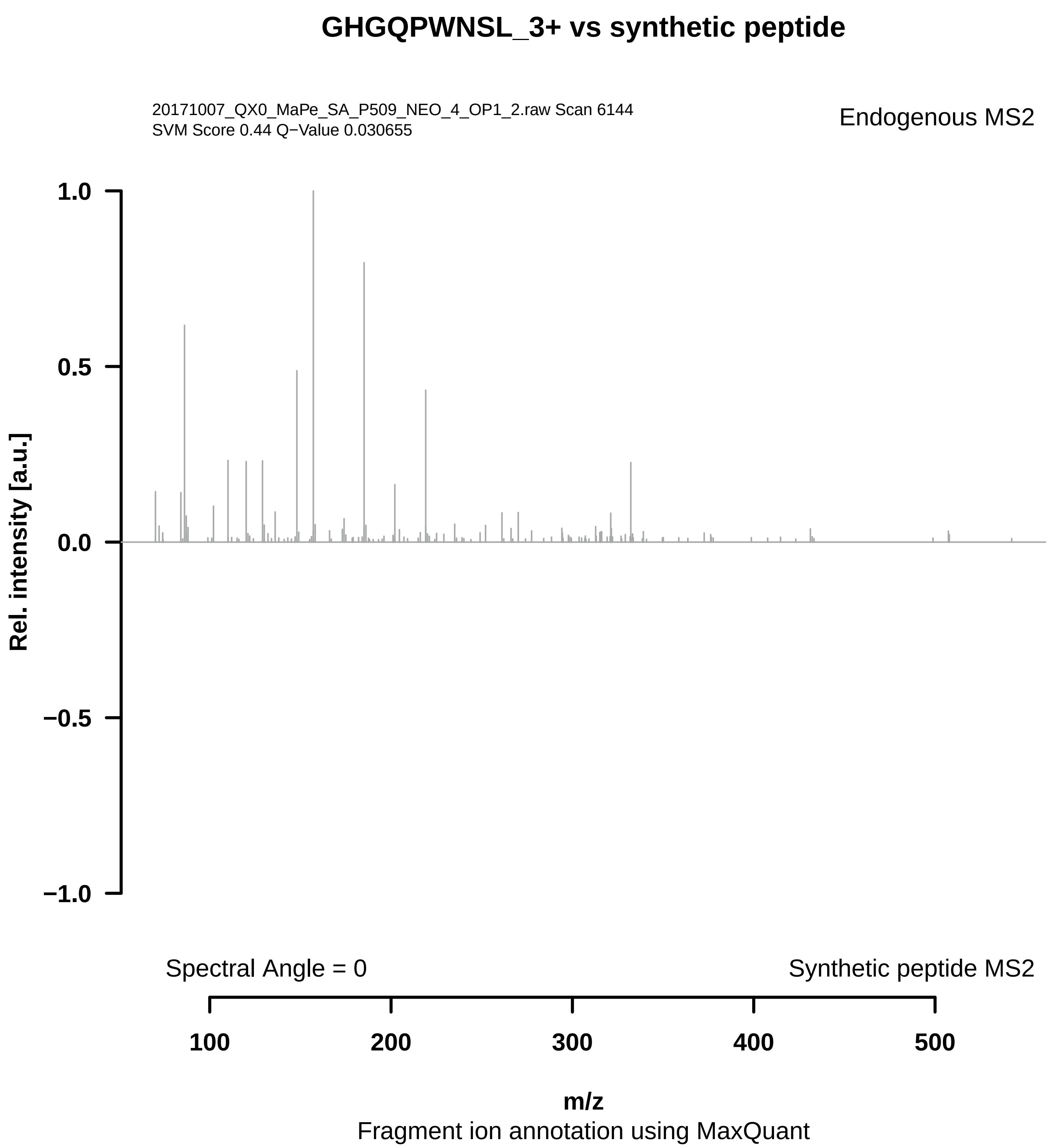
GHGQPWNSL_3+ vs synthetic peptide
20171007_QX0_MaPe_SA_P509_NEO_4_OP1_2.raw Scan 6144
SVM Score 0.44 Q−Value 0.030655
Endogenous MS2
1.0
0.5
0.0
−0.5
−1.0
Rel. intensity [a.u.]
Spectral Angle = 0
Synthetic peptide MS2
100
200
300
400
500
m/z
Fragment ion annotation using MaxQuant

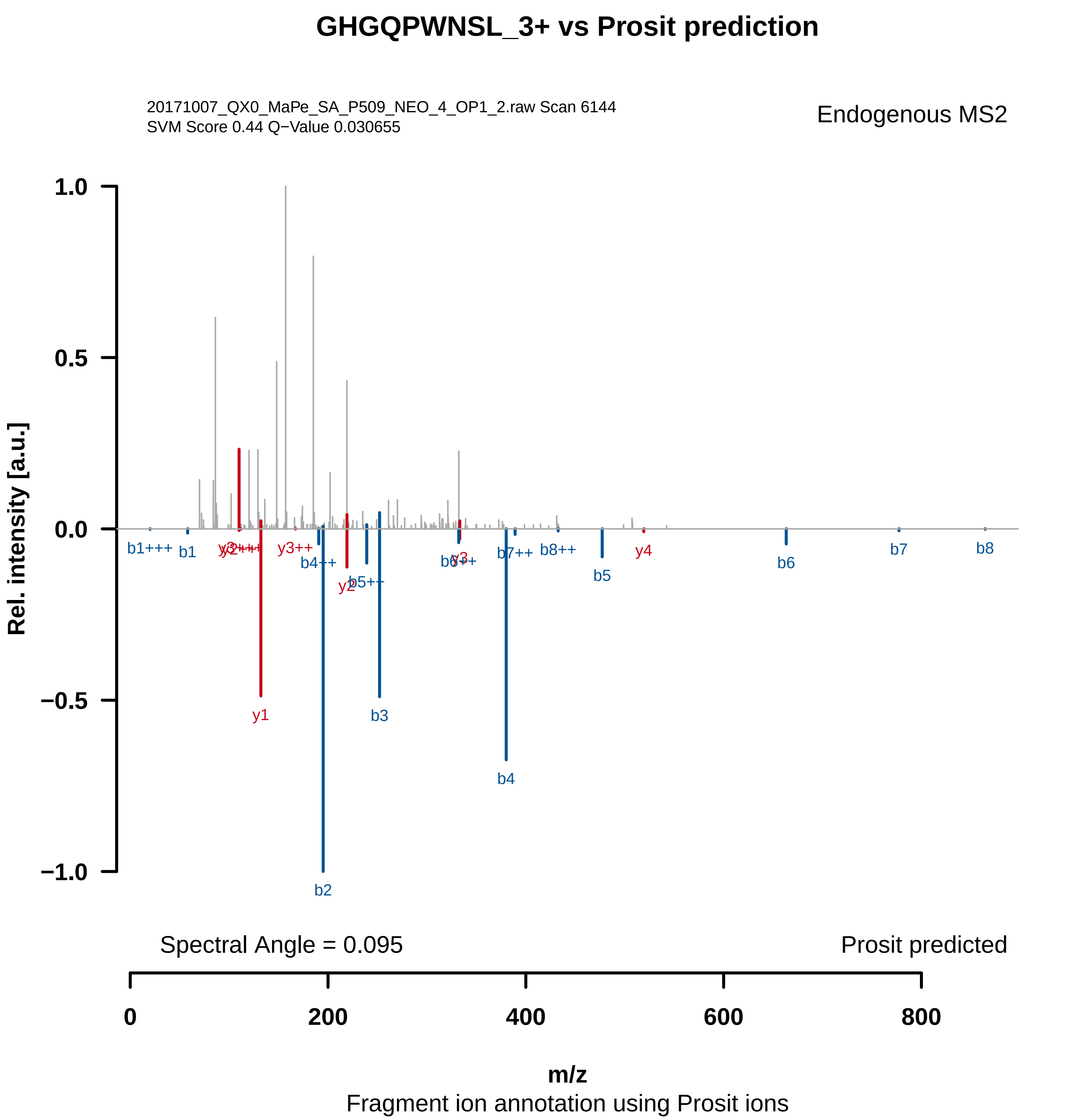
GHGQPWNSL_3+ vs Prosit prediction
20171007_QX0_MaPe_SA_P509_NEO_4_OP1_2.raw Scan 6144
SVM Score 0.44 Q−Value 0.030655
Endogenous MS2
1.0
0.5
0.0
−0.5
−1.0
Rel. intensity [a.u.]
b1+++
b1
y3++
b4++
y2
b5++
y1
b3
b2
b7++
b8++
b5
y4
b4
b6
b7
b8
Spectral Angle = 0.095
Prosit predicted
0
200
400
600
800
m/z
Fragment ion annotation using Prosit ions

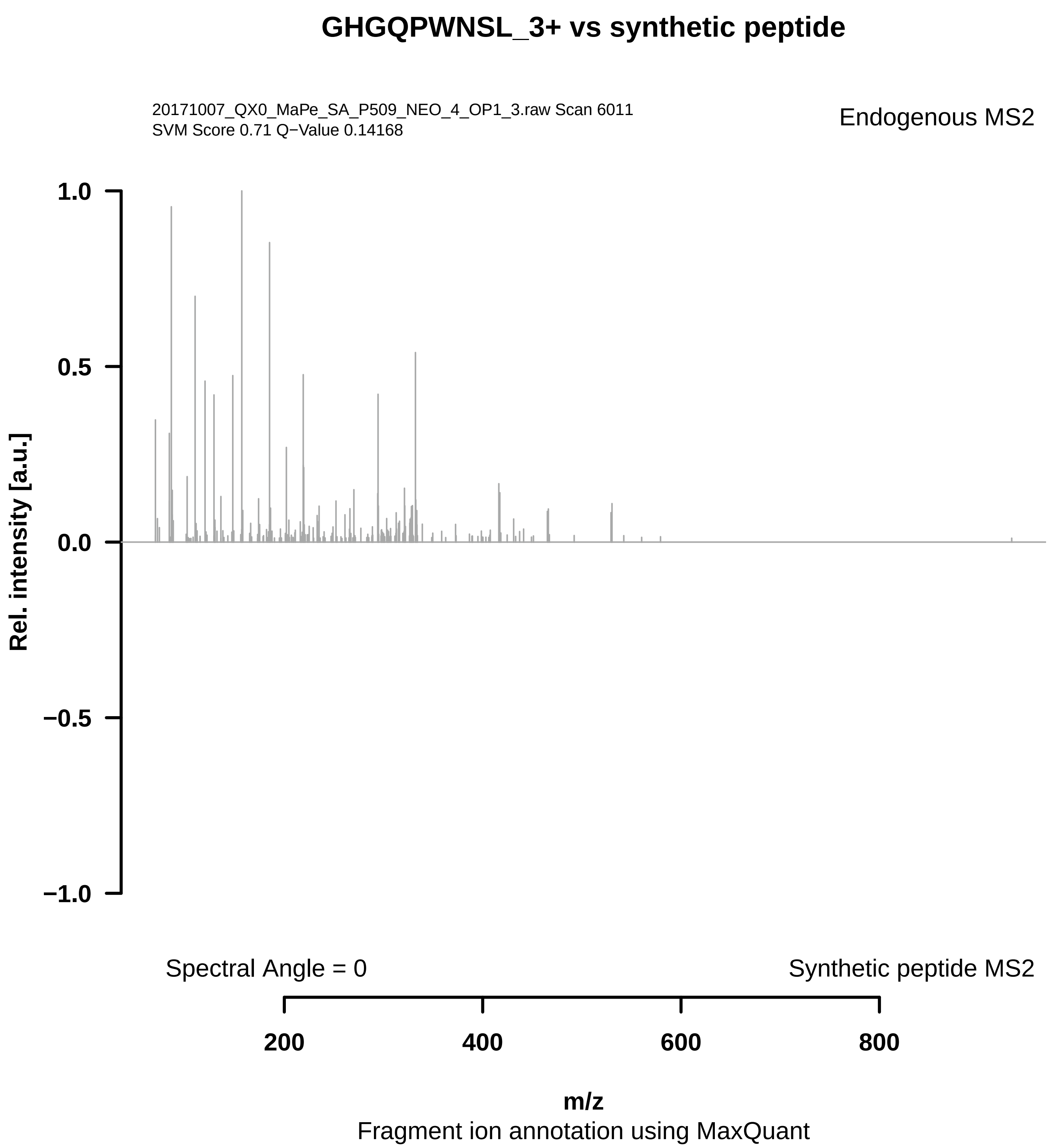

GHGQPWNSL_3+ vs synthetic peptide
20171007_QX0_MaPe_SA_P509_NEO_4_OP1_3.raw Scan 6011
SVM Score 0.71 Q−Value 0.14168
Endogenous MS2
Rel. intensity [a.u.]
Spectral Angle = 0
Synthetic peptide MS2
m/z
Fragment ion annotation using MaxQuant

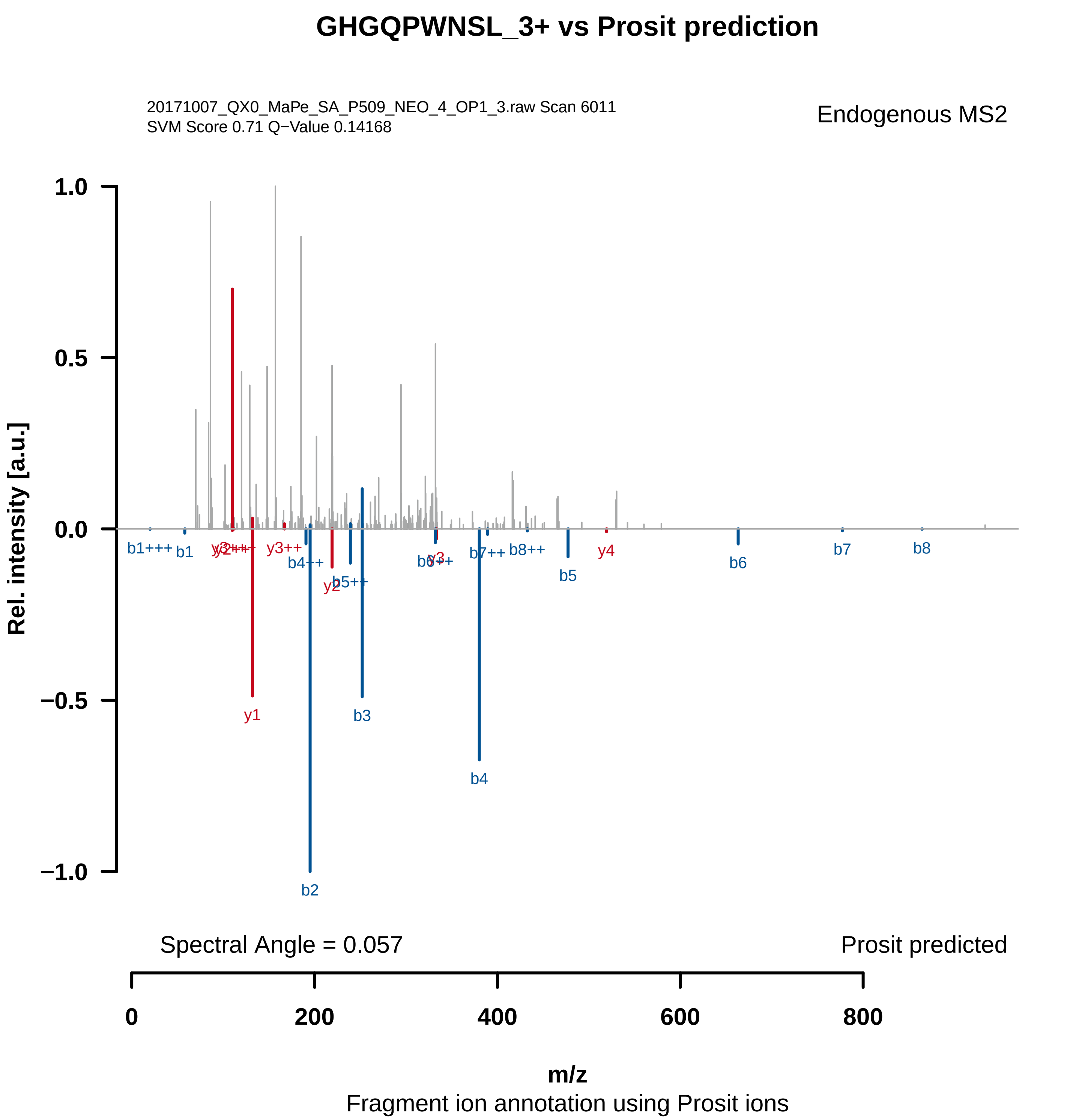

GHGQPWNSL_3+ vs Prosit prediction
20171007_QX0_MaPe_SA_P509_NEO_4_OP1_3.raw Scan 6011
SVM Score 0.71 Q−Value 0.14168
Endogenous MS2
Rel. intensity [a.u.]
Spectral Angle = 0.057
Prosit predicted
m/z
Fragment ion annotation using Prosit ions

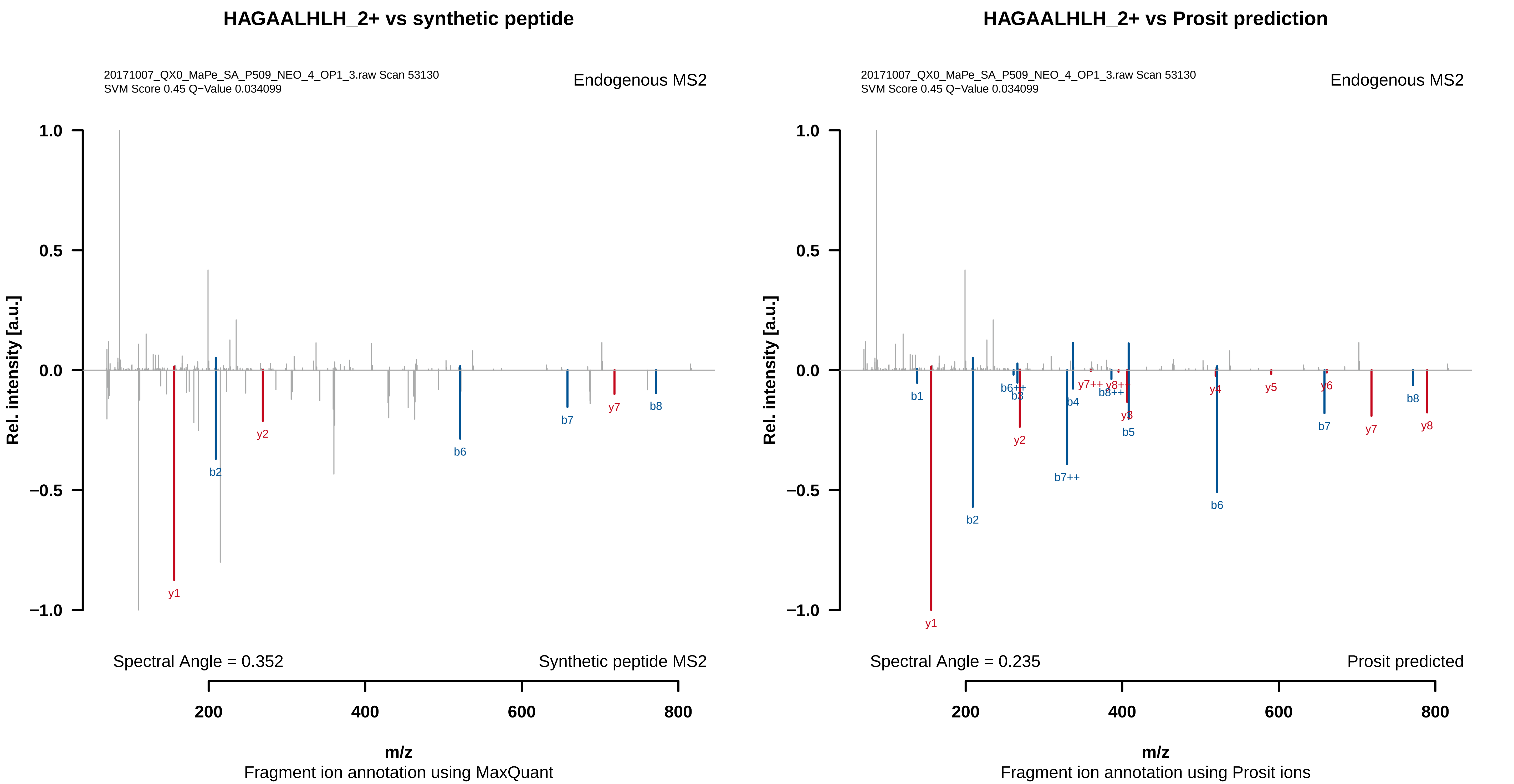
HAGAALHLH_2+ vs synthetic peptide
20171007_QX0_MaPe_SA_P509_NEO_4_OP1_3.raw Scan 53130
SVM Score 0.45 Q−Value 0.034099
Endogenous MS2
Rel. intensity [a.u.]
1.0
0.5
0.0
−0.5
−1.0
y1
b2
y2
b6
b7
y7
b8
Spectral Angle = 0.352
Synthetic peptide MS2
200
400
600
800
m/z
Fragment ion annotation using MaxQuant
HAGAALHLH_2+ vs Prosit prediction
20171007_QX0_MaPe_SA_P509_NEO_4_OP1_3.raw Scan 53130
SVM Score 0.45 Q−Value 0.034099
Endogenous MS2
Rel. intensity [a.u.]
b1
y1
b2
b6++
b3
y2
b7++
b4
y7++
y8++
b8++
y3
b5
y4
b6
y5
y6
b7
y7
b8
y8
Spectral Angle = 0.235
Prosit predicted
m/z
Fragment ion annotation using Prosit ions

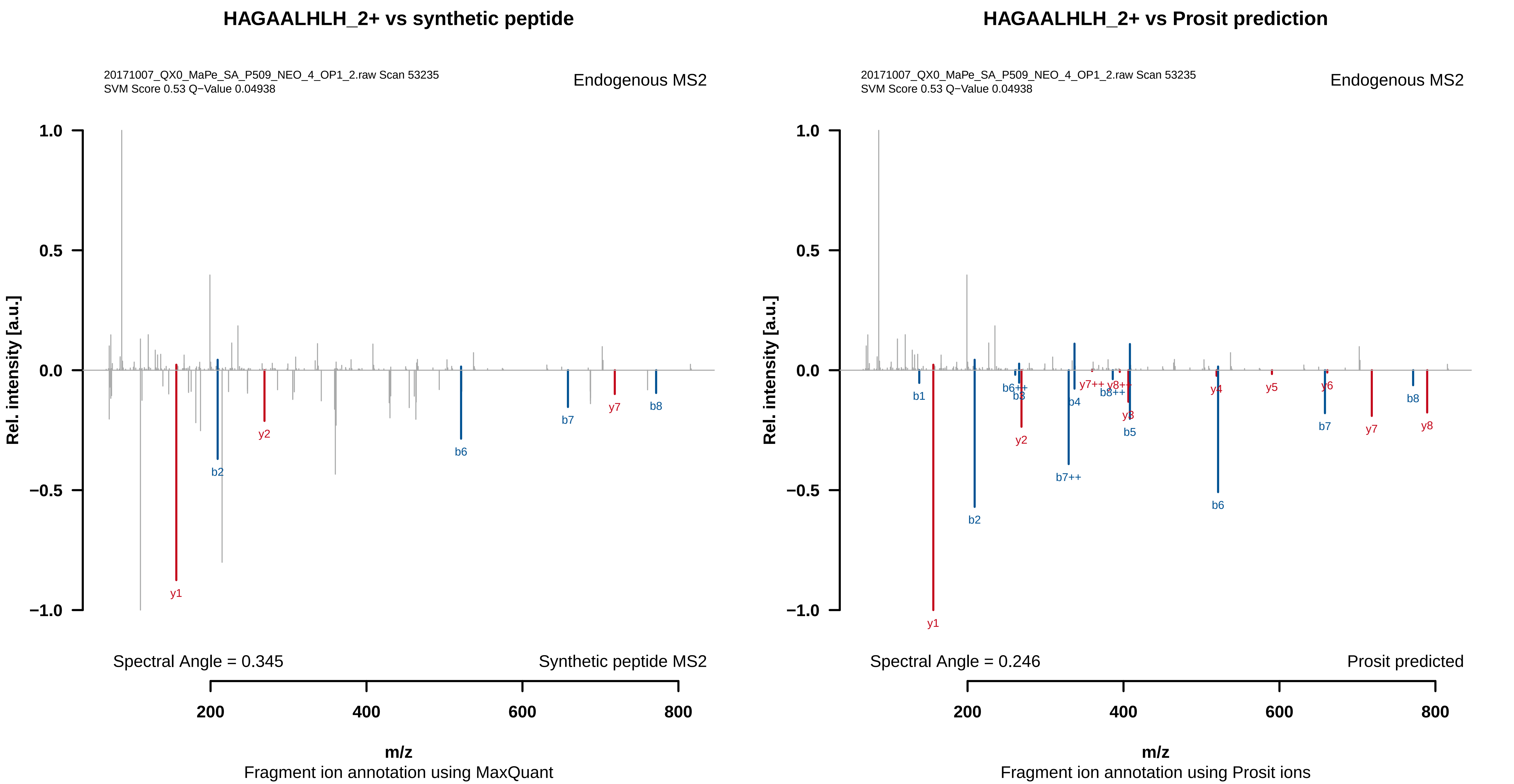
HAGAALHLH_2+ vs synthetic peptide
20171007_QX0_MaPe_SA_P509_NEO_4_OP1_2.raw Scan 53235
SVM Score 0.53 Q−Value 0.04938
Endogenous MS2
Rel. intensity [a.u.]
1.0
0.5
0.0
−0.5
−1.0
y1
b2
y2
b6
b7
y7
b8
Spectral Angle = 0.345
Synthetic peptide MS2
200
400
600
800
m/z
Fragment ion annotation using MaxQuant
HAGAALHLH_2+ vs Prosit prediction
20171007_QX0_MaPe_SA_P509_NEO_4_OP1_2.raw Scan 53235
SVM Score 0.53 Q−Value 0.04938
Endogenous MS2
Rel. intensity [a.u.]
1.0
0.5
0.0
−0.5
−1.0
b1
y1
b2
b6++
b3
y2
b7++
b4
y7++
y8++
b8++
y3
b5
y4
b6
y5
y6
b7
y7
b8
y8
Spectral Angle = 0.246
Prosit predicted
200
400
600
800
m/z
Fragment ion annotation using Prosit ions

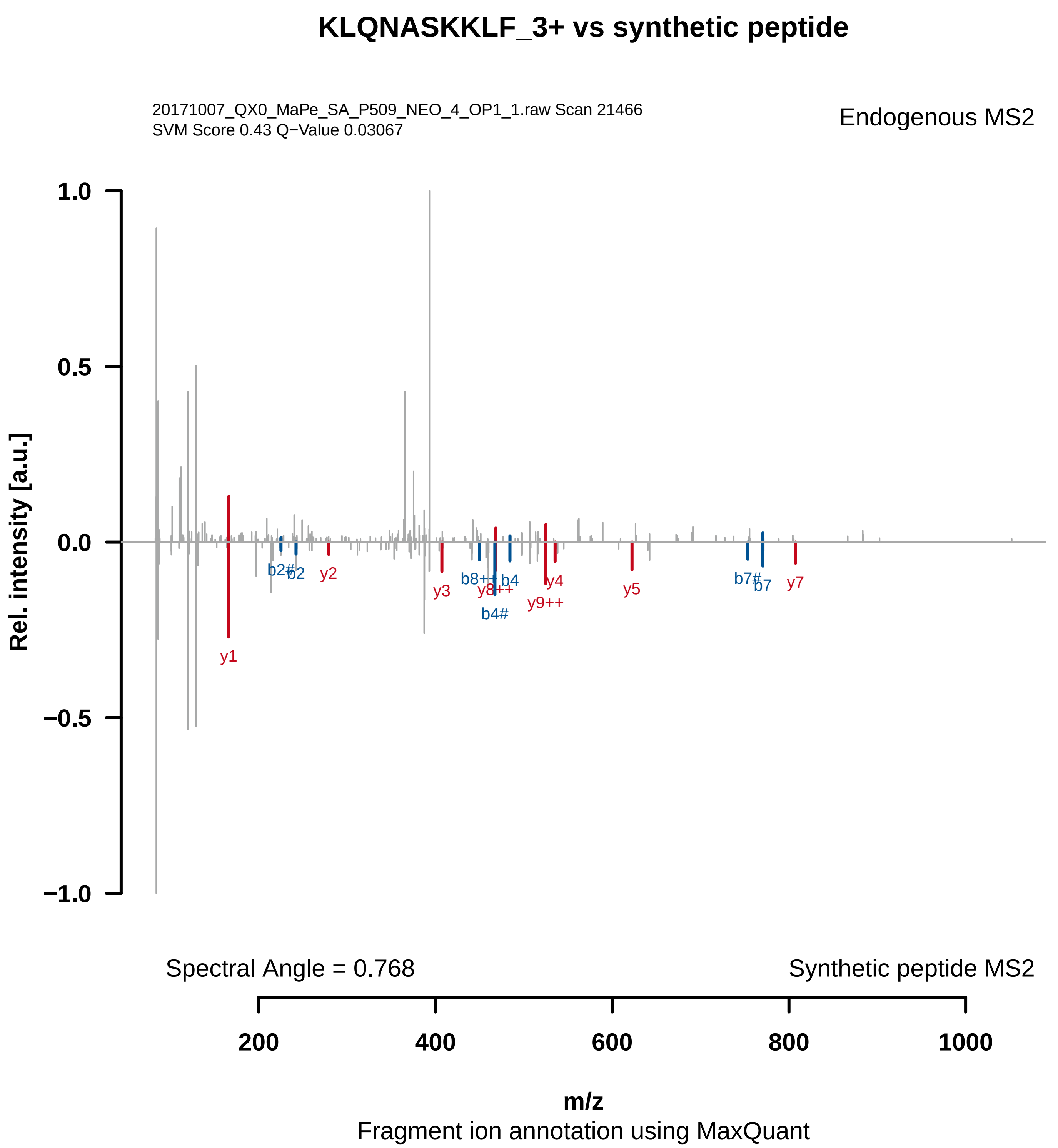
KLQNASKKLF_3+ vs synthetic peptide
20171007_QX0_MaPe_SA_P509_NEO_4_OP1_1.raw Scan 21466
SVM Score 0.43 Q−Value 0.03067
Endogenous MS2
Rel. intensity [a.u.]
Spectral Angle = 0.768
Synthetic peptide MS2
m/z
Fragment ion annotation using MaxQuant

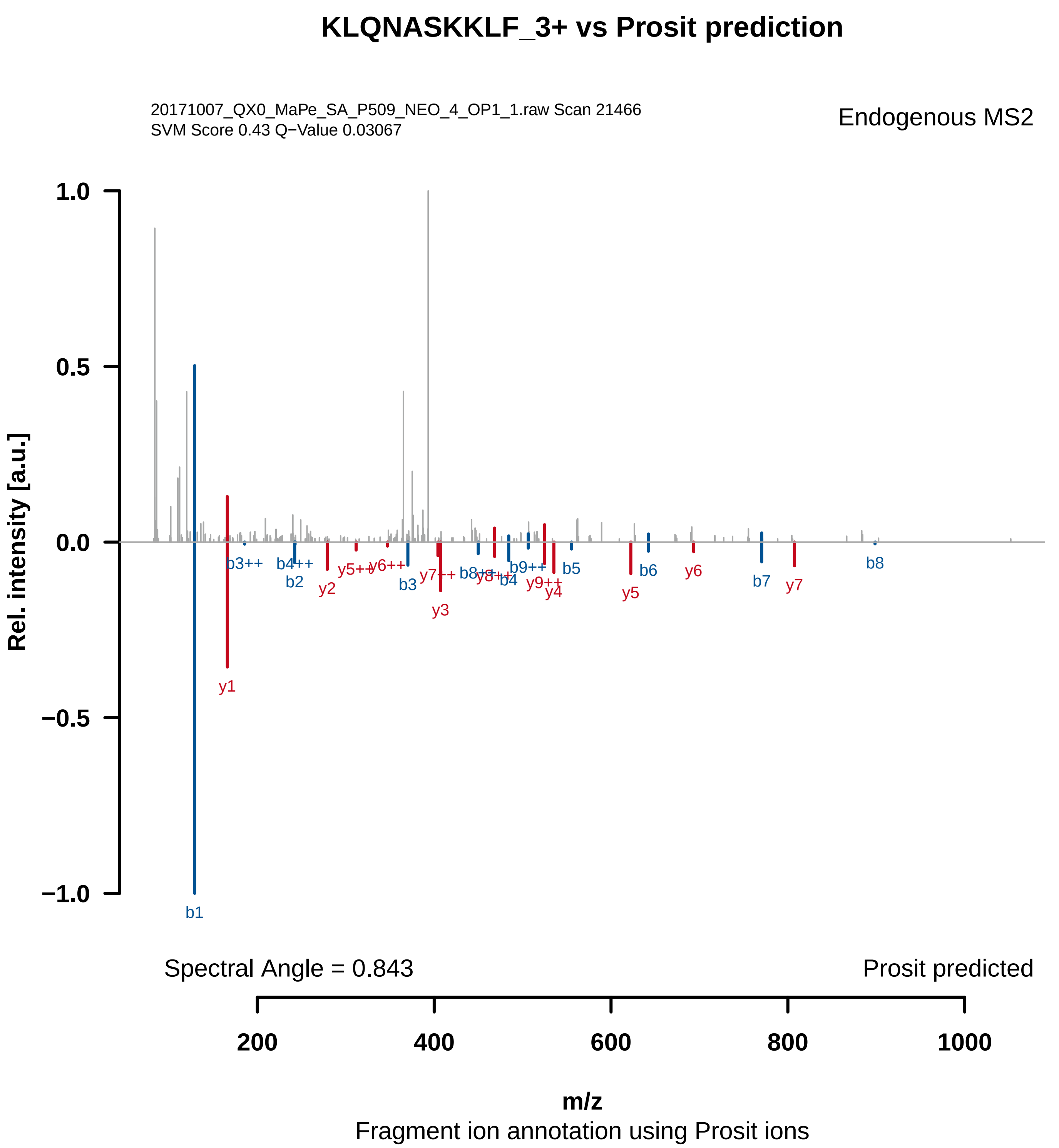
KLQNASKKLF_3+ vs Prosit prediction
20171007_QX0_MaPe_SA_P509_NEO_4_OP1_1.raw Scan 21466
SVM Score 0.43 Q−Value 0.03067
Endogenous MS2
Rel. intensity [a.u.]
Spectral Angle = 0.843
Prosit predicted
m/z
Fragment ion annotation using Prosit ions

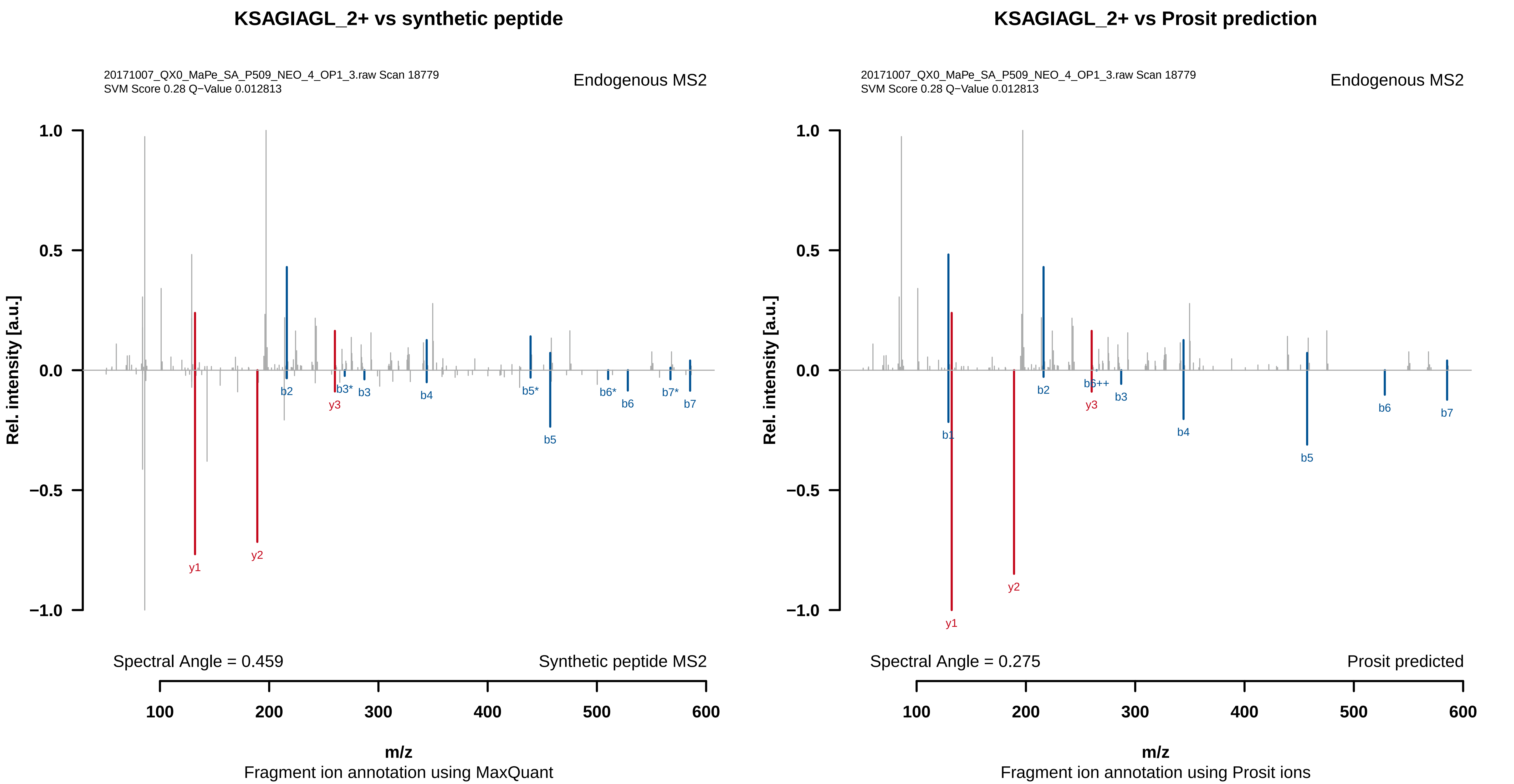
KSAGIAGL_2+ vs synthetic peptide
20171007_QX0_MaPe_SA_P509_NEO_4_OP1_3.raw Scan 18779
SVM Score 0.28 Q−Value 0.012813
Endogenous MS2
Rel. intensity [a.u.]
1.0
0.5
0.0
−0.5
−1.0
b2
b3*
b3
y3
b4
b5*
b5
b6*
b6
b7*
b7
y1
y2
Spectral Angle = 0.459
Synthetic peptide MS2
100
200
300
400
500
600
m/z
Fragment ion annotation using MaxQuant
KSAGIAGL_2+ vs Prosit prediction
20171007_QX0_MaPe_SA_P509_NEO_4_OP1_3.raw Scan 18779
SVM Score 0.28 Q−Value 0.012813
Endogenous MS2
Rel. intensity [a.u.]
1.0
0.5
0.0
−0.5
−1.0
b1
b2
b6++
y3
b3
b4
b5
b6
b7
y1
y2
Spectral Angle = 0.275
Prosit predicted
100
200
300
400
500
600
m/z
Fragment ion annotation using Prosit ions

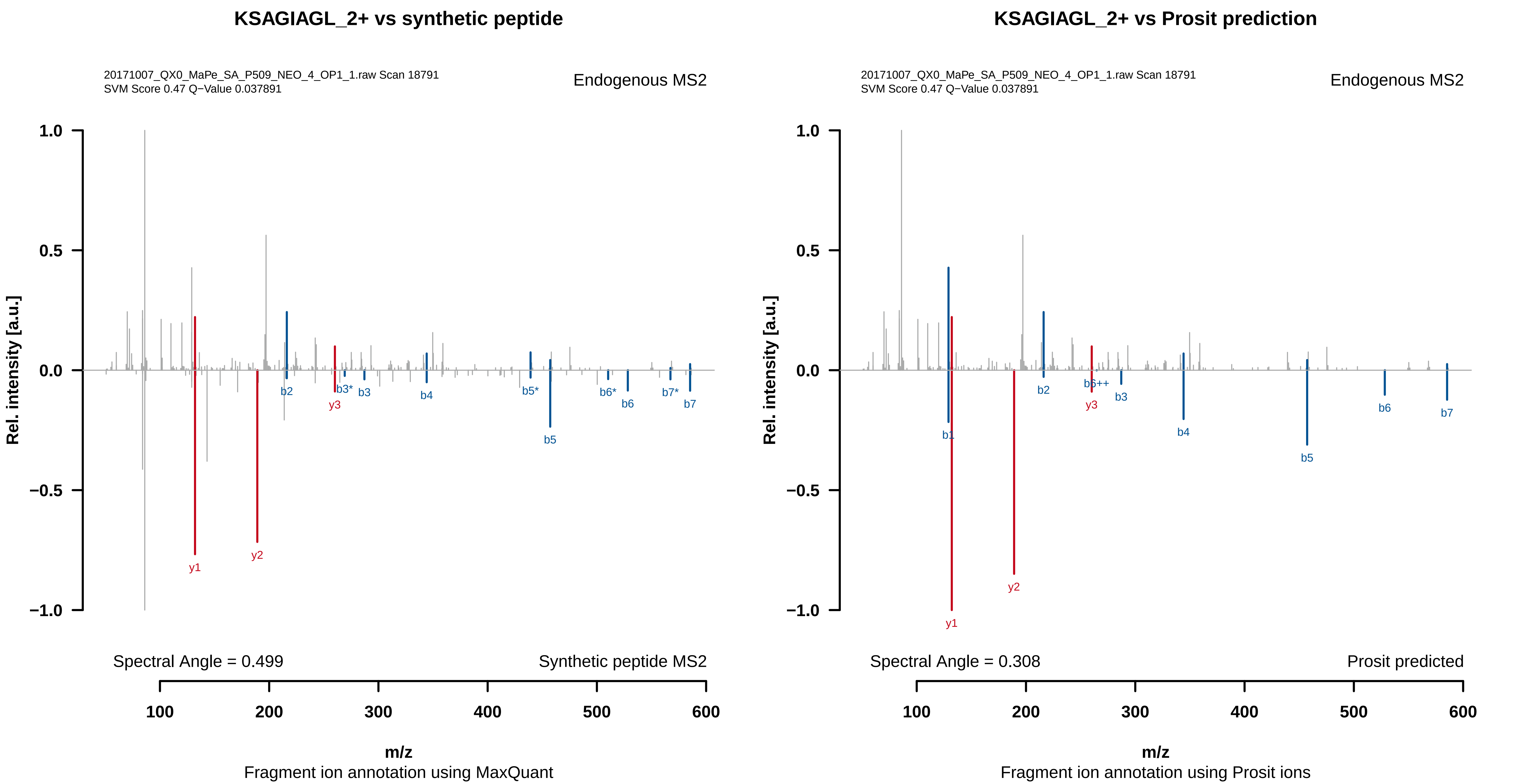

KSAGIAGL_2+ vs synthetic peptide
20171007_QX0_MaPe_SA_P509_NEO_4_OP1_1.raw Scan 18791
SVM Score 0.47 Q−Value 0.037891
Endogenous MS2
Rel. intensity [a.u.]
1.0
0.5
0.0
−0.5
−1.0
b2
b3*
b3
b4
b5*
b5
b6*
b6
b7*
b7
y1
y2
y3
Spectral Angle = 0.499
Synthetic peptide MS2
100
200
300
400
500
600
m/z
Fragment ion annotation using MaxQuant
KSAGIAGL_2+ vs Prosit prediction
20171007_QX0_MaPe_SA_P509_NEO_4_OP1_1.raw Scan 18791
SVM Score 0.47 Q−Value 0.037891
Endogenous MS2
Rel. intensity [a.u.]
1.0
0.5
0.0
−0.5
−1.0
b1
b2
b6++
b3
b4
b5
b6
b7
y1
y2
y3
Spectral Angle = 0.308
Prosit predicted
100
200
300
400
500
600
m/z
Fragment ion annotation using Prosit ions

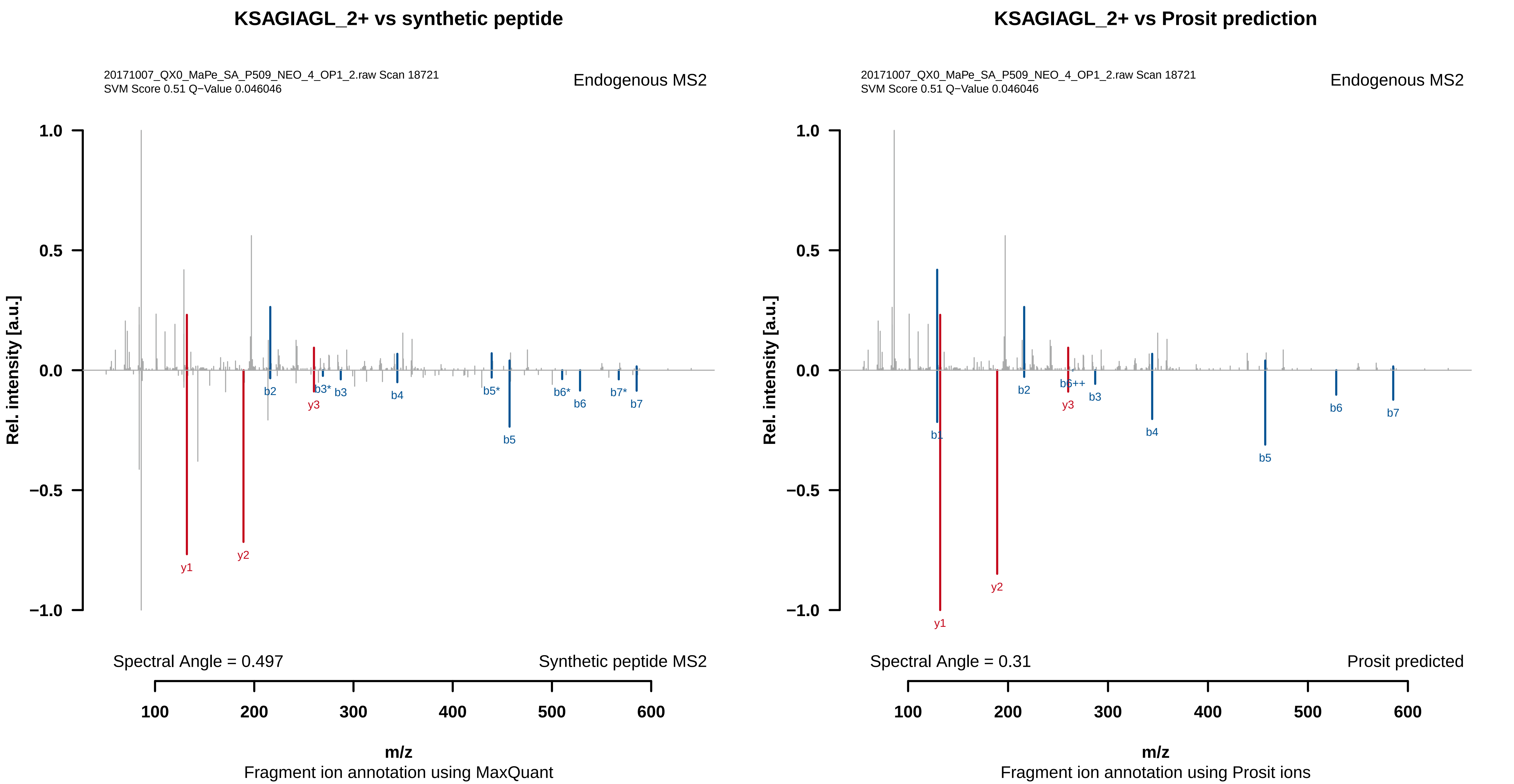
KSAGIAGL_2+ vs synthetic peptide
20171007_QX0_MaPe_SA_P509_NEO_4_OP1_2.raw Scan 18721
SVM Score 0.51 Q−Value 0.046046
Endogenous MS2
Rel. intensity [a.u.]
Spectral Angle = 0.497
Synthetic peptide MS2
m/z
Fragment ion annotation using MaxQuant
KSAGIAGL_2+ vs Prosit prediction
20171007_QX0_MaPe_SA_P509_NEO_4_OP1_2.raw Scan 18721
SVM Score 0.51 Q−Value 0.046046
Endogenous MS2
Rel. intensity [a.u.]
Spectral Angle = 0.31
Prosit predicted
m/z
Fragment ion annotation using Prosit ions

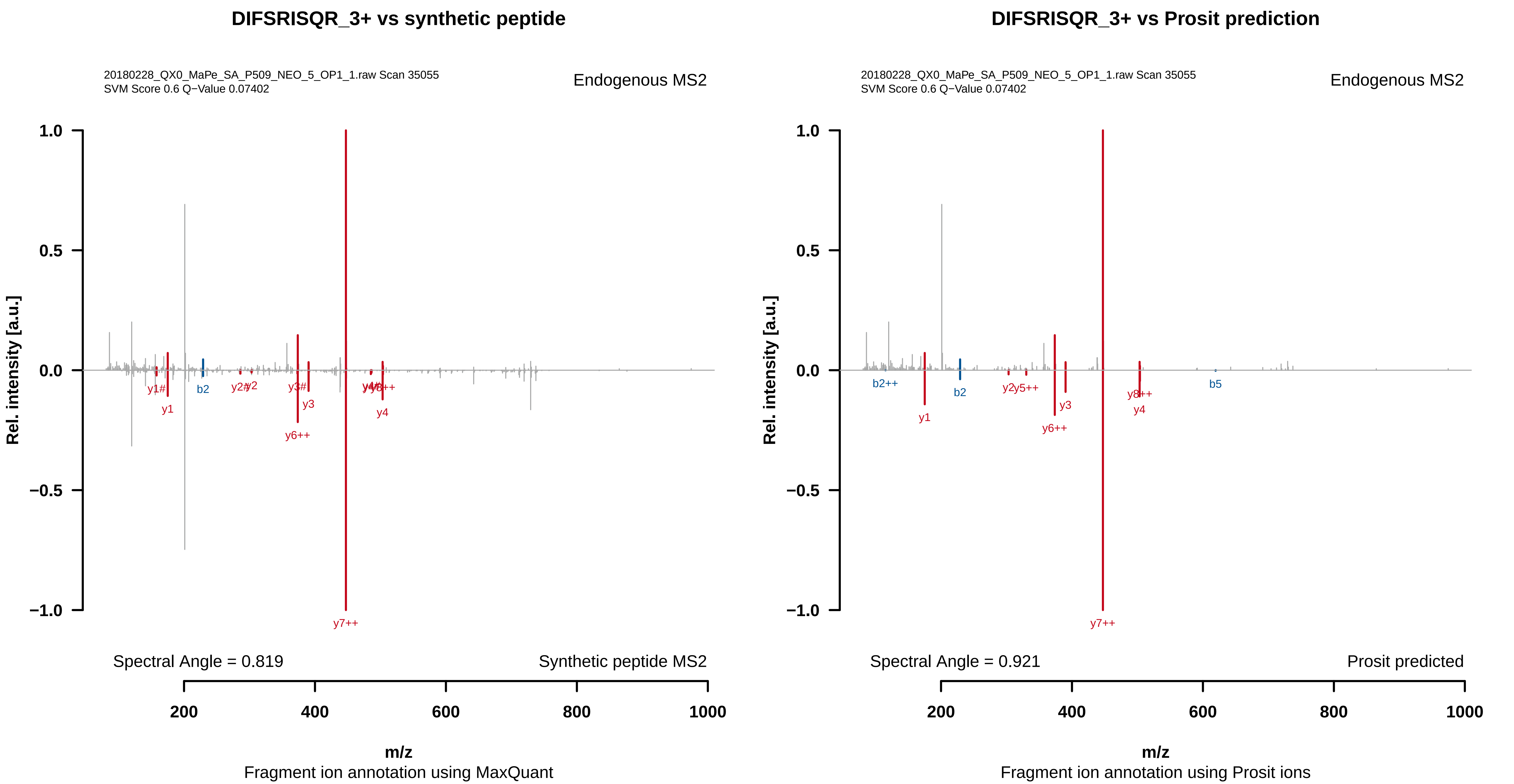

DIFSRISQR_3+ vs synthetic peptide
20180228_QX0_MaPe_SA_P509_NEO_5_OP1_1.raw Scan 35055
SVM Score 0.6 Q−Value 0.07402
Endogenous MS2
Rel. intensity [a.u.]
1.0
0.5
0.0
−0.5
−1.0
y1#
y1
b2
y2#
y2
y3#
y3
y6++
y7++
y4#
y8++
y4
Spectral Angle = 0.819
Synthetic peptide MS2
200
400
600
800
1000
m/z
Fragment ion annotation using MaxQuant
DIFSRISQR_3+ vs Prosit prediction
20180228_QX0_MaPe_SA_P509_NEO_5_OP1_1.raw Scan 35055
SVM Score 0.6 Q−Value 0.07402
Endogenous MS2
Rel. intensity [a.u.]
1.0
0.5
0.0
−0.5
−1.0
b2++
y1
b2
y2
y5++
y3
y6++
y7++
y8++
y4
b5
Spectral Angle = 0.921
Prosit predicted
200
400
600
800
1000
m/z
Fragment ion annotation using Prosit ions

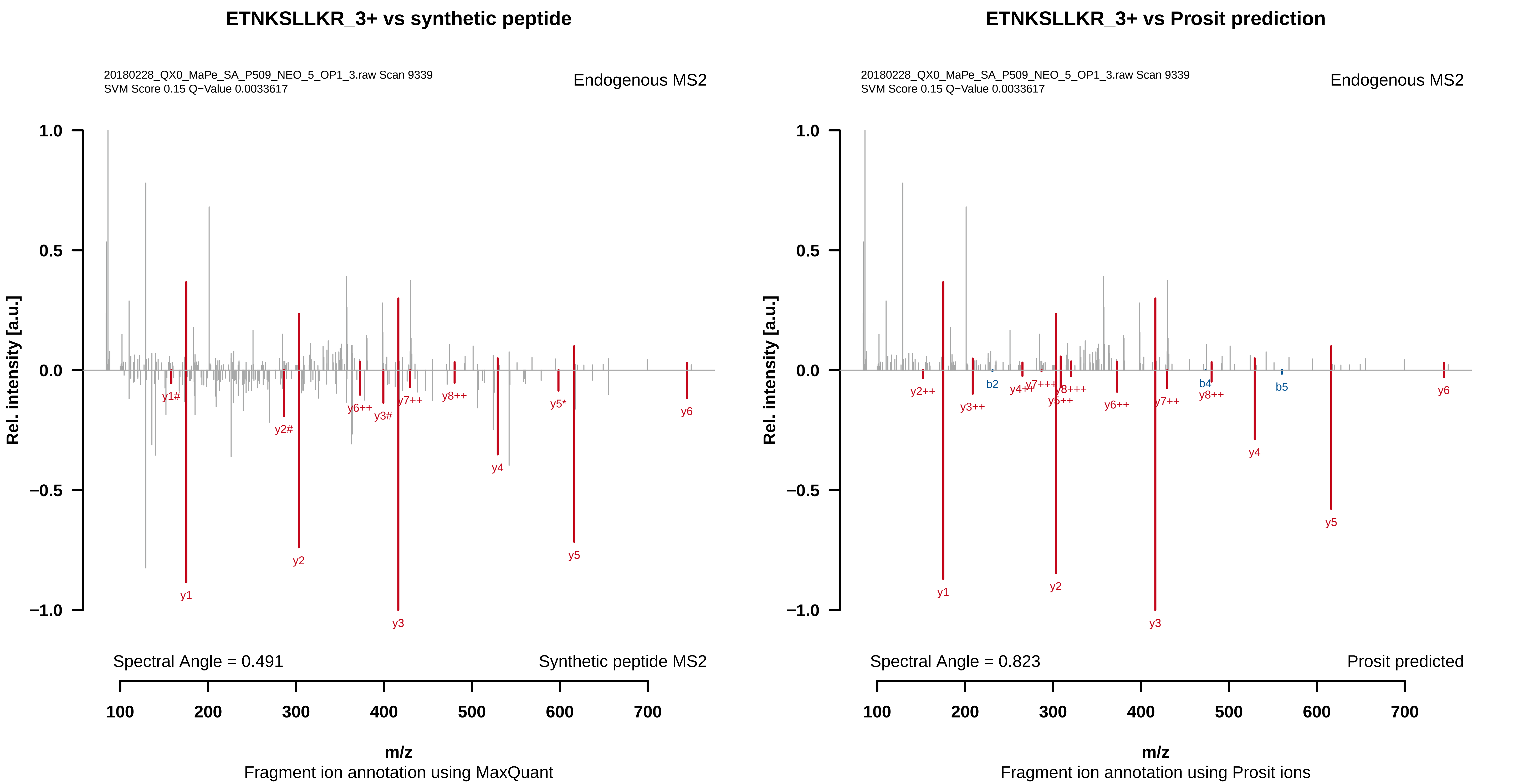
ETNKSLLKR_3+ vs synthetic peptide
20180228_QX0_MaPe_SA_P509_NEO_5_OP1_3.raw Scan 9339
SVM Score 0.15 Q−Value 0.0033617
Endogenous MS2
Rel. intensity [a.u.]
1.0
0.5
0.0
−0.5
−1.0
y1#
y1
y2#
y2
y6++
y3#
y3
y7++
y8++
y4
y5*
y5
y6
Spectral Angle = 0.491
Synthetic peptide MS2
100
200
300
400
500
600
700
m/z
Fragment ion annotation using MaxQuant
ETNKSLLKR_3+ vs Prosit prediction
20180228_QX0_MaPe_SA_P509_NEO_5_OP1_3.raw Scan 9339
SVM Score 0.15 Q−Value 0.0033617
Endogenous MS2
Rel. intensity [a.u.]
1.0
0.5
0.0
−0.5
−1.0
y2++
y1
y3++
b2
y4++
y7+++
y8+++
y5++
y2
y6++
y7++
y3
b4
y8++
y4
b5
y5
y6
Spectral Angle = 0.823
Prosit predicted
100
200
300
400
500
600
700
m/z
Fragment ion annotation using Prosit ions

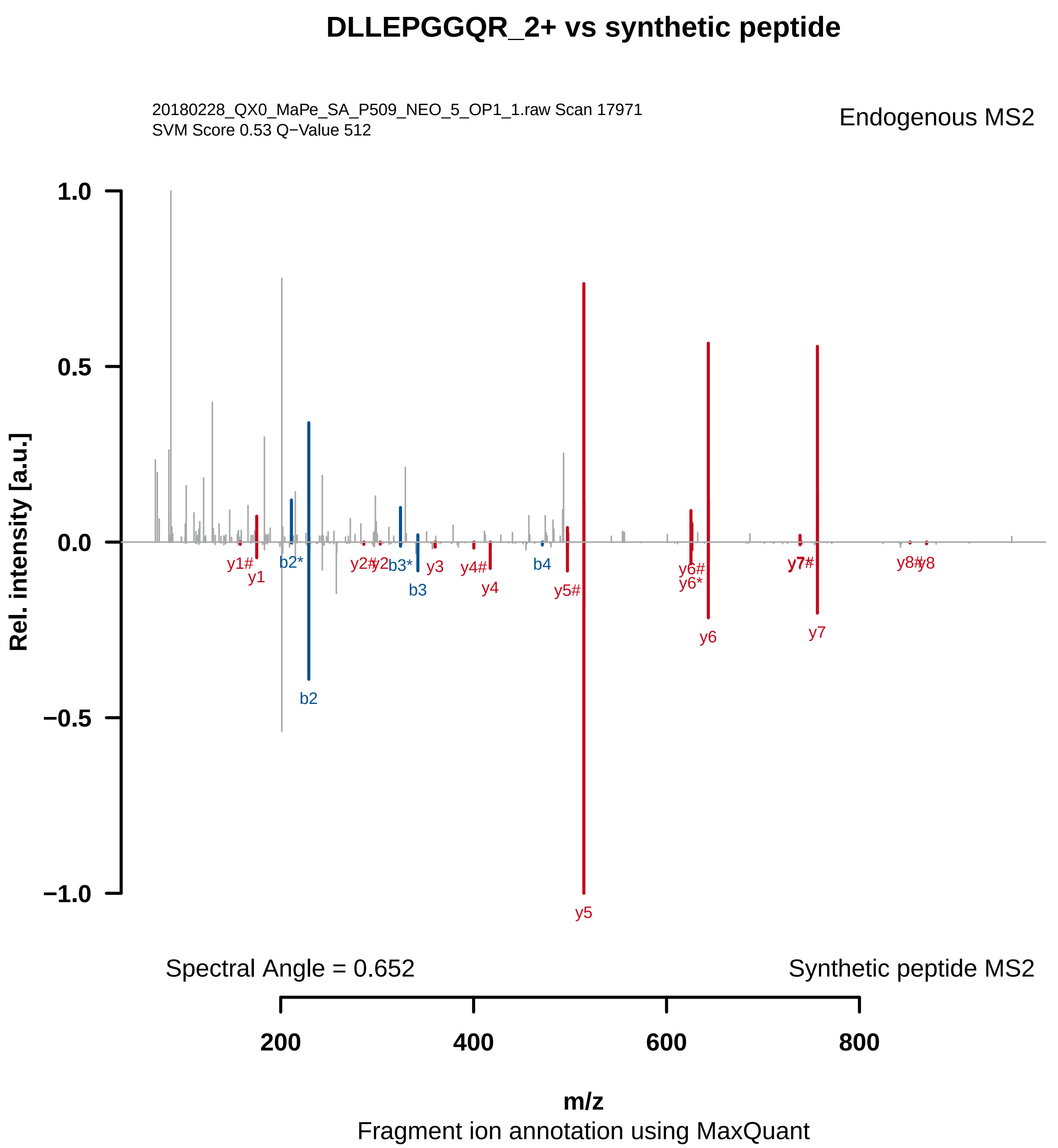

DLLEPGGQR_2+ vs synthetic peptide
20180228_QX0_MaPe_SA_P509_NEO_5_OP1_1.raw Scan 17971
SVM Score 0.53 Q−Value 512
Endogenous MS2
Rel. intensity [a.u.]
1.0
0.5
0.0
−0.5
−1.0
y1#
y1
b2*
b2
y2#
y2
b3*
b3
y3
y4#
y4
b4
y5#
y5
y6#
y6*
y6
y7#
y7
y8#
y8
Spectral Angle = 0.652
Synthetic peptide MS2
200
400
600
800
m/z
Fragment ion annotation using MaxQuant

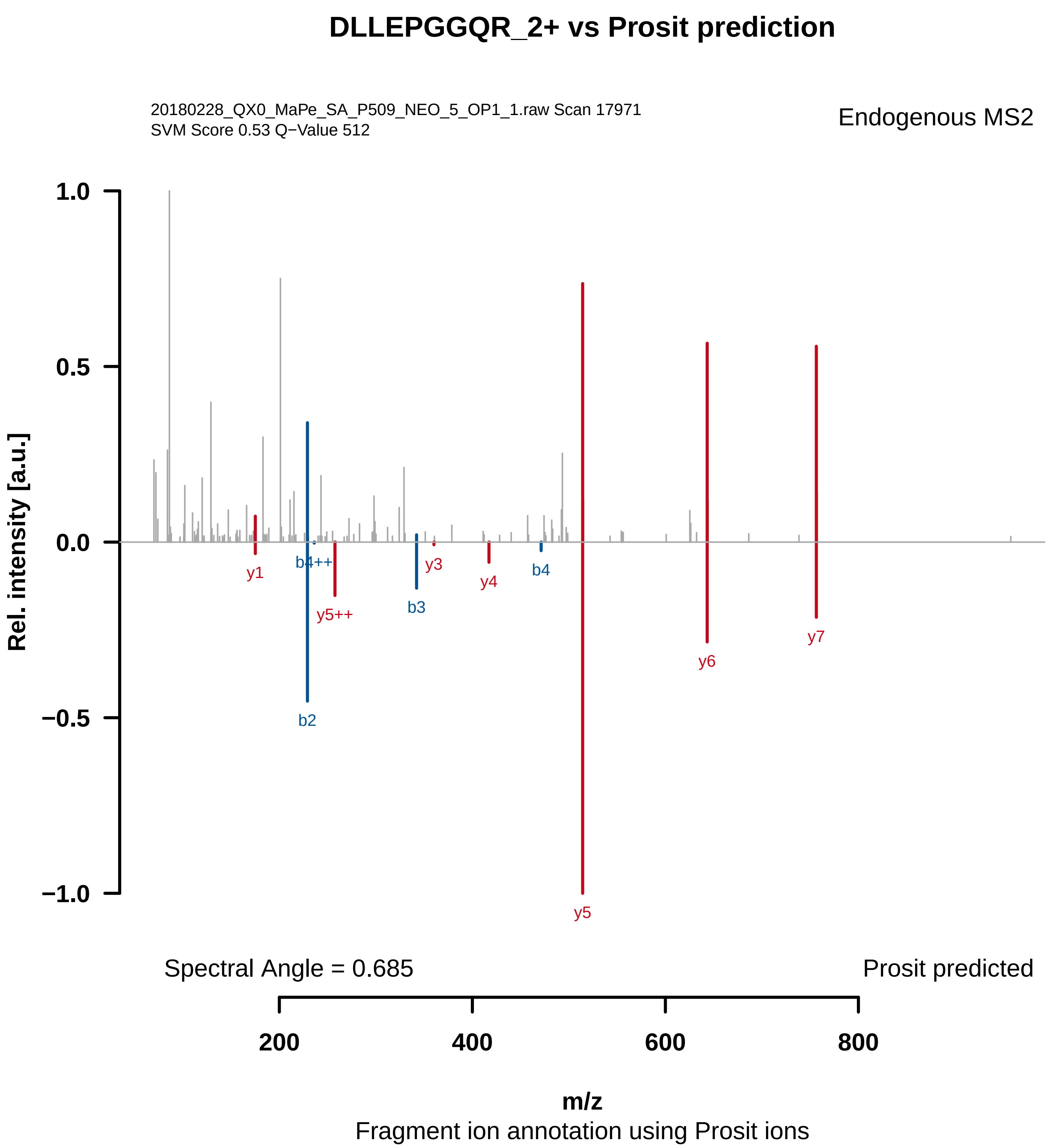

DLLEPGGQR_2+ vs Prosit prediction
20180228_QX0_MaPe_SA_P509_NEO_5_OP1_1.raw Scan 17971
SVM Score 0.53 Q−Value 512
Endogenous MS2
Rel. intensity [a.u.]
1.0
0.5
0.0
−0.5
−1.0
y1
b4++
b2
y5++
b3
y3
y4
b4
y5
y6
y7
Spectral Angle = 0.685
Prosit predicted
200
400
600
800
m/z
Fragment ion annotation using Prosit ions

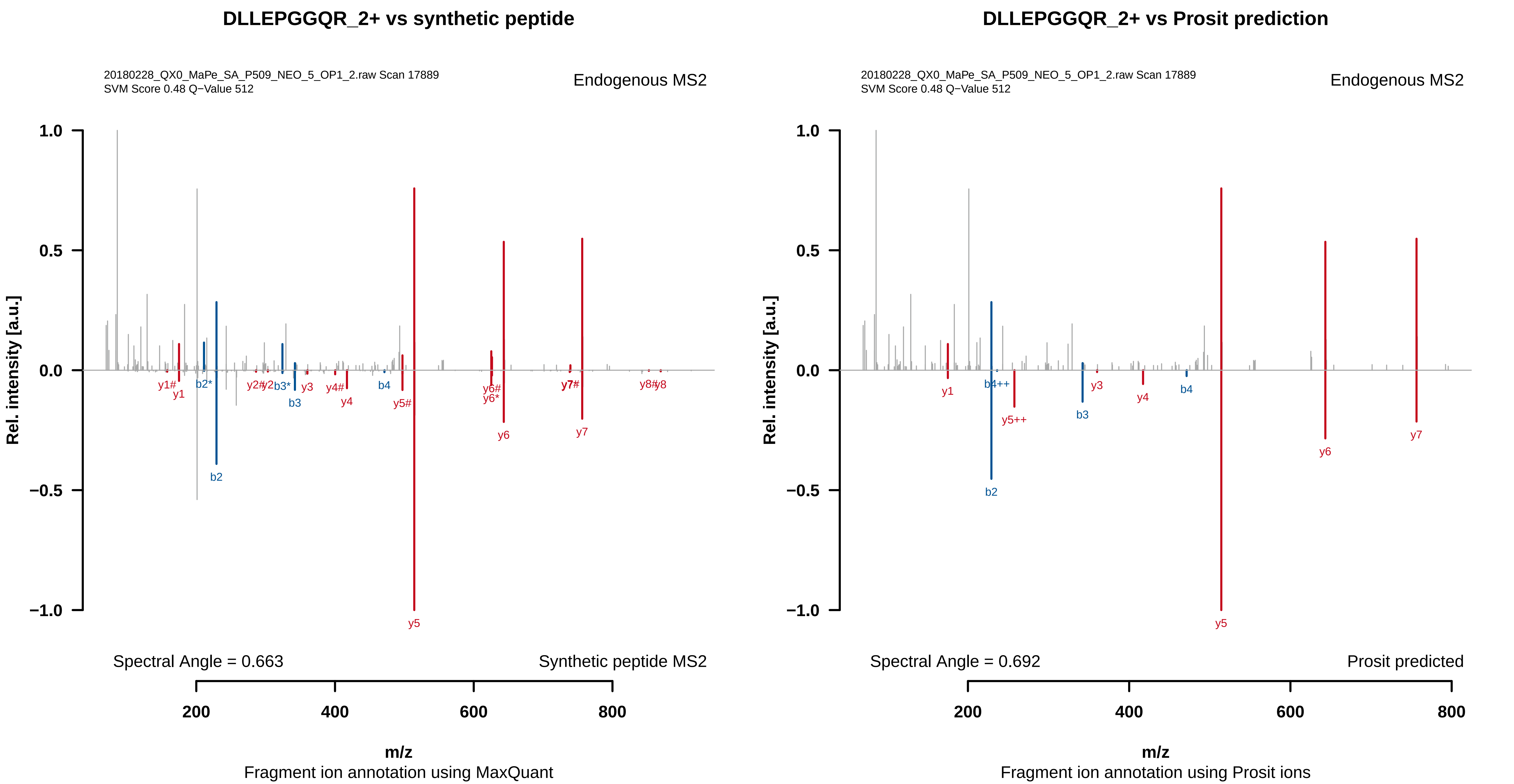

DLLEPGGQR_2+ vs synthetic peptide
20180228_QX0_MaPe_SA_P509_NEO_5_OP1_2.raw Scan 17889
SVM Score 0.48 Q−Value 512
Endogenous MS2
Rel. intensity [a.u.]
1.0
0.5
0.0
−0.5
−1.0
y1#
y1
b2*
b2
y2#
y2
b3*
b3
y3
y4#
y4
b4
y5#
y5
y6#
y6*
y6
y7#
y7
y8#
y8
Spectral Angle = 0.663
Synthetic peptide MS2
200
400
600
800
m/z
Fragment ion annotation using MaxQuant
DLLEPGGQR_2+ vs Prosit prediction
20180228_QX0_MaPe_SA_P509_NEO_5_OP1_2.raw Scan 17889
SVM Score 0.48 Q−Value 512
Endogenous MS2
Rel. intensity [a.u.]
1.0
0.5
0.0
−0.5
−1.0
y1
b4++
b2
y5++
b3
y3
y4
b4
y5
y6
y7
Spectral Angle = 0.692
Prosit predicted
200
400
600
800
m/z
Fragment ion annotation using Prosit ions

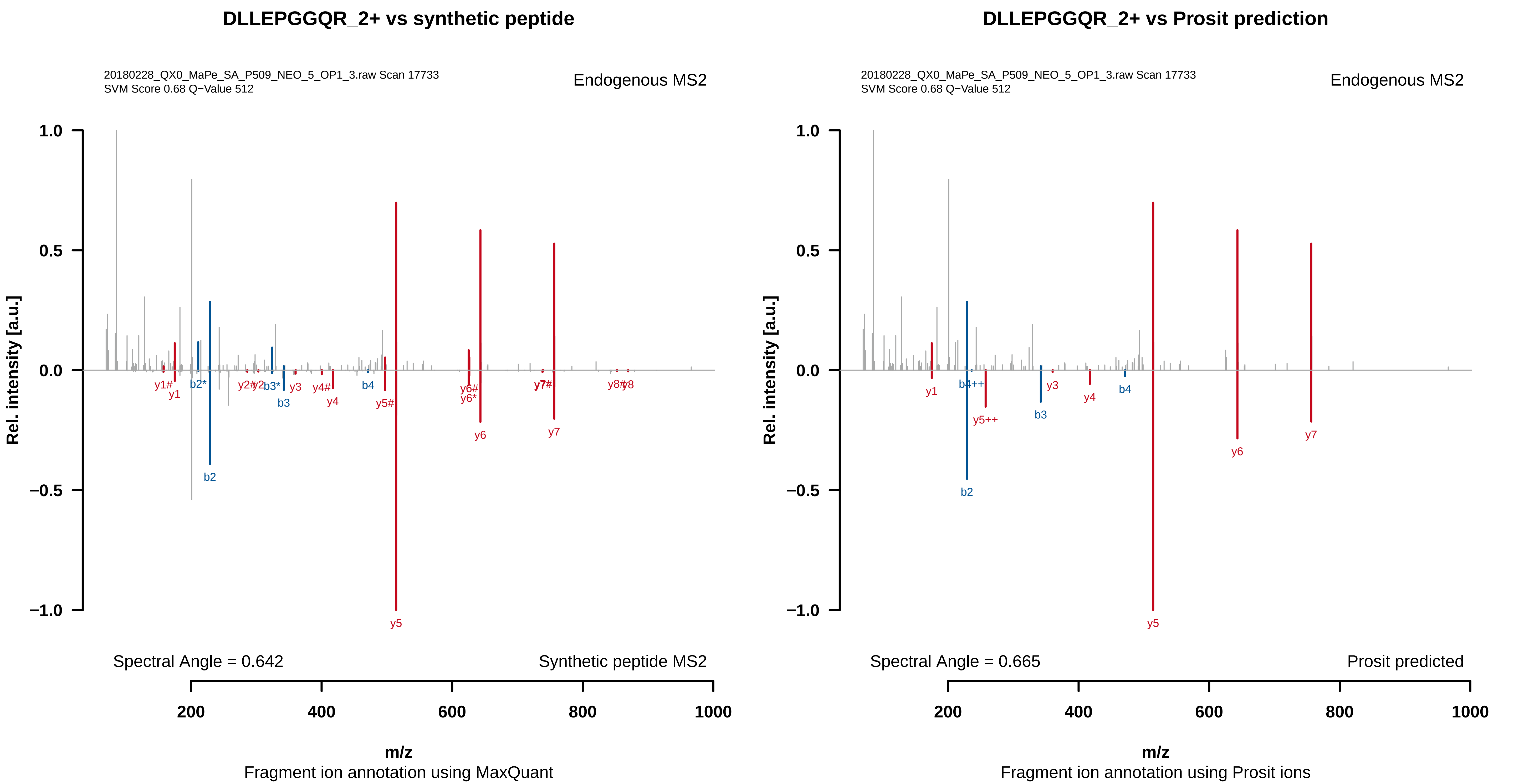

DLLEPGGQR_2+ vs synthetic peptide
20180228_QX0_MaPe_SA_P509_NEO_5_OP1_3.raw Scan 17733
SVM Score 0.68 Q−Value 512
Endogenous MS2
Rel. intensity [a.u.]
1.0
0.5
0.0
−0.5
−1.0
y1#
y1
b2*
b2
y2#
y2
b3*
b3
y3
y4#
y4
b4
y5#
y5
y6#
y6*
y6
y7#
y7
y8#
y8
Spectral Angle = 0.642
Synthetic peptide MS2
200
400
600
800
1000
m/z
Fragment ion annotation using MaxQuant
DLLEPGGQR_2+ vs Prosit prediction
20180228_QX0_MaPe_SA_P509_NEO_5_OP1_3.raw Scan 17733
SVM Score 0.68 Q−Value 512
Endogenous MS2
Rel. intensity [a.u.]
1.0
0.5
0.0
−0.5
−1.0
y1
b4++
b2
y5++
b3
y3
y4
b4
y5
y6
y7
Spectral Angle = 0.665
Prosit predicted
200
400
600
800
1000
m/z
Fragment ion annotation using Prosit ions

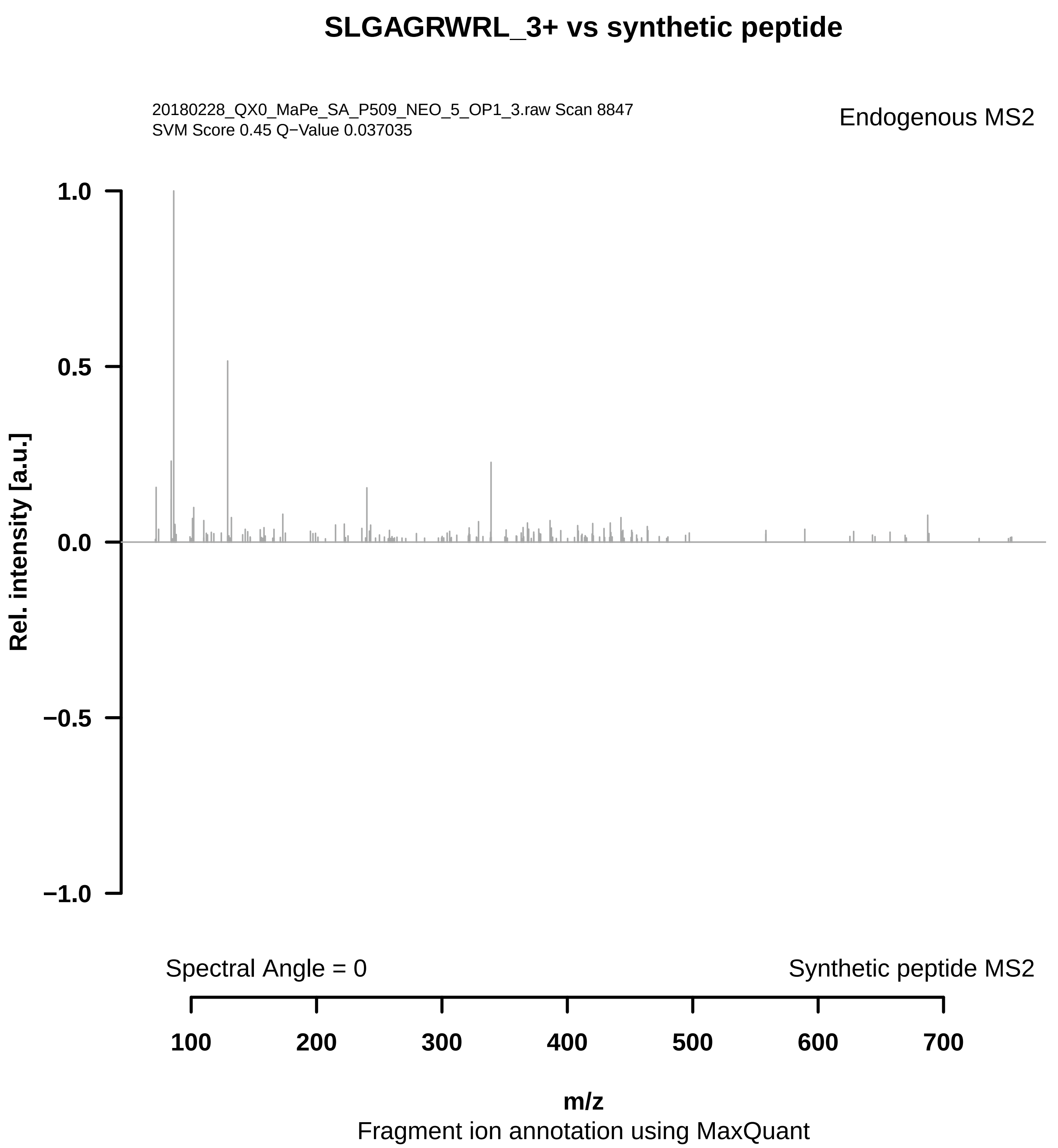
SLGAGRWRL_3+ vs synthetic peptide
20180228_QX0_MaPe_SA_P509_NEO_5_OP1_3.raw Scan 8847
SVM Score 0.45 Q−Value 0.037035
Endogenous MS2
Rel. intensity [a.u.]
1.0
0.5
0.0
−0.5
−1.0
Spectral Angle = 0
Synthetic peptide MS2
100
200
300
400
500
600
700
m/z
Fragment ion annotation using MaxQuant

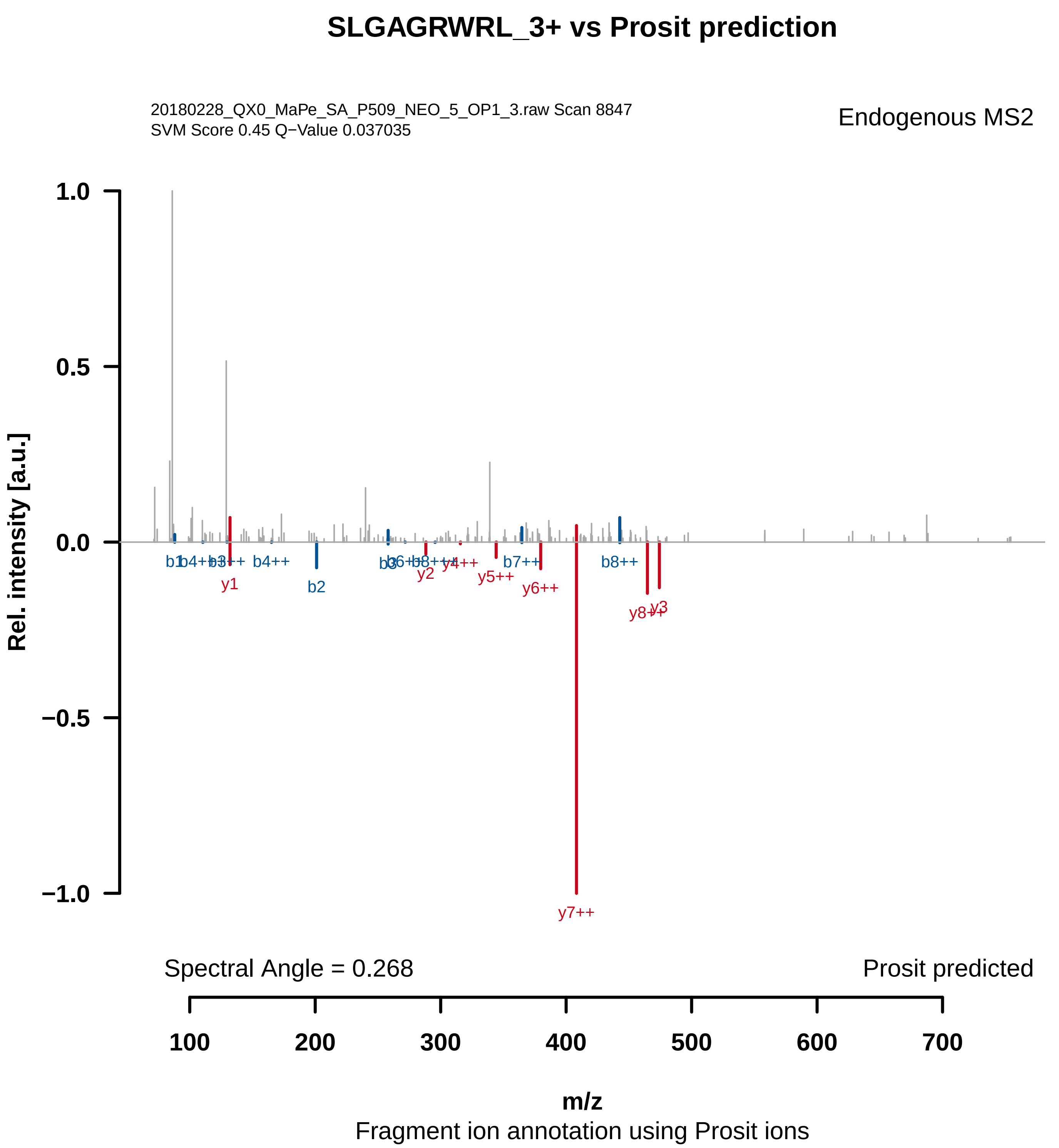
SLGAGRWRL_3+ vs Prosit prediction
20180228_QX0_MaPe_SA_P509_NEO_5_OP1_3.raw Scan 8847
SVM Score 0.45 Q−Value 0.037035
Endogenous MS2
Rel. intensity [a.u.]
1.0
0.5
0.0
−0.5
−1.0
b1
b4++
b3++
b4++
b2
b3
b6++
b8++
y4++
y2
y1
y5++
b7++
y6++
b8++
y8++
y3
y7++
Spectral Angle = 0.268
Prosit predicted
100
200
300
400
500
600
700
m/z
Fragment ion annotation using Prosit ions

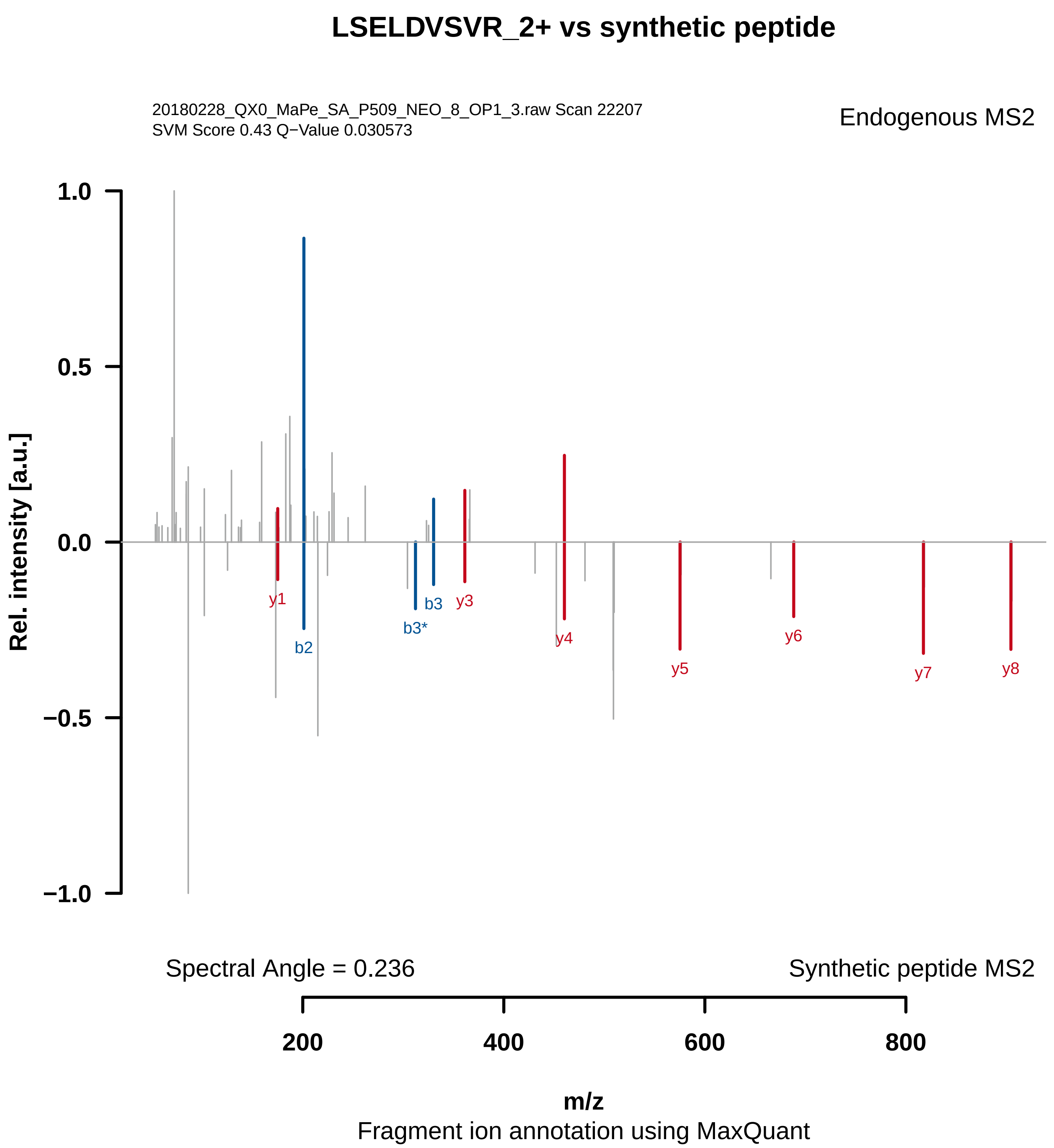
LSELDVSVR_2+ vs synthetic peptide
20180228_QX0_MaPe_SA_P509_NEO_8_OP1_3.raw Scan 22207
SVM Score 0.43 Q−Value 0.030573
Endogenous MS2
Rel. intensity [a.u.]
1.0
0.5
0.0
−0.5
−1.0
y1
b2
b3*
b3
y3
y4
y5
y6
y7
y8
Spectral Angle = 0.236
Synthetic peptide MS2
200
400
600
800
m/z
Fragment ion annotation using MaxQuant

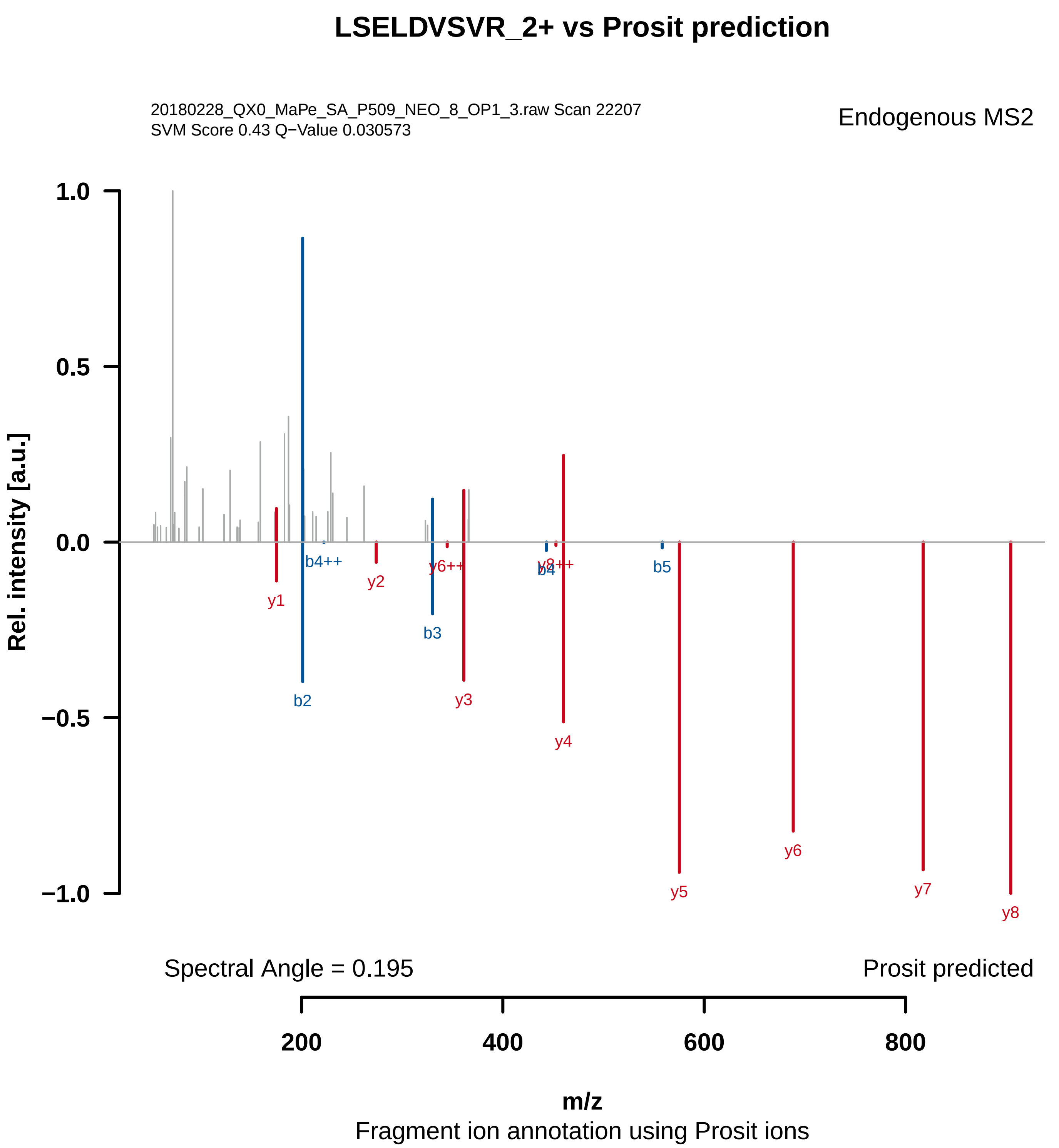
LSELDVSVR_2+ vs Prosit prediction
20180228_QX0_MaPe_SA_P509_NEO_8_OP1_3.raw Scan 22207
SVM Score 0.43 Q−Value 0.030573
Endogenous MS2
Rel. intensity [a.u.]
1.0
0.5
0.0
−0.5
−1.0
b4++
y1
y2
y6++
b3
b2
y3
y8++
b4
y4
b5
y5
y6
y7
y8
Spectral Angle = 0.195
Prosit predicted
200
400
600
800
m/z
Fragment ion annotation using Prosit ions

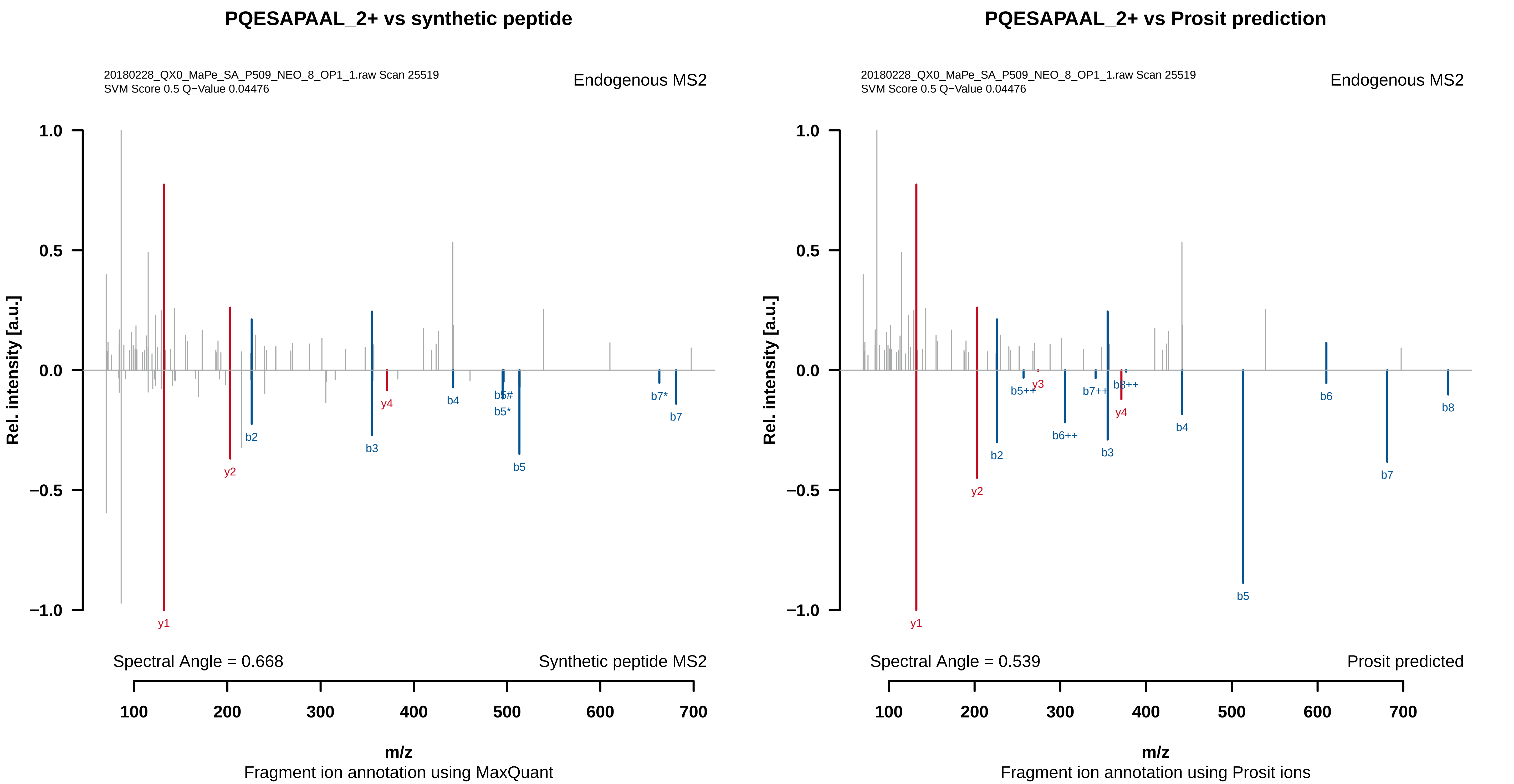
PQESAPAAL_2+ vs synthetic peptide
20180228_QX0_MaPe_SA_P509_NEO_8_OP1_1.raw Scan 25519
SVM Score 0.5 Q−Value 0.04476
Endogenous MS2
Rel. intensity [a.u.]
1.0
0.5
0.0
−0.5
−1.0
y1
y2
b2
b3
y4
b4
b5#
b5*
b5
b7*
b7
Spectral Angle = 0.668
Synthetic peptide MS2
100
200
300
400
500
600
700
m/z
Fragment ion annotation using MaxQuant
PQESAPAAL_2+ vs Prosit prediction
20180228_QX0_MaPe_SA_P509_NEO_8_OP1_1.raw Scan 25519
SVM Score 0.5 Q−Value 0.04476
Endogenous MS2
Rel. intensity [a.u.]
1.0
0.5
0.0
−0.5
−1.0
y1
y2
b2
b5++
y3
b6++
b7++
b8++
b3
y4
b4
b5
b6
b7
b8
Spectral Angle = 0.539
Prosit predicted
100
200
300
400
500
600
700
m/z
Fragment ion annotation using Prosit ions

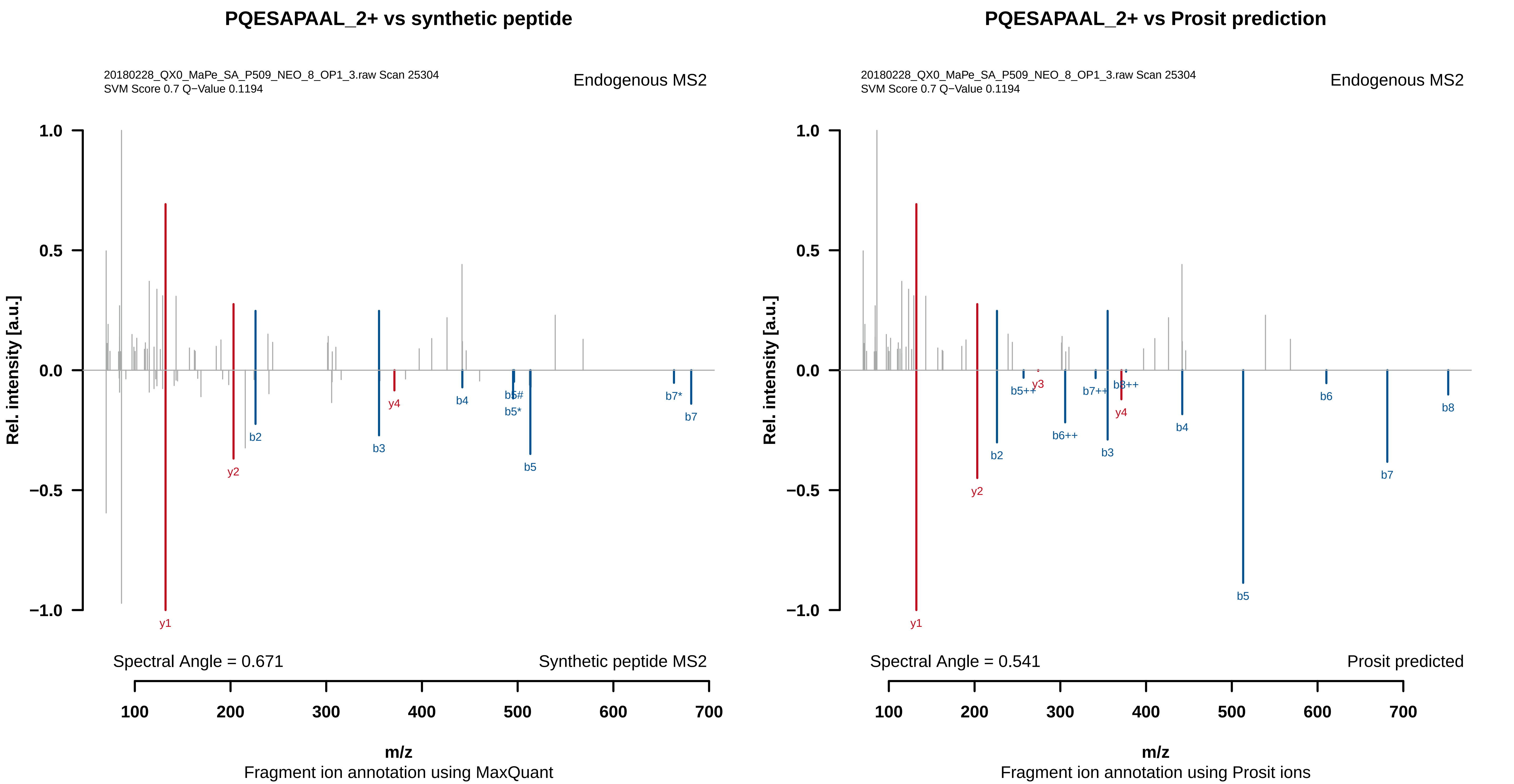

PQESAPAAL_2+ vs synthetic peptide
20180228_QX0_MaPe_SA_P509_NEO_8_OP1_3.raw Scan 25304
SVM Score 0.7 Q−Value 0.1194
Endogenous MS2
Rel. intensity [a.u.]
1.0
0.5
0.0
−0.5
−1.0
y1
y2
b2
b3
y4
b4
b5#
b5*
b5
b7*
b7
Spectral Angle = 0.671
Synthetic peptide MS2
100
200
300
400
500
600
700
m/z
Fragment ion annotation using MaxQuant
PQESAPAAL_2+ vs Prosit prediction
20180228_QX0_MaPe_SA_P509_NEO_8_OP1_3.raw Scan 25304
SVM Score 0.7 Q−Value 0.1194
Endogenous MS2
Rel. intensity [a.u.]
1.0
0.5
0.0
−0.5
−1.0
y1
y2
b2
b5++
y3
b6++
b7++
b3
b8++
y4
b4
b5
b6
b7
b8
Spectral Angle = 0.541
Prosit predicted
100
200
300
400
500
600
700
m/z
Fragment ion annotation using Prosit ions

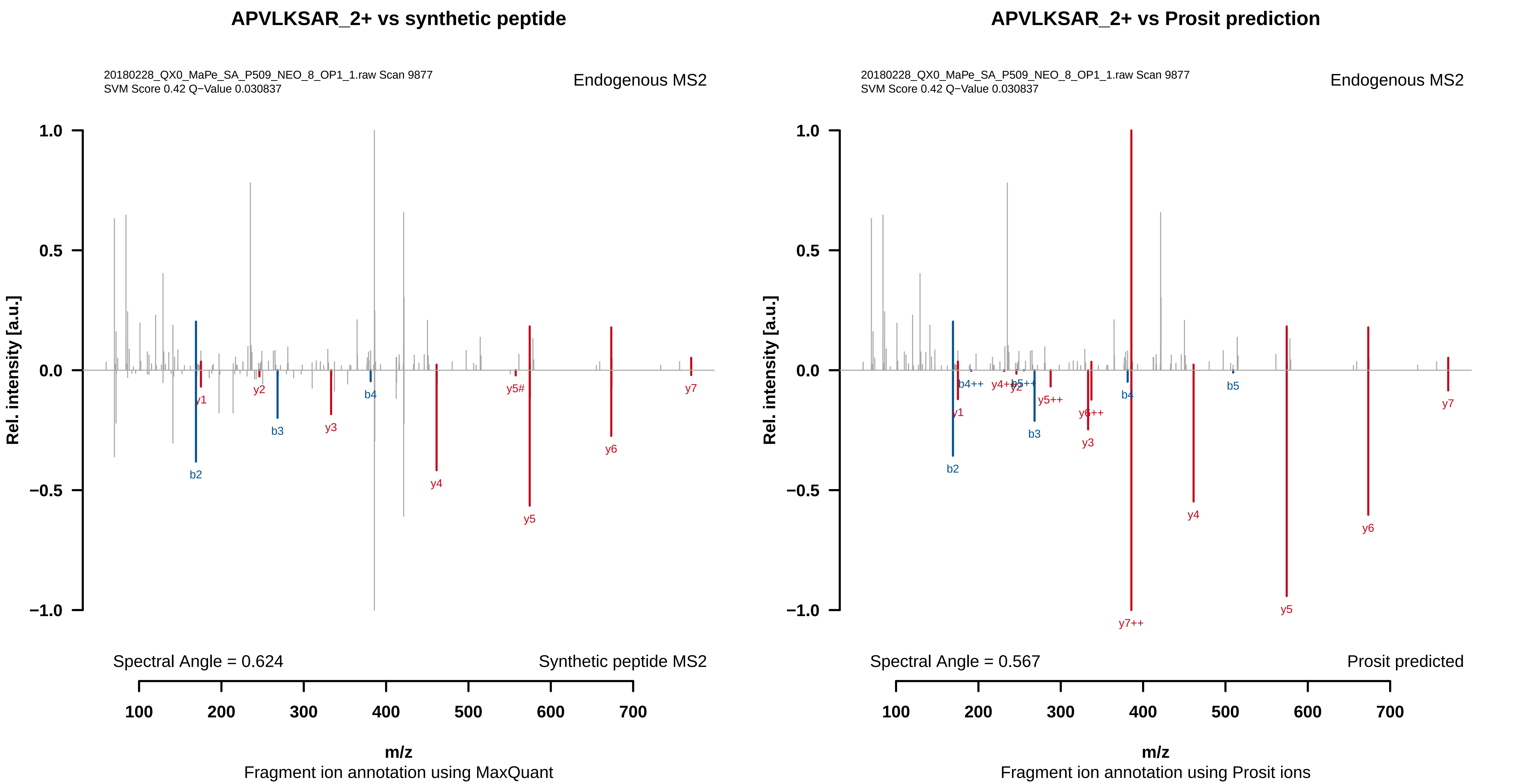
APVLKSAR_2+ vs synthetic peptide
20180228_QX0_MaPe_SA_P509_NEO_8_OP1_1.raw Scan 9877
SVM Score 0.42 Q−Value 0.030837
Endogenous MS2
Rel. intensity [a.u.]
1.0
0.5
0.0
−0.5
−1.0
b2
y1
y2
b3
y3
b4
y4
y5#
y5
y6
y7
Spectral Angle = 0.624
Synthetic peptide MS2
100
200
300
400
500
600
700
m/z
Fragment ion annotation using MaxQuant
APVLKSAR_2+ vs Prosit prediction
20180228_QX0_MaPe_SA_P509_NEO_8_OP1_1.raw Scan 9877
SVM Score 0.42 Q−Value 0.030837
Endogenous MS2
Rel. intensity [a.u.]
1.0
0.5
0.0
−0.5
−1.0
b2
y1
b4++
y4++
b5++
y2
y5++
b3
y6++
y3
b4
y7++
y4
b5
y5
y6
y7
Spectral Angle = 0.567
Prosit predicted
100
200
300
400
500
600
700
m/z
Fragment ion annotation using Prosit ions

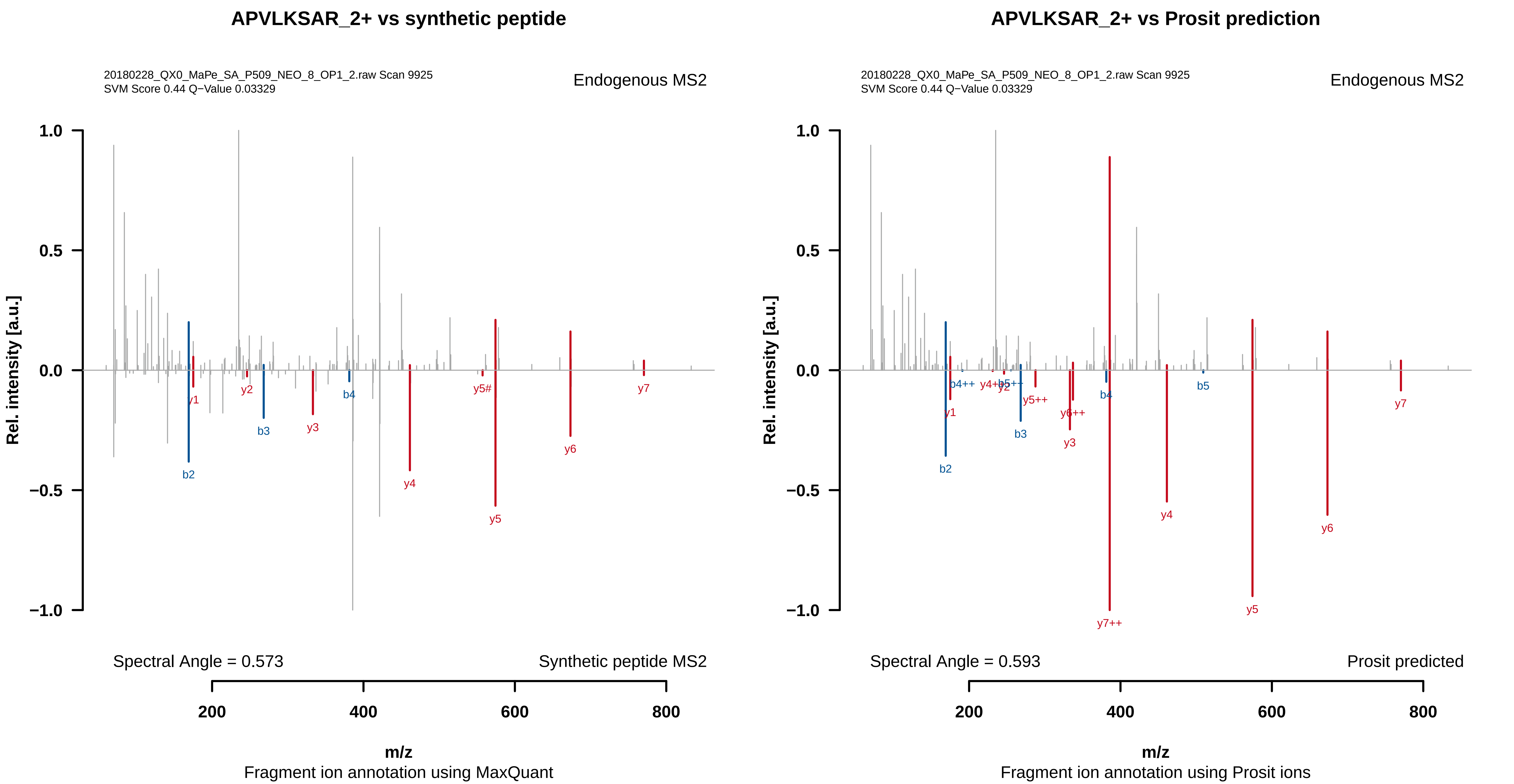
APVLKSAR_2+ vs synthetic peptide
20180228_QX0_MaPe_SA_P509_NEO_8_OP1_2.raw Scan 9925
SVM Score 0.44 Q−Value 0.03329
Endogenous MS2
Rel. intensity [a.u.]
1.0
0.5
0.0
−0.5
−1.0
y1
y2
b2
b3
y3
b4
y4
y5#
y5
y6
y7
Spectral Angle = 0.573
Synthetic peptide MS2
200
400
600
800
m/z
Fragment ion annotation using MaxQuant
APVLKSAR_2+ vs Prosit prediction
20180228_QX0_MaPe_SA_P509_NEO_8_OP1_2.raw Scan 9925
SVM Score 0.44 Q−Value 0.03329
Endogenous MS2
Rel. intensity [a.u.]
1.0
0.5
0.0
−0.5
−1.0
b4++
y4++
b5++
y2
y1
y5++
y6++
b2
b3
y3
b4
b5
y4
y6
y7++
y5
y7
Spectral Angle = 0.593
Prosit predicted
200
400
600
800
m/z
Fragment ion annotation using Prosit ions

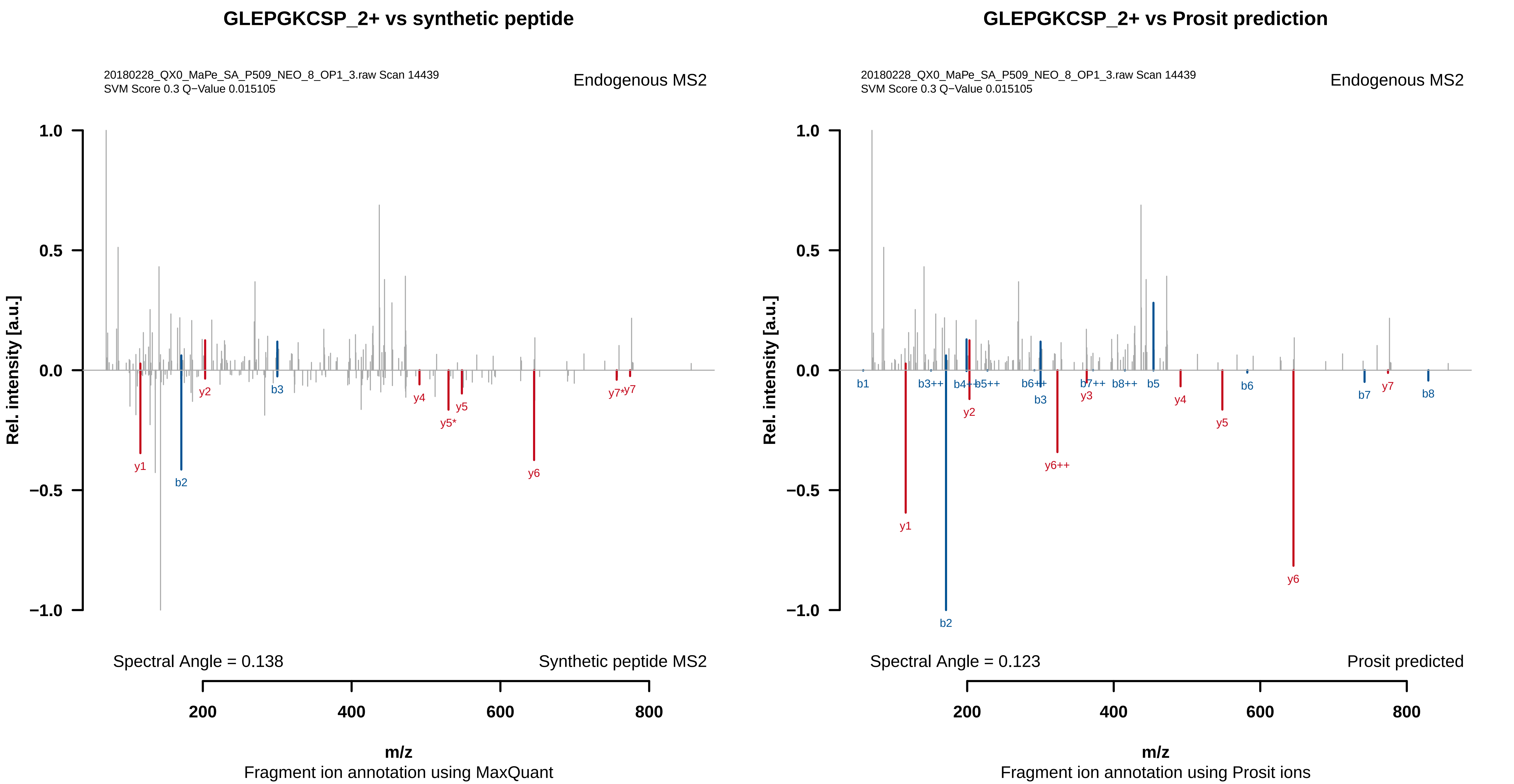

GLEPGKCSP_2+ vs synthetic peptide
20180228_QX0_MaPe_SA_P509_NEO_8_OP1_3.raw Scan 14439
SVM Score 0.3 Q−Value 0.015105
Endogenous MS2
Rel. intensity [a.u.]
1.0
0.5
0.0
−0.5
−1.0
y1
b2
y2
b3
y4
y5*
y5
y6
y7*
y7
Spectral Angle = 0.138
Synthetic peptide MS2
200
400
600
800
m/z
Fragment ion annotation using MaxQuant
GLEPGKCSP_2+ vs Prosit prediction
20180228_QX0_MaPe_SA_P509_NEO_8_OP1_3.raw Scan 14439
SVM Score 0.3 Q−Value 0.015105
Endogenous MS2
Rel. intensity [a.u.]
1.0
0.5
0.0
−0.5
−1.0
b1
b3++
b4++
b5++
b6++
b7++
b8++
b5
b6
b7
b8
b3
y1
y2
y3
y6++
y4
y5
y6
y7
b2
Spectral Angle = 0.123
Prosit predicted
200
400
600
800
m/z
Fragment ion annotation using Prosit ions

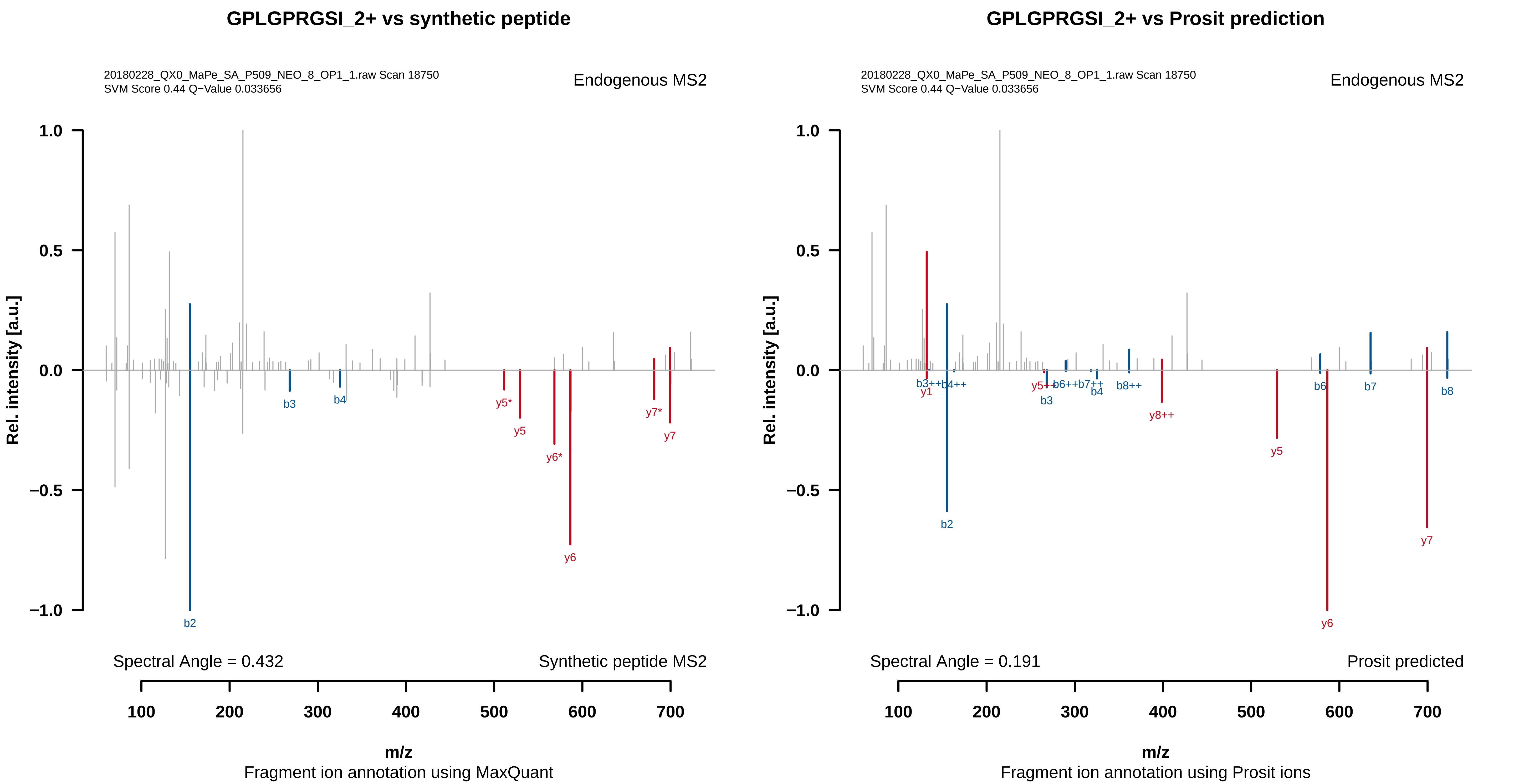

GPLGPRGSI_2+ vs synthetic peptide
20180228_QX0_MaPe_SA_P509_NEO_8_OP1_1.raw Scan 18750
SVM Score 0.44 Q−Value 0.033656
Endogenous MS2
Rel. intensity [a.u.]
b2
b3
b4
y5*
y5
y6*
y6
y7*
y7
Spectral Angle = 0.432
Synthetic peptide MS2
m/z
Fragment ion annotation using MaxQuant
GPLGPRGSI_2+ vs Prosit prediction
20180228_QX0_MaPe_SA_P509_NEO_8_OP1_1.raw Scan 18750
SVM Score 0.44 Q−Value 0.033656
Endogenous MS2
Rel. intensity [a.u.]
b3++
b4++
y1
y5++
b6++
b7++
b3
b4
b8++
y8++
y5
b6
b7
b8
b2
y6
y7
Spectral Angle = 0.191
Prosit predicted
m/z
Fragment ion annotation using Prosit ions

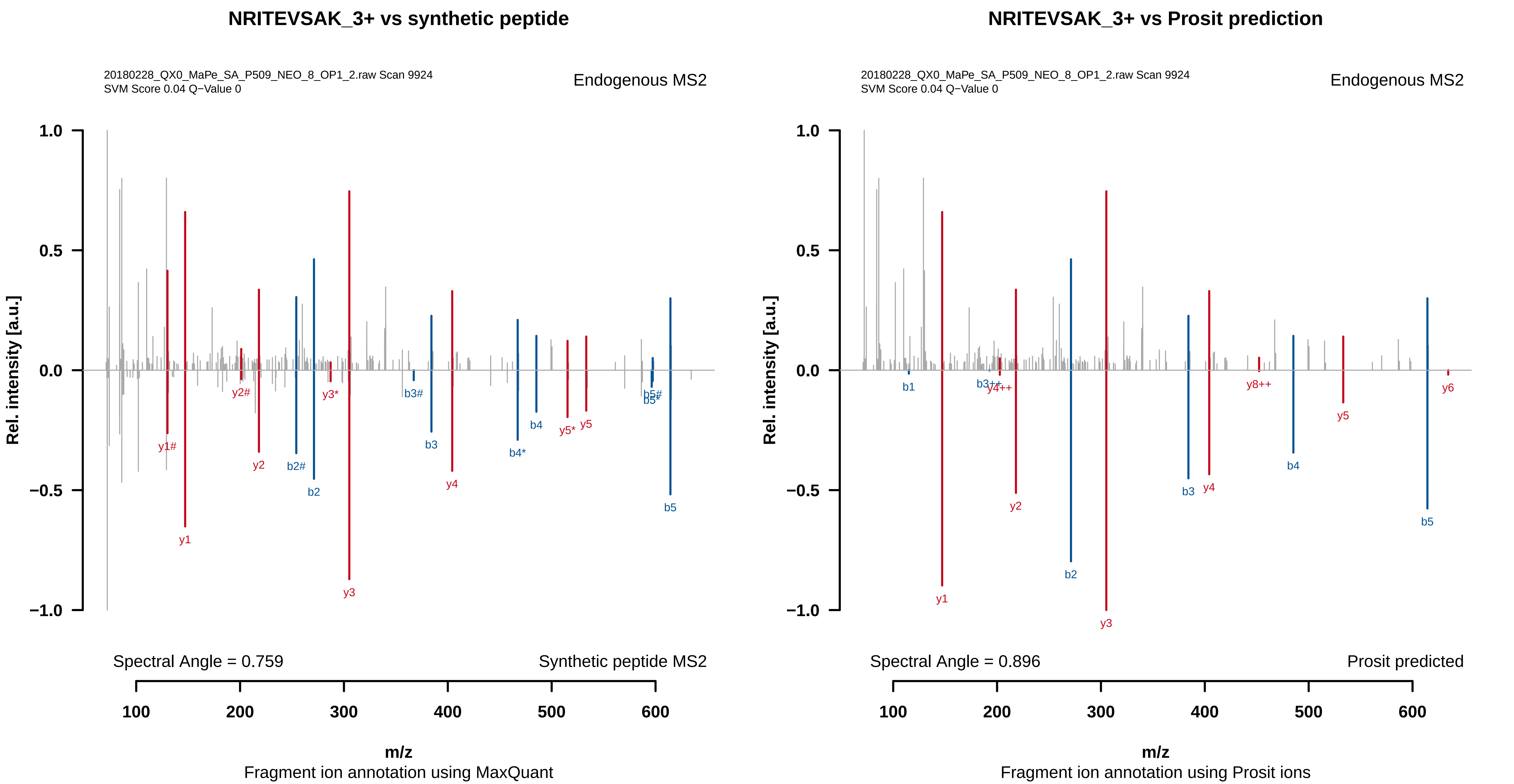

NRITEVSAK_3+ vs synthetic peptide
20180228_QX0_MaPe_SA_P509_NEO_8_OP1_2.raw Scan 9924
SVM Score 0.04 Q−Value 0
Endogenous MS2
Rel. intensity [a.u.]
1.0
0.5
0.0
−0.5
−1.0
y1# y1 y2# y2 b2# b2 y3* y3 b3# b3 y4 b4* b4 y5* y5 b5# b5* b5
Spectral Angle = 0.759
Synthetic peptide MS2
100
200
300
400
500
600
m/z
Fragment ion annotation using MaxQuant
NRITEVSAK_3+ vs Prosit prediction
20180228_QX0_MaPe_SA_P509_NEO_8_OP1_2.raw Scan 9924
SVM Score 0.04 Q−Value 0
Endogenous MS2
Rel. intensity [a.u.]
1.0
0.5
0.0
−0.5
−1.0
b1 y1 b3++ y4++ y2 b2 y3 b3 y4 y8++ b4 y5 b5 y6
Spectral Angle = 0.896
Prosit predicted
100
200
300
400
500
600
m/z
Fragment ion annotation using Prosit ions

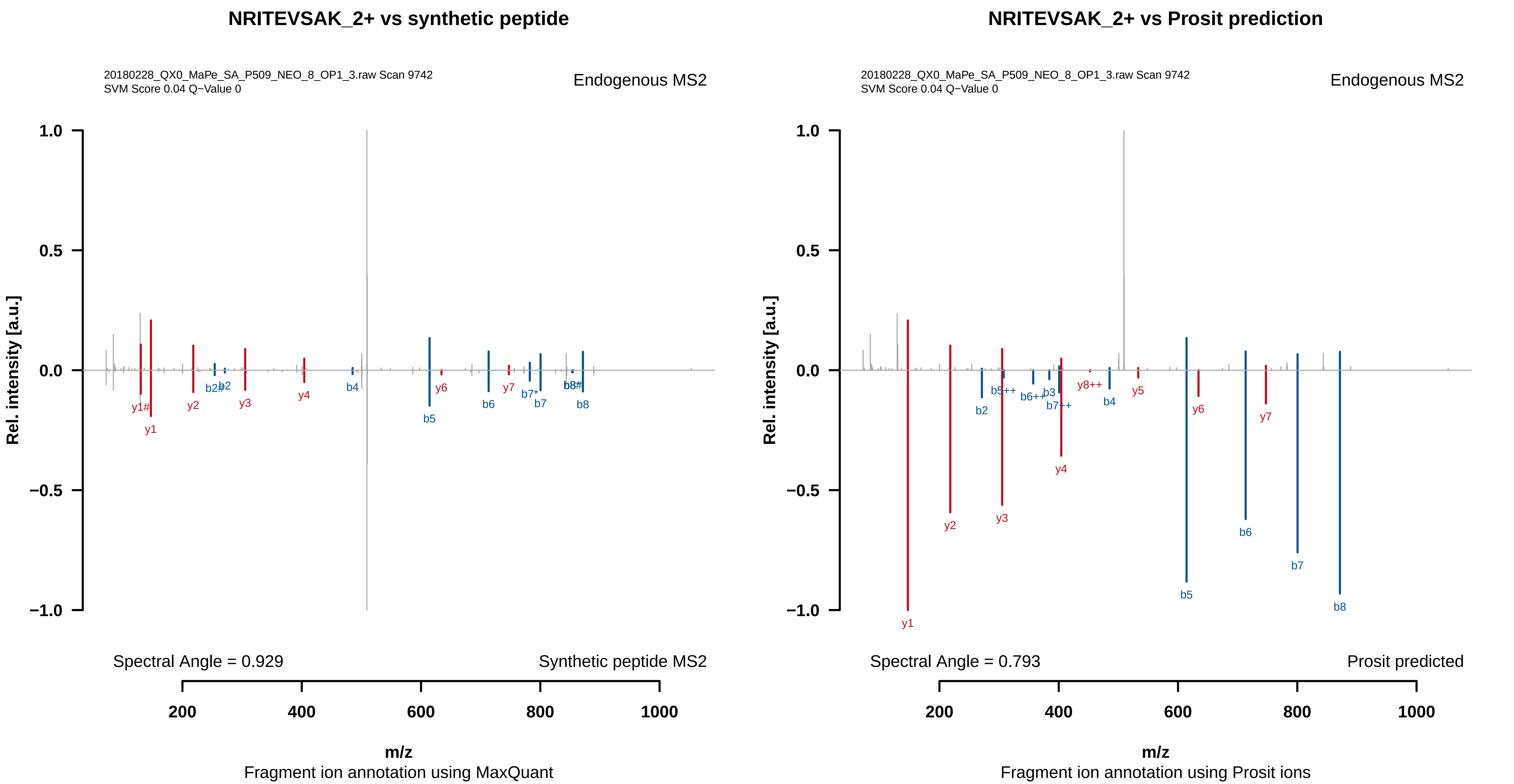
NRITEVSAK_2+ vs synthetic peptide
20180228_QX0_MaPe_SA_P509_NEO_8_OP1_3.raw Scan 9742
SVM Score 0.04 Q−Value 0
Endogenous MS2
Rel. intensity [a.u.]
1.0
0.5
0.0
−0.5
−1.0
y1#
y1
y2
b2#
b2
y3
y4
b4
b5
y6
b6
y7
b7*
b7
b8#
b8
Spectral Angle = 0.929
Synthetic peptide MS2
200
400
600
800
1000
m/z
Fragment ion annotation using MaxQuant
NRITEVSAK_2+ vs Prosit prediction
20180228_QX0_MaPe_SA_P509_NEO_8_OP1_3.raw Scan 9742
SVM Score 0.04 Q−Value 0
Endogenous MS2
Rel. intensity [a.u.]
y1
y2
b2
b5++
y3
b6++
b3
b7++
y4
y8++
b4
y5
b5
y6
b6
y7
b7
b8
Spectral Angle = 0.793
Prosit predicted
m/z
Fragment ion annotation using Prosit ions

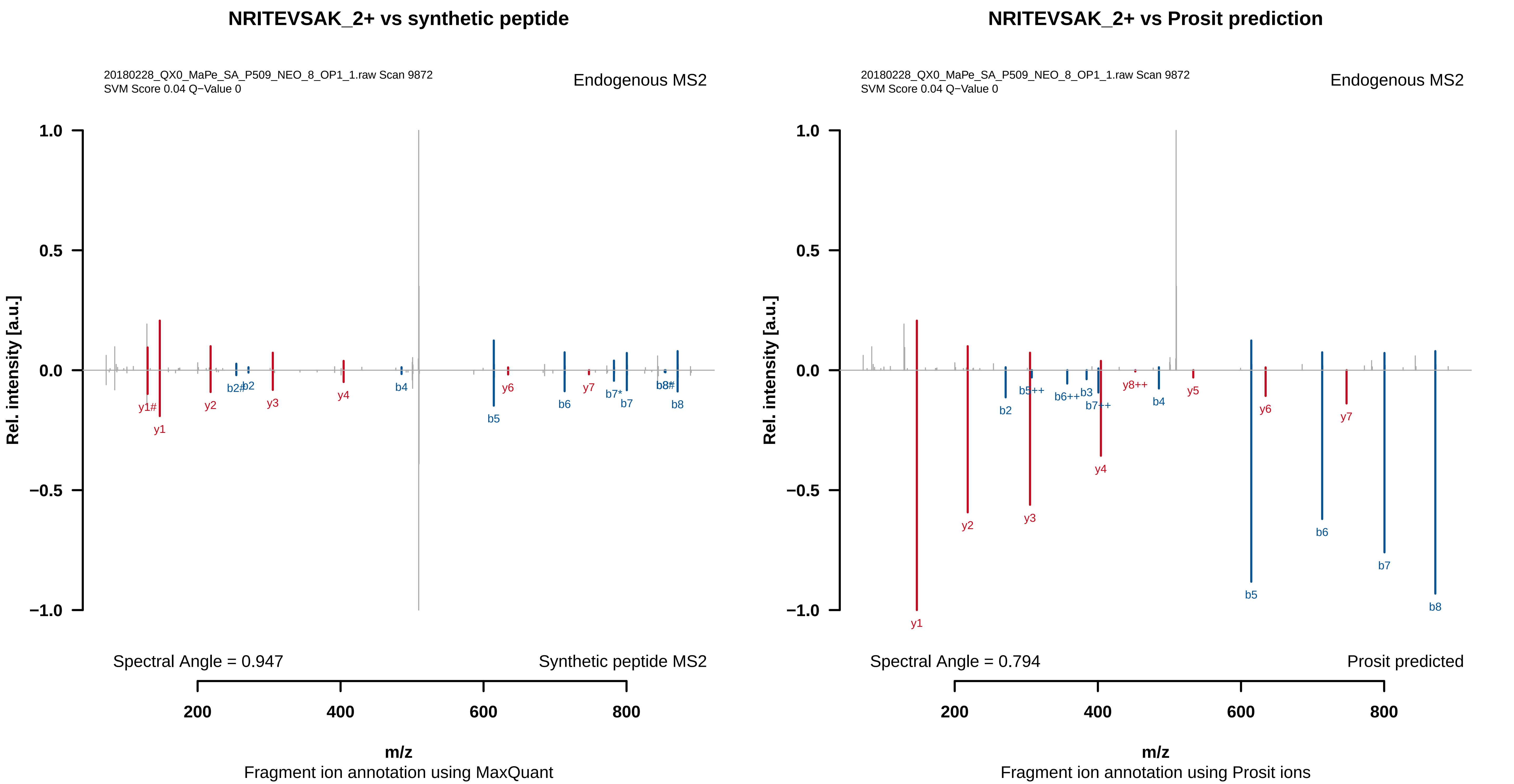
NRITEVSAK_2+ vs synthetic peptide
20180228_QX0_MaPe_SA_P509_NEO_8_OP1_1.raw Scan 9872
SVM Score 0.04 Q−Value 0
Endogenous MS2
Rel. intensity [a.u.]
1.0
0.5
0.0
−0.5
−1.0
y1#
y1
y2
b2#
b2
y3
y4
b4
b5
y6
b6
y7
b7*
b7
b8#
b8
Spectral Angle = 0.947
Synthetic peptide MS2
200
400
600
800
m/z
Fragment ion annotation using MaxQuant
NRITEVSAK_2+ vs Prosit prediction
20180228_QX0_MaPe_SA_P509_NEO_8_OP1_1.raw Scan 9872
SVM Score 0.04 Q−Value 0
Endogenous MS2
Rel. intensity [a.u.]
1.0
0.5
0.0
−0.5
−1.0
y1
y2
b2
b5++
y3
b6++
b3
b7++
y4
y8++
b4
y5
b5
y6
b6
y7
b7
b8
Spectral Angle = 0.794
Prosit predicted
200
400
600
800
m/z
Fragment ion annotation using Prosit ions

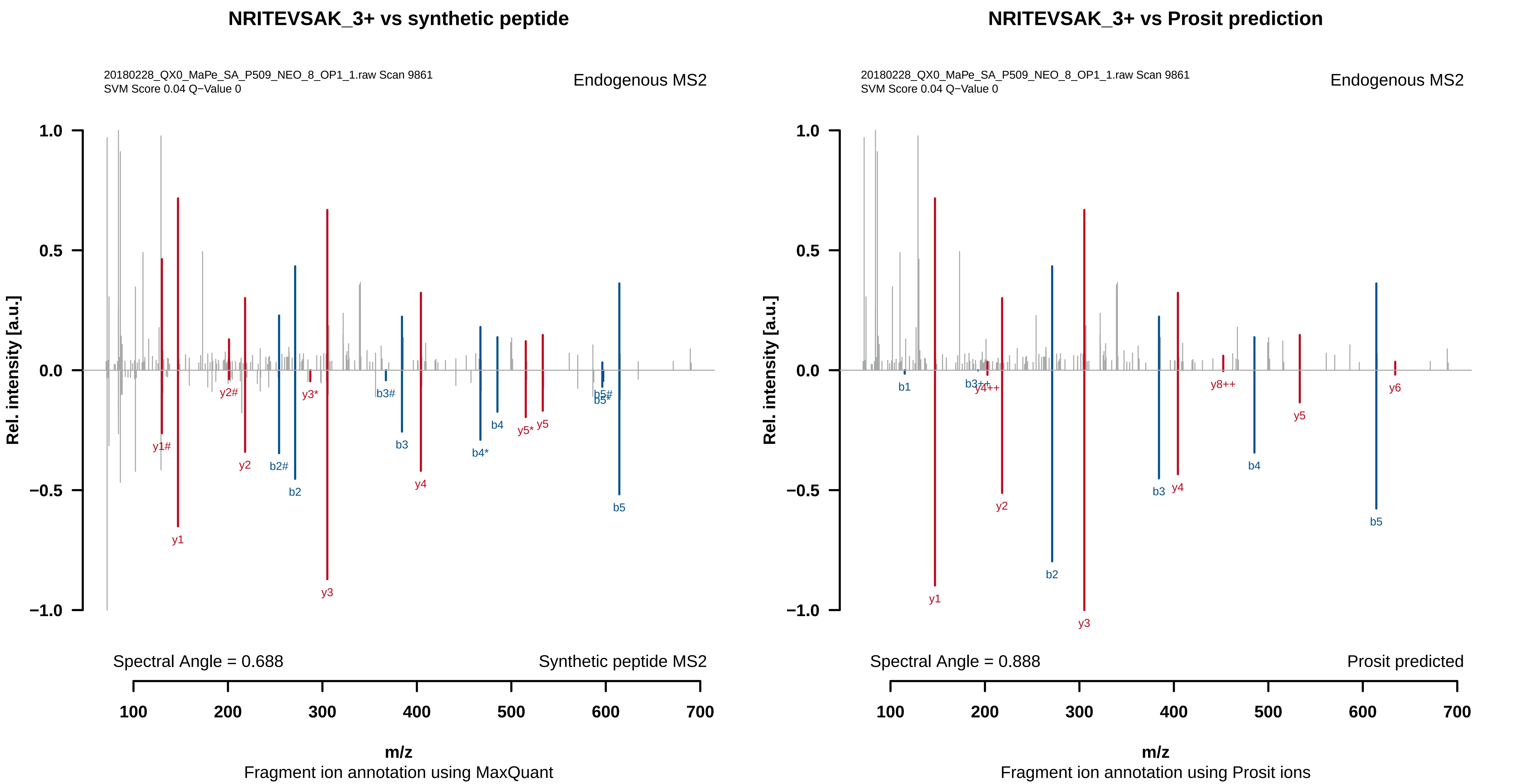
NRITEVSAK_3+ vs synthetic peptide
20180228_QX0_MaPe_SA_P509_NEO_8_OP1_1.raw Scan 9861
SVM Score 0.04 Q−Value 0
Endogenous MS2
Rel. intensity [a.u.]
1.0
0.5
0.0
−0.5
−1.0
y1#
y1
y2#
y2
b2#
b2
y3*
y3
b3#
b3
y4
b4*
b4
y5*
y5
b5#
b5*
b5
Spectral Angle = 0.688
Synthetic peptide MS2
100
200
300
400
500
600
700
m/z
Fragment ion annotation using MaxQuant
NRITEVSAK_3+ vs Prosit prediction
20180228_QX0_MaPe_SA_P509_NEO_8_OP1_1.raw Scan 9861
SVM Score 0.04 Q−Value 0
Endogenous MS2
Rel. intensity [a.u.]
1.0
0.5
0.0
−0.5
−1.0
b1
y1
b3++
y4++
y2
b2
y3
b3
y4
y8++
b4
y5
b5
y6
Spectral Angle = 0.888
Prosit predicted
100
200
300
400
500
600
700
m/z
Fragment ion annotation using Prosit ions

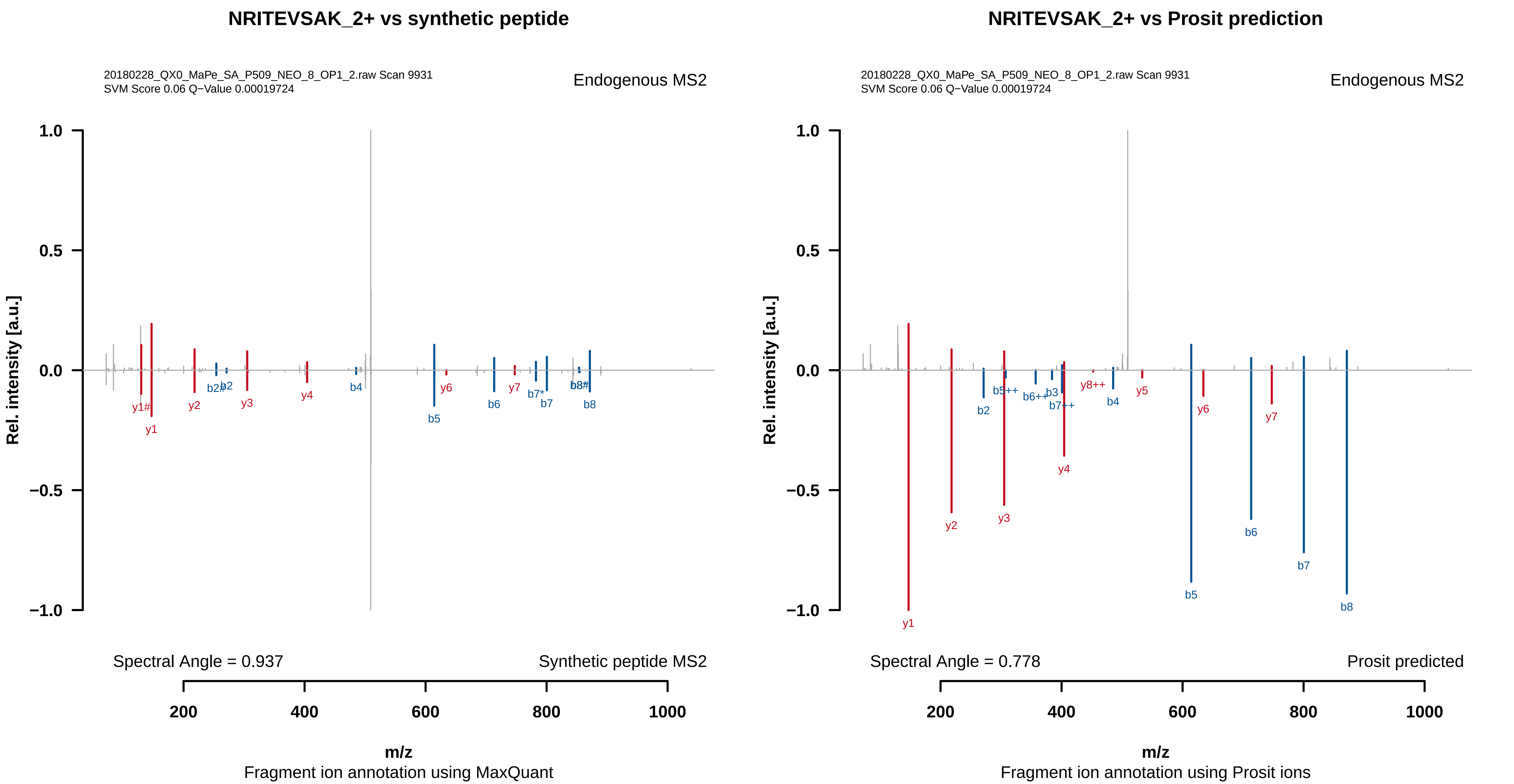

NRITEVSAK_2+ vs synthetic peptide
20180228_QX0_MaPe_SA_P509_NEO_8_OP1_2.raw Scan 9931
SVM Score 0.06 Q−Value 0.00019724
Endogenous MS2
Rel. intensity [a.u.]
1.0
0.5
0.0
−0.5
−1.0
y1#
y1
y2
b2#
b2
y3
y4
b4
b5
y6
b6
y7
b7*
b7
b8#
b8
Spectral Angle = 0.937
Synthetic peptide MS2
200
400
600
800
1000
m/z
Fragment ion annotation using MaxQuant
NRITEVSAK_2+ vs Prosit prediction
20180228_QX0_MaPe_SA_P509_NEO_8_OP1_2.raw Scan 9931
SVM Score 0.06 Q−Value 0.00019724
Endogenous MS2
Rel. intensity [a.u.]
1.0
0.5
0.0
−0.5
−1.0
y1
y2
b2
y3
b5++
b6++
b3
b7++
y4
y8++
b4
y5
b5
y6
b6
y7
b7
b8
Spectral Angle = 0.778
Prosit predicted
200
400
600
800
1000
m/z
Fragment ion annotation using Prosit ions

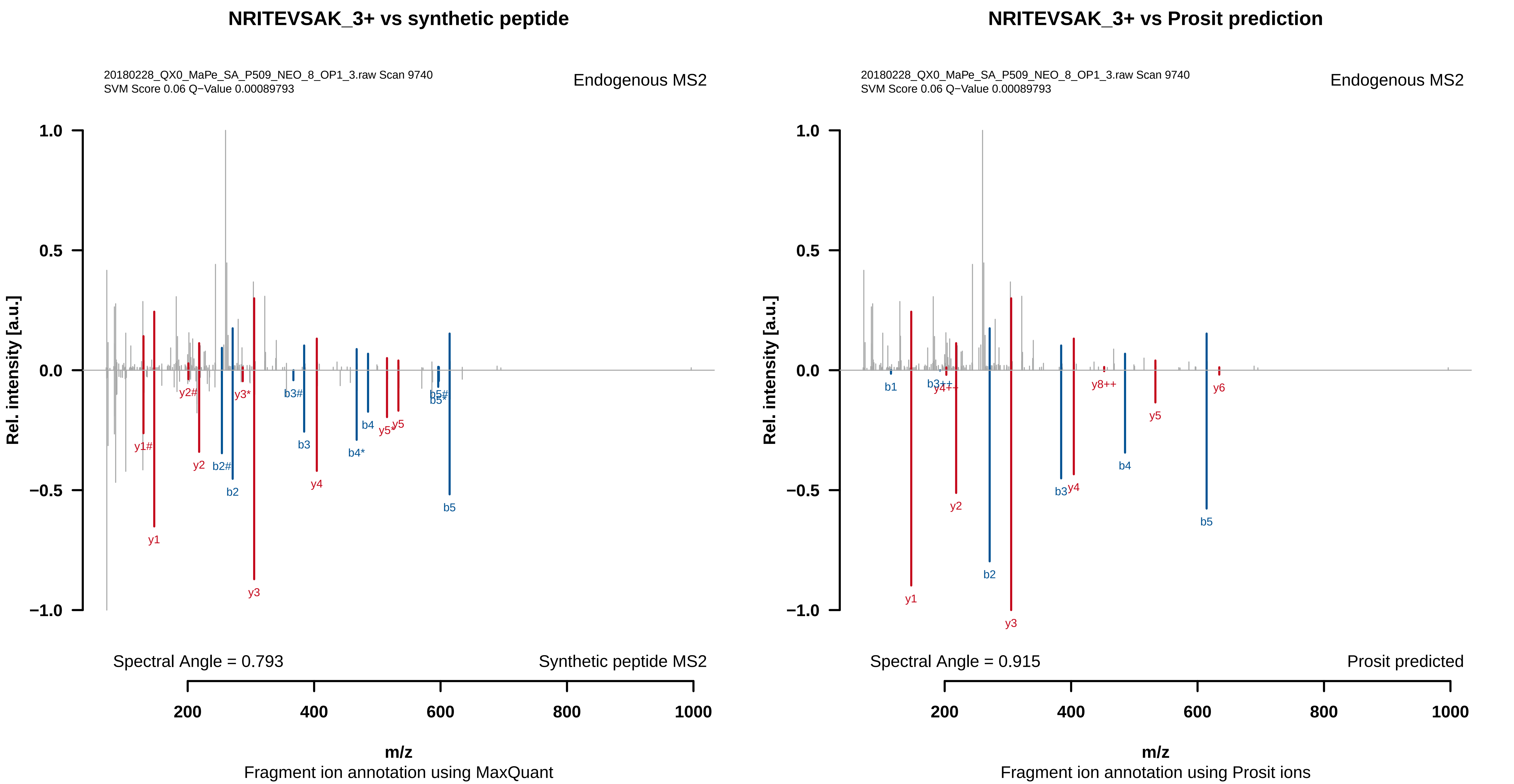

NRITEVSAK_3+ vs synthetic peptide
20180228_QX0_MaPe_SA_P509_NEO_8_OP1_3.raw Scan 9740
SVM Score 0.06 Q−Value 0.00089793
Endogenous MS2
Rel. intensity [a.u.]
1.0
0.5
0.0
−0.5
−1.0
y1#
y1
y2#
y2
b2#
b2
y3*
y3
b3#
b3
y4
b4*
b4
y5*
y5
b5#
b5*
b5
Spectral Angle = 0.793
Synthetic peptide MS2
200
400
600
800
1000
m/z
Fragment ion annotation using MaxQuant
NRITEVSAK_3+ vs Prosit prediction
20180228_QX0_MaPe_SA_P509_NEO_8_OP1_3.raw Scan 9740
SVM Score 0.06 Q−Value 0.00089793
Endogenous MS2
Rel. intensity [a.u.]
1.0
0.5
0.0
−0.5
−1.0
b1
y1
b3++
y4++
y2
b2
y3
b3
y4
y8++
b4
y5
b5
y6
Spectral Angle = 0.915
Prosit predicted
200
400
600
800
1000
m/z
Fragment ion annotation using Prosit ions

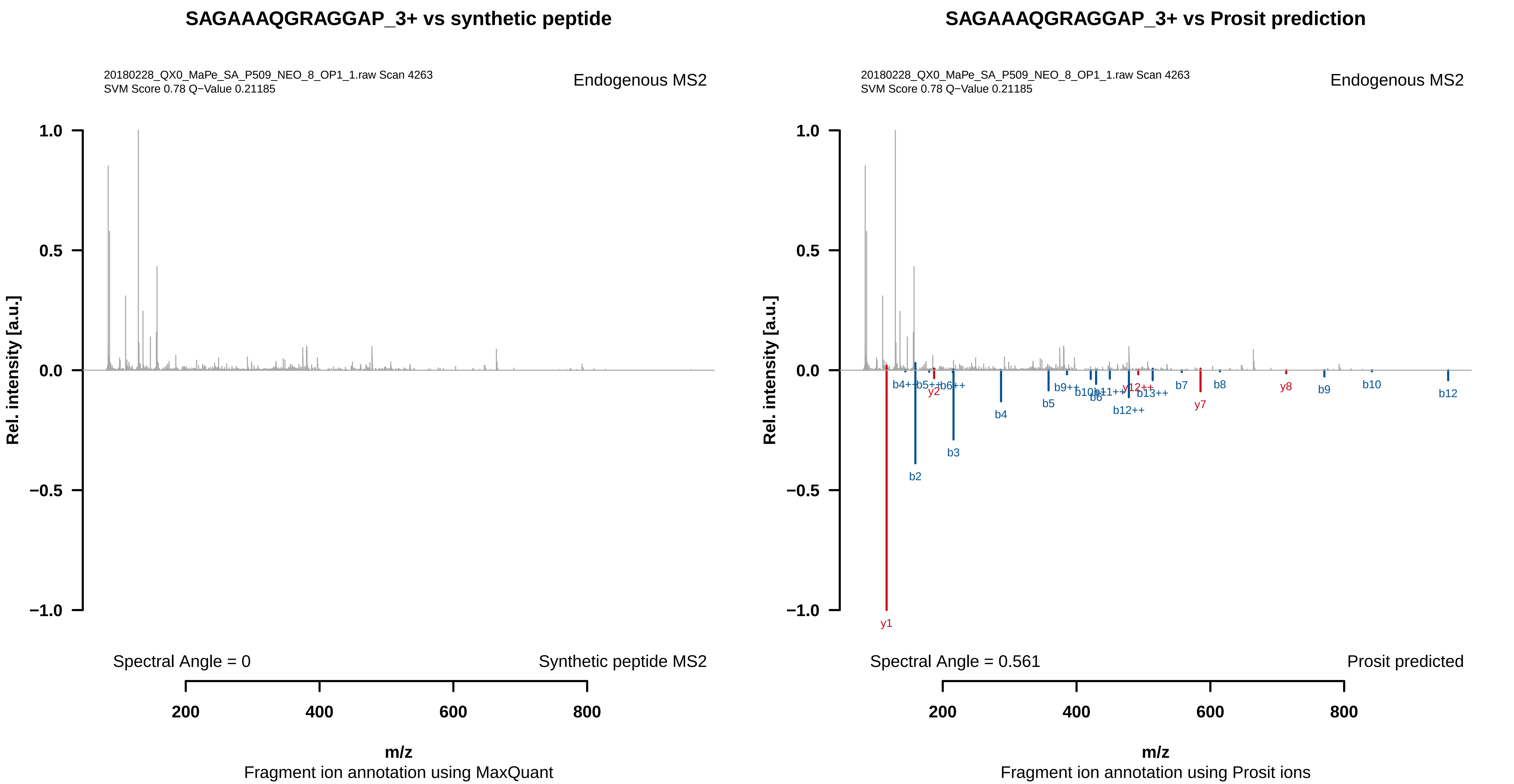

SAGAAAQGRAGGAP_3+ vs synthetic peptide
20180228_QX0_MaPe_SA_P509_NEO_8_OP1_1.raw Scan 4263
SVM Score 0.78 Q−Value 0.21185
Endogenous MS2
Rel. intensity [a.u.]
Spectral Angle = 0
Synthetic peptide MS2
m/z
Fragment ion annotation using MaxQuant
SAGAAAQGRAGGAP_3+ vs Prosit prediction
20180228_QX0_MaPe_SA_P509_NEO_8_OP1_1.raw Scan 4263
SVM Score 0.78 Q−Value 0.21185
Endogenous MS2
Rel. intensity [a.u.]
y1
y2
y7
y8
y12++
b2
b3
b4
b5
b6
b7
b8
b9
b10
b12
b4++
b5++
b6++
b9++
b10++
b11++
b12++
b13++
Spectral Angle = 0.561
Prosit predicted
m/z
Fragment ion annotation using Prosit ions

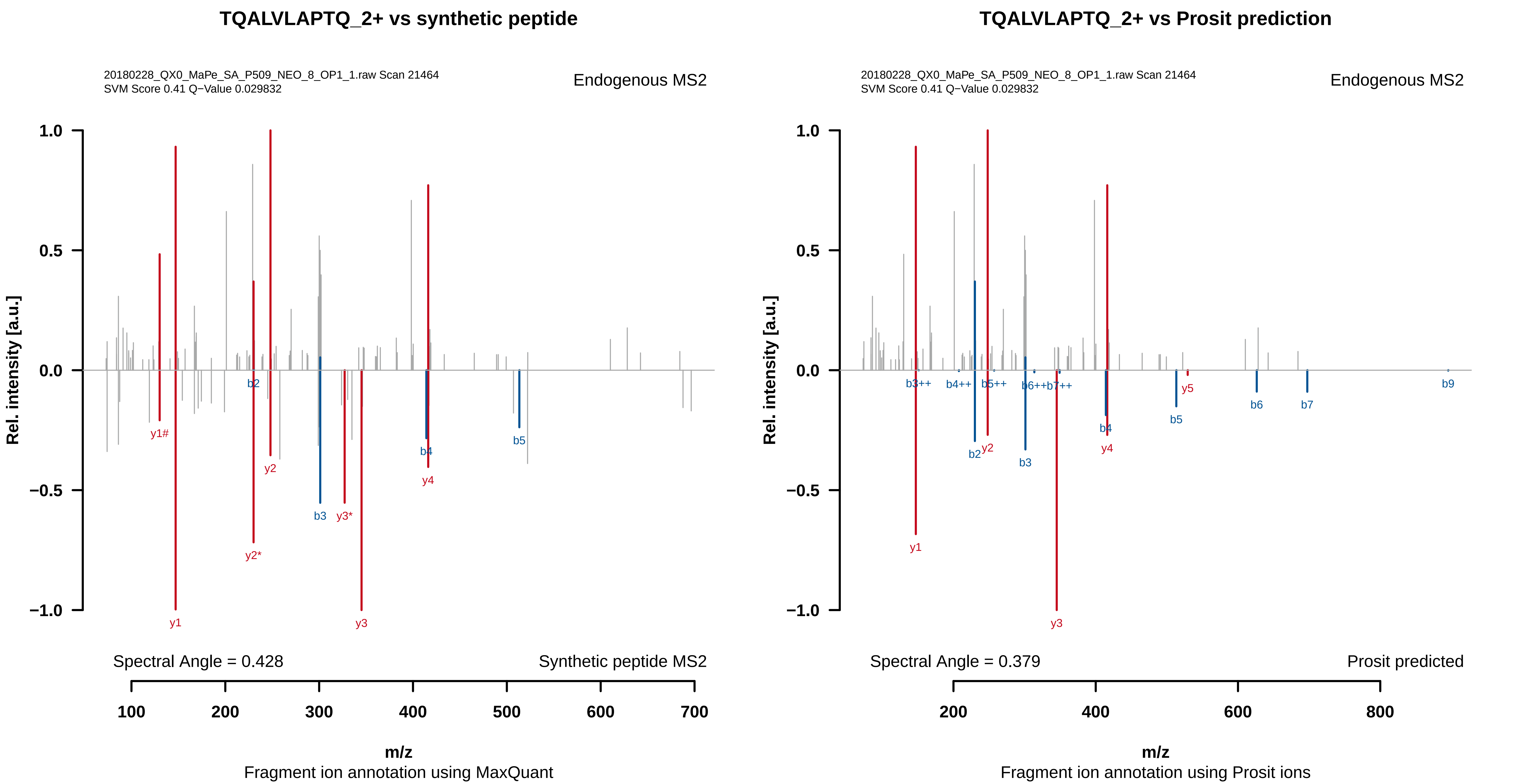
TQALVLAPTQ_2+ vs synthetic peptide
20180228_QX0_MaPe_SA_P509_NEO_8_OP1_1.raw Scan 21464
SVM Score 0.41 Q−Value 0.029832
Endogenous MS2
Rel. intensity [a.u.]
Spectral Angle = 0.428
Synthetic peptide MS2
m/z
Fragment ion annotation using MaxQuant
TQALVLAPTQ_2+ vs Prosit prediction
20180228_QX0_MaPe_SA_P509_NEO_8_OP1_1.raw Scan 21464
SVM Score 0.41 Q−Value 0.029832
Endogenous MS2
Rel. intensity [a.u.]
Spectral Angle = 0.379
Prosit predicted
m/z
Fragment ion annotation using Prosit ions

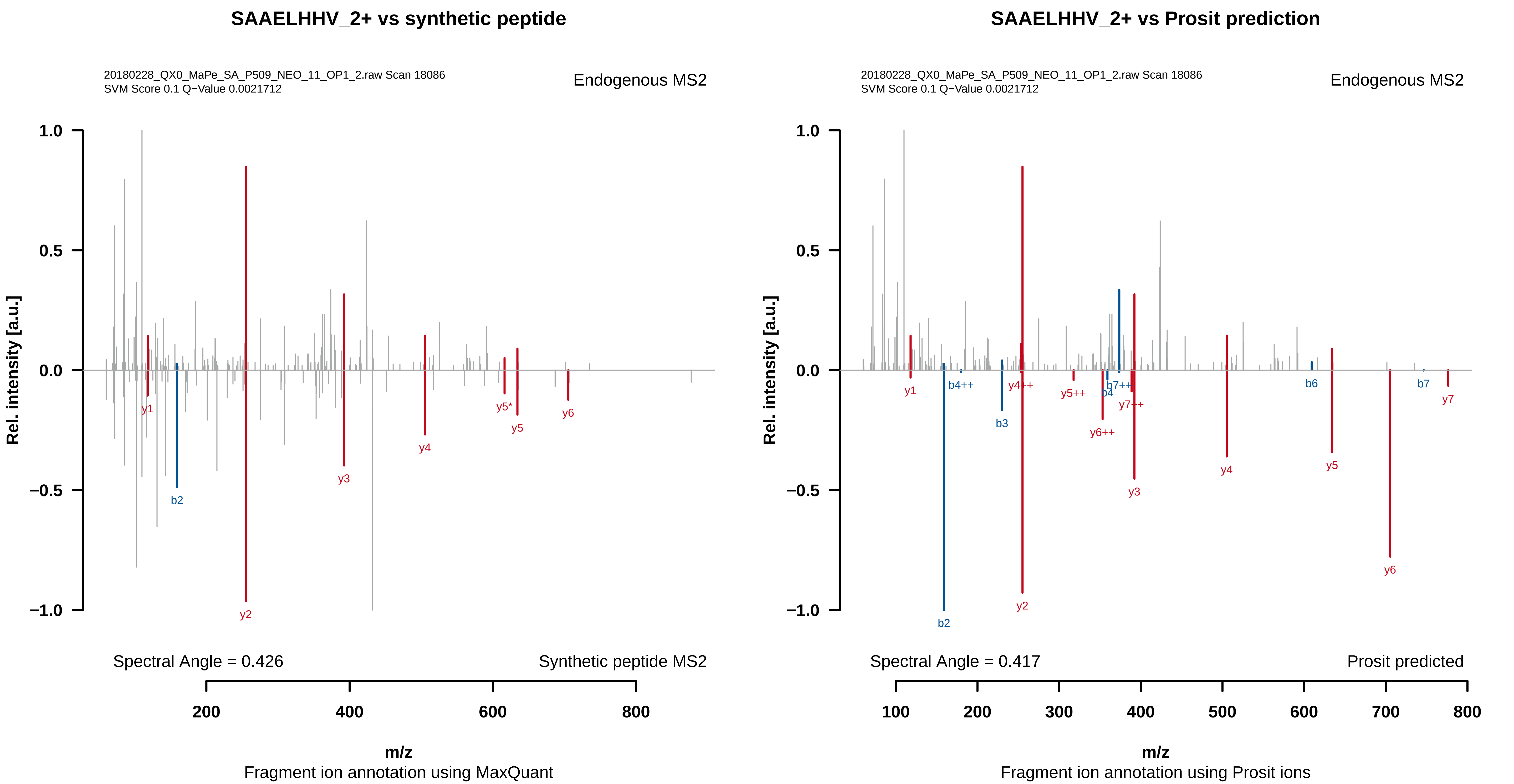
SAAELHHV_2+ vs synthetic peptide
20180228_QX0_MaPe_SA_P509_NEO_11_OP1_2.raw Scan 18086
SVM Score 0.1 Q−Value 0.0021712
Endogenous MS2
Rel. intensity [a.u.]
1.0
0.5
0.0
−0.5
−1.0
y1
b2
y2
y3
y4
y5*
y5
y6
Spectral Angle = 0.426
Synthetic peptide MS2
200
400
600
800
m/z
Fragment ion annotation using MaxQuant
SAAELHHV_2+ vs Prosit prediction
20180228_QX0_MaPe_SA_P509_NEO_11_OP1_2.raw Scan 18086
SVM Score 0.1 Q−Value 0.0021712
Endogenous MS2
Rel. intensity [a.u.]
1.0
0.5
0.0
−0.5
−1.0
y1
b4++
y4++
y5++
b4
b7++
y7++
y6++
b3
b2
y2
y3
y4
b6
y5
y6
b7
y7
Spectral Angle = 0.417
Prosit predicted
100
200
300
400
500
600
700
800
m/z
Fragment ion annotation using Prosit ions

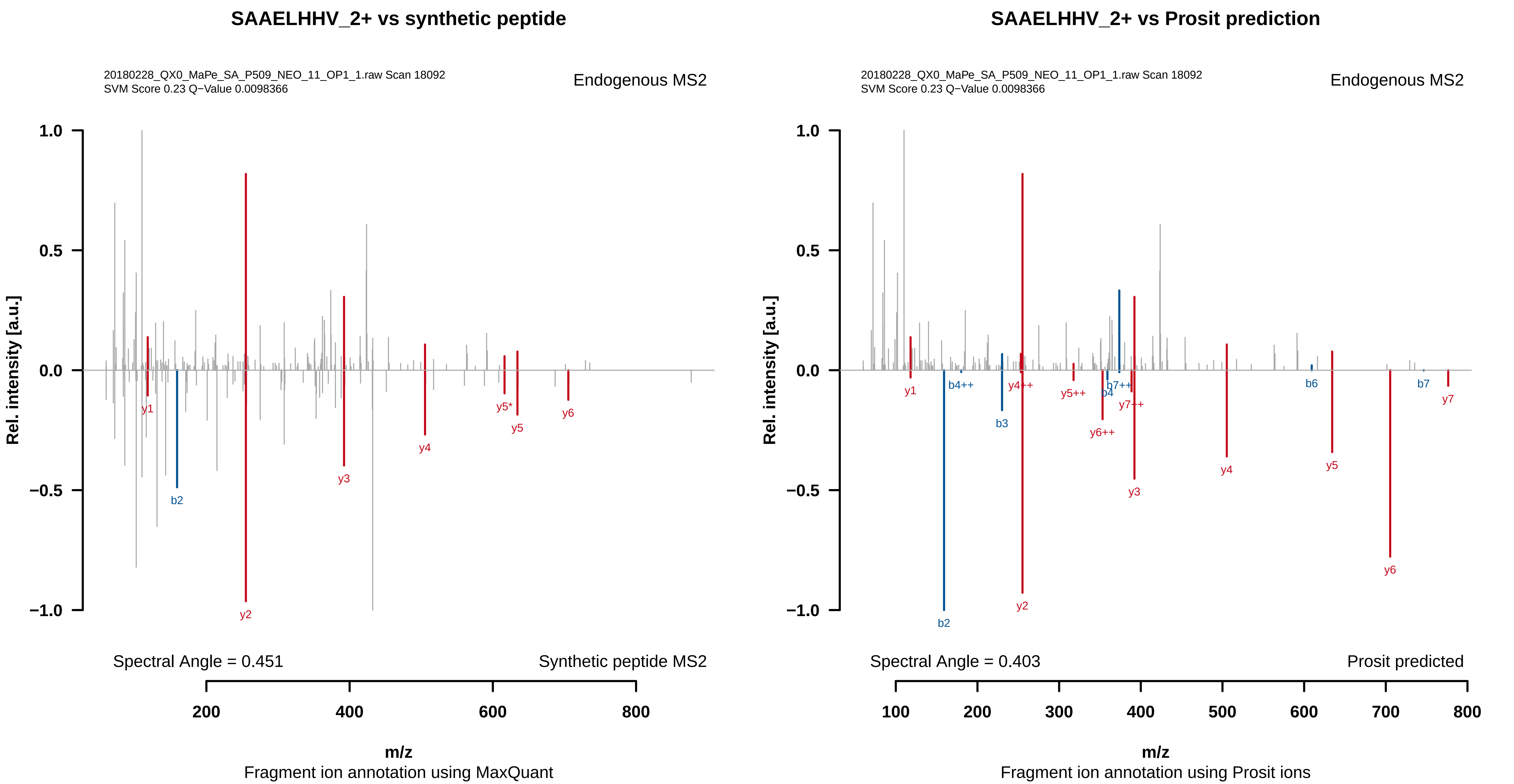

SAAELHHV_2+ vs synthetic peptide
20180228_QX0_MaPe_SA_P509_NEO_11_OP1_1.raw Scan 18092
SVM Score 0.23 Q−Value 0.0098366
Endogenous MS2
Rel. intensity [a.u.]
1.0
0.5
0.0
−0.5
−1.0
y1
b2
y2
y3
y4
y5*
y5
y6
Spectral Angle = 0.451
Synthetic peptide MS2
200
400
600
800
m/z
Fragment ion annotation using MaxQuant
SAAELHHV_2+ vs Prosit prediction
20180228_QX0_MaPe_SA_P509_NEO_11_OP1_1.raw Scan 18092
SVM Score 0.23 Q−Value 0.0098366
Endogenous MS2
Rel. intensity [a.u.]
1.0
0.5
0.0
−0.5
−1.0
y1
b4++
y4++
y5++
b7++
b4
y7++
y6++
b3
b6
b7
y7
y5
y4
y3
y6
y2
b2
Spectral Angle = 0.403
Prosit predicted
100
200
300
400
500
600
700
800
m/z
Fragment ion annotation using Prosit ions

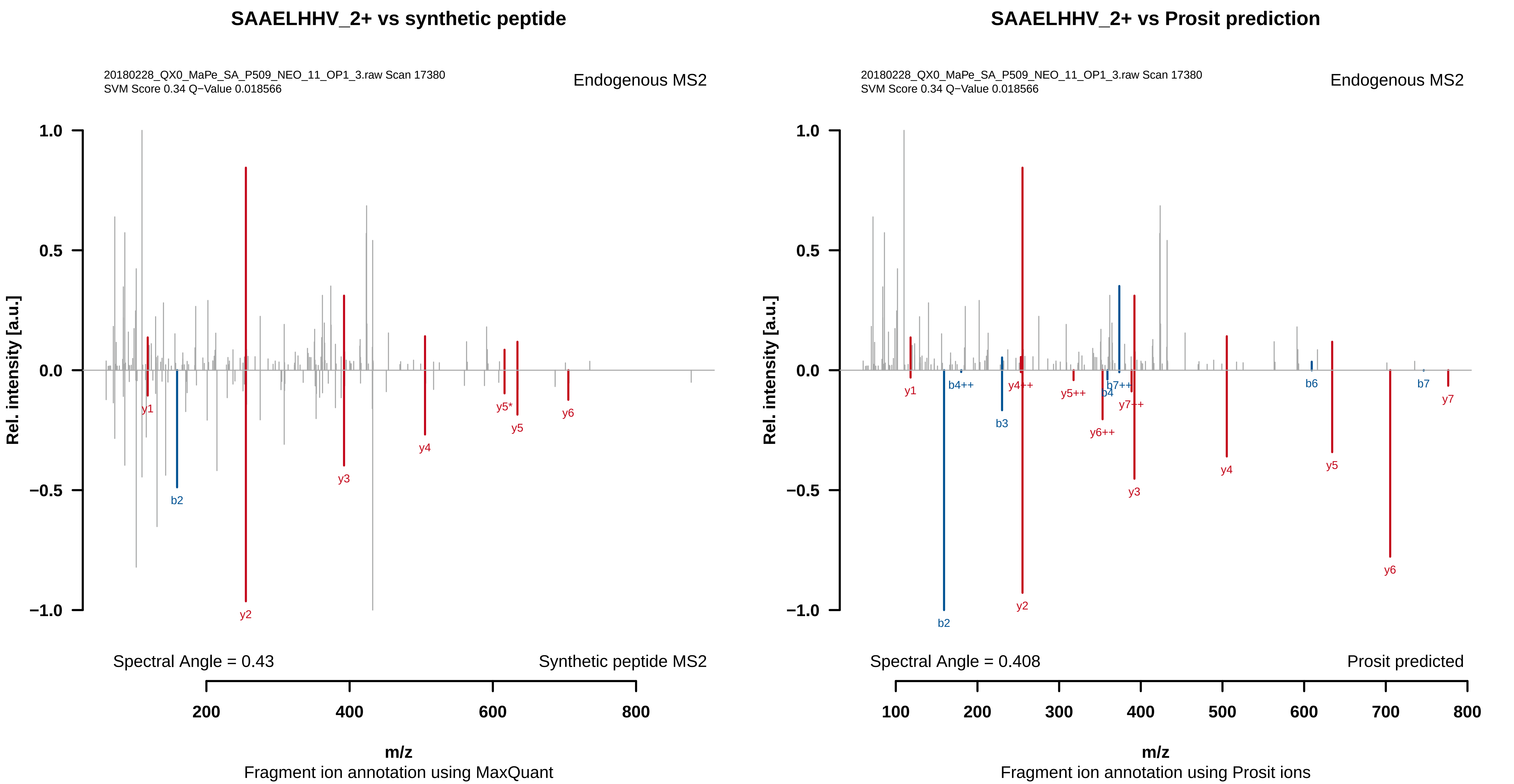
SAAELHHV_2+ vs synthetic peptide
20180228_QX0_MaPe_SA_P509_NEO_11_OP1_3.raw Scan 17380
SVM Score 0.34 Q−Value 0.018566
Endogenous MS2
Rel. intensity [a.u.]
1.0
0.5
0.0
−0.5
−1.0
y1
b2
y2
y3
y4
y5*
y5
y6
Spectral Angle = 0.43
Synthetic peptide MS2
200
400
600
800
m/z
Fragment ion annotation using MaxQuant
SAAELHHV_2+ vs Prosit prediction
20180228_QX0_MaPe_SA_P509_NEO_11_OP1_3.raw Scan 17380
SVM Score 0.34 Q−Value 0.018566
Endogenous MS2
Rel. intensity [a.u.]
1.0
0.5
0.0
−0.5
−1.0
y1
b4++
y4++
y5++
b4
b7++
y7++
y6++
b3
b2
y2
y3
y4
b6
y5
y6
b7
y7
Spectral Angle = 0.408
Prosit predicted
100
200
300
400
500
600
700
800
m/z
Fragment ion annotation using Prosit ions

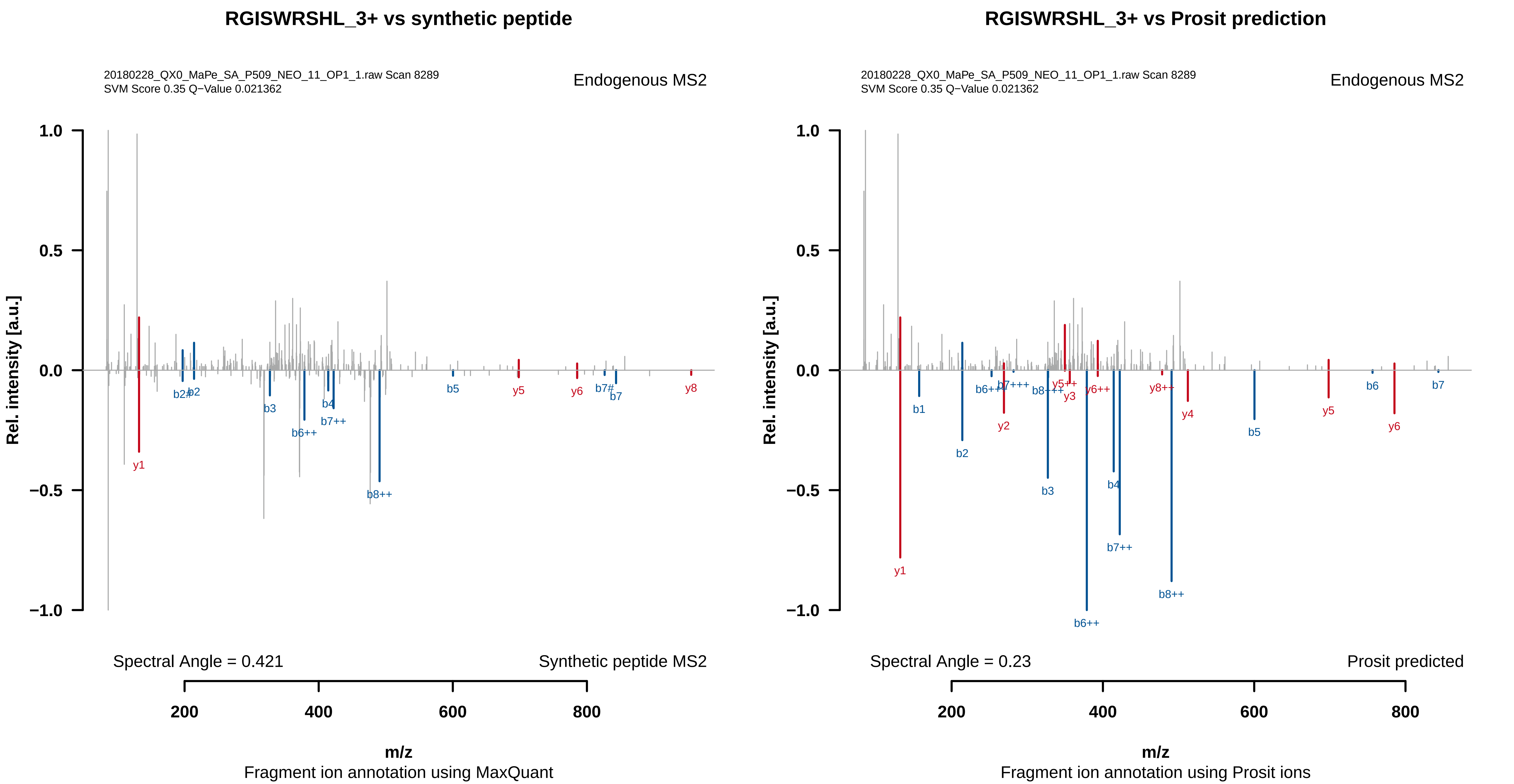
RGISWRSHL_3+ vs synthetic peptide
20180228_QX0_MaPe_SA_P509_NEO_11_OP1_1.raw Scan 8289
SVM Score 0.35 Q−Value 0.021362
Endogenous MS2
Rel. intensity [a.u.]
1.0
0.5
0.0
−0.5
−1.0
y1
b2#
b2
b3
b6++
b4
b7++
b8++
b5
y5
y6
b7#
b7
y8
Spectral Angle = 0.421
Synthetic peptide MS2
200
400
600
800
m/z
Fragment ion annotation using MaxQuant
RGISWRSHL_3+ vs Prosit prediction
20180228_QX0_MaPe_SA_P509_NEO_11_OP1_1.raw Scan 8289
SVM Score 0.35 Q−Value 0.021362
Endogenous MS2
Rel. intensity [a.u.]
1.0
0.5
0.0
−0.5
−1.0
y1
b1
b2
b6+++
b7+++
y2
b8+++
y5++
y3
b3
y6++
b6++
b4
b7++
y8++
b8++
y4
b5
y5
b6
y6
b7
Spectral Angle = 0.23
Prosit predicted
200
400
600
800
m/z
Fragment ion annotation using Prosit ions

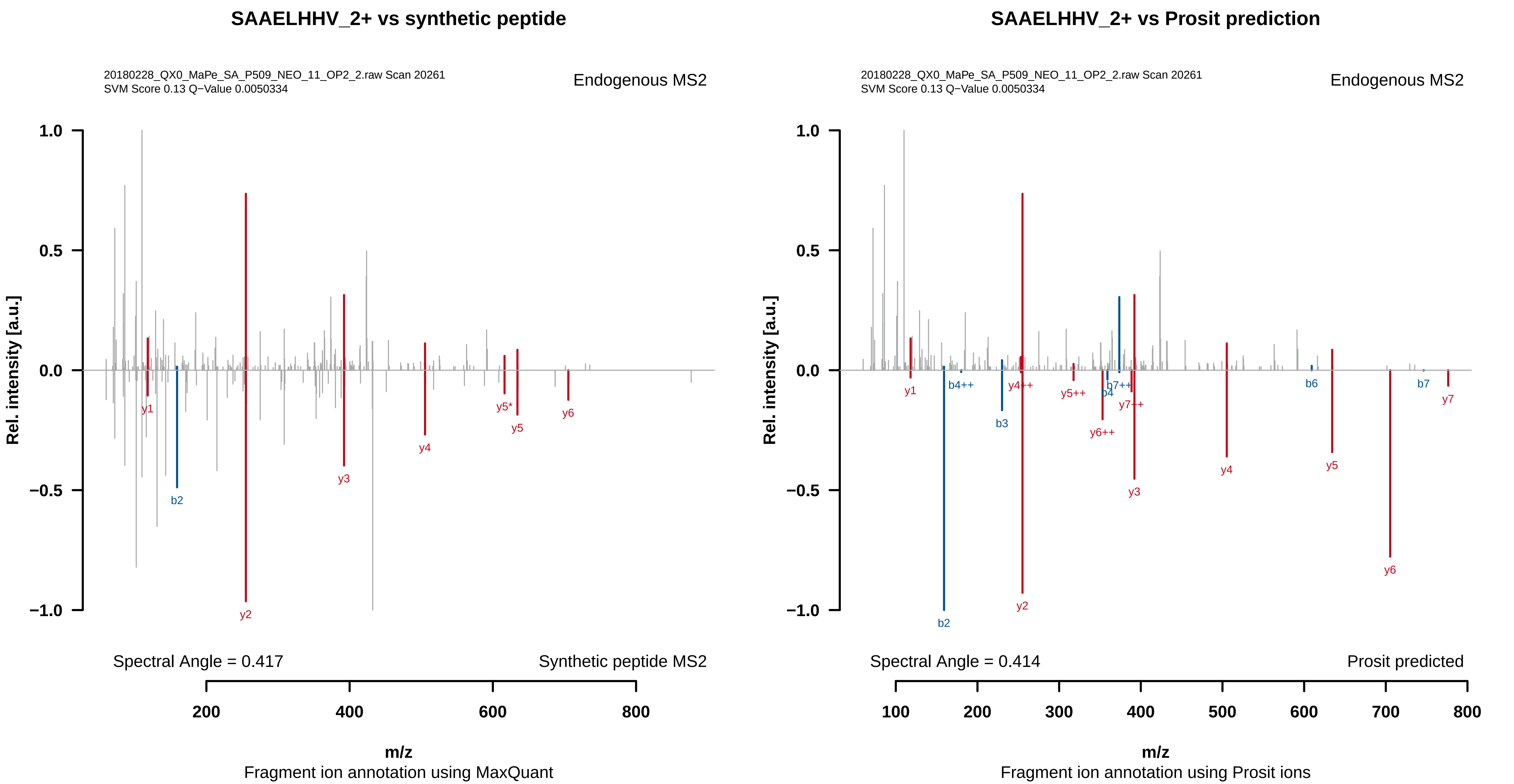
SAAELHHV_2+ vs synthetic peptide
20180228_QX0_MaPe_SA_P509_NEO_11_OP2_2.raw Scan 20261
SVM Score 0.13 Q−Value 0.0050334
Endogenous MS2
Rel. intensity [a.u.]
1.0
0.5
0.0
−0.5
−1.0
y1
b2
y2
y3
y4
y5*
y5
y6
Spectral Angle = 0.417
Synthetic peptide MS2
200
400
600
800
m/z
Fragment ion annotation using MaxQuant
SAAELHHV_2+ vs Prosit prediction
20180228_QX0_MaPe_SA_P509_NEO_11_OP2_2.raw Scan 20261
SVM Score 0.13 Q−Value 0.0050334
Endogenous MS2
Rel. intensity [a.u.]
1.0
0.5
0.0
−0.5
−1.0
y1
b4++
y4++
y5++
b4
b7++
y7++
y6++
b3
b2
y2
y3
y4
b6
y5
y6
b7
y7
Spectral Angle = 0.414
Prosit predicted
100
200
300
400
500
600
700
800
m/z
Fragment ion annotation using Prosit ions

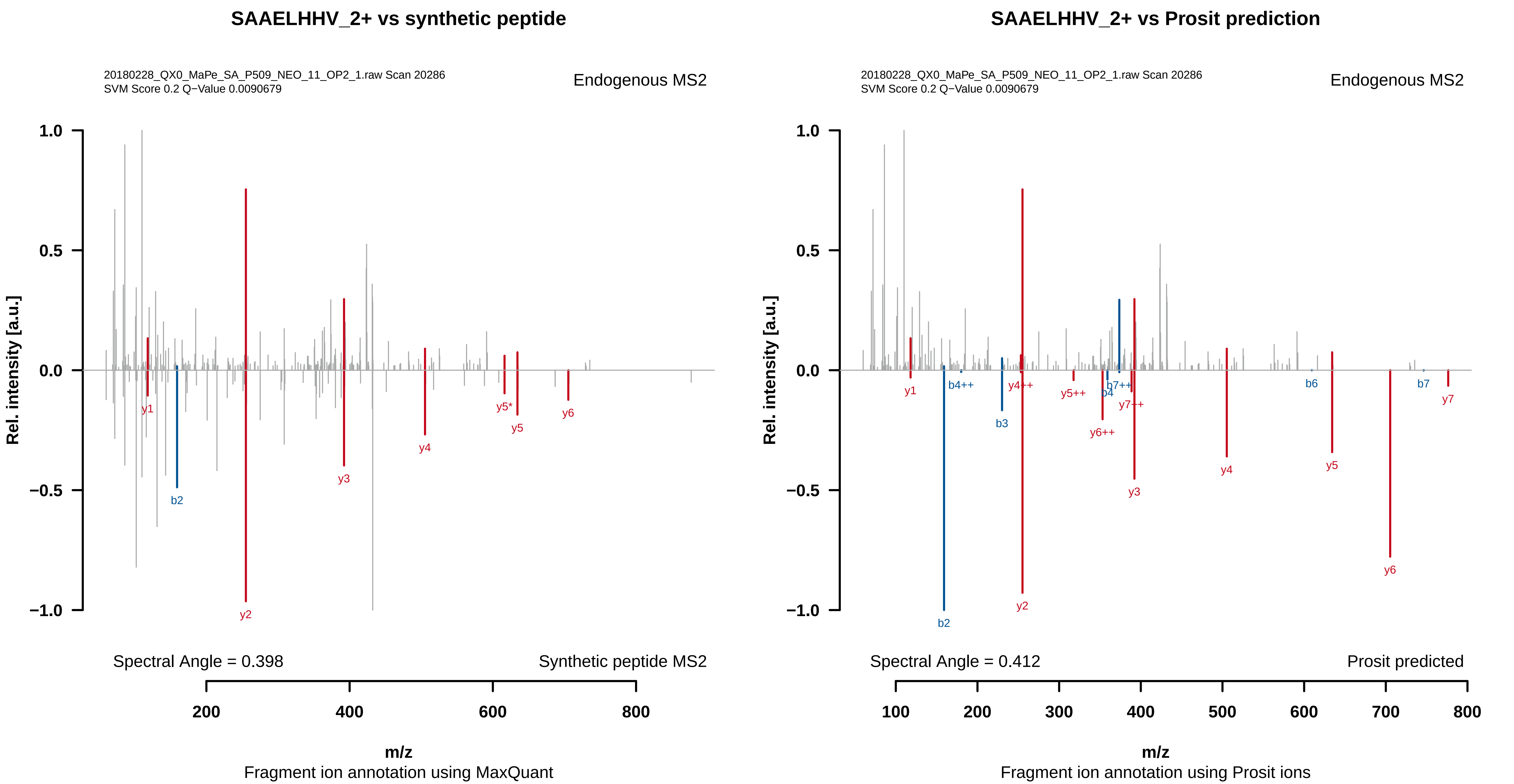

SAAELHHV_2+ vs synthetic peptide
20180228_QX0_MaPe_SA_P509_NEO_11_OP2_1.raw Scan 20286
SVM Score 0.2 Q−Value 0.0090679
Endogenous MS2
Rel. intensity [a.u.]
1.0
0.5
0.0
−0.5
−1.0
y1
b2
y2
y3
y4
y5*
y5
y6
Spectral Angle = 0.398
Synthetic peptide MS2
200
400
600
800
m/z
Fragment ion annotation using MaxQuant
SAAELHHV_2+ vs Prosit prediction
20180228_QX0_MaPe_SA_P509_NEO_11_OP2_1.raw Scan 20286
SVM Score 0.2 Q−Value 0.0090679
Endogenous MS2
Rel. intensity [a.u.]
y1
b4++
y4++
y5++
b7++
b4
y7++
y6++
b3
b6
b7
y7
y4
y5
y3
y6
b2
y2
Spectral Angle = 0.412
Prosit predicted
100
200
300
400
500
600
700
800
m/z
Fragment ion annotation using Prosit ions

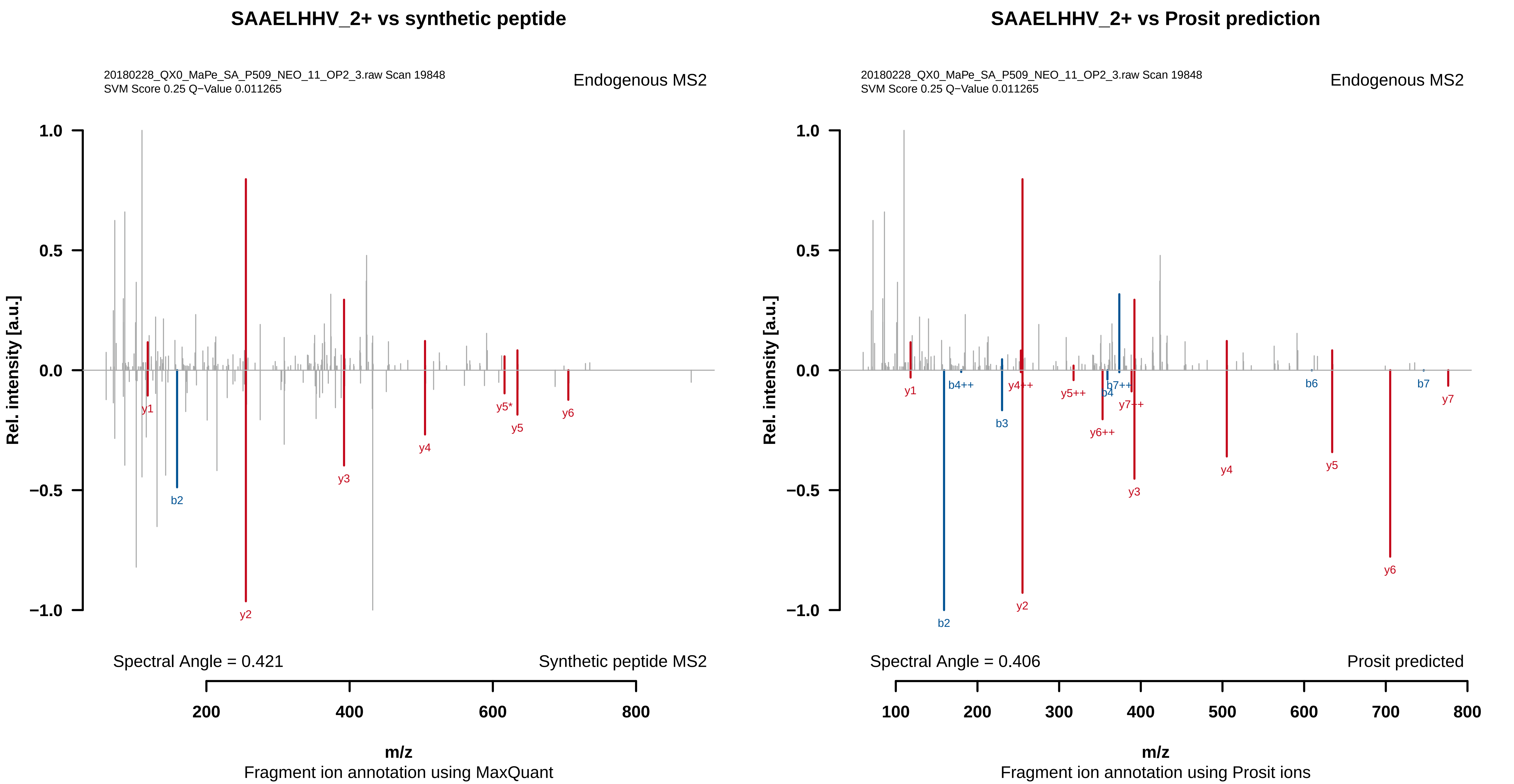

SAAELHHV_2+ vs synthetic peptide
20180228_QX0_MaPe_SA_P509_NEO_11_OP2_3.raw Scan 19848
SVM Score 0.25 Q−Value 0.011265
Endogenous MS2
Rel. intensity [a.u.]
1.0
0.5
0.0
−0.5
−1.0
y1
b2
y2
y3
y4
y5*
y5
y6
Spectral Angle = 0.421
Synthetic peptide MS2
200
400
600
800
m/z
Fragment ion annotation using MaxQuant
SAAELHHV_2+ vs Prosit prediction
20180228_QX0_MaPe_SA_P509_NEO_11_OP2_3.raw Scan 19848
SVM Score 0.25 Q−Value 0.011265
Endogenous MS2
Rel. intensity [a.u.]
1.0
0.5
0.0
−0.5
−1.0
y1
b4++
y4++
y5++
b4
b7++
y7++
y6++
b3
b2
y2
y3
y4
b6
y5
y6
b7
y7
Spectral Angle = 0.406
Prosit predicted
100
200
300
400
500
600
700
800
m/z
Fragment ion annotation using Prosit ions

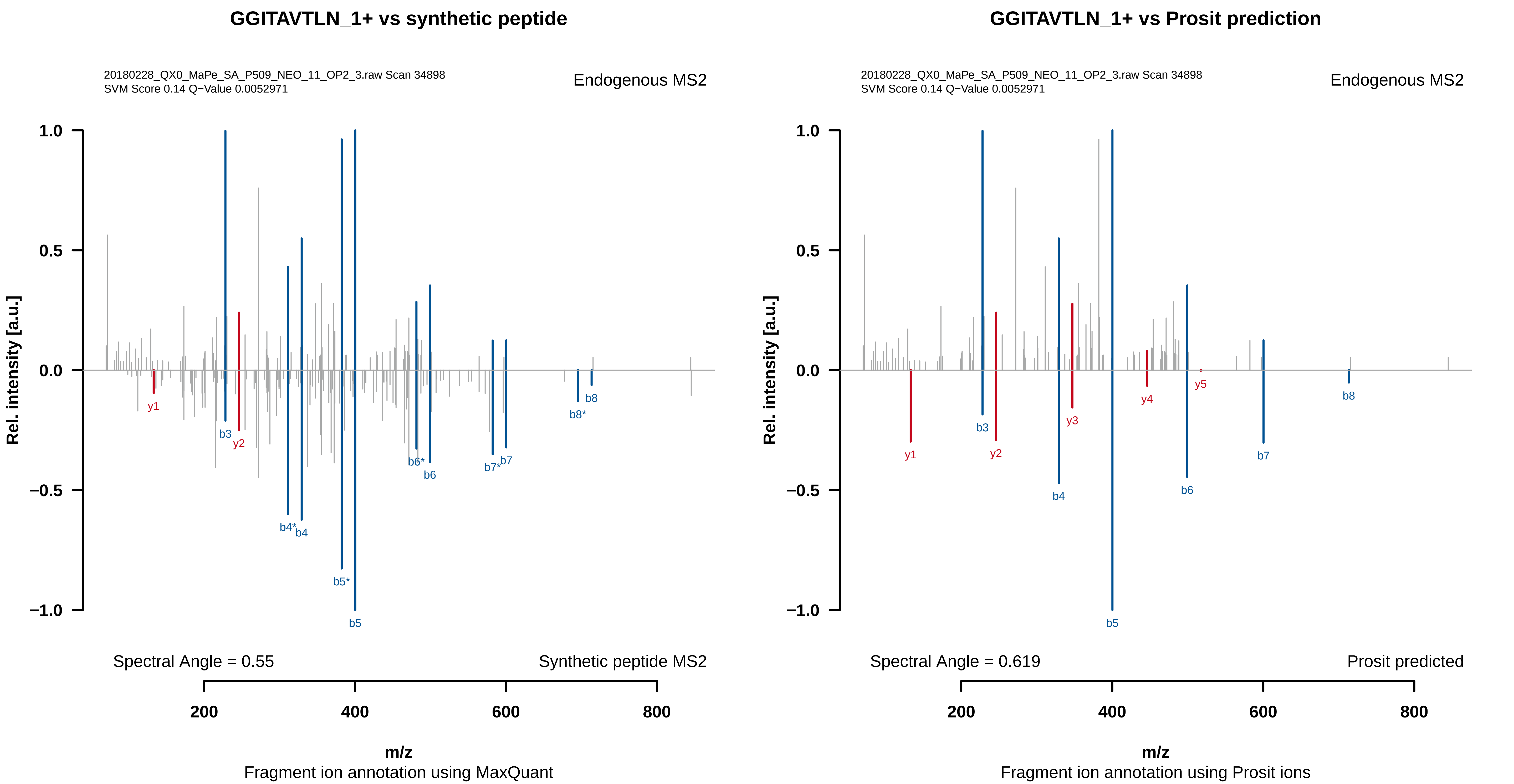
GGITAVTLN_1+ vs synthetic peptide
20180228_QX0_MaPe_SA_P509_NEO_11_OP2_3.raw Scan 34898
SVM Score 0.14 Q−Value 0.0052971
Endogenous MS2
Rel. intensity [a.u.]
1.0
0.5
0.0
−0.5
−1.0
y1
b3
y2
b4*
b4
b5*
b5
b6*
b6
b7*
b7
b8*
b8
Spectral Angle = 0.55
Synthetic peptide MS2
200
400
600
800
m/z
Fragment ion annotation using MaxQuant
GGITAVTLN_1+ vs Prosit prediction
20180228_QX0_MaPe_SA_P509_NEO_11_OP2_3.raw Scan 34898
SVM Score 0.14 Q−Value 0.0052971
Endogenous MS2
Rel. intensity [a.u.]
1.0
0.5
0.0
−0.5
−1.0
y1
b3
y2
b4
y3
b5
y4
y5
b6
b7
b8
Spectral Angle = 0.619
Prosit predicted
200
400
600
800
m/z
Fragment ion annotation using Prosit ions

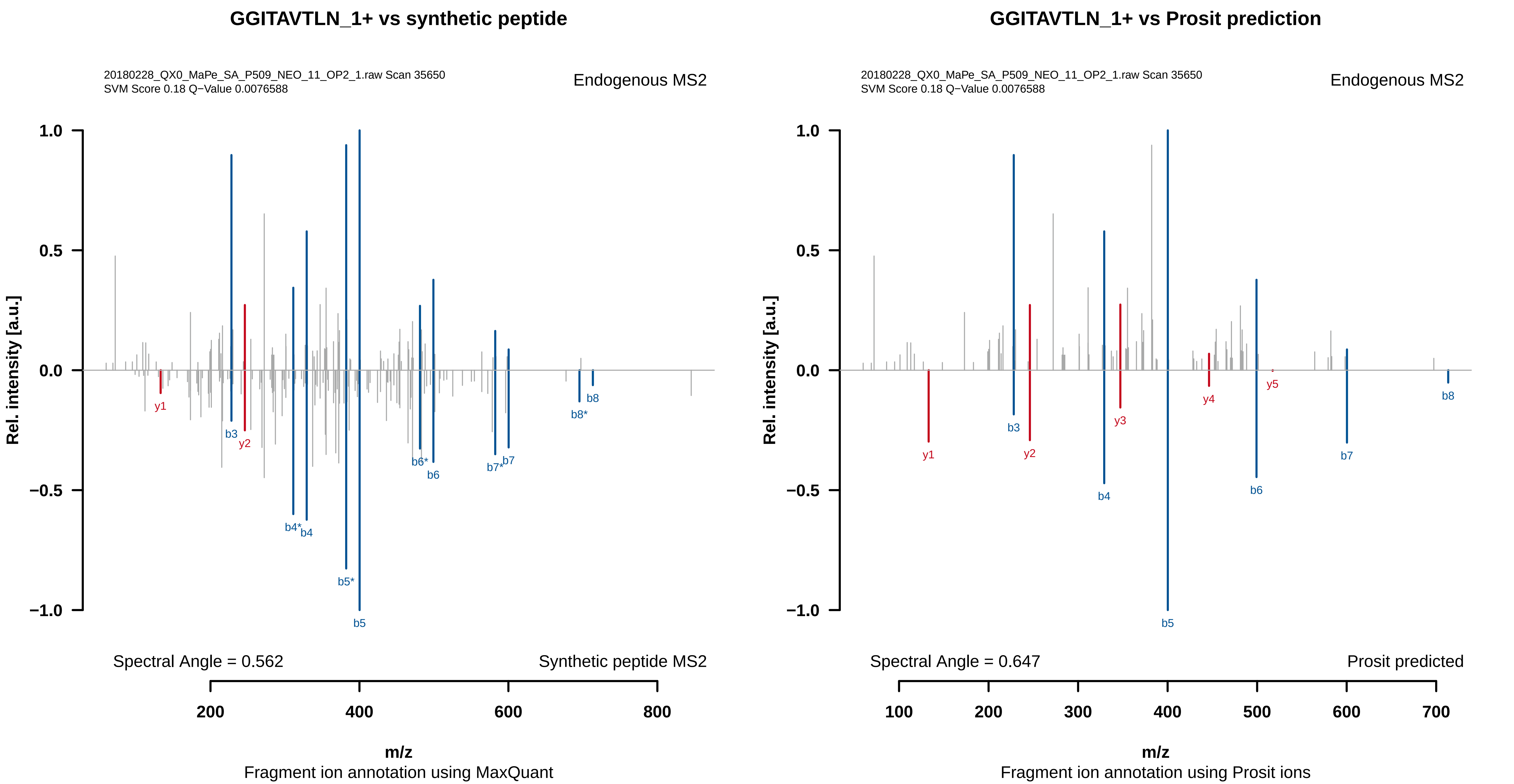

GGITAVTLN_1+ vs synthetic peptide
20180228_QX0_MaPe_SA_P509_NEO_11_OP2_1.raw Scan 35650
SVM Score 0.18 Q−Value 0.0076588
Endogenous MS2
Rel. intensity [a.u.]
1.0
0.5
0.0
−0.5
−1.0
y1
b3
y2
b4*
b4
b5*
b5
b6*
b6
b7*
b7
b8*
b8
Spectral Angle = 0.562
Synthetic peptide MS2
200
400
600
800
m/z
Fragment ion annotation using MaxQuant
GGITAVTLN_1+ vs Prosit prediction
20180228_QX0_MaPe_SA_P509_NEO_11_OP2_1.raw Scan 35650
SVM Score 0.18 Q−Value 0.0076588
Endogenous MS2
Rel. intensity [a.u.]
y1
b3
y2
y3
b4
b5
y4
y5
b6
b7
b8
Spectral Angle = 0.647
Prosit predicted
100
200
300
400
500
600
700
m/z
Fragment ion annotation using Prosit ions

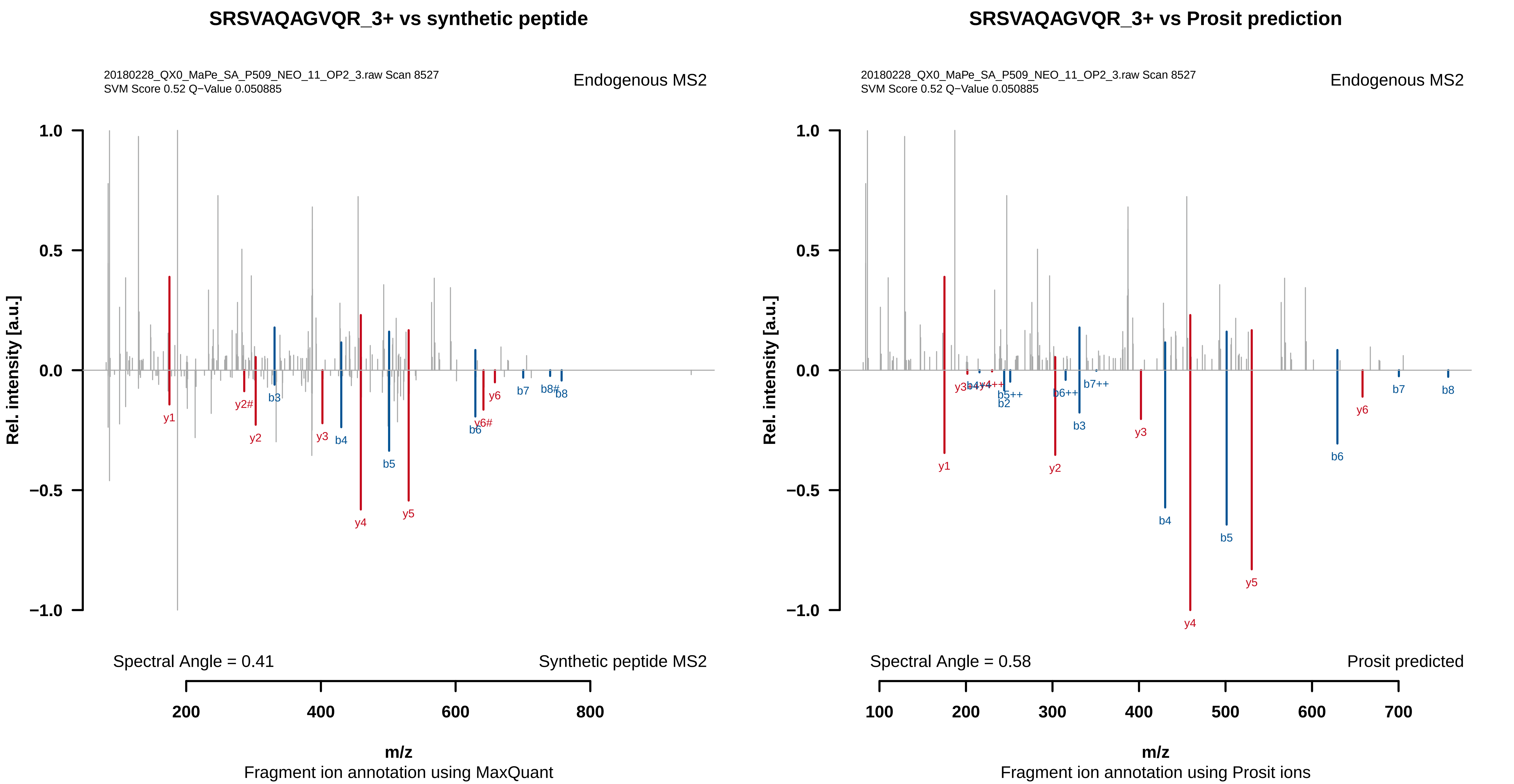

SRSVAQAGVQR_3+ vs synthetic peptide
20180228_QX0_MaPe_SA_P509_NEO_11_OP2_3.raw Scan 8527
SVM Score 0.52 Q−Value 0.050885
Endogenous MS2
Rel. intensity [a.u.]
1.0
0.5
0.0
−0.5
−1.0
y1
y2#
y2
b3
y3
b4
y4
b5
y5
b6
y6#
y6
b7
b8#
b8
Spectral Angle = 0.41
Synthetic peptide MS2
200
400
600
800
m/z
Fragment ion annotation using MaxQuant
SRSVAQAGVQR_3+ vs Prosit prediction
20180228_QX0_MaPe_SA_P509_NEO_11_OP2_3.raw Scan 8527
SVM Score 0.52 Q−Value 0.050885
Endogenous MS2
Rel. intensity [a.u.]
1.0
0.5
0.0
−0.5
−1.0
y1
b5++
b2
b6++
b7++
y2
b3
y3
b4
y4
b5
y5
b6
y6
b7
b8
Spectral Angle = 0.58
Prosit predicted
100
200
300
400
500
600
700
m/z
Fragment ion annotation using Prosit ions

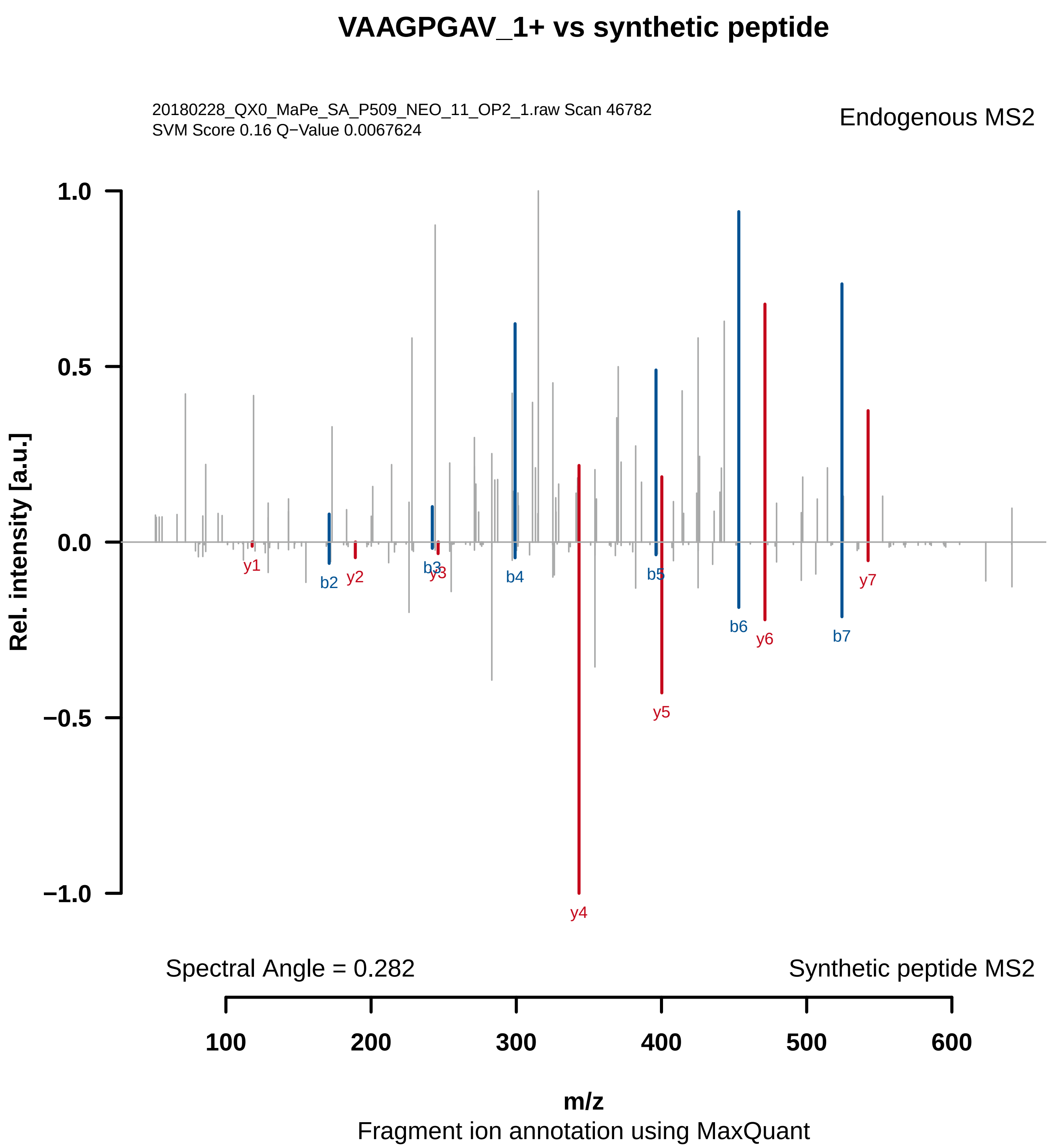

VAAGPGAV_1+ vs synthetic peptide
20180228_QX0_MaPe_SA_P509_NEO_11_OP2_1.raw Scan 46782
SVM Score 0.16 Q−Value 0.0067624
Endogenous MS2
Rel. intensity [a.u.]
1.0
0.5
0.0
−0.5
−1.0
y1
b2
y2
b3
y3
b4
b5
y4
y5
b6
y6
b7
y7
Spectral Angle = 0.282
Synthetic peptide MS2
100
200
300
400
500
600
m/z
Fragment ion annotation using MaxQuant

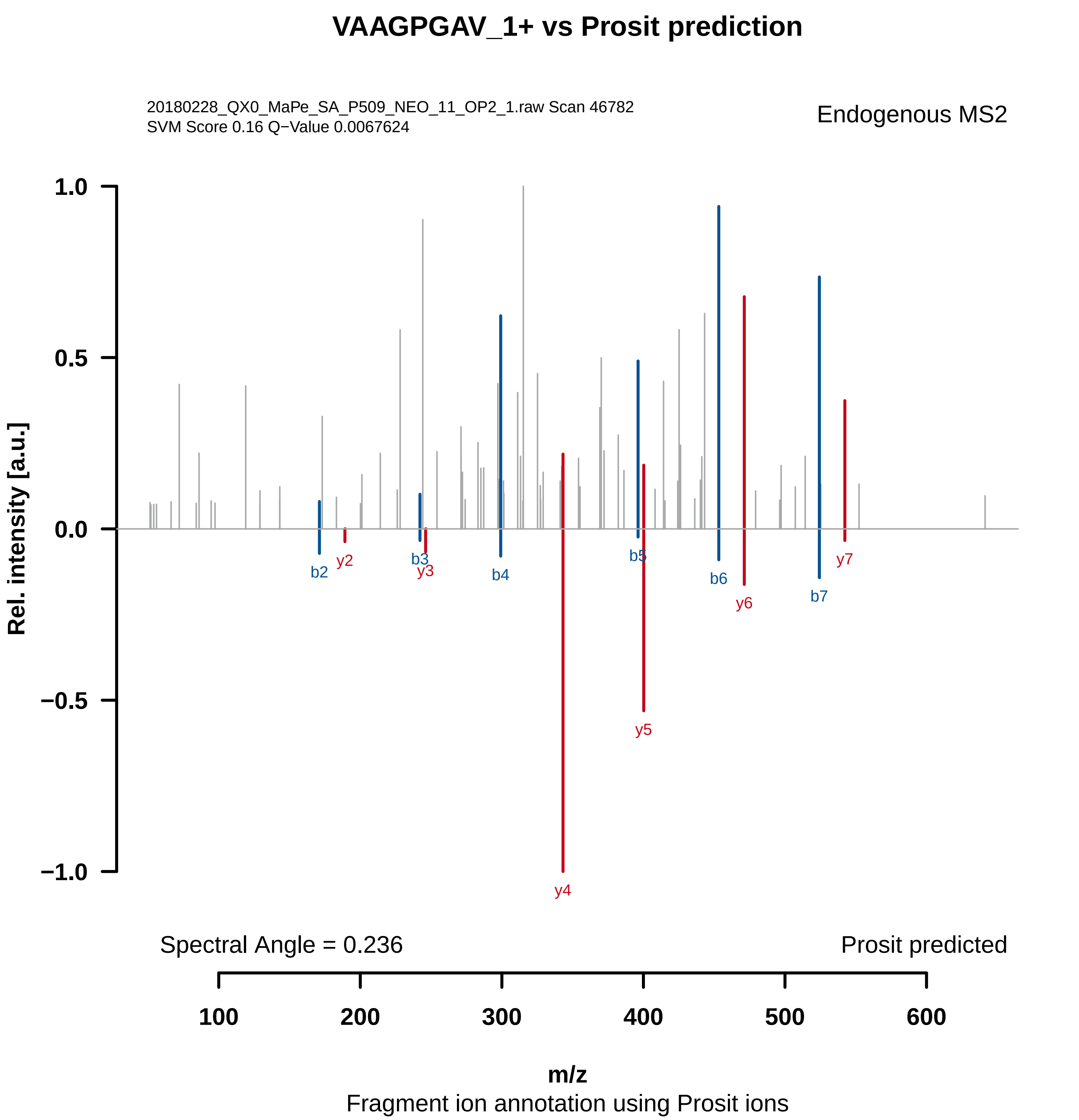

VAAGPGAV_1+ vs Prosit prediction
20180228_QX0_MaPe_SA_P509_NEO_11_OP2_1.raw Scan 46782
SVM Score 0.16 Q−Value 0.0067624
Endogenous MS2
Rel. intensity [a.u.]
1.0
0.5
0.0
−0.5
−1.0
b2
y2
b3
y3
b4
b5
y4
y5
b6
y6
b7
y7
Spectral Angle = 0.236
Prosit predicted
100
200
300
400
500
600
m/z
Fragment ion annotation using Prosit ions

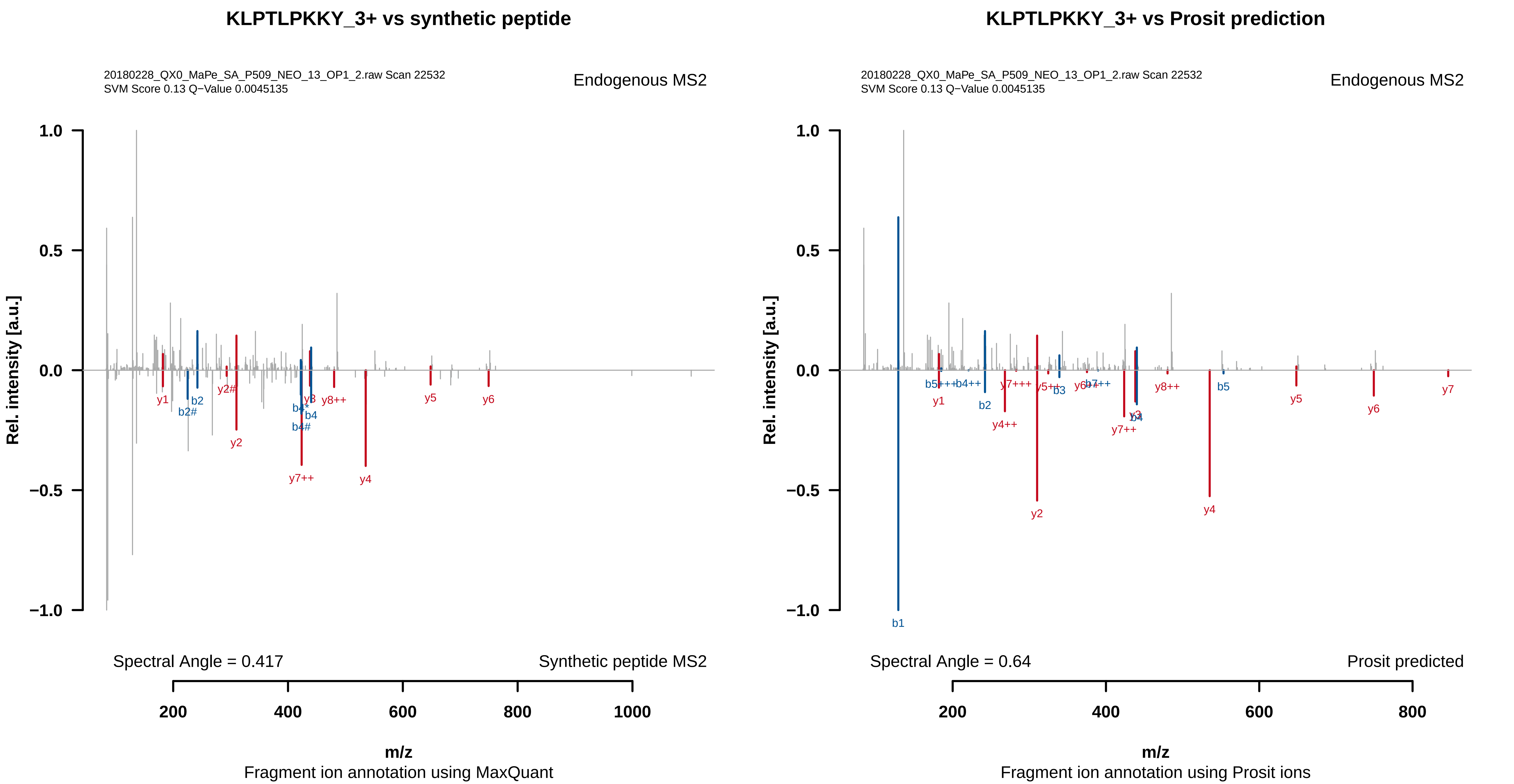

KLPTLPKKY_3+ vs synthetic peptide
20180228_QX0_MaPe_SA_P509_NEO_13_OP1_2.raw Scan 22532
SVM Score 0.13 Q−Value 0.0045135
Endogenous MS2
Rel. intensity [a.u.]
1.0
0.5
0.0
−0.5
−1.0
y1
b2
b2#
y2#
y2
b4*
y3
b4
b4#
y8++
y7++
y4
y5
y6
Spectral Angle = 0.417
Synthetic peptide MS2
200
400
600
800
1000
m/z
Fragment ion annotation using MaxQuant
KLPTLPKKY_3+ vs Prosit prediction
20180228_QX0_MaPe_SA_P509_NEO_13_OP1_2.raw Scan 22532
SVM Score 0.13 Q−Value 0.0045135
Endogenous MS2
Rel. intensity [a.u.]
1.0
0.5
0.0
−0.5
−1.0
b5+++b4++
y1
b2
y7+++
y5++
b3
y6++b7++
y4++
y2
y7++
y3
b4
y8++
b5
y4
y5
y6
y7
b1
Spectral Angle = 0.64
Prosit predicted
200
400
600
800
m/z
Fragment ion annotation using Prosit ions

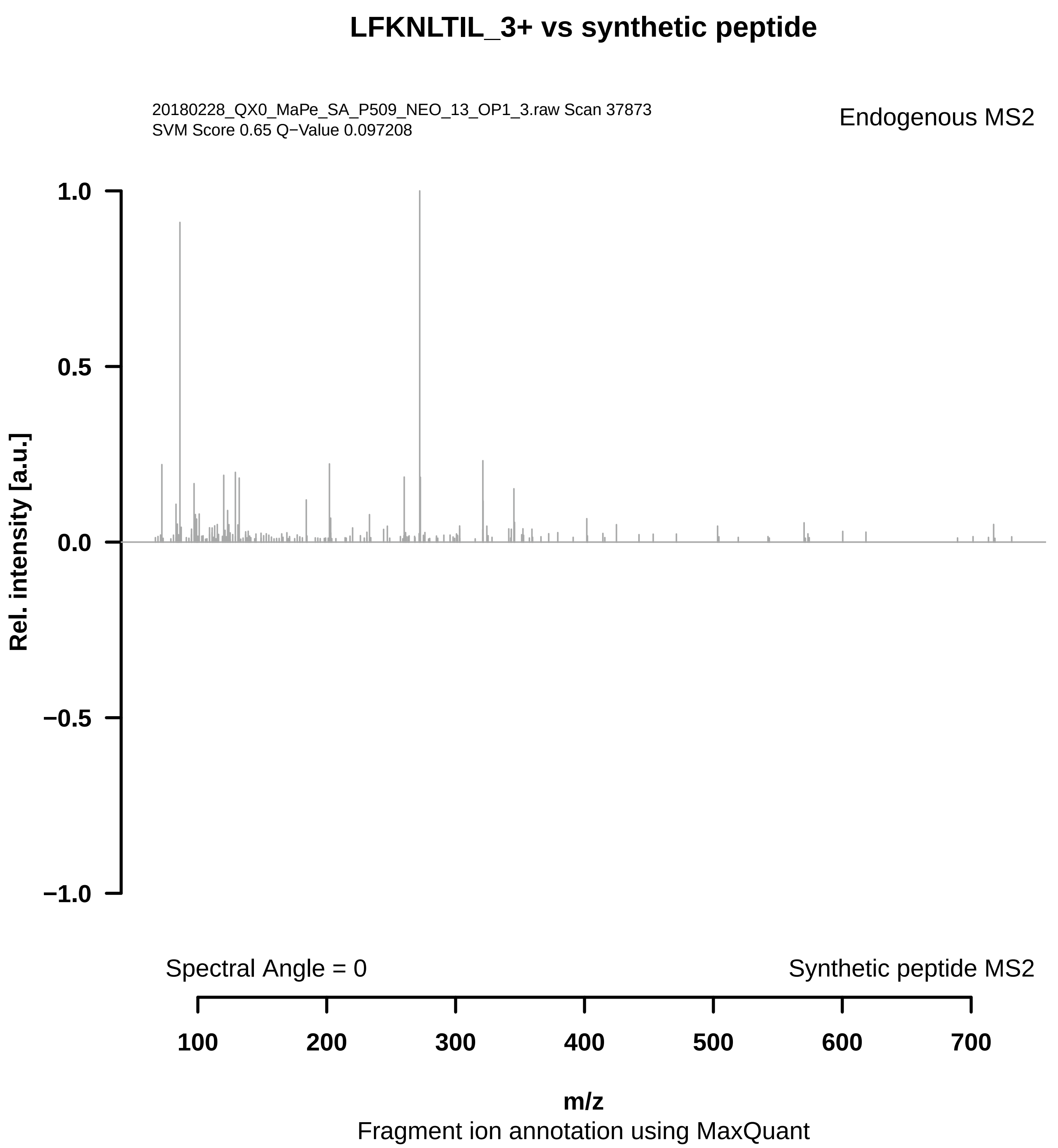
LFKNLTIL_3+ vs synthetic peptide
20180228_QX0_MaPe_SA_P509_NEO_13_OP1_3.raw Scan 37873
SVM Score 0.65 Q−Value 0.097208
Endogenous MS2
Rel. intensity [a.u.]
1.0
0.5
0.0
−0.5
−1.0
Spectral Angle = 0
Synthetic peptide MS2
100
200
300
400
500
600
700
m/z
Fragment ion annotation using MaxQuant

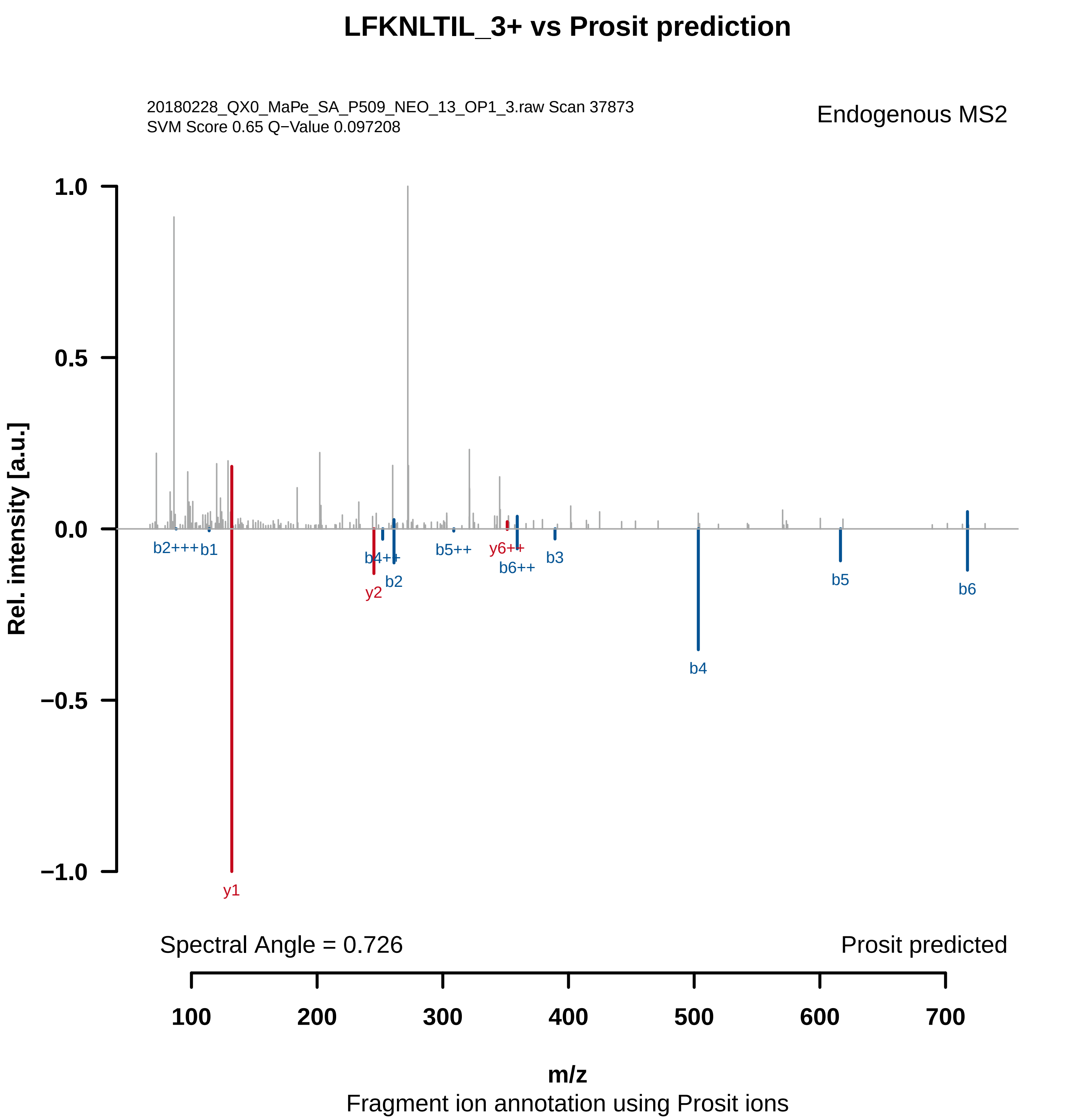
LFKNLTIL_3+ vs Prosit prediction
20180228_QX0_MaPe_SA_P509_NEO_13_OP1_3.raw Scan 37873
SVM Score 0.65 Q−Value 0.097208
Endogenous MS2
Rel. intensity [a.u.]
1.0
0.5
0.0
−0.5
−1.0
b2+++
b1
b4++
b2
y2
b5++
y6++
b6++
b3
b4
b5
b6
y1
Spectral Angle = 0.726
Prosit predicted
100
200
300
400
500
600
700
m/z
Fragment ion annotation using Prosit ions

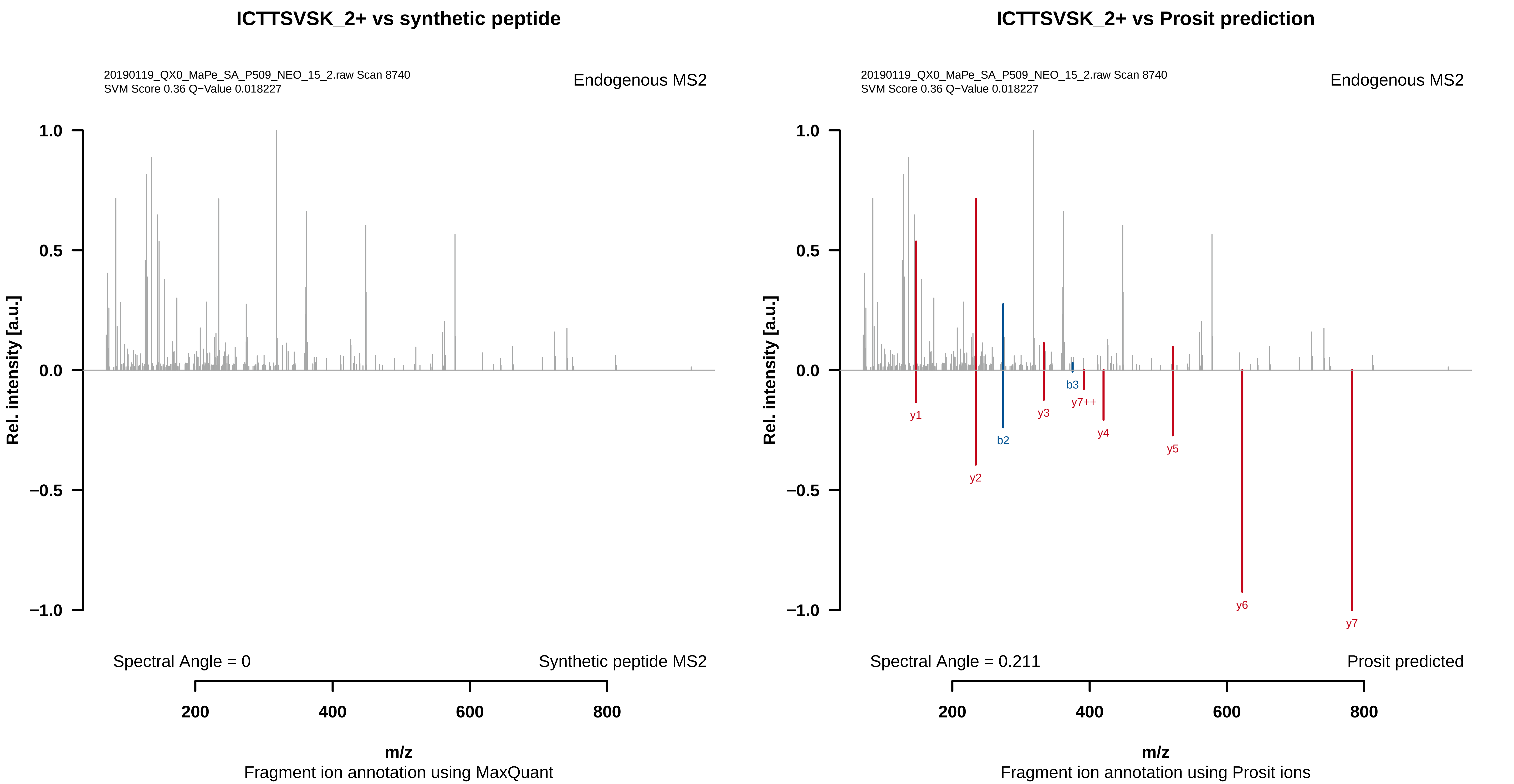

ICTTSVSK_2+ vs synthetic peptide
20190119_QX0_MaPe_SA_P509_NEO_15_2.raw Scan 8740
SVM Score 0.36 Q−Value 0.018227
Endogenous MS2
Rel. intensity [a.u.]
1.0
0.5
0.0
−0.5
−1.0
Spectral Angle = 0
Synthetic peptide MS2
200
400
600
800
m/z
Fragment ion annotation using MaxQuant
ICTTSVSK_2+ vs Prosit prediction
20190119_QX0_MaPe_SA_P509_NEO_15_2.raw Scan 8740
SVM Score 0.36 Q−Value 0.018227
Endogenous MS2
Rel. intensity [a.u.]
y1
y2
b2
y3
b3
y7++
y4
y5
y6
y7
Spectral Angle = 0.211
Prosit predicted
m/z
Fragment ion annotation using Prosit ions

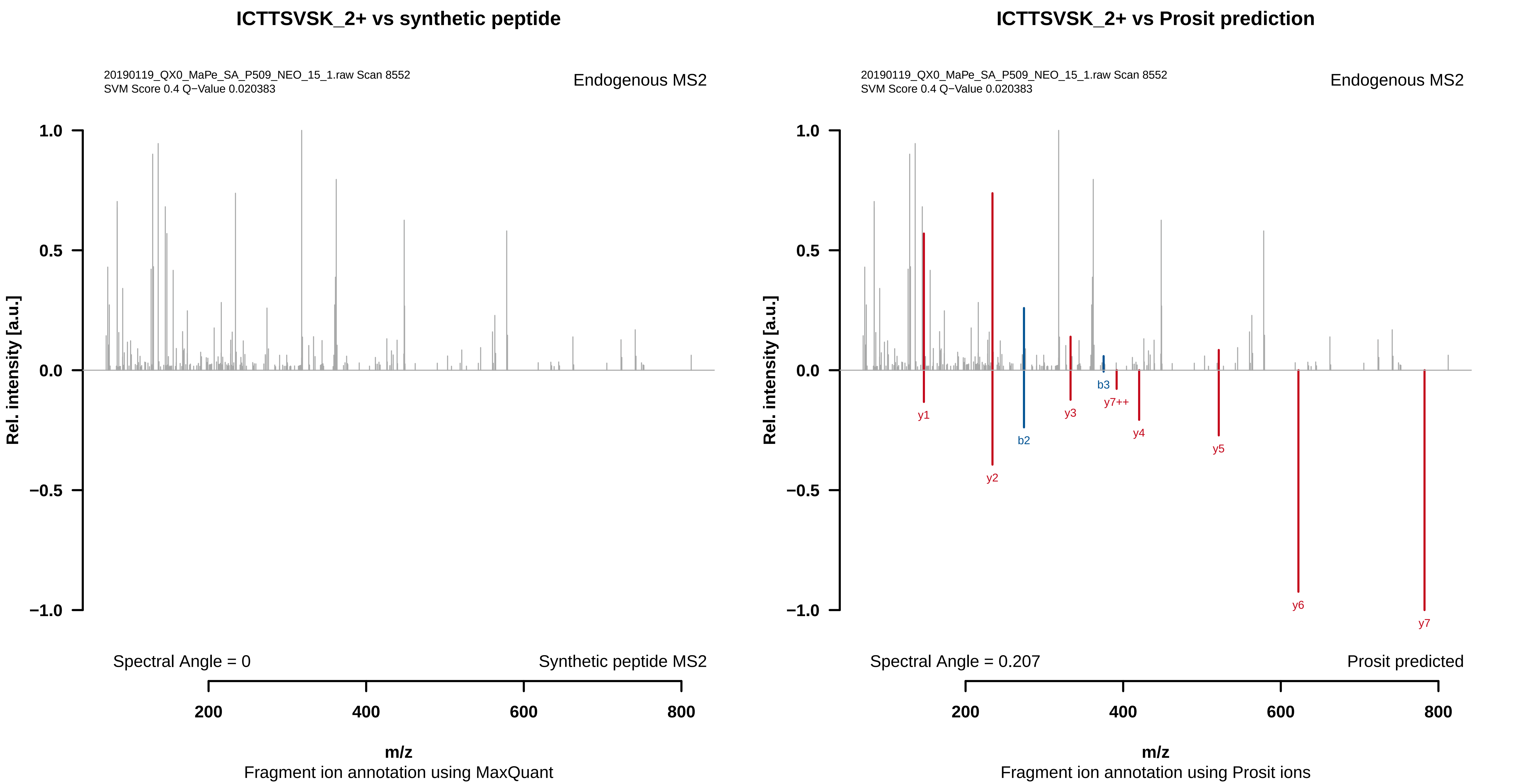
ICTTSVSK_2+ vs synthetic peptide
20190119_QX0_MaPe_SA_P509_NEO_15_1.raw Scan 8552
SVM Score 0.4 Q−Value 0.020383
Endogenous MS2
Rel. intensity [a.u.]
1.0
0.5
0.0
−0.5
−1.0
Spectral Angle = 0
Synthetic peptide MS2
200
400
600
800
m/z
Fragment ion annotation using MaxQuant
ICTTSVSK_2+ vs Prosit prediction
20190119_QX0_MaPe_SA_P509_NEO_15_1.raw Scan 8552
SVM Score 0.4 Q−Value 0.020383
Endogenous MS2
Rel. intensity [a.u.]
y1
y2
b2
y3
b3
y7++
y4
y5
y6
y7
Spectral Angle = 0.207
Prosit predicted
m/z
Fragment ion annotation using Prosit ions

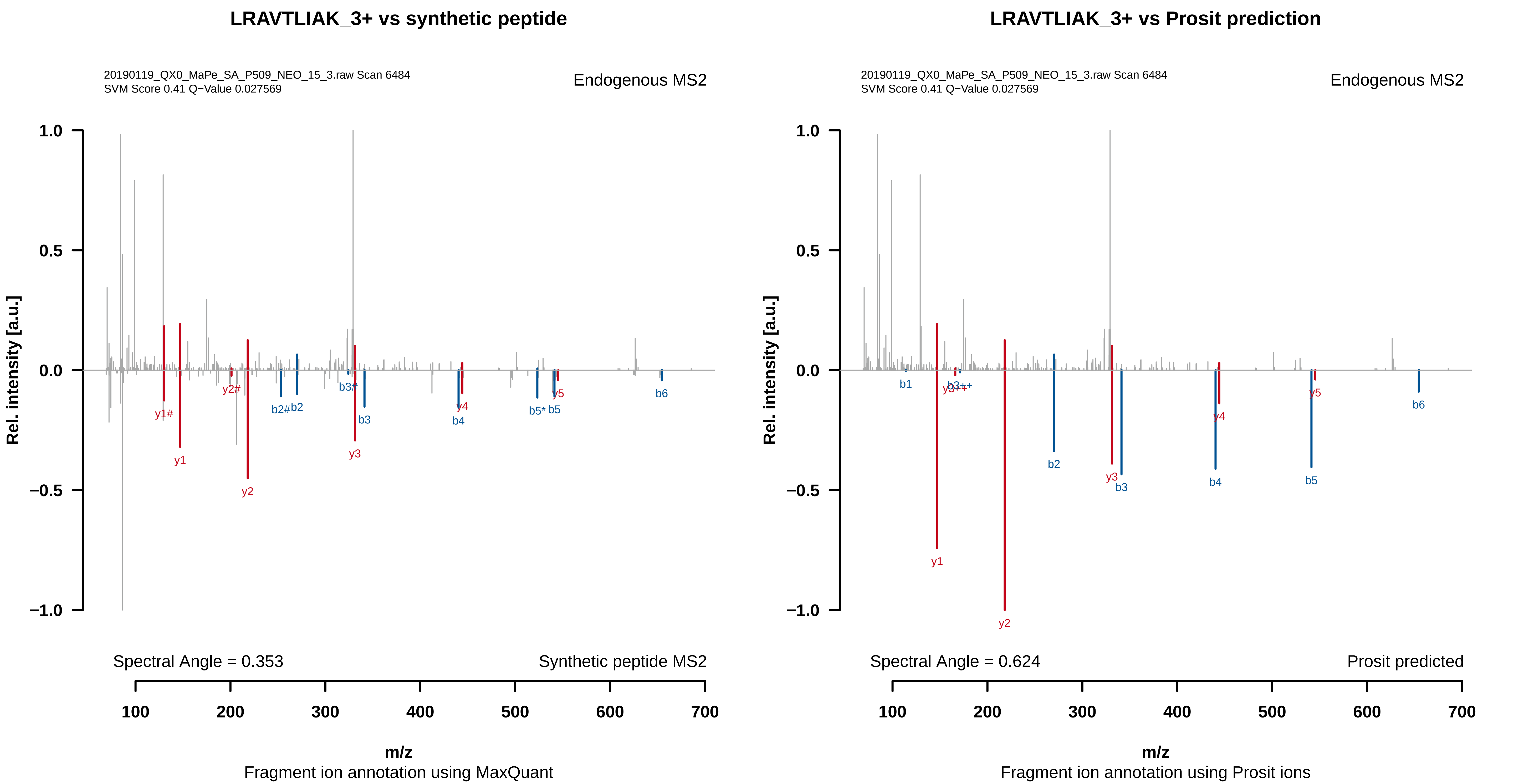

LRAVTLIAK_3+ vs synthetic peptide
20190119_QX0_MaPe_SA_P509_NEO_15_3.raw Scan 6484
SVM Score 0.41 Q−Value 0.027569
Endogenous MS2
Rel. intensity [a.u.]
Spectral Angle = 0.353
Synthetic peptide MS2
m/z
Fragment ion annotation using MaxQuant
LRAVTLIAK_3+ vs Prosit prediction
20190119_QX0_MaPe_SA_P509_NEO_15_3.raw Scan 6484
SVM Score 0.41 Q−Value 0.027569
Endogenous MS2
Rel. intensity [a.u.]
Spectral Angle = 0.624
Prosit predicted
m/z
Fragment ion annotation using Prosit ions

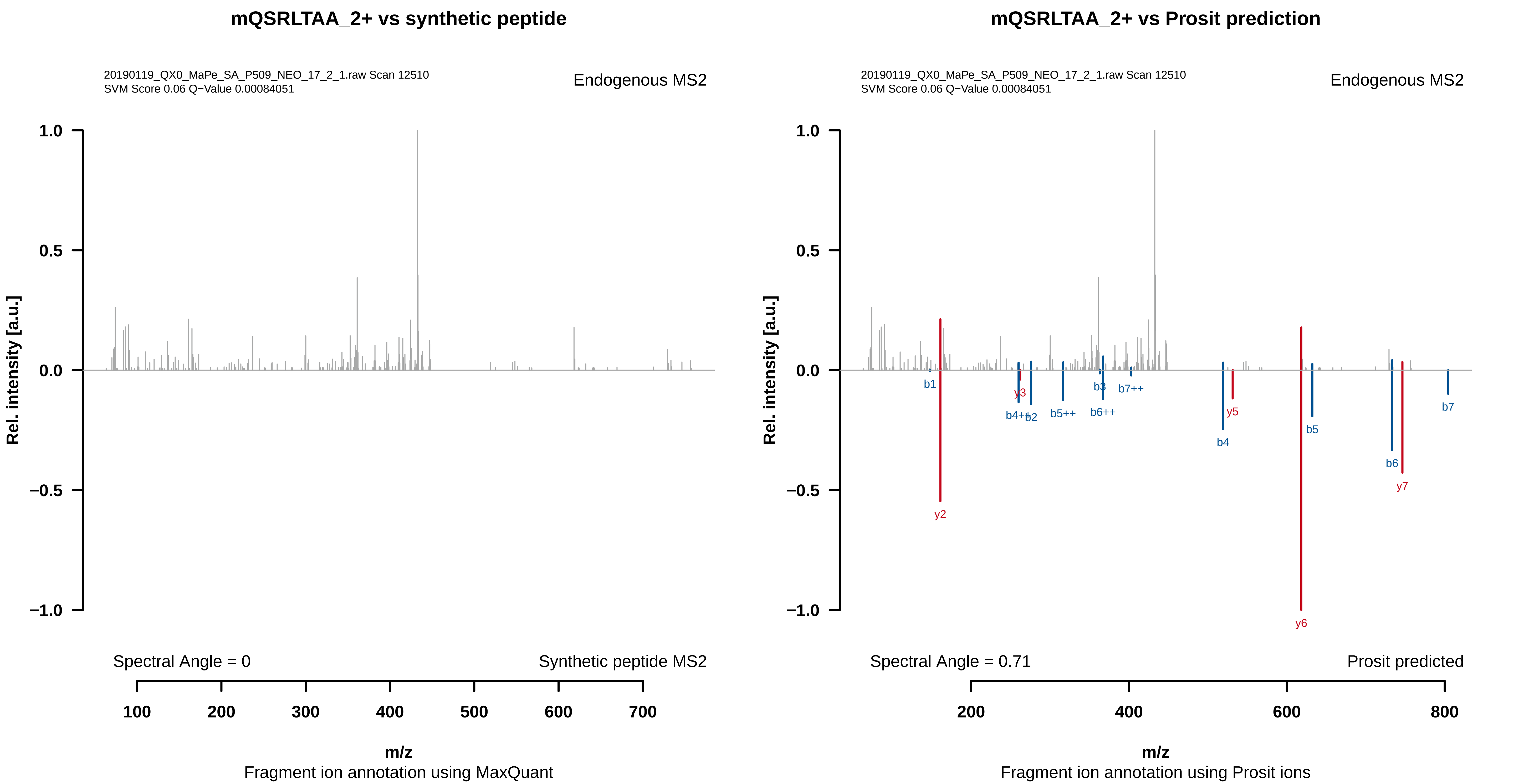

mQSRLTAA_2+ vs synthetic peptide
20190119_QX0_MaPe_SA_P509_NEO_17_2_1.raw Scan 12510
SVM Score 0.06 Q−Value 0.00084051
Endogenous MS2
Rel. intensity [a.u.]
1.0
0.5
0.0
−0.5
−1.0
Spectral Angle = 0
Synthetic peptide MS2
100
200
300
400
500
600
700
m/z
Fragment ion annotation using MaxQuant
mQSRLTAA_2+ vs Prosit prediction
20190119_QX0_MaPe_SA_P509_NEO_17_2_1.raw Scan 12510
SVM Score 0.06 Q−Value 0.00084051
Endogenous MS2
Rel. intensity [a.u.]
1.0
0.5
0.0
−0.5
−1.0
b1
y3
b4++
b2
b5++
b3
b6++
b7++
y2
y5
b4
b5
y6
b6
y7
b7
Spectral Angle = 0.71
Prosit predicted
200
400
600
800
m/z
Fragment ion annotation using Prosit ions

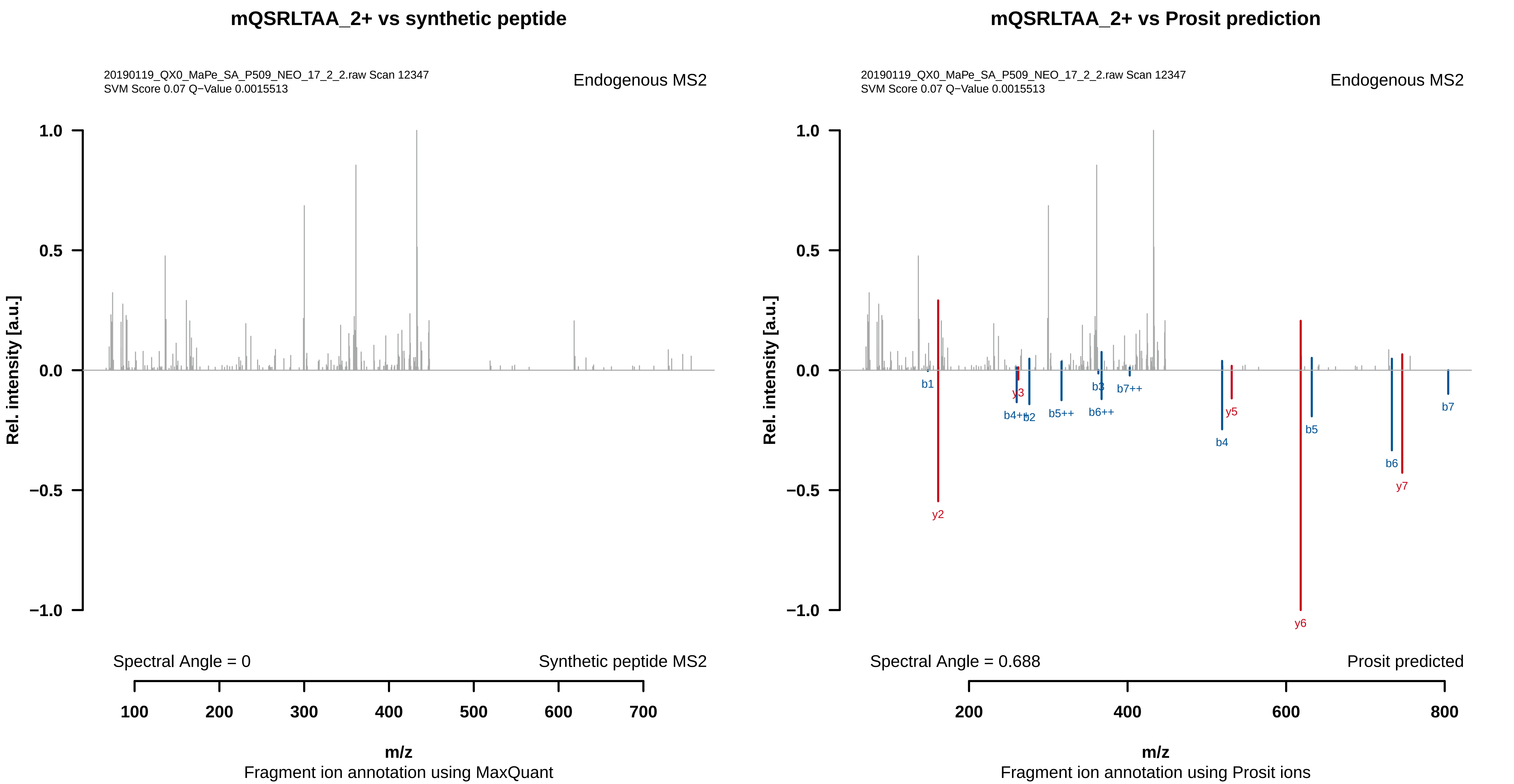

mQSRLTAA_2+ vs synthetic peptide
20190119_QX0_MaPe_SA_P509_NEO_17_2_2.raw Scan 12347
SVM Score 0.07 Q−Value 0.0015513
Endogenous MS2
Rel. intensity [a.u.]
1.0
0.5
0.0
−0.5
−1.0
Spectral Angle = 0
Synthetic peptide MS2
100
200
300
400
500
600
700
m/z
Fragment ion annotation using MaxQuant
mQSRLTAA_2+ vs Prosit prediction
20190119_QX0_MaPe_SA_P509_NEO_17_2_2.raw Scan 12347
SVM Score 0.07 Q−Value 0.0015513
Endogenous MS2
Rel. intensity [a.u.]
b1
y2
y3
b4++
b2
b5++
b3
b6++
b7++
b4
y5
y6
b5
b6
y7
b7
Spectral Angle = 0.688
Prosit predicted
200
400
600
800
m/z
Fragment ion annotation using Prosit ions

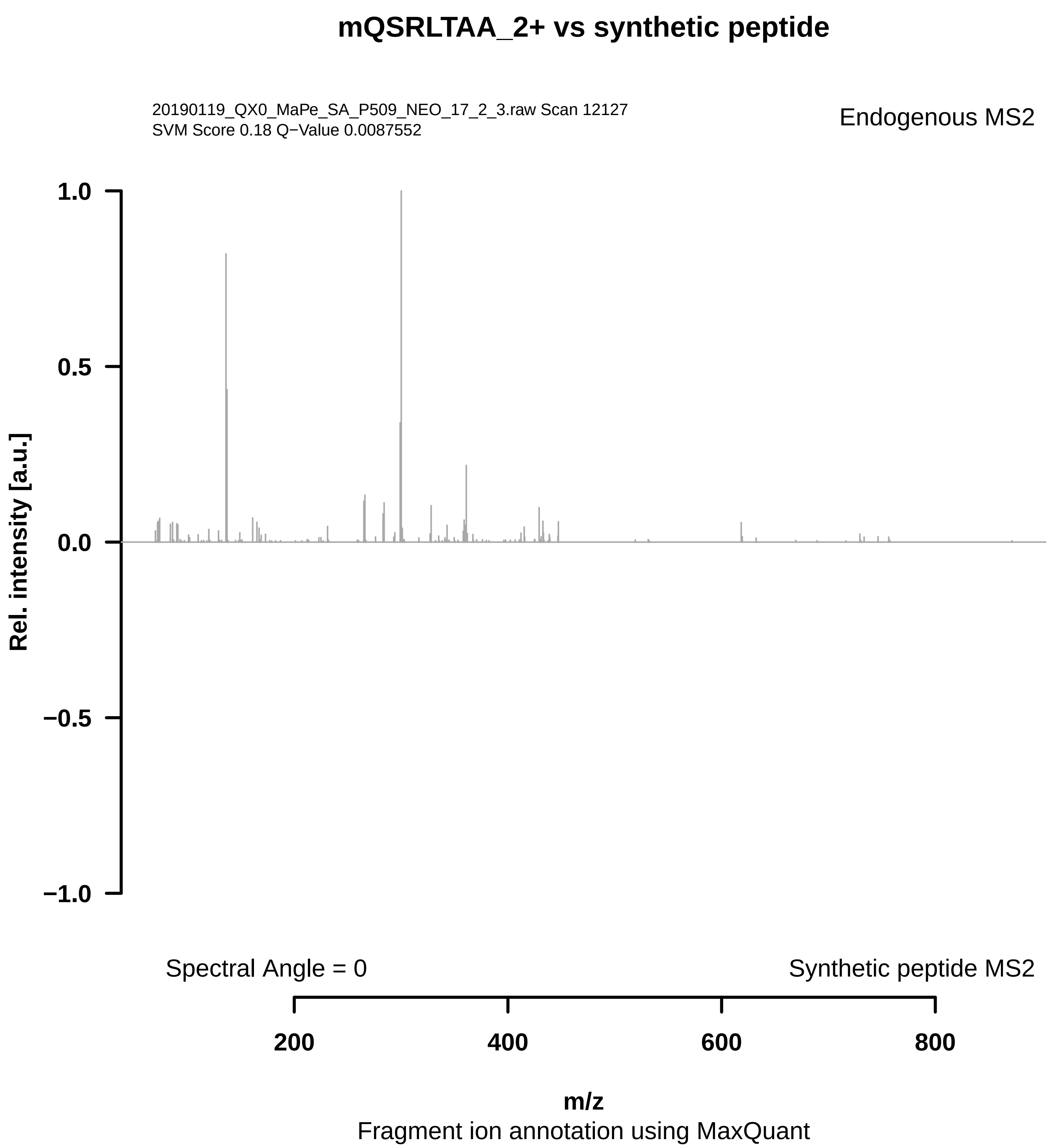

mQSRLTAA_2+ vs synthetic peptide
20190119_QX0_MaPe_SA_P509_NEO_17_2_3.raw Scan 12127
SVM Score 0.18 Q−Value 0.0087552
Endogenous MS2
Rel. intensity [a.u.]
1.0
0.5
0.0
−0.5
−1.0
Spectral Angle = 0
Synthetic peptide MS2
200
400
600
800
m/z
Fragment ion annotation using MaxQuant

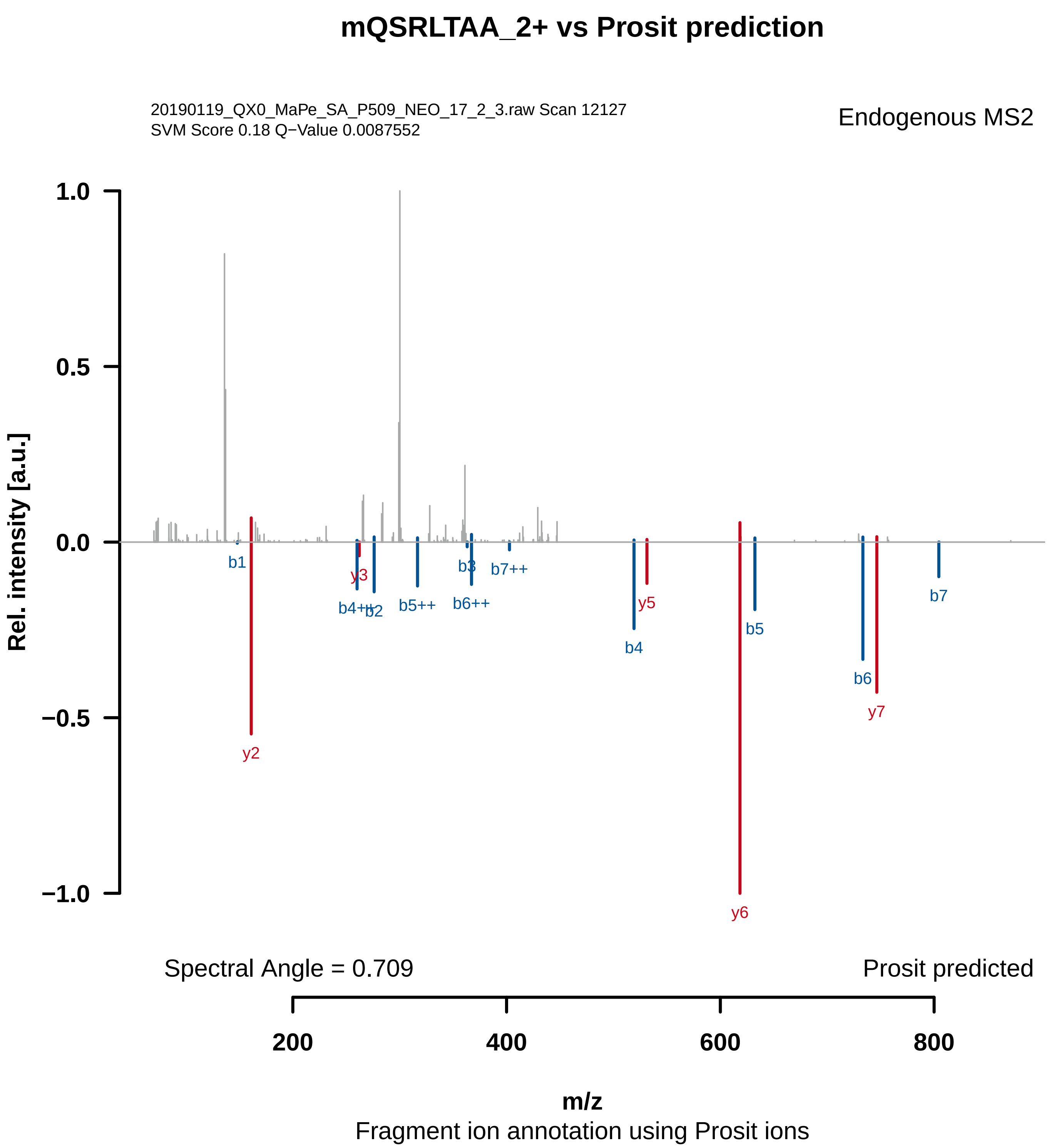

mQSRLTAA_2+ vs Prosit prediction
20190119_QX0_MaPe_SA_P509_NEO_17_2_3.raw Scan 12127
SVM Score 0.18 Q−Value 0.0087552
Endogenous MS2
Rel. intensity [a.u.]
1.0
0.5
0.0
−0.5
−1.0
b1
y2
y3
b4++
b2
b5++
b3
b6++
b7++
b4
y5
y6
b5
b6
y7
b7
Spectral Angle = 0.709
Prosit predicted
200
400
600
800
m/z
Fragment ion annotation using Prosit ions

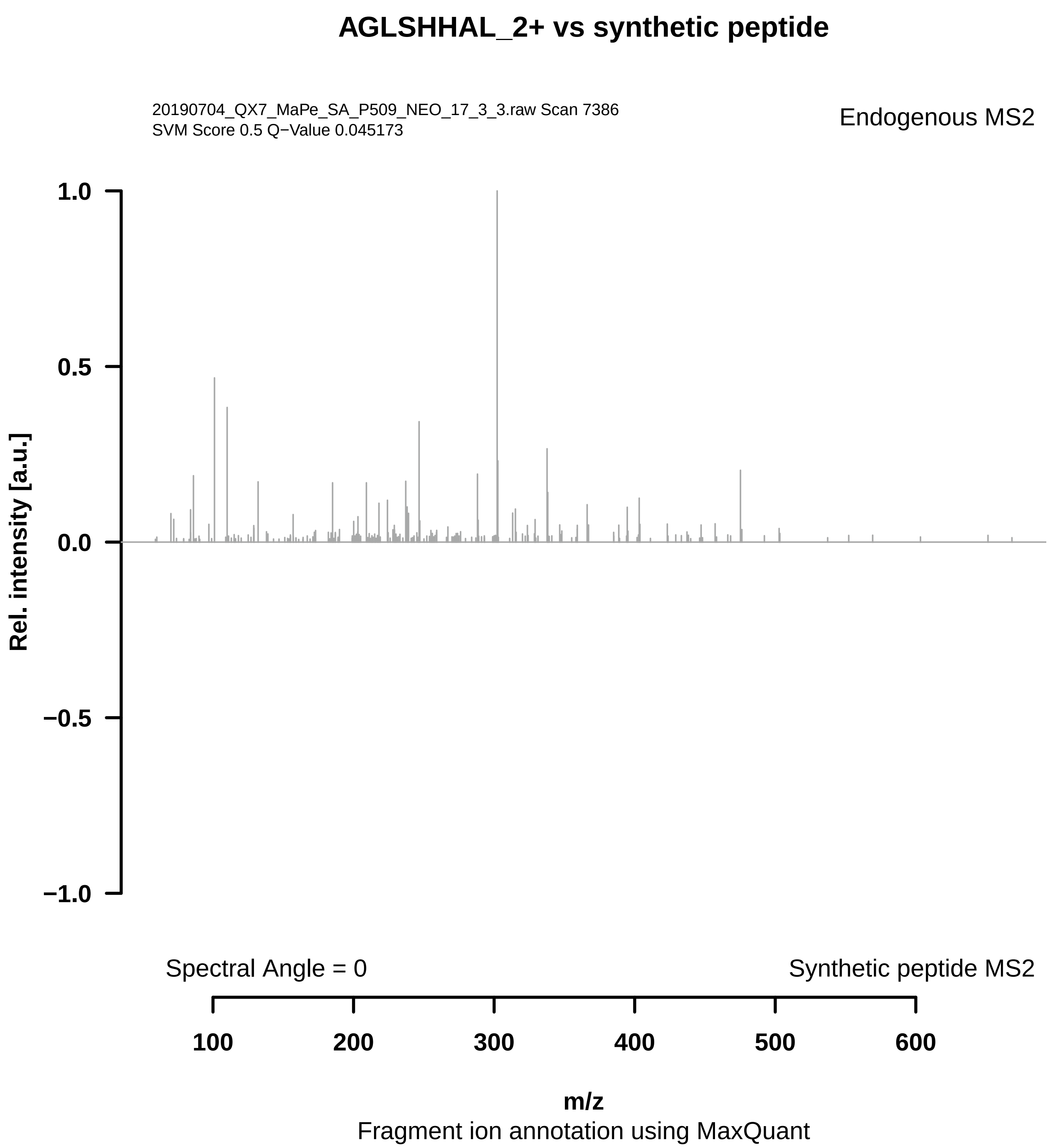
AGLSHHAL_2+ vs synthetic peptide
20190704_QX7_MaPe_SA_P509_NEO_17_3_3.raw Scan 7386
SVM Score 0.5 Q−Value 0.045173
Endogenous MS2
Rel. intensity [a.u.]
1.0
0.5
0.0
−0.5
−1.0
Spectral Angle = 0
Synthetic peptide MS2
100
200
300
400
500
600
m/z
Fragment ion annotation using MaxQuant

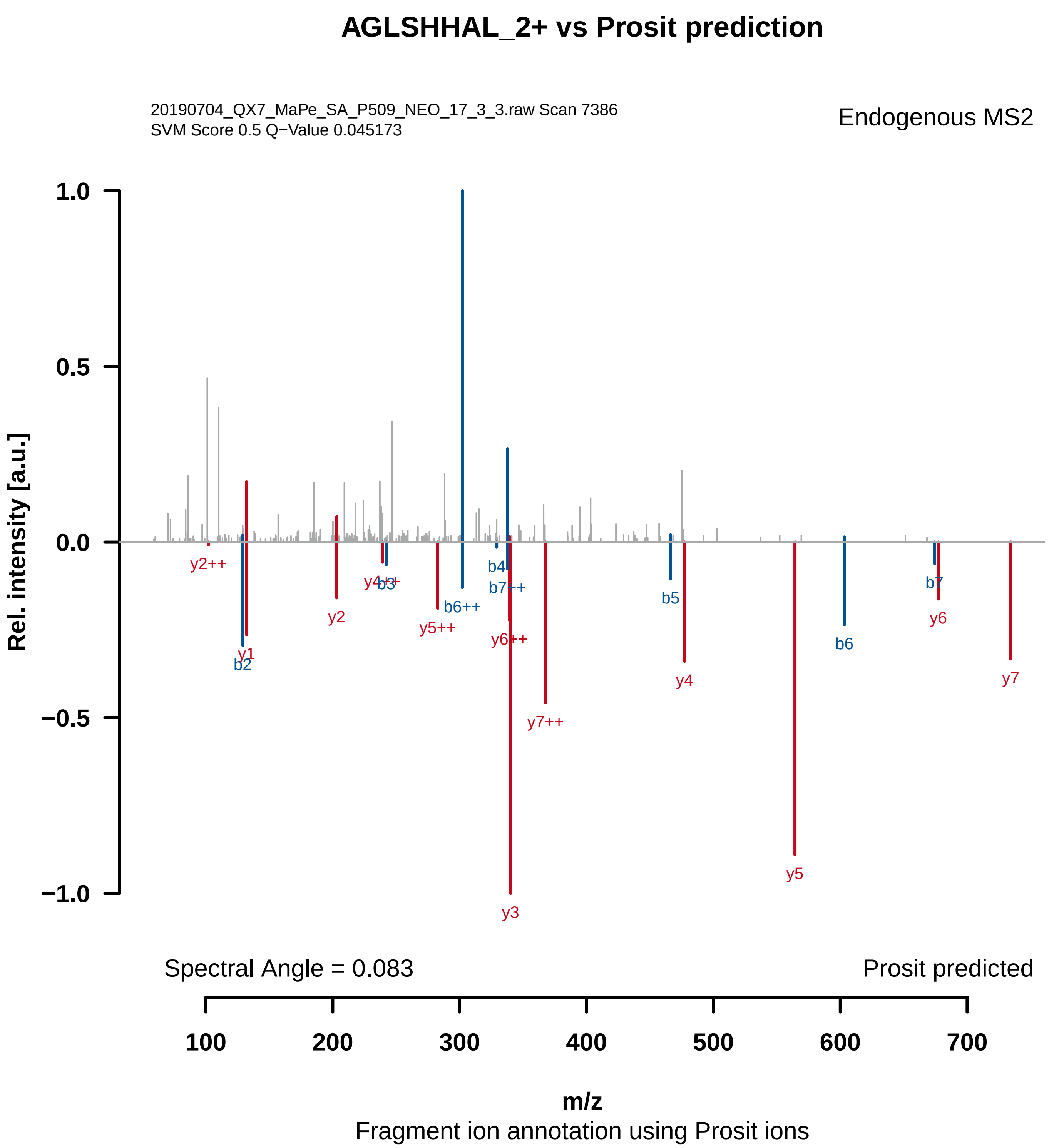
AGLSHHAL_2+ vs Prosit prediction
20190704_QX7_MaPe_SA_P509_NEO_17_3_3.raw Scan 7386
SVM Score 0.5 Q−Value 0.045173
Endogenous MS2
Rel. intensity [a.u.]
1.0
0.5
0.0
−0.5
−1.0
y2++
y1
b2
y2
y4++
b3
y5++
b4
b7++
b6++
y6++
y3
y7++
b5
y4
y5
b6
b7
y6
y7
Spectral Angle = 0.083
Prosit predicted
100
200
300
400
500
600
700
m/z
Fragment ion annotation using Prosit ions

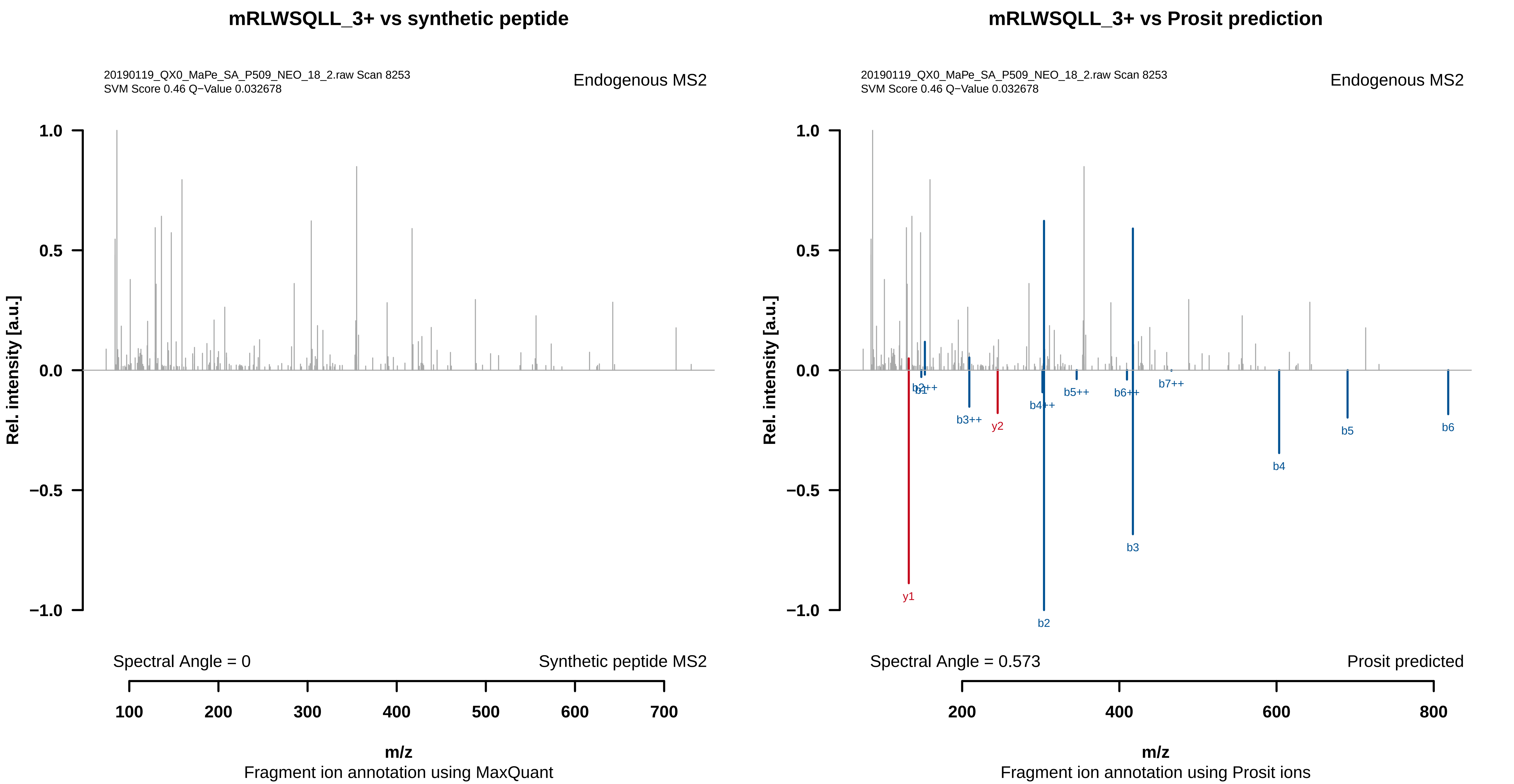
mRLWSQLL_3+ vs synthetic peptide
20190119_QX0_MaPe_SA_P509_NEO_18_2.raw Scan 8253
SVM Score 0.46 Q−Value 0.032678
Endogenous MS2
Rel. intensity [a.u.]
Spectral Angle = 0
Synthetic peptide MS2
m/z
Fragment ion annotation using MaxQuant
mRLWSQLL_3+ vs Prosit prediction
20190119_QX0_MaPe_SA_P509_NEO_18_2.raw Scan 8253
SVM Score 0.46 Q−Value 0.032678
Endogenous MS2
Rel. intensity [a.u.]
y1
y2
b2
b3
b4
b5
b6
b3++
b4++
b5++
b6++
b7++
Spectral Angle = 0.573
Prosit predicted
m/z
Fragment ion annotation using Prosit ions

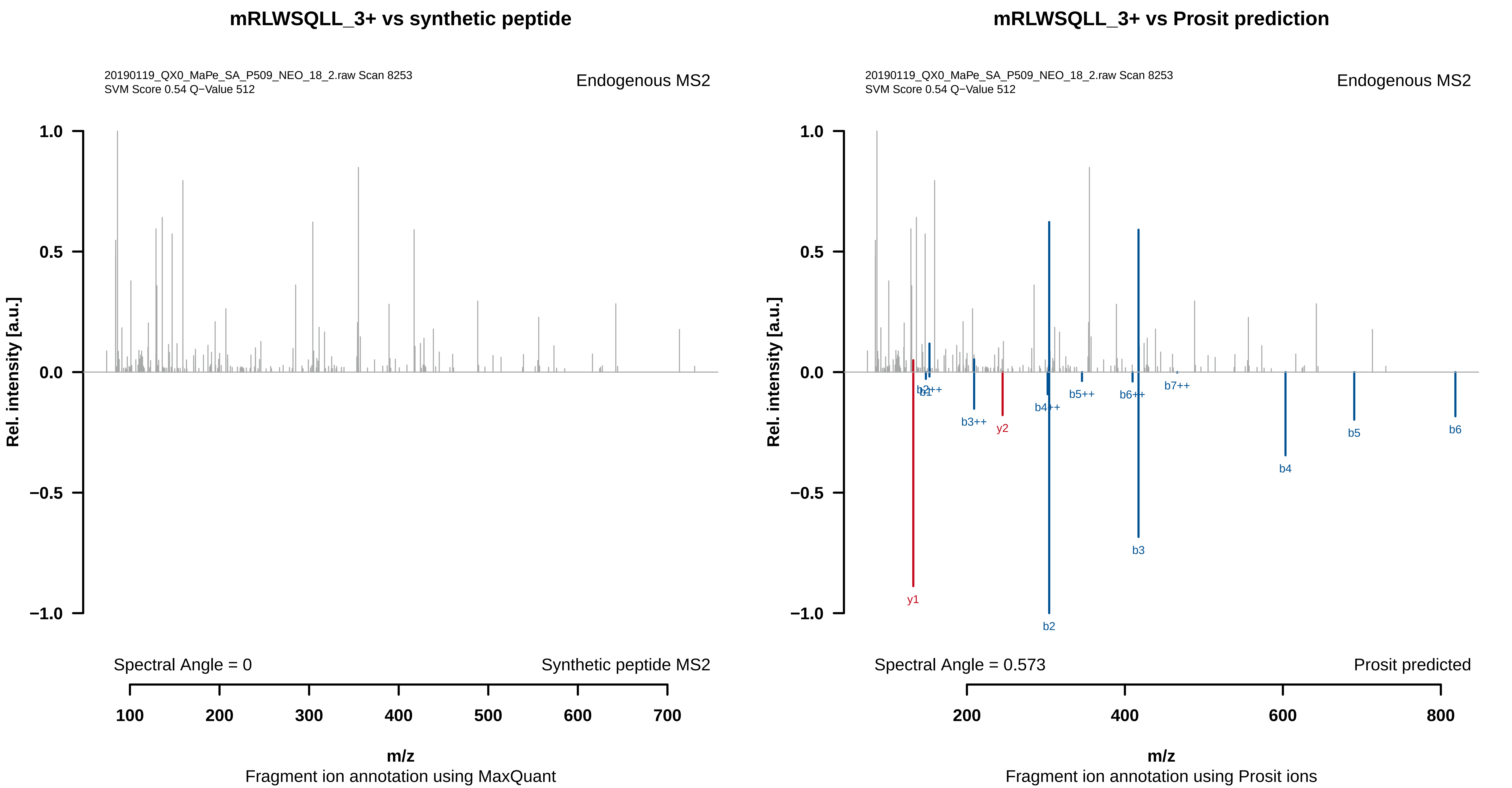

mRLWSQLL_3+ vs synthetic peptide
20190119_QX0_MaPe_SA_P509_NEO_18_2.raw Scan 8253
SVM Score 0.54 Q−Value 512
Endogenous MS2
Rel. intensity [a.u.]
1.0
0.5
0.0
−0.5
−1.0
Spectral Angle = 0
Synthetic peptide MS2
100
200
300
400
500
600
700
m/z
Fragment ion annotation using MaxQuant
mRLWSQLL_3+ vs Prosit prediction
20190119_QX0_MaPe_SA_P509_NEO_18_2.raw Scan 8253
SVM Score 0.54 Q−Value 512
Endogenous MS2
Rel. intensity [a.u.]
b2++
b1
b3++
y2
b4++
b5++
b6++
b7++
b4
b5
b6
b3
y1
b2
Spectral Angle = 0.573
Prosit predicted
200
400
600
800
m/z
Fragment ion annotation using Prosit ions

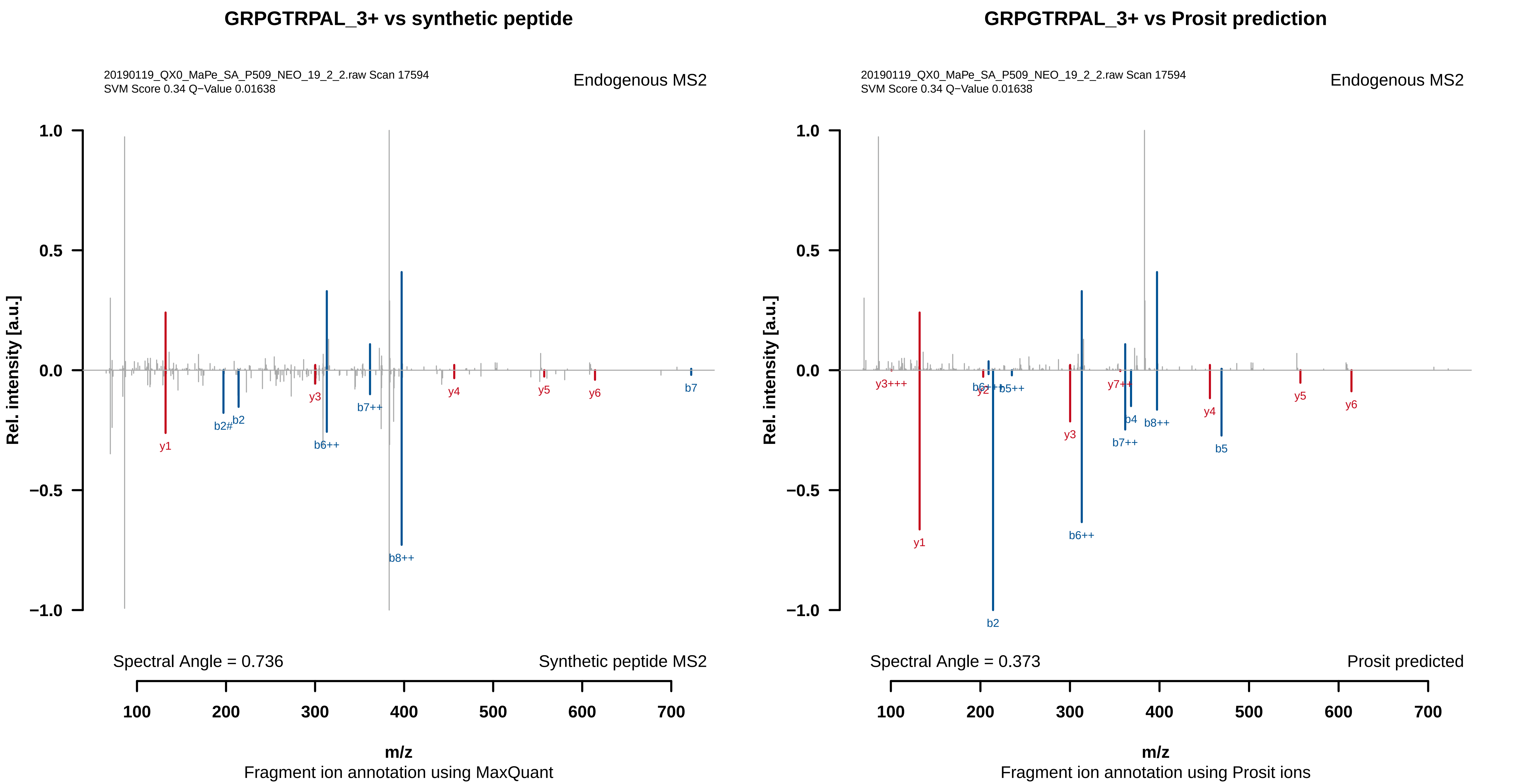
GRPGTRPAL_3+ vs synthetic peptide
20190119_QX0_MaPe_SA_P509_NEO_19_2_2.raw Scan 17594
SVM Score 0.34 Q−Value 0.01638
Endogenous MS2
Rel. intensity [a.u.]
1.0
0.5
0.0
−0.5
−1.0
y1
b2#
b2
y3
b6++
b7++
b8++
y4
y5
y6
b7
Spectral Angle = 0.736
Synthetic peptide MS2
100
200
300
400
500
600
700
m/z
Fragment ion annotation using MaxQuant
GRPGTRPAL_3+ vs Prosit prediction
20190119_QX0_MaPe_SA_P509_NEO_19_2_2.raw Scan 17594
SVM Score 0.34 Q−Value 0.01638
Endogenous MS2
Rel. intensity [a.u.]
y3+++
y1
b6+++
y2
b5++
b2
y3
b6++
y7++
b4
b7++
b8++
y4
b5
y5
y6
Spectral Angle = 0.373
Prosit predicted
m/z
Fragment ion annotation using Prosit ions

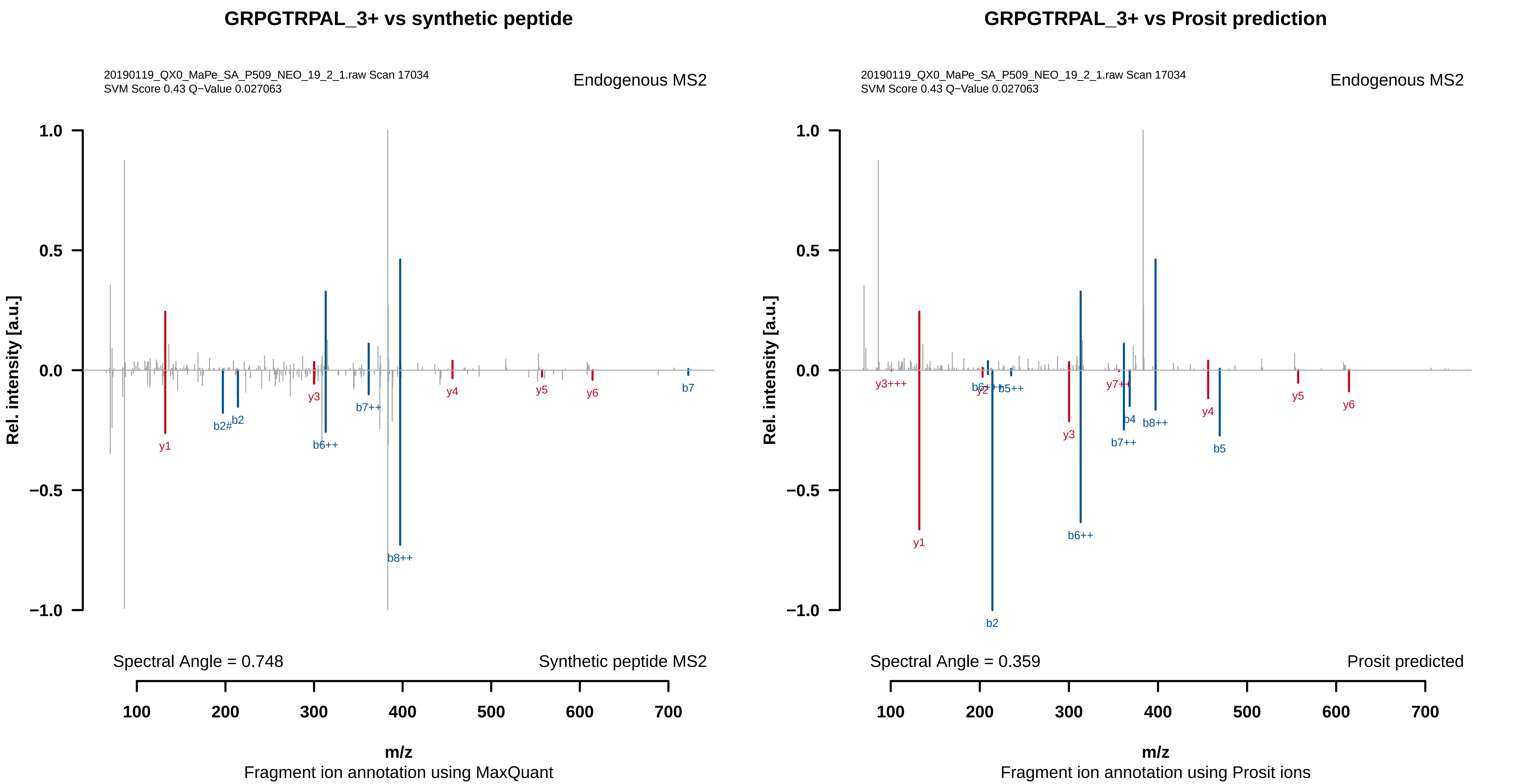
GRPGTRPAL_3+ vs synthetic peptide
20190119_QX0_MaPe_SA_P509_NEO_19_2_1.raw Scan 17034
SVM Score 0.43 Q−Value 0.027063
Endogenous MS2
Rel. intensity [a.u.]
1.0
0.5
0.0
−0.5
−1.0
y1
b2#
b2
y3
b6++
b7++
b8++
y4
y5
y6
b7
Spectral Angle = 0.748
Synthetic peptide MS2
100
200
300
400
500
600
700
m/z
Fragment ion annotation using MaxQuant
GRPGTRPAL_3+ vs Prosit prediction
20190119_QX0_MaPe_SA_P509_NEO_19_2_1.raw Scan 17034
SVM Score 0.43 Q−Value 0.027063
Endogenous MS2
Rel. intensity [a.u.]
y3+++
y1
b6+++
y2
b5++
b2
y3
b6++
y7++
b4
b7++
b8++
y4
b5
y5
y6
Spectral Angle = 0.359
Prosit predicted
m/z
Fragment ion annotation using Prosit ions

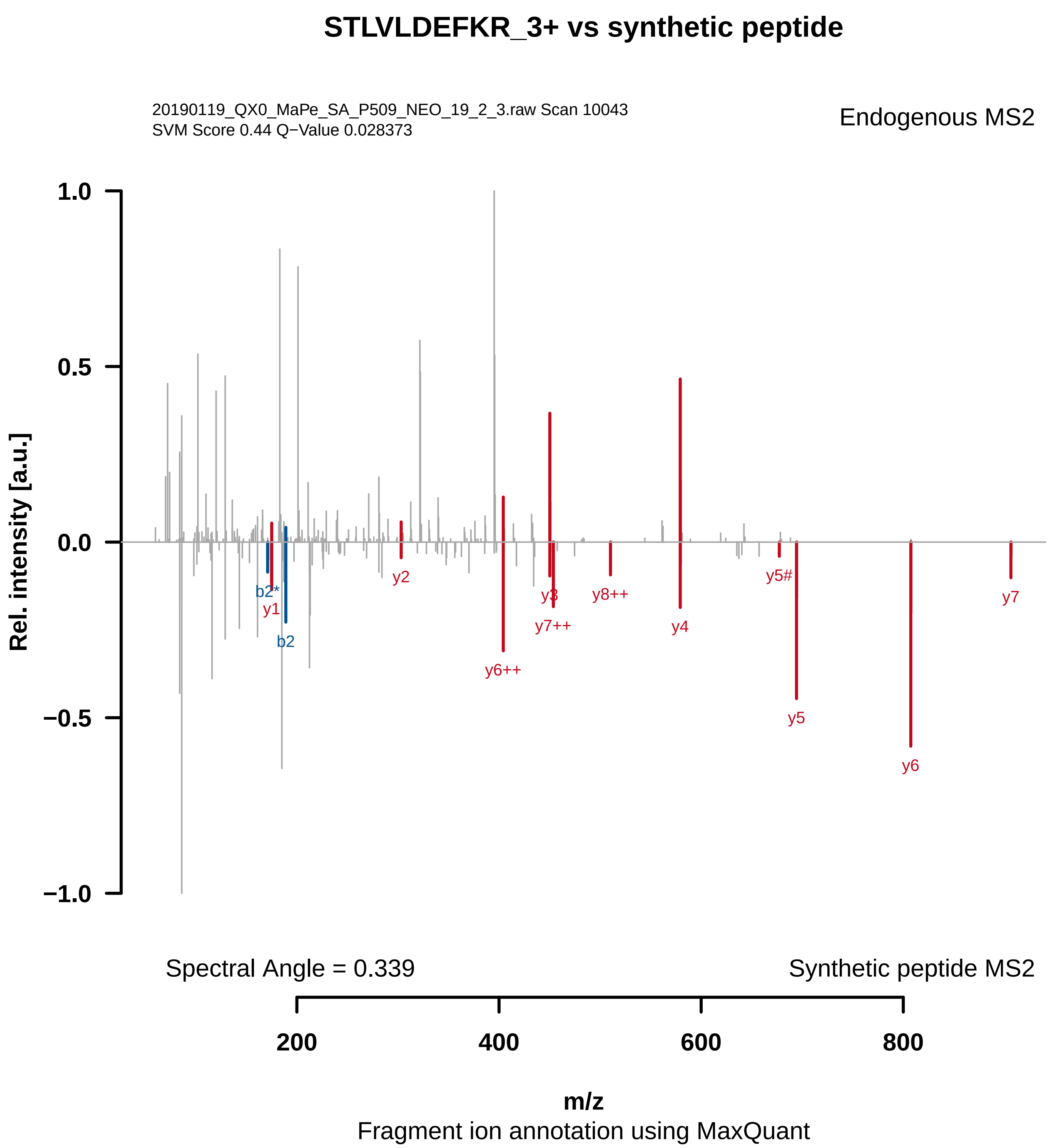
STLVLDEFKR_3+ vs synthetic peptide
20190119_QX0_MaPe_SA_P509_NEO_19_2_3.raw Scan 10043
SVM Score 0.44 Q−Value 0.028373
Endogenous MS2
Rel. intensity [a.u.]
1.0
0.5
0.0
−0.5
−1.0
b2*
y1
b2
y2
y6++
y3
y7++
y8++
y4
y5#
y5
y6
y7
Spectral Angle = 0.339
Synthetic peptide MS2
200
400
600
800
m/z
Fragment ion annotation using MaxQuant
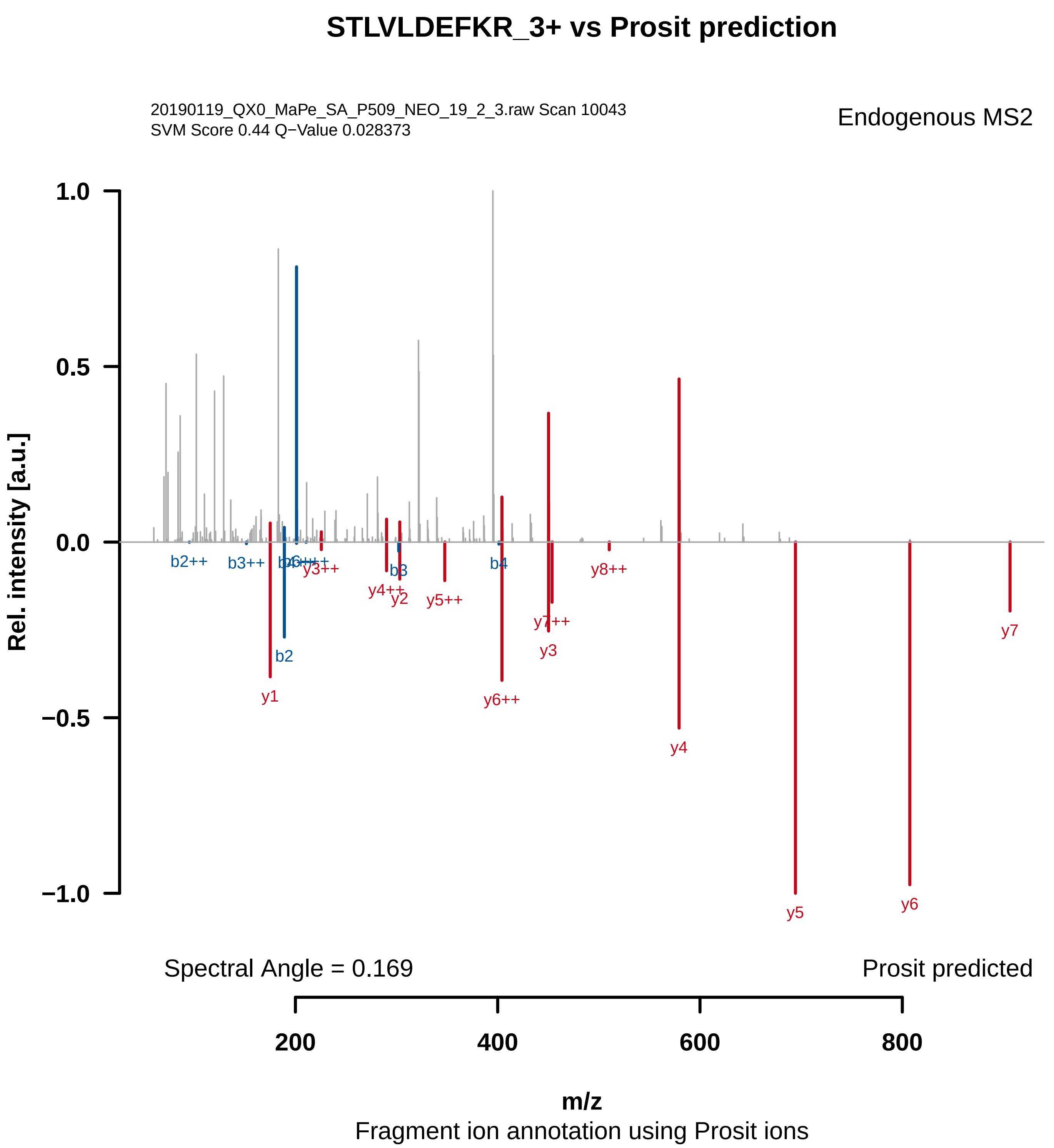
STLVLDEFKR_3+ vs Prosit prediction
20190119_QX0_MaPe_SA_P509_NEO_19_2_3.raw Scan 10043
SVM Score 0.44 Q−Value 0.028373
Endogenous MS2
Rel. intensity [a.u.]
1.0
0.5
0.0
−0.5
−1.0
b2++
b3++
y3++
y4++
b3
y2
y5++
b4
y7++
y3
y8++
b2
y1
y6++
y4
y5
y6
y7
Spectral Angle = 0.169
Prosit predicted
200
400
600
800
m/z
Fragment ion annotation using Prosit ions

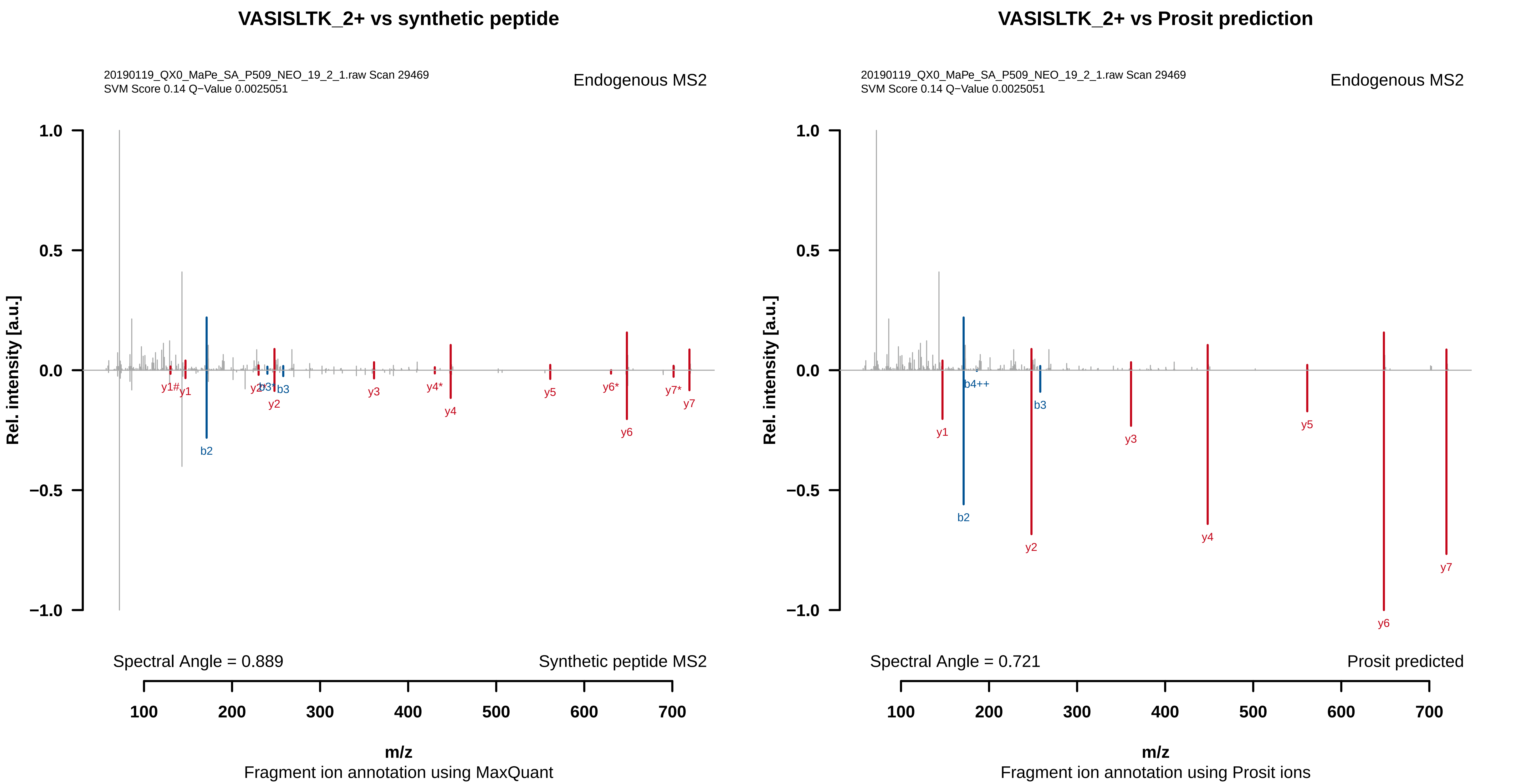

VASISLTK_2+ vs synthetic peptide
20190119_QX0_MaPe_SA_P509_NEO_19_2_1.raw Scan 29469
SVM Score 0.14 Q−Value 0.0025051
Endogenous MS2
Rel. intensity [a.u.]
1.0
0.5
0.0
−0.5
−1.0
y1#
y1
b2
y2
b3*
b3
y2
y3
y4*
y4
y5
y6*
y6
y7*
y7
Spectral Angle = 0.889
Synthetic peptide MS2
100
200
300
400
500
600
700
m/z
Fragment ion annotation using MaxQuant
VASISLTK_2+ vs Prosit prediction
20190119_QX0_MaPe_SA_P509_NEO_19_2_1.raw Scan 29469
SVM Score 0.14 Q−Value 0.0025051
Endogenous MS2
Rel. intensity [a.u.]
1.0
0.5
0.0
−0.5
−1.0
b4++
b3
y1
b2
y2
y3
y4
y5
y6
y7
Spectral Angle = 0.721
Prosit predicted
100
200
300
400
500
600
700
m/z
Fragment ion annotation using Prosit ions

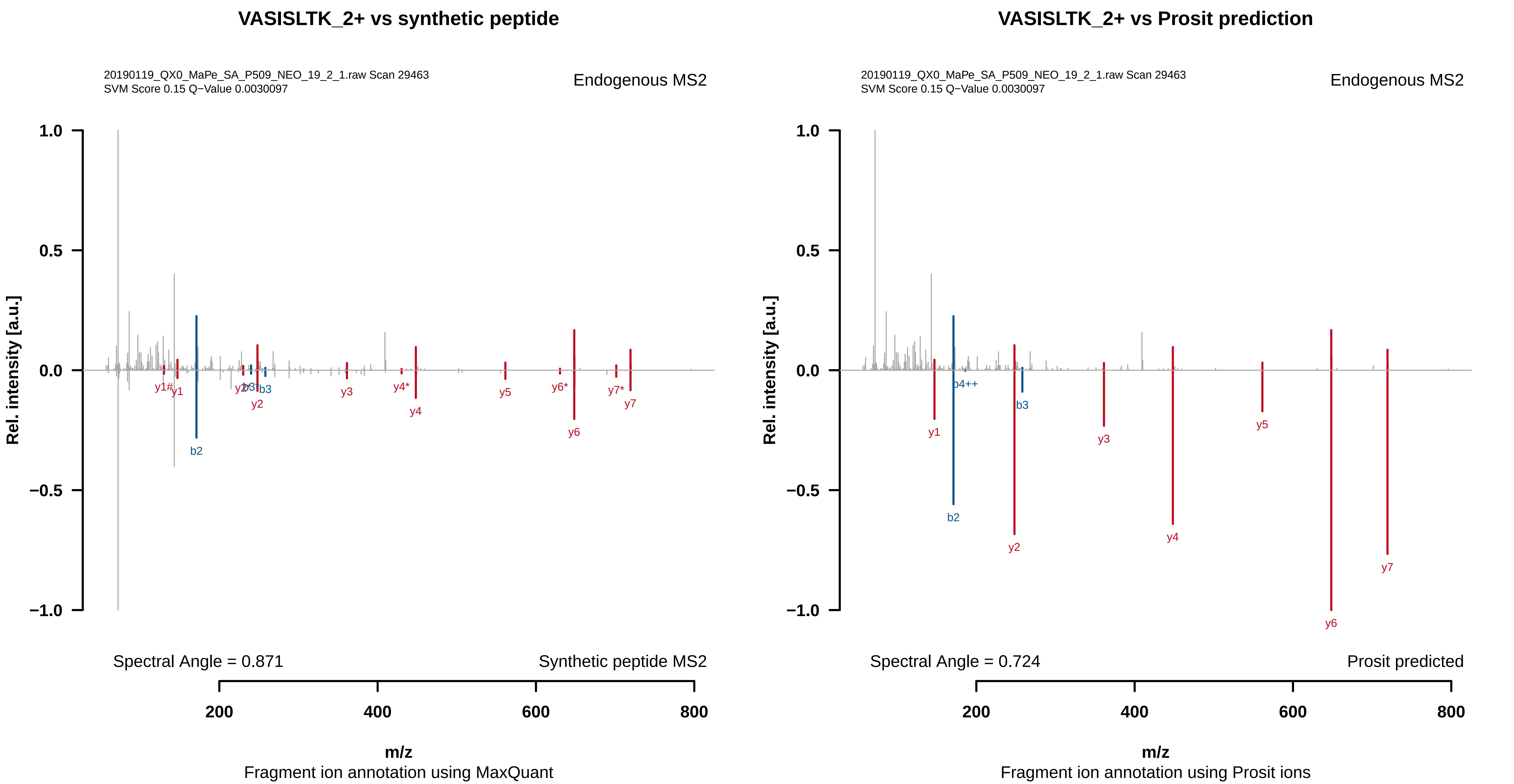
VASISLTK_2+ vs synthetic peptide
20190119_QX0_MaPe_SA_P509_NEO_19_2_1.raw Scan 29463
SVM Score 0.15 Q−Value 0.0030097
Endogenous MS2
Rel. intensity [a.u.]
Spectral Angle = 0.871
Synthetic peptide MS2
m/z
Fragment ion annotation using MaxQuant
VASISLTK_2+ vs Prosit prediction
20190119_QX0_MaPe_SA_P509_NEO_19_2_1.raw Scan 29463
SVM Score 0.15 Q−Value 0.0030097
Endogenous MS2
Rel. intensity [a.u.]
Spectral Angle = 0.724
Prosit predicted
m/z
Fragment ion annotation using Prosit ions

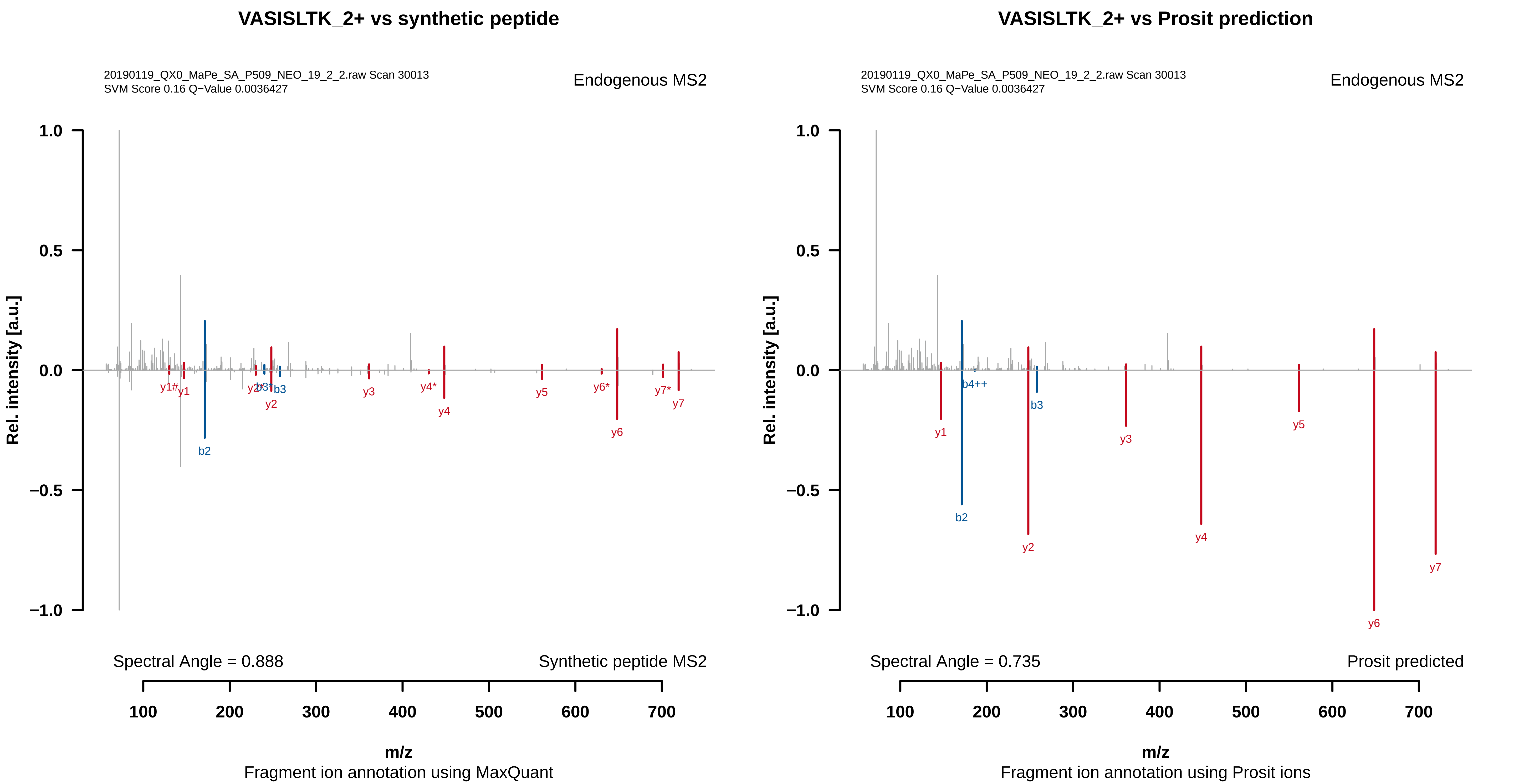
VASISLTK_2+ vs synthetic peptide
20190119_QX0_MaPe_SA_P509_NEO_19_2_2.raw Scan 30013
SVM Score 0.16 Q−Value 0.0036427
Endogenous MS2
Rel. intensity [a.u.]
1.0
0.5
0.0
−0.5
−1.0
y1#
y1
b2
y2
b3
y2
y3
y4*
y4
y5
y6*
y6
y7*
y7
Spectral Angle = 0.888
Synthetic peptide MS2
100
200
300
400
500
600
700
m/z
Fragment ion annotation using MaxQuant
VASISLTK_2+ vs Prosit prediction
20190119_QX0_MaPe_SA_P509_NEO_19_2_2.raw Scan 30013
SVM Score 0.16 Q−Value 0.0036427
Endogenous MS2
Rel. intensity [a.u.]
1.0
0.5
0.0
−0.5
−1.0
b4++
b3
y1
y3
y5
b2
y2
y4
y7
y6
Spectral Angle = 0.735
Prosit predicted
100
200
300
400
500
600
700
m/z
Fragment ion annotation using Prosit ions

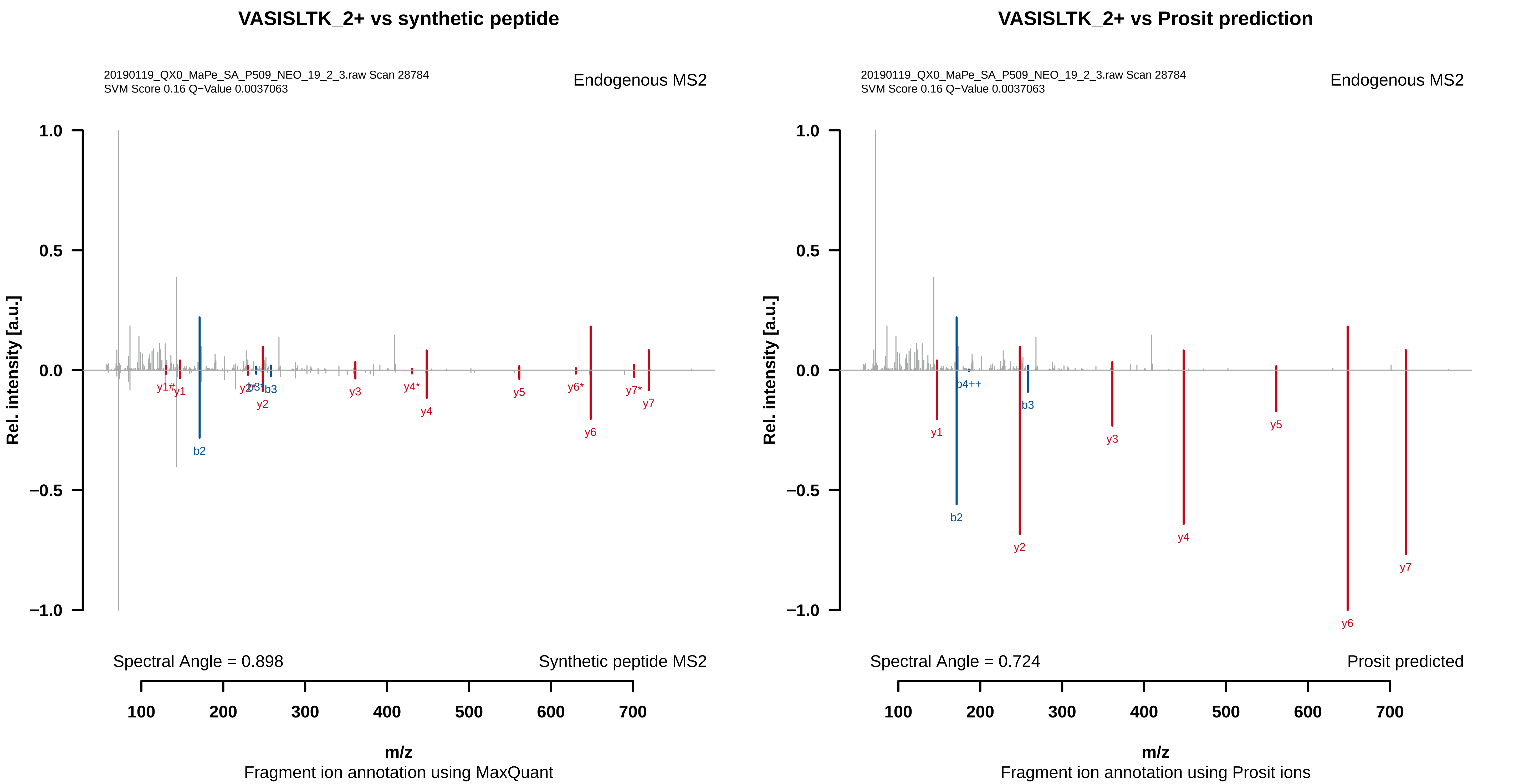
VASISLTK_2+ vs synthetic peptide
20190119_QX0_MaPe_SA_P509_NEO_19_2_3.raw Scan 28784
SVM Score 0.16 Q−Value 0.0037063
Endogenous MS2
1.0
0.5
0.0
−0.5
−1.0
Rel. intensity [a.u.]
y1#
y1
b2
y2
b3
y3
y4*
y4
y5
y6*
y6
y7*
y7
Spectral Angle = 0.898
Synthetic peptide MS2
100
200
300
400
500
600
700
m/z
Fragment ion annotation using MaxQuant
VASISLTK_2+ vs Prosit prediction
20190119_QX0_MaPe_SA_P509_NEO_19_2_3.raw Scan 28784
SVM Score 0.16 Q−Value 0.0037063
Endogenous MS2
1.0
0.5
0.0
−0.5
−1.0
Rel. intensity [a.u.]
y1
b4++
b2
y2
b3
y3
y4
y5
y6
y7
Spectral Angle = 0.724
Prosit predicted
100
200
300
400
500
600
700
m/z
Fragment ion annotation using Prosit ions

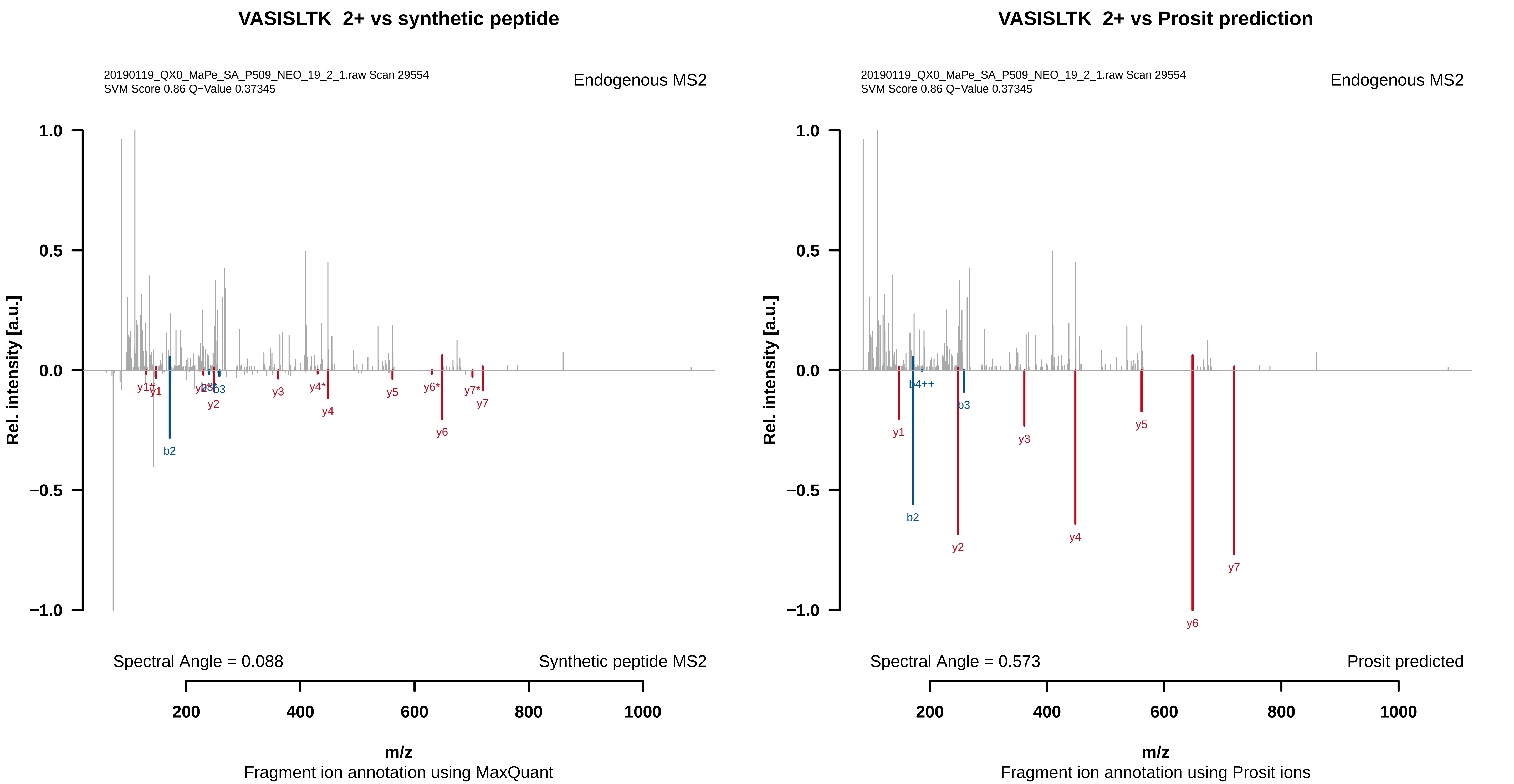

VASISLTK_2+ vs synthetic peptide
20190119_QX0_MaPe_SA_P509_NEO_19_2_1.raw Scan 29554
SVM Score 0.86 Q−Value 0.37345
Endogenous MS2
Rel. intensity [a.u.]
1.0
0.5
0.0
−0.5
−1.0
y1#
y1
y2*
b3*
b3
y2
b2
y3
y4*
y4
y5
y6*
y6
y7*
y7
Spectral Angle = 0.088
Synthetic peptide MS2
200
400
600
800
1000
m/z
Fragment ion annotation using MaxQuant
VASISLTK_2+ vs Prosit prediction
20190119_QX0_MaPe_SA_P509_NEO_19_2_1.raw Scan 29554
SVM Score 0.86 Q−Value 0.37345
Endogenous MS2
Rel. intensity [a.u.]
1.0
0.5
0.0
−0.5
−1.0
y1
b4++
b2
y2
b3
y3
y4
y5
y6
y7
Spectral Angle = 0.573
Prosit predicted
200
400
600
800
1000
m/z
Fragment ion annotation using Prosit ions

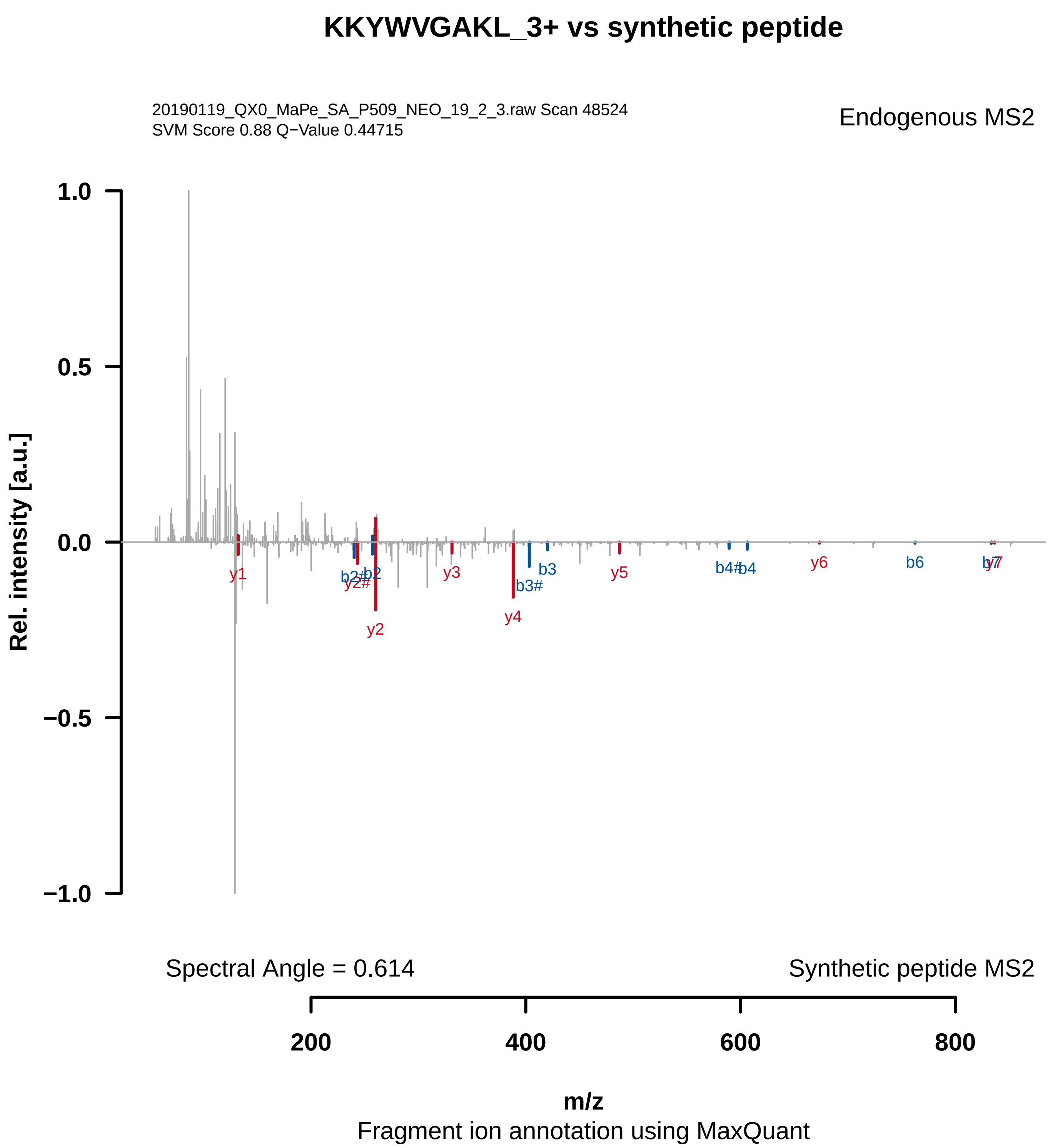
KKYWVGAKL_3+ vs synthetic peptide
20190119_QX0_MaPe_SA_P509_NEO_19_2_3.raw Scan 48524
SVM Score 0.88 Q−Value 0.44715
Endogenous MS2
Rel. intensity [a.u.]
1.0
0.5
0.0
−0.5
−1.0
y1
b2#
y2#
b2
y2
y3
y4
b3
b3#
y5
b4#
b4
y6
b6
b7
y7
Spectral Angle = 0.614
Synthetic peptide MS2
200
400
600
800
m/z
Fragment ion annotation using MaxQuant

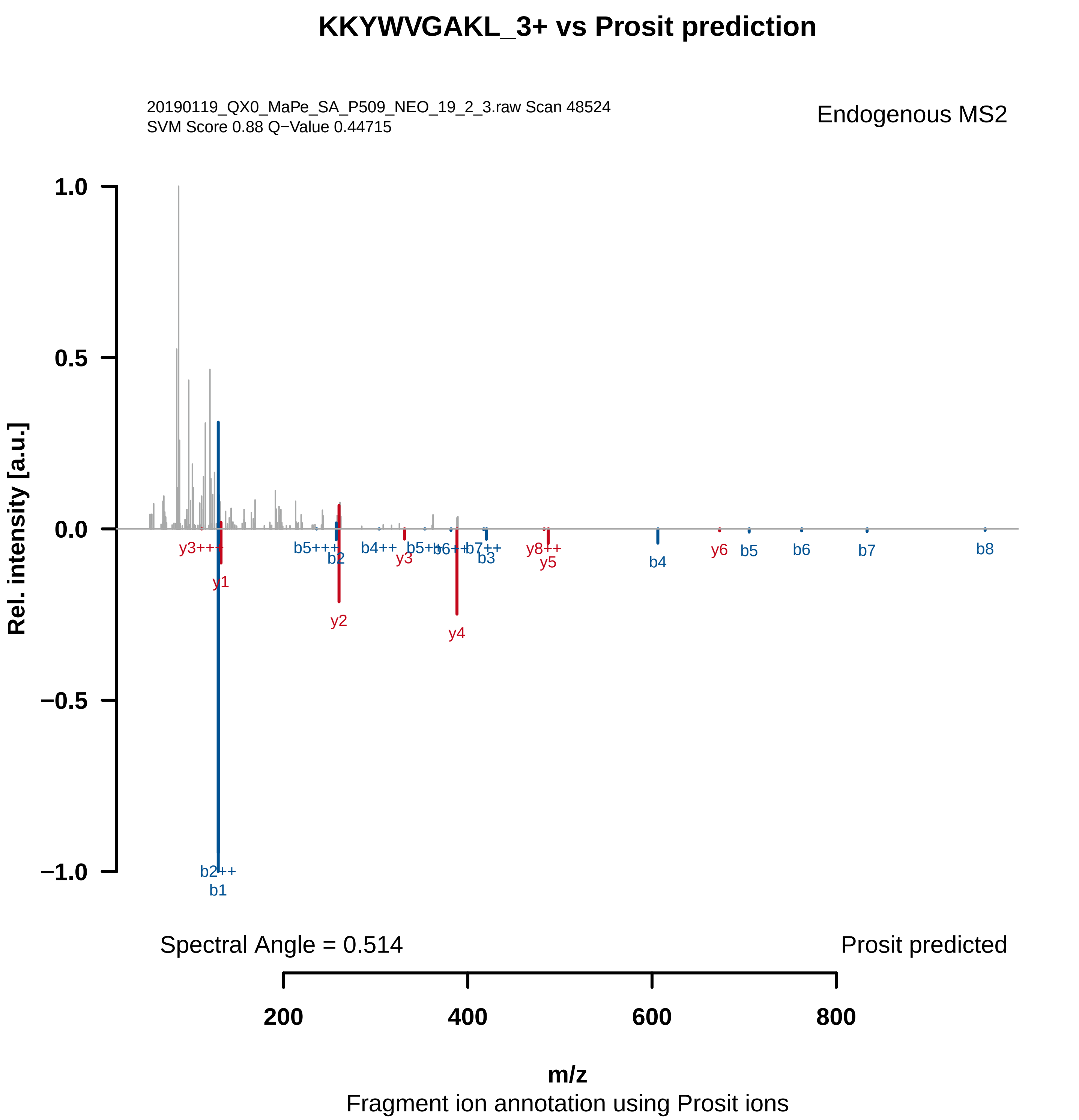
KKYWVGAKL_3+ vs Prosit prediction
20190119_QX0_MaPe_SA_P509_NEO_19_2_3.raw Scan 48524
SVM Score 0.88 Q−Value 0.44715
Endogenous MS2
Rel. intensity [a.u.]
1.0
0.5
0.0
−0.5
−1.0
y3+++
y1
b5+++
b2
y2
b4++
y3
b5++
b6++
b7++
b3
y4
y8++
y5
b4
y6
b5
b6
b7
b8
b2++
b1
Spectral Angle = 0.514
Prosit predicted
200
400
600
800
m/z
Fragment ion annotation using Prosit ions

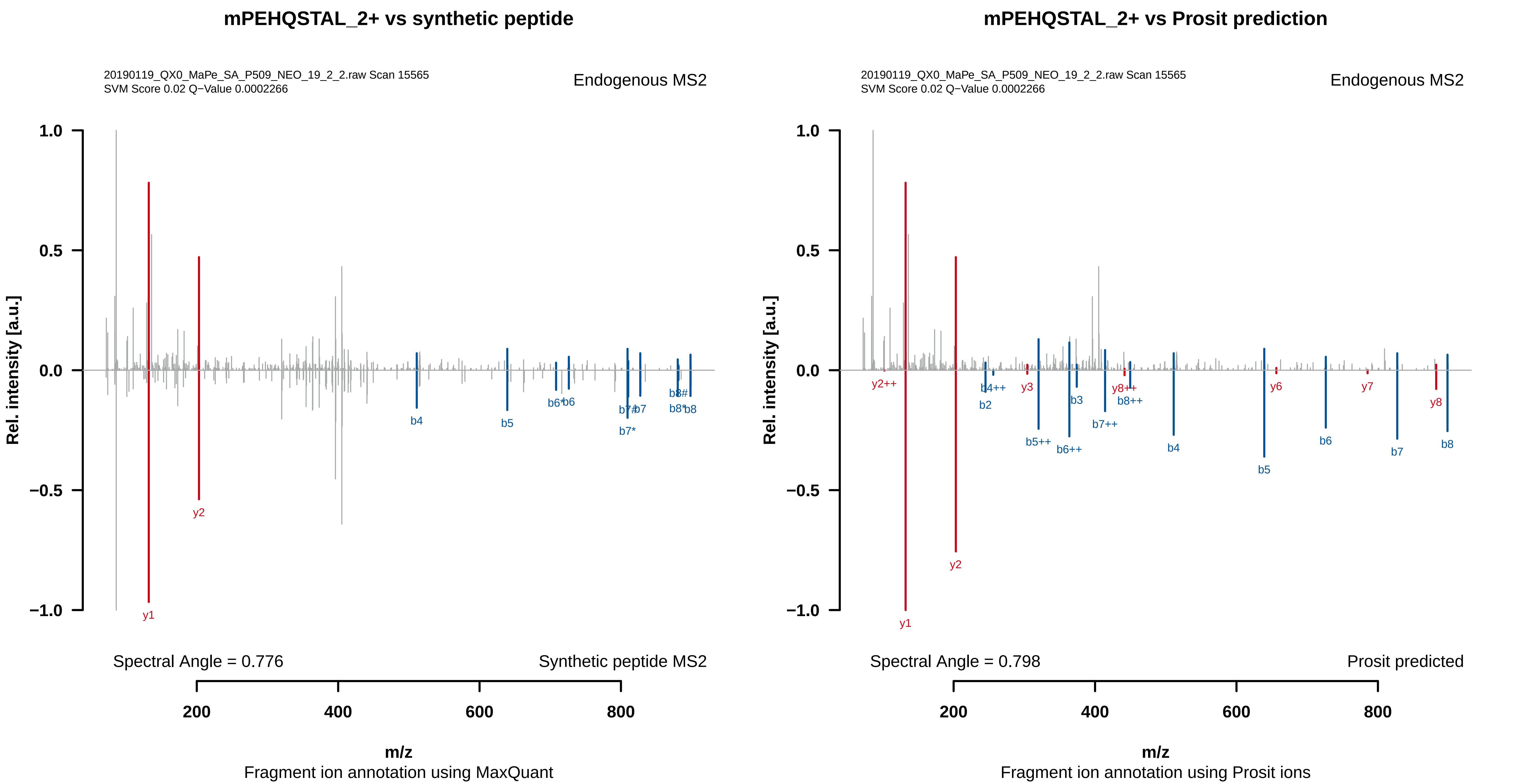
mPEHQSTAL_2+ vs synthetic peptide
20190119_QX0_MaPe_SA_P509_NEO_19_2_2.raw Scan 15565
SVM Score 0.02 Q−Value 0.0002266
Endogenous MS2
Rel. intensity [a.u.]
1.0
0.5
0.0
−0.5
−1.0
y1
y2
b4
b5
b6* b6
b7# b7
b7*
b8#
b8* b8
Spectral Angle = 0.776
Synthetic peptide MS2
200
400
600
800
m/z
Fragment ion annotation using MaxQuant
mPEHQSTAL_2+ vs Prosit prediction
20190119_QX0_MaPe_SA_P509_NEO_19_2_2.raw Scan 15565
SVM Score 0.02 Q−Value 0.0002266
Endogenous MS2
Rel. intensity [a.u.]
1.0
0.5
0.0
−0.5
−1.0
y2++
y1
y2
b4++
b2
y3
b5++
b6++
b3
y8++
b8++
b7++
b4
b5
y6
b6
y7
b7
y8
b8
Spectral Angle = 0.798
Prosit predicted
200
400
600
800
m/z
Fragment ion annotation using Prosit ions

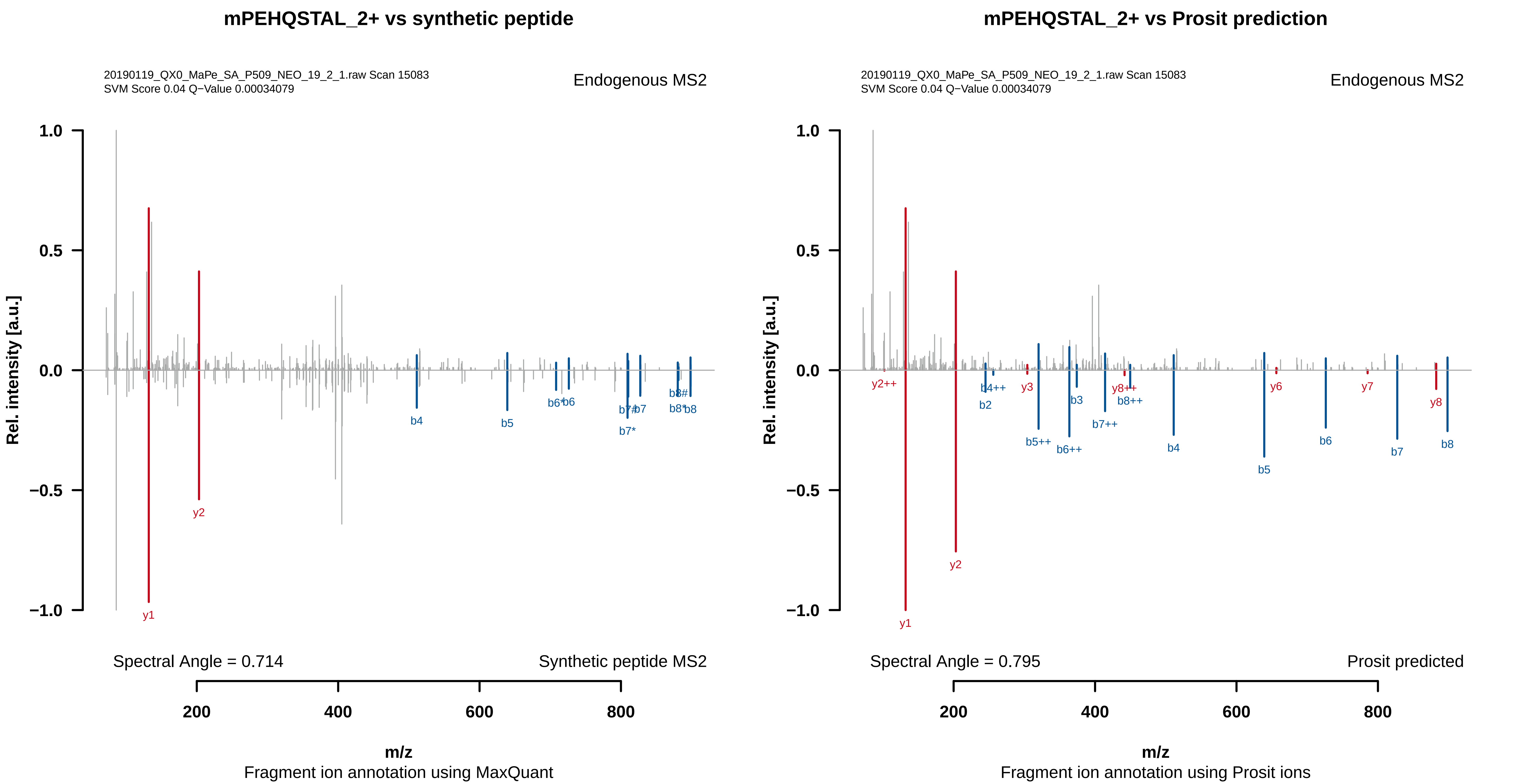

mPEHQSTAL_2+ vs synthetic peptide
20190119_QX0_MaPe_SA_P509_NEO_19_2_1.raw Scan 15083
SVM Score 0.04 Q−Value 0.00034079
Endogenous MS2
Rel. intensity [a.u.]
1.0
0.5
0.0
−0.5
−1.0
y1
y2
b4
b5
b6*
b6
b7#
b7
b7*
b8#
b8*
b8
Spectral Angle = 0.714
Synthetic peptide MS2
200
400
600
800
m/z
Fragment ion annotation using MaxQuant
mPEHQSTAL_2+ vs Prosit prediction
20190119_QX0_MaPe_SA_P509_NEO_19_2_1.raw Scan 15083
SVM Score 0.04 Q−Value 0.00034079
Endogenous MS2
Rel. intensity [a.u.]
1.0
0.5
0.0
−0.5
−1.0
y2++
y1
y2
b4++
b2
y3
b5++
b6++
b3
y8++
b8++
b7++
b4
b5
y6
b6
y7
b7
y8
b8
Spectral Angle = 0.795
Prosit predicted
200
400
600
800
m/z
Fragment ion annotation using Prosit ions

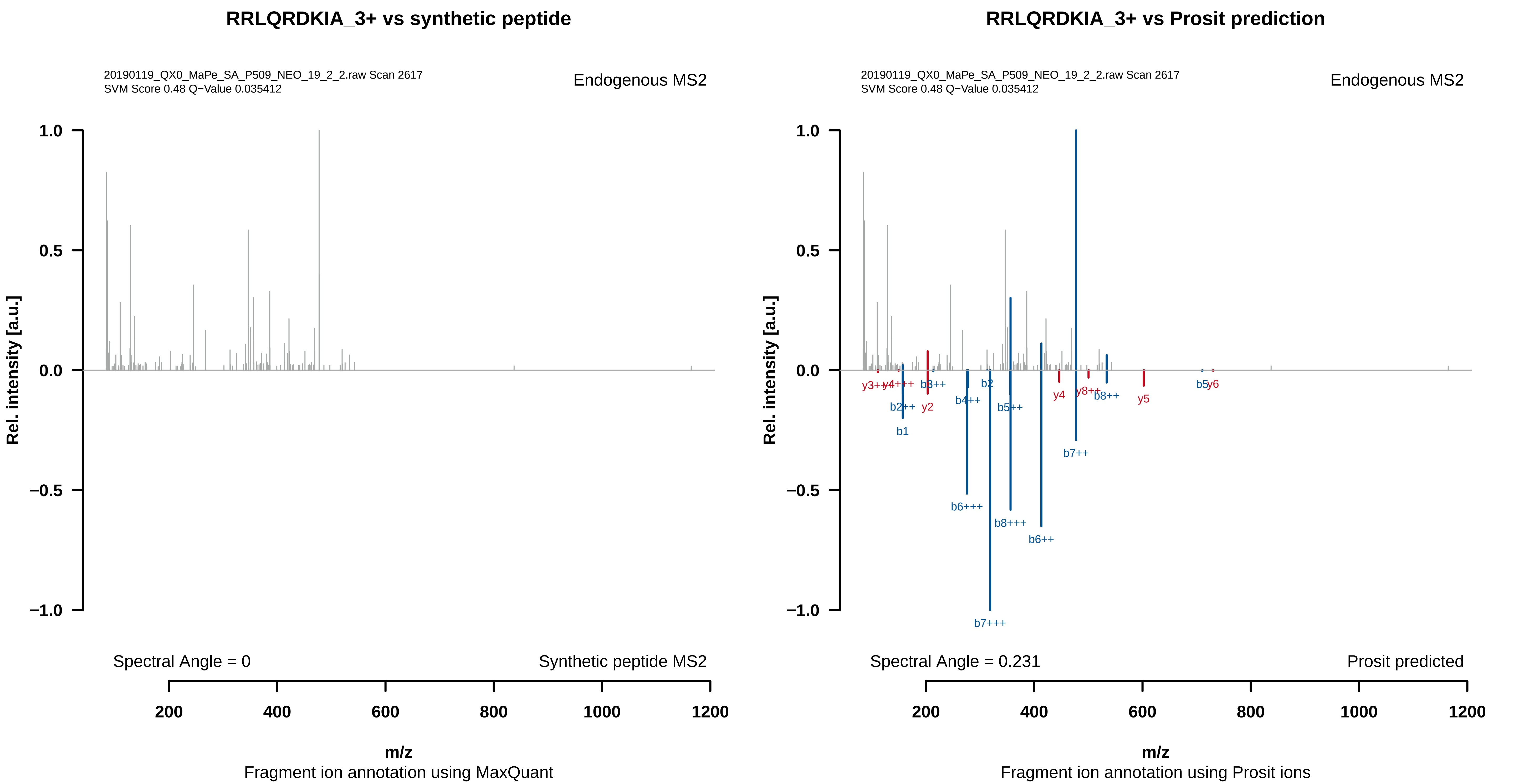
RRLQRDKIA_3+ vs synthetic peptide
20190119_QX0_MaPe_SA_P509_NEO_19_2_2.raw Scan 2617
SVM Score 0.48 Q−Value 0.035412
Endogenous MS2
Rel. intensity [a.u.]
1.0
0.5
0.0
−0.5
−1.0
Spectral Angle = 0
Synthetic peptide MS2
200
400
600
800
1000
1200
m/z
Fragment ion annotation using MaxQuant
RRLQRDKIA_3+ vs Prosit prediction
20190119_QX0_MaPe_SA_P509_NEO_19_2_2.raw Scan 2617
SVM Score 0.48 Q−Value 0.035412
Endogenous MS2
Rel. intensity [a.u.]
1.0
0.5
0.0
−0.5
−1.0
y3+++
y4+++
b3++
b2
b2++
y2
b1
b4++
b5++
y4
y8++
b8++
y5
b5
y6
b7++
b6+++
b8+++
b6++
b7+++
Spectral Angle = 0.231
Prosit predicted
200
400
600
800
1000
1200
m/z
Fragment ion annotation using Prosit ions

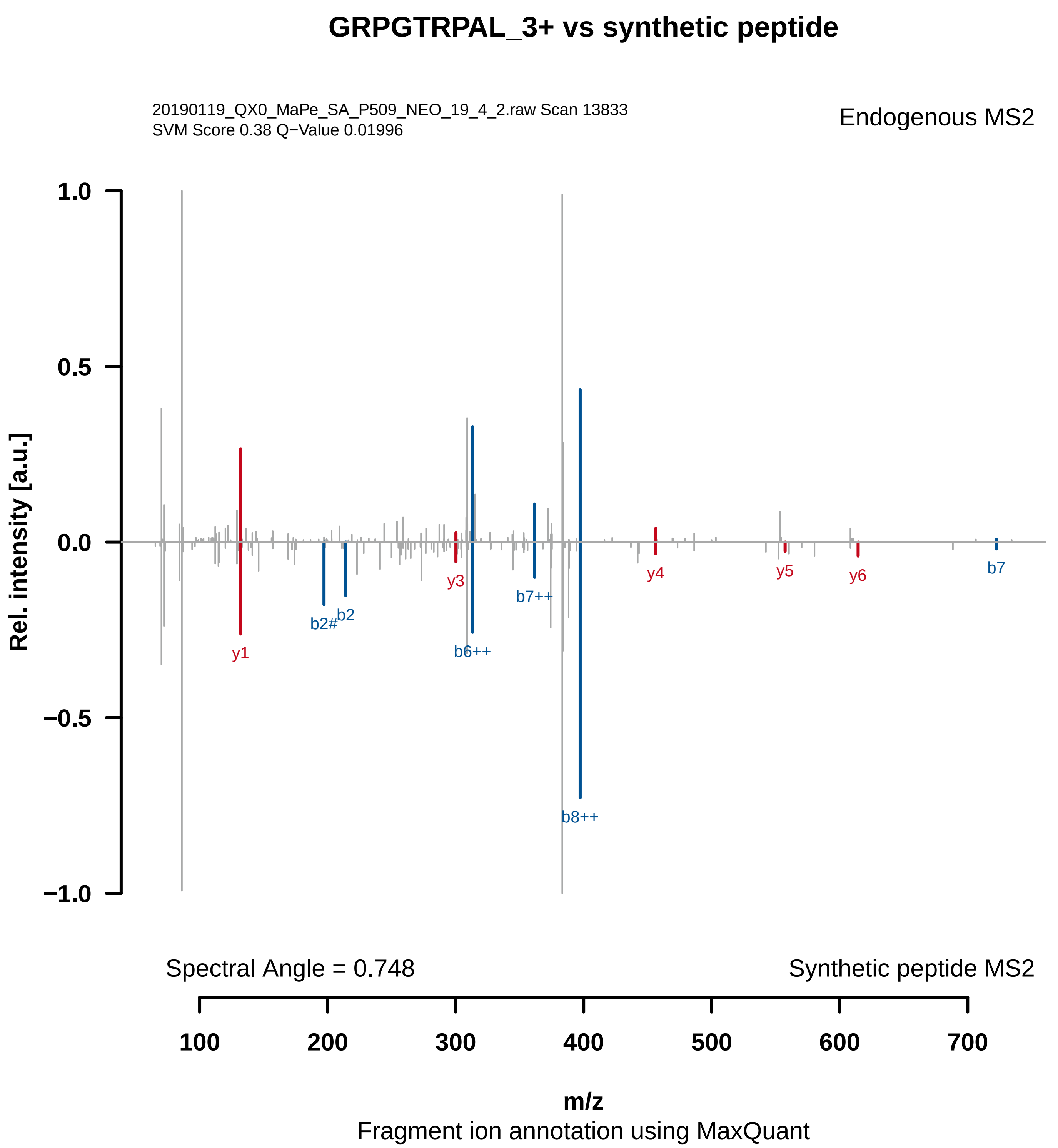
GRPGTRPAL_3+ vs synthetic peptide
20190119_QX0_MaPe_SA_P509_NEO_19_4_2.raw Scan 13833
SVM Score 0.38 Q−Value 0.01996
Endogenous MS2
Rel. intensity [a.u.]
1.0
0.5
0.0
−0.5
−1.0
y1
b2#
b2
y3
b6++
b7++
b8++
y4
y5
y6
b7
Spectral Angle = 0.748
Synthetic peptide MS2
100
200
300
400
500
600
700
m/z
Fragment ion annotation using MaxQuant

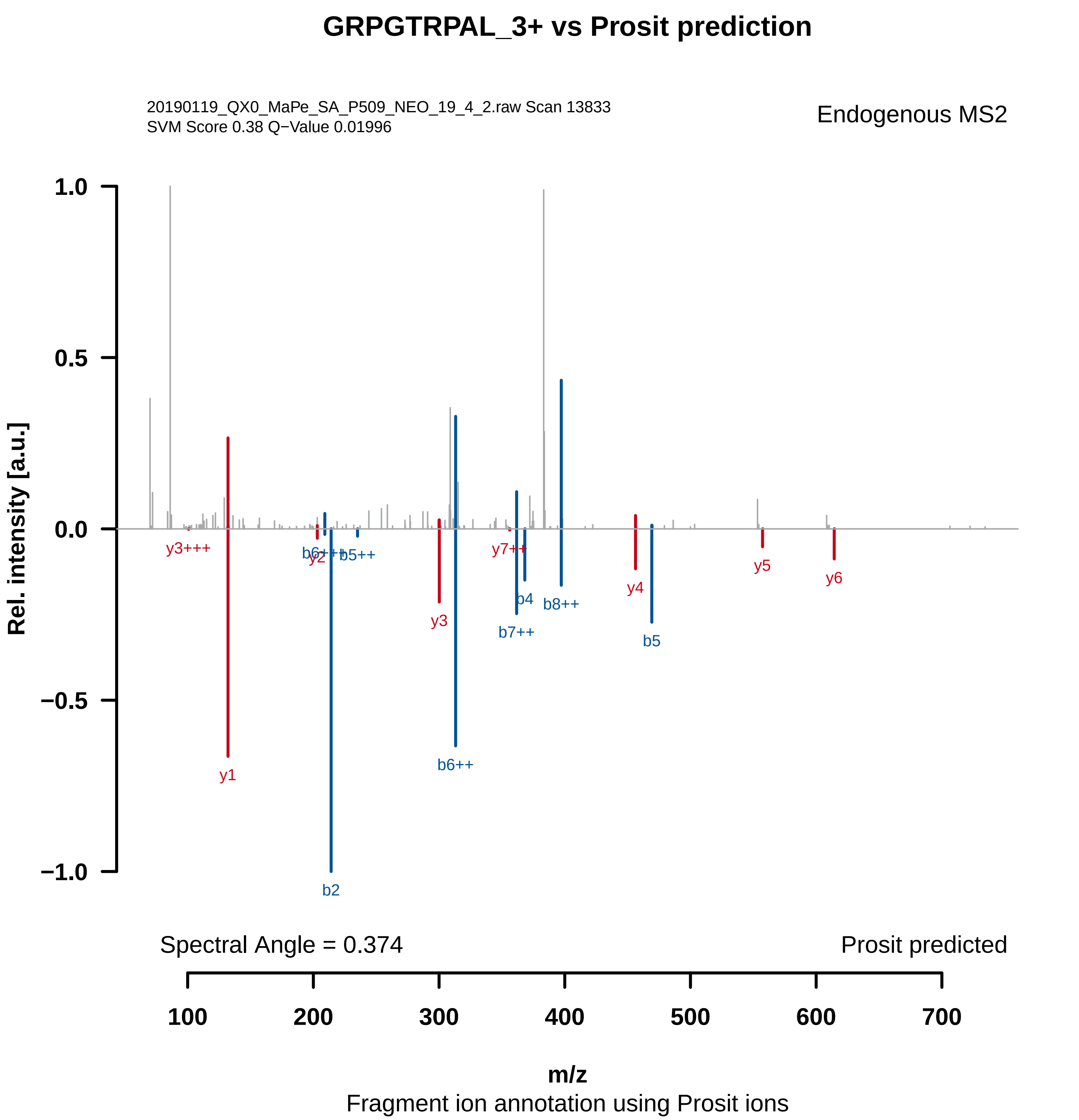
GRPGTRPAL_3+ vs Prosit prediction
20190119_QX0_MaPe_SA_P509_NEO_19_4_2.raw Scan 13833
SVM Score 0.38 Q−Value 0.01996
Endogenous MS2
Rel. intensity [a.u.]
1.0
0.5
0.0
−0.5
−1.0
y3+++
y1
b6+++
y2
b5++
b2
y3
b6++
y7++
b4
b7++
b8++
y4
b5
y5
y6
Spectral Angle = 0.374
Prosit predicted
100
200
300
400
500
600
700
m/z
Fragment ion annotation using Prosit ions

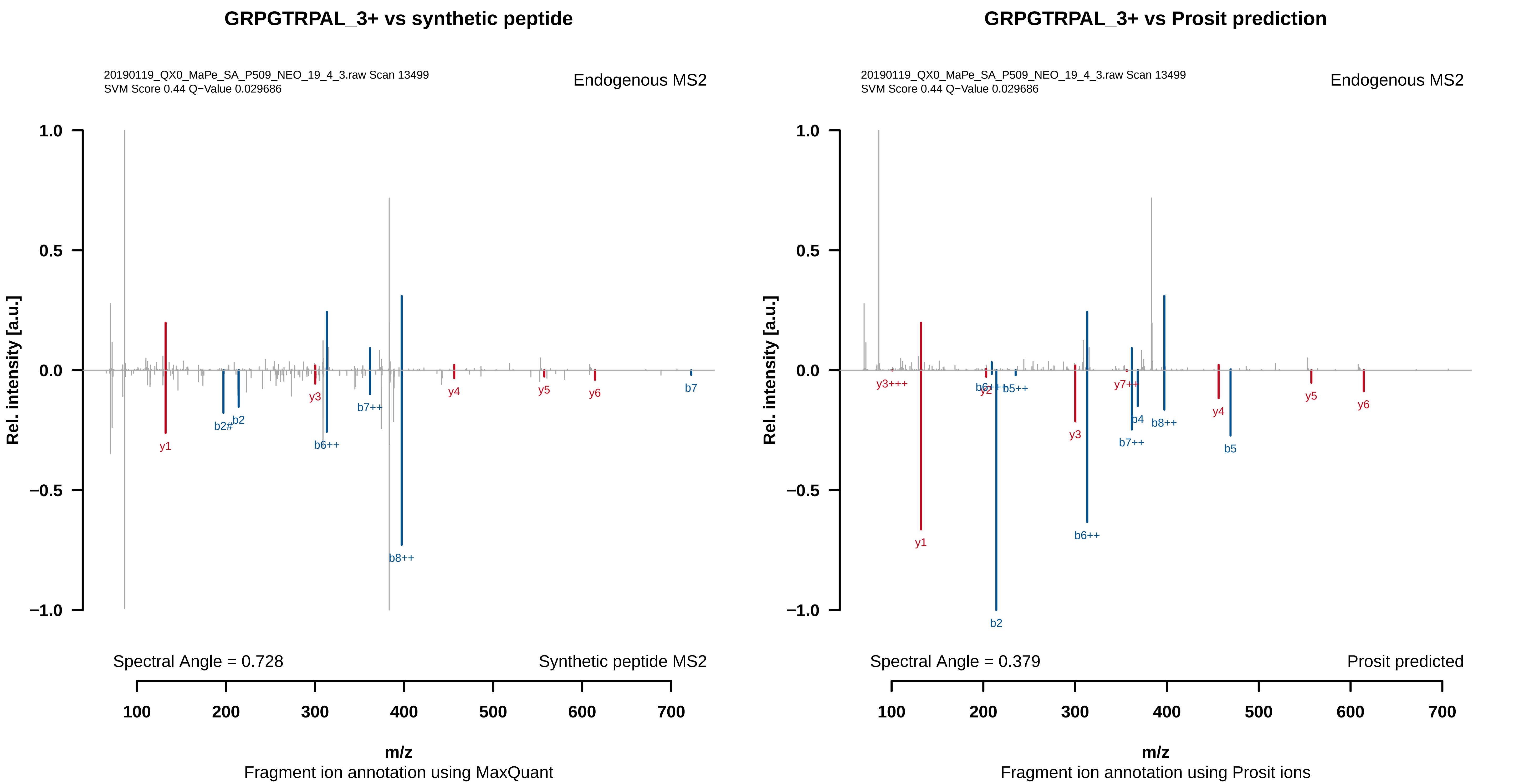
GRPGTRPAL_3+ vs synthetic peptide
20190119_QX0_MaPe_SA_P509_NEO_19_4_3.raw Scan 13499
SVM Score 0.44 Q−Value 0.029686
Endogenous MS2
Rel. intensity [a.u.]
y1
b2#
b2
y3
b6++
b7++
b8++
y4
y5
y6
b7
Spectral Angle = 0.728
Synthetic peptide MS2
m/z
Fragment ion annotation using MaxQuant
GRPGTRPAL_3+ vs Prosit prediction
20190119_QX0_MaPe_SA_P509_NEO_19_4_3.raw Scan 13499
SVM Score 0.44 Q−Value 0.029686
Endogenous MS2
Rel. intensity [a.u.]
y3+++
y1
y2
b2
b5++
y3
b6++
y7++
b4
b7++
b8++
y4
b5
y5
y6
Spectral Angle = 0.379
Prosit predicted
m/z
Fragment ion annotation using Prosit ions

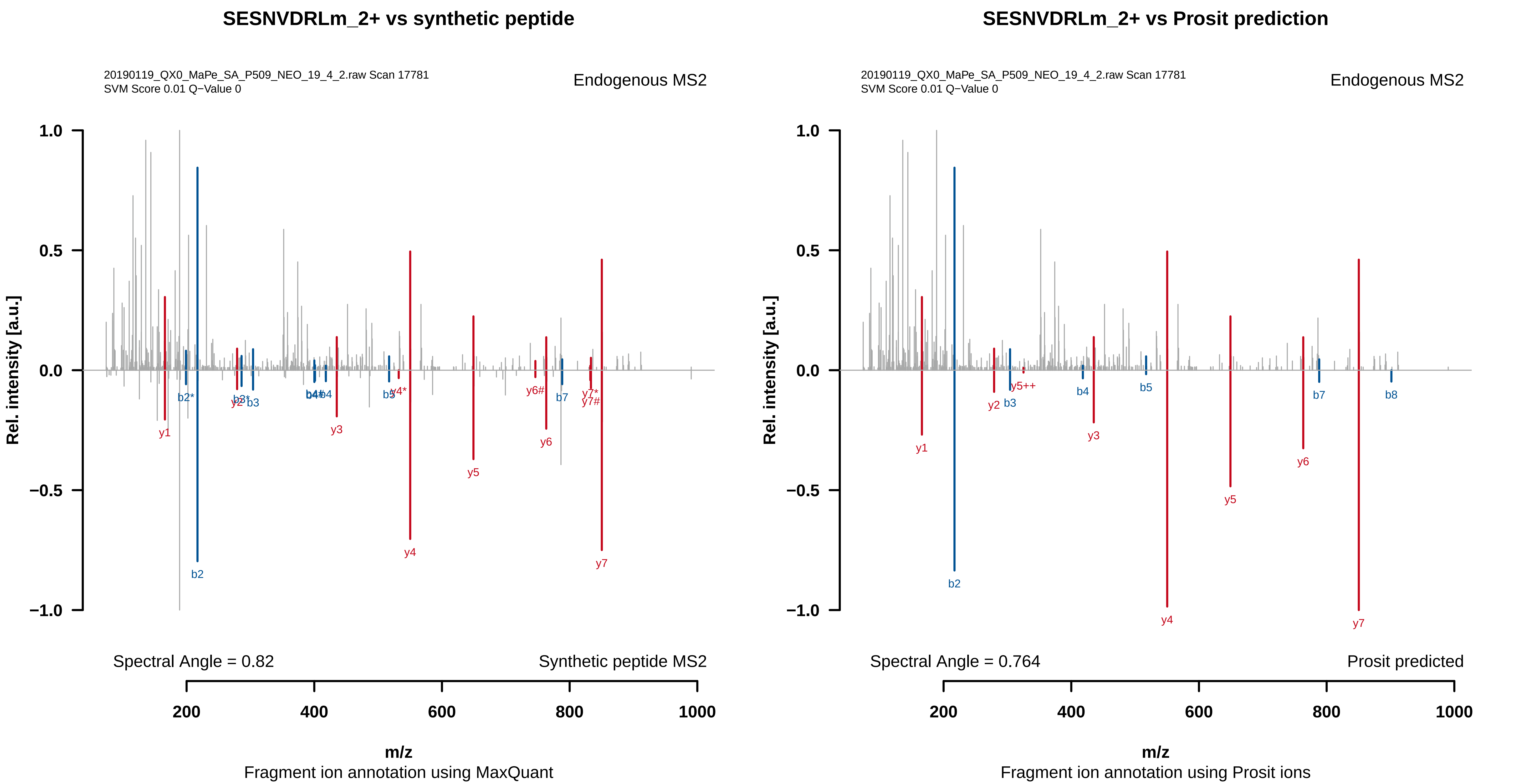

SESNVDRLm_2+ vs synthetic peptide
20190119_QX0_MaPe_SA_P509_NEO_19_4_2.raw Scan 17781
SVM Score 0.01 Q−Value 0
Endogenous MS2
Rel. intensity [a.u.]
1.0
0.5
0.0
−0.5
−1.0
y1
b2*
b2
y2
b3*
b3
b4*
b4
y3
b5
y4*
y4
y5
y6#
y6
b7
y7*
y7#
y7
Spectral Angle = 0.82
Synthetic peptide MS2
200
400
600
800
1000
m/z
Fragment ion annotation using MaxQuant
SESNVDRLm_2+ vs Prosit prediction
20190119_QX0_MaPe_SA_P509_NEO_19_4_2.raw Scan 17781
SVM Score 0.01 Q−Value 0
Endogenous MS2
Rel. intensity [a.u.]
1.0
0.5
0.0
−0.5
−1.0
y1
b2
y2
b3
y5++
b4
y3
b5
y4
y5
y6
b7
y7
b8
Spectral Angle = 0.764
Prosit predicted
200
400
600
800
1000
m/z
Fragment ion annotation using Prosit ions

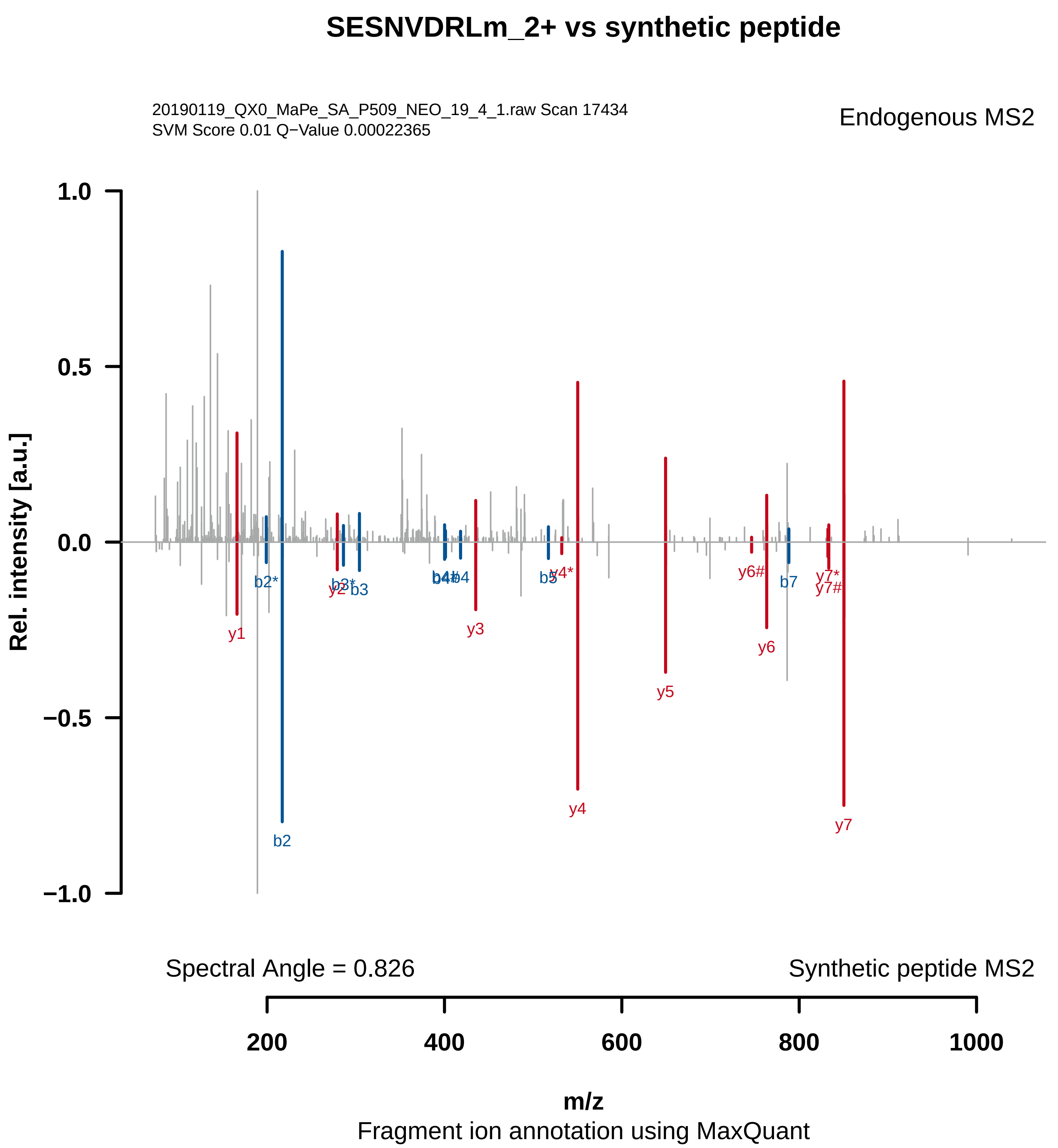
SESNVDRLm_2+ vs synthetic peptide
20190119_QX0_MaPe_SA_P509_NEO_19_4_1.raw Scan 17434
SVM Score 0.01 Q−Value 0.00022365
Endogenous MS2
Rel. intensity [a.u.]
1.0
0.5
0.0
−0.5
−1.0
y1
b2*
b2
y2
b3*
b3
b4
y3
b5
y4*
y4
y5
y6#
y6
b7
y7*
y7#
y7
Spectral Angle = 0.826
Synthetic peptide MS2
200
400
600
800
1000
m/z
Fragment ion annotation using MaxQuant

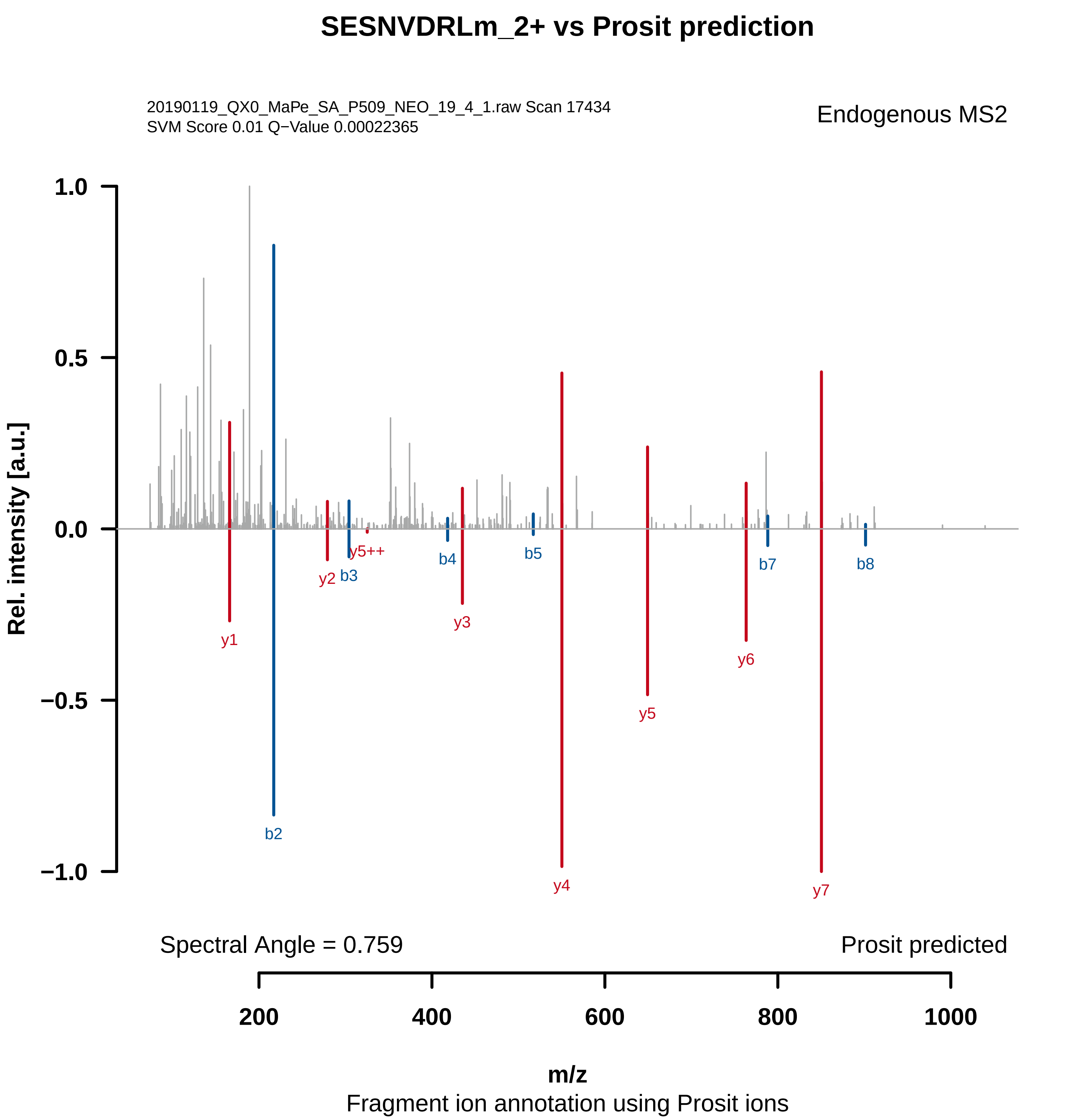
SESNVDRLm_2+ vs Prosit prediction
20190119_QX0_MaPe_SA_P509_NEO_19_4_1.raw Scan 17434
SVM Score 0.01 Q−Value 0.00022365
Endogenous MS2
Rel. intensity [a.u.]
1.0
0.5
0.0
−0.5
−1.0
y1
b2
y2
b3
y5++
b4
y3
b5
y4
y5
y6
b7
y7
b8
Spectral Angle = 0.759
Prosit predicted
200
400
600
800
1000
m/z
Fragment ion annotation using Prosit ions

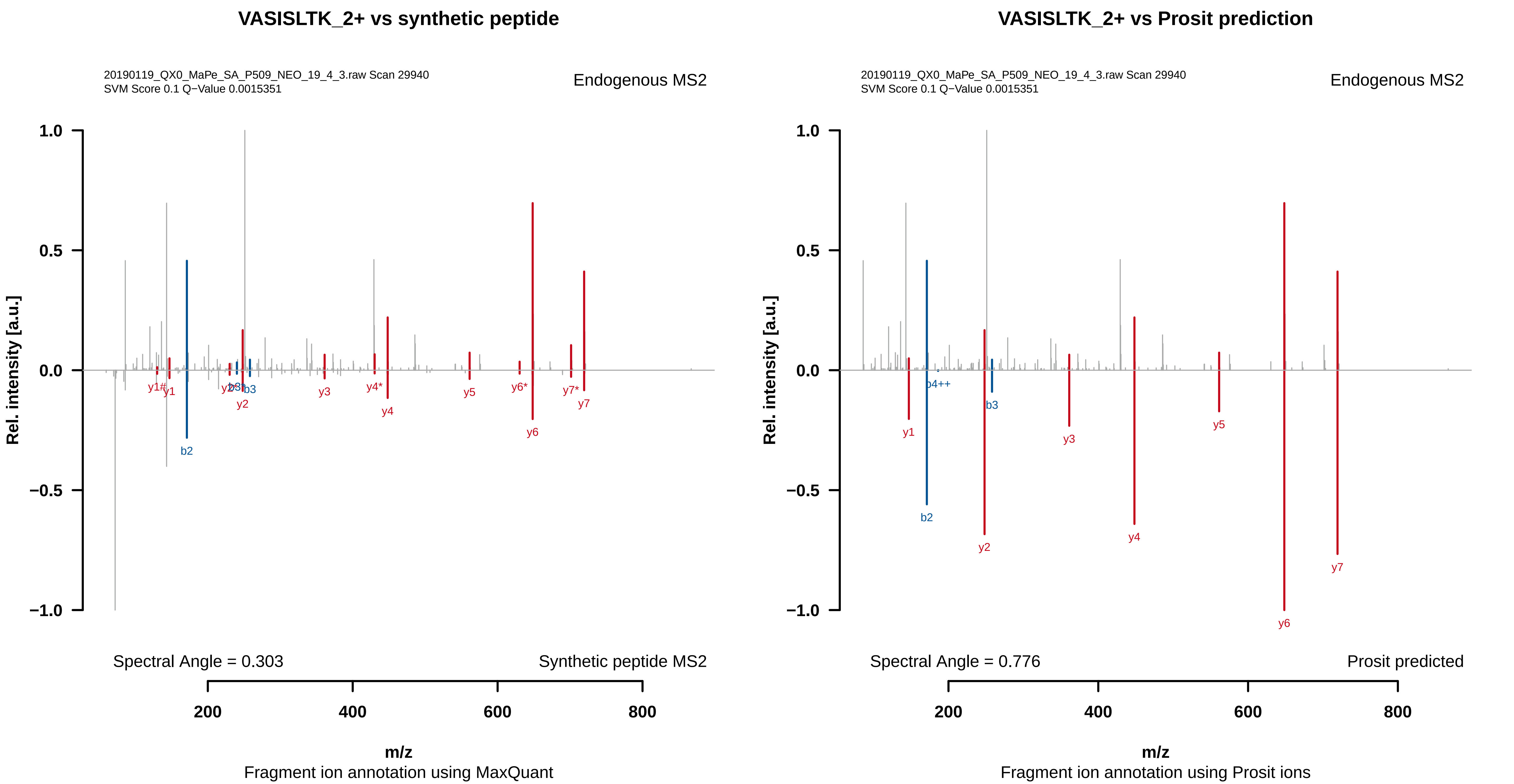
VASISLTK_2+ vs synthetic peptide
20190119_QX0_MaPe_SA_P509_NEO_19_4_3.raw Scan 29940
SVM Score 0.1 Q−Value 0.0015351
Endogenous MS2
Rel. intensity [a.u.]
1.0
0.5
0.0
−0.5
−1.0
y1#
y1
y2
b3
b3
y2
y3
y4*
y4
y5
y6*
y6
y7*
y7
b2
Spectral Angle = 0.303
Synthetic peptide MS2
200
400
600
800
m/z
Fragment ion annotation using MaxQuant
VASISLTK_2+ vs Prosit prediction
20190119_QX0_MaPe_SA_P509_NEO_19_4_3.raw Scan 29940
SVM Score 0.1 Q−Value 0.0015351
Endogenous MS2
Rel. intensity [a.u.]
1.0
0.5
0.0
−0.5
−1.0
b4++
b3
y1
y3
y5
b2
y2
y4
y6
y7
Spectral Angle = 0.776
Prosit predicted
200
400
600
800
m/z
Fragment ion annotation using Prosit ions

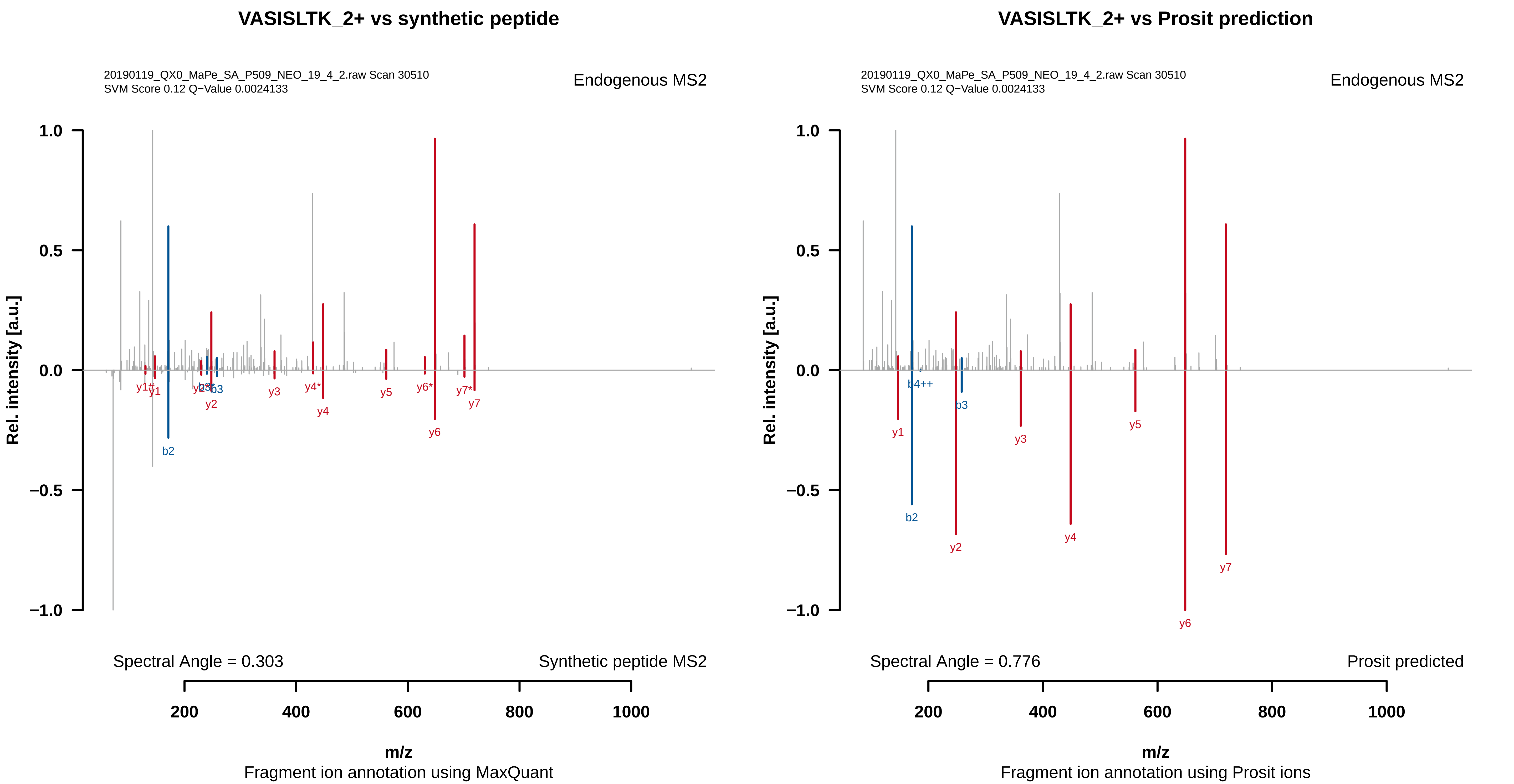

VASISLTK_2+ vs synthetic peptide
20190119_QX0_MaPe_SA_P509_NEO_19_4_2.raw Scan 30510
SVM Score 0.12 Q−Value 0.0024133
Endogenous MS2
Rel. intensity [a.u.]
1.0
0.5
0.0
−0.5
−1.0
y1#
y1
b2
y2*
b3*
b3
y2
y3
y4*
y4
y5
y6*
y6
y7*
y7
Spectral Angle = 0.303
Synthetic peptide MS2
200
400
600
800
1000
m/z
Fragment ion annotation using MaxQuant
VASISLTK_2+ vs Prosit prediction
20190119_QX0_MaPe_SA_P509_NEO_19_4_2.raw Scan 30510
SVM Score 0.12 Q−Value 0.0024133
Endogenous MS2
Rel. intensity [a.u.]
1.0
0.5
0.0
−0.5
−1.0
b4++
b3
y1
b2
y2
y3
y4
y5
y6
y7
Spectral Angle = 0.776
Prosit predicted
200
400
600
800
1000
m/z
Fragment ion annotation using Prosit ions

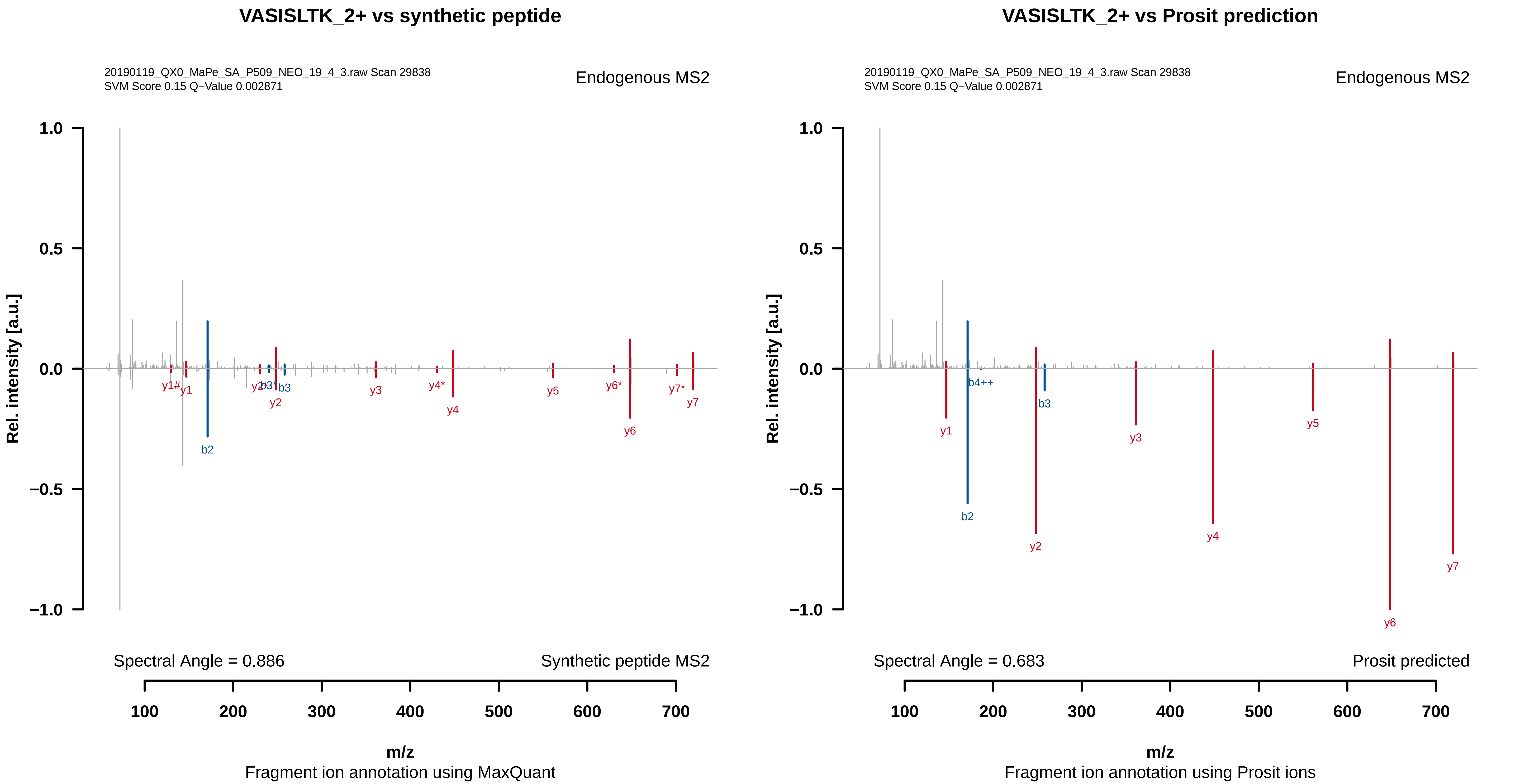
VASISLTK_2+ vs synthetic peptide
20190119_QX0_MaPe_SA_P509_NEO_19_4_3.raw Scan 29838
SVM Score 0.15 Q−Value 0.002871
Endogenous MS2
Rel. intensity [a.u.]
1.0
0.5
0.0
−0.5
−1.0
y1#
y1
y2*
b3*
b3
y2
b2
y3
y4*
y4
y5
y6*
y6
y7*
y7
Spectral Angle = 0.886
Synthetic peptide MS2
100
200
300
400
500
600
700
m/z
Fragment ion annotation using MaxQuant
VASISLTK_2+ vs Prosit prediction
20190119_QX0_MaPe_SA_P509_NEO_19_4_3.raw Scan 29838
SVM Score 0.15 Q−Value 0.002871
Endogenous MS2
Rel. intensity [a.u.]
1.0
0.5
0.0
−0.5
−1.0
b4++
b3
y1
b2
y2
y3
y4
y5
y6
y7
Spectral Angle = 0.683
Prosit predicted
100
200
300
400
500
600
700
m/z
Fragment ion annotation using Prosit ions

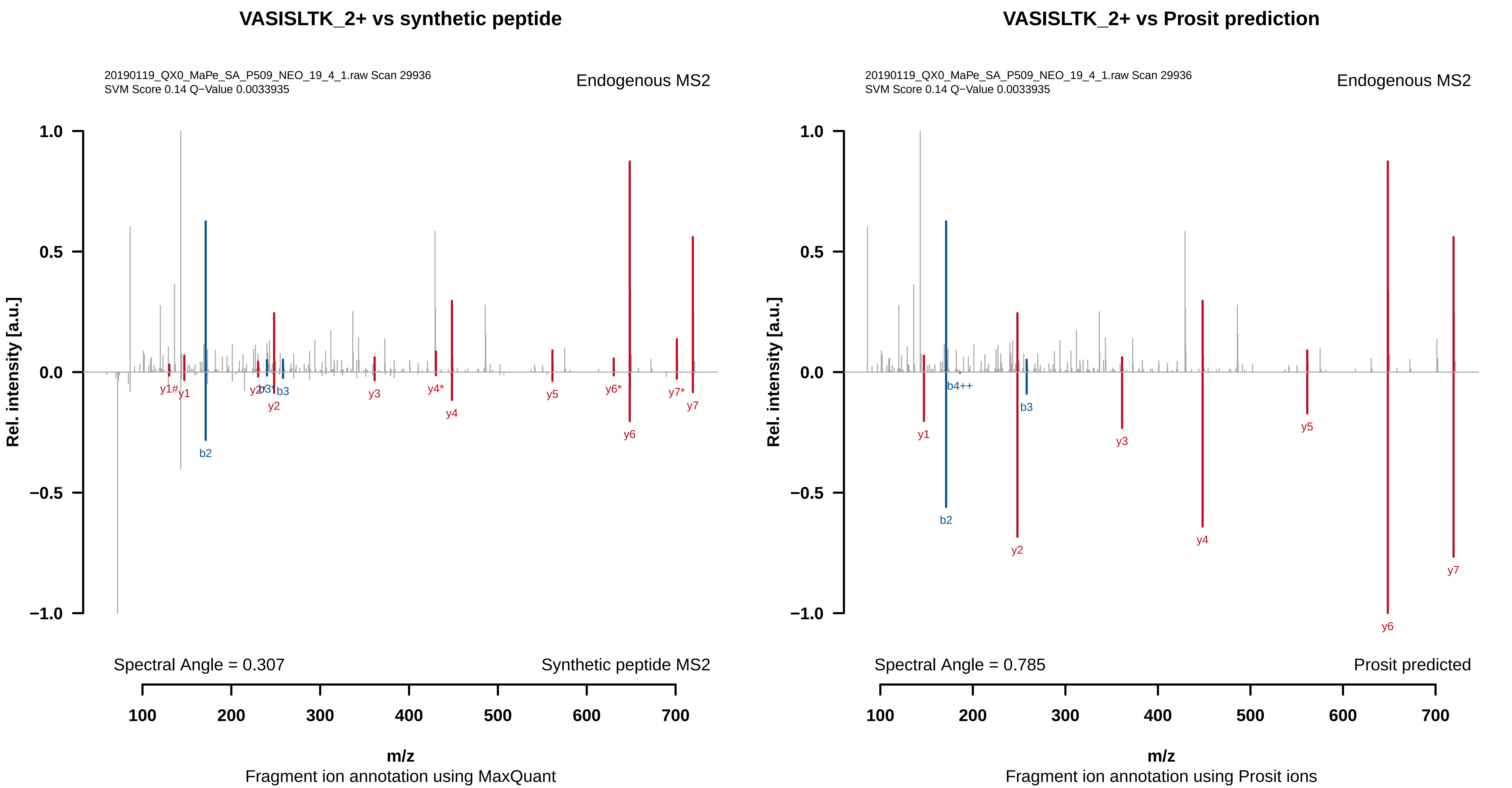

VASISLTK_2+ vs synthetic peptide
20190119_QX0_MaPe_SA_P509_NEO_19_4_1.raw Scan 29936
SVM Score 0.14 Q−Value 0.0033935
Endogenous MS2
Rel. intensity [a.u.]
Spectral Angle = 0.307
Synthetic peptide MS2
m/z
Fragment ion annotation using MaxQuant
VASISLTK_2+ vs Prosit prediction
20190119_QX0_MaPe_SA_P509_NEO_19_4_1.raw Scan 29936
SVM Score 0.14 Q−Value 0.0033935
Endogenous MS2
Rel. intensity [a.u.]
Spectral Angle = 0.785
Prosit predicted
m/z
Fragment ion annotation using Prosit ions

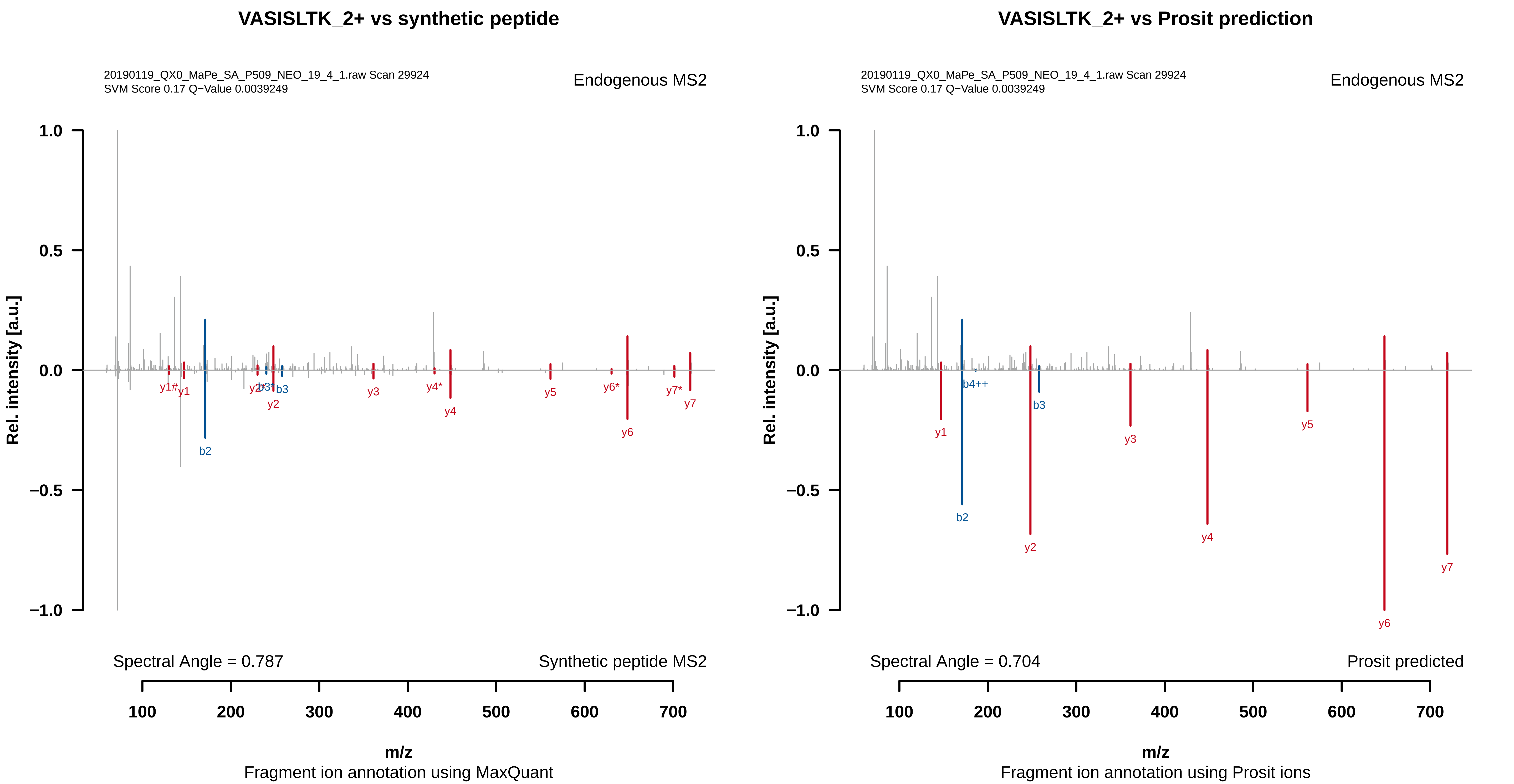
VASISLTK_2+ vs synthetic peptide
20190119_QX0_MaPe_SA_P509_NEO_19_4_1.raw Scan 29924
SVM Score 0.17 Q−Value 0.0039249
Endogenous MS2
Rel. intensity [a.u.]
1.0
0.5
0.0
−0.5
−1.0
y1#
y1
b2
y2
b3*
b3
y2
y3
y4*
y4
y5
y6*
y6
y7*
y7
Spectral Angle = 0.787
Synthetic peptide MS2
100
200
300
400
500
600
700
m/z
Fragment ion annotation using MaxQuant
VASISLTK_2+ vs Prosit prediction
20190119_QX0_MaPe_SA_P509_NEO_19_4_1.raw Scan 29924
SVM Score 0.17 Q−Value 0.0039249
Endogenous MS2
Rel. intensity [a.u.]
1.0
0.5
0.0
−0.5
−1.0
b4++
b3
y1
b2
y2
y3
y4
y5
y6
y7
Spectral Angle = 0.704
Prosit predicted
100
200
300
400
500
600
700
m/z
Fragment ion annotation using Prosit ions

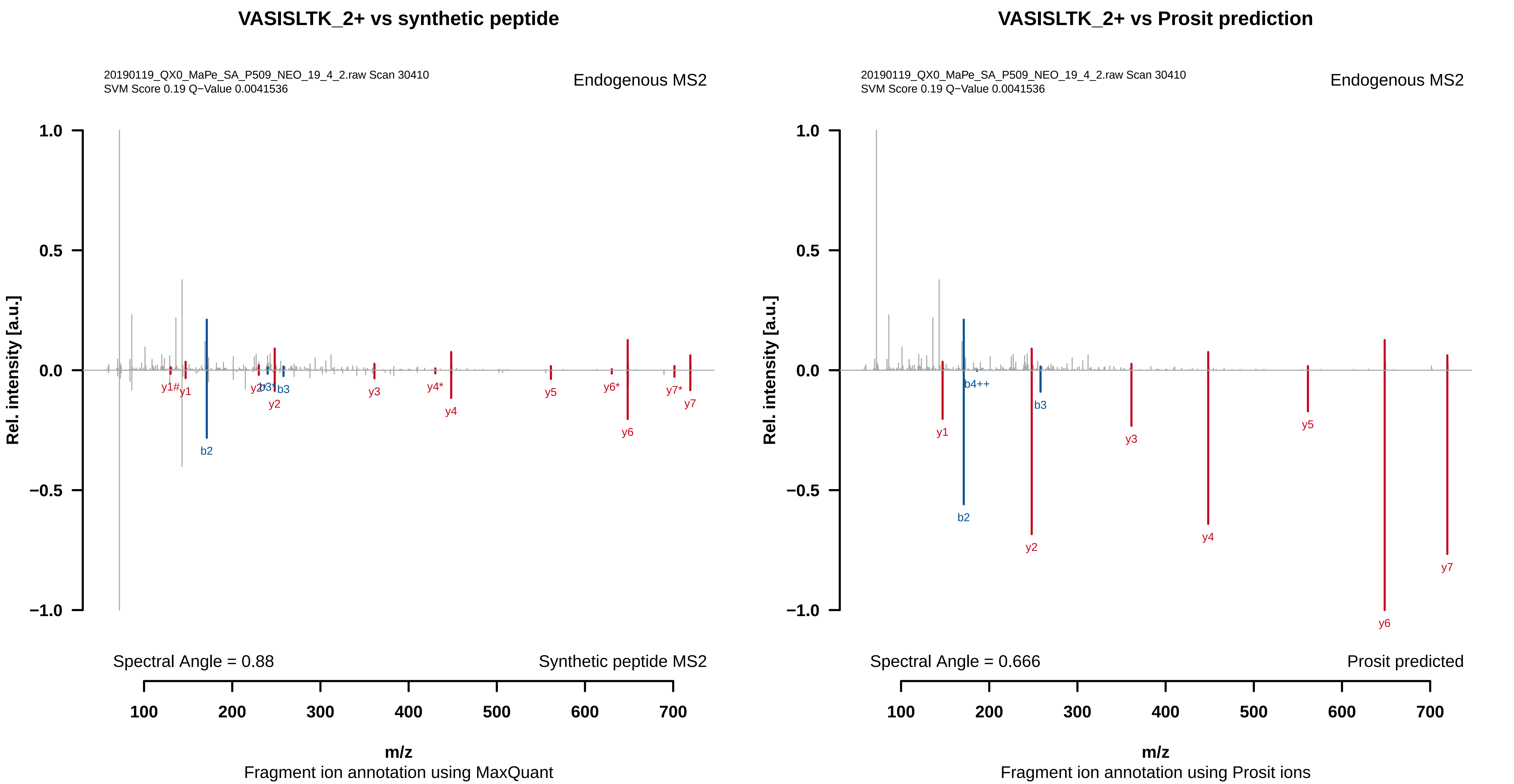

VASISLTK_2+ vs synthetic peptide
20190119_QX0_MaPe_SA_P509_NEO_19_4_2.raw Scan 30410
SVM Score 0.19 Q−Value 0.0041536
Endogenous MS2
Rel. intensity [a.u.]
1.0
0.5
0.0
−0.5
−1.0
y1# y1 b2 y2 b3* b3 y2 y3 y4* y4 y5 y6* y6 y7* y7
Spectral Angle = 0.88
Synthetic peptide MS2
100 200 300 400 500 600 700
m/z
Fragment ion annotation using MaxQuant
VASISLTK_2+ vs Prosit prediction
20190119_QX0_MaPe_SA_P509_NEO_19_4_2.raw Scan 30410
SVM Score 0.19 Q−Value 0.0041536
Endogenous MS2
Rel. intensity [a.u.]
1.0
0.5
0.0
−0.5
−1.0
y1 b4++ b2 b3 y2 y3 y4 y5 y6 y7
Spectral Angle = 0.666
Prosit predicted
100 200 300 400 500 600 700
m/z
Fragment ion annotation using Prosit ions

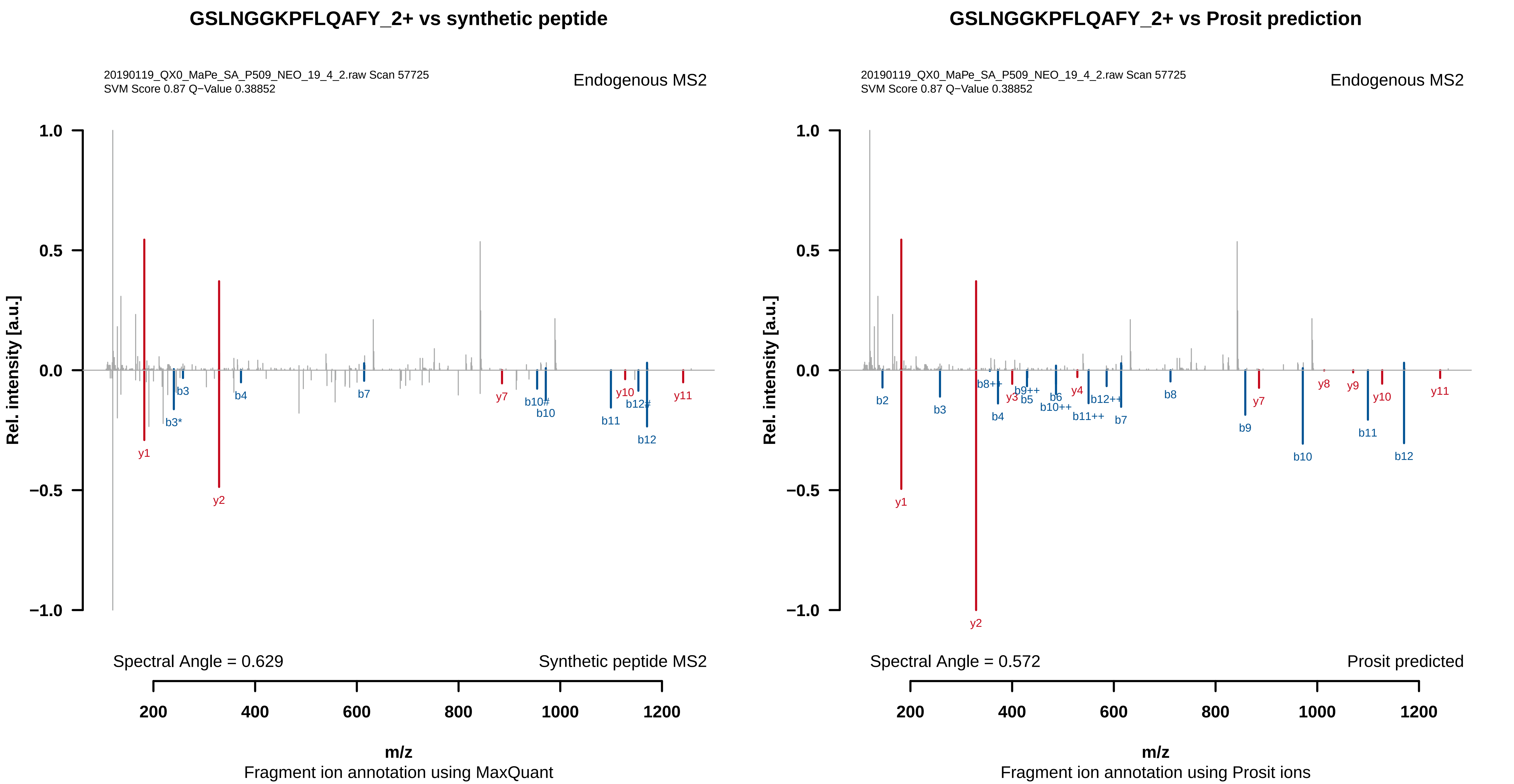
GSLNGGKPFLQAFY_2+ vs synthetic peptide
20190119_QX0_MaPe_SA_P509_NEO_19_4_2.raw Scan 57725
SVM Score 0.87 Q−Value 0.38852
Endogenous MS2
Rel. intensity [a.u.]
Spectral Angle = 0.629
Synthetic peptide MS2
m/z
Fragment ion annotation using MaxQuant
GSLNGGKPFLQAFY_2+ vs Prosit prediction
20190119_QX0_MaPe_SA_P509_NEO_19_4_2.raw Scan 57725
SVM Score 0.87 Q−Value 0.38852
Endogenous MS2
Rel. intensity [a.u.]
Spectral Angle = 0.572
Prosit predicted
m/z
Fragment ion annotation using Prosit ions

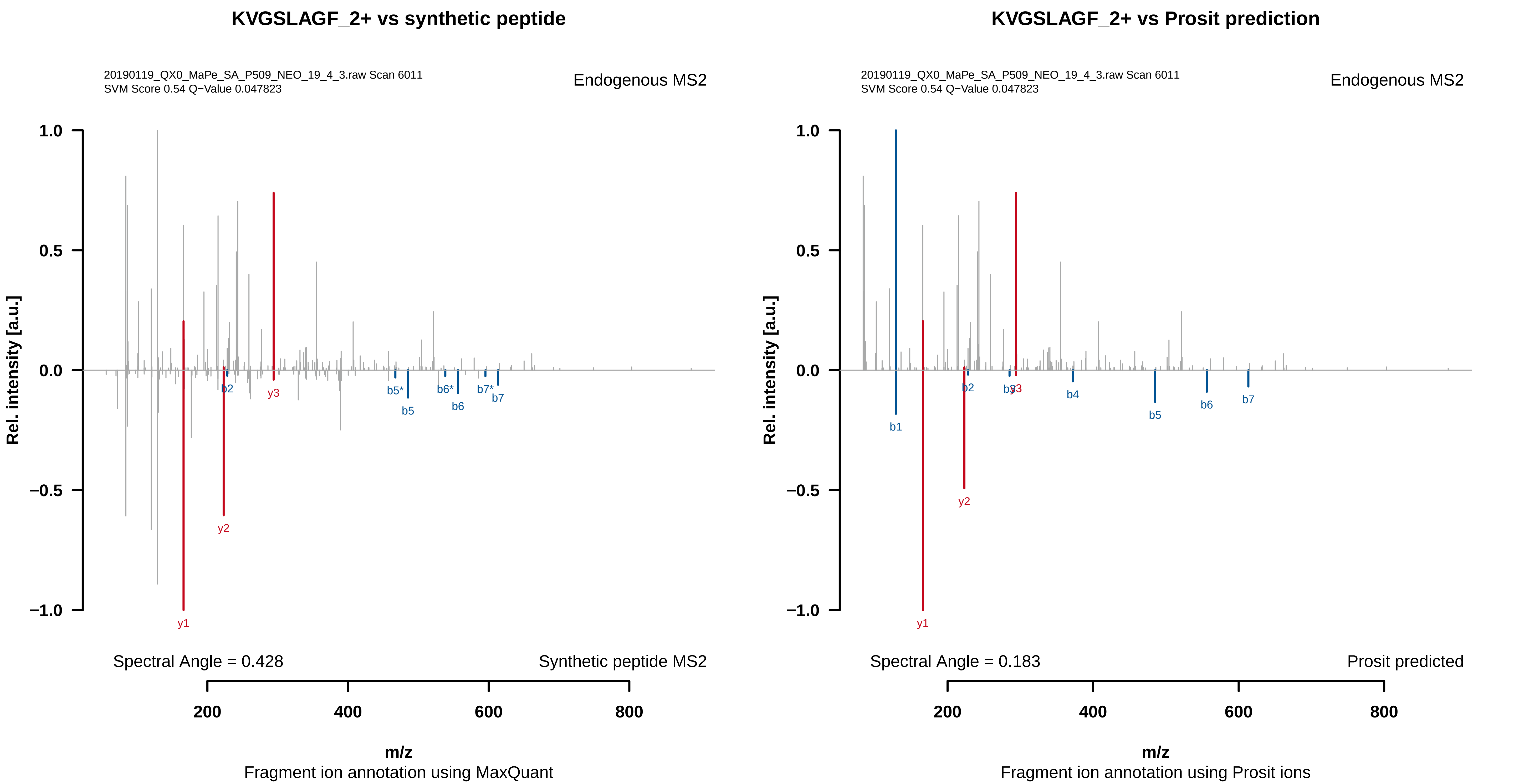

KVGSLAGF_2+ vs synthetic peptide
20190119_QX0_MaPe_SA_P509_NEO_19_4_3.raw Scan 6011
SVM Score 0.54 Q−Value 0.047823
Endogenous MS2
Rel. intensity [a.u.]
1.0
0.5
0.0
−0.5
−1.0
y1
y2
y3
b2
b5*
b5
b6*
b6
b7*
b7
Spectral Angle = 0.428
Synthetic peptide MS2
200
400
600
800
m/z
Fragment ion annotation using MaxQuant
KVGSLAGF_2+ vs Prosit prediction
20190119_QX0_MaPe_SA_P509_NEO_19_4_3.raw Scan 6011
SVM Score 0.54 Q−Value 0.047823
Endogenous MS2
Rel. intensity [a.u.]
1.0
0.5
0.0
−0.5
−1.0
b1
y1
y2
b2
b3
y3
b4
b5
b6
b7
Spectral Angle = 0.183
Prosit predicted
200
400
600
800
m/z
Fragment ion annotation using Prosit ions

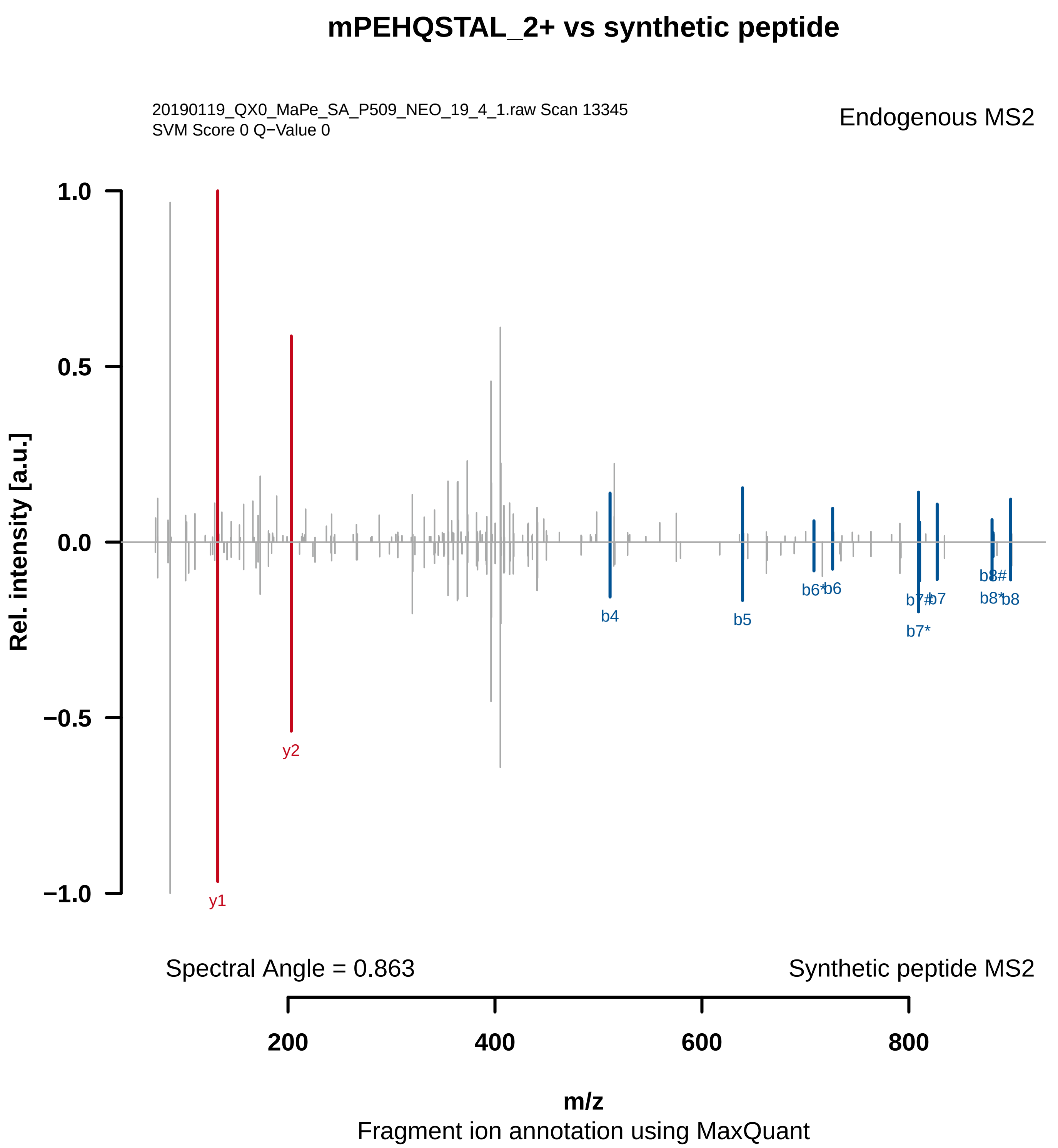
mPEHQSTAL_2+ vs synthetic peptide
20190119_QX0_MaPe_SA_P509_NEO_19_4_1.raw Scan 13345
SVM Score 0 Q−Value 0
Endogenous MS2
Rel. intensity [a.u.]
1.0
0.5
0.0
−0.5
−1.0
y1
y2
b4
b5
b6* b6
b7# b7
b7*
b8#
b8* b8
Spectral Angle = 0.863
Synthetic peptide MS2
200
400
600
800
m/z
Fragment ion annotation using MaxQuant

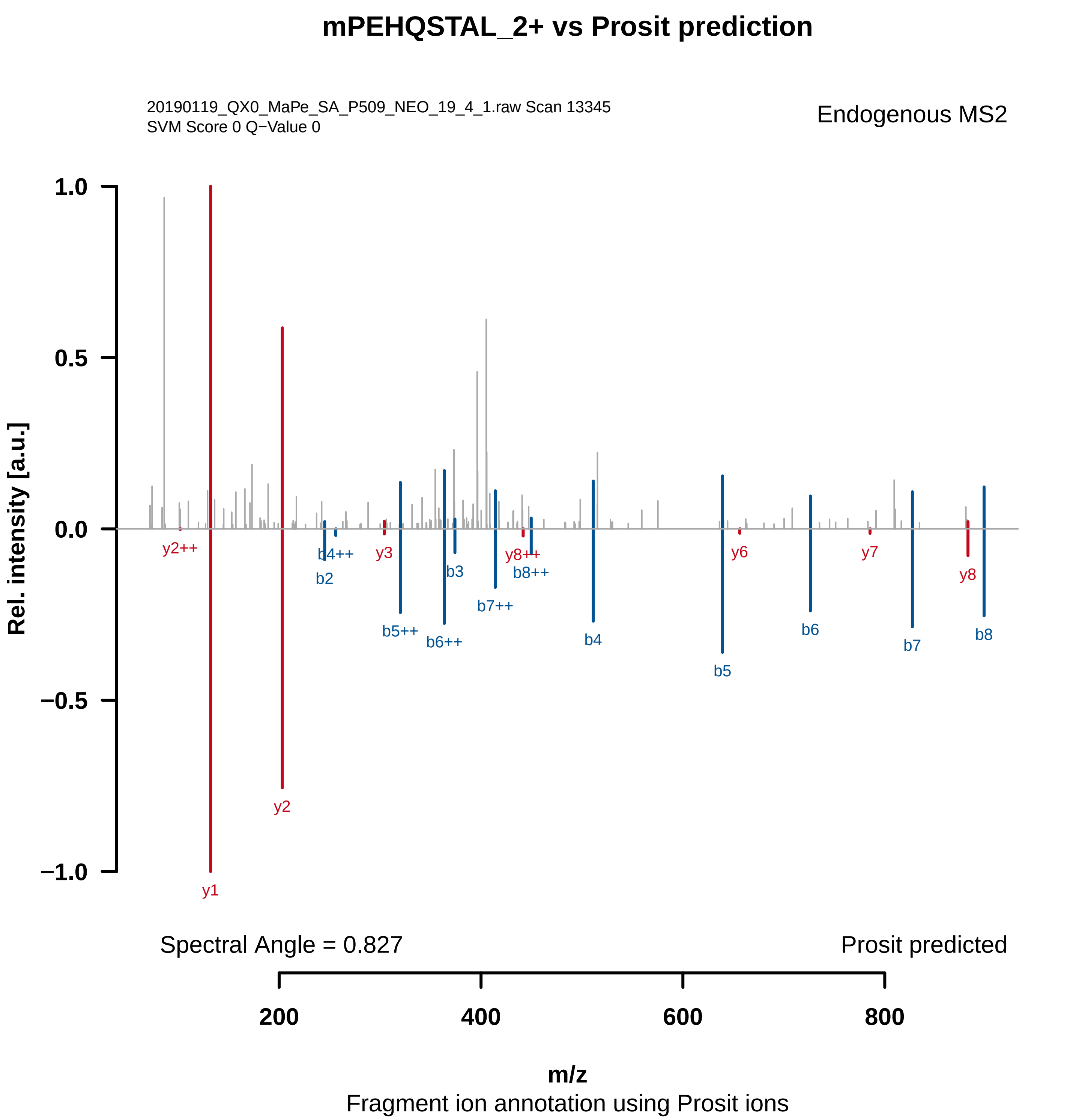
mPEHQSTAL_2+ vs Prosit prediction
20190119_QX0_MaPe_SA_P509_NEO_19_4_1.raw Scan 13345
SVM Score 0 Q−Value 0
Endogenous MS2
Rel. intensity [a.u.]
1.0
0.5
0.0
−0.5
−1.0
y2++
y1
y2
b4++
b2
y3
b5++
b3
b6++
b7++
y8++
b8++
b4
b5
y6
b6
y7
b7
y8
b8
Spectral Angle = 0.827
Prosit predicted
200
400
600
800
m/z
Fragment ion annotation using Prosit ions

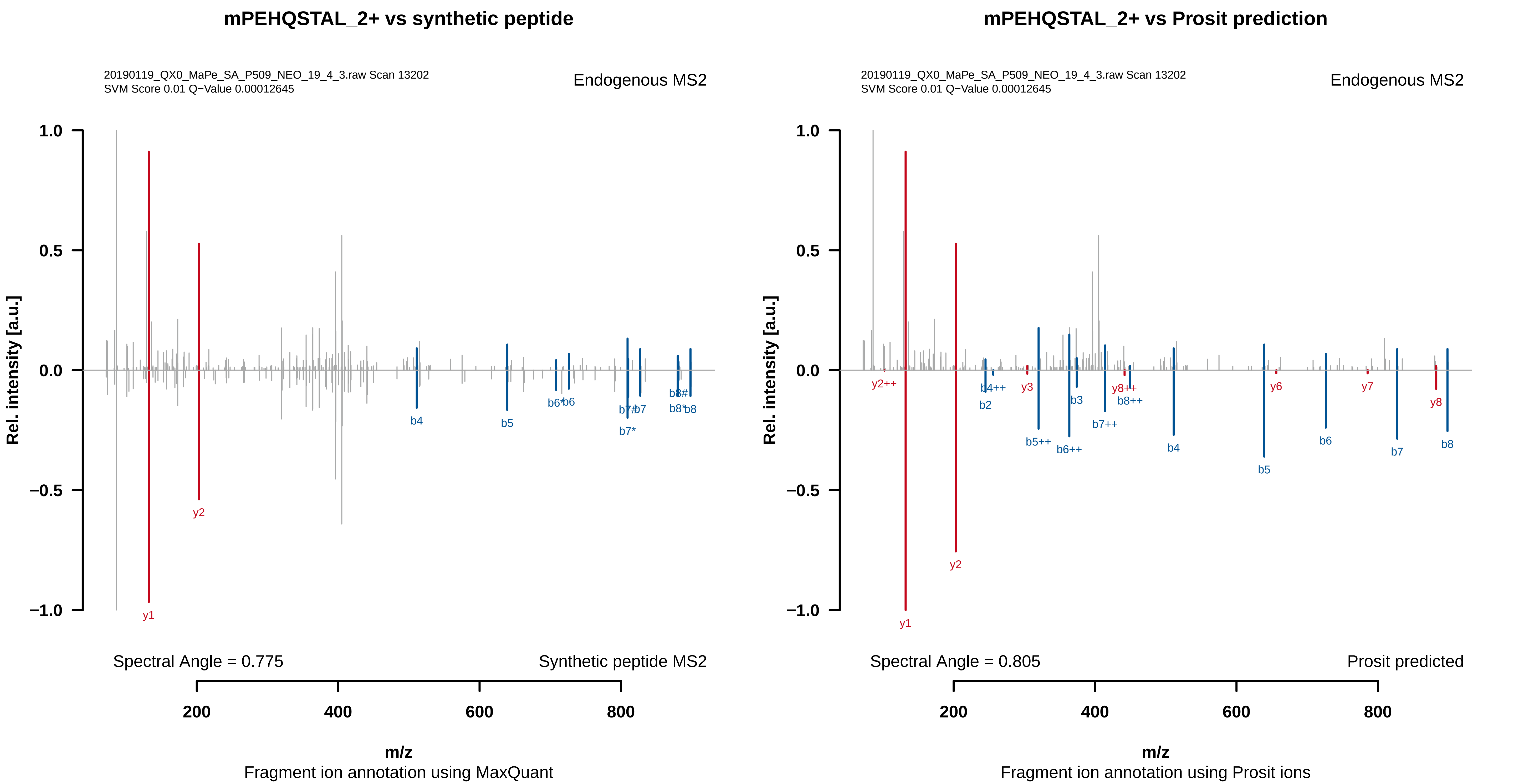
mPEHQSTAL_2+ vs synthetic peptide
20190119_QX0_MaPe_SA_P509_NEO_19_4_3.raw Scan 13202
SVM Score 0.01 Q−Value 0.00012645
Endogenous MS2
Rel. intensity [a.u.]
Spectral Angle = 0.775
Synthetic peptide MS2
m/z
Fragment ion annotation using MaxQuant
mPEHQSTAL_2+ vs Prosit prediction
20190119_QX0_MaPe_SA_P509_NEO_19_4_3.raw Scan 13202
SVM Score 0.01 Q−Value 0.00012645
Endogenous MS2
Rel. intensity [a.u.]
Spectral Angle = 0.805
Prosit predicted
m/z
Fragment ion annotation using Prosit ions

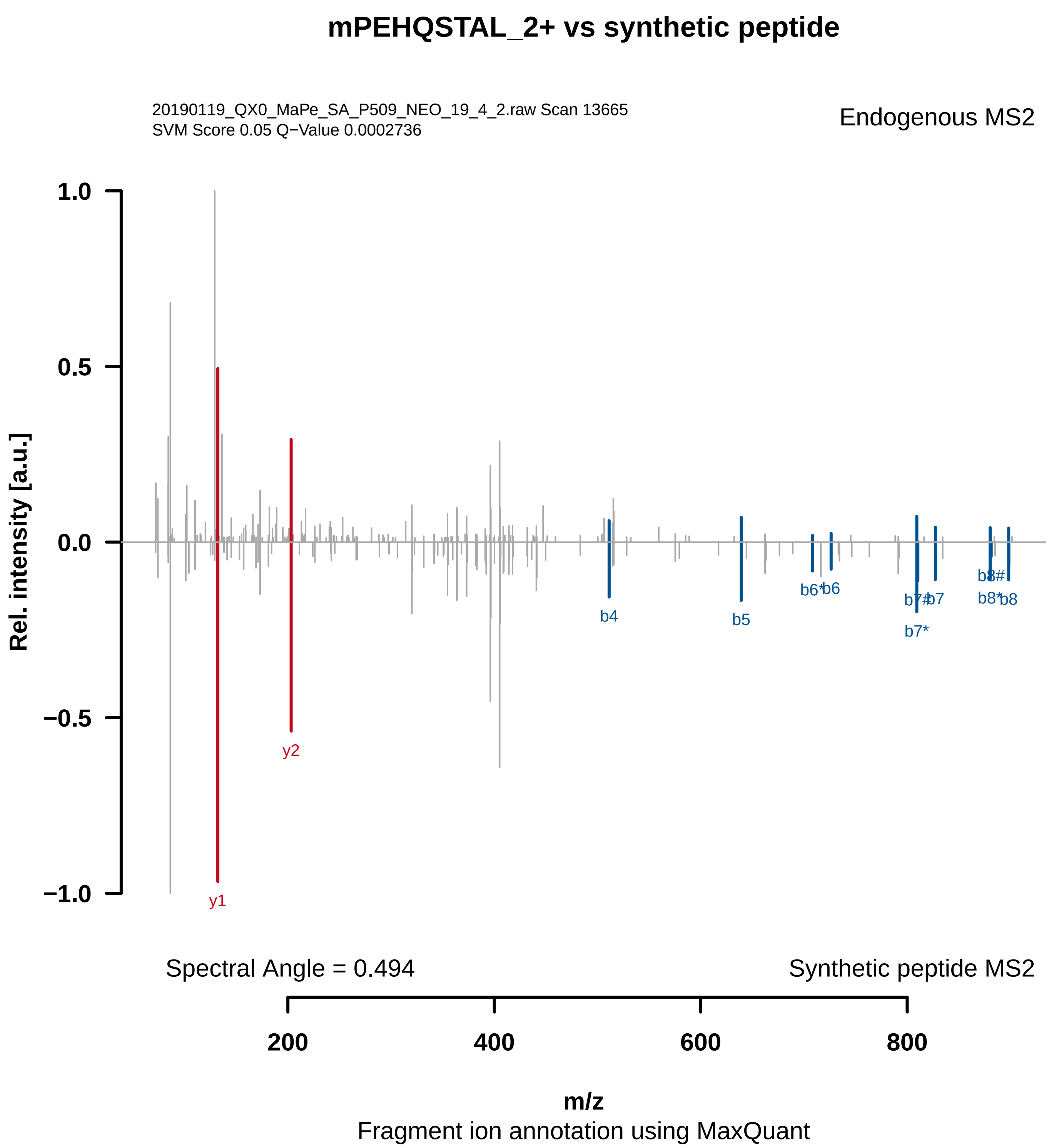
mPEHQSTAL_2+ vs synthetic peptide
20190119_QX0_MaPe_SA_P509_NEO_19_4_2.raw Scan 13665
SVM Score 0.05 Q−Value 0.0002736
Endogenous MS2
Rel. intensity [a.u.]
1.0
0.5
0.0
−0.5
−1.0
y1
y2
b4
b5
b6*
b6
b7#
b7
b7*
b8#
b8*
b8
Spectral Angle = 0.494
Synthetic peptide MS2
200
400
600
800
m/z
Fragment ion annotation using MaxQuant

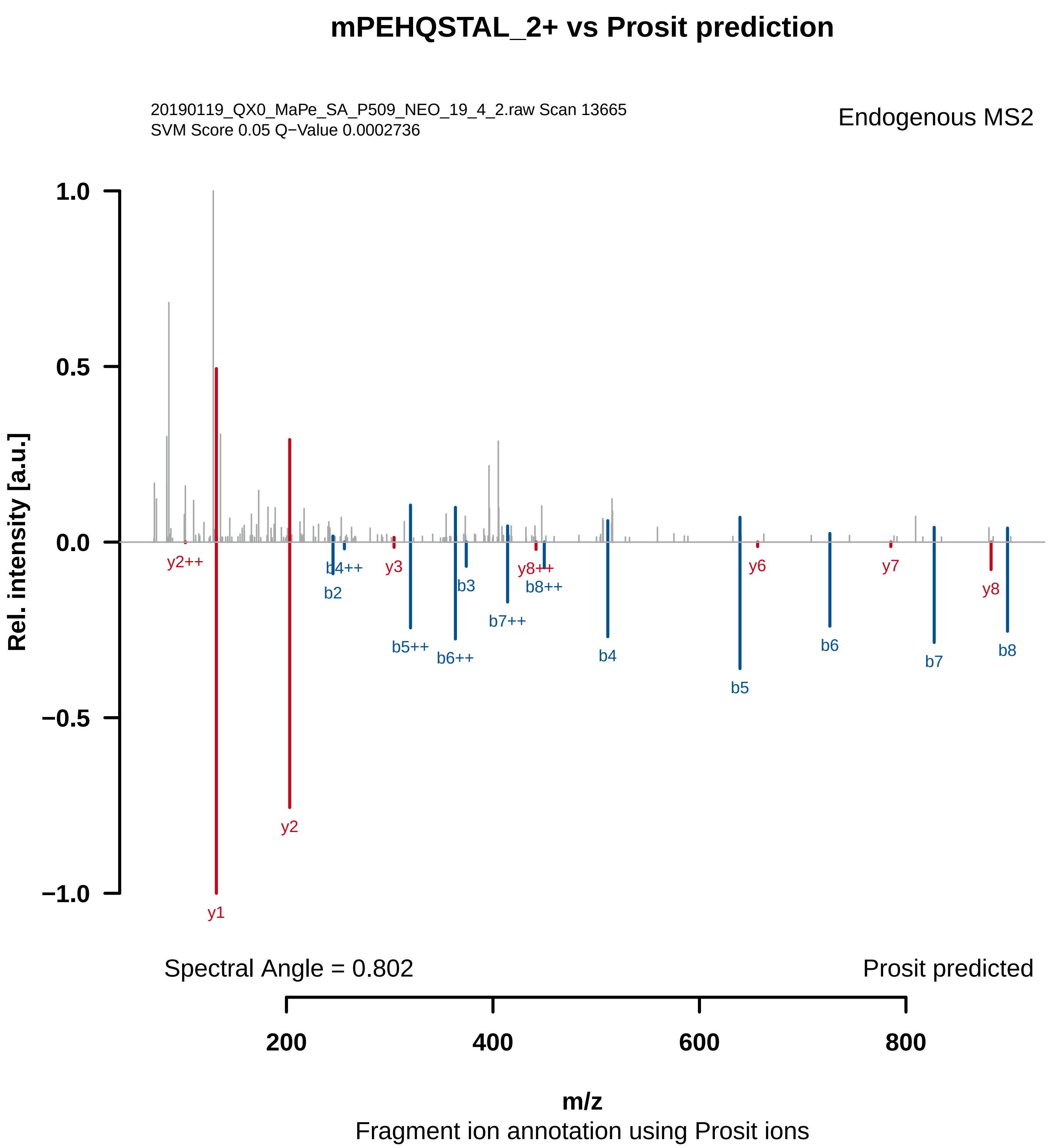
mPEHQSTAL_2+ vs Prosit prediction
20190119_QX0_MaPe_SA_P509_NEO_19_4_2.raw Scan 13665
SVM Score 0.05 Q−Value 0.0002736
Endogenous MS2
Rel. intensity [a.u.]
1.0
0.5
0.0
−0.5
−1.0
y2++
y1
y2
b4++
b2
y3
b5++
b6++
b3
b7++
y8++
b8++
b4
b5
y6
b6
y7
b7
y8
b8
Spectral Angle = 0.802
Prosit predicted
200
400
600
800
m/z
Fragment ion annotation using Prosit ions

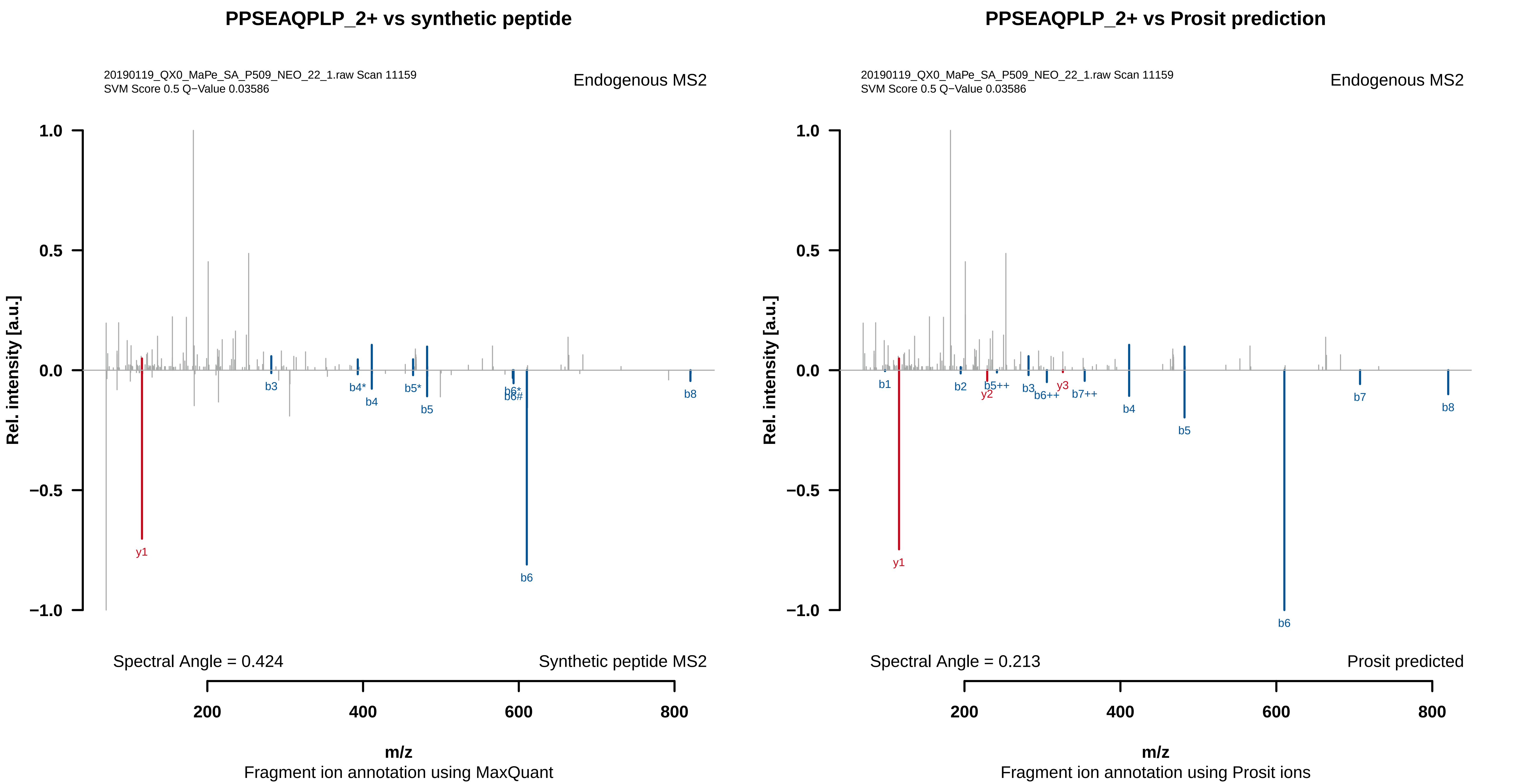
PPSEAQPLP_2+ vs synthetic peptide
20190119_QX0_MaPe_SA_P509_NEO_22_1.raw Scan 11159
SVM Score 0.5 Q−Value 0.03586
Endogenous MS2
Rel. intensity [a.u.]
1.0
0.5
0.0
−0.5
−1.0
y1
b3
b4*
b4
b5*
b5
b6*
b6#
b6
b8
Spectral Angle = 0.424
Synthetic peptide MS2
200
400
600
800
m/z
Fragment ion annotation using MaxQuant
PPSEAQPLP_2+ vs Prosit prediction
20190119_QX0_MaPe_SA_P509_NEO_22_1.raw Scan 11159
SVM Score 0.5 Q−Value 0.03586
Endogenous MS2
Rel. intensity [a.u.]
1.0
0.5
0.0
−0.5
−1.0
b1
y1
b2
y2
b5++
b3
b6++
y3
b7++
b4
b5
b6
b7
b8
Spectral Angle = 0.213
Prosit predicted
200
400
600
800
m/z
Fragment ion annotation using Prosit ions

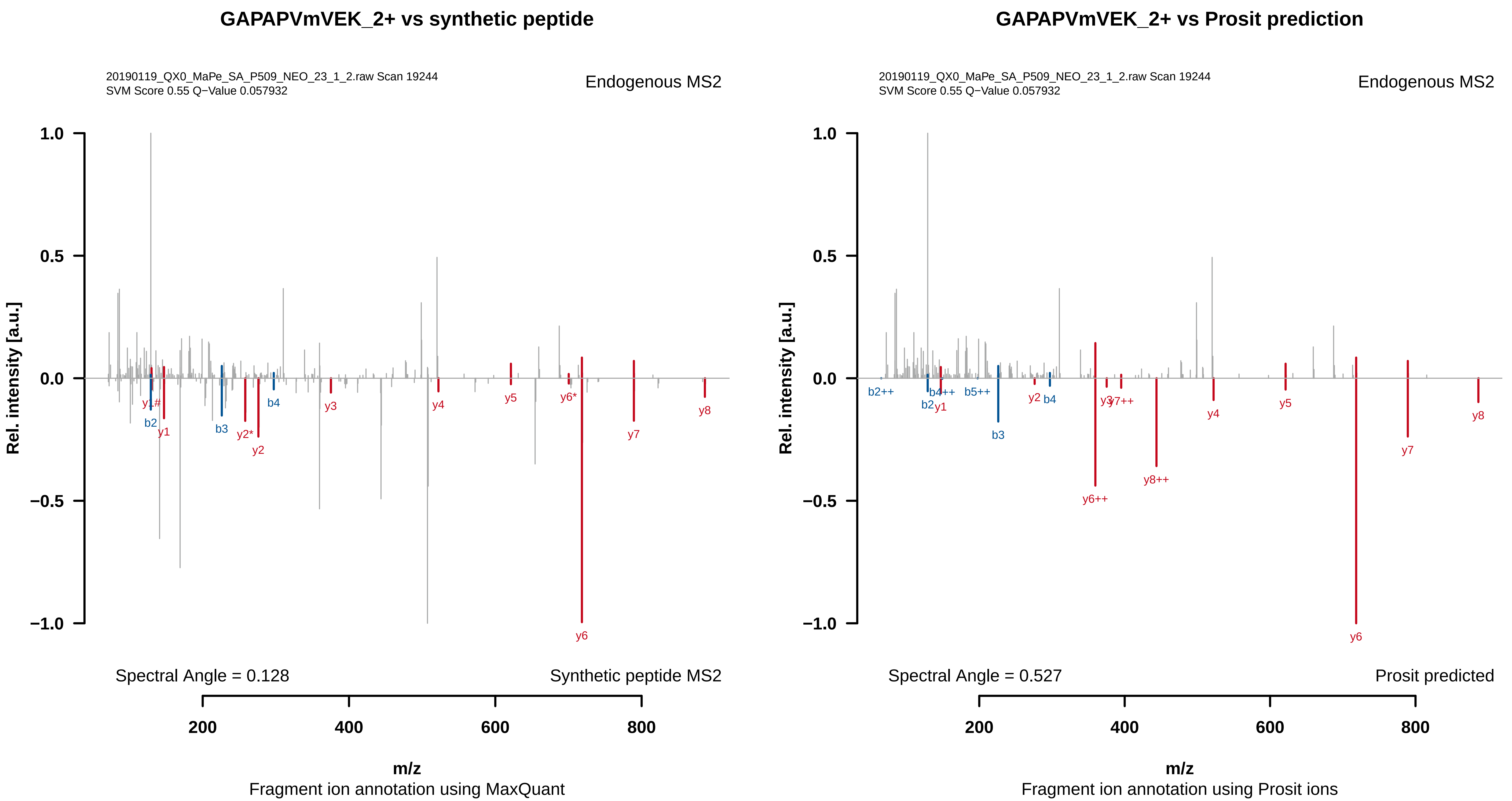
GAPAPVmVEK_2+ vs synthetic peptide
20190119_QX0_MaPe_SA_P509_NEO_23_1_2.raw Scan 19244
SVM Score 0.55 Q−Value 0.057932
Endogenous MS2
Rel. intensity [a.u.]
1.0
0.5
0.0
−0.5
−1.0
y1#
b2
y1
b3
y2*
y2
b4
y3
y4
y5
y6*
y6
y7
y8
Spectral Angle = 0.128
Synthetic peptide MS2
200
400
600
800
m/z
Fragment ion annotation using MaxQuant
GAPAPVmVEK_2+ vs Prosit prediction
20190119_QX0_MaPe_SA_P509_NEO_23_1_2.raw Scan 19244
SVM Score 0.55 Q−Value 0.057932
Endogenous MS2
Rel. intensity [a.u.]
b2++
b2
b4++
y1
b5++
b3
y2
b4
y6++
y3
y7++
y8++
y4
y5
y6
y7
y8
Spectral Angle = 0.527
Prosit predicted
m/z
Fragment ion annotation using Prosit ions

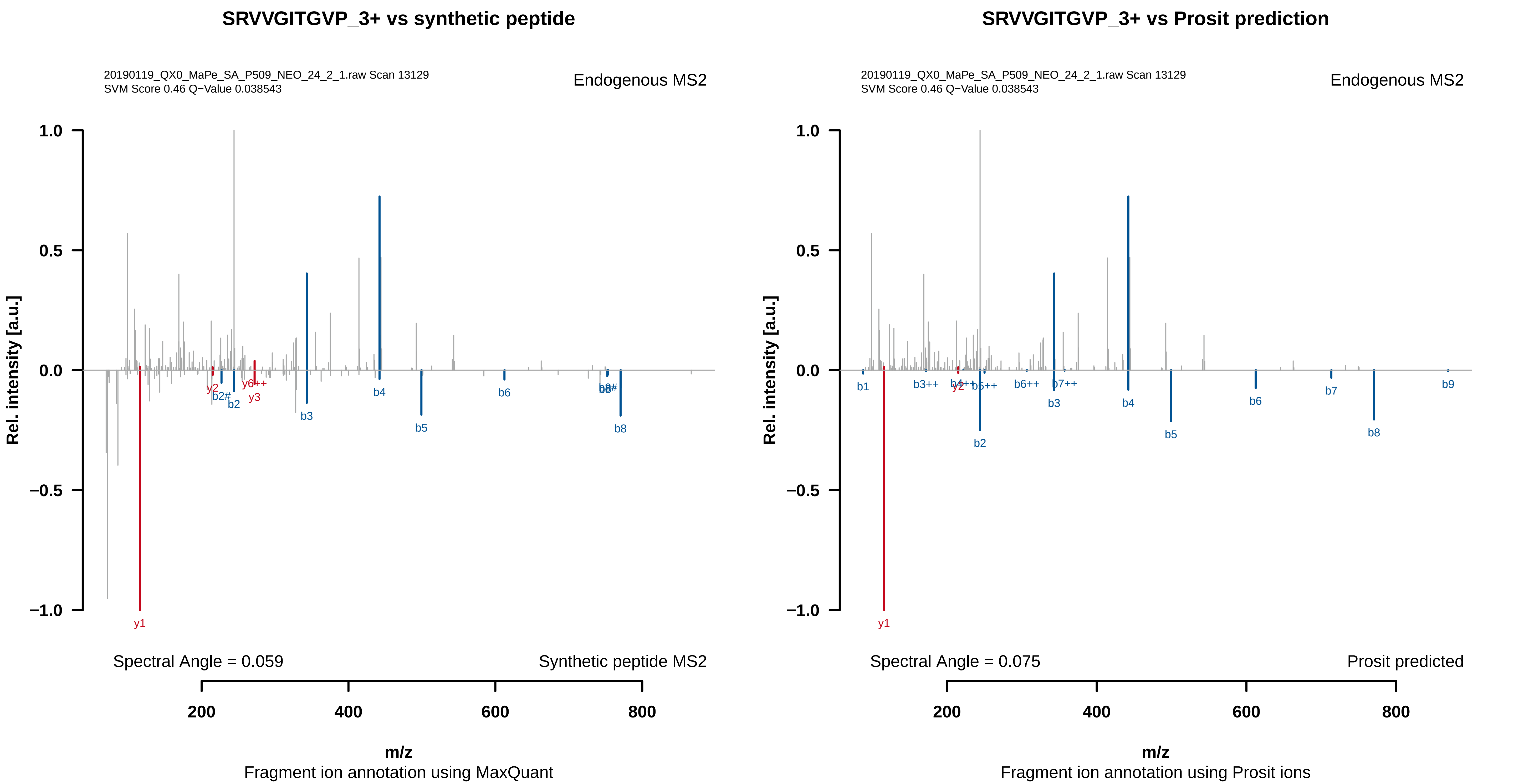
SRVVGITGVP_3+ vs synthetic peptide
20190119_QX0_MaPe_SA_P509_NEO_24_2_1.raw Scan 13129
SVM Score 0.46 Q−Value 0.038543
Endogenous MS2
Rel. intensity [a.u.]
Spectral Angle = 0.059
Synthetic peptide MS2
m/z
Fragment ion annotation using MaxQuant
SRVVGITGVP_3+ vs Prosit prediction
20190119_QX0_MaPe_SA_P509_NEO_24_2_1.raw Scan 13129
SVM Score 0.46 Q−Value 0.038543
Endogenous MS2
Rel. intensity [a.u.]
Spectral Angle = 0.075
Prosit predicted
m/z
Fragment ion annotation using Prosit ions

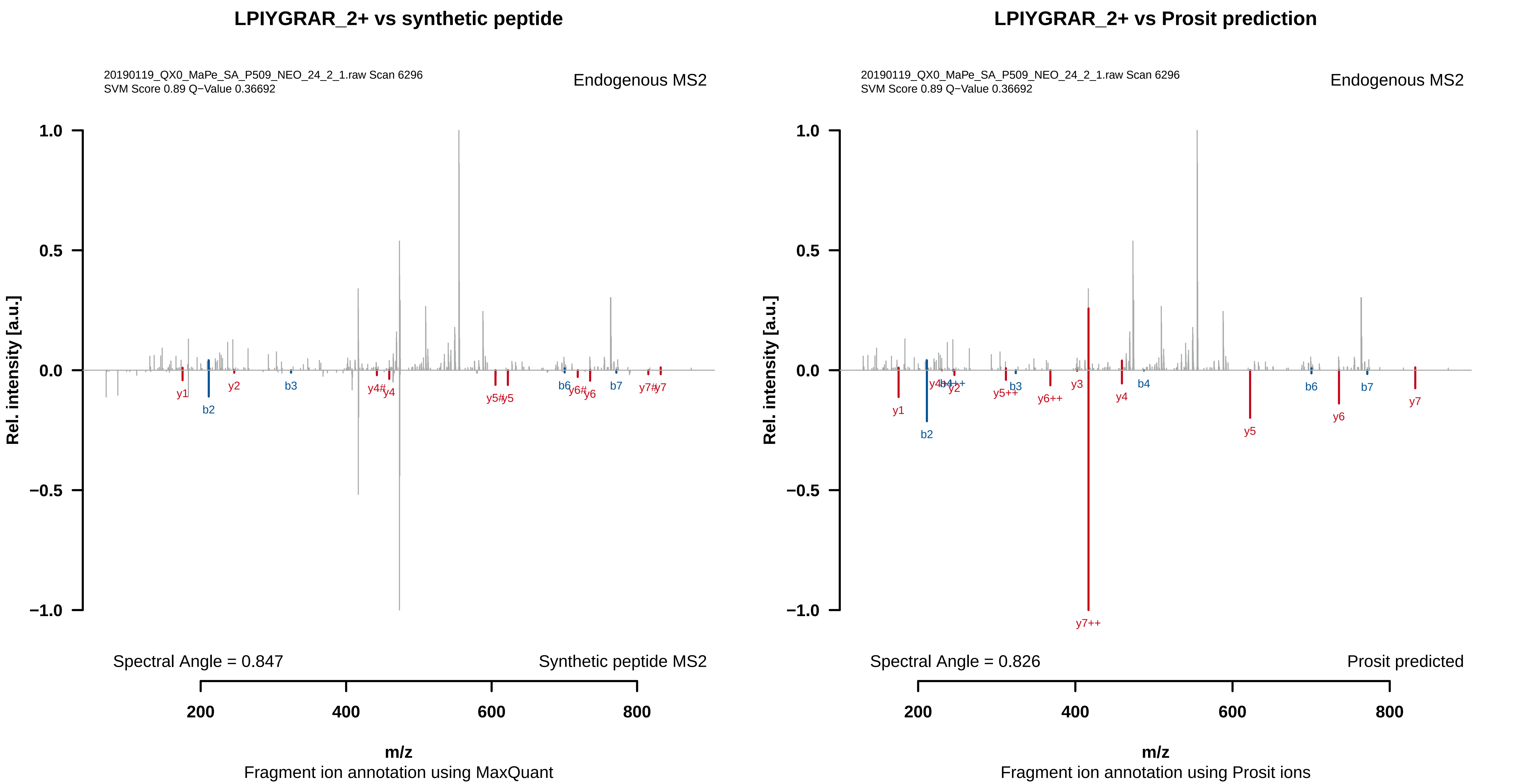
LPIYGRAR_2+ vs synthetic peptide
20190119_QX0_MaPe_SA_P509_NEO_24_2_1.raw Scan 6296
SVM Score 0.89 Q−Value 0.36692
Endogenous MS2
Rel. intensity [a.u.]
1.0
0.5
0.0
−0.5
−1.0
y1
b2
y2
b3
y4#
y4
y5#
y5
b6
y6#
y6
b7
y7#
y7
Spectral Angle = 0.847
Synthetic peptide MS2
200
400
600
800
m/z
Fragment ion annotation using MaxQuant
LPIYGRAR_2+ vs Prosit prediction
20190119_QX0_MaPe_SA_P509_NEO_24_2_1.raw Scan 6296
SVM Score 0.89 Q−Value 0.36692
Endogenous MS2
Rel. intensity [a.u.]
1.0
0.5
0.0
−0.5
−1.0
y1
b2
y4++
b4++
y2
y5++
b3
y6++
y3
y7++
y4
b4
y5
b6
y6
b7
y7
Spectral Angle = 0.826
Prosit predicted
200
400
600
800
m/z
Fragment ion annotation using Prosit ions

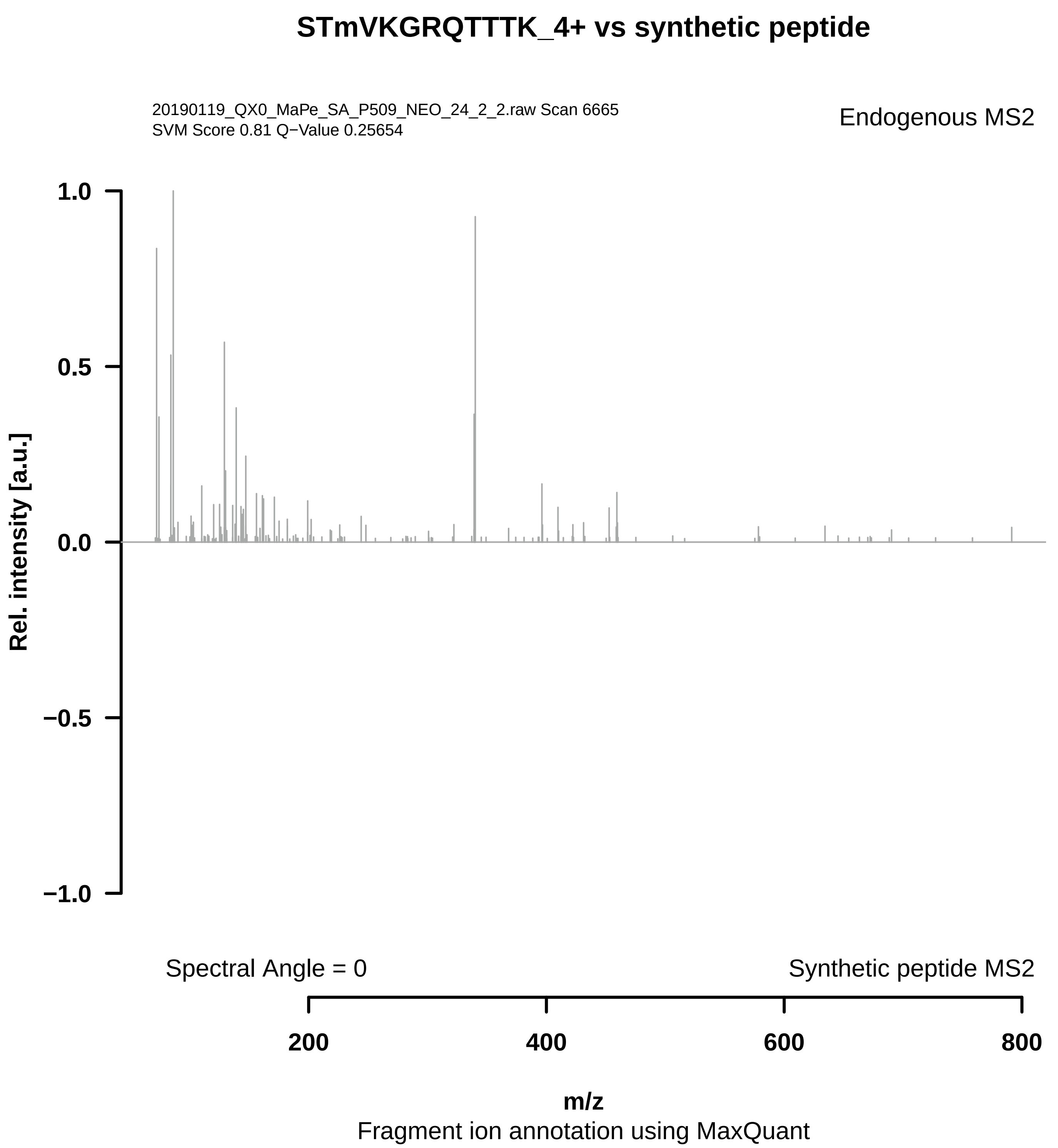

STmVKGRQTTTK_4+ vs synthetic peptide
20190119_QX0_MaPe_SA_P509_NEO_24_2_2.raw Scan 6665
SVM Score 0.81 Q−Value 0.25654
Endogenous MS2
Rel. intensity [a.u.]
Spectral Angle = 0
Synthetic peptide MS2
m/z
Fragment ion annotation using MaxQuant

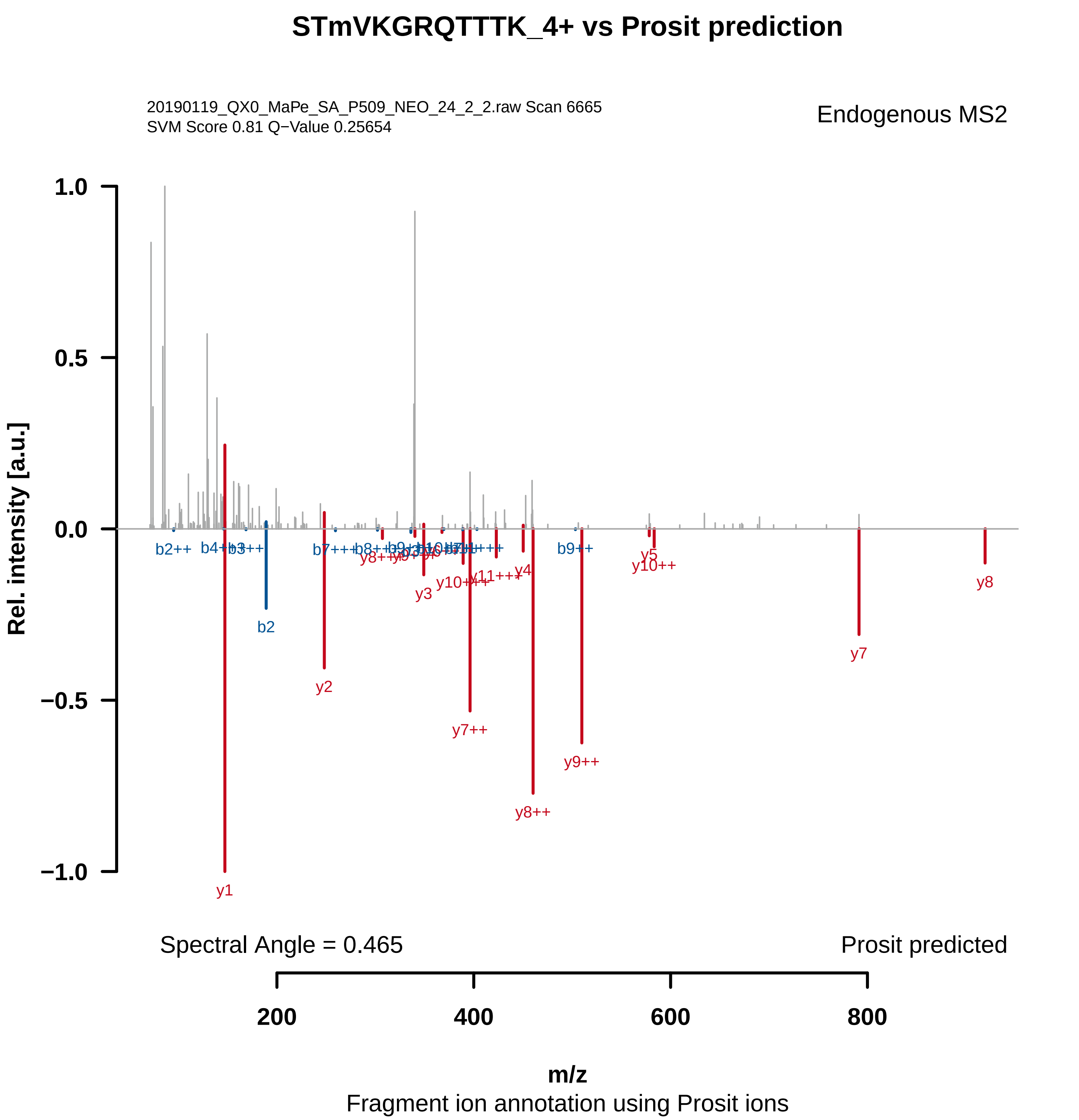

STmVKGRQTTTK_4+ vs Prosit prediction
20190119_QX0_MaPe_SA_P509_NEO_24_2_2.raw Scan 6665
SVM Score 0.81 Q−Value 0.25654
Endogenous MS2
Rel. intensity [a.u.]
Spectral Angle = 0.465
Prosit predicted
m/z
Fragment ion annotation using Prosit ions

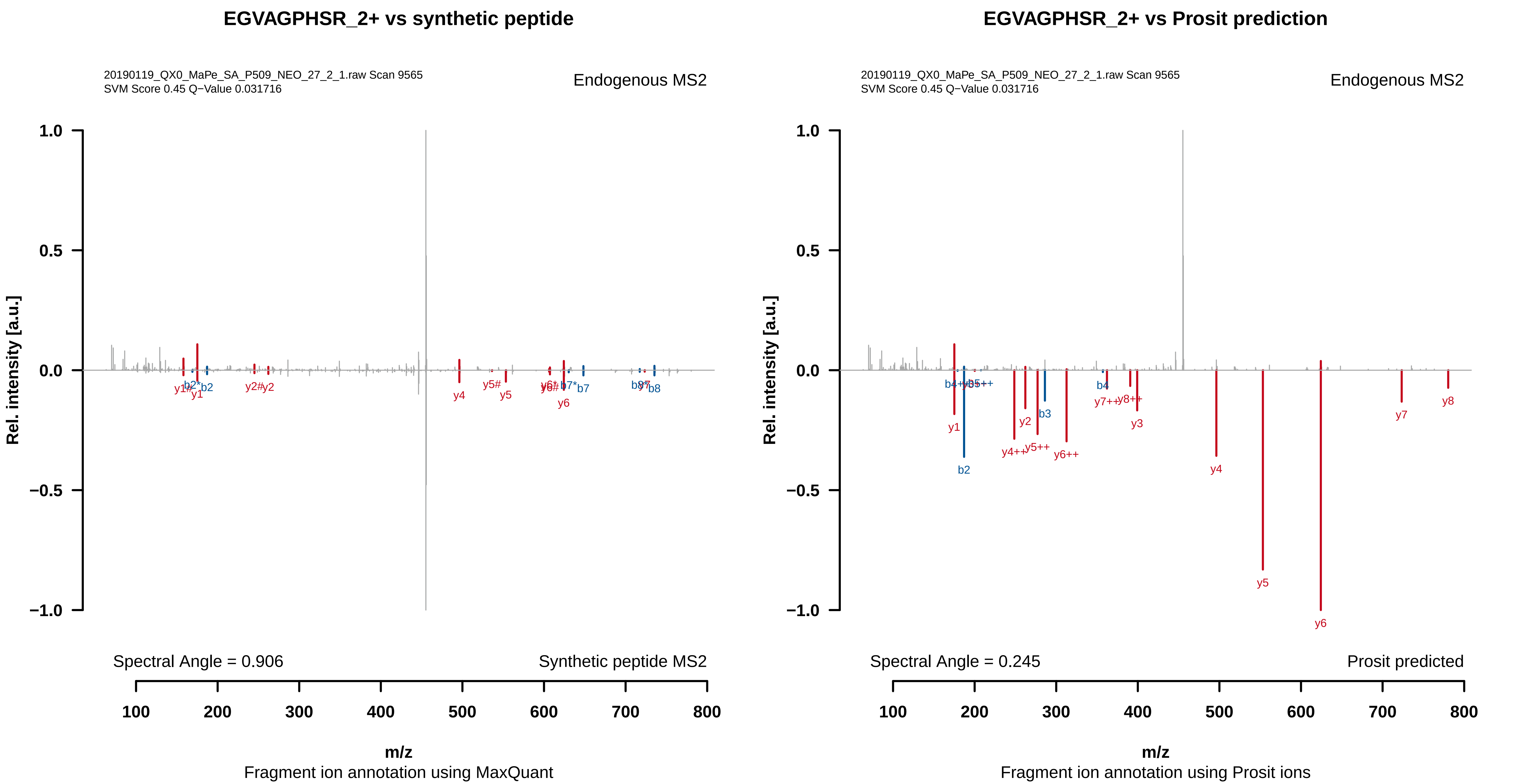

EGVAGPHSR_2+ vs synthetic peptide
20190119_QX0_MaPe_SA_P509_NEO_27_2_1.raw Scan 9565
SVM Score 0.45 Q−Value 0.031716
Endogenous MS2
Rel. intensity [a.u.]
1.0
0.5
0.0
−0.5
−1.0
y1
b2
y2#
y2
y4
y5#
y5
y6
b7
b8
Spectral Angle = 0.906
Synthetic peptide MS2
100
200
300
400
500
600
700
800
m/z
Fragment ion annotation using MaxQuant
EGVAGPHSR_2+ vs Prosit prediction
20190119_QX0_MaPe_SA_P509_NEO_27_2_1.raw Scan 9565
SVM Score 0.45 Q−Value 0.031716
Endogenous MS2
Rel. intensity [a.u.]
1.0
0.5
0.0
−0.5
−1.0
y1
b2
y4++
y2
y5++
b3
y6++
b4
y7++
y8++
y3
y4
y5
y6
y7
y8
Spectral Angle = 0.245
Prosit predicted
100
200
300
400
500
600
700
800
m/z
Fragment ion annotation using Prosit ions

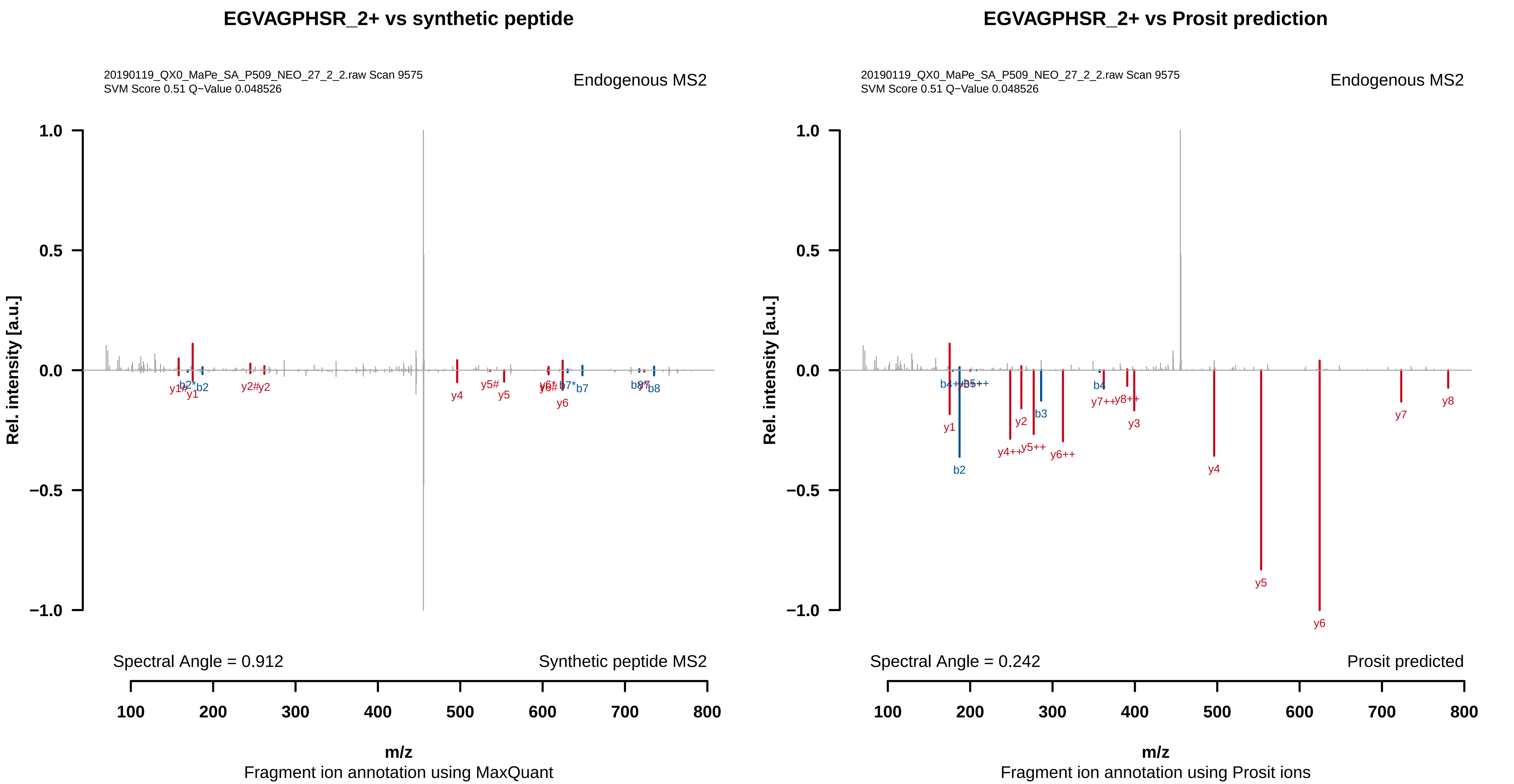
EGVAGPHSR_2+ vs synthetic peptide
20190119_QX0_MaPe_SA_P509_NEO_27_2_2.raw Scan 9575
SVM Score 0.51 Q−Value 0.048526
Endogenous MS2
Rel. intensity [a.u.]
Spectral Angle = 0.912
Synthetic peptide MS2
m/z
Fragment ion annotation using MaxQuant
EGVAGPHSR_2+ vs Prosit prediction
20190119_QX0_MaPe_SA_P509_NEO_27_2_2.raw Scan 9575
SVM Score 0.51 Q−Value 0.048526
Endogenous MS2
Rel. intensity [a.u.]
Spectral Angle = 0.242
Prosit predicted
m/z
Fragment ion annotation using Prosit ions

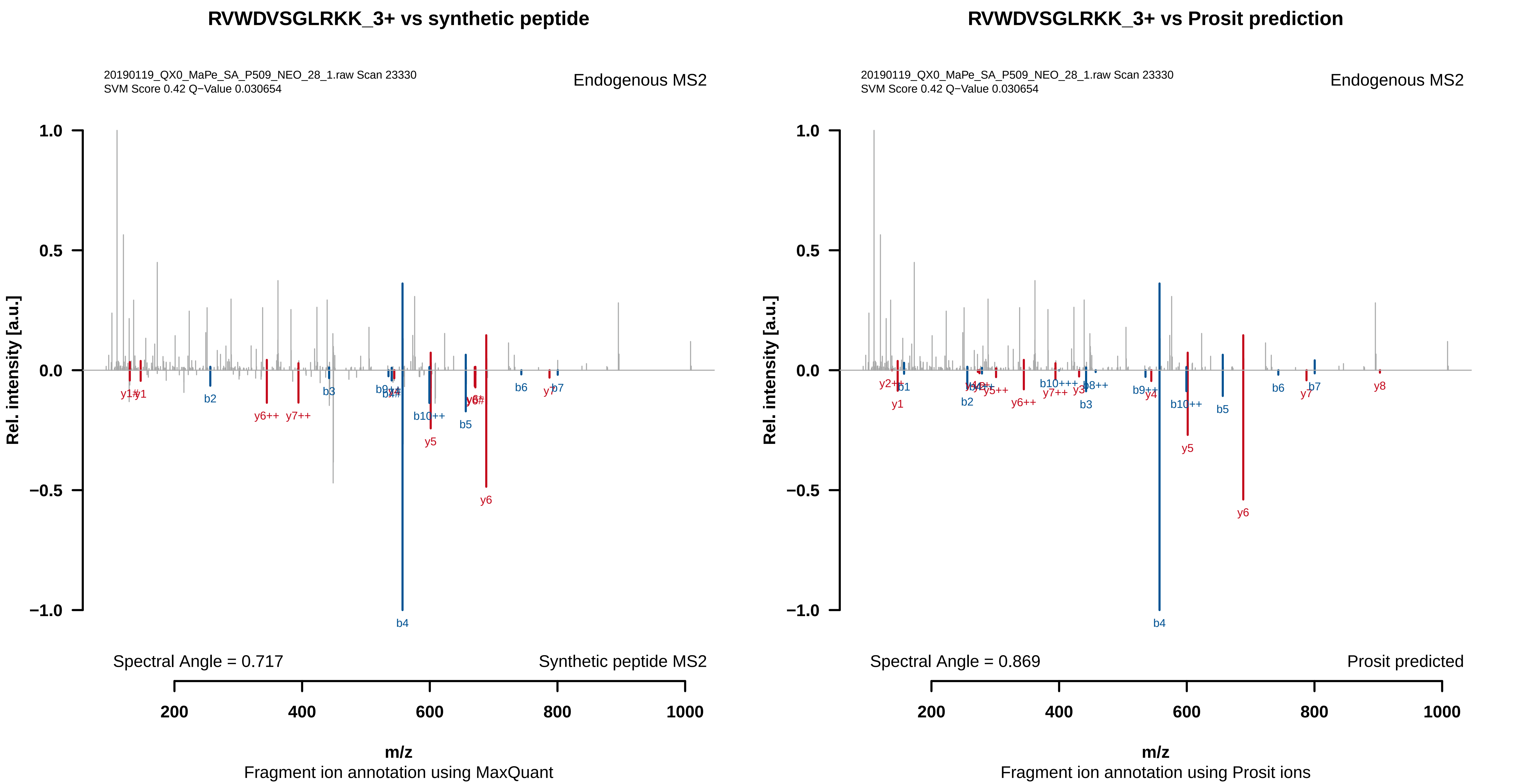
RVWDVSGLRKK_3+ vs synthetic peptide
20190119_QX0_MaPe_SA_P509_NEO_28_1.raw Scan 23330
SVM Score 0.42 Q−Value 0.030654
Endogenous MS2
Rel. intensity [a.u.]
1.0
0.5
0.0
−0.5
−1.0
y1
b2
y6++
y7++
b3
b10++
y5
b5
y6
b6
b7
b4
Spectral Angle = 0.717
Synthetic peptide MS2
200
400
600
800
1000
m/z
Fragment ion annotation using MaxQuant
RVWDVSGLRKK_3+ vs Prosit prediction
20190119_QX0_MaPe_SA_P509_NEO_28_1.raw Scan 23330
SVM Score 0.42 Q−Value 0.030654
Endogenous MS2
Rel. intensity [a.u.]
1.0
0.5
0.0
−0.5
−1.0
y2++
b1
y1
b2
y5++
y6++
b10+++
y7++
y3
b8++
b3
b9++
y4
b10++
b5
y5
y6
b6
y7
b7
y8
b4
Spectral Angle = 0.869
Prosit predicted
200
400
600
800
1000
m/z
Fragment ion annotation using Prosit ions

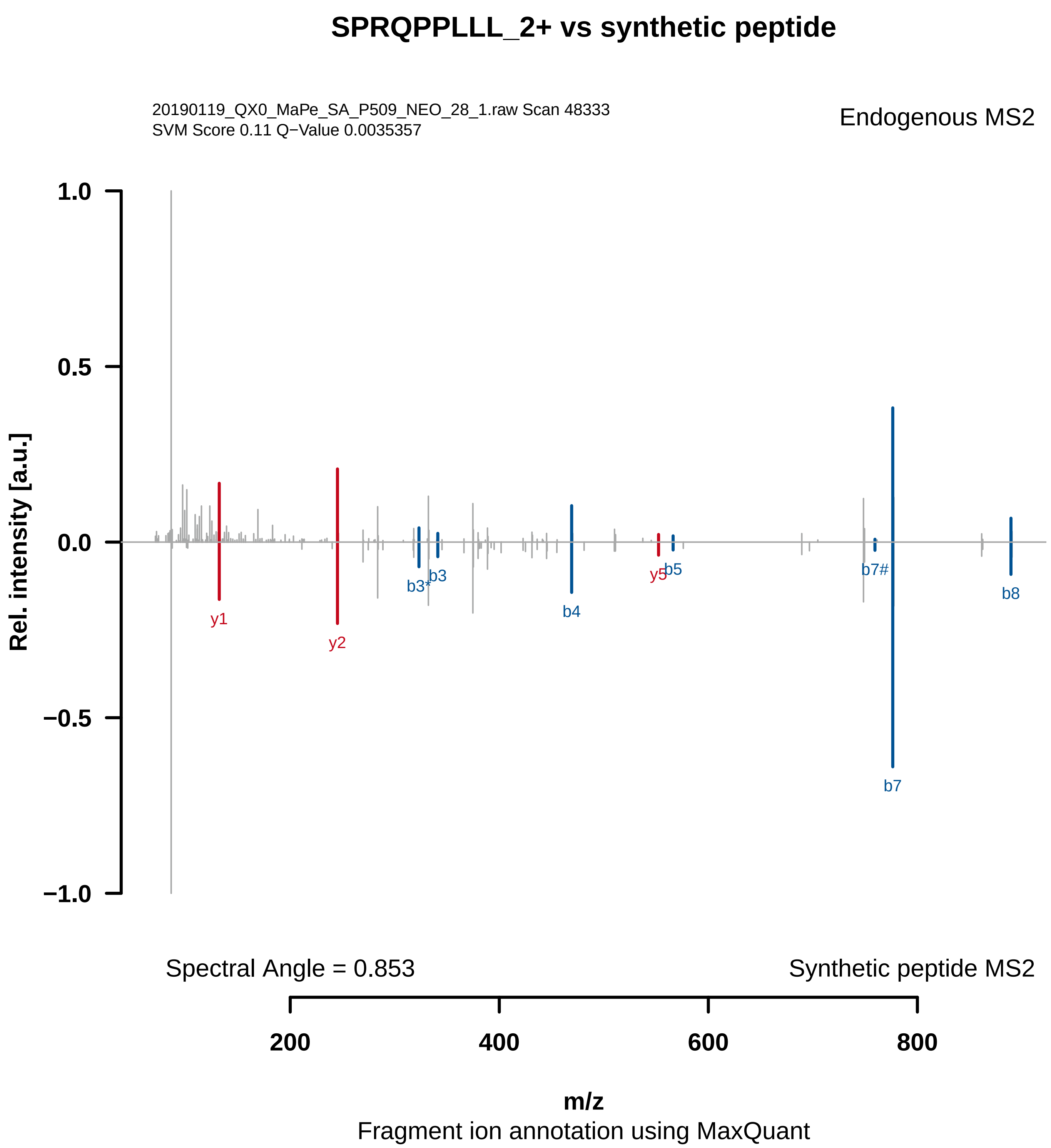
SPRQPPLLL_2+ vs synthetic peptide
20190119_QX0_MaPe_SA_P509_NEO_28_1.raw Scan 48333
SVM Score 0.11 Q−Value 0.0035357
Endogenous MS2
Rel. intensity [a.u.]
1.0
0.5
0.0
−0.5
−1.0
y1
y2
b3*
b3
b4
y5
b5
b7#
b7
b8
Spectral Angle = 0.853
Synthetic peptide MS2
200
400
600
800
m/z
Fragment ion annotation using MaxQuant

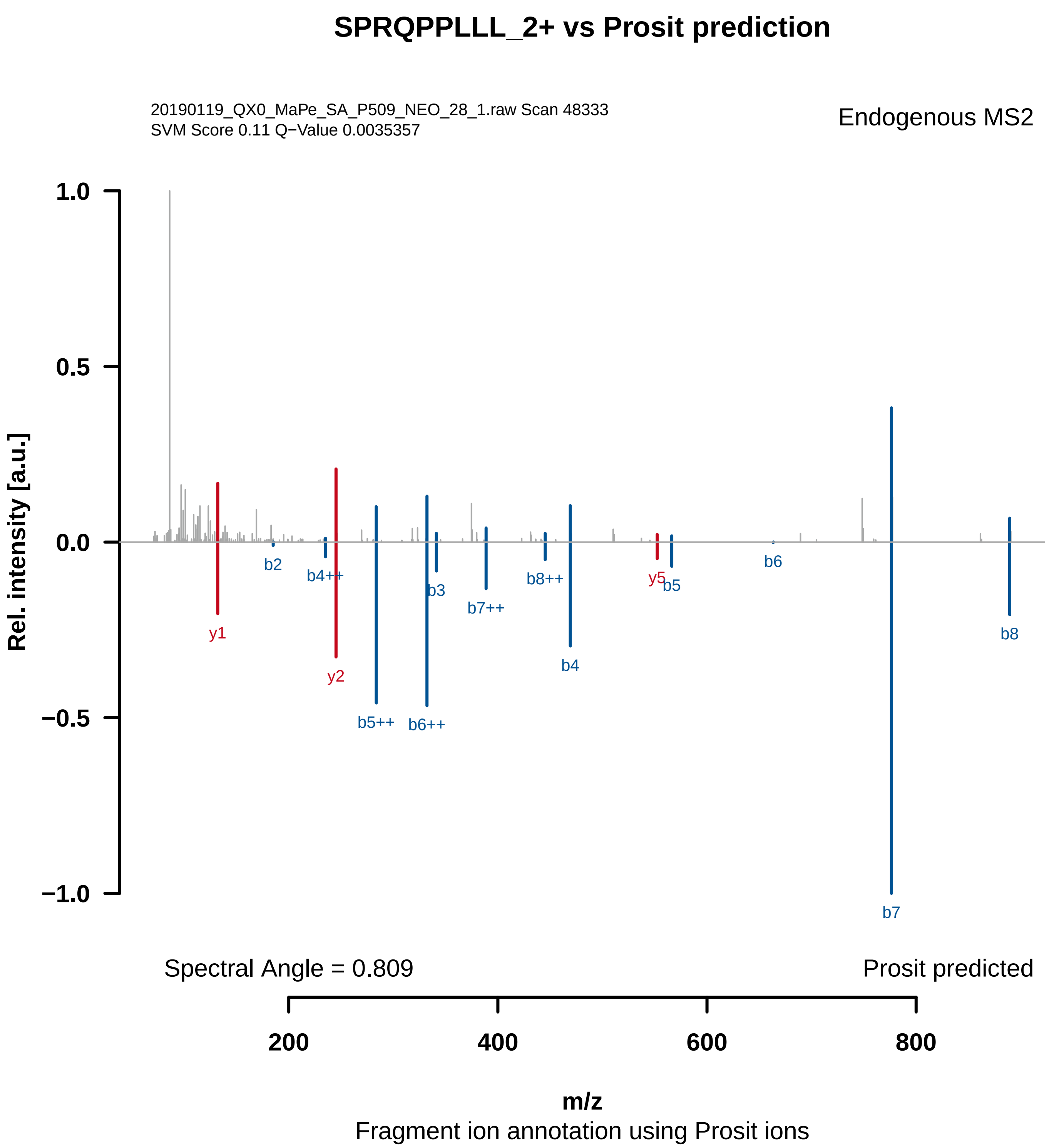
SPRQPPLLL_2+ vs Prosit prediction
20190119_QX0_MaPe_SA_P509_NEO_28_1.raw Scan 48333
SVM Score 0.11 Q−Value 0.0035357
Endogenous MS2
Rel. intensity [a.u.]
1.0
0.5
0.0
−0.5
−1.0
y1
b2
b4++
y2
b5++
b6++
b3
b7++
b8++
b4
y5
b5
b6
b7
b8
Spectral Angle = 0.809
Prosit predicted
200
400
600
800
m/z
Fragment ion annotation using Prosit ions

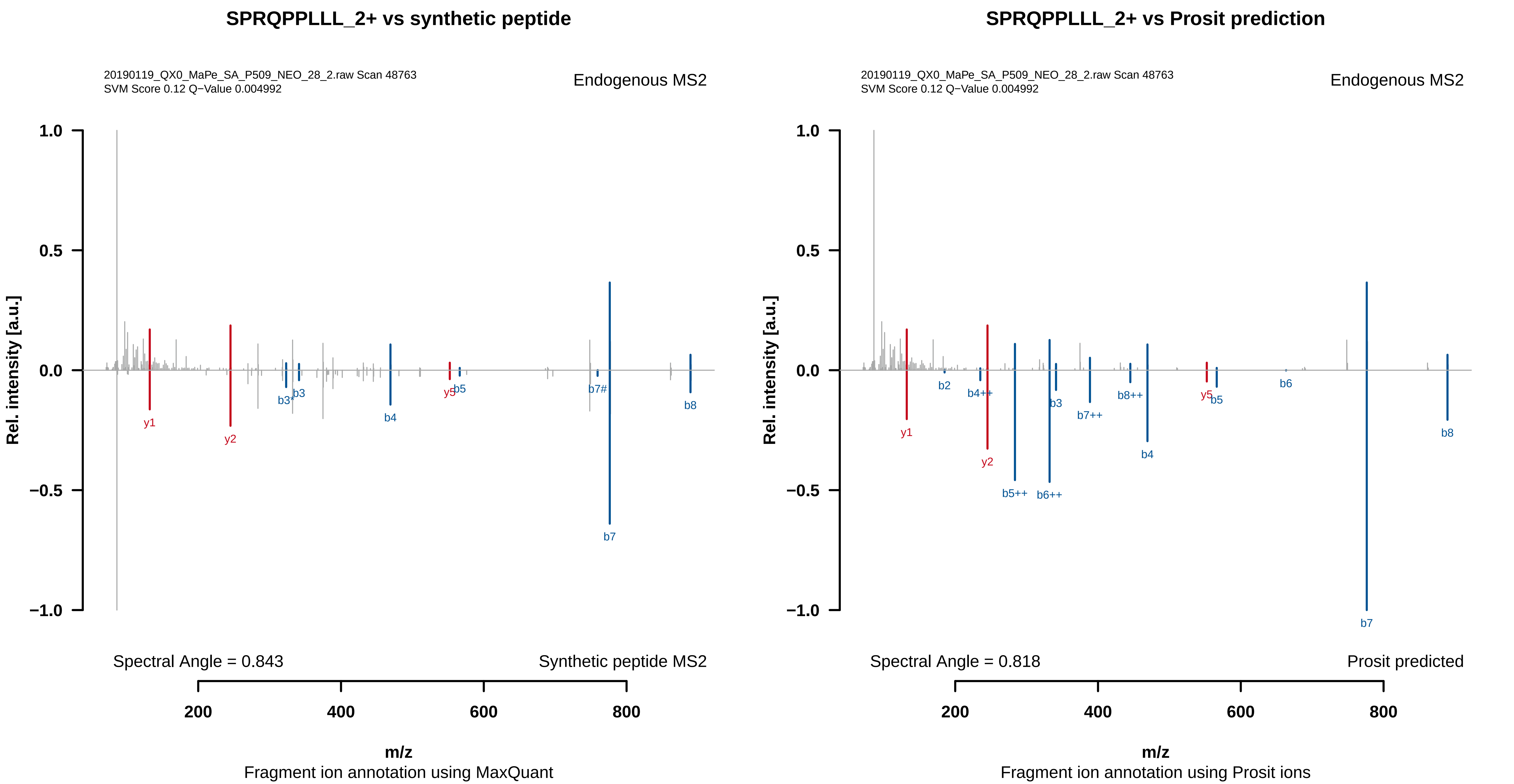
SPRQPPLLL_2+ vs synthetic peptide
20190119_QX0_MaPe_SA_P509_NEO_28_2.raw Scan 48763
SVM Score 0.12 Q−Value 0.004992
Endogenous MS2
Rel. intensity [a.u.]
y1
y2
b3*
b3
b4
y5
b5
b7#
b7
b8
Spectral Angle = 0.843
Synthetic peptide MS2
m/z
Fragment ion annotation using MaxQuant
SPRQPPLLL_2+ vs Prosit prediction
20190119_QX0_MaPe_SA_P509_NEO_28_2.raw Scan 48763
SVM Score 0.12 Q−Value 0.004992
Endogenous MS2
Rel. intensity [a.u.]
y1
b2
b4++
y2
b5++
b6++
b3
b7++
b8++
b4
y5
b5
b6
b7
b8
Spectral Angle = 0.818
Prosit predicted
m/z
Fragment ion annotation using Prosit ions

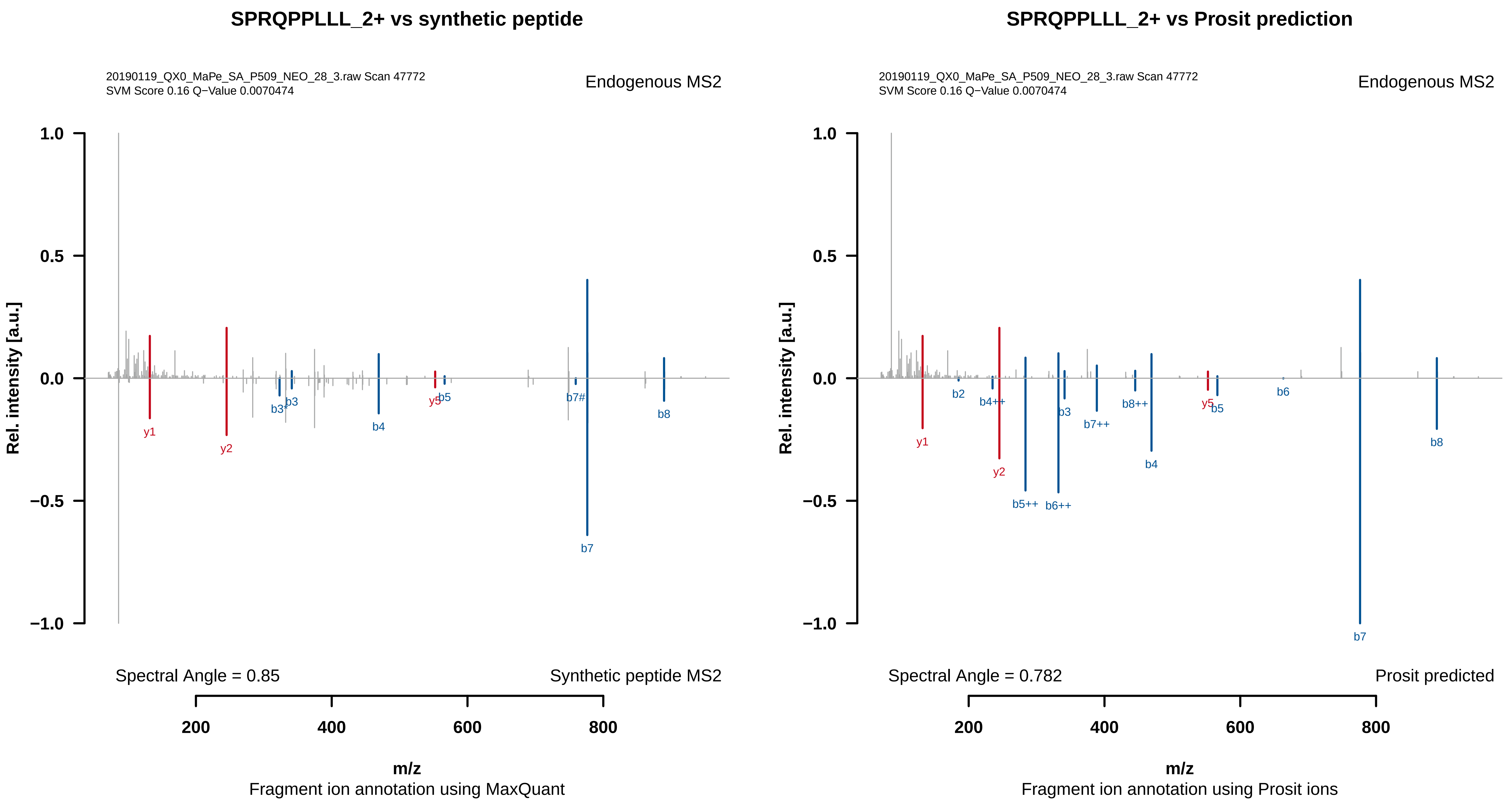
SPRQPPLLL_2+ vs synthetic peptide
20190119_QX0_MaPe_SA_P509_NEO_28_3.raw Scan 47772
SVM Score 0.16 Q−Value 0.0070474
Endogenous MS2
Rel. intensity [a.u.]
1.0
0.5
0.0
−0.5
−1.0
y1
y2
b3*
b3
b4
y5
b5
b7#
b7
b8
Spectral Angle = 0.85
Synthetic peptide MS2
200
400
600
800
m/z
Fragment ion annotation using MaxQuant
SPRQPPLLL_2+ vs Prosit prediction
20190119_QX0_MaPe_SA_P509_NEO_28_3.raw Scan 47772
SVM Score 0.16 Q−Value 0.0070474
Endogenous MS2
Rel. intensity [a.u.]
1.0
0.5
0.0
−0.5
−1.0
y1
b2
b4++
y2
b5++
b6++
b3
b7++
b8++
b4
y5
b5
b6
b7
b8
Spectral Angle = 0.782
Prosit predicted
200
400
600
800
m/z
Fragment ion annotation using Prosit ions

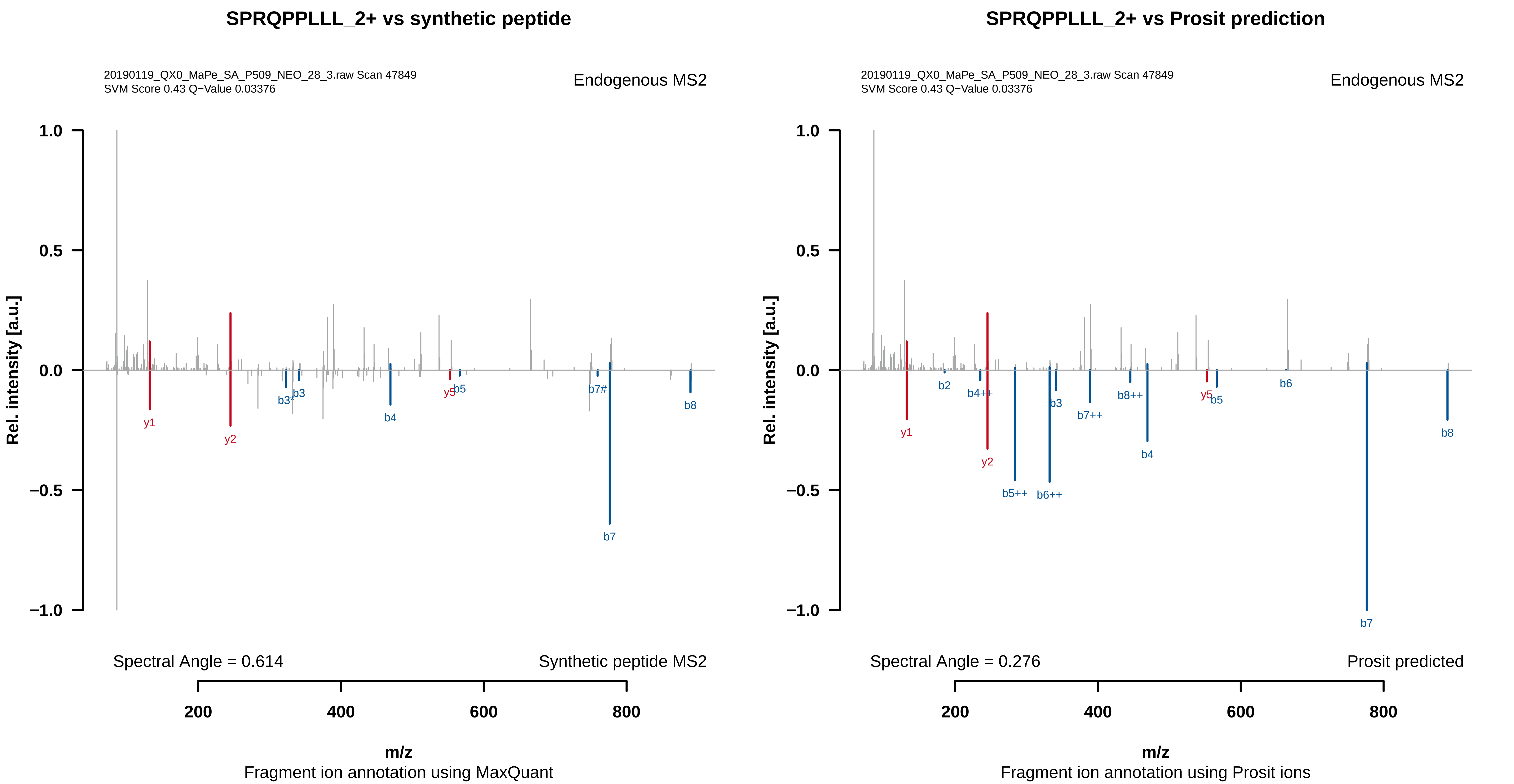
SPRQPPLLL_2+ vs synthetic peptide
20190119_QX0_MaPe_SA_P509_NEO_28_3.raw Scan 47849
SVM Score 0.43 Q−Value 0.03376
Endogenous MS2
Rel. intensity [a.u.]
1.0
0.5
0.0
−0.5
−1.0
y1
y2
b3*
b3
b4
y5
b5
b7#
b7
b8
Spectral Angle = 0.614
Synthetic peptide MS2
200
400
600
800
m/z
Fragment ion annotation using MaxQuant
SPRQPPLLL_2+ vs Prosit prediction
20190119_QX0_MaPe_SA_P509_NEO_28_3.raw Scan 47849
SVM Score 0.43 Q−Value 0.03376
Endogenous MS2
Rel. intensity [a.u.]
1.0
0.5
0.0
−0.5
−1.0
y1
b2
b4++
y2
b5++
b6++
b3
b7++
b8++
b4
y5
b5
b6
b7
b8
Spectral Angle = 0.276
Prosit predicted
200
400
600
800
m/z
Fragment ion annotation using Prosit ions

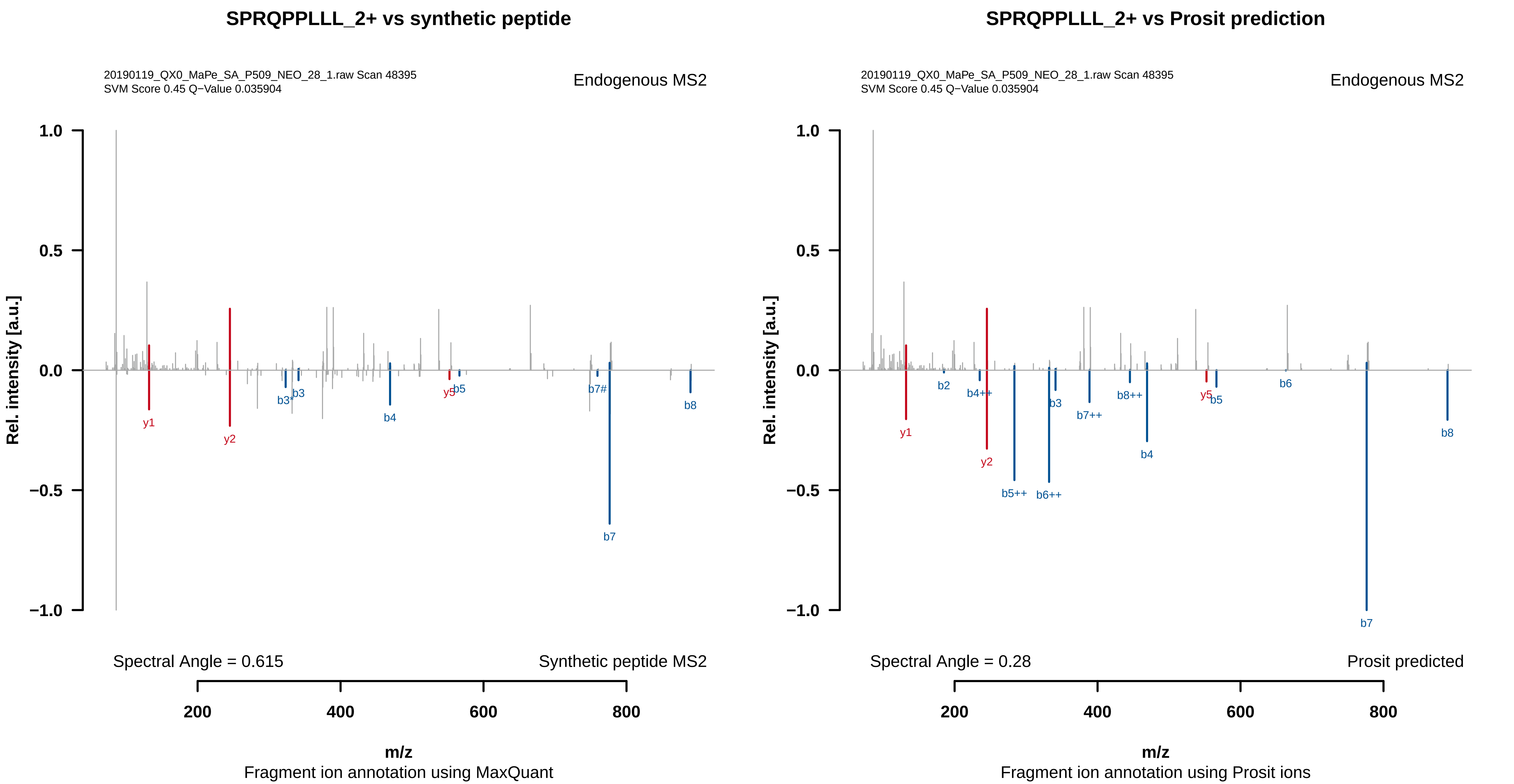
SPRQPPLLL_2+ vs synthetic peptide
20190119_QX0_MaPe_SA_P509_NEO_28_1.raw Scan 48395
SVM Score 0.45 Q−Value 0.035904
Endogenous MS2
Rel. intensity [a.u.]
1.0
0.5
0.0
−0.5
−1.0
y1
y2
b3*
b3
b4
y5
b5
b7#
b7
b8
Spectral Angle = 0.615
Synthetic peptide MS2
200
400
600
800
m/z
Fragment ion annotation using MaxQuant
SPRQPPLLL_2+ vs Prosit prediction
20190119_QX0_MaPe_SA_P509_NEO_28_1.raw Scan 48395
SVM Score 0.45 Q−Value 0.035904
Endogenous MS2
Rel. intensity [a.u.]
1.0
0.5
0.0
−0.5
−1.0
y1
b2
b4++
y2
b5++
b6++
b3
b7++
b8++
b4
y5
b5
b6
b7
b8
Spectral Angle = 0.28
Prosit predicted
200
400
600
800
m/z
Fragment ion annotation using Prosit ions

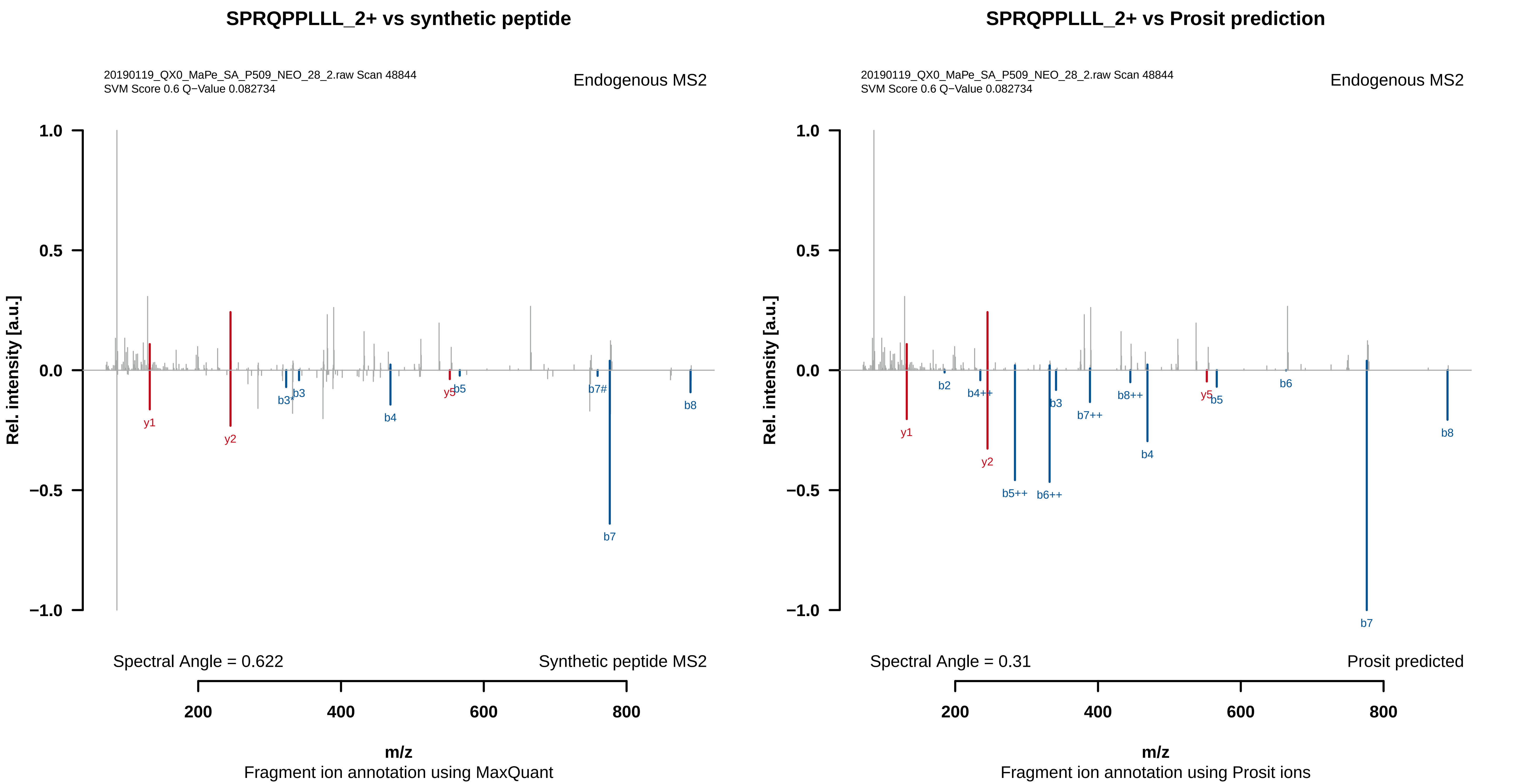
SPRQPPLLL_2+ vs synthetic peptide
20190119_QX0_MaPe_SA_P509_NEO_28_2.raw Scan 48844
SVM Score 0.6 Q−Value 0.082734
Endogenous MS2
1.0
0.5
0.0
−0.5
−1.0
Rel. intensity [a.u.]
y1
y2
b3*
b3
b4
y5
b5
b7#
b7
b8
Spectral Angle = 0.622
Synthetic peptide MS2
200
400
600
800
m/z
Fragment ion annotation using MaxQuant
SPRQPPLLL_2+ vs Prosit prediction
20190119_QX0_MaPe_SA_P509_NEO_28_2.raw Scan 48844
SVM Score 0.6 Q−Value 0.082734
Endogenous MS2
1.0
0.5
0.0
−0.5
−1.0
Rel. intensity [a.u.]
y1
b2
b4++
y2
b5++
b6++
b3
b7++
b8++
b4
y5
b5
b6
b7
b8
Spectral Angle = 0.31
Prosit predicted
200
400
600
800
m/z
Fragment ion annotation using Prosit ions

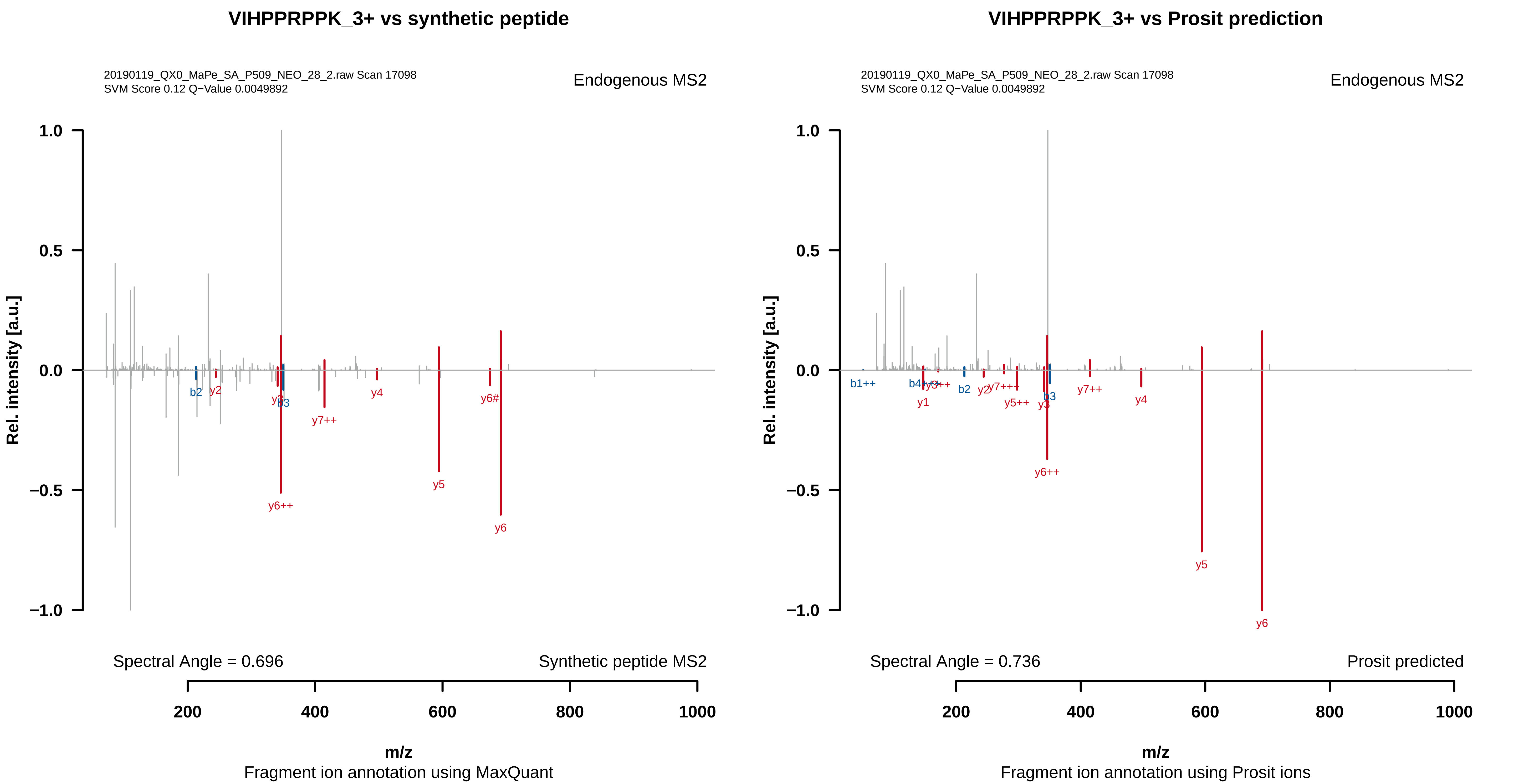
VIHPPRPPK_3+ vs synthetic peptide
20190119_QX0_MaPe_SA_P509_NEO_28_2.raw Scan 17098
SVM Score 0.12 Q−Value 0.0049892
Endogenous MS2
Rel. intensity [a.u.]
1.0
0.5
0.0
−0.5
−1.0
b2
y2
b3
y7++
y4
y6#
y5
y6++
y6
Spectral Angle = 0.696
Synthetic peptide MS2
200
400
600
800
1000
m/z
Fragment ion annotation using MaxQuant
VIHPPRPPK_3+ vs Prosit prediction
20190119_QX0_MaPe_SA_P509_NEO_28_2.raw Scan 17098
SVM Score 0.12 Q−Value 0.0049892
Endogenous MS2
Rel. intensity [a.u.]
1.0
0.5
0.0
−0.5
−1.0
b1++
y1
b2
y2
y7+++
y5++
b3
y7++
y4
y6++
y5
y6
Spectral Angle = 0.736
Prosit predicted
200
400
600
800
1000
m/z
Fragment ion annotation using Prosit ions

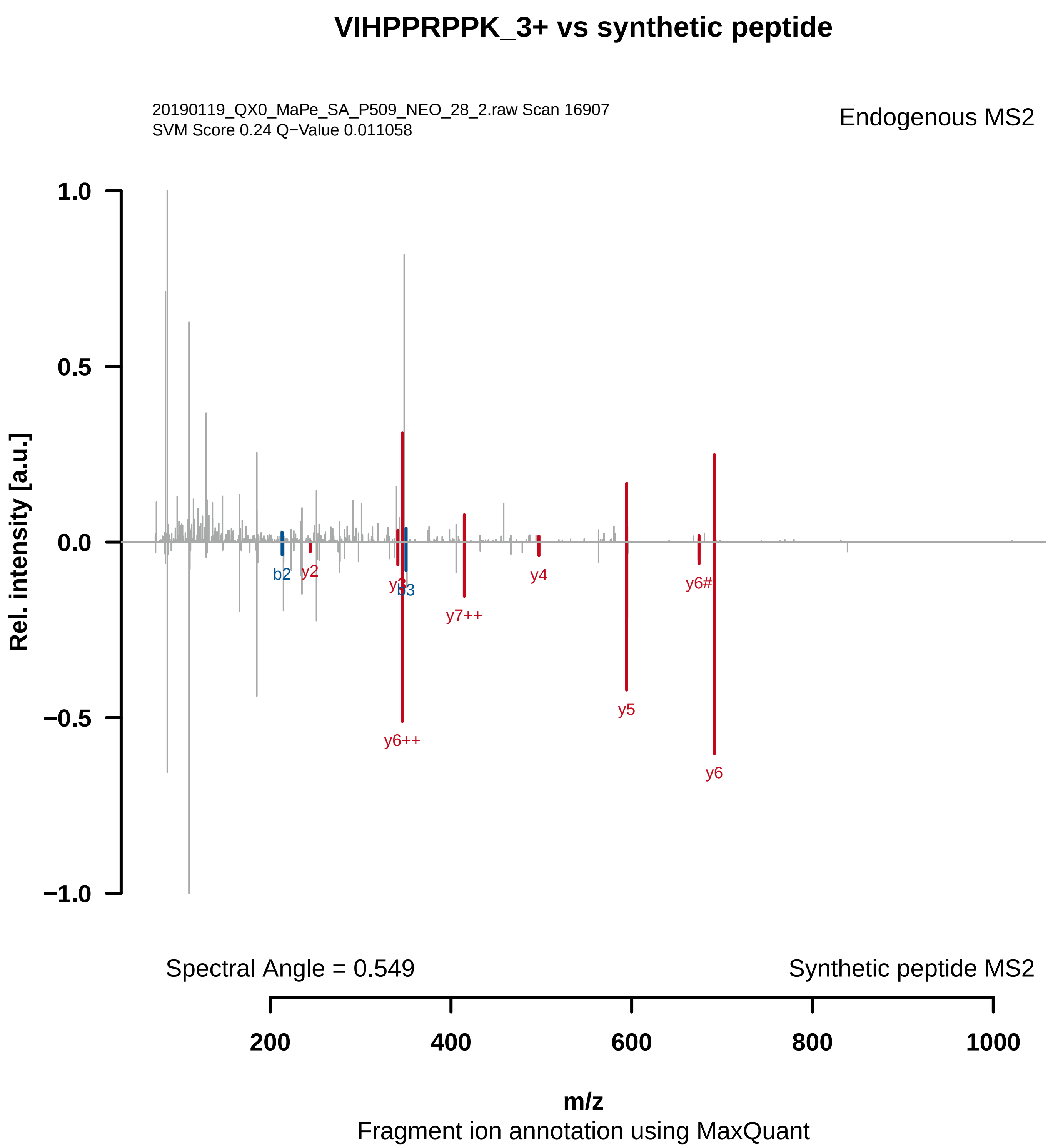

VIHPPRPPK_3+ vs synthetic peptide
20190119_QX0_MaPe_SA_P509_NEO_28_2.raw Scan 16907
SVM Score 0.24 Q−Value 0.011058
Endogenous MS2
Rel. intensity [a.u.]
1.0
0.5
0.0
−0.5
−1.0
b2
y2
y3
b3
y7++
y4
y6#
y6++
y5
y6
Spectral Angle = 0.549
Synthetic peptide MS2
200
400
600
800
1000
m/z
Fragment ion annotation using MaxQuant

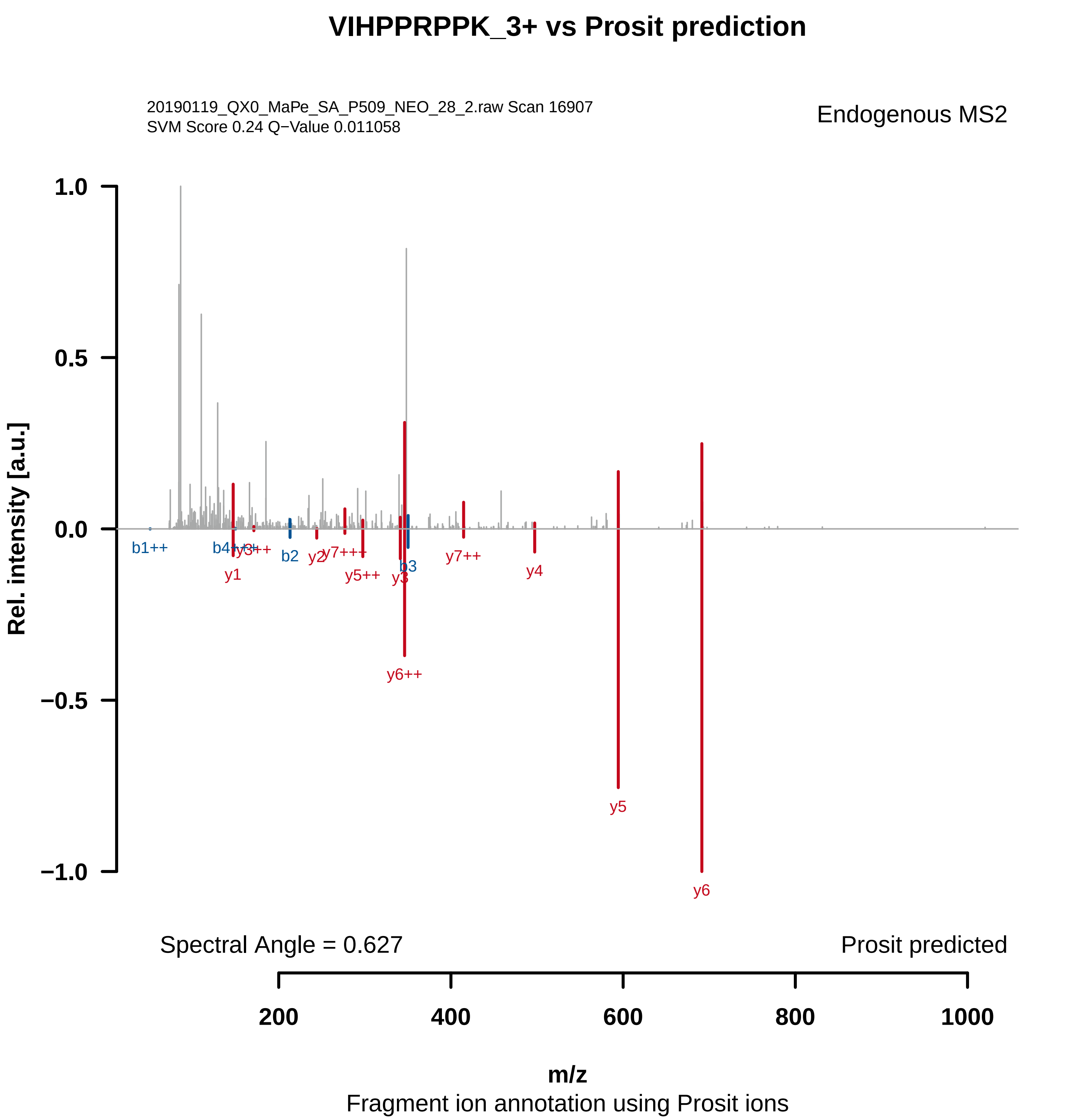

VIHPPRPPK_3+ vs Prosit prediction
20190119_QX0_MaPe_SA_P509_NEO_28_2.raw Scan 16907
SVM Score 0.24 Q−Value 0.011058
Endogenous MS2
Rel. intensity [a.u.]
1.0
0.5
0.0
−0.5
−1.0
b1++
y1
b2
y2
y7+++
y5++
y3
b3
y7++
y4
y6++
y5
y6
Spectral Angle = 0.627
Prosit predicted
200
400
600
800
1000
m/z
Fragment ion annotation using Prosit ions

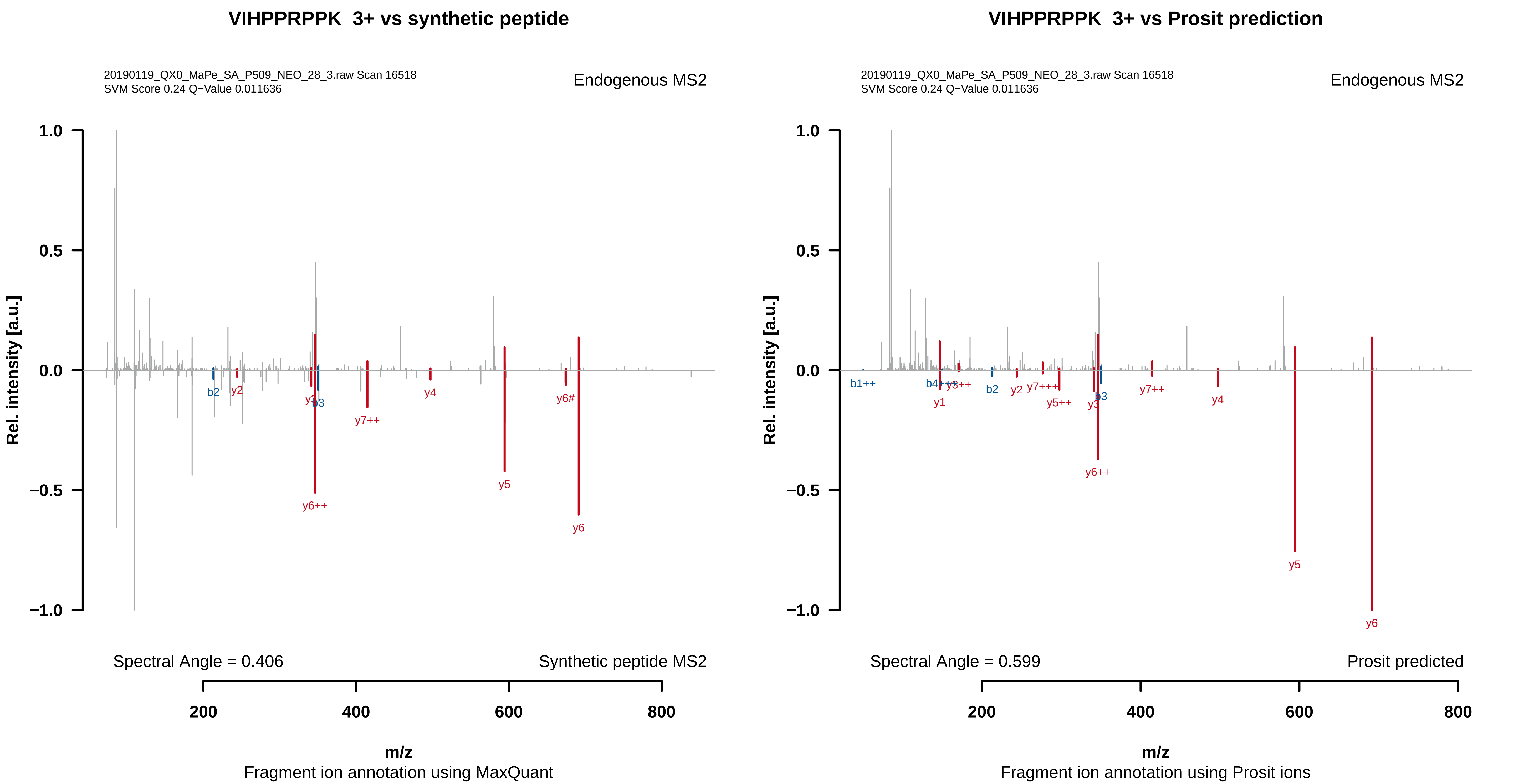
VIHPPRPPK_3+ vs synthetic peptide
20190119_QX0_MaPe_SA_P509_NEO_28_3.raw Scan 16518
SVM Score 0.24 Q−Value 0.011636
Endogenous MS2
1.0
0.5
0.0
−0.5
−1.0
Rel. intensity [a.u.]
b2
y2
y3
b3
y7++
y4
y6#
y5
y6++
y6
Spectral Angle = 0.406
Synthetic peptide MS2
200
400
600
800
m/z
Fragment ion annotation using MaxQuant
VIHPPRPPK_3+ vs Prosit prediction
20190119_QX0_MaPe_SA_P509_NEO_28_3.raw Scan 16518
SVM Score 0.24 Q−Value 0.011636
Endogenous MS2
1.0
0.5
0.0
−0.5
−1.0
Rel. intensity [a.u.]
b1++
b4++
y3++
y1
b2
y2
y7+++
y5++
y3
b3
y7++
y4
y6++
y5
y6
Spectral Angle = 0.599
Prosit predicted
200
400
600
800
m/z
Fragment ion annotation using Prosit ions

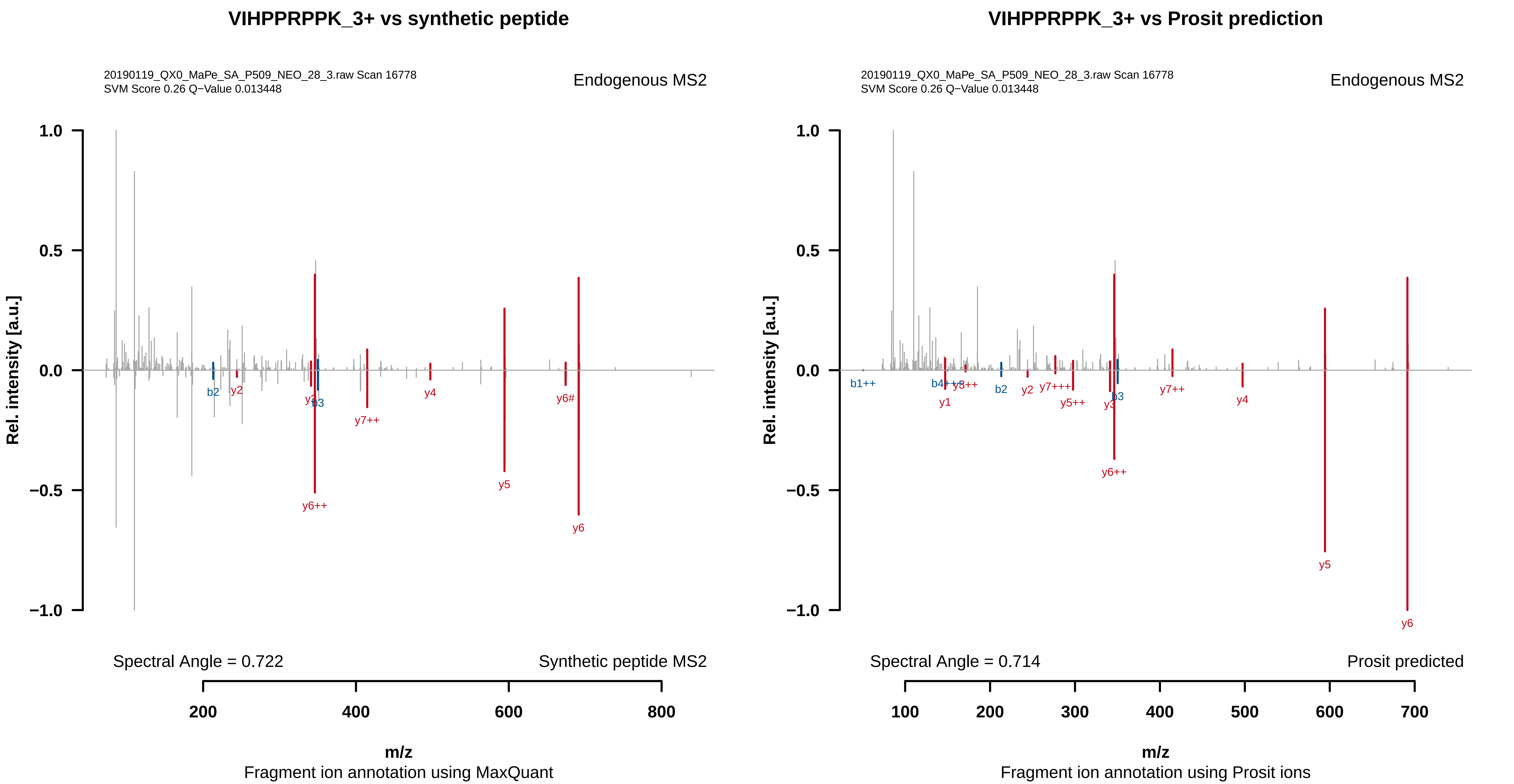
VIHPPRPPK_3+ vs synthetic peptide
20190119_QX0_MaPe_SA_P509_NEO_28_3.raw Scan 16778
SVM Score 0.26 Q−Value 0.013448
Endogenous MS2
Rel. intensity [a.u.]
1.0
0.5
0.0
−0.5
−1.0
b2
y2
y3
b3
y7++
y4
y6#
y6++
y5
y6
Spectral Angle = 0.722
Synthetic peptide MS2
200
400
600
800
m/z
Fragment ion annotation using MaxQuant
VIHPPRPPK_3+ vs Prosit prediction
20190119_QX0_MaPe_SA_P509_NEO_28_3.raw Scan 16778
SVM Score 0.26 Q−Value 0.013448
Endogenous MS2
Rel. intensity [a.u.]
1.0
0.5
0.0
−0.5
−1.0
b1++
b4++
y8++
y1
b2
y2
y7+++
y5++
y3
b3
y7++
y4
y6++
y5
y6
Spectral Angle = 0.714
Prosit predicted
100
200
300
400
500
600
700
m/z
Fragment ion annotation using Prosit ions

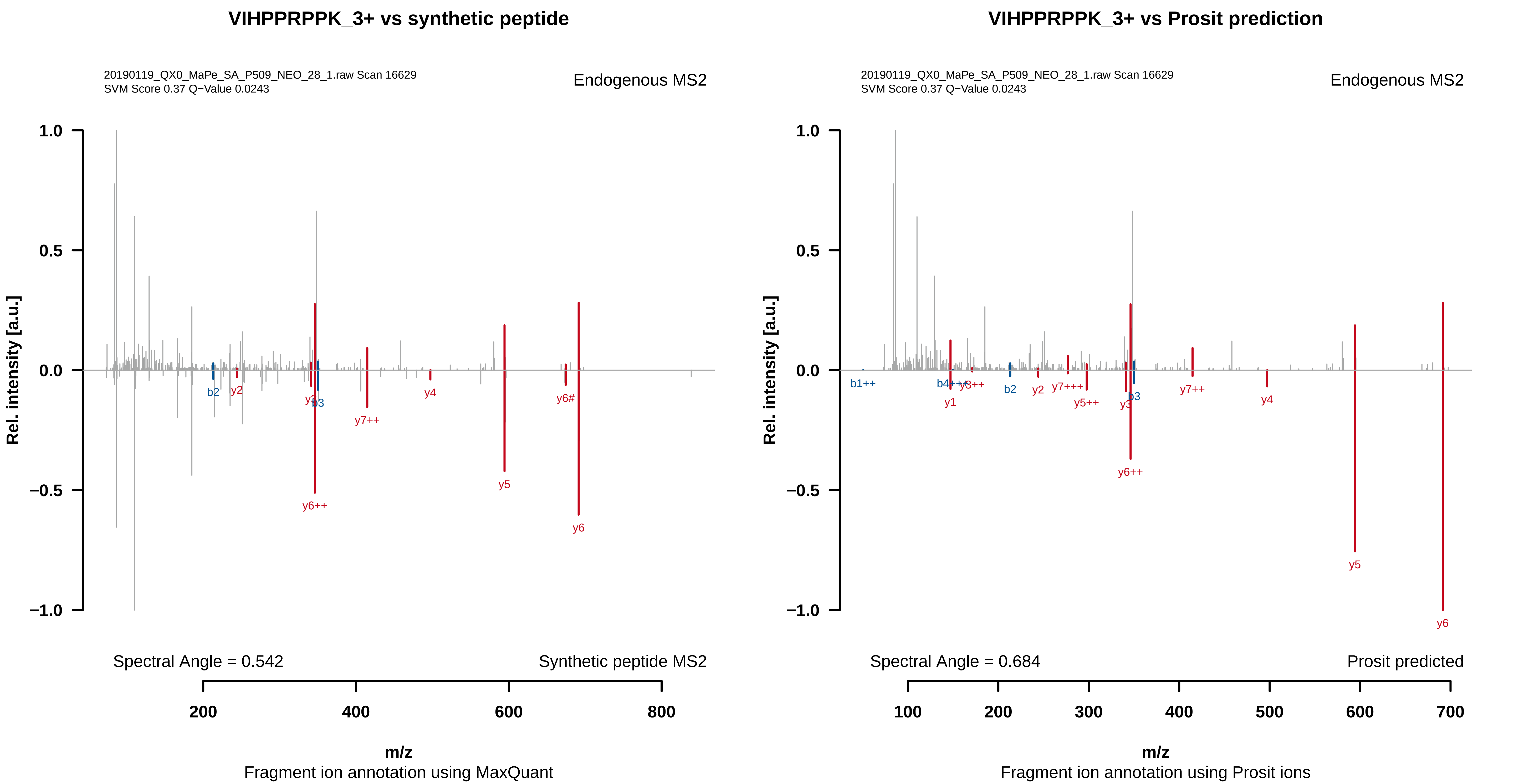

VIHPPRPPK_3+ vs synthetic peptide
20190119_QX0_MaPe_SA_P509_NEO_28_1.raw Scan 16629
SVM Score 0.37 Q−Value 0.0243
Endogenous MS2
Rel. intensity [a.u.]
1.0
0.5
0.0
−0.5
−1.0
b2
y2
y3
b3
y7++
y4
y6#
y6++
y5
y6
Spectral Angle = 0.542
Synthetic peptide MS2
200
400
600
800
m/z
Fragment ion annotation using MaxQuant
VIHPPRPPK_3+ vs Prosit prediction
20190119_QX0_MaPe_SA_P509_NEO_28_1.raw Scan 16629
SVM Score 0.37 Q−Value 0.0243
Endogenous MS2
Rel. intensity [a.u.]
1.0
0.5
0.0
−0.5
−1.0
b1++
b4++
y3++
y1
b2
y2
y7+++
y5++
y3
b3
y7++
y4
y6++
y5
y6
Spectral Angle = 0.684
Prosit predicted
100
200
300
400
500
600
700
m/z
Fragment ion annotation using Prosit ions

VIHPPRPPK_3+ vs synthetic peptide

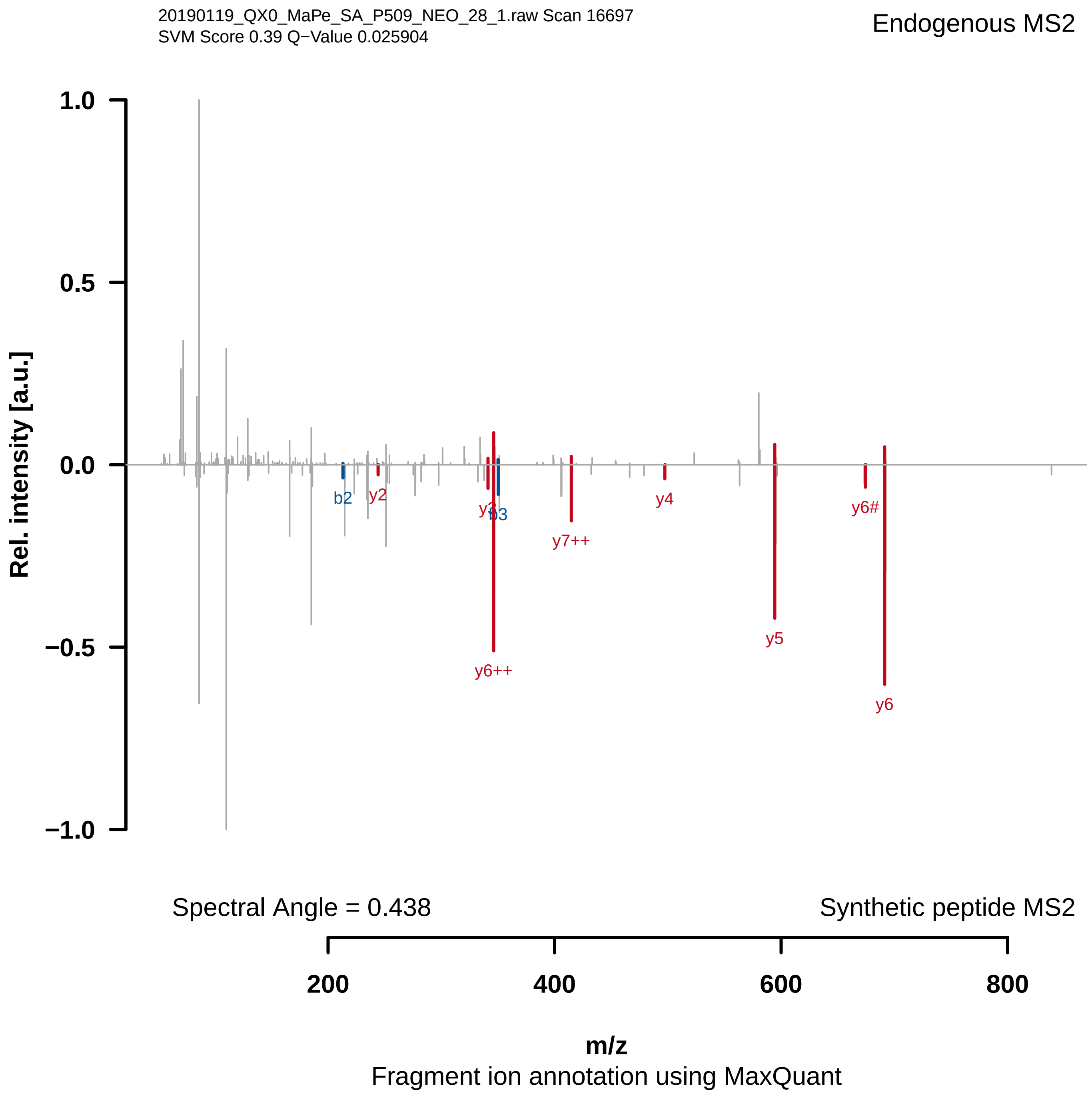

VIHPPRPPK_3+ vs Prosit prediction

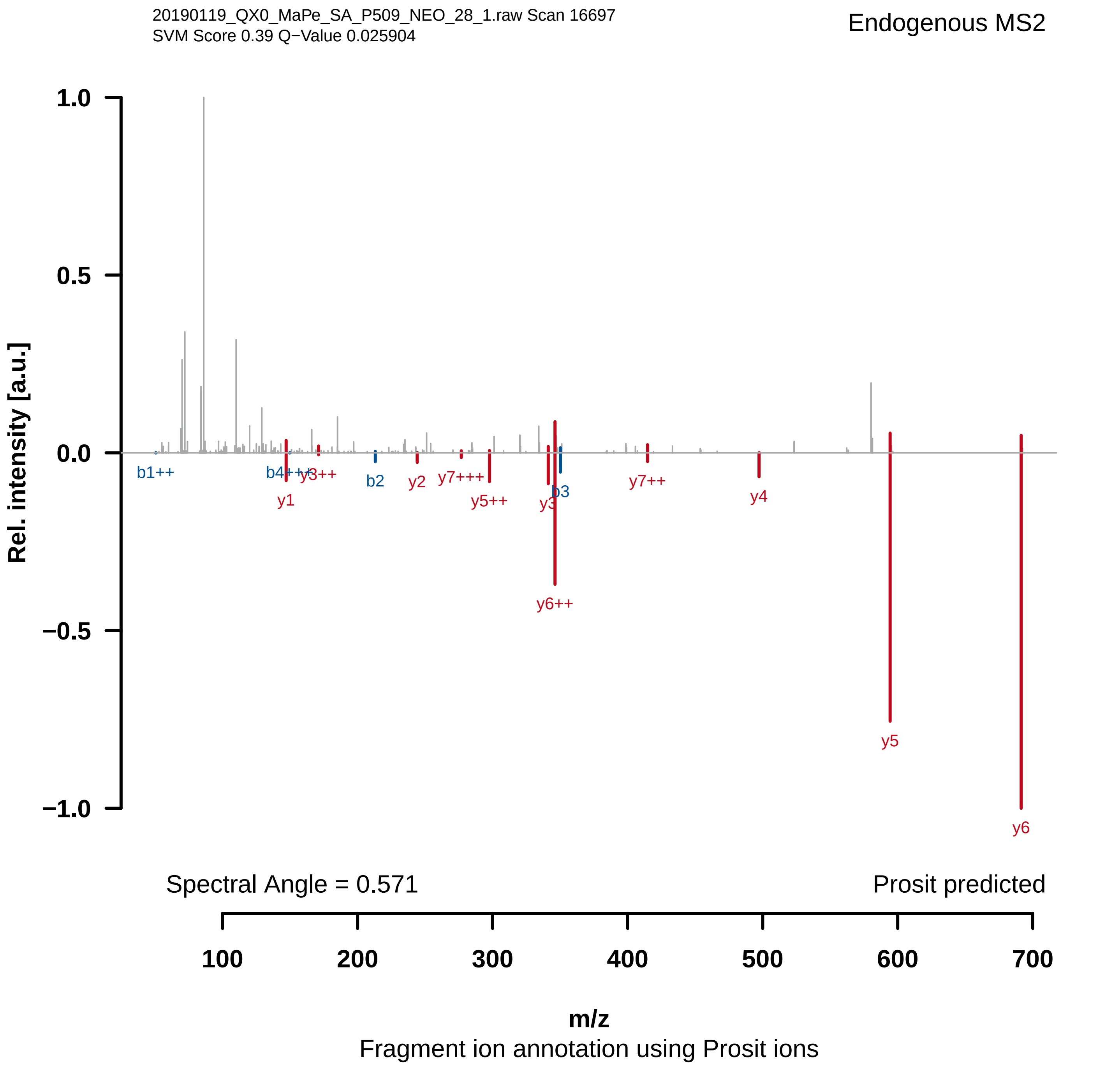

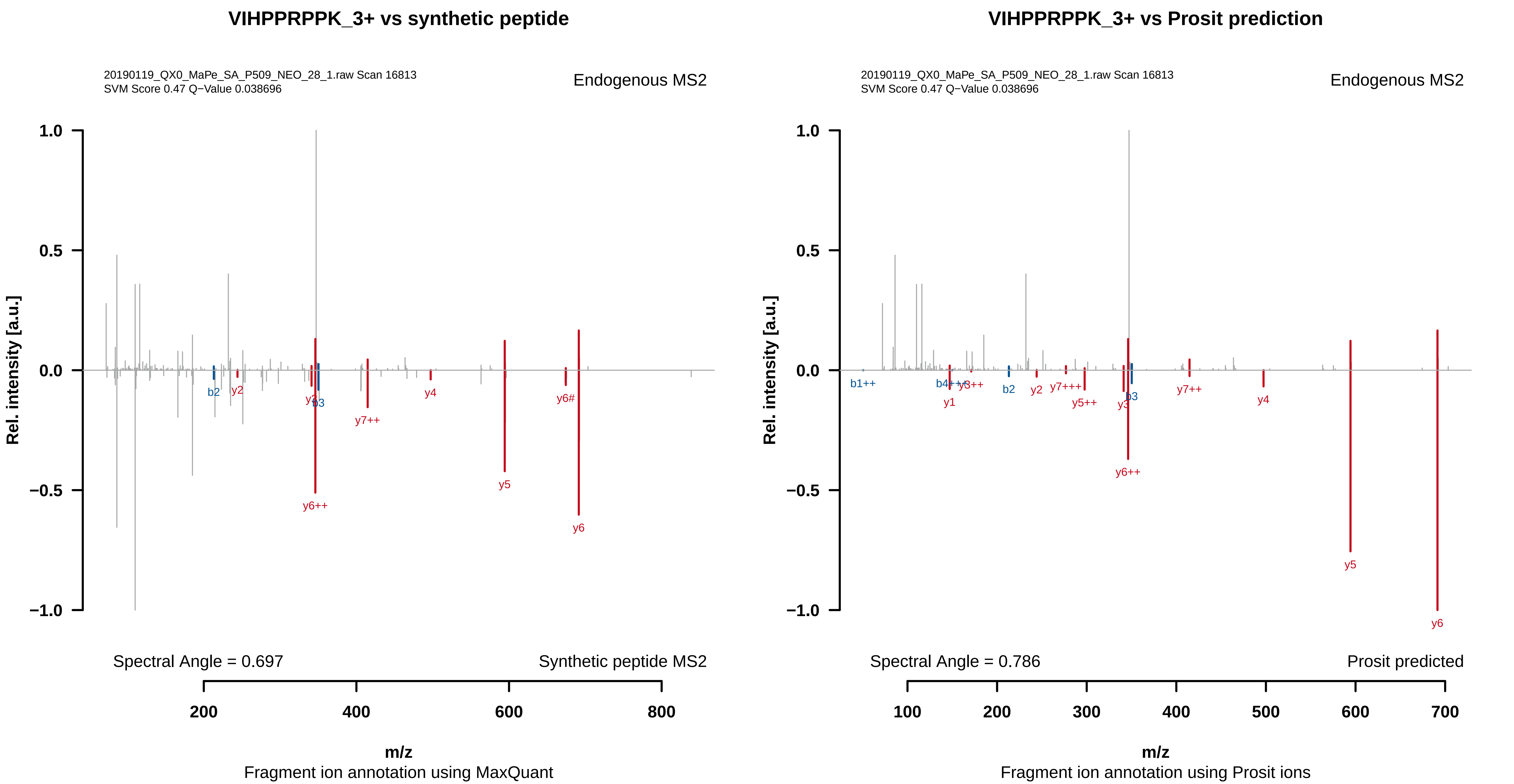

VIHPPRPPK_3+ vs synthetic peptide
20190119_QX0_MaPe_SA_P509_NEO_28_1.raw Scan 16813
SVM Score 0.47 Q−Value 0.038696
Endogenous MS2
Rel. intensity [a.u.]
1.0
0.5
0.0
−0.5
−1.0
b2
y2
y3
b3
y7++
y4
y6#
y5
y6++
y6
Spectral Angle = 0.697
Synthetic peptide MS2
200
400
600
800
m/z
Fragment ion annotation using MaxQuant
VIHPPRPPK_3+ vs Prosit prediction
20190119_QX0_MaPe_SA_P509_NEO_28_1.raw Scan 16813
SVM Score 0.47 Q−Value 0.038696
Endogenous MS2
Rel. intensity [a.u.]
1.0
0.5
0.0
−0.5
−1.0
b1++
b4++
y3++
y1
b2
y2
y7+++
y5++
y3
b3
y7++
y4
y6++
y5
y6
Spectral Angle = 0.786
Prosit predicted
100
200
300
400
500
600
700
m/z
Fragment ion annotation using Prosit ions

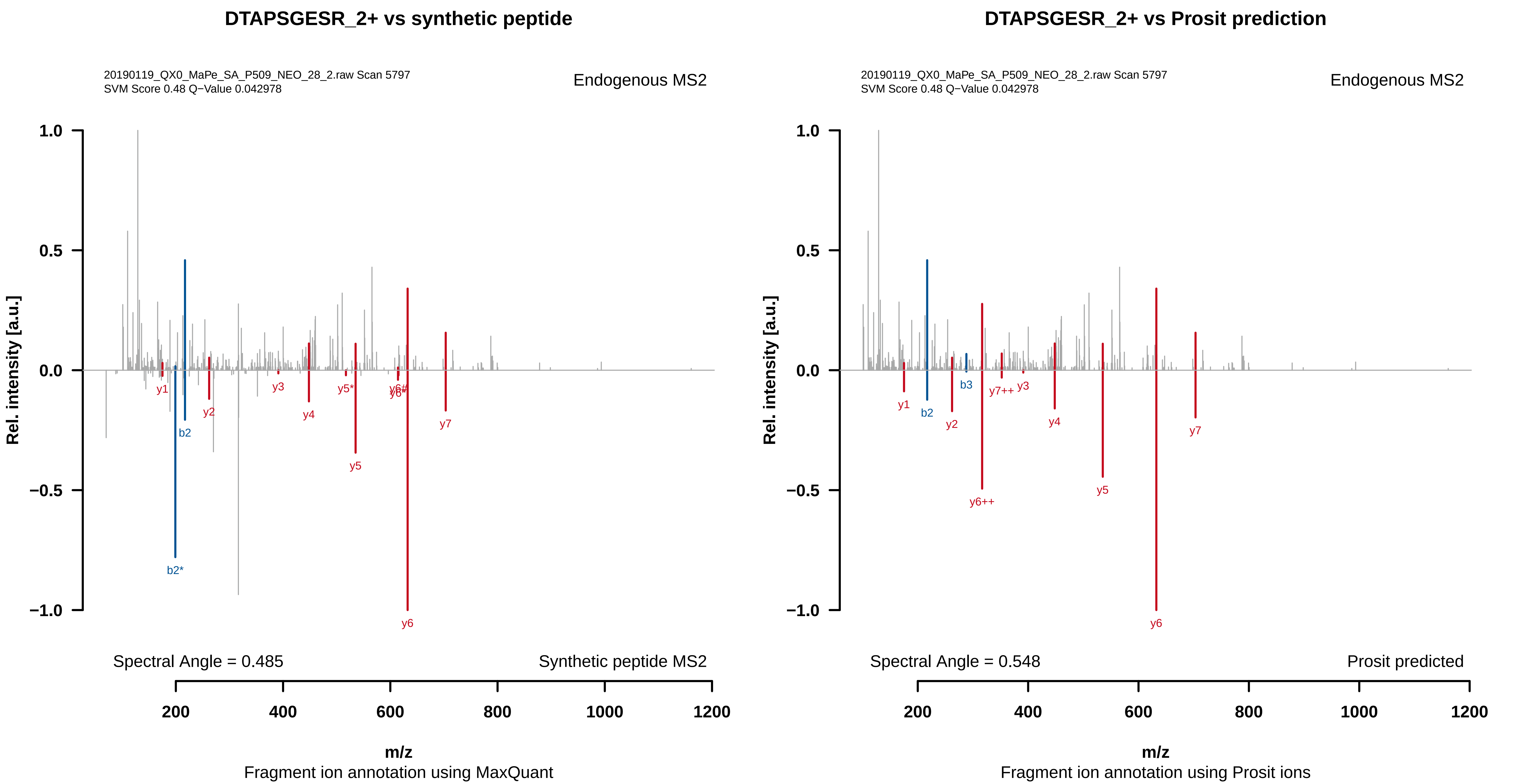
DTAPSGESR_2+ vs synthetic peptide
20190119_QX0_MaPe_SA_P509_NEO_28_2.raw Scan 5797
SVM Score 0.48 Q−Value 0.042978
Endogenous MS2
Rel. intensity [a.u.]
1.0
0.5
0.0
−0.5
−1.0
y1
b2
y2
y3
y4
y5*
y5
y7
y6
b2*
Spectral Angle = 0.485
Synthetic peptide MS2
200
400
600
800
1000
1200
m/z
Fragment ion annotation using MaxQuant
DTAPSGESR_2+ vs Prosit prediction
20190119_QX0_MaPe_SA_P509_NEO_28_2.raw Scan 5797
SVM Score 0.48 Q−Value 0.042978
Endogenous MS2
Rel. intensity [a.u.]
y1
b2
y2
b3
y7++
y3
y4
y6++
y5
y7
y6
Spectral Angle = 0.548
Prosit predicted
m/z
Fragment ion annotation using Prosit ions

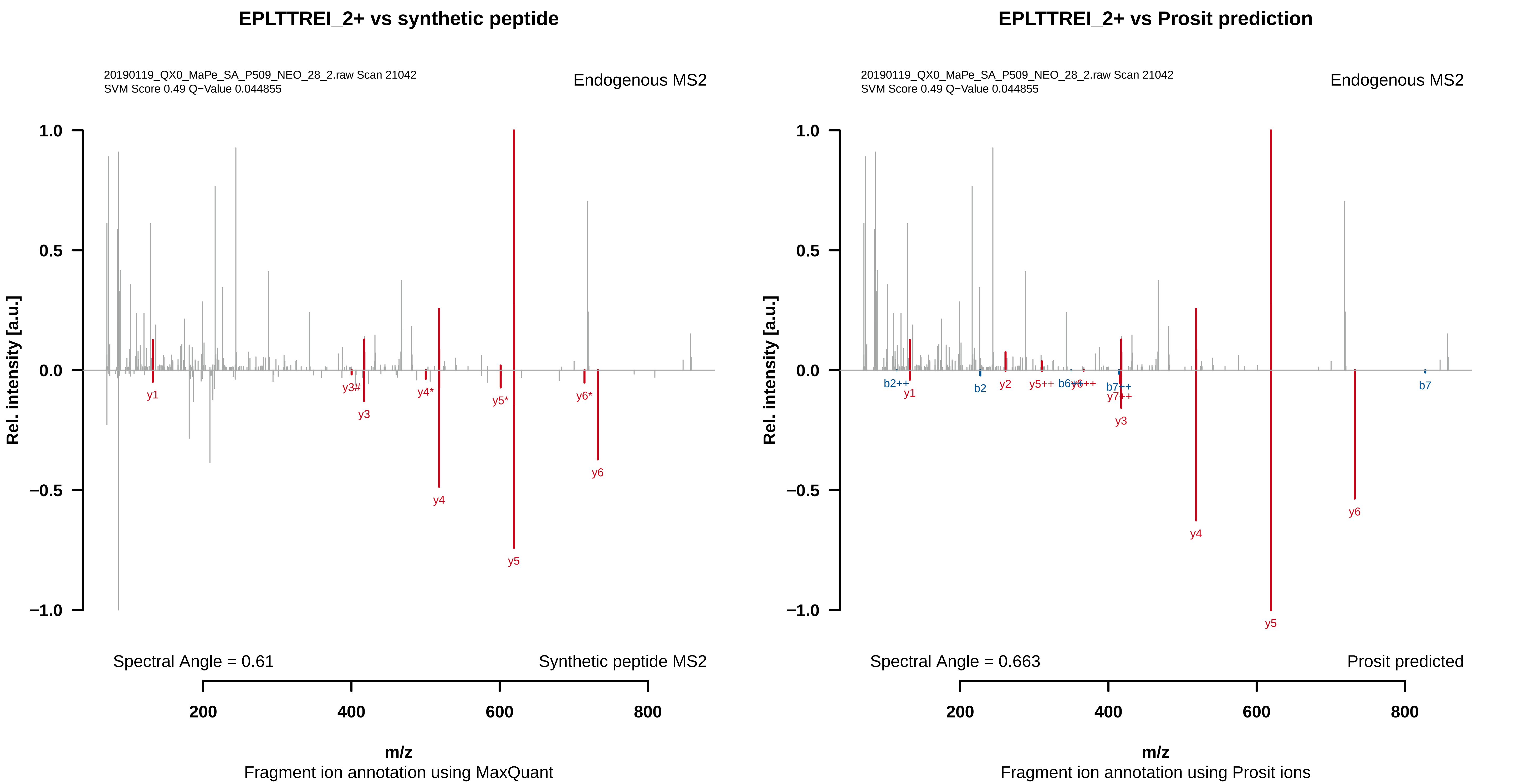

EPLTTREI_2+ vs synthetic peptide
20190119_QX0_MaPe_SA_P509_NEO_28_2.raw Scan 21042
SVM Score 0.49 Q−Value 0.044855
Endogenous MS2
Rel. intensity [a.u.]
1.0
0.5
0.0
−0.5
−1.0
y1
y3#
y3
y4*
y4
y5*
y5
y6*
y6
Spectral Angle = 0.61
Synthetic peptide MS2
200
400
600
800
m/z
Fragment ion annotation using MaxQuant
EPLTTREI_2+ vs Prosit prediction
20190119_QX0_MaPe_SA_P509_NEO_28_2.raw Scan 21042
SVM Score 0.49 Q−Value 0.044855
Endogenous MS2
Rel. intensity [a.u.]
b2++
y1
b2
y2
y5++
b6++
y6++
b7++
y7++
y3
y4
y5
y6
b7
Spectral Angle = 0.663
Prosit predicted
m/z
Fragment ion annotation using Prosit ions

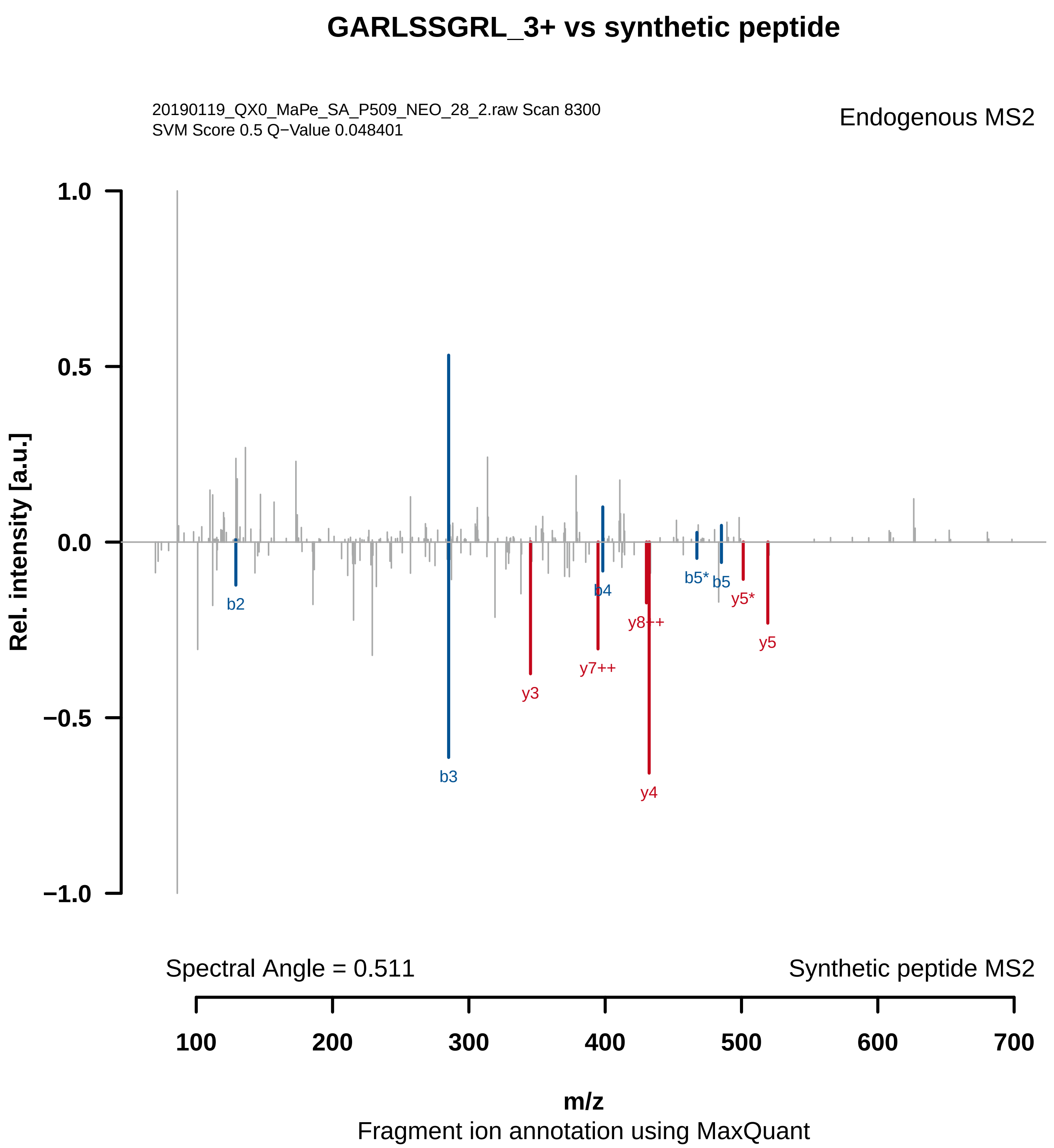
GARLSSGRL_3+ vs synthetic peptide
20190119_QX0_MaPe_SA_P509_NEO_28_2.raw Scan 8300
SVM Score 0.5 Q−Value 0.048401
Endogenous MS2
Rel. intensity [a.u.]
b2
b3
y3
y7++
b4
y8++
y4
b5*
b5
y5*
y5
Spectral Angle = 0.511
Synthetic peptide MS2
m/z
Fragment ion annotation using MaxQuant

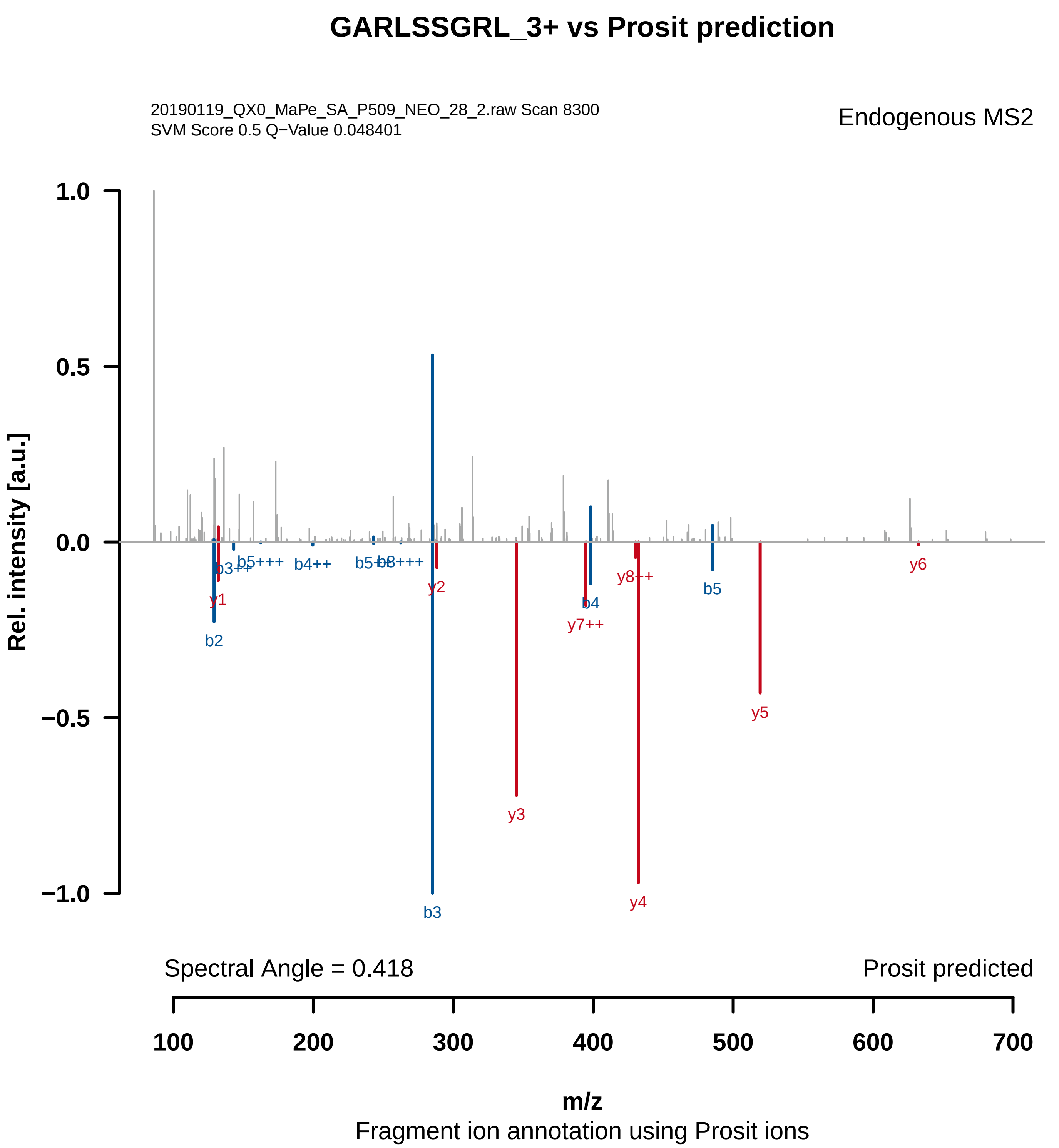
GARLSSGRL_3+ vs Prosit prediction
20190119_QX0_MaPe_SA_P509_NEO_28_2.raw Scan 8300
SVM Score 0.5 Q−Value 0.048401
Endogenous MS2
Rel. intensity [a.u.]
b2
y1
b3++
b5+++
b4++
b8+++
y2
b3
y3
b4
y7++
y8++
y4
b5
y5
y6
Spectral Angle = 0.418
Prosit predicted
m/z
Fragment ion annotation using Prosit ions

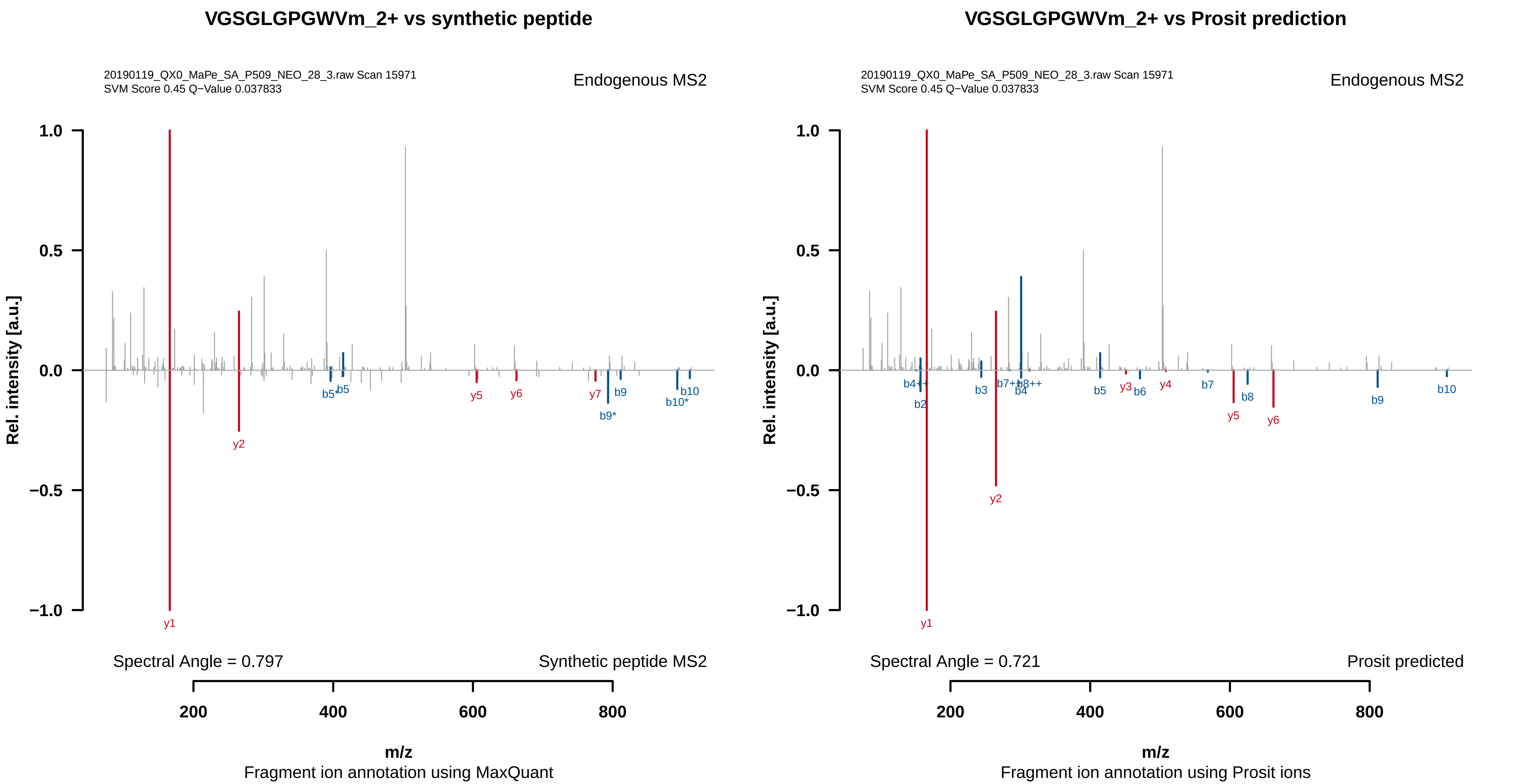

VGSGLGPGWVm_2+ vs synthetic peptide
20190119_QX0_MaPe_SA_P509_NEO_28_3.raw Scan 15971
SVM Score 0.45 Q−Value 0.037833
Endogenous MS2
Rel. intensity [a.u.]
1.0
0.5
0.0
−0.5
−1.0
y1
y2
b5*
b5
y5
y6
y7
b9
b9*
b10
b10*
Spectral Angle = 0.797
Synthetic peptide MS2
200
400
600
800
m/z
Fragment ion annotation using MaxQuant
VGSGLGPGWVm_2+ vs Prosit prediction
20190119_QX0_MaPe_SA_P509_NEO_28_3.raw Scan 15971
SVM Score 0.45 Q−Value 0.037833
Endogenous MS2
Rel. intensity [a.u.]
1.0
0.5
0.0
−0.5
−1.0
b4++
b2
b3
b7++
b8++
b4
b5
y3
b6
y4
b7
b8
y5
y6
b9
b10
y1
y2
Spectral Angle = 0.721
Prosit predicted
200
400
600
800
m/z
Fragment ion annotation using Prosit ions

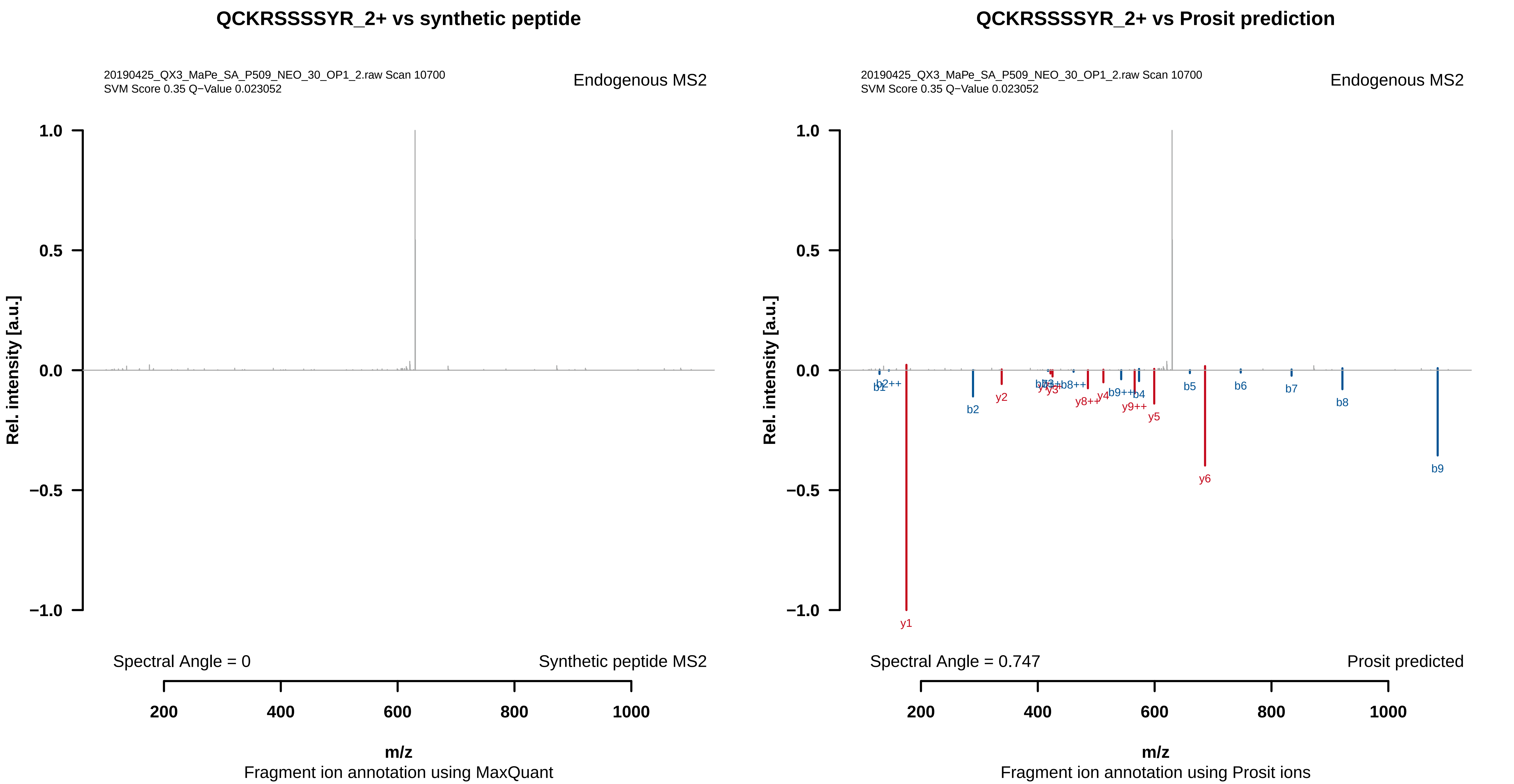
QCKRSSSSYR_2+ vs synthetic peptide
20190425_QX3_MaPe_SA_P509_NEO_30_OP1_2.raw Scan 10700
SVM Score 0.35 Q−Value 0.023052
Endogenous MS2
1.0
0.5
0.0
−0.5
−1.0
Rel. intensity [a.u.]
Spectral Angle = 0
Synthetic peptide MS2
200
400
600
800
1000
m/z
Fragment ion annotation using MaxQuant
QCKRSSSSYR_2+ vs Prosit prediction
20190425_QX3_MaPe_SA_P509_NEO_30_OP1_2.raw Scan 10700
SVM Score 0.35 Q−Value 0.023052
Endogenous MS2
1.0
0.5
0.0
−0.5
−1.0
Rel. intensity [a.u.]
b1
b2++
y1
b2
y2
y3
b8++
y8++
y4
b9++
b4
y9++
y5
b5
y6
b6
b7
b8
b9
Spectral Angle = 0.747
Prosit predicted
200
400
600
800
1000
m/z
Fragment ion annotation using Prosit ions

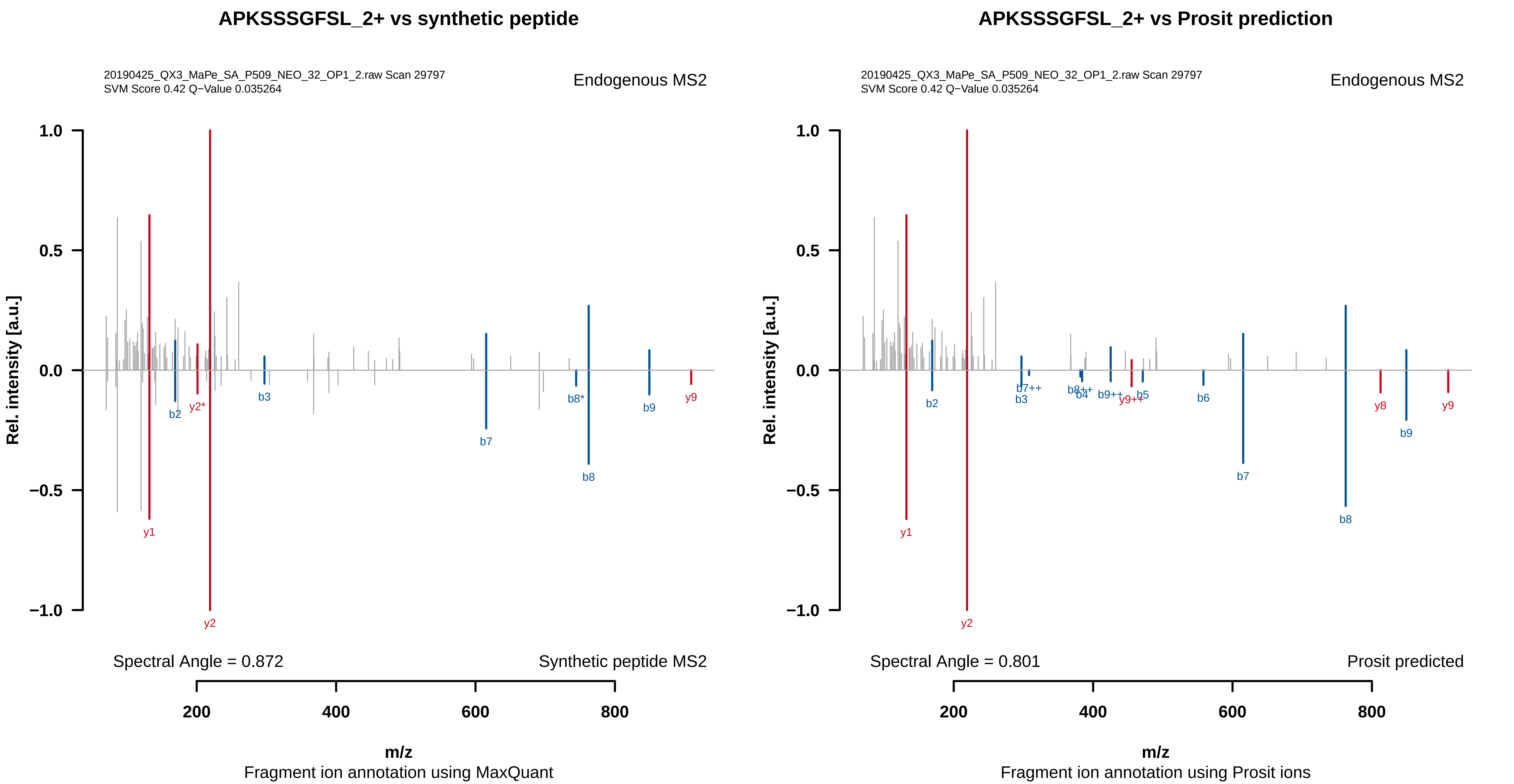

APKSSSGFSL_2+ vs synthetic peptide
20190425_QX3_MaPe_SA_P509_NEO_32_OP1_2.raw Scan 29797
SVM Score 0.42 Q−Value 0.035264
Endogenous MS2
Rel. intensity [a.u.]
1.0
0.5
0.0
−0.5
−1.0
y1
b2
y2*
y2
b3
b7
b8*
b8
b9
y9
Spectral Angle = 0.872
Synthetic peptide MS2
200
400
600
800
m/z
Fragment ion annotation using MaxQuant
APKSSSGFSL_2+ vs Prosit prediction
20190425_QX3_MaPe_SA_P509_NEO_32_OP1_2.raw Scan 29797
SVM Score 0.42 Q−Value 0.035264
Endogenous MS2
Rel. intensity [a.u.]
1.0
0.5
0.0
−0.5
−1.0
y1
b2
y2
b7++
b3
b8++
b4
b9++
y9++
b5
b6
b7
b8
y8
b9
y9
Spectral Angle = 0.801
Prosit predicted
200
400
600
800
m/z
Fragment ion annotation using Prosit ions

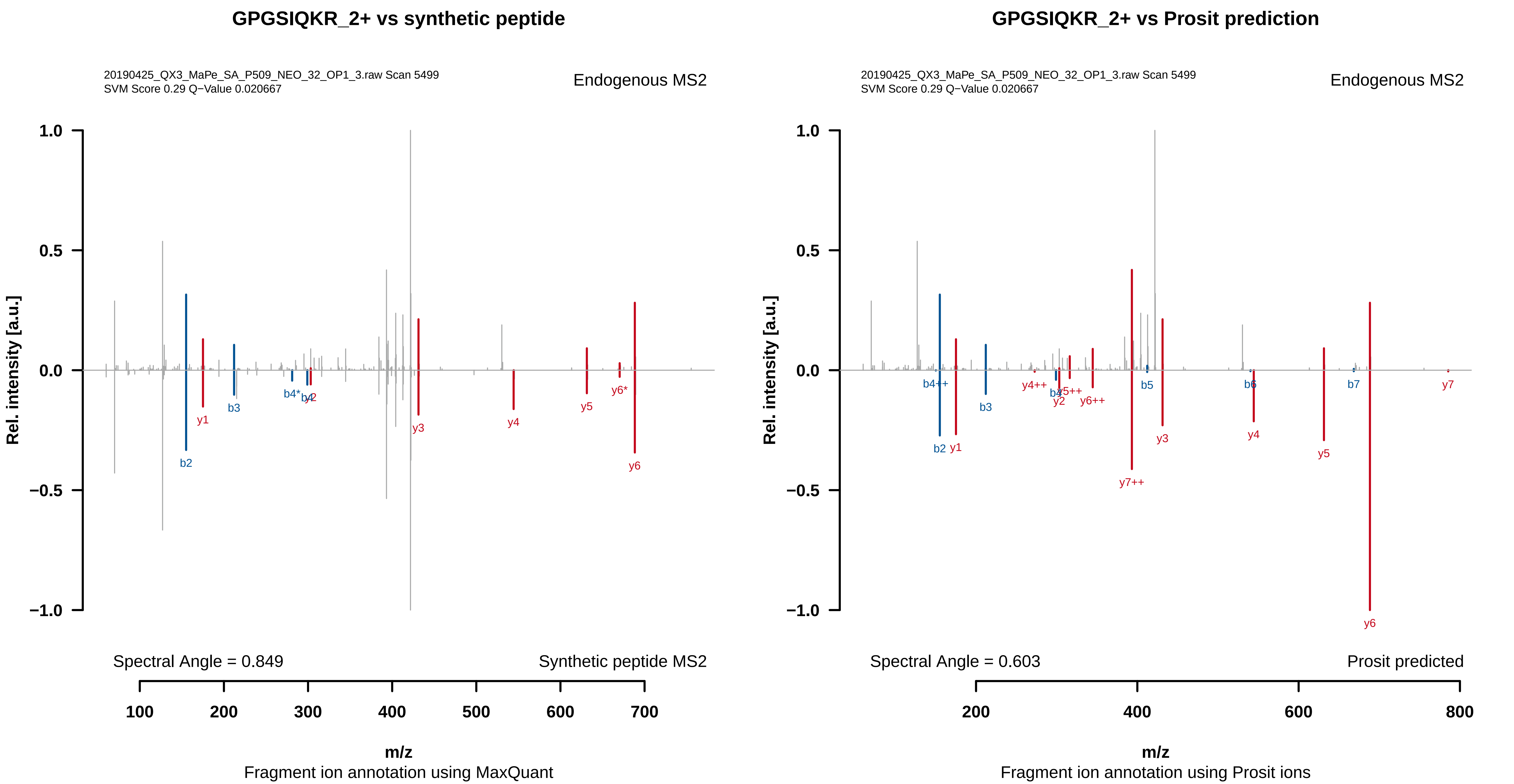
GPGSIQKR_2+ vs synthetic peptide
20190425_QX3_MaPe_SA_P509_NEO_32_OP1_3.raw Scan 5499
SVM Score 0.29 Q−Value 0.020667
Endogenous MS2
Rel. intensity [a.u.]
1.0
0.5
0.0
−0.5
−1.0
b2
y1
b3
b4*
b4
y2
y3
y4
y5
y6*
y6
Spectral Angle = 0.849
Synthetic peptide MS2
100
200
300
400
500
600
700
m/z
Fragment ion annotation using MaxQuant
GPGSIQKR_2+ vs Prosit prediction
20190425_QX3_MaPe_SA_P509_NEO_32_OP1_3.raw Scan 5499
SVM Score 0.29 Q−Value 0.020667
Endogenous MS2
Rel. intensity [a.u.]
1.0
0.5
0.0
−0.5
−1.0
b4++
b2
y1
b3
y4++
b4
y2
y5++
y6++
y7++
b5
y3
b6
y4
y5
b7
y6
y7
Spectral Angle = 0.603
Prosit predicted
200
400
600
800
m/z
Fragment ion annotation using Prosit ions

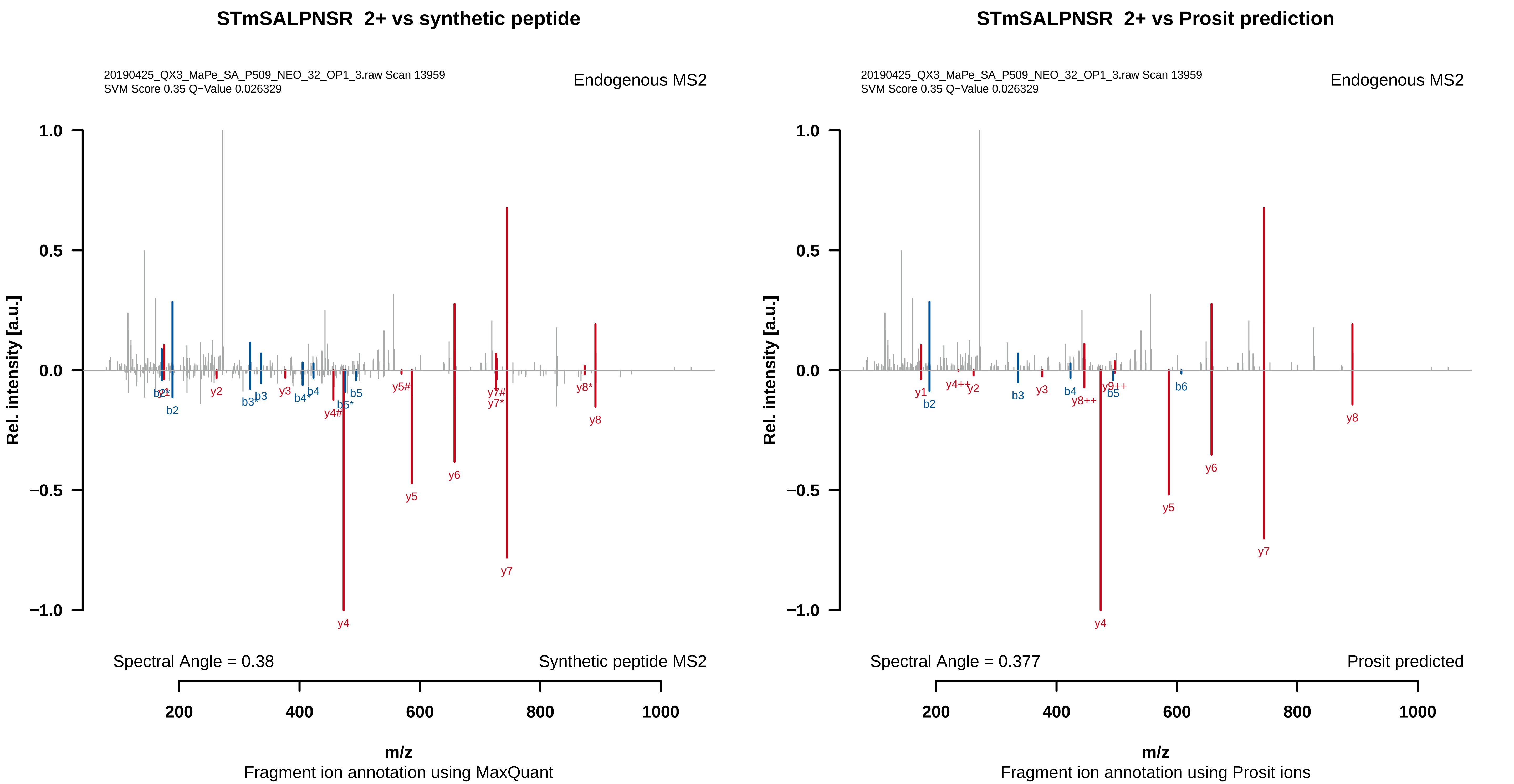

STmSALPNSR_2+ vs synthetic peptide
20190425_QX3_MaPe_SA_P509_NEO_32_OP1_3.raw Scan 13959
SVM Score 0.35 Q−Value 0.026329
Endogenous MS2
Rel. intensity [a.u.]
Spectral Angle = 0.38
Synthetic peptide MS2
m/z
Fragment ion annotation using MaxQuant
STmSALPNSR_2+ vs Prosit prediction
20190425_QX3_MaPe_SA_P509_NEO_32_OP1_3.raw Scan 13959
SVM Score 0.35 Q−Value 0.026329
Endogenous MS2
Rel. intensity [a.u.]
Spectral Angle = 0.377
Prosit predicted
m/z
Fragment ion annotation using Prosit ions

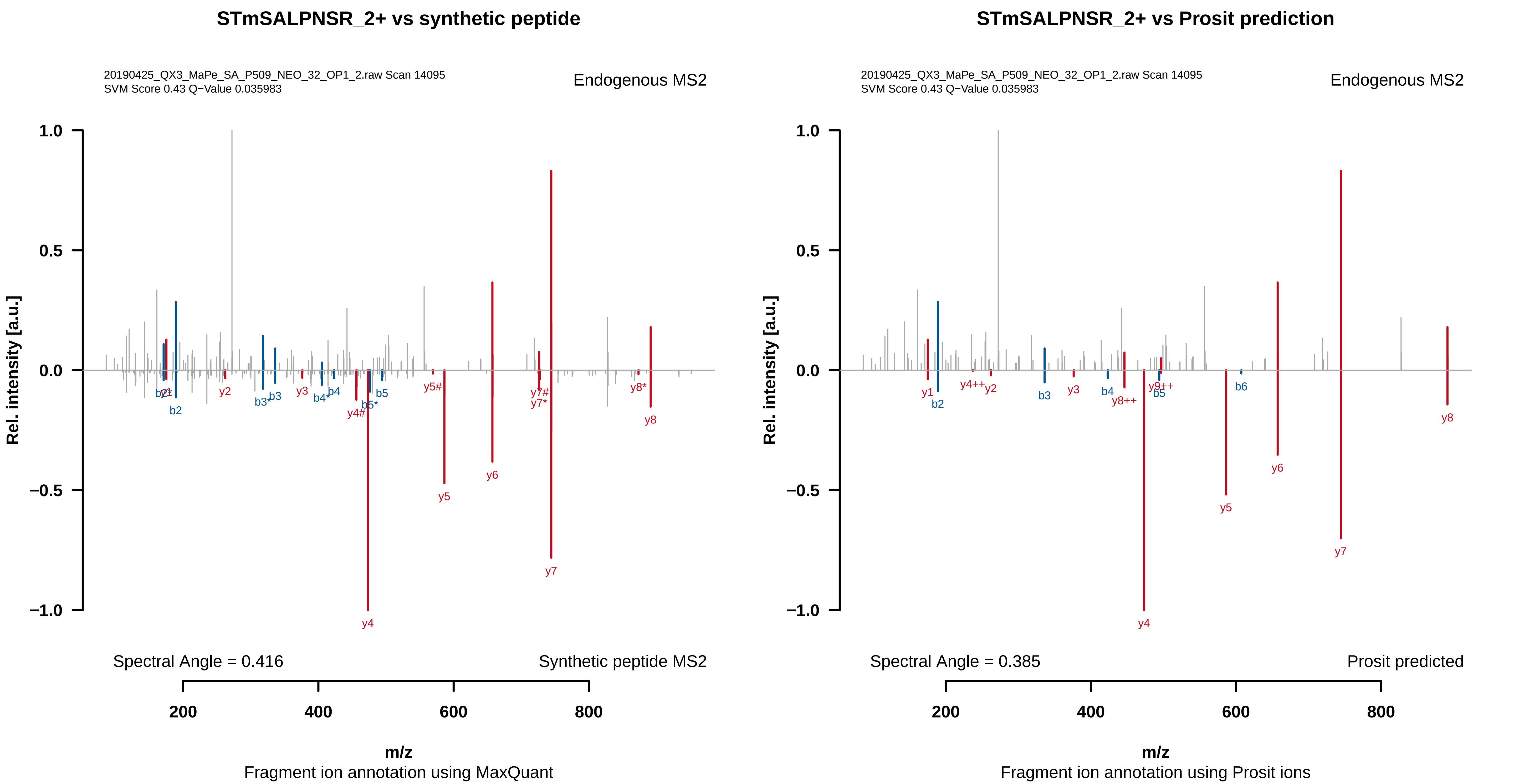
STmSALPNSR_2+ vs synthetic peptide
20190425_QX3_MaPe_SA_P509_NEO_32_OP1_2.raw Scan 14095
SVM Score 0.43 Q−Value 0.035983
Endogenous MS2
Rel. intensity [a.u.]
1.0
0.5
0.0
−0.5
−1.0
Spectral Angle = 0.416
Synthetic peptide MS2
200
400
600
800
m/z
Fragment ion annotation using MaxQuant
STmSALPNSR_2+ vs Prosit prediction
20190425_QX3_MaPe_SA_P509_NEO_32_OP1_2.raw Scan 14095
SVM Score 0.43 Q−Value 0.035983
Endogenous MS2
Rel. intensity [a.u.]
Spectral Angle = 0.385
Prosit predicted
m/z
Fragment ion annotation using Prosit ions

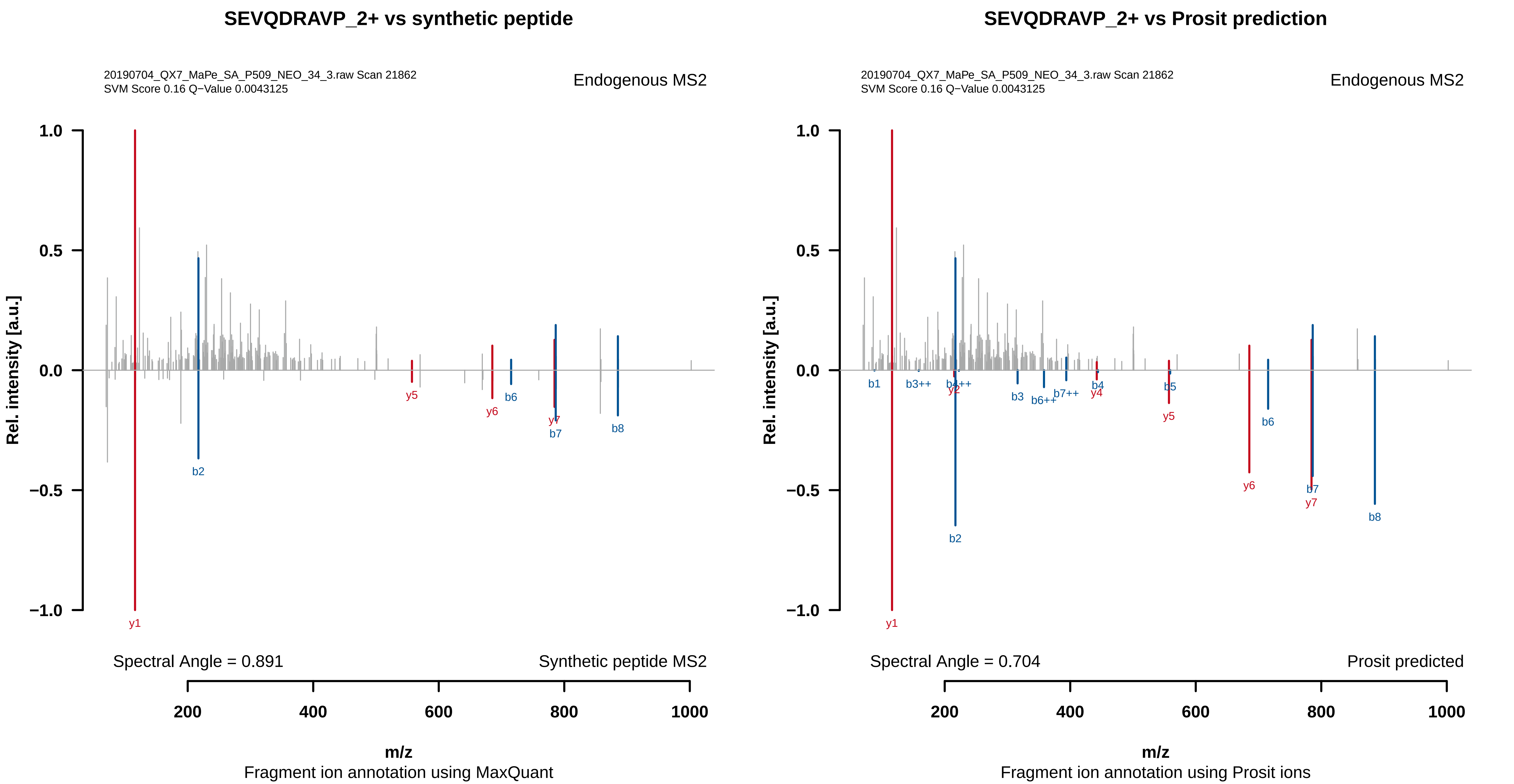
SEVQDRAVP_2+ vs synthetic peptide
20190704_QX7_MaPe_SA_P509_NEO_34_3.raw Scan 21862
SVM Score 0.16 Q−Value 0.0043125
Endogenous MS2
Rel. intensity [a.u.]
1.0
0.5
0.0
−0.5
−1.0
y1
b2
y5
y6
b6
y7
b7
b8
Spectral Angle = 0.891
Synthetic peptide MS2
200
400
600
800
1000
m/z
Fragment ion annotation using MaxQuant
SEVQDRAVP_2+ vs Prosit prediction
20190704_QX7_MaPe_SA_P509_NEO_34_3.raw Scan 21862
SVM Score 0.16 Q−Value 0.0043125
Endogenous MS2
Rel. intensity [a.u.]
b1
b3++
b4++
y2
b3
b6++
b7++
b4
y4
b5
y5
b6
y6
b7
y7
b8
b2
y1
Spectral Angle = 0.704
Prosit predicted
m/z
Fragment ion annotation using Prosit ions

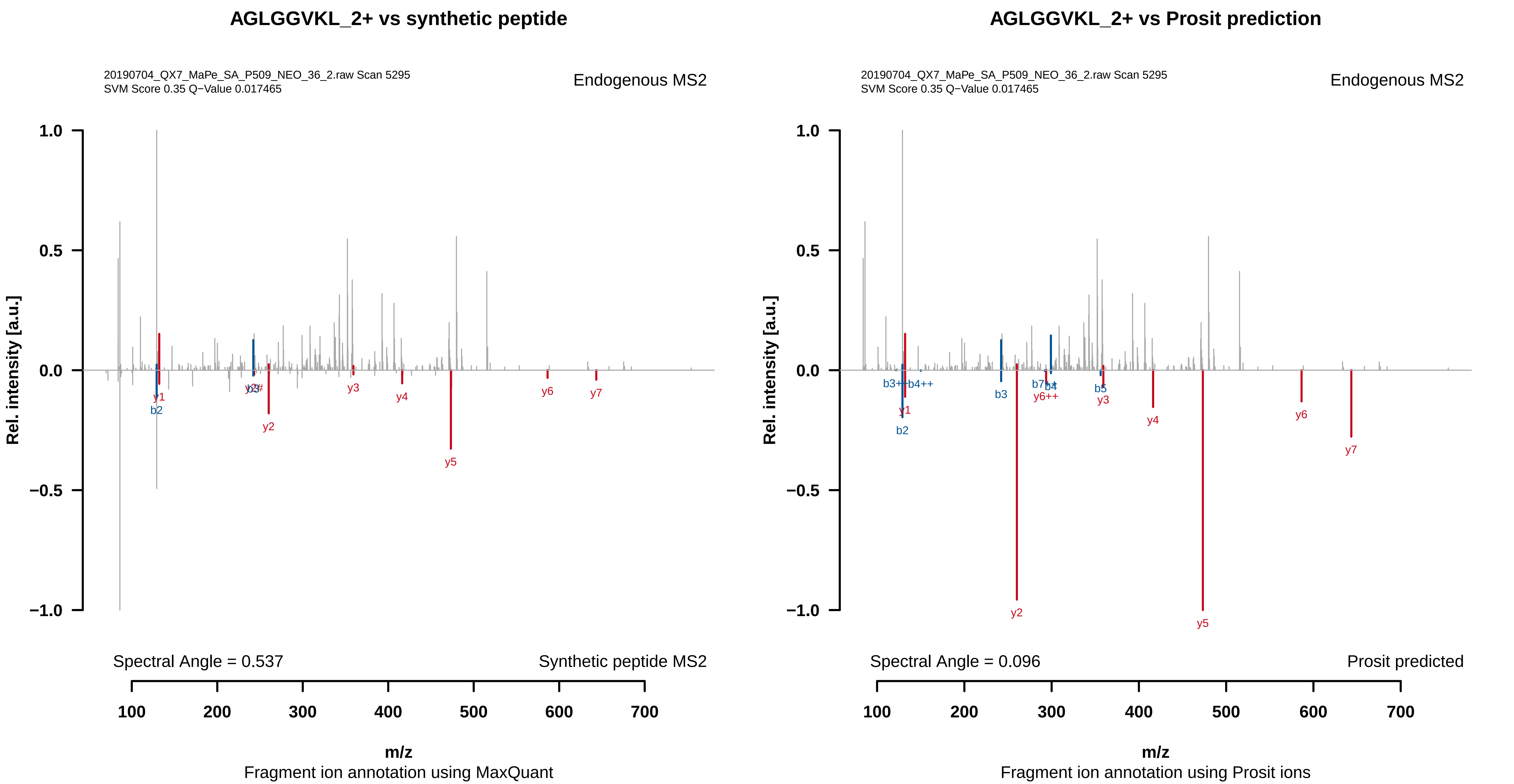

AGLGGVKL_2+ vs synthetic peptide
20190704_QX7_MaPe_SA_P509_NEO_36_2.raw Scan 5295
SVM Score 0.35 Q−Value 0.017465
Endogenous MS2
Rel. intensity [a.u.]
1.0
0.5
0.0
−0.5
−1.0
y1
b2
y3#
b3
y2
y3
y4
y5
y6
y7
Spectral Angle = 0.537
Synthetic peptide MS2
100
200
300
400
500
600
700
m/z
Fragment ion annotation using MaxQuant
AGLGGVKL_2+ vs Prosit prediction
20190704_QX7_MaPe_SA_P509_NEO_36_2.raw Scan 5295
SVM Score 0.35 Q−Value 0.017465
Endogenous MS2
Rel. intensity [a.u.]
1.0
0.5
0.0
−0.5
−1.0
b3++
b4++
y1
b2
b3
b7++
b4
y6++
b5
y3
y2
y4
y5
y6
y7
Spectral Angle = 0.096
Prosit predicted
100
200
300
400
500
600
700
m/z
Fragment ion annotation using Prosit ions

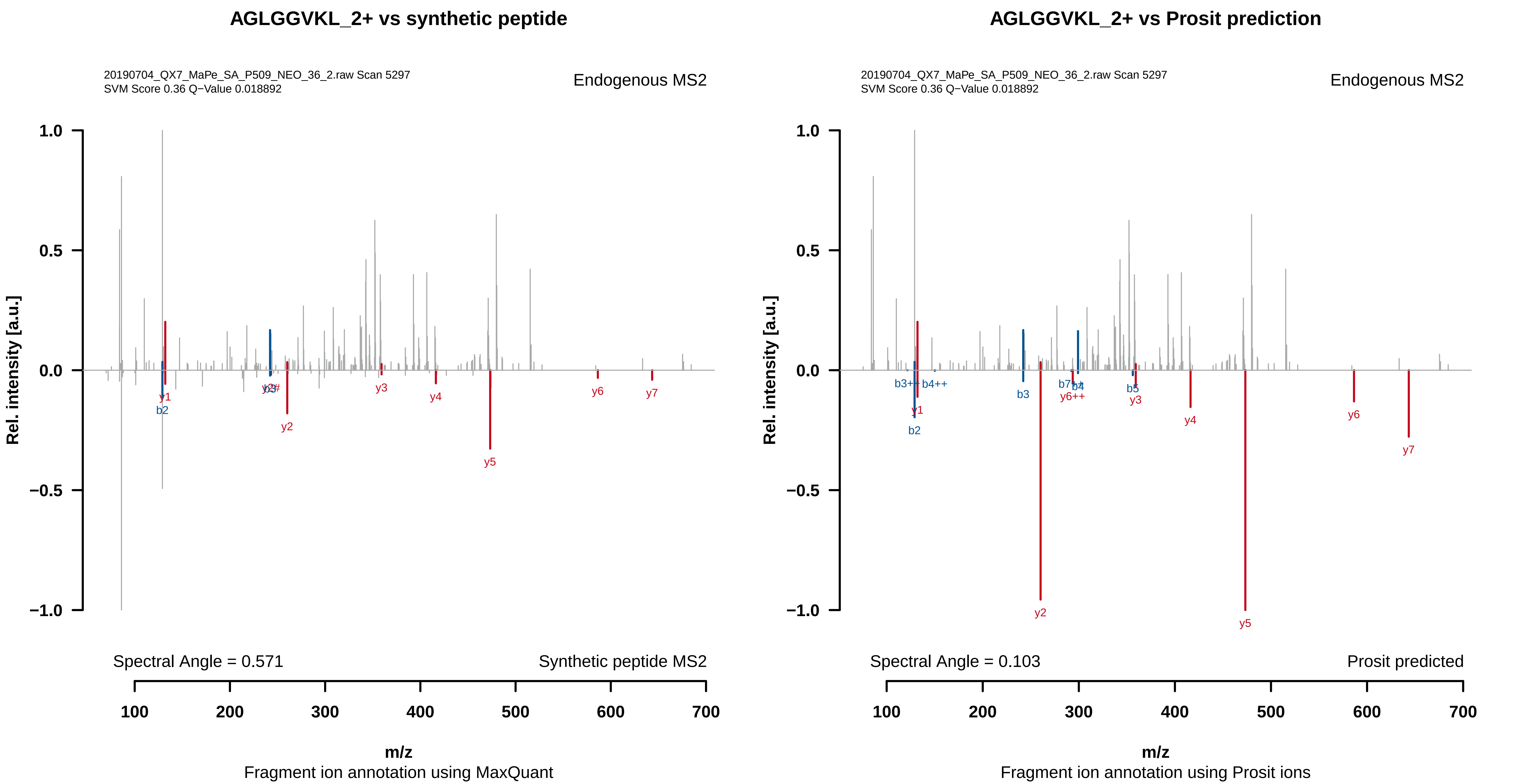

AGLGGVKL_2+ vs synthetic peptide
20190704_QX7_MaPe_SA_P509_NEO_36_2.raw Scan 5297
SVM Score 0.36 Q−Value 0.018892
Endogenous MS2
Rel. intensity [a.u.]
1.0
0.5
0.0
−0.5
−1.0
y1
b2
y2
y3
y4
y5
y6
y7
Spectral Angle = 0.571
Synthetic peptide MS2
100
200
300
400
500
600
700
m/z
Fragment ion annotation using MaxQuant
AGLGGVKL_2+ vs Prosit prediction
20190704_QX7_MaPe_SA_P509_NEO_36_2.raw Scan 5297
SVM Score 0.36 Q−Value 0.018892
Endogenous MS2
Rel. intensity [a.u.]
b3++
b4++
b3
b4
b5
y6++
y1
b2
y2
y3
y4
y5
y6
y7
Spectral Angle = 0.103
Prosit predicted
m/z
Fragment ion annotation using Prosit ions

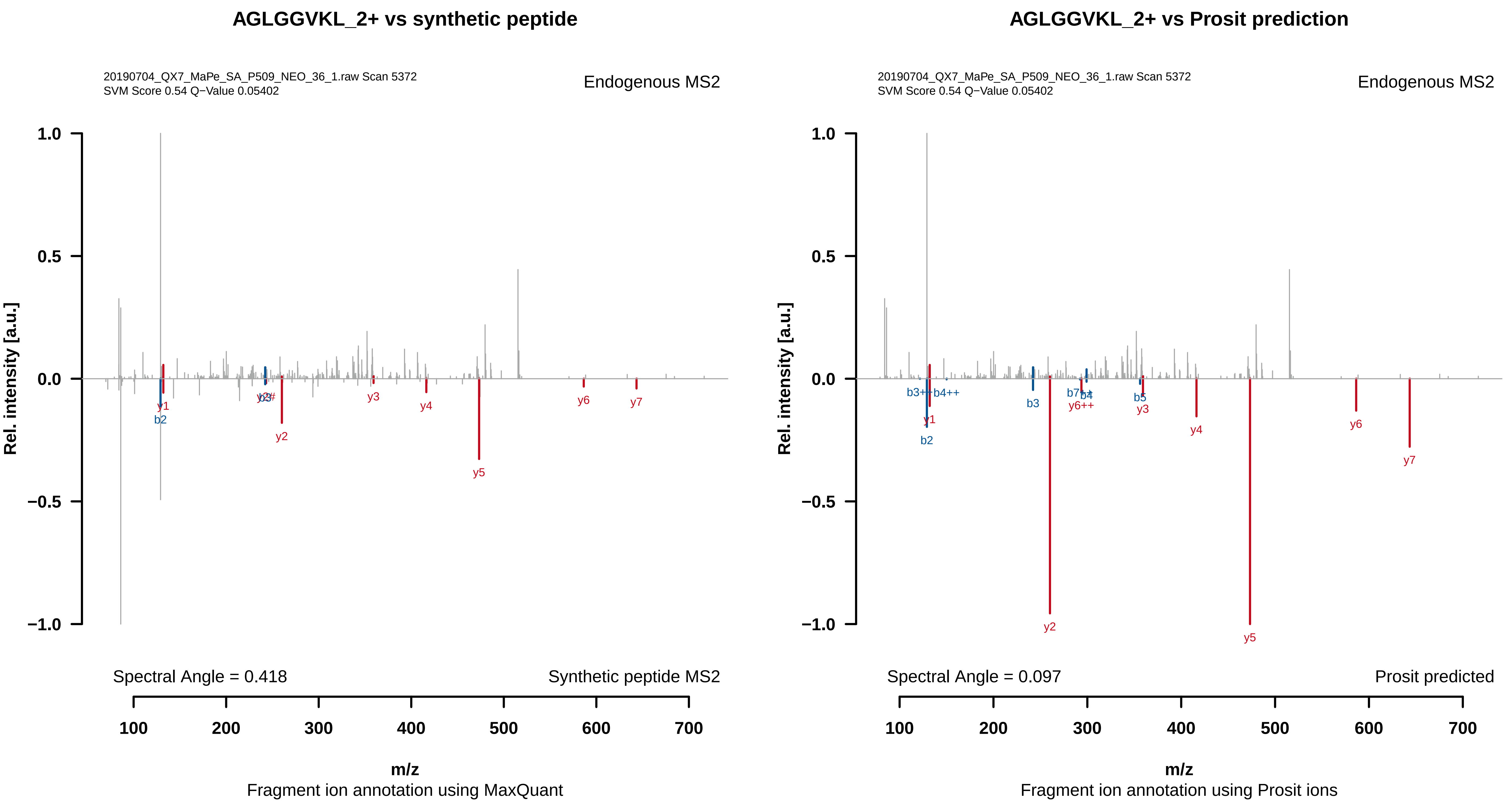
AGLGGVKL_2+ vs synthetic peptide
20190704_QX7_MaPe_SA_P509_NEO_36_1.raw Scan 5372
SVM Score 0.54 Q−Value 0.05402
Endogenous MS2
Rel. intensity [a.u.]
1.0
0.5
0.0
−0.5
−1.0
y1
b2
y2
y3
y4
y5
y6
y7
Spectral Angle = 0.418
Synthetic peptide MS2
100
200
300
400
500
600
700
m/z
Fragment ion annotation using MaxQuant
AGLGGVKL_2+ vs Prosit prediction
20190704_QX7_MaPe_SA_P509_NEO_36_1.raw Scan 5372
SVM Score 0.54 Q−Value 0.05402
Endogenous MS2
Rel. intensity [a.u.]
1.0
0.5
0.0
−0.5
−1.0
b4++
y1
b2
b3
y6++
b5
y3
y4
y2
y5
y6
y7
Spectral Angle = 0.097
Prosit predicted
100
200
300
400
500
600
700
m/z
Fragment ion annotation using Prosit ions

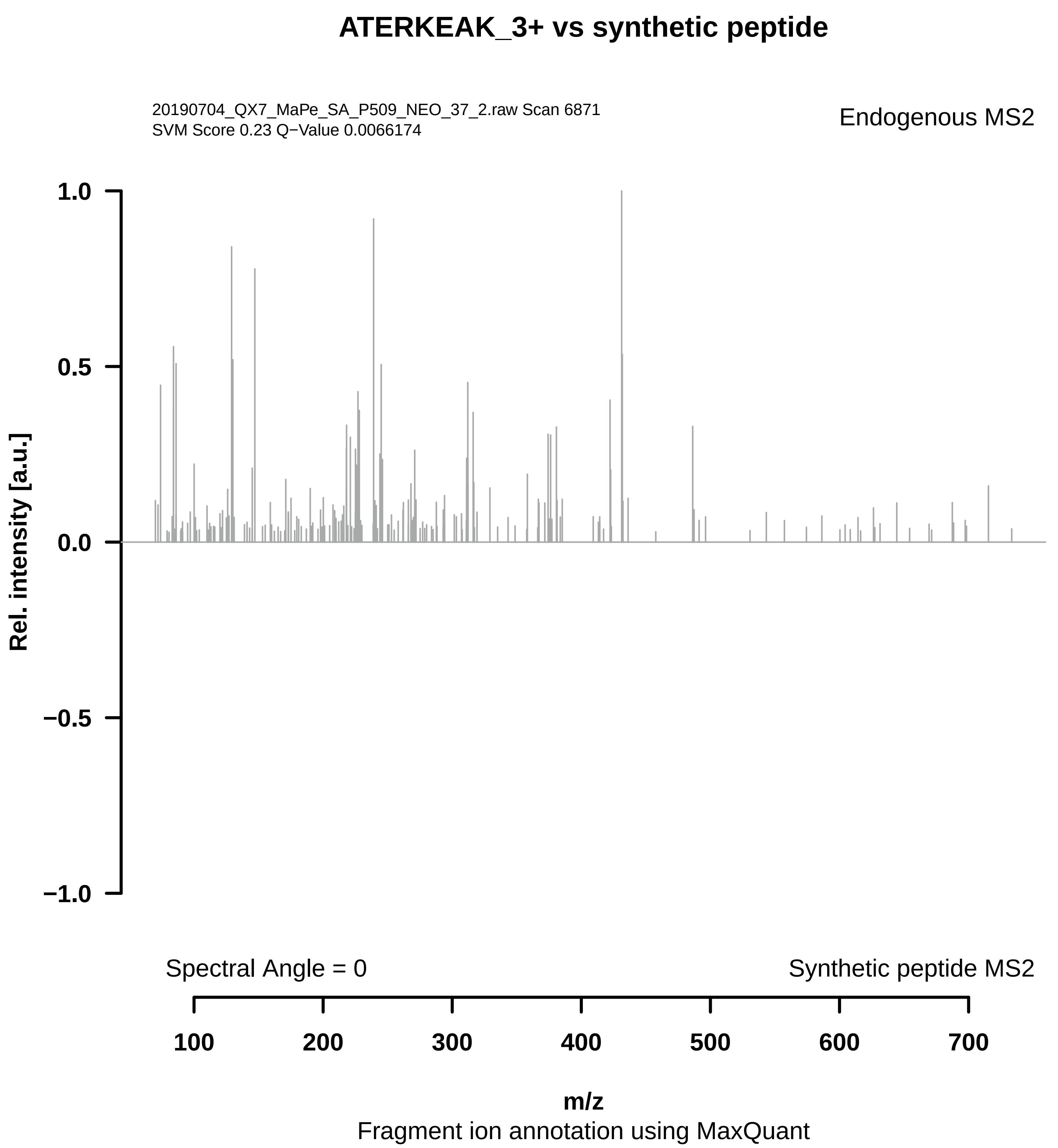
ATERKEAK_3+ vs synthetic peptide
20190704_QX7_MaPe_SA_P509_NEO_37_2.raw Scan 6871
SVM Score 0.23 Q−Value 0.0066174
Endogenous MS2
1.0
0.5
0.0
−0.5
−1.0
Rel. intensity [a.u.]
Spectral Angle = 0
Synthetic peptide MS2
100
200
300
400
500
600
700
m/z
Fragment ion annotation using MaxQuant

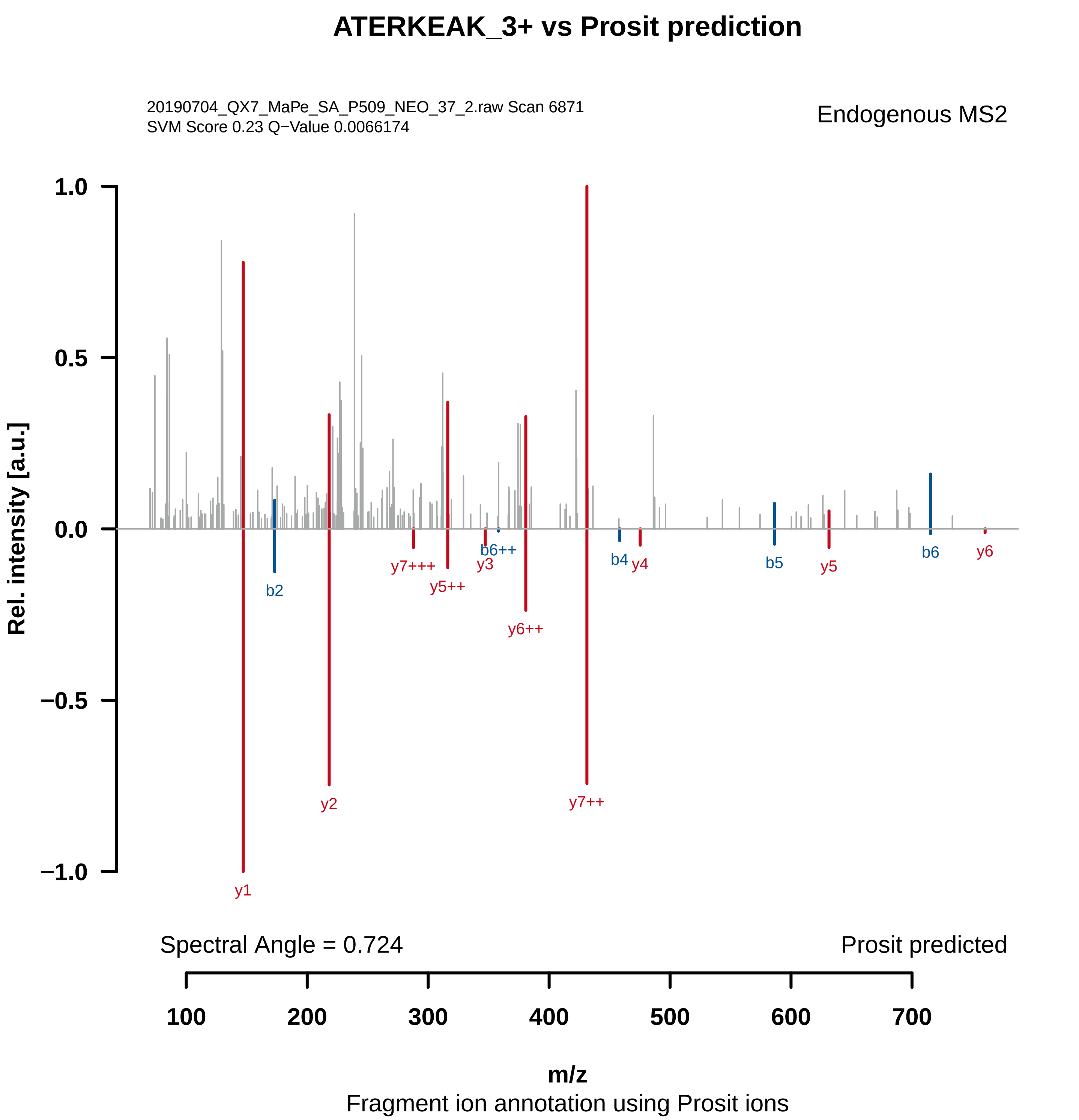
ATERKEAK_3+ vs Prosit prediction
20190704_QX7_MaPe_SA_P509_NEO_37_2.raw Scan 6871
SVM Score 0.23 Q−Value 0.0066174
Endogenous MS2
1.0
0.5
0.0
−0.5
−1.0
Rel. intensity [a.u.]
b2
y7+++
y5++
y3
b6++
y6++
b4
y4
b5
y5
b6
y6
y2
y7++
y1
Spectral Angle = 0.724
Prosit predicted
100
200
300
400
500
600
700
m/z
Fragment ion annotation using Prosit ions

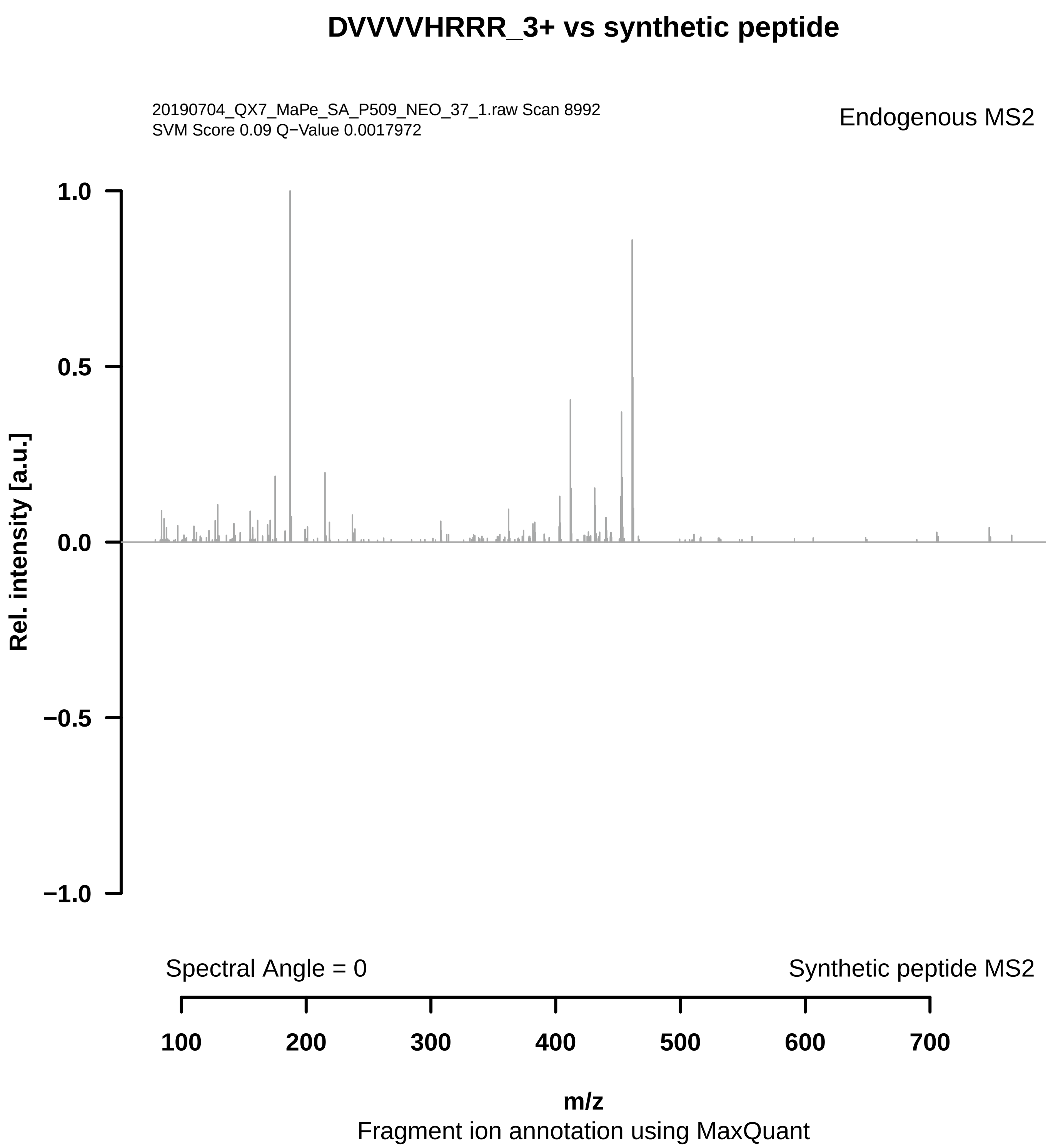

DVVVVHRRR_3+ vs synthetic peptide
20190704_QX7_MaPe_SA_P509_NEO_37_1.raw Scan 8992
SVM Score 0.09 Q−Value 0.0017972
Endogenous MS2
Rel. intensity [a.u.]
1.0
0.5
0.0
−0.5
−1.0
Spectral Angle = 0
Synthetic peptide MS2
100
200
300
400
500
600
700
m/z
Fragment ion annotation using MaxQuant

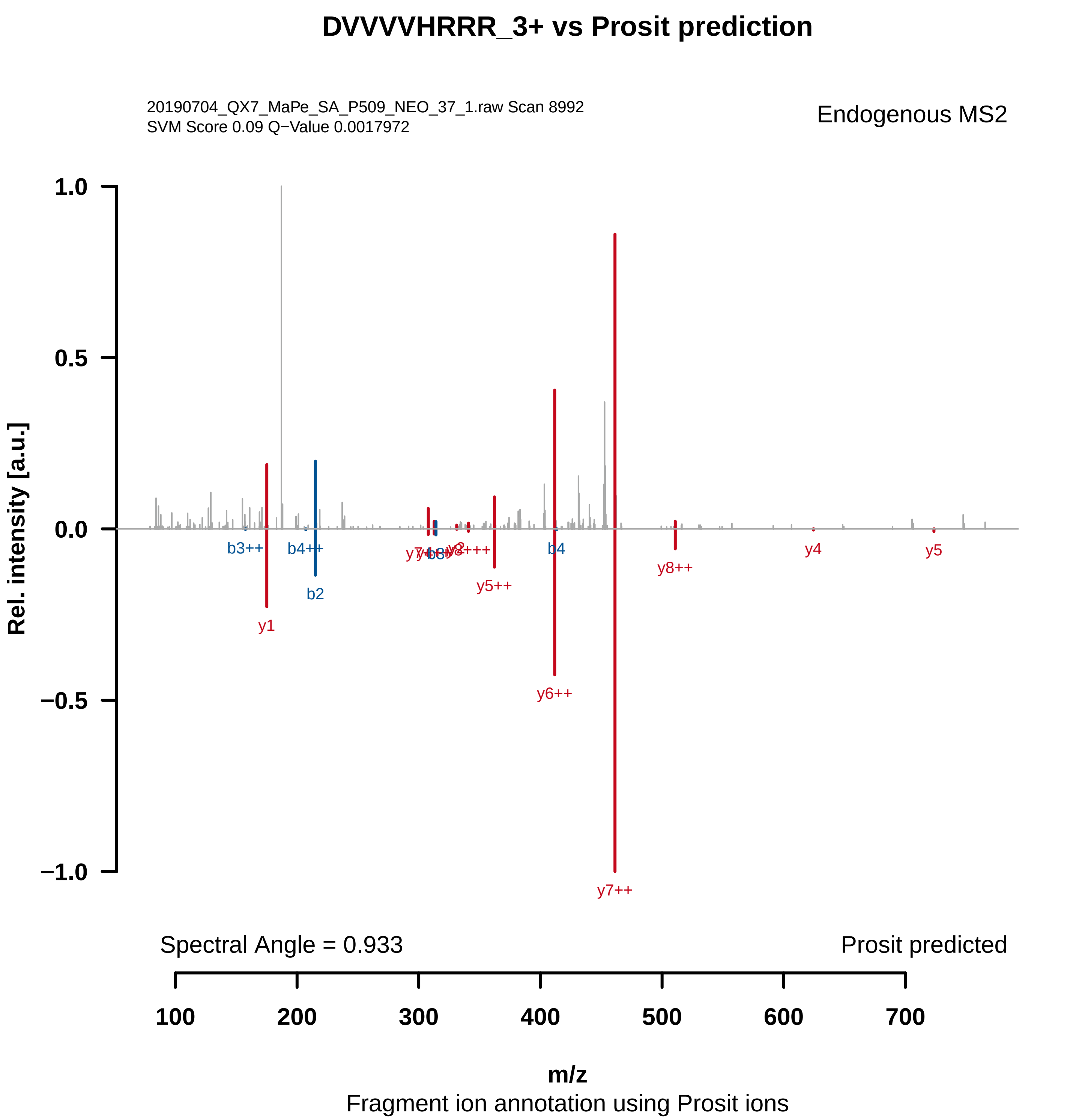

DVVVVHRRR_3+ vs Prosit prediction
20190704_QX7_MaPe_SA_P509_NEO_37_1.raw Scan 8992
SVM Score 0.09 Q−Value 0.0017972
Endogenous MS2
Rel. intensity [a.u.]
1.0
0.5
0.0
−0.5
−1.0
b3++
b4++
b2
y1
y5++
b4
y6++
y8++
y4
y5
y7++
Spectral Angle = 0.933
Prosit predicted
100
200
300
400
500
600
700
m/z
Fragment ion annotation using Prosit ions

DVVVVHRRR_3+ vs synthetic peptide

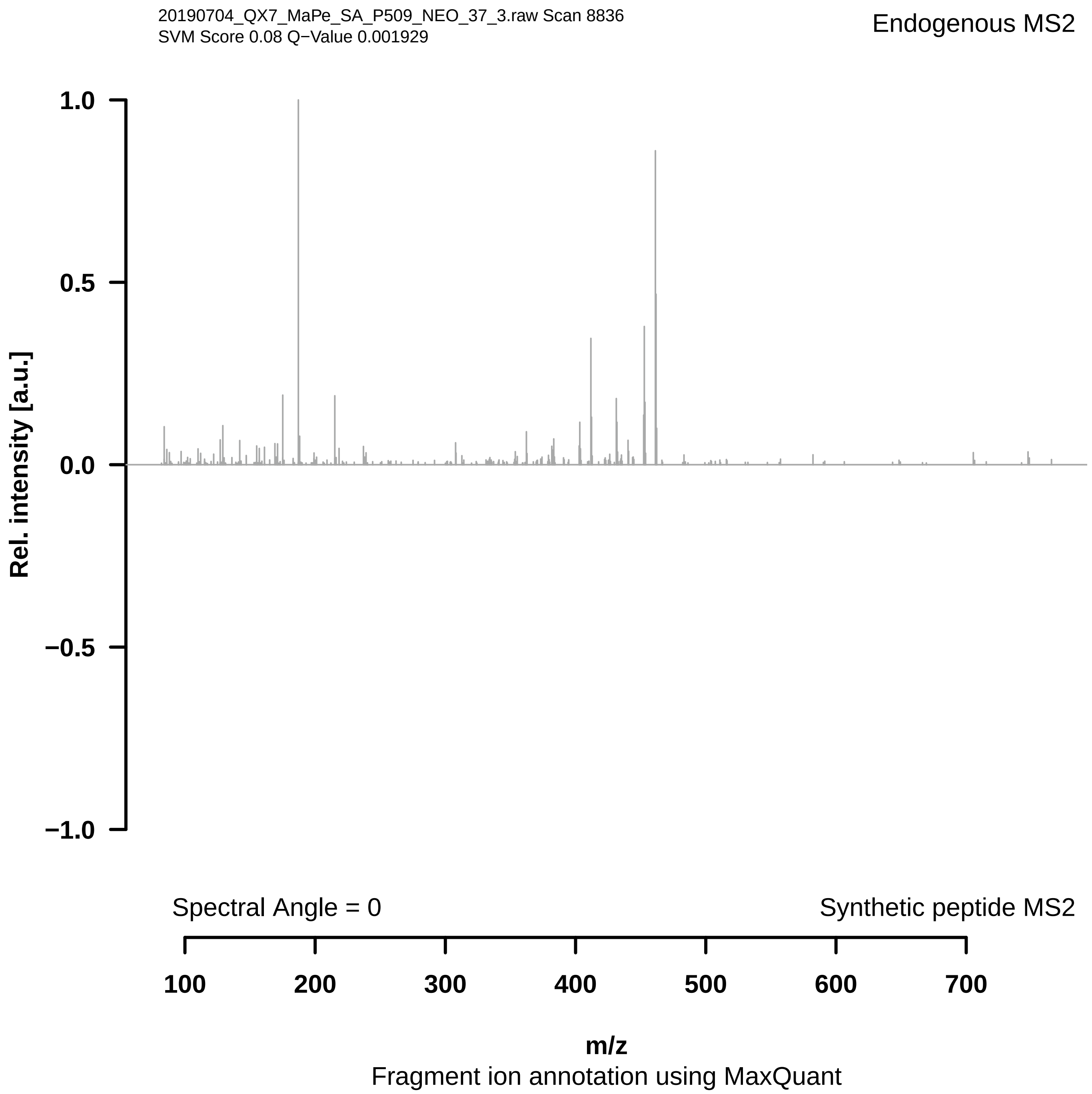

DVVVVHRRR_3+ vs Prosit prediction

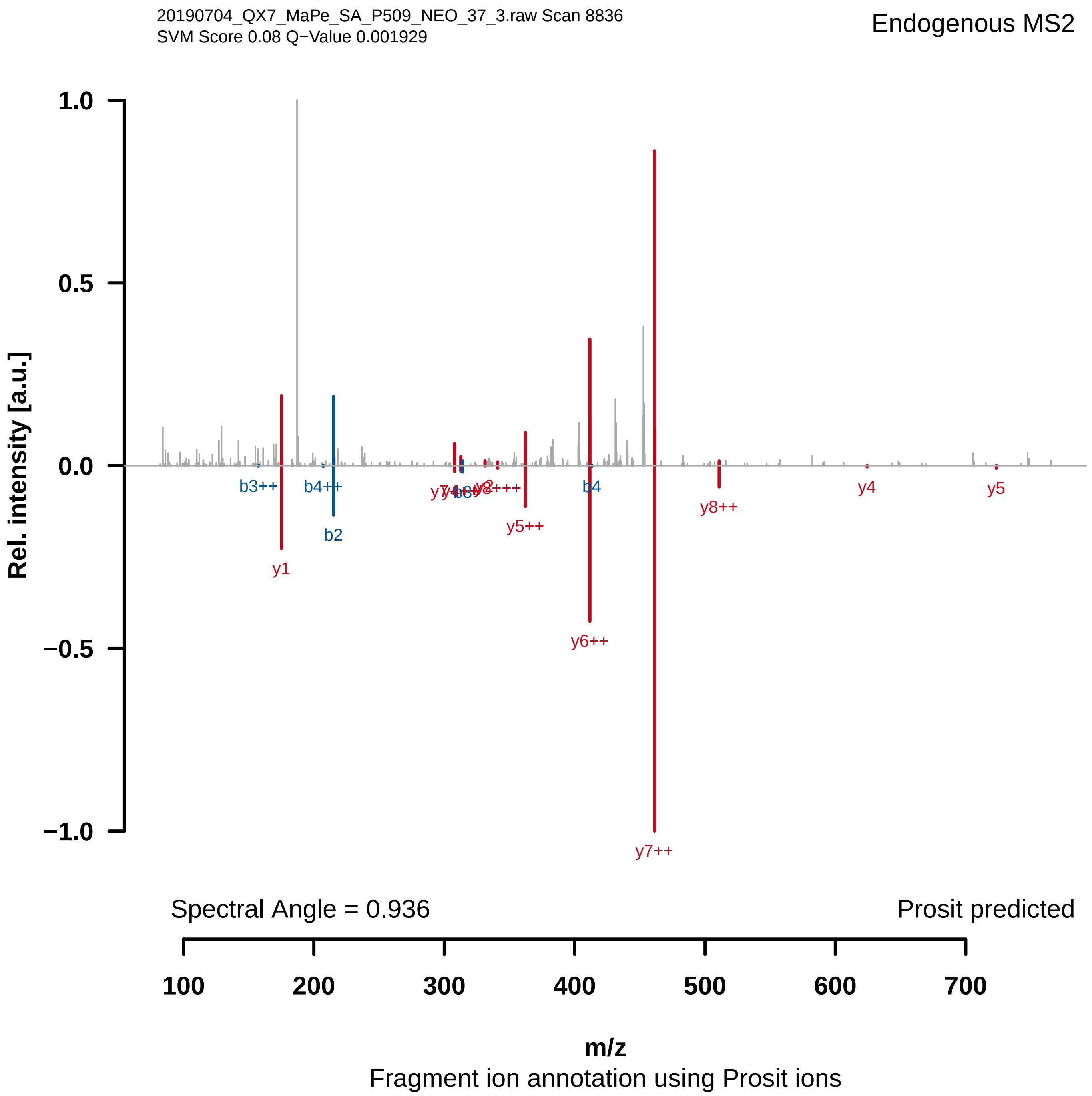

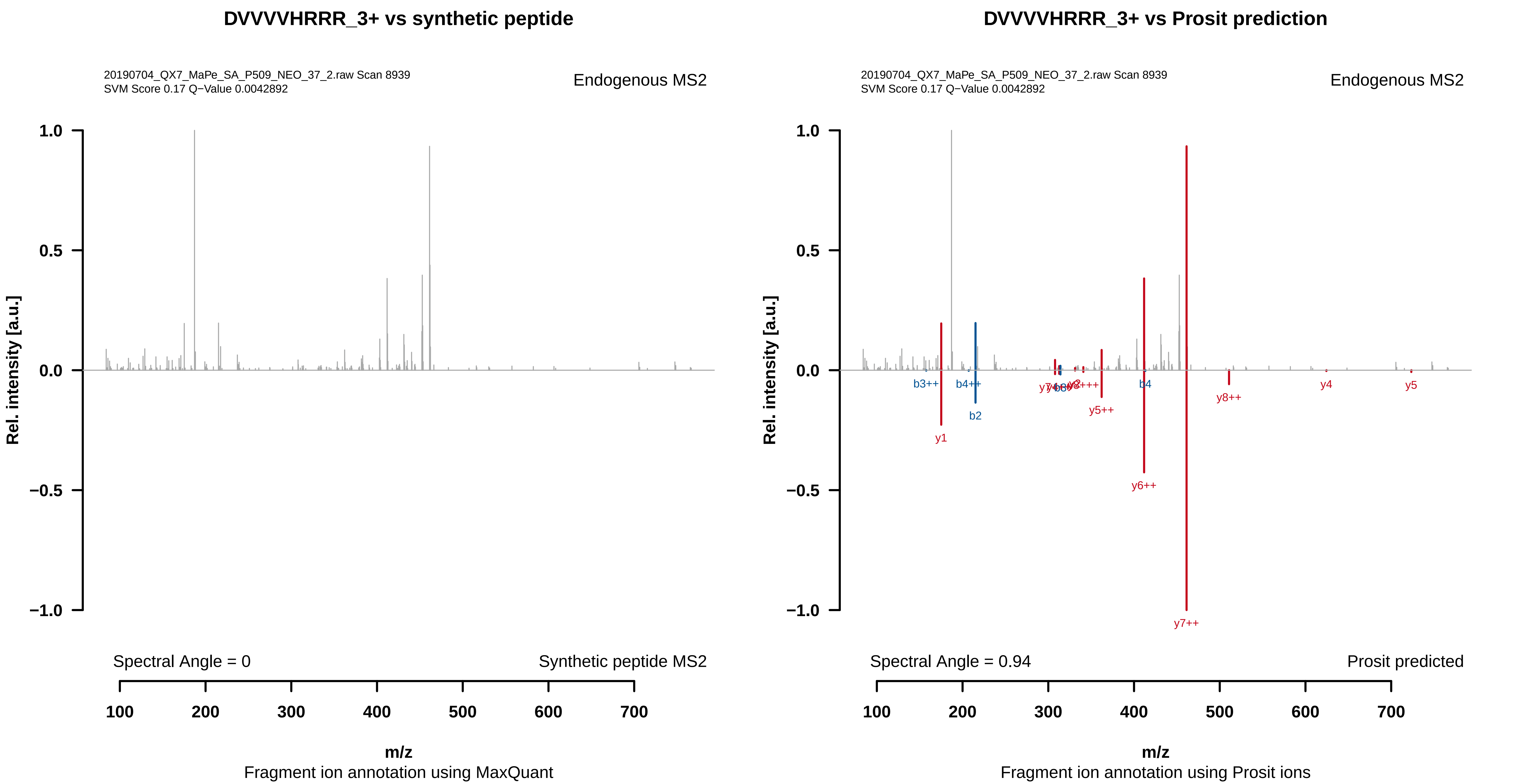
DVVVVHRRR_3+ vs synthetic peptide
20190704_QX7_MaPe_SA_P509_NEO_37_2.raw Scan 8939
SVM Score 0.17 Q−Value 0.0042892
Endogenous MS2
Rel. intensity [a.u.]
Spectral Angle = 0
Synthetic peptide MS2
m/z
Fragment ion annotation using MaxQuant
DVVVVHRRR_3+ vs Prosit prediction
20190704_QX7_MaPe_SA_P509_NEO_37_2.raw Scan 8939
SVM Score 0.17 Q−Value 0.0042892
Endogenous MS2
Rel. intensity [a.u.]
b3++
b4++
b2
y1
y5++
b4
y6++
y8++
y4
y5
y7++
Spectral Angle = 0.94
Prosit predicted
m/z
Fragment ion annotation using Prosit ions

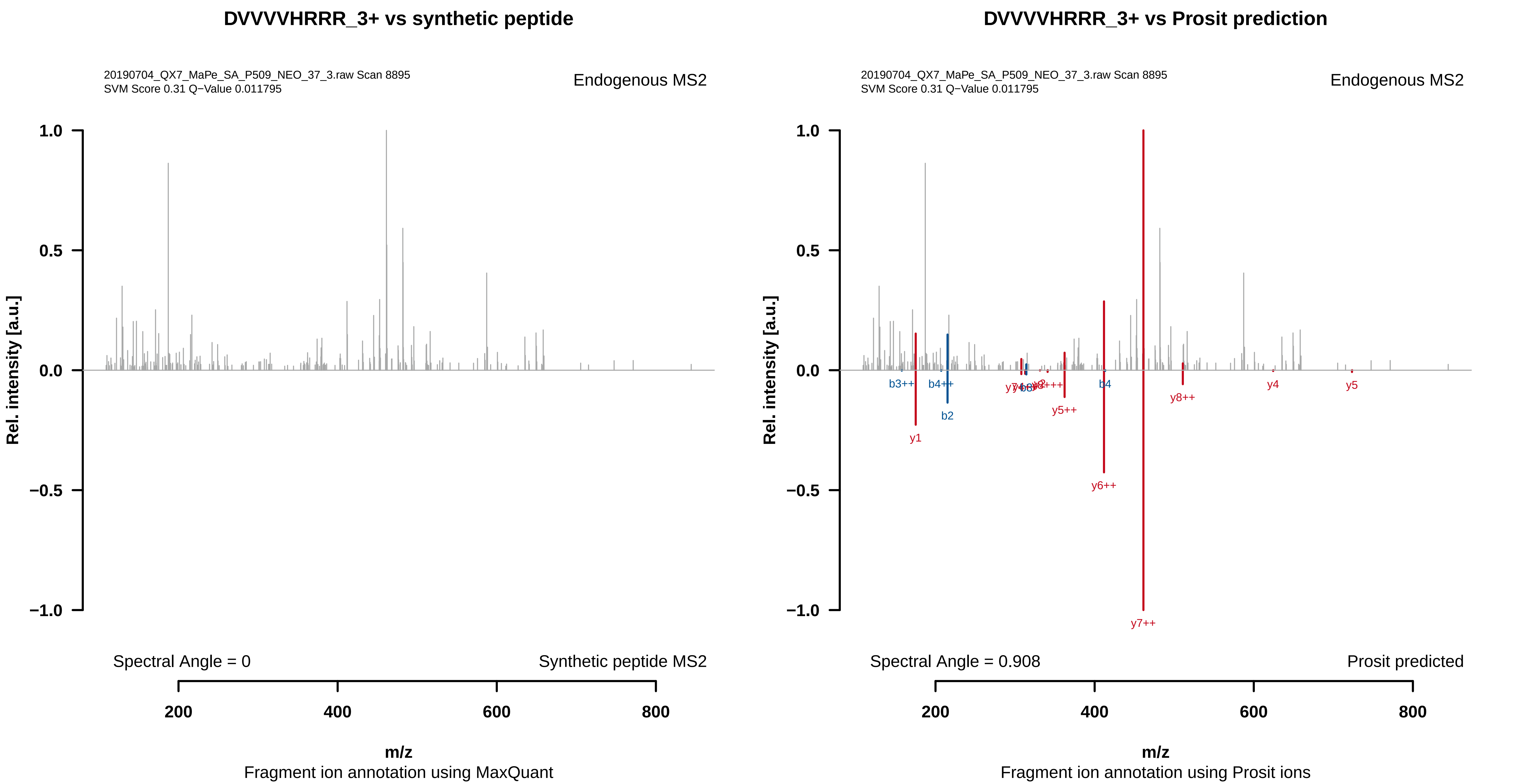
DVVVVHRRR_3+ vs synthetic peptide
20190704_QX7_MaPe_SA_P509_NEO_37_3.raw Scan 8895
SVM Score 0.31 Q−Value 0.011795
Endogenous MS2
1.0
0.5
0.0
−0.5
−1.0
Rel. intensity [a.u.]
Spectral Angle = 0
Synthetic peptide MS2
200
400
600
800
m/z
Fragment ion annotation using MaxQuant
DVVVVHRRR_3+ vs Prosit prediction
20190704_QX7_MaPe_SA_P509_NEO_37_3.raw Scan 8895
SVM Score 0.31 Q−Value 0.011795
Endogenous MS2
1.0
0.5
0.0
−0.5
−1.0
Rel. intensity [a.u.]
b3++
b4++
b2
y1
y5++
b4
y6++
y7++
y8++
y4
y5
Spectral Angle = 0.908
Prosit predicted
200
400
600
800
m/z
Fragment ion annotation using Prosit ions

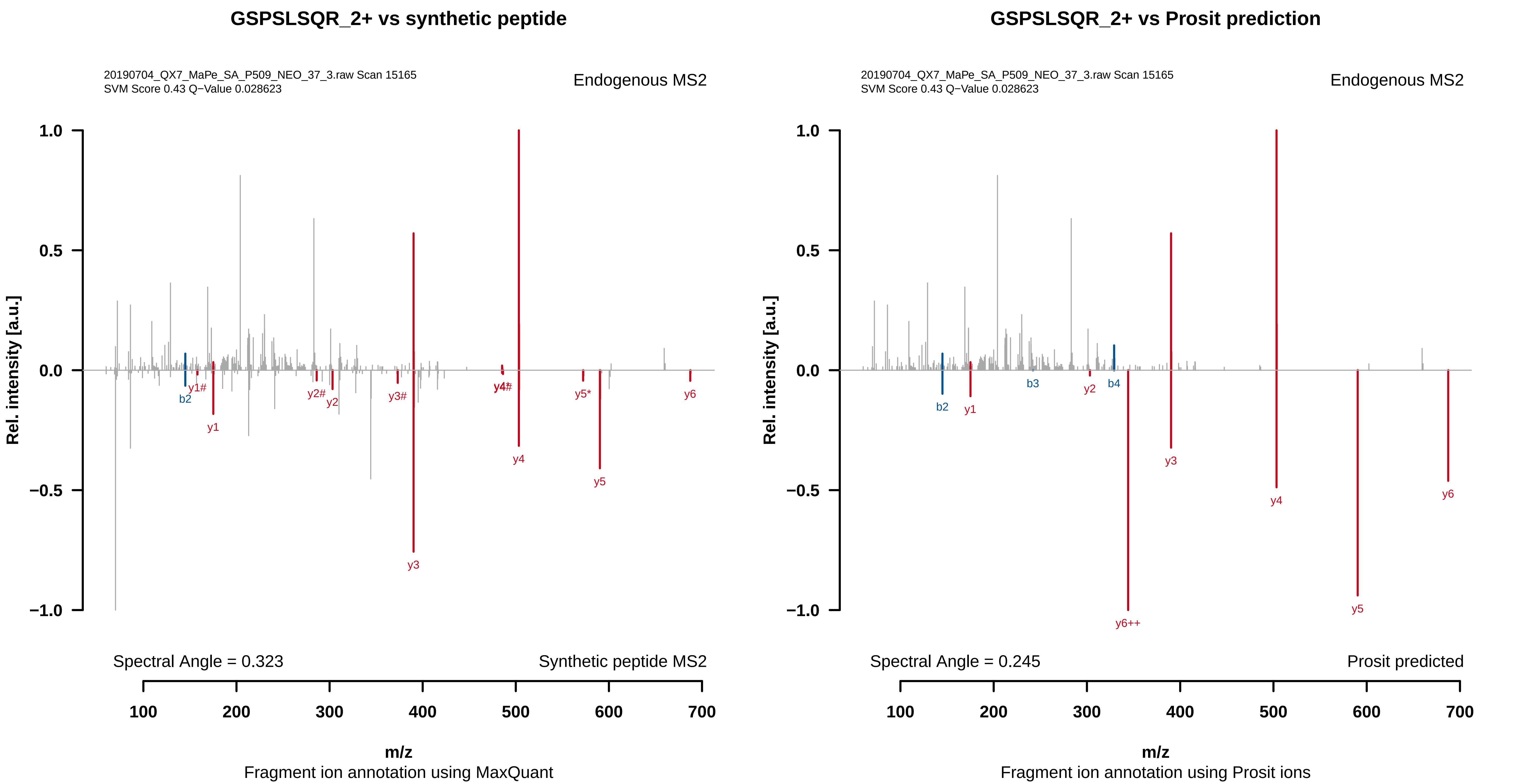
GSPSLSQR_2+ vs synthetic peptide
20190704_QX7_MaPe_SA_P509_NEO_37_3.raw Scan 15165
SVM Score 0.43 Q−Value 0.028623
Endogenous MS2
Rel. intensity [a.u.]
1.0
0.5
0.0
−0.5
−1.0
b2
y1#
y1
y2#
y2
y3#
y3
y4#
y4
y5*
y5
y6
Spectral Angle = 0.323
Synthetic peptide MS2
100
200
300
400
500
600
700
m/z
Fragment ion annotation using MaxQuant
GSPSLSQR_2+ vs Prosit prediction
20190704_QX7_MaPe_SA_P509_NEO_37_3.raw Scan 15165
SVM Score 0.43 Q−Value 0.028623
Endogenous MS2
Rel. intensity [a.u.]
b2
y1
b3
y2
b4
y6++
y3
y4
y5
y6
Spectral Angle = 0.245
Prosit predicted
m/z
Fragment ion annotation using Prosit ions

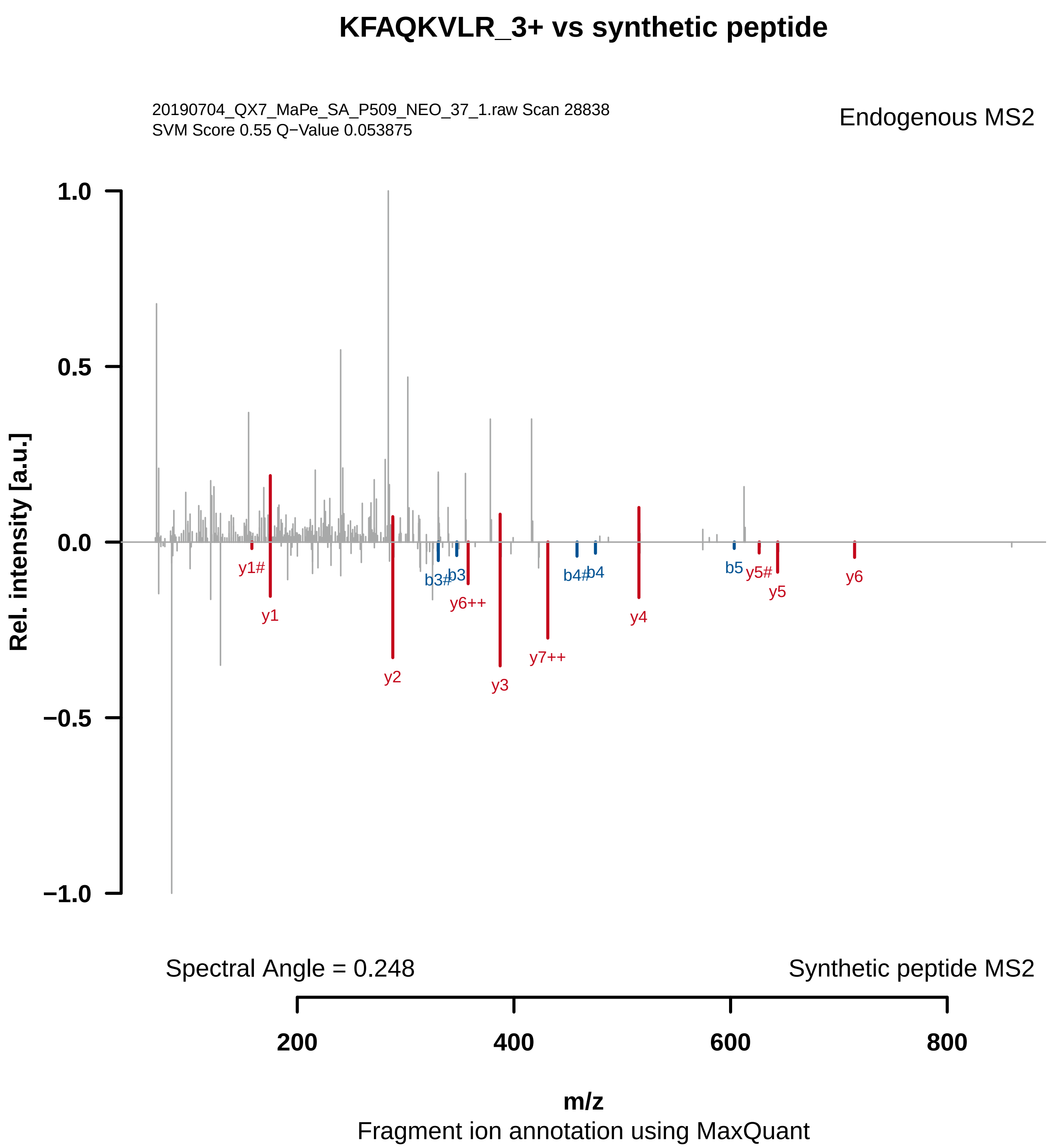

KFAQKVLR_3+ vs synthetic peptide
20190704_QX7_MaPe_SA_P509_NEO_37_1.raw Scan 28838
SVM Score 0.55 Q−Value 0.053875
Endogenous MS2
Rel. intensity [a.u.]
1.0
0.5
0.0
−0.5
−1.0
y1#
y1
y2
b3#
b3
y6++
y3
y7++
b4#
b4
y4
b5
y5#
y5
y6
Spectral Angle = 0.248
Synthetic peptide MS2
200
400
600
800
m/z
Fragment ion annotation using MaxQuant

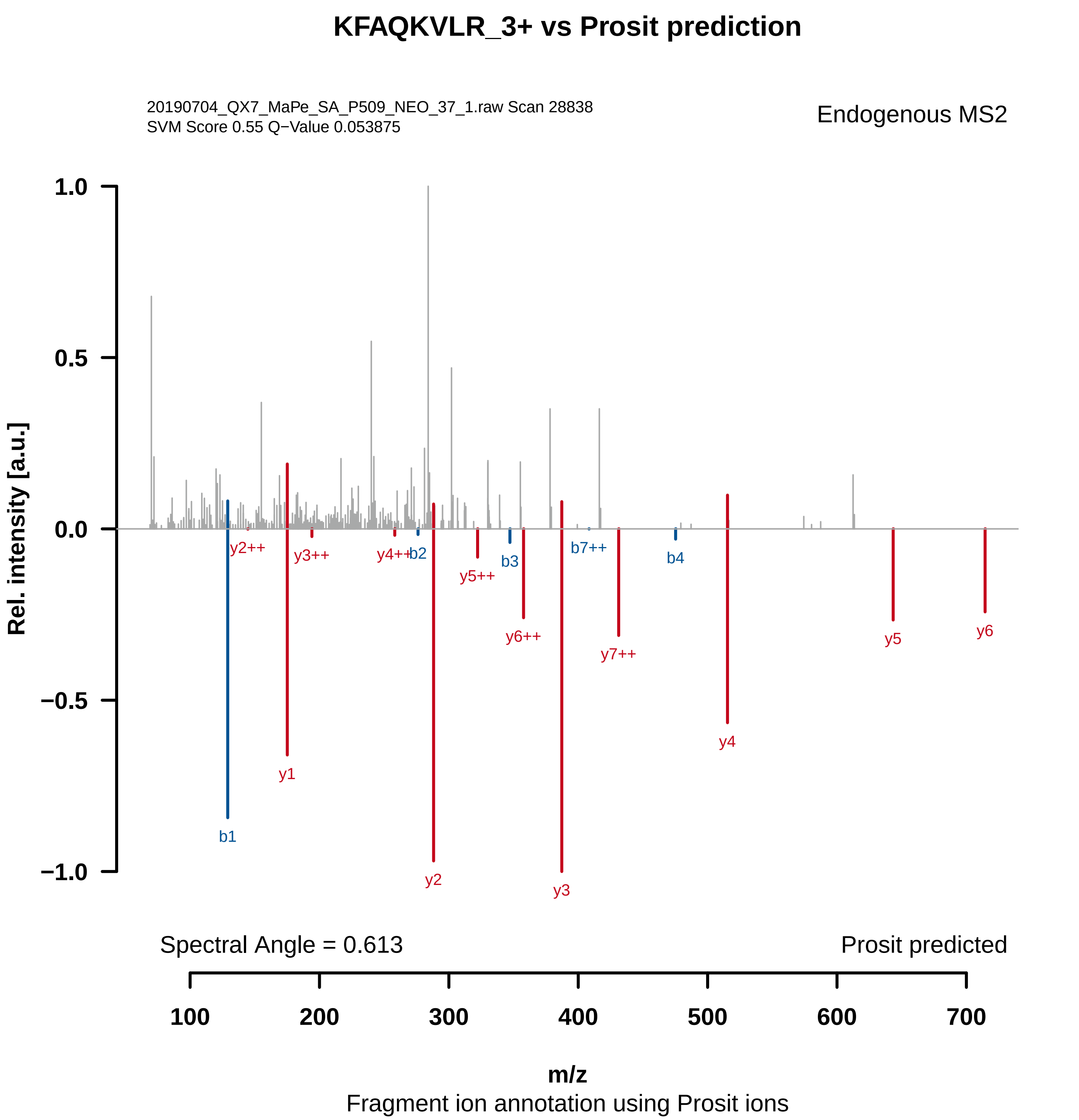

KFAQKVLR_3+ vs Prosit prediction
20190704_QX7_MaPe_SA_P509_NEO_37_1.raw Scan 28838
SVM Score 0.55 Q−Value 0.053875
Endogenous MS2
Rel. intensity [a.u.]
1.0
0.5
0.0
−0.5
−1.0
y2++
b1
y1
y3++
y4++
b2
y2
y5++
b3
y6++
y3
b7++
y7++
b4
y4
y5
y6
Spectral Angle = 0.613
Prosit predicted
100
200
300
400
500
600
700
m/z
Fragment ion annotation using Prosit ions

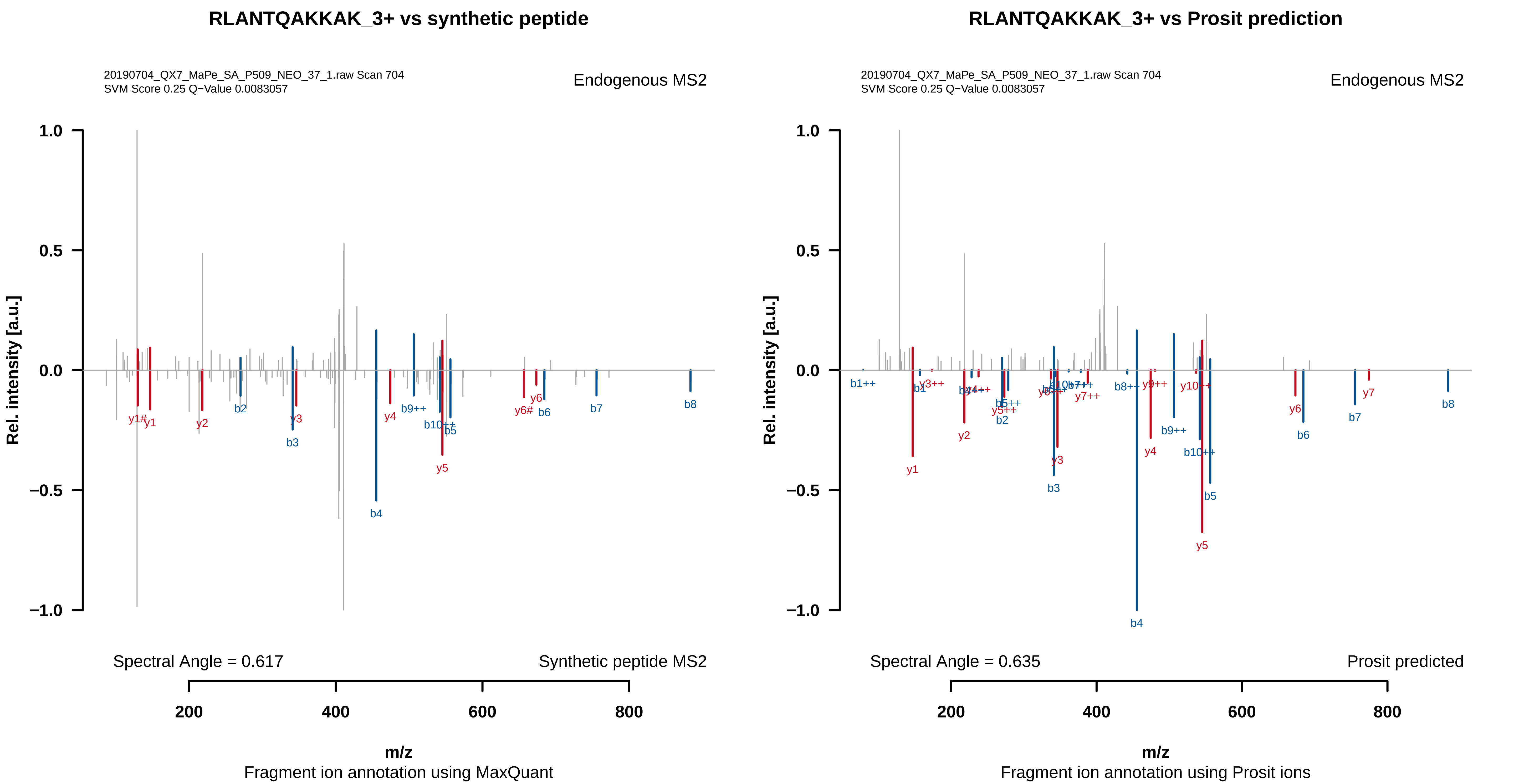

RLANTQAKKAK_3+ vs synthetic peptide
20190704_QX7_MaPe_SA_P509_NEO_37_1.raw Scan 704
SVM Score 0.25 Q−Value 0.0083057
Endogenous MS2
1.0
0.5
0.0
−0.5
−1.0
Rel. intensity [a.u.]
y1# y1 y2 b2 b3 y3 b4 y4 b9++ b10++ b5 y5 y6# y6 b6 b7 b8
Spectral Angle = 0.617
Synthetic peptide MS2
200
400
600
800
m/z
Fragment ion annotation using MaxQuant
RLANTQAKKAK_3+ vs Prosit prediction
20190704_QX7_MaPe_SA_P509_NEO_37_1.raw Scan 704
SVM Score 0.25 Q−Value 0.0083057
Endogenous MS2
1.0
0.5
0.0
−0.5
−1.0
Rel. intensity [a.u.]
b1++ b1 y3++ y1 y2 b2 y5++ b3 y3 b8++ y9++ y4 b4 b9++ y10++ b10++ b5 y5 y7++ y6 b6 b7 y7 b8
Spectral Angle = 0.635
Prosit predicted
200
400
600
800
m/z
Fragment ion annotation using Prosit ions

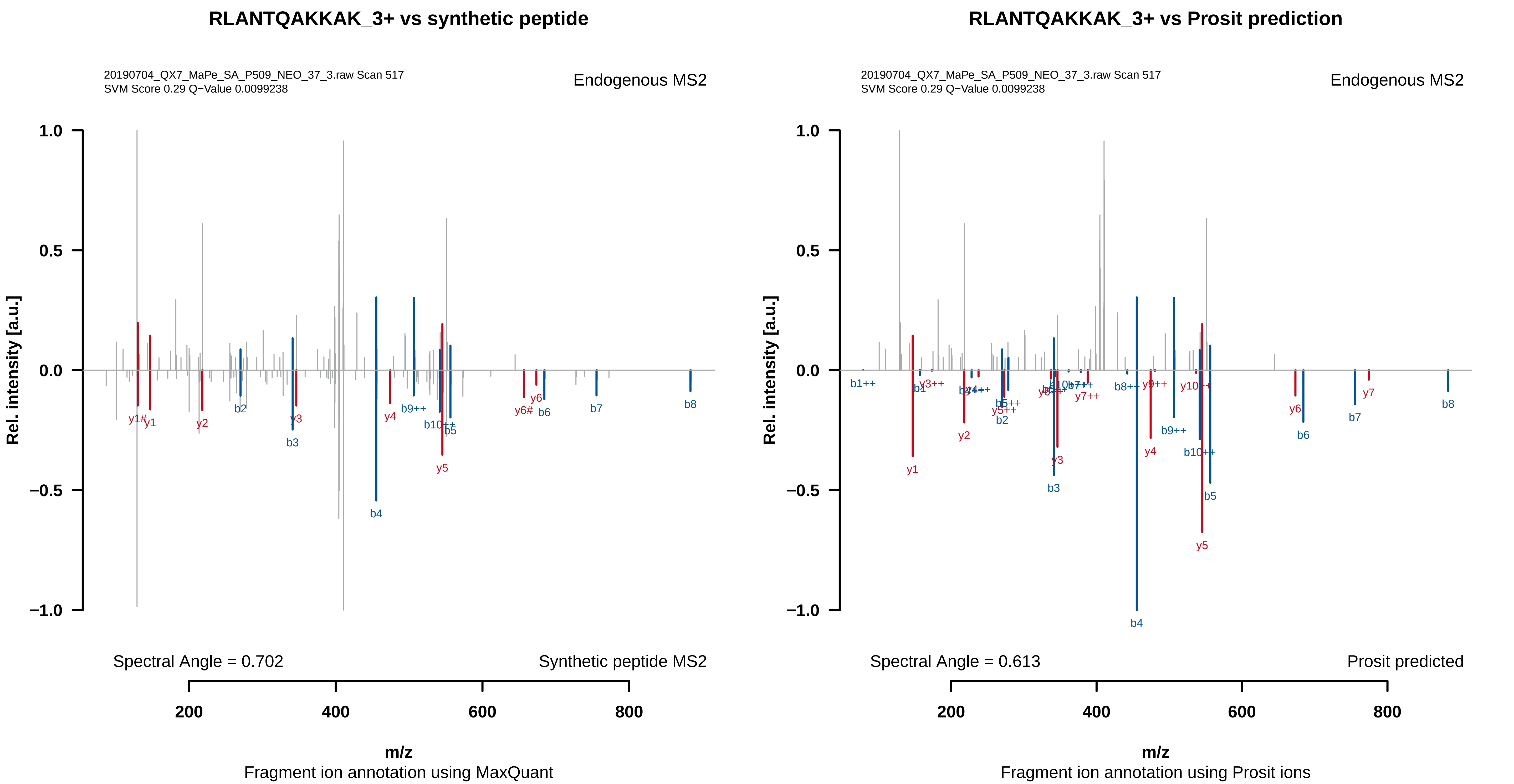

RLANTQAKKAK_3+ vs synthetic peptide
20190704_QX7_MaPe_SA_P509_NEO_37_3.raw Scan 517
SVM Score 0.29 Q−Value 0.0099238
Endogenous MS2
Rel. intensity [a.u.]
1.0
0.5
0.0
−0.5
−1.0
y1# y1 y2 b2 y3 b3 b4 y4 b9++ b10++ b5 y5 y6 y6# b6 b7 b8
Spectral Angle = 0.702
Synthetic peptide MS2
200
400
600
800
m/z
Fragment ion annotation using MaxQuant
RLANTQAKKAK_3+ vs Prosit prediction
20190704_QX7_MaPe_SA_P509_NEO_37_3.raw Scan 517
SVM Score 0.29 Q−Value 0.0099238
Endogenous MS2
Rel. intensity [a.u.]
1.0
0.5
0.0
−0.5
−1.0
b1++ b1 y3++ y1 y2 b4++ y4++ b5++ y5++ b2 y3 b3 y7++ b8++ y9++ y4 b9++ y10++ b10++ b5 y5 b4 y6 b6 b7 y7 b8
Spectral Angle = 0.613
Prosit predicted
200
400
600
800
m/z
Fragment ion annotation using Prosit ions

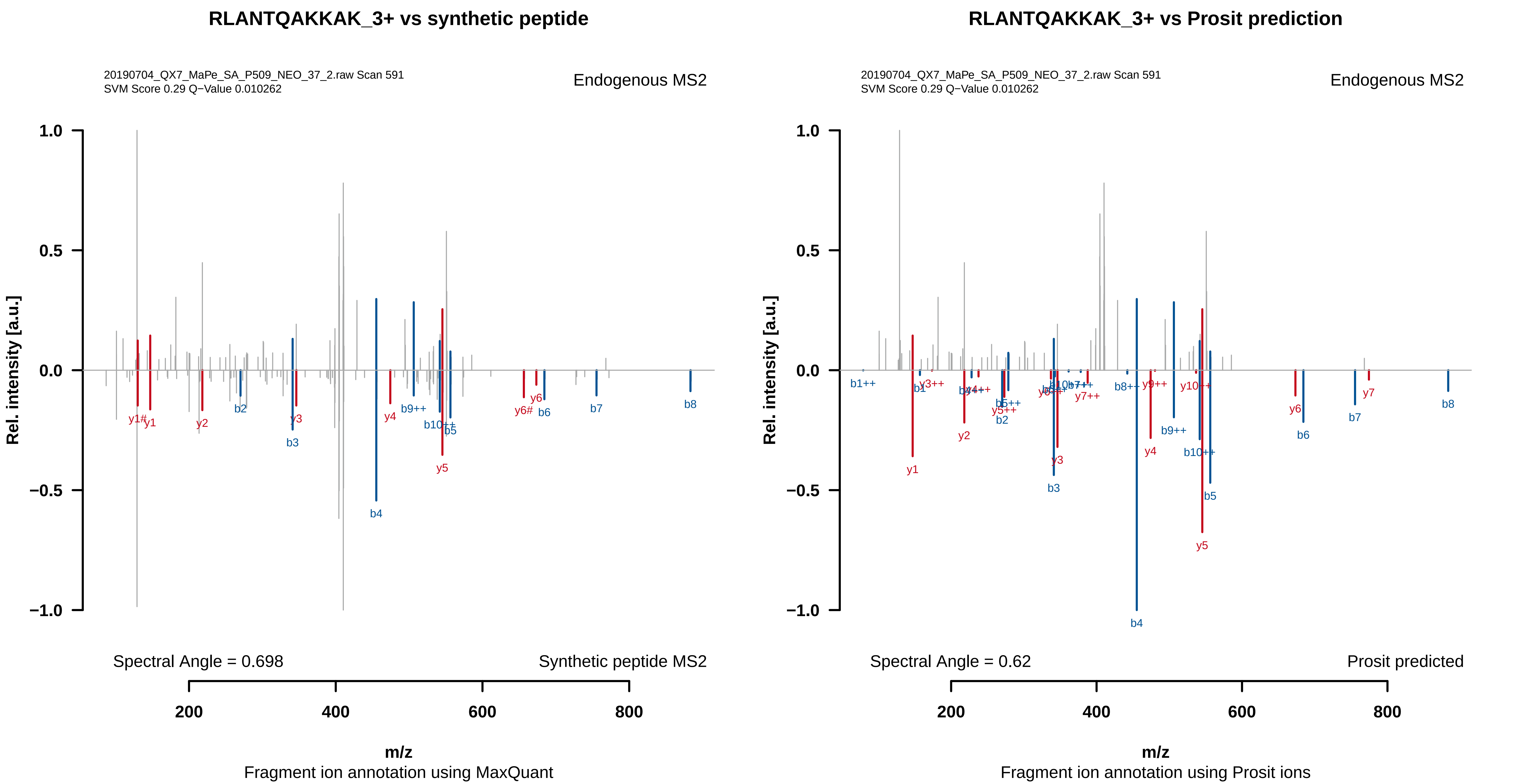
RLANTQAKKAK_3+ vs synthetic peptide
20190704_QX7_MaPe_SA_P509_NEO_37_2.raw Scan 591
SVM Score 0.29 Q−Value 0.010262
Endogenous MS2
Rel. intensity [a.u.]
Spectral Angle = 0.698
Synthetic peptide MS2
m/z
Fragment ion annotation using MaxQuant
RLANTQAKKAK_3+ vs Prosit prediction
20190704_QX7_MaPe_SA_P509_NEO_37_2.raw Scan 591
SVM Score 0.29 Q−Value 0.010262
Endogenous MS2
Rel. intensity [a.u.]
Spectral Angle = 0.62
Prosit predicted
m/z
Fragment ion annotation using Prosit ions

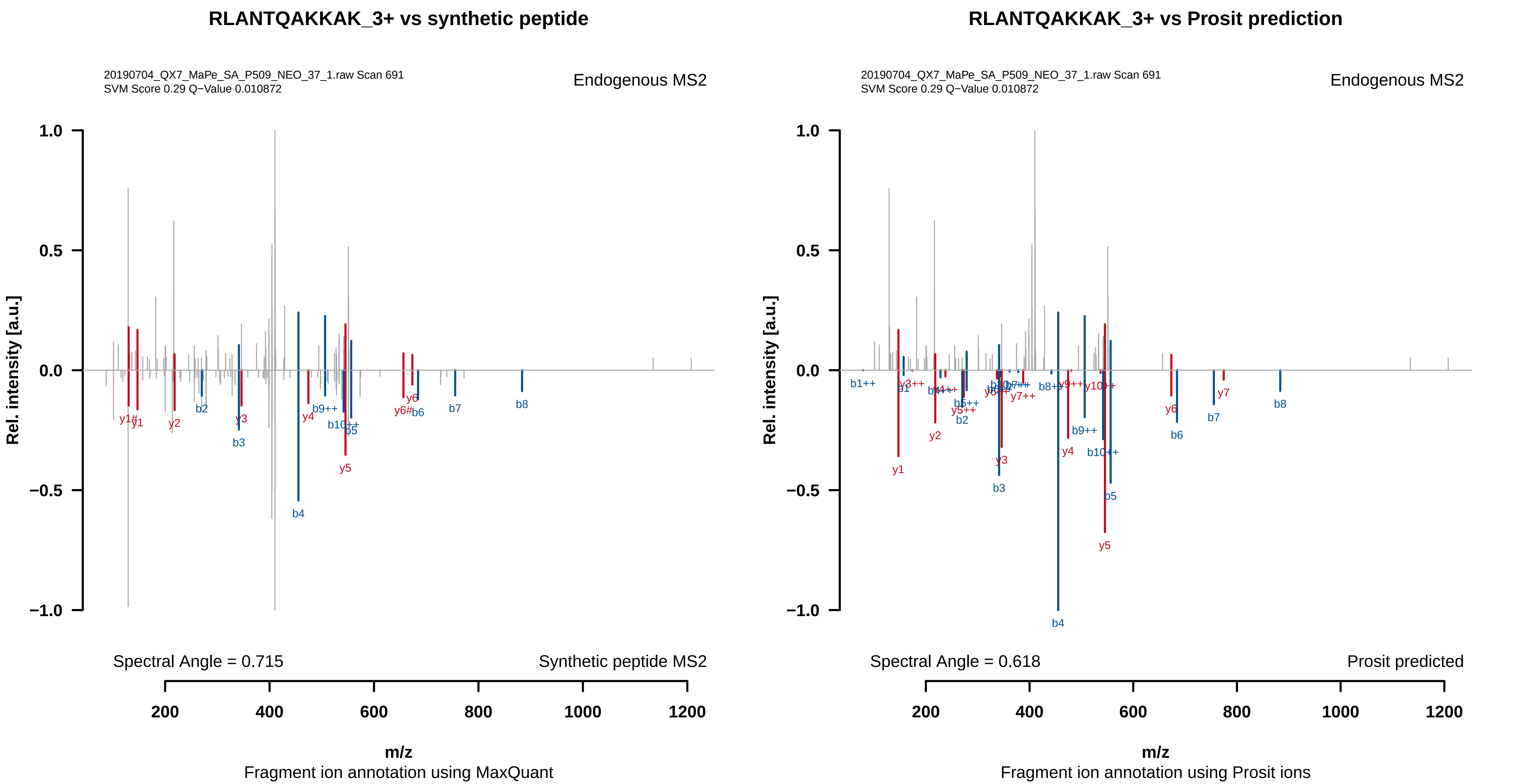
RLANTQAKKAK_3+ vs synthetic peptide
20190704_QX7_MaPe_SA_P509_NEO_37_1.raw Scan 691
SVM Score 0.29 Q−Value 0.010872
Endogenous MS2
Rel. intensity [a.u.]
1.0
0.5
0.0
−0.5
−1.0
y1#
y1
y2
b2
y3
b3
y4
b4
b9++
b10++
b5
y5
y6
y6#
b6
b7
b8
Spectral Angle = 0.715
Synthetic peptide MS2
200
400
600
800
1000
1200
m/z
Fragment ion annotation using MaxQuant
RLANTQAKKAK_3+ vs Prosit prediction
20190704_QX7_MaPe_SA_P509_NEO_37_1.raw Scan 691
SVM Score 0.29 Q−Value 0.010872
Endogenous MS2
Rel. intensity [a.u.]
1.0
0.5
0.0
−0.5
−1.0
b1++
y3++
y1
y2
b5++
y5++
b2
y7++
b8++
y9++
y10++
y3
b3
b4
b9++
y4
b10++
b5
y5
y6
b6
y7
b7
b8
Spectral Angle = 0.618
Prosit predicted
200
400
600
800
1000
1200
m/z
Fragment ion annotation using Prosit ions

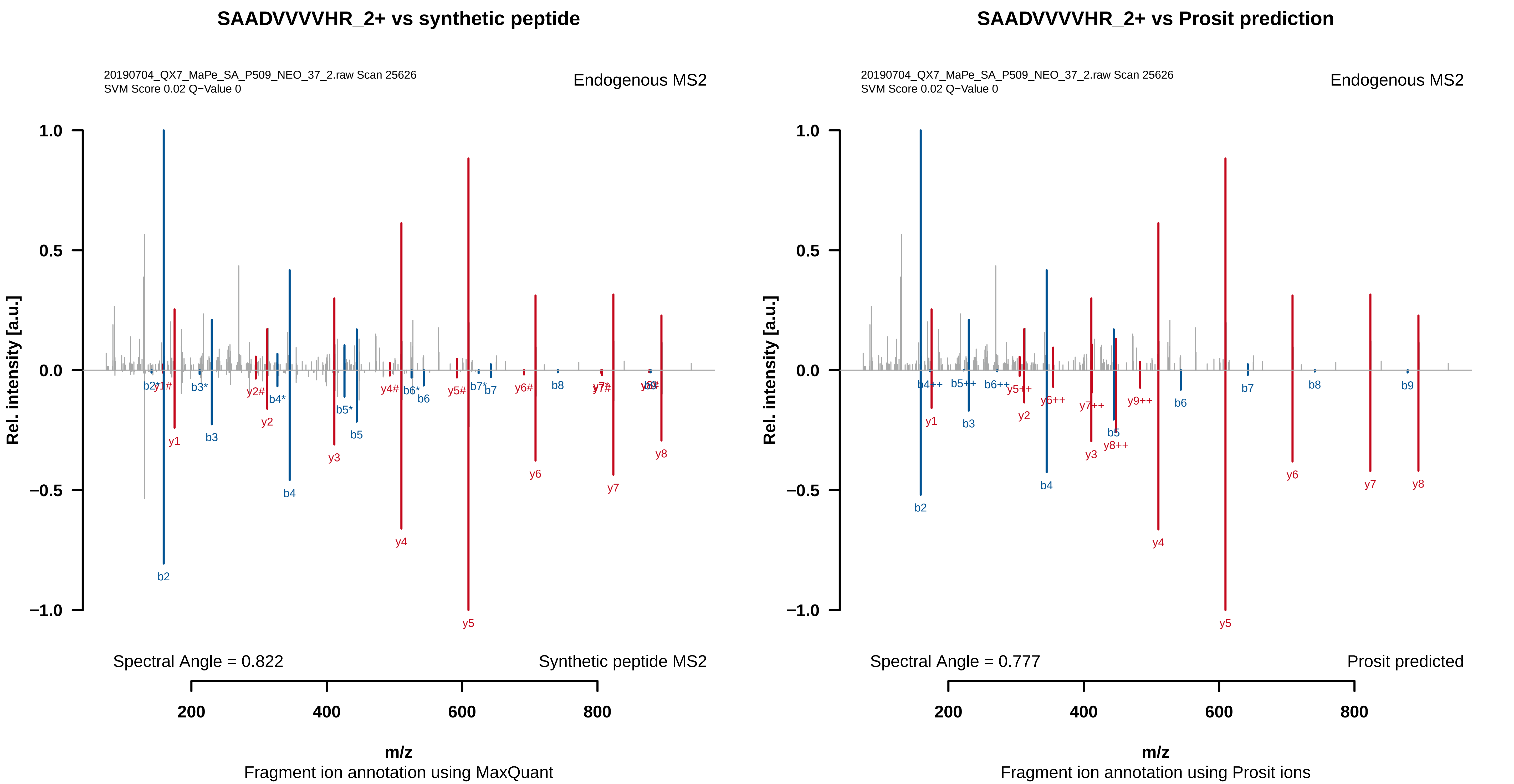

SAADVVVVHR_2+ vs synthetic peptide
20190704_QX7_MaPe_SA_P509_NEO_37_2.raw Scan 25626
SVM Score 0.02 Q−Value 0
Endogenous MS2
Rel. intensity [a.u.]
1.0
0.5
0.0
−0.5
−1.0
b2
y1
b3
y2
b4
y3
b5
y4
y5
y6
y7
y8
b6
b7
b8
Spectral Angle = 0.822
Synthetic peptide MS2
200
400
600
800
m/z
Fragment ion annotation using MaxQuant
SAADVVVVHR_2+ vs Prosit prediction
20190704_QX7_MaPe_SA_P509_NEO_37_2.raw Scan 25626
SVM Score 0.02 Q−Value 0
Endogenous MS2
Rel. intensity [a.u.]
b4++
b5++
b6++
y5++
y6++
y7++
y8++
y9++
b9
Spectral Angle = 0.777
Prosit predicted
m/z
Fragment ion annotation using Prosit ions

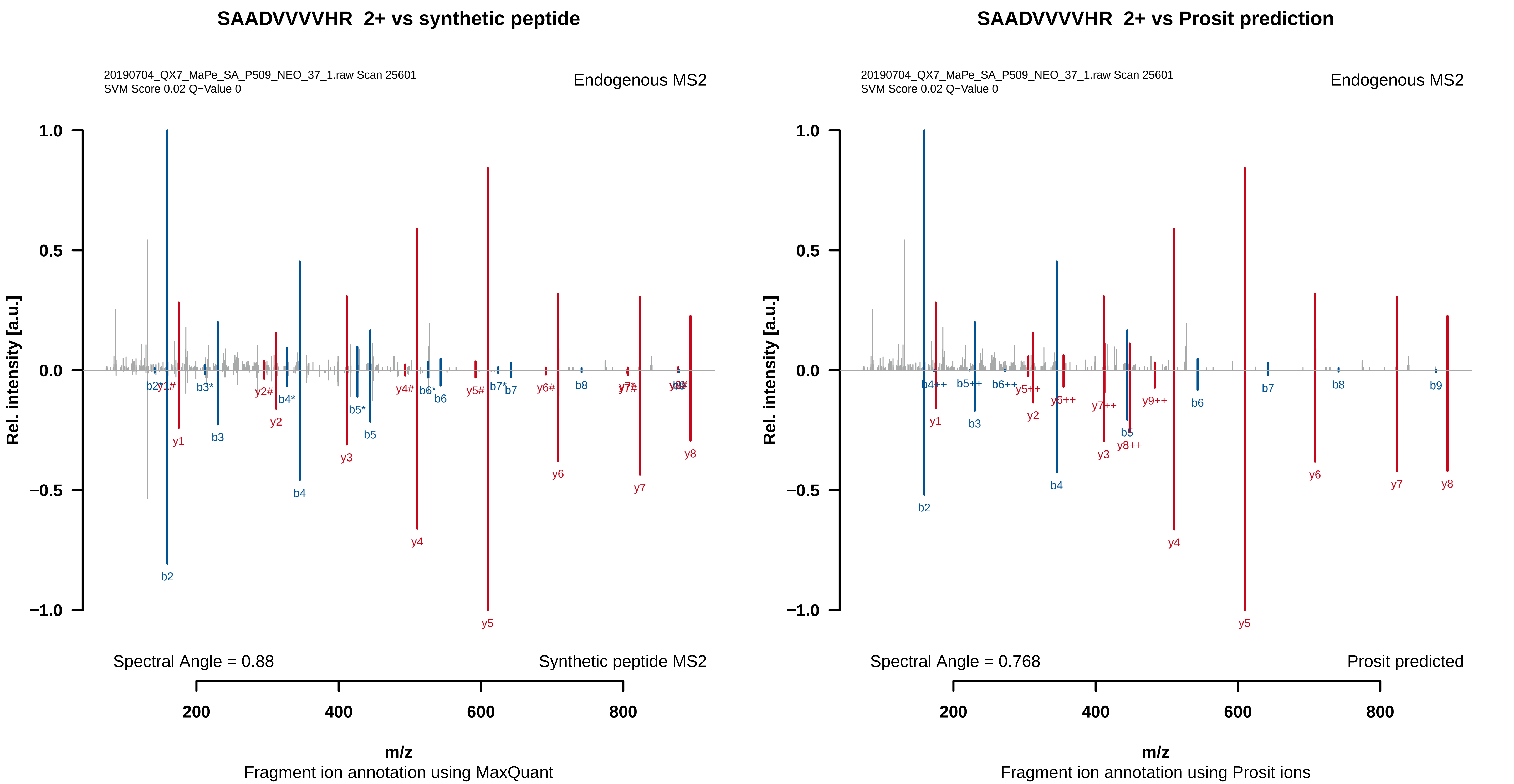

SAADVVVVHR_2+ vs synthetic peptide
20190704_QX7_MaPe_SA_P509_NEO_37_1.raw Scan 25601
SVM Score 0.02 Q−Value 0
Endogenous MS2
Rel. intensity [a.u.]
1.0
0.5
0.0
−0.5
−1.0
b2* y1# b3* y2# b4* b5* y4# b6* b6 y5# b7* b7 y6# b8 y7* y7# y8# b9#
y1 b3 y2 b4 b5 y3 y4 y5 y6 y7 y8 b2
Spectral Angle = 0.88
Synthetic peptide MS2
200
400
600
800
m/z
Fragment ion annotation using MaxQuant
SAADVVVVHR_2+ vs Prosit prediction
20190704_QX7_MaPe_SA_P509_NEO_37_1.raw Scan 25601
SVM Score 0.02 Q−Value 0
Endogenous MS2
Rel. intensity [a.u.]
1.0
0.5
0.0
−0.5
−1.0
b4++ b5++ b6++ y5++ y6++ y7++ y9++ b6 b7 b8 b9
y1 b3 y2 b4 y3 b5 y8++ y4 y5 y6 y7 y8 b2
Spectral Angle = 0.768
Prosit predicted
200
400
600
800
m/z
Fragment ion annotation using Prosit ions

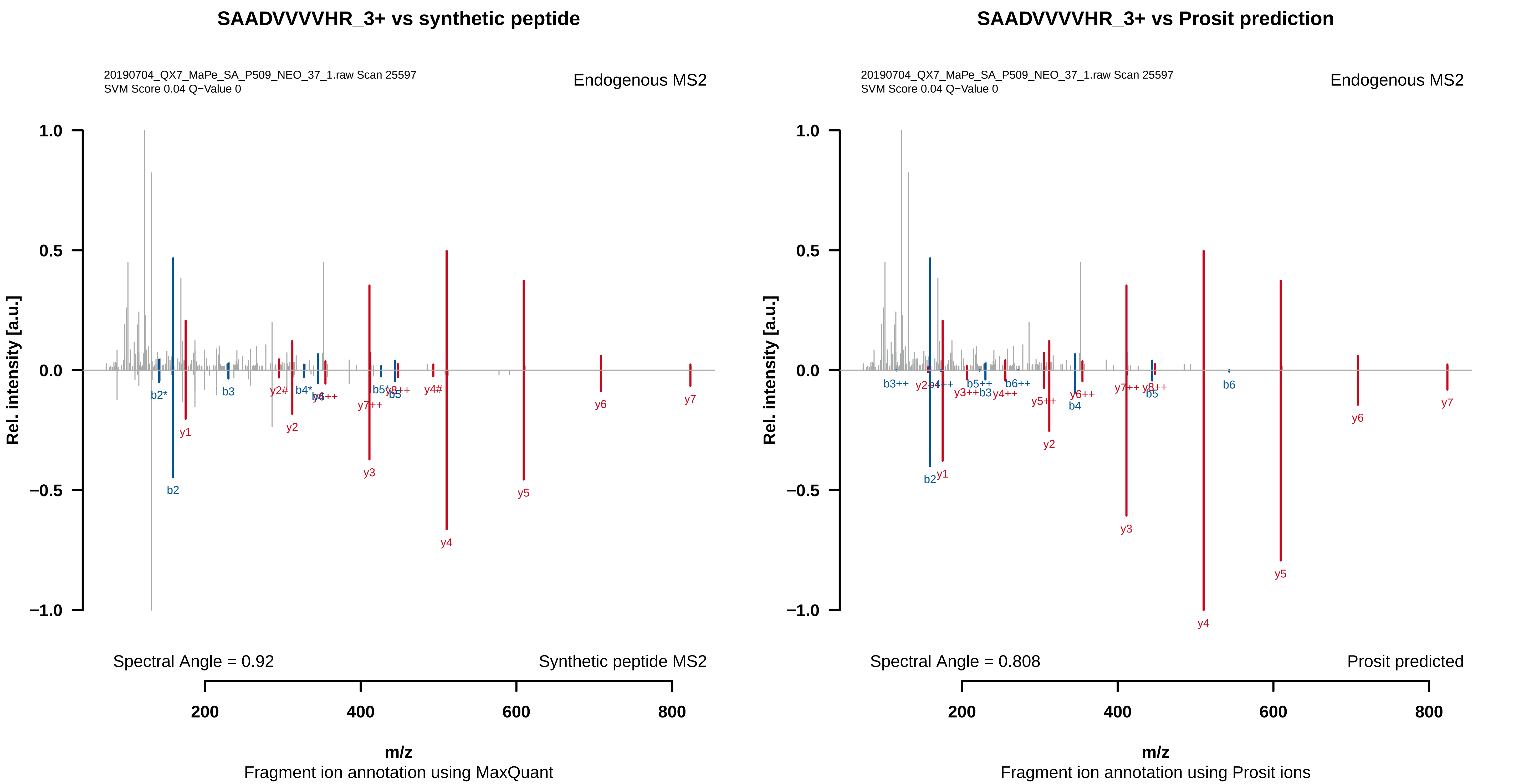
SAADVVVVHR_3+ vs synthetic peptide
20190704_QX7_MaPe_SA_P509_NEO_37_1.raw Scan 25597
SVM Score 0.04 Q−Value 0
Endogenous MS2
Rel. intensity [a.u.]
1.0
0.5
0.0
−0.5
−1.0
b2*
b2
b3
y1
y2#
y2
b4*
y3
y7++
y4#
y4
y5
y6
y7
Spectral Angle = 0.92
Synthetic peptide MS2
200
400
600
800
m/z
Fragment ion annotation using MaxQuant
SAADVVVVHR_3+ vs Prosit prediction
20190704_QX7_MaPe_SA_P509_NEO_37_1.raw Scan 25597
SVM Score 0.04 Q−Value 0
Endogenous MS2
Rel. intensity [a.u.]
1.0
0.5
0.0
−0.5
−1.0
b3++
b5++
b6++
y3++
y4++
y5++
y6++
y7++
b2
y1
y2
b4
y3
b5
b6
y4
y5
y6
y7
Spectral Angle = 0.808
Prosit predicted
200
400
600
800
m/z
Fragment ion annotation using Prosit ions

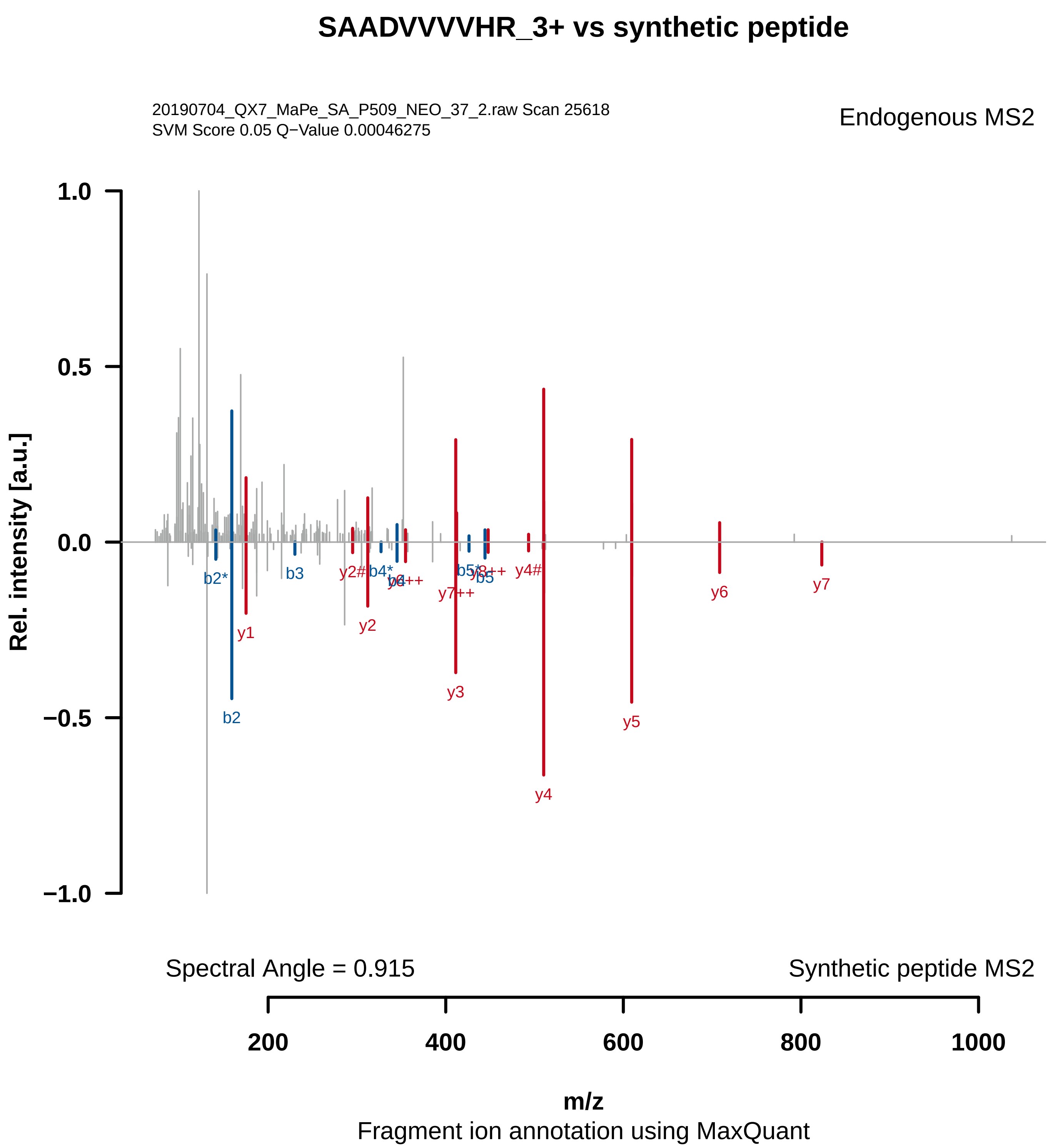
SAADVVVVHR_3+ vs synthetic peptide
20190704_QX7_MaPe_SA_P509_NEO_37_2.raw Scan 25618
SVM Score 0.05 Q−Value 0.00046275
Endogenous MS2
Rel. intensity [a.u.]
1.0
0.5
0.0
−0.5
−1.0
Spectral Angle = 0.915
Synthetic peptide MS2
200
400
600
800
1000
m/z
Fragment ion annotation using MaxQuant

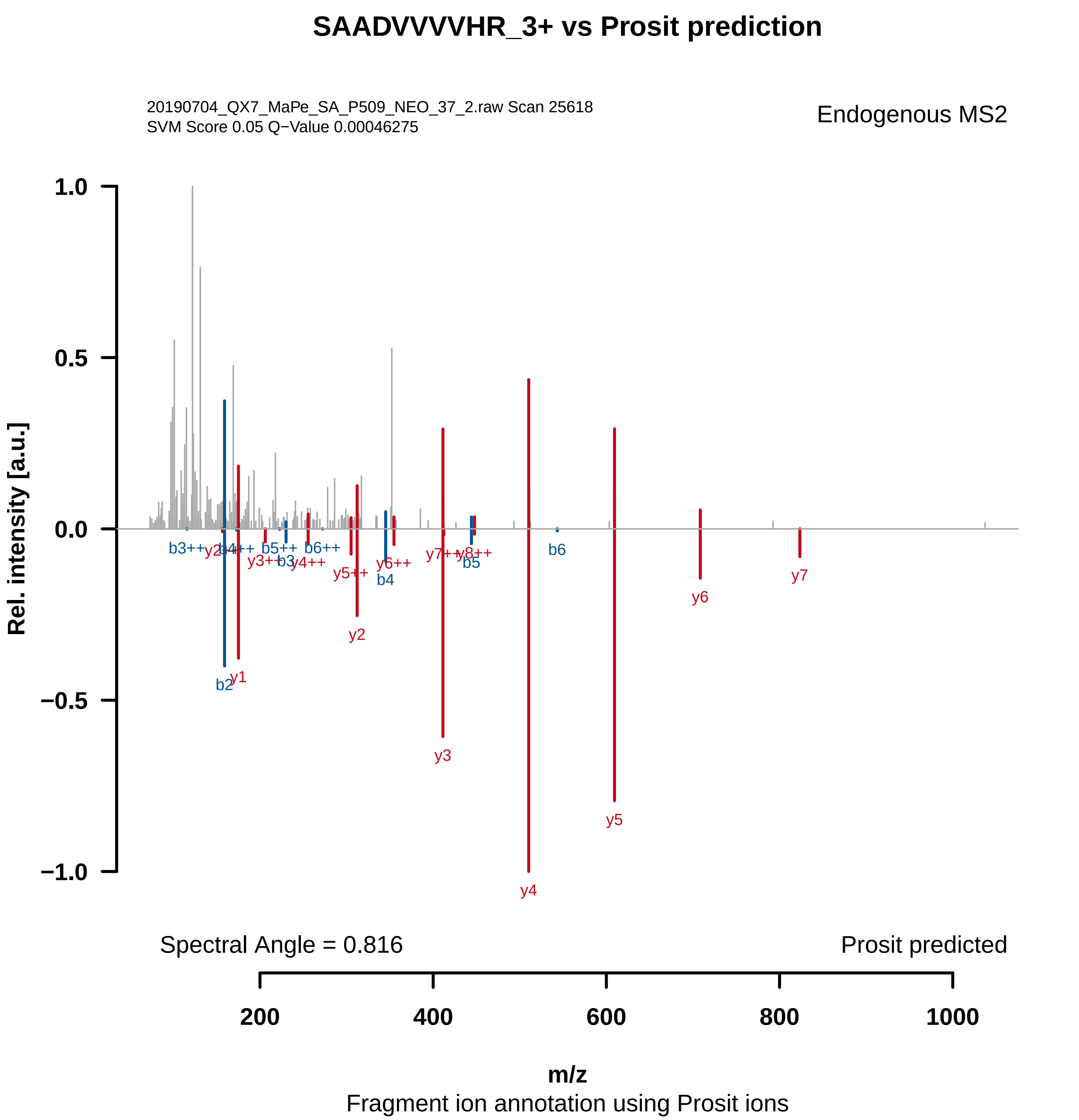
SAADVVVVHR_3+ vs Prosit prediction
20190704_QX7_MaPe_SA_P509_NEO_37_2.raw Scan 25618
SVM Score 0.05 Q−Value 0.00046275
Endogenous MS2
Rel. intensity [a.u.]
1.0
0.5
0.0
−0.5
−1.0
Spectral Angle = 0.816
Prosit predicted
200
400
600
800
1000
m/z
Fragment ion annotation using Prosit ions

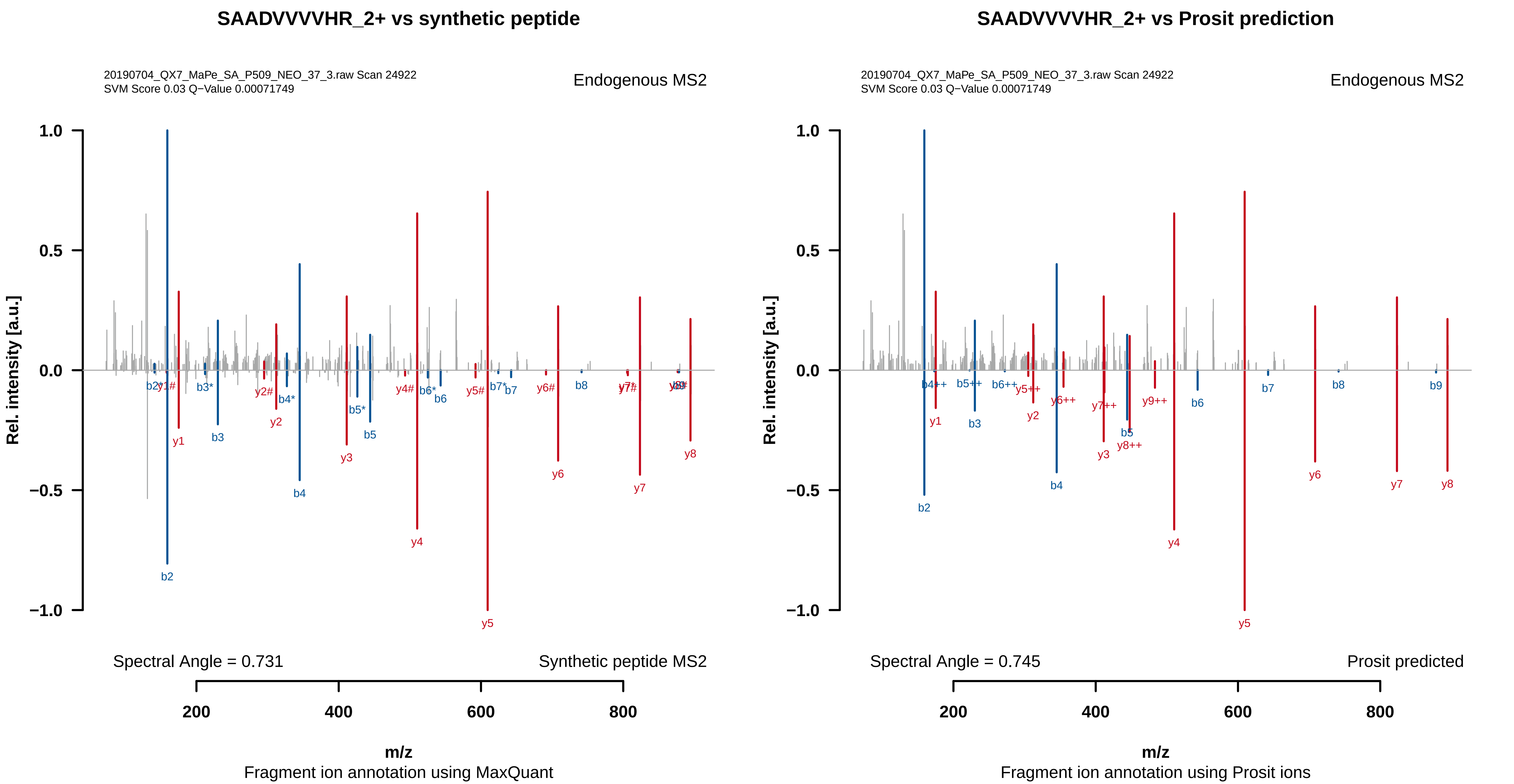

SAADVVVVHR_2+ vs synthetic peptide
20190704_QX7_MaPe_SA_P509_NEO_37_3.raw Scan 24922
SVM Score 0.03 Q−Value 0.00071749
Endogenous MS2
Rel. intensity [a.u.]
Spectral Angle = 0.731
Synthetic peptide MS2
m/z
Fragment ion annotation using MaxQuant
SAADVVVVHR_2+ vs Prosit prediction
20190704_QX7_MaPe_SA_P509_NEO_37_3.raw Scan 24922
SVM Score 0.03 Q−Value 0.00071749
Endogenous MS2
Rel. intensity [a.u.]
Spectral Angle = 0.745
Prosit predicted
m/z
Fragment ion annotation using Prosit ions

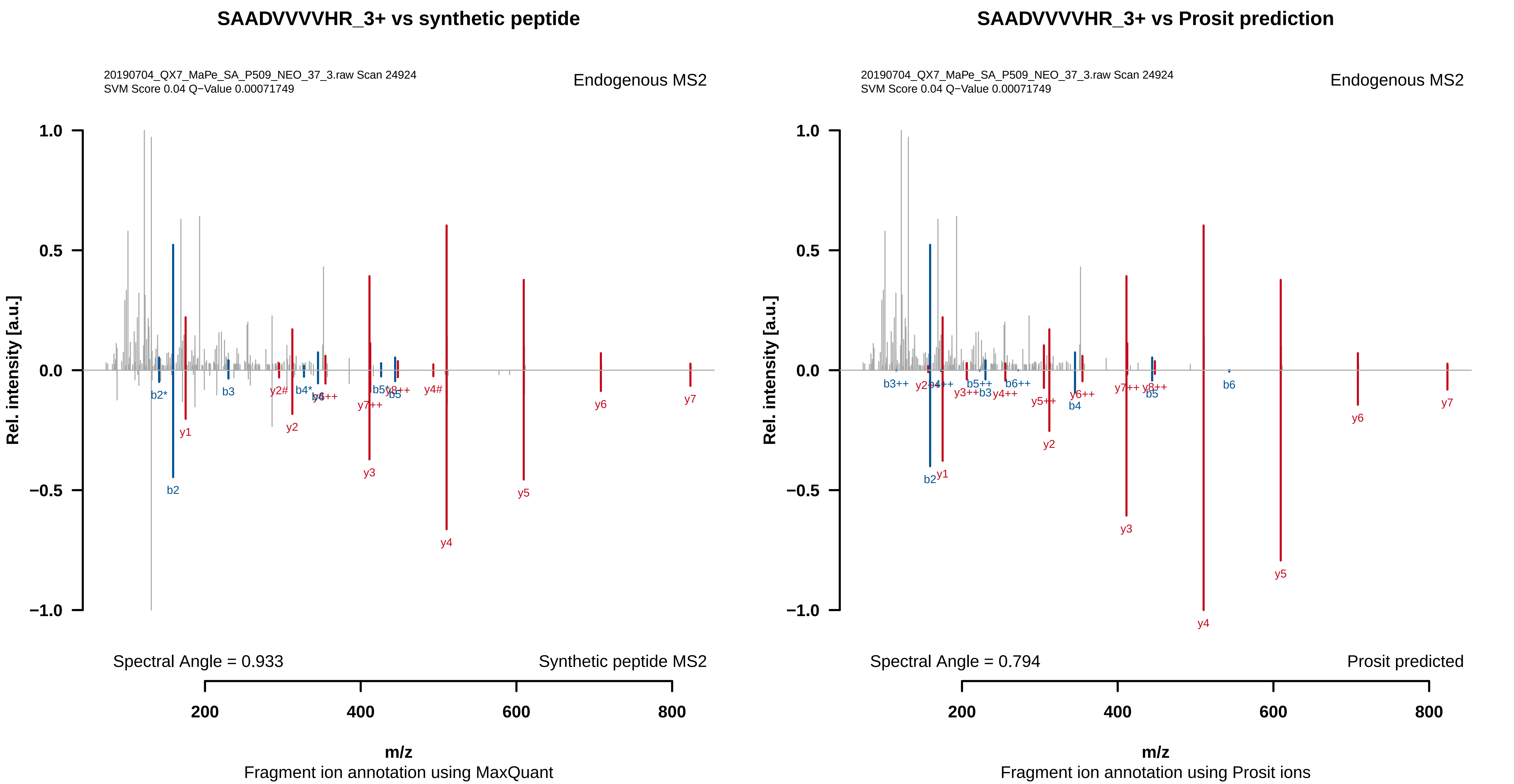
SAADVVVVHR_3+ vs synthetic peptide
20190704_QX7_MaPe_SA_P509_NEO_37_3.raw Scan 24924
SVM Score 0.04 Q−Value 0.00071749
Endogenous MS2
Rel. intensity [a.u.]
1.0
0.5
0.0
−0.5
−1.0
b2*
b2
y1
b3
y2#
y2
b4*
y3
y7++
b5*
b5
y8++
y4#
y4
y5
y6
y7
Spectral Angle = 0.933
Synthetic peptide MS2
200
400
600
800
m/z
Fragment ion annotation using MaxQuant
SAADVVVVHR_3+ vs Prosit prediction
20190704_QX7_MaPe_SA_P509_NEO_37_3.raw Scan 24924
SVM Score 0.04 Q−Value 0.00071749
Endogenous MS2
Rel. intensity [a.u.]
1.0
0.5
0.0
−0.5
−1.0
b3++
b2
y1
b5++
b3
y3++
y4++
b6++
y5++
y2
y6++
b4
y7++
y8++
b5
y3
b6
y4
y5
y6
y7
Spectral Angle = 0.794
Prosit predicted
200
400
600
800
m/z
Fragment ion annotation using Prosit ions

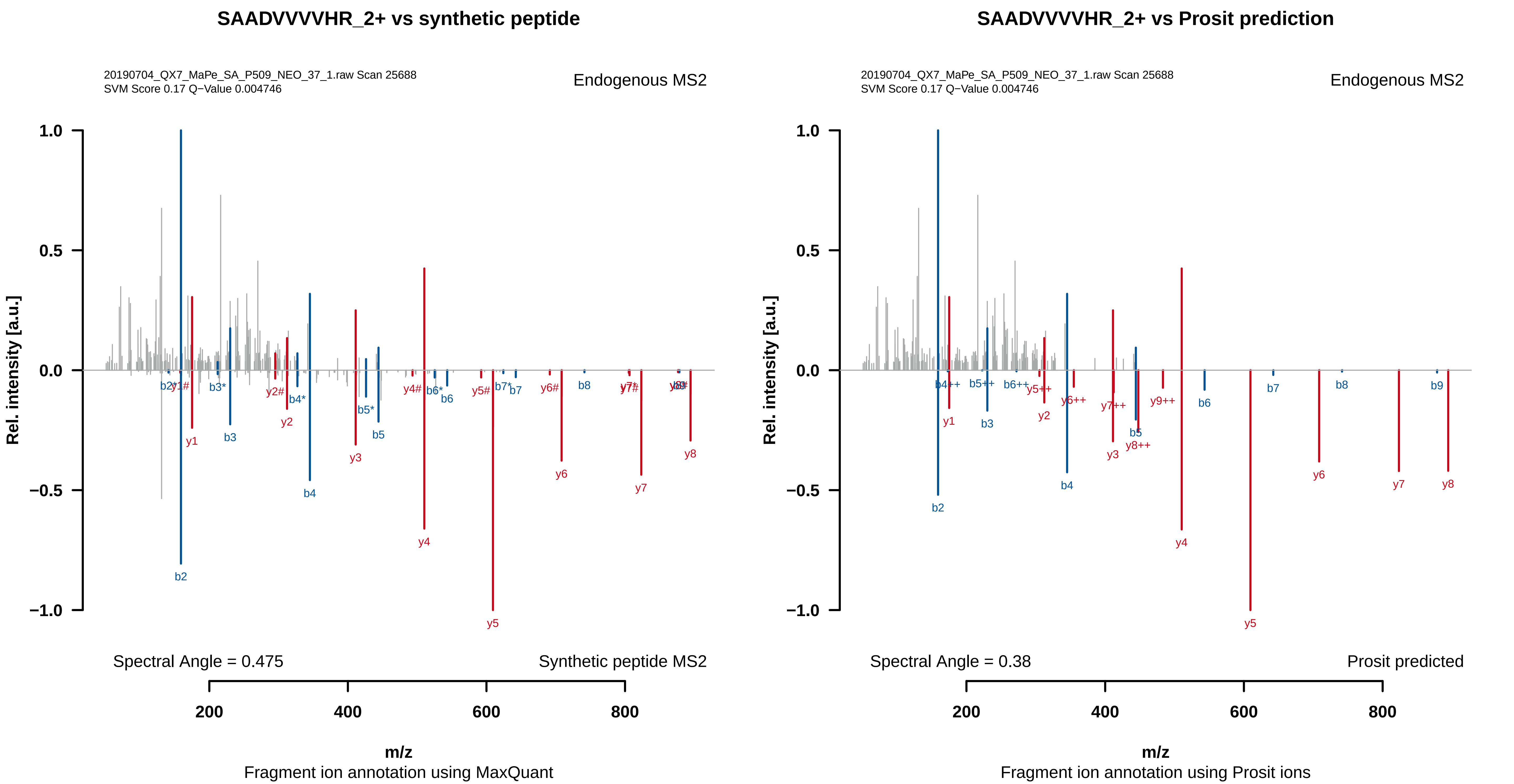

SAADVVVVHR_2+ vs synthetic peptide
20190704_QX7_MaPe_SA_P509_NEO_37_1.raw Scan 25688
SVM Score 0.17 Q−Value 0.004746
Endogenous MS2
Rel. intensity [a.u.]
1.0
0.5
0.0
−0.5
−1.0
Spectral Angle = 0.475
Synthetic peptide MS2
200
400
600
800
m/z
Fragment ion annotation using MaxQuant
SAADVVVVHR_2+ vs Prosit prediction
20190704_QX7_MaPe_SA_P509_NEO_37_1.raw Scan 25688
SVM Score 0.17 Q−Value 0.004746
Endogenous MS2
Rel. intensity [a.u.]
1.0
0.5
0.0
−0.5
−1.0
Spectral Angle = 0.38
Prosit predicted
200
400
600
800
m/z
Fragment ion annotation using Prosit ions

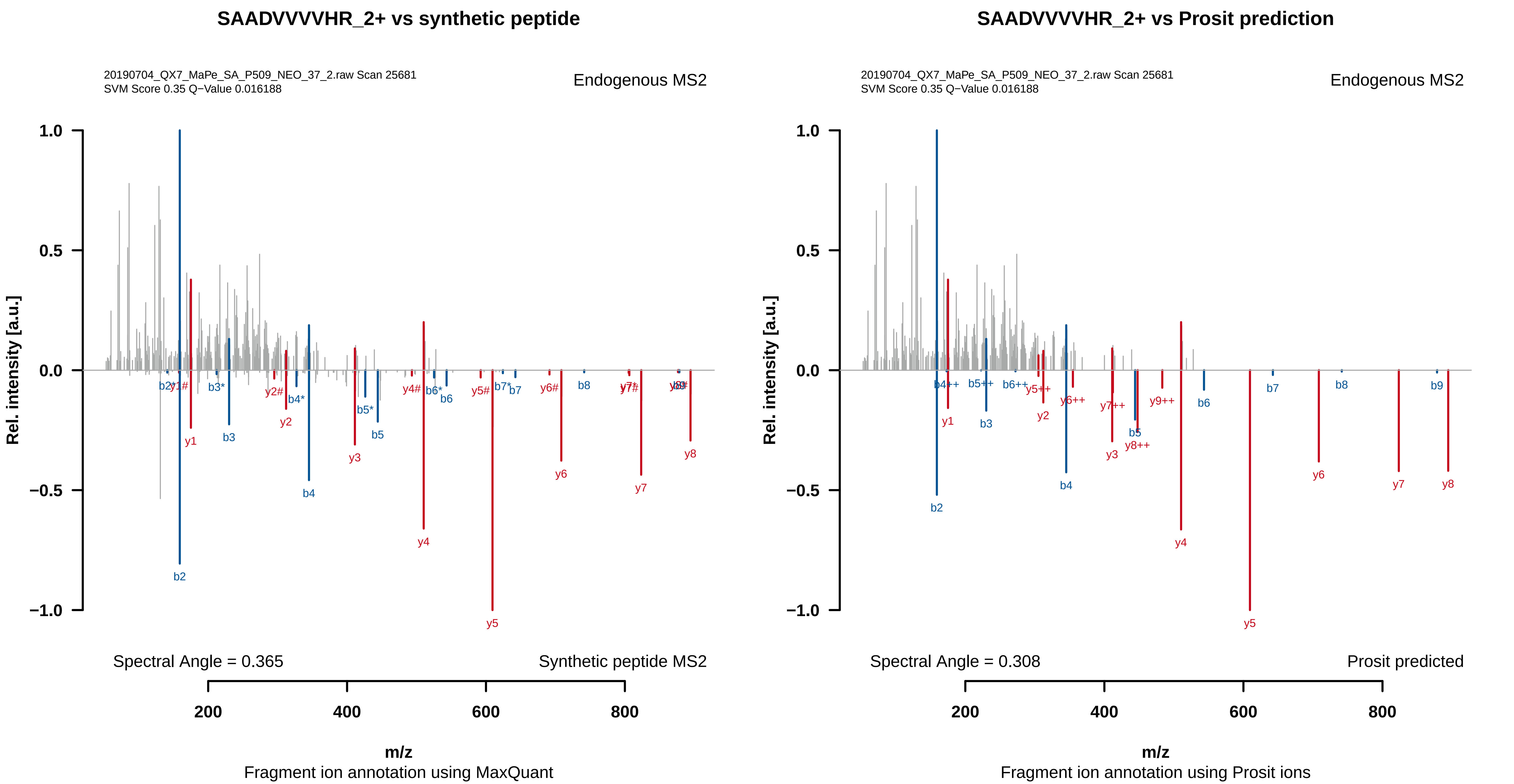
SAADVVVVHR_2+ vs synthetic peptide
20190704_QX7_MaPe_SA_P509_NEO_37_2.raw Scan 25681
SVM Score 0.35 Q−Value 0.016188
Endogenous MS2
Rel. intensity [a.u.]
1.0
0.5
0.0
−0.5
−1.0
b2
b3
b4
b5
b6
b7
b8
y1
y2
y3
y4
y5
y6
y7
y8
Spectral Angle = 0.365
Synthetic peptide MS2
200
400
600
800
m/z
Fragment ion annotation using MaxQuant
SAADVVVVHR_2+ vs Prosit prediction
20190704_QX7_MaPe_SA_P509_NEO_37_2.raw Scan 25681
SVM Score 0.35 Q−Value 0.016188
Endogenous MS2
Rel. intensity [a.u.]
1.0
0.5
0.0
−0.5
−1.0
b2
b3
b4
b5
b6
b7
b8
b9
y1
y2
y3
y4
y5
y6
y7
y8
Spectral Angle = 0.308
Prosit predicted
200
400
600
800
m/z
Fragment ion annotation using Prosit ions

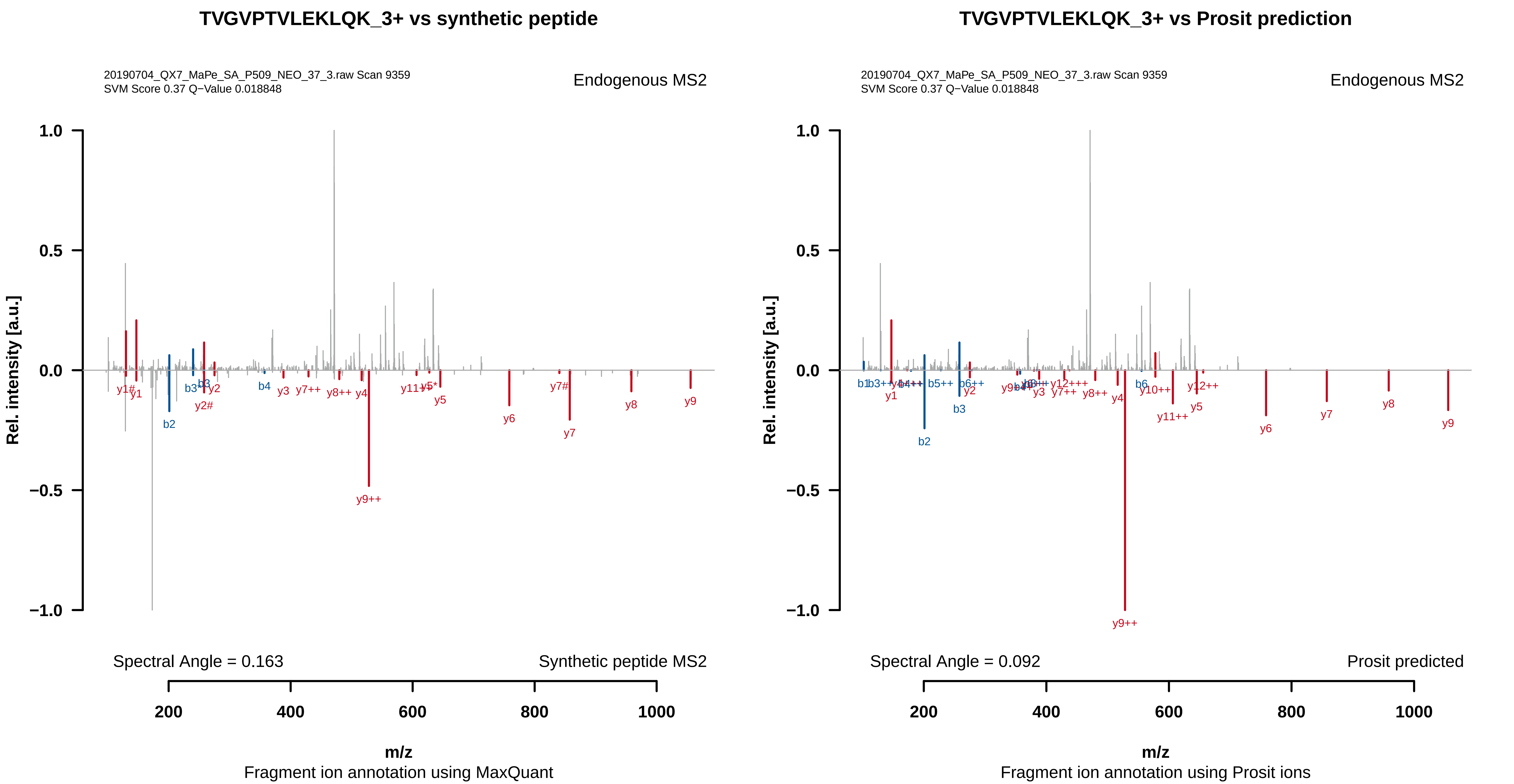

TVGVPTVLEKLQK_3+ vs synthetic peptide
20190704_QX7_MaPe_SA_P509_NEO_37_3.raw Scan 9359
SVM Score 0.37 Q−Value 0.018848
Endogenous MS2
Rel. intensity [a.u.]
Spectral Angle = 0.163
Synthetic peptide MS2
m/z
Fragment ion annotation using MaxQuant
TVGVPTVLEKLQK_3+ vs Prosit prediction
20190704_QX7_MaPe_SA_P509_NEO_37_3.raw Scan 9359
SVM Score 0.37 Q−Value 0.018848
Endogenous MS2
Rel. intensity [a.u.]
Spectral Angle = 0.092
Prosit predicted
m/z
Fragment ion annotation using Prosit ions

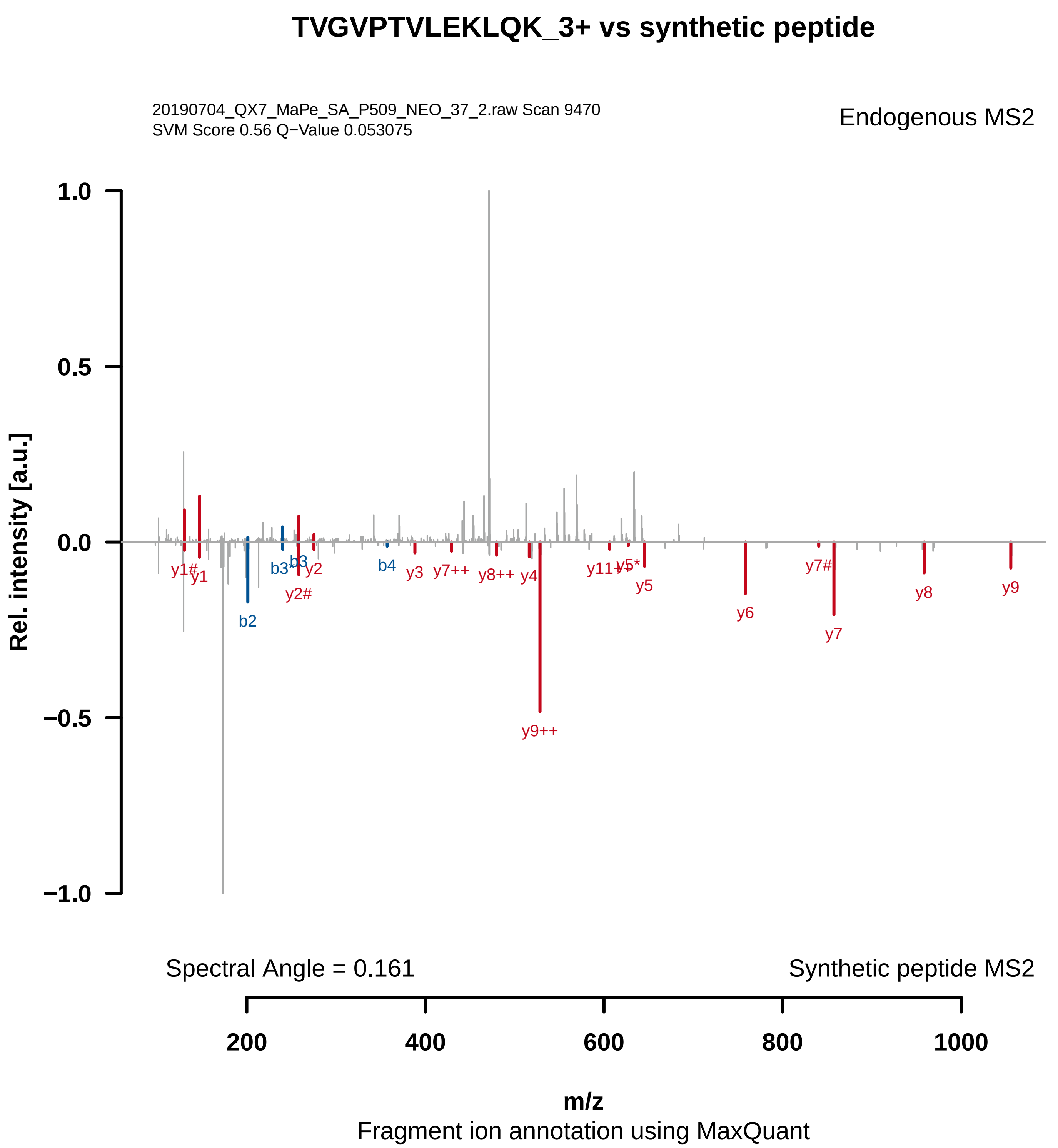
TVGVPTVLEKLQK_3+ vs synthetic peptide
20190704_QX7_MaPe_SA_P509_NEO_37_2.raw Scan 9470
SVM Score 0.56 Q−Value 0.053075
Endogenous MS2
Rel. intensity [a.u.]
1.0
0.5
0.0
−0.5
−1.0
y1#
y1
b2
b3
y2
y2#
b4
y3
y7++
y8++
y4
y9++
y11++
y5*
y5
y6
y7#
y7
y8
y9
Spectral Angle = 0.161
Synthetic peptide MS2
200
400
600
800
1000
m/z
Fragment ion annotation using MaxQuant

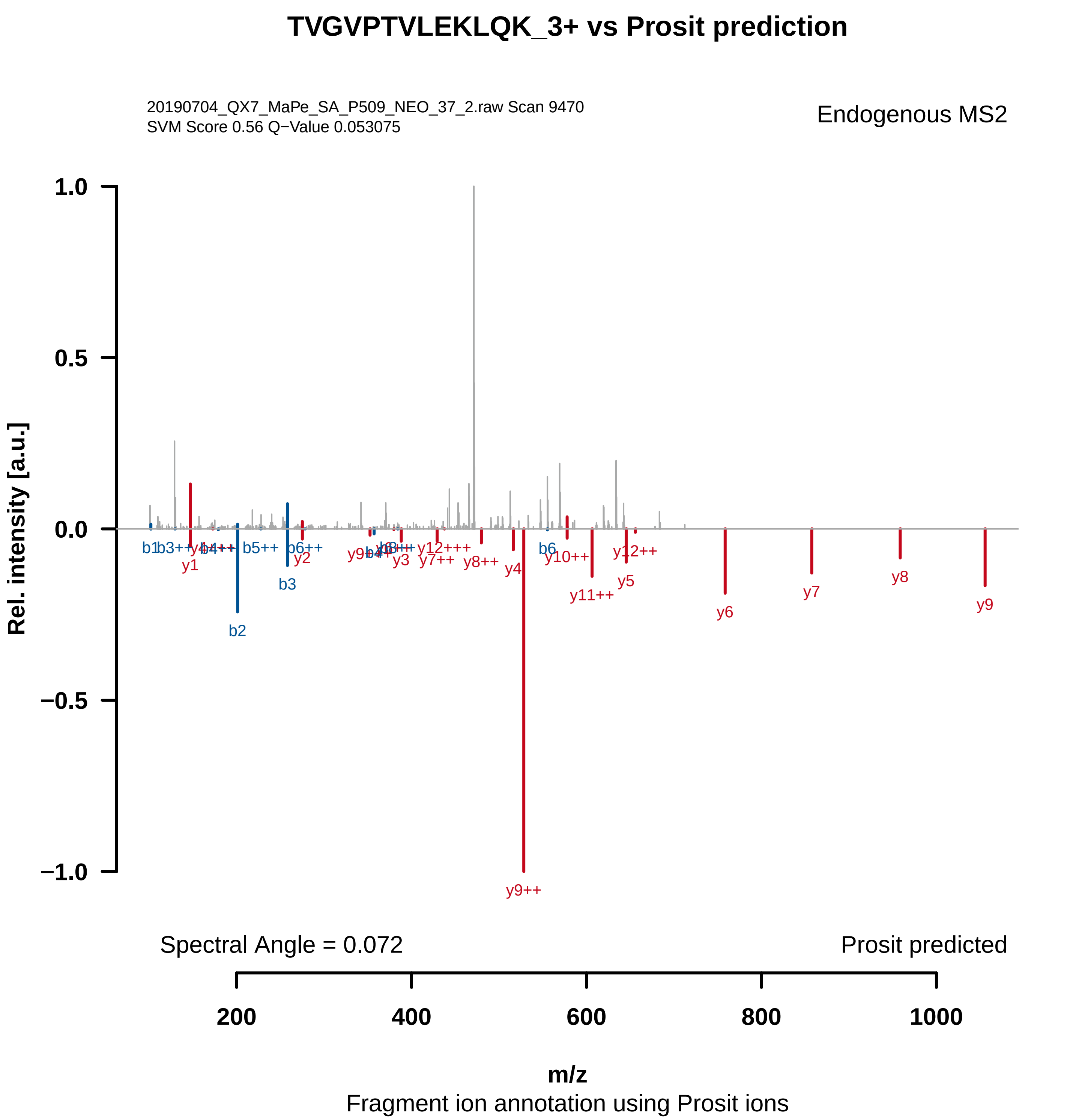
TVGVPTVLEKLQK_3+ vs Prosit prediction
20190704_QX7_MaPe_SA_P509_NEO_37_2.raw Scan 9470
SVM Score 0.56 Q−Value 0.053075
Endogenous MS2
Rel. intensity [a.u.]
1.0
0.5
0.0
−0.5
−1.0
y1
b2
b5++
b6++
y2
b3
y3
y7++
y12+++
y8++
y4
y9++
b6
y10++
y11++
y12++
y5
y6
y7
y8
y9
Spectral Angle = 0.072
Prosit predicted
200
400
600
800
1000
m/z
Fragment ion annotation using Prosit ions

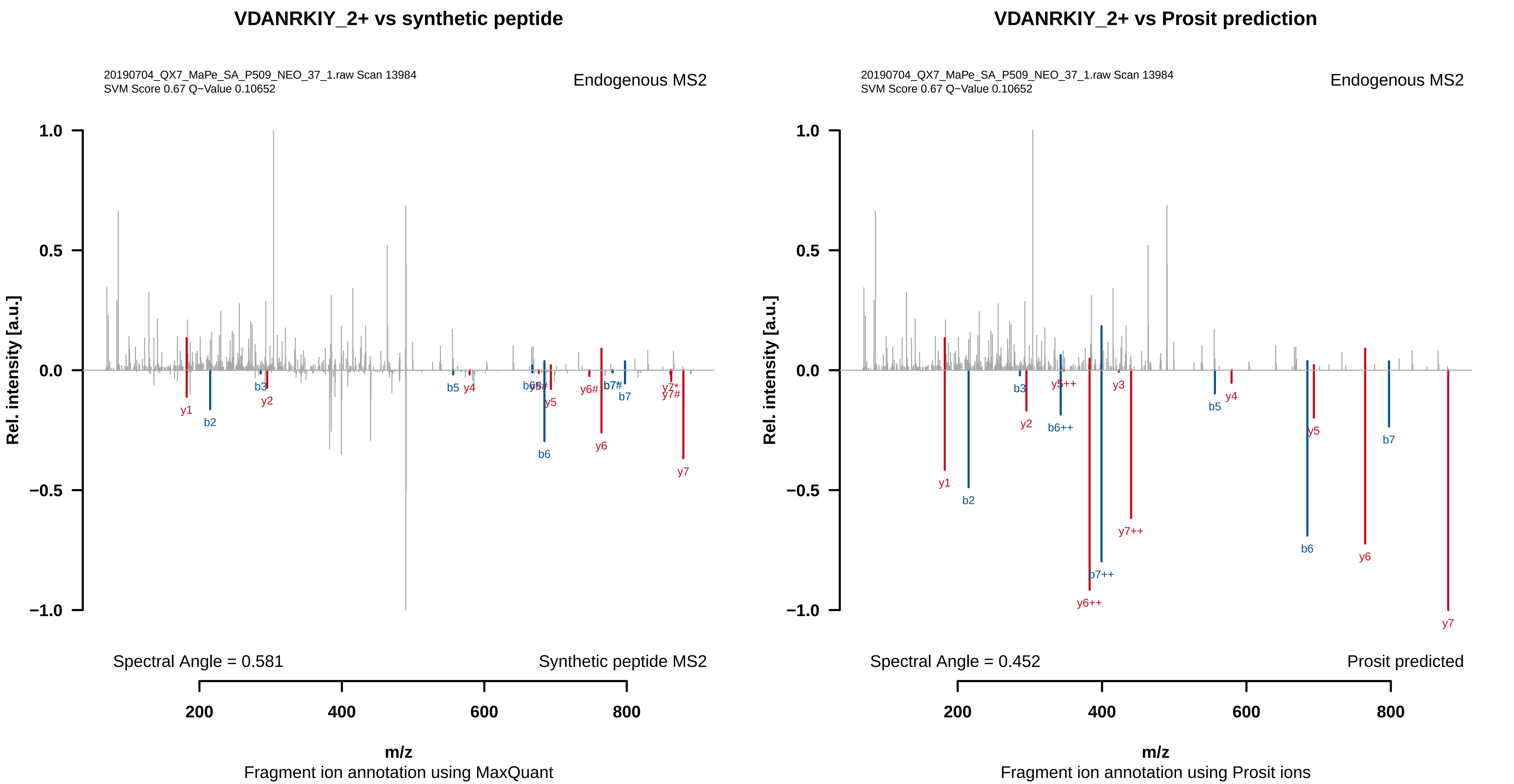
VDANRKIY_2+ vs synthetic peptide
20190704_QX7_MaPe_SA_P509_NEO_37_1.raw Scan 13984
SVM Score 0.67 Q−Value 0.10652
Endogenous MS2
Rel. intensity [a.u.]
Spectral Angle = 0.581
Synthetic peptide MS2
m/z
Fragment ion annotation using MaxQuant
VDANRKIY_2+ vs Prosit prediction
20190704_QX7_MaPe_SA_P509_NEO_37_1.raw Scan 13984
SVM Score 0.67 Q−Value 0.10652
Endogenous MS2
Rel. intensity [a.u.]
Spectral Angle = 0.452
Prosit predicted
m/z
Fragment ion annotation using Prosit ions

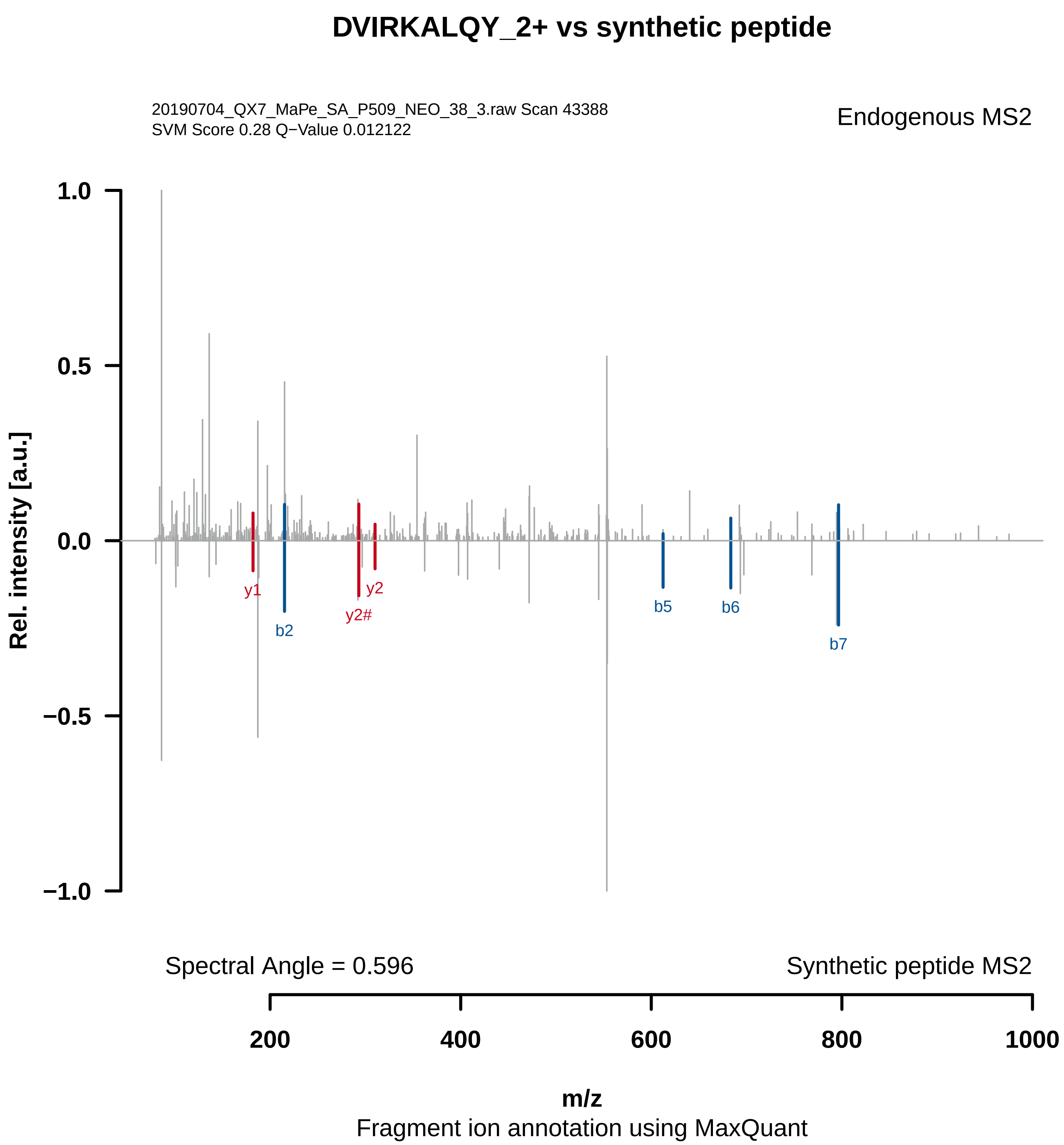
DVIRKALQY_2+ vs synthetic peptide
20190704_QX7_MaPe_SA_P509_NEO_38_3.raw Scan 43388
SVM Score 0.28 Q−Value 0.012122
Endogenous MS2
Rel. intensity [a.u.]
1.0
0.5
0.0
−0.5
−1.0
y1
b2
y2#
y2
b5
b6
b7
Spectral Angle = 0.596
Synthetic peptide MS2
200
400
600
800
1000
m/z
Fragment ion annotation using MaxQuant

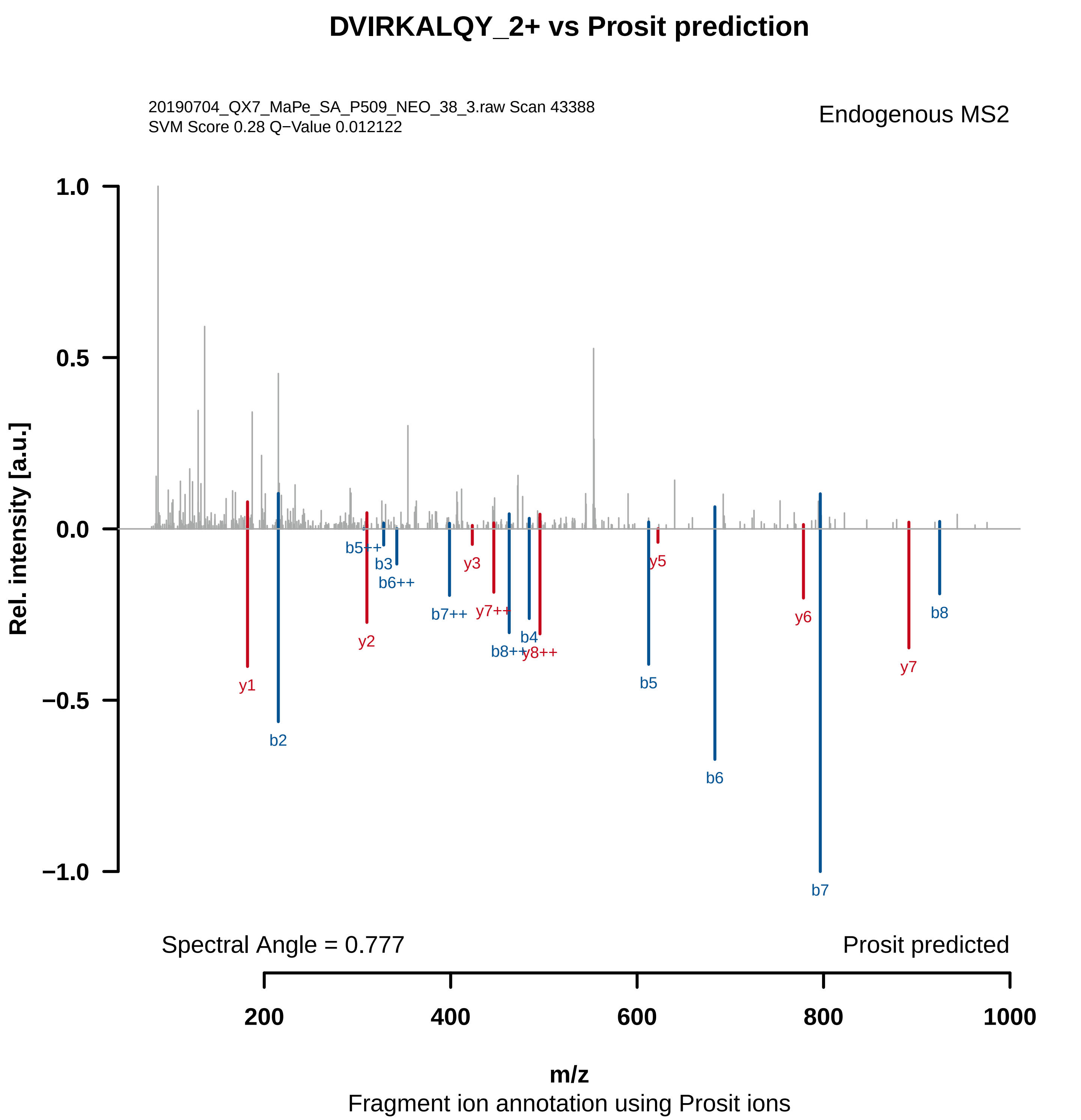
DVIRKALQY_2+ vs Prosit prediction
20190704_QX7_MaPe_SA_P509_NEO_38_3.raw Scan 43388
SVM Score 0.28 Q−Value 0.012122
Endogenous MS2
Rel. intensity [a.u.]
1.0
0.5
0.0
−0.5
−1.0
y1
b2
b5++
b3
b6++
y2
b7++
y3
y7++
b8++
b4
y8++
b5
y5
b6
y6
b7
y7
b8
Spectral Angle = 0.777
Prosit predicted
200
400
600
800
1000
m/z
Fragment ion annotation using Prosit ions

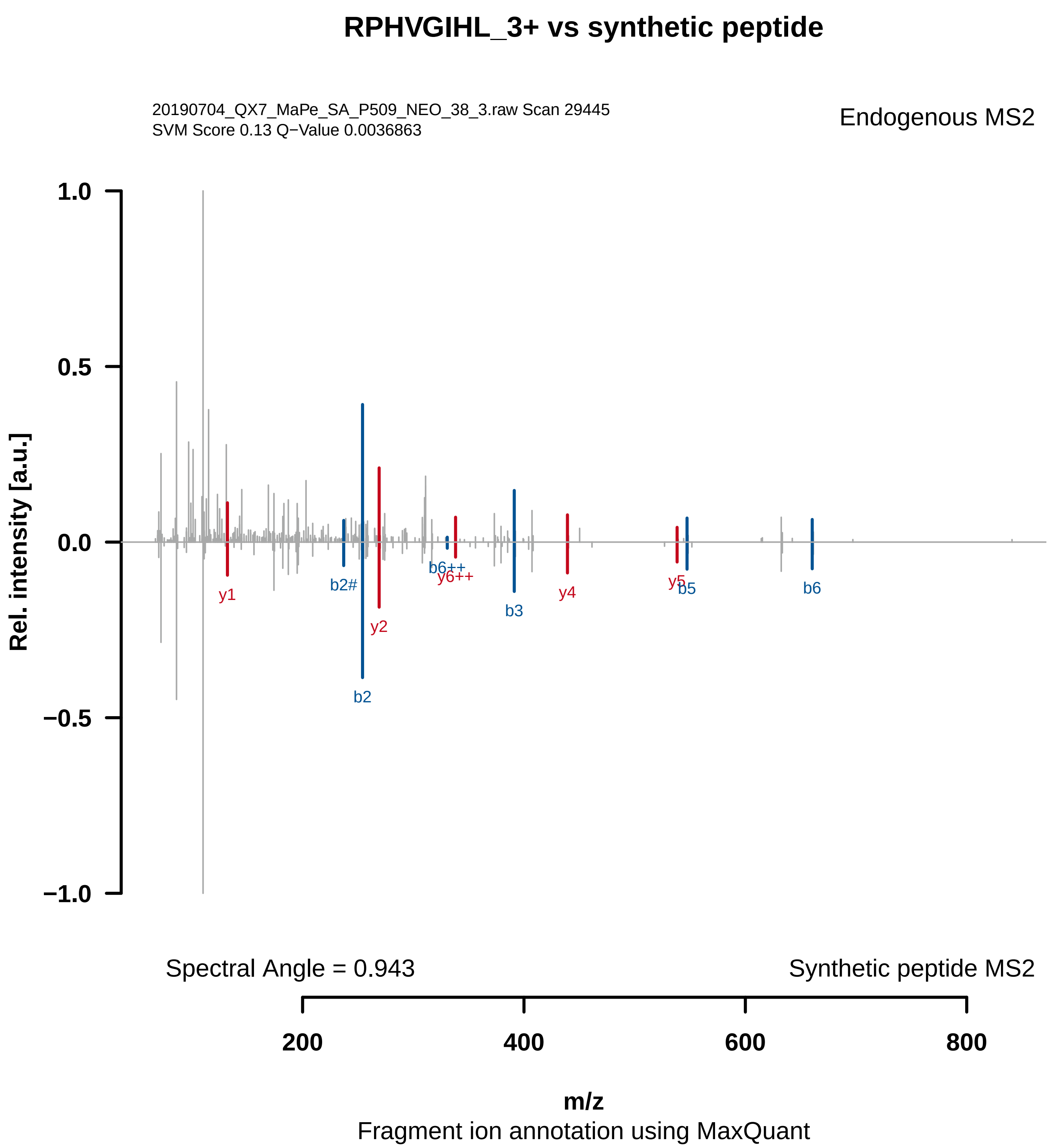
RPHVGIHL_3+ vs synthetic peptide
20190704_QX7_MaPe_SA_P509_NEO_38_3.raw Scan 29445
SVM Score 0.13 Q−Value 0.0036863
Endogenous MS2
Rel. intensity [a.u.]
1.0
0.5
0.0
−0.5
−1.0
y1
b2#
b6++
y6++
b2
y2
b3
y4
y5
b5
b6
Spectral Angle = 0.943
Synthetic peptide MS2
200
400
600
800
m/z
Fragment ion annotation using MaxQuant

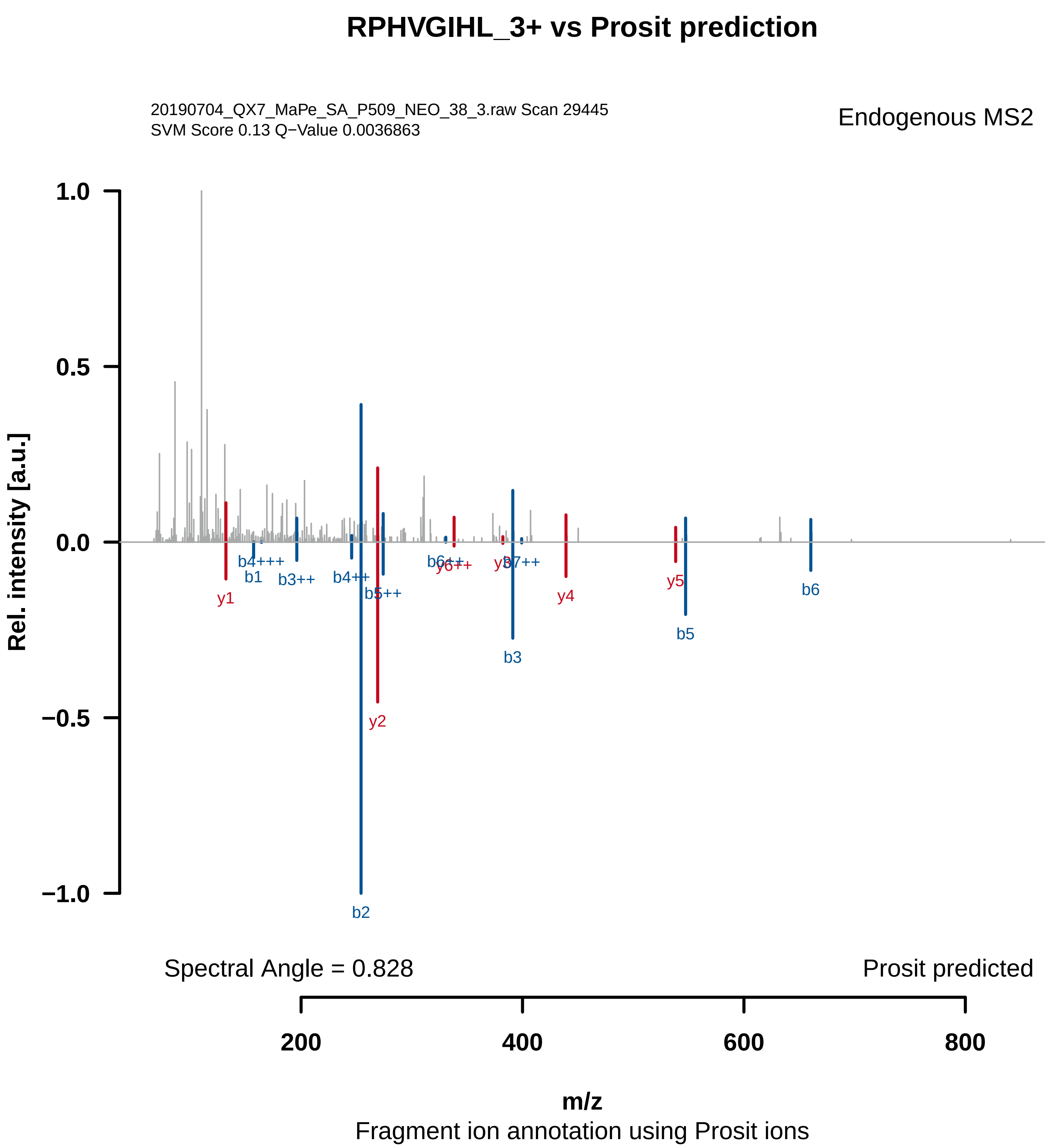
RPHVGIHL_3+ vs Prosit prediction
20190704_QX7_MaPe_SA_P509_NEO_38_3.raw Scan 29445
SVM Score 0.13 Q−Value 0.0036863
Endogenous MS2
Rel. intensity [a.u.]
1.0
0.5
0.0
−0.5
−1.0
y1
b4+++
b1
b3++
b4++
b5++
b6++
y6++
b7++
y4
y5
b6
b3
b5
y2
b2
Spectral Angle = 0.828
Prosit predicted
200
400
600
800
m/z
Fragment ion annotation using Prosit ions

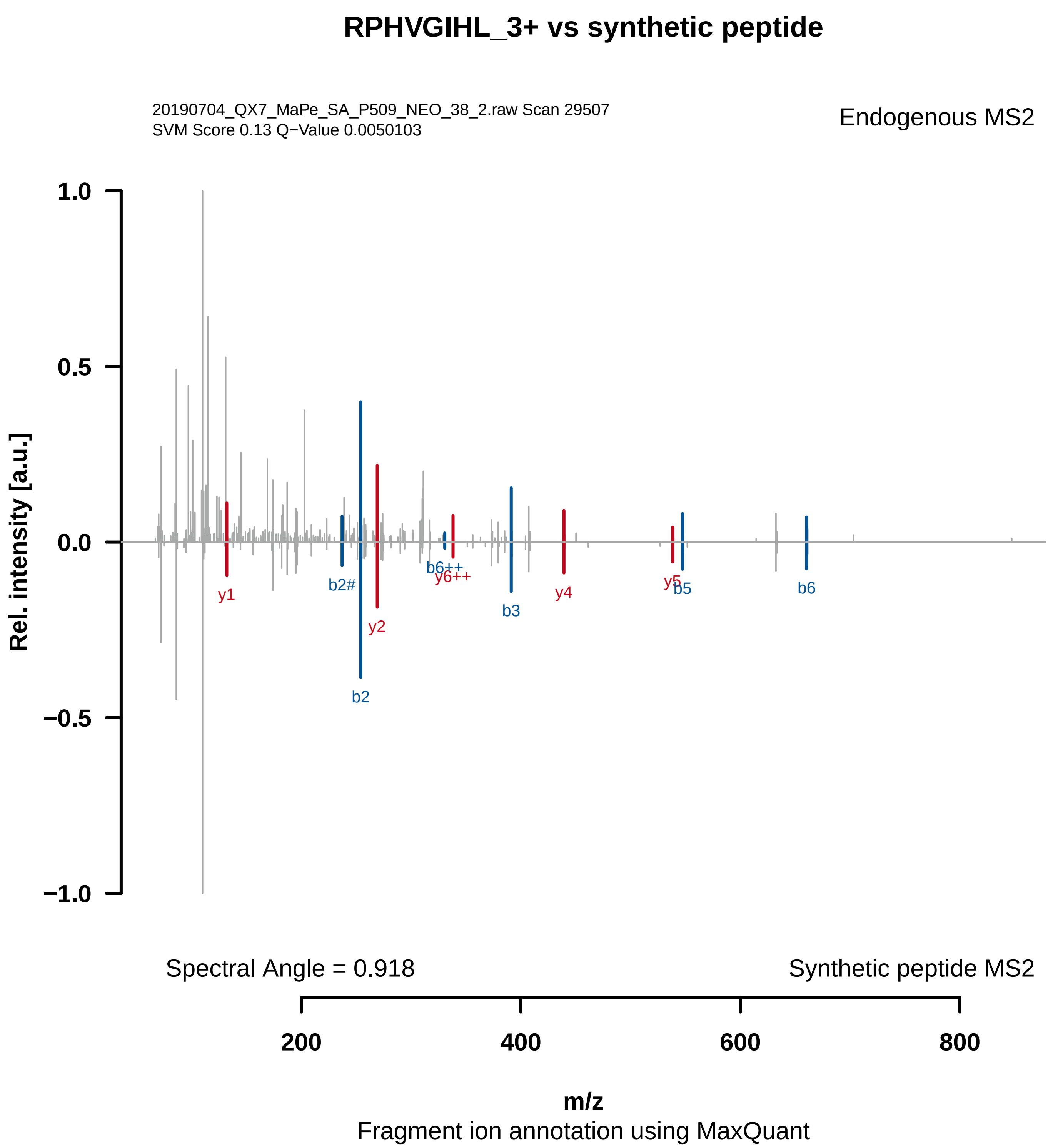
RPHVGIHL_3+ vs synthetic peptide
20190704_QX7_MaPe_SA_P509_NEO_38_2.raw Scan 29507
SVM Score 0.13 Q−Value 0.0050103
Endogenous MS2
Rel. intensity [a.u.]
1.0
0.5
0.0
−0.5
−1.0
y1
b2#
b6++
y6++
y2
b2
b3
y4
y5
b5
b6
Spectral Angle = 0.918
Synthetic peptide MS2
200
400
600
800
m/z
Fragment ion annotation using MaxQuant

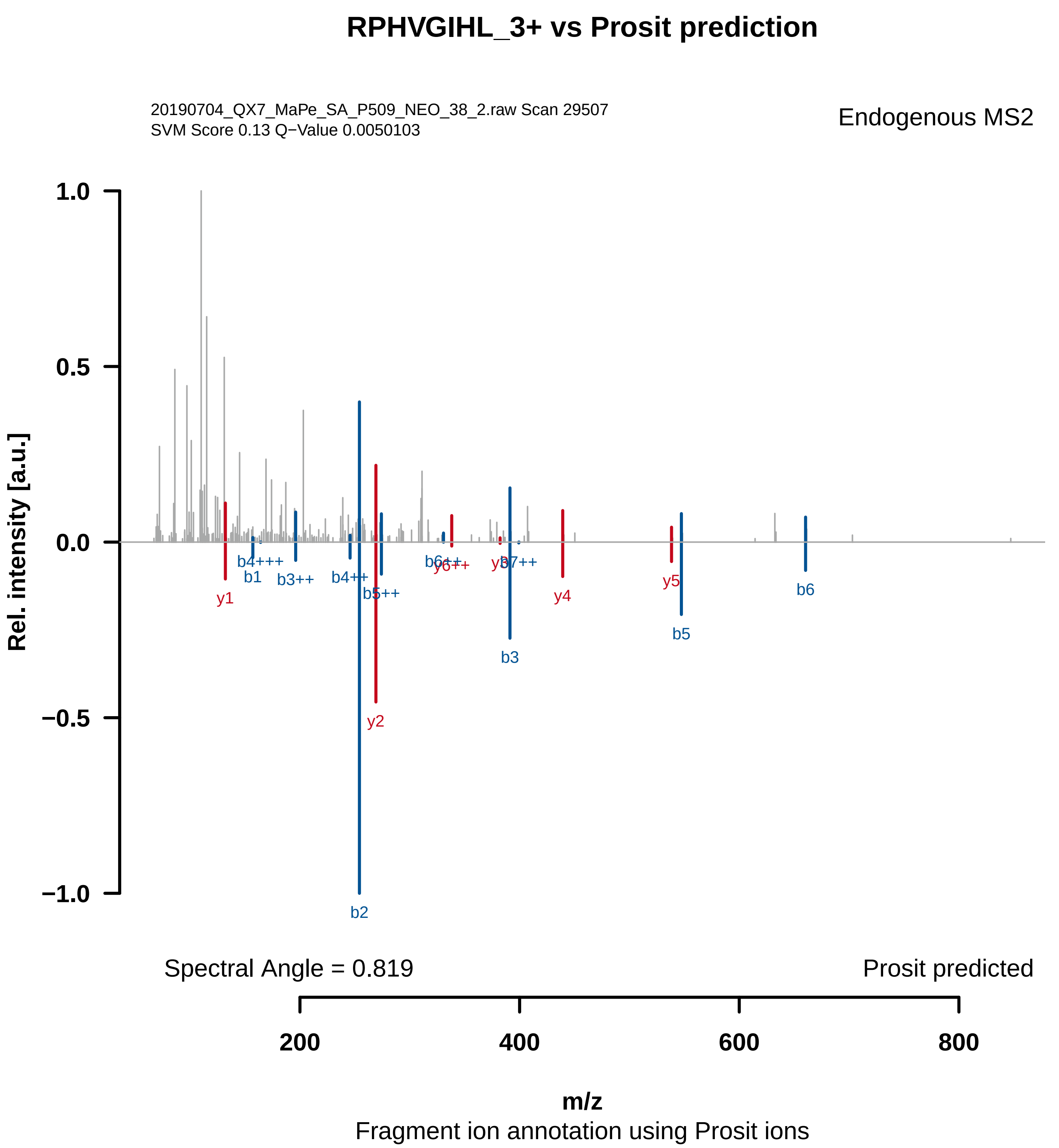
RPHVGIHL_3+ vs Prosit prediction
20190704_QX7_MaPe_SA_P509_NEO_38_2.raw Scan 29507
SVM Score 0.13 Q−Value 0.0050103
Endogenous MS2
Rel. intensity [a.u.]
1.0
0.5
0.0
−0.5
−1.0
y1
b4+++
b1
b3++
b4++
b5++
b6++
y6++
b7++
y2
b2
b3
y4
y5
b5
b6
Spectral Angle = 0.819
Prosit predicted
200
400
600
800
m/z
Fragment ion annotation using Prosit ions

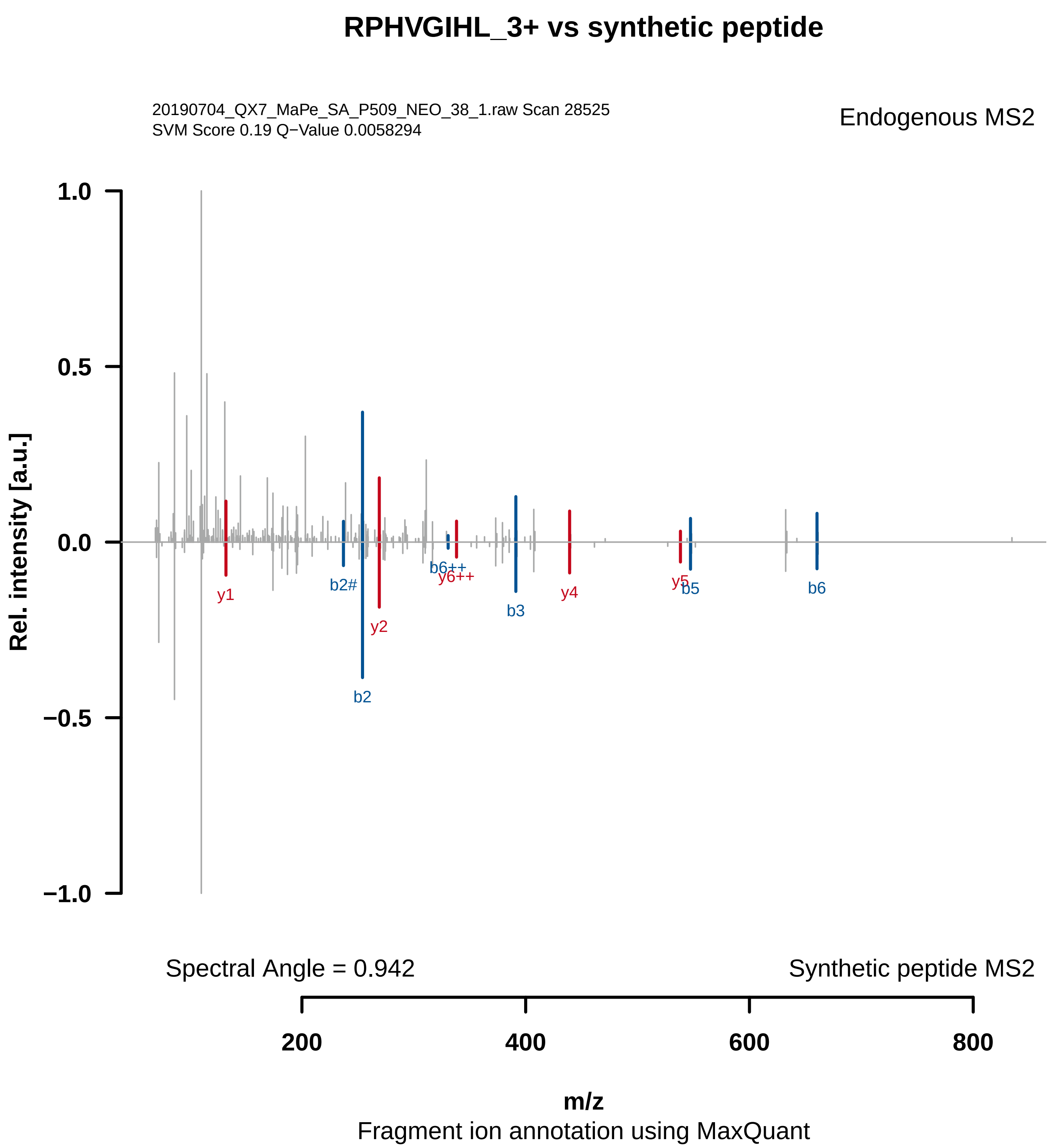

RPHVGIHL_3+ vs synthetic peptide
20190704_QX7_MaPe_SA_P509_NEO_38_1.raw Scan 28525
SVM Score 0.19 Q−Value 0.0058294
Endogenous MS2
Rel. intensity [a.u.]
1.0
0.5
0.0
−0.5
−1.0
y1
b2#
y2
b2
b6++
y6++
b3
y4
y5
b5
b6
Spectral Angle = 0.942
Synthetic peptide MS2
200
400
600
800
m/z
Fragment ion annotation using MaxQuant

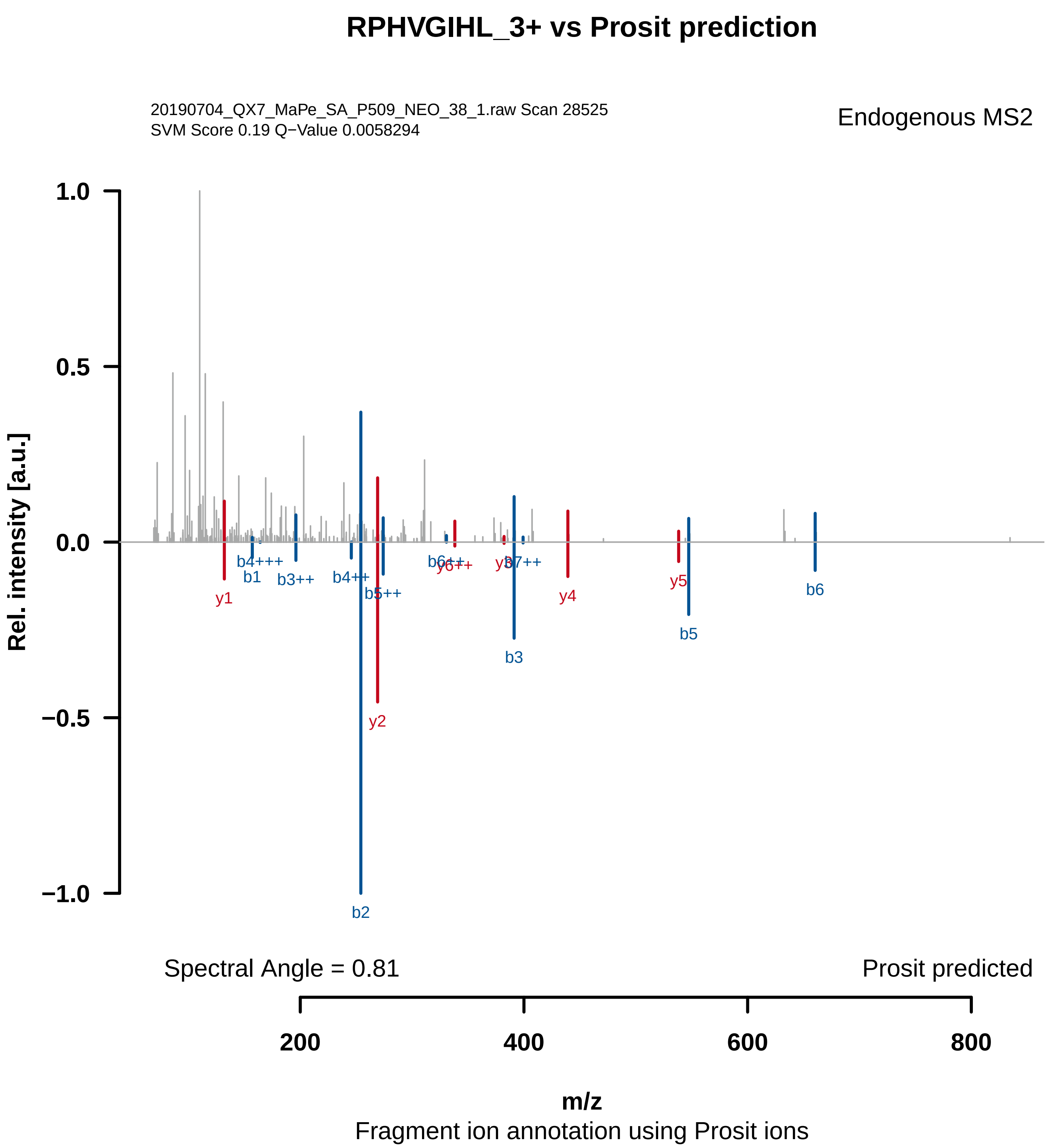

RPHVGIHL_3+ vs Prosit prediction
20190704_QX7_MaPe_SA_P509_NEO_38_1.raw Scan 28525
SVM Score 0.19 Q−Value 0.0058294
Endogenous MS2
Rel. intensity [a.u.]
1.0
0.5
0.0
−0.5
−1.0
y1
b4+++
b1
b3++
b4++
b5++
b2
y2
b6++
y6++
y3
b7++
b3
y4
y5
b5
b6
Spectral Angle = 0.81
Prosit predicted
200
400
600
800
m/z
Fragment ion annotation using Prosit ions

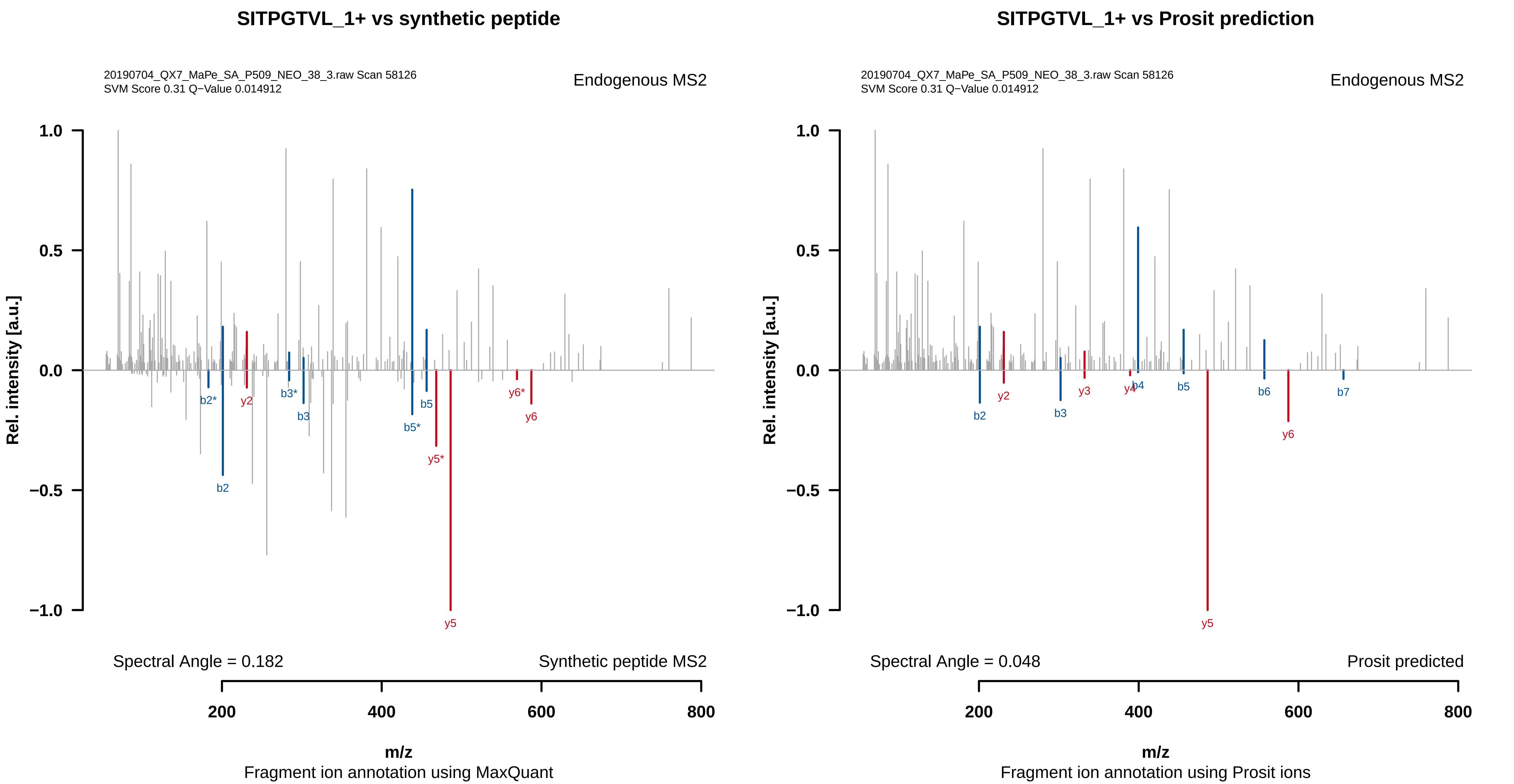

SITPGTVL_1+ vs synthetic peptide
20190704_QX7_MaPe_SA_P509_NEO_38_3.raw Scan 58126
SVM Score 0.31 Q−Value 0.014912
Endogenous MS2
Rel. intensity [a.u.]
b2* b2 y2 b3* b3 b5* b5 y5* y5 y6* y6
Spectral Angle = 0.182
Synthetic peptide MS2
m/z
Fragment ion annotation using MaxQuant
SITPGTVL_1+ vs Prosit prediction
20190704_QX7_MaPe_SA_P509_NEO_38_3.raw Scan 58126
SVM Score 0.31 Q−Value 0.014912
Endogenous MS2
Rel. intensity [a.u.]
b2 y2 b3 y3 y4 b4 b5 y5 b6 y6 b7
Spectral Angle = 0.048
Prosit predicted
m/z
Fragment ion annotation using Prosit ions

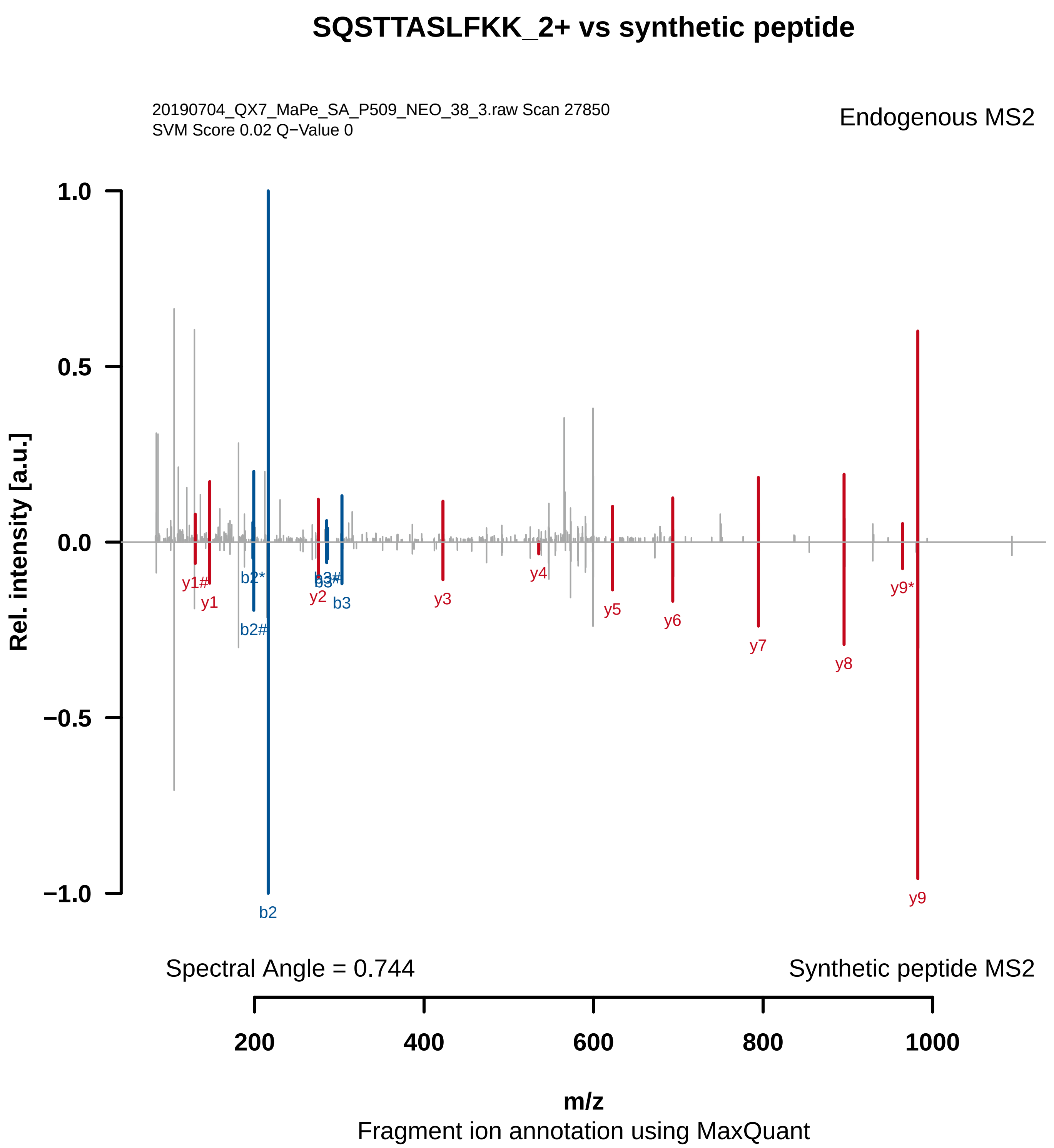

SQSTTASLFKK_2+ vs synthetic peptide
20190704_QX7_MaPe_SA_P509_NEO_38_3.raw Scan 27850
SVM Score 0.02 Q−Value 0
Endogenous MS2
1.0
0.5
0.0
−0.5
−1.0
Rel. intensity [a.u.]
y1#
y1
b2*
b2#
b2
y2
b3
y3
y4
y5
y6
y7
y8
y9*
y9
Spectral Angle = 0.744
Synthetic peptide MS2
200
400
600
800
1000
m/z
Fragment ion annotation using MaxQuant

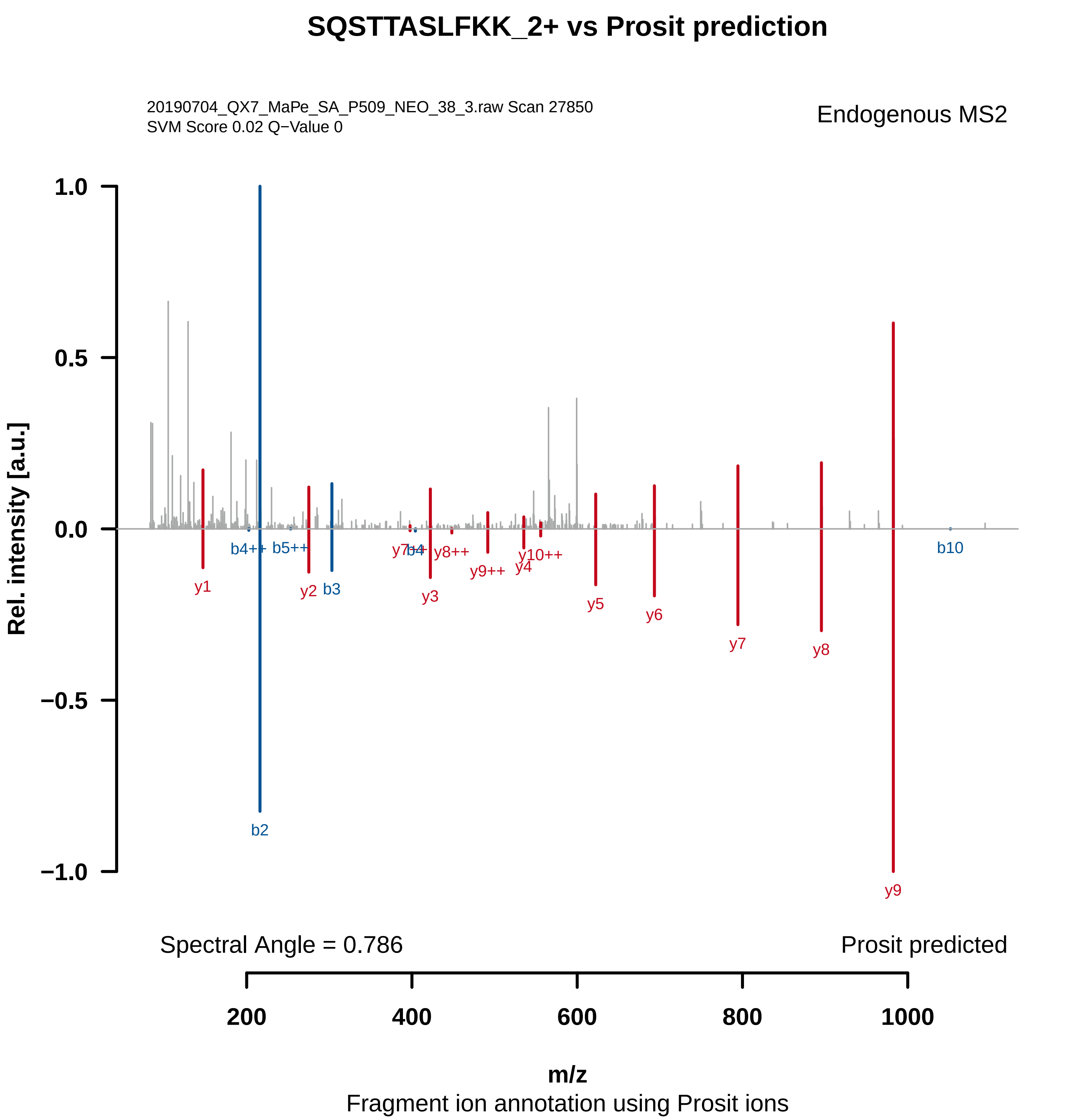

SQSTTASLFKK_2+ vs Prosit prediction
20190704_QX7_MaPe_SA_P509_NEO_38_3.raw Scan 27850
SVM Score 0.02 Q−Value 0
Endogenous MS2
1.0
0.5
0.0
−0.5
−1.0
Rel. intensity [a.u.]
y1
b4++
b5++
b2
y2
b3
y3
y8++
y9++
y4
y10++
y5
y6
y7
y8
y9
b10
Spectral Angle = 0.786
Prosit predicted
200
400
600
800
1000
m/z
Fragment ion annotation using Prosit ions

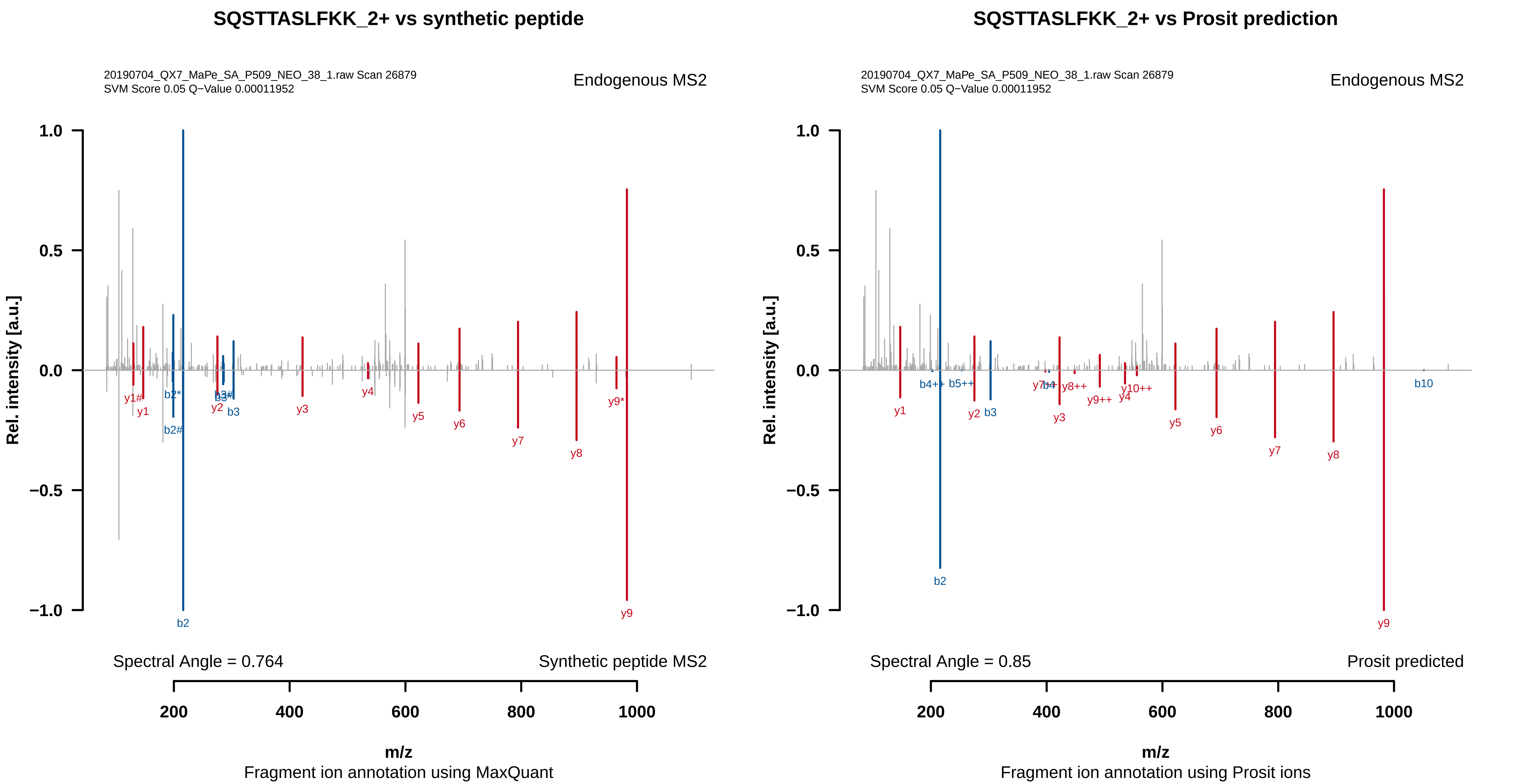
SQSTTASLFKK_2+ vs synthetic peptide
20190704_QX7_MaPe_SA_P509_NEO_38_1.raw Scan 26879
SVM Score 0.05 Q−Value 0.00011952
Endogenous MS2
Rel. intensity [a.u.]
1.0
0.5
0.0
−0.5
−1.0
y1#
y1
b2*
b2#
b3#
b3*
y2
b3
y3
y4
y5
y6
y7
y8
y9*
b2
y9
Spectral Angle = 0.764
Synthetic peptide MS2
200
400
600
800
1000
m/z
Fragment ion annotation using MaxQuant
SQSTTASLFKK_2+ vs Prosit prediction
20190704_QX7_MaPe_SA_P509_NEO_38_1.raw Scan 26879
SVM Score 0.05 Q−Value 0.00011952
Endogenous MS2
Rel. intensity [a.u.]
1.0
0.5
0.0
−0.5
−1.0
b4++
b5++
y1
y2
b3
y7++
b4
y8++
y3
y9++
y4
y10++
y5
y6
y7
y8
b10
b2
y9
Spectral Angle = 0.85
Prosit predicted
200
400
600
800
1000
m/z
Fragment ion annotation using Prosit ions

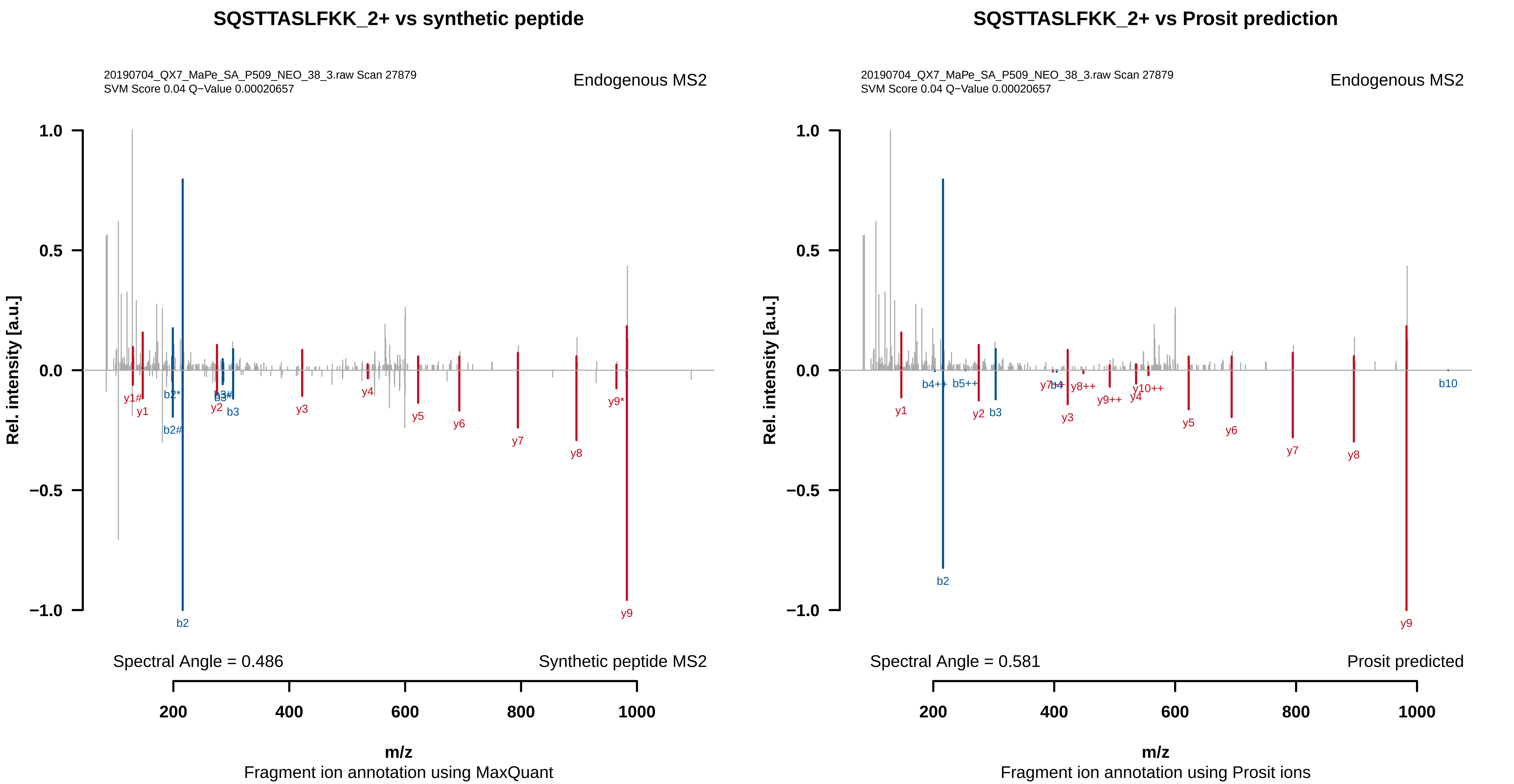

SQSTTASLFKK_2+ vs synthetic peptide
20190704_QX7_MaPe_SA_P509_NEO_38_3.raw Scan 27879
SVM Score 0.04 Q−Value 0.00020657
Endogenous MS2
Rel. intensity [a.u.]
1.0
0.5
0.0
−0.5
−1.0
y1#
y1
b2*
b2#
b2
y2
b3#
b3*
b3
y3
y4
y5
y6
y7
y8
y9*
y9
Spectral Angle = 0.486
Synthetic peptide MS2
200
400
600
800
1000
m/z
Fragment ion annotation using MaxQuant
SQSTTASLFKK_2+ vs Prosit prediction
20190704_QX7_MaPe_SA_P509_NEO_38_3.raw Scan 27879
SVM Score 0.04 Q−Value 0.00020657
Endogenous MS2
Rel. intensity [a.u.]
1.0
0.5
0.0
−0.5
−1.0
y1
b4++
b5++
b2
y2
b3
y7++
b4
y3
y8++
y9++
y4
y10++
y5
y6
y7
y8
y9
b10
Spectral Angle = 0.581
Prosit predicted
200
400
600
800
1000
m/z
Fragment ion annotation using Prosit ions

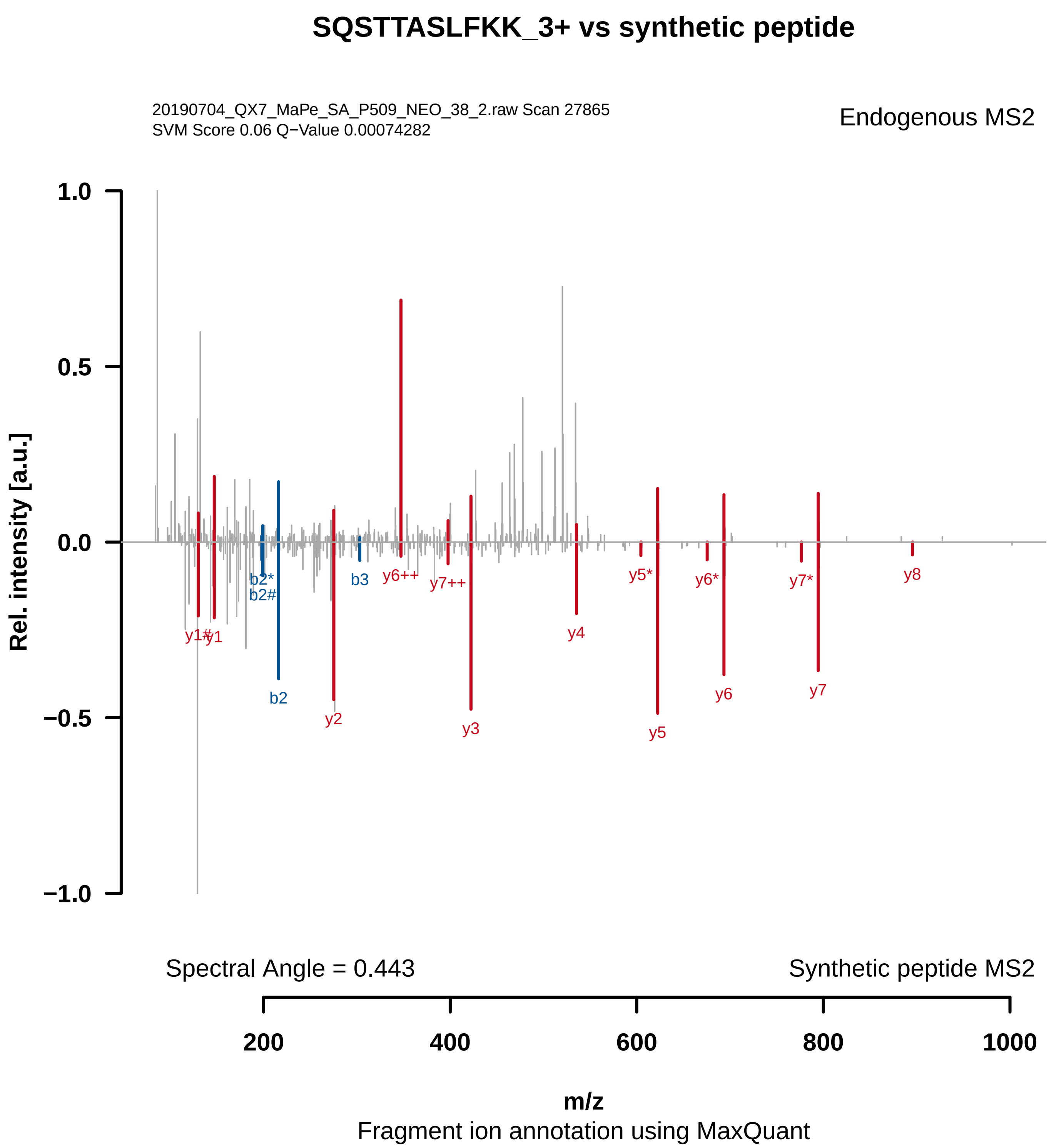
SQSTTASLFKK_3+ vs synthetic peptide
20190704_QX7_MaPe_SA_P509_NEO_38_2.raw Scan 27865
SVM Score 0.06 Q−Value 0.00074282
Endogenous MS2
Rel. intensity [a.u.]
1.0
0.5
0.0
−0.5
−1.0
b2*
b2#
b3
y6++
y7++
y5*
y6*
y7*
y8
y1#
y1
b2
y2
y3
y4
y5
y6
y7
Spectral Angle = 0.443
Synthetic peptide MS2
200
400
600
800
1000
m/z
Fragment ion annotation using MaxQuant

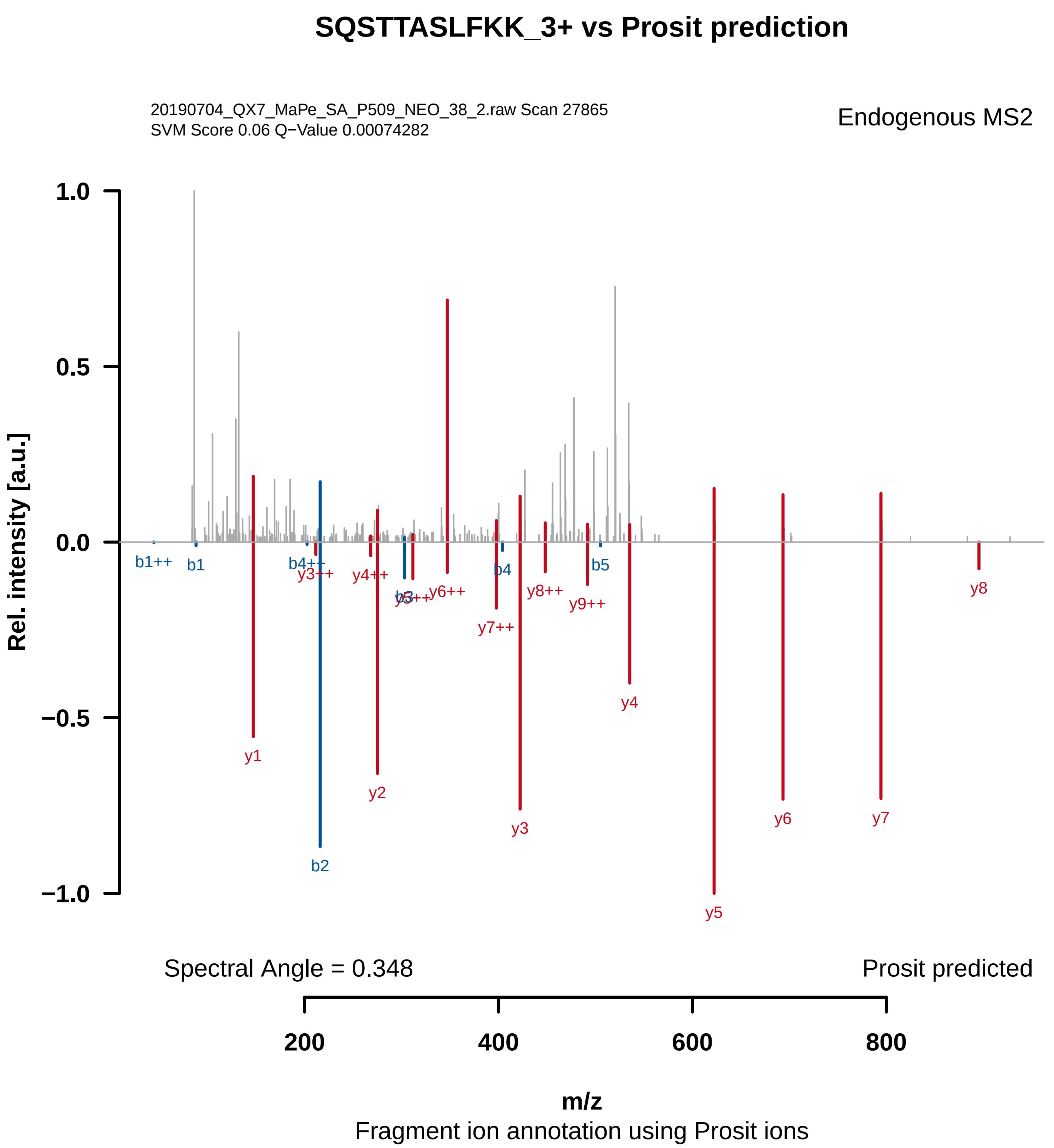
SQSTTASLFKK_3+ vs Prosit prediction
20190704_QX7_MaPe_SA_P509_NEO_38_2.raw Scan 27865
SVM Score 0.06 Q−Value 0.00074282
Endogenous MS2
Rel. intensity [a.u.]
1.0
0.5
0.0
−0.5
−1.0
b1++
b1
b4++
y3++
y4++
b3
y5++
y6++
b4
y7++
y8++
y9++
b5
y8
y1
b2
y2
y3
y4
y5
y6
y7
Spectral Angle = 0.348
Prosit predicted
200
400
600
800
m/z
Fragment ion annotation using Prosit ions

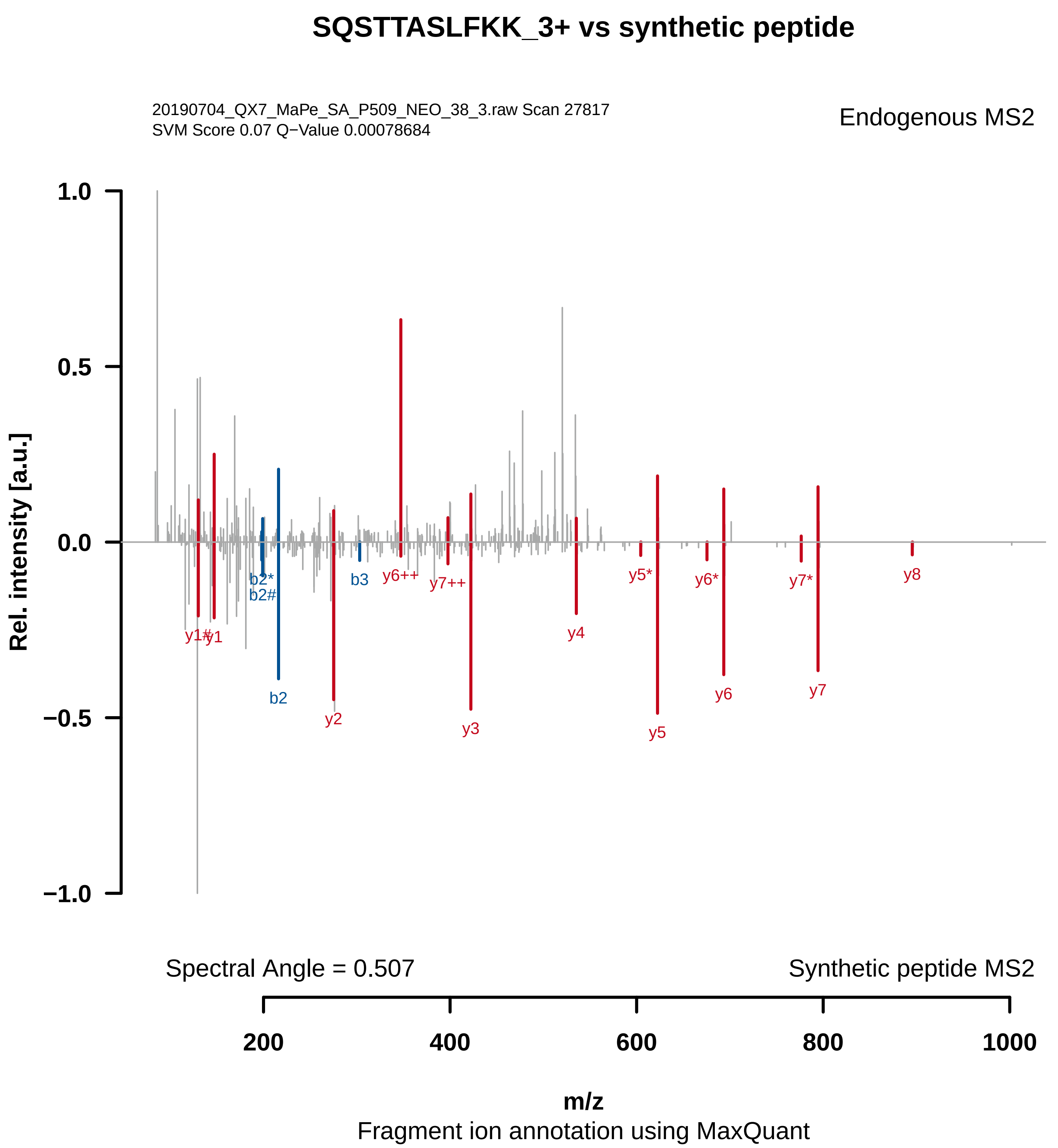
SQSTTASLFKK_3+ vs synthetic peptide
20190704_QX7_MaPe_SA_P509_NEO_38_3.raw Scan 27817
SVM Score 0.07 Q−Value 0.00078684
Endogenous MS2
Rel. intensity [a.u.]
1.0
0.5
0.0
−0.5
−1.0
Spectral Angle = 0.507
Synthetic peptide MS2
200
400
600
800
1000
m/z
Fragment ion annotation using MaxQuant

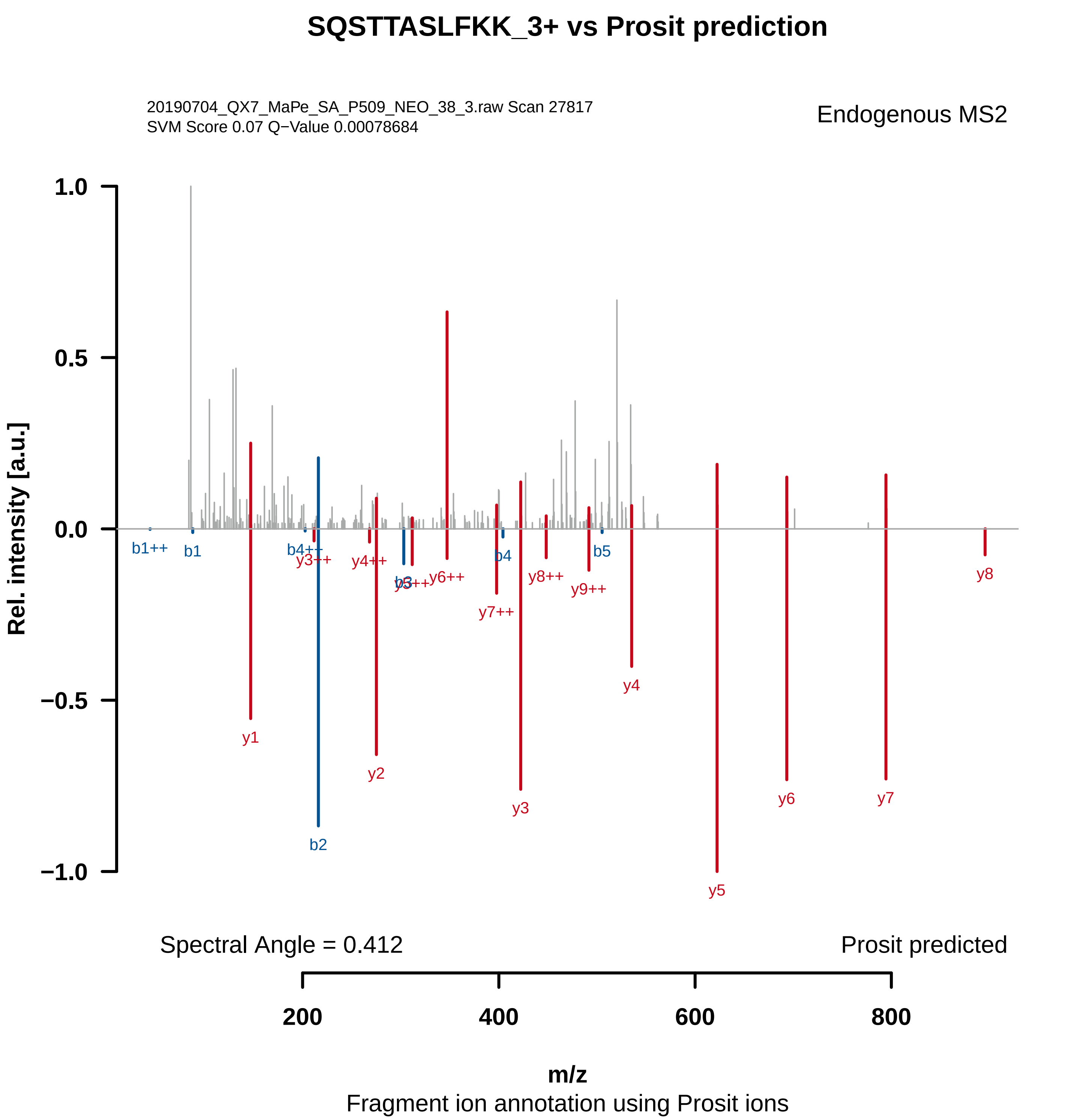
SQSTTASLFKK_3+ vs Prosit prediction
20190704_QX7_MaPe_SA_P509_NEO_38_3.raw Scan 27817
SVM Score 0.07 Q−Value 0.00078684
Endogenous MS2
Rel. intensity [a.u.]
1.0
0.5
0.0
−0.5
−1.0
Spectral Angle = 0.412
Prosit predicted
200
400
600
800
m/z
Fragment ion annotation using Prosit ions

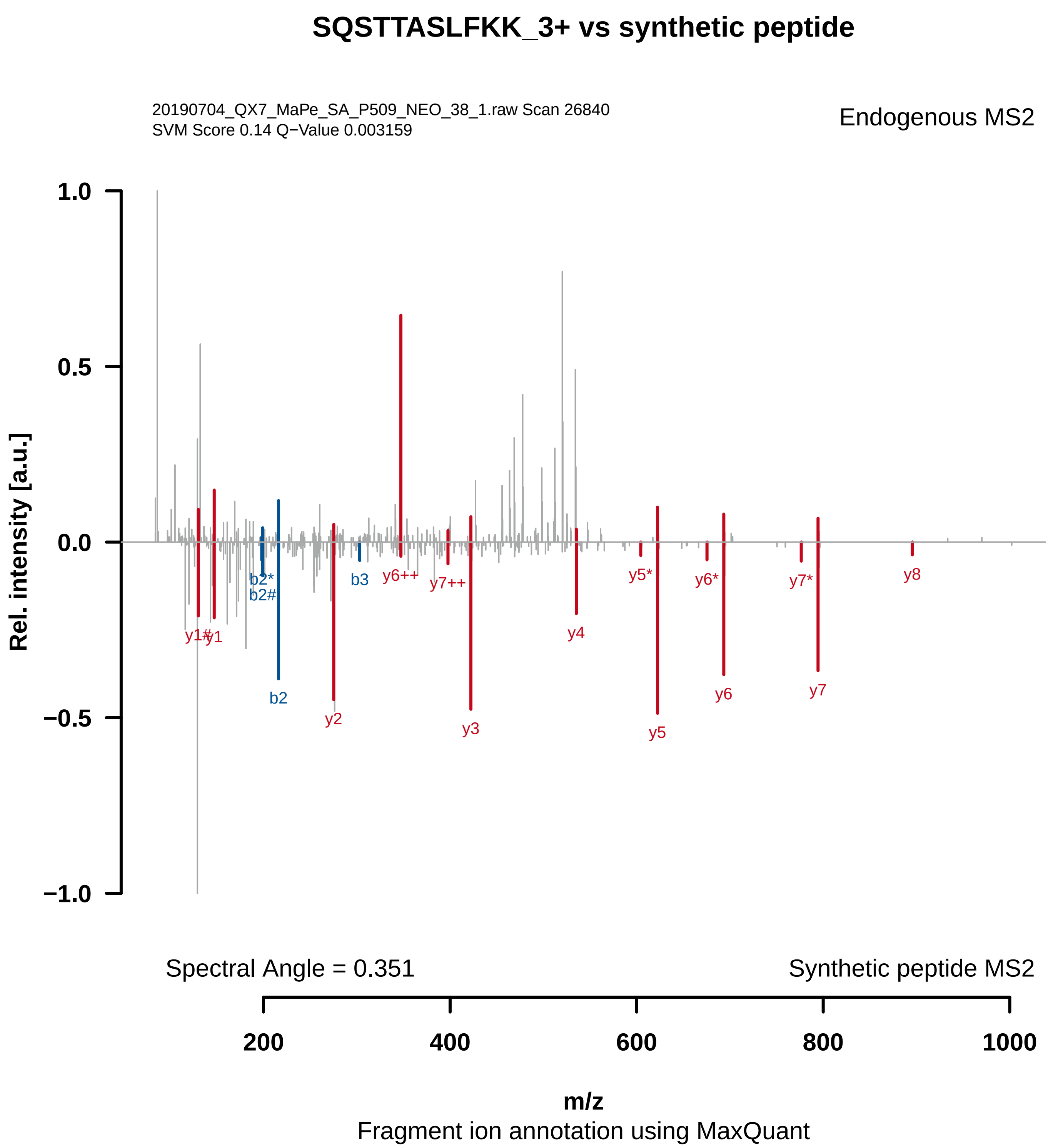
SQSTTASLFKK_3+ vs synthetic peptide
20190704_QX7_MaPe_SA_P509_NEO_38_1.raw Scan 26840
SVM Score 0.14 Q−Value 0.003159
Endogenous MS2
Rel. intensity [a.u.]
1.0
0.5
0.0
−0.5
−1.0
Spectral Angle = 0.351
Synthetic peptide MS2
200
400
600
800
1000
m/z
Fragment ion annotation using MaxQuant

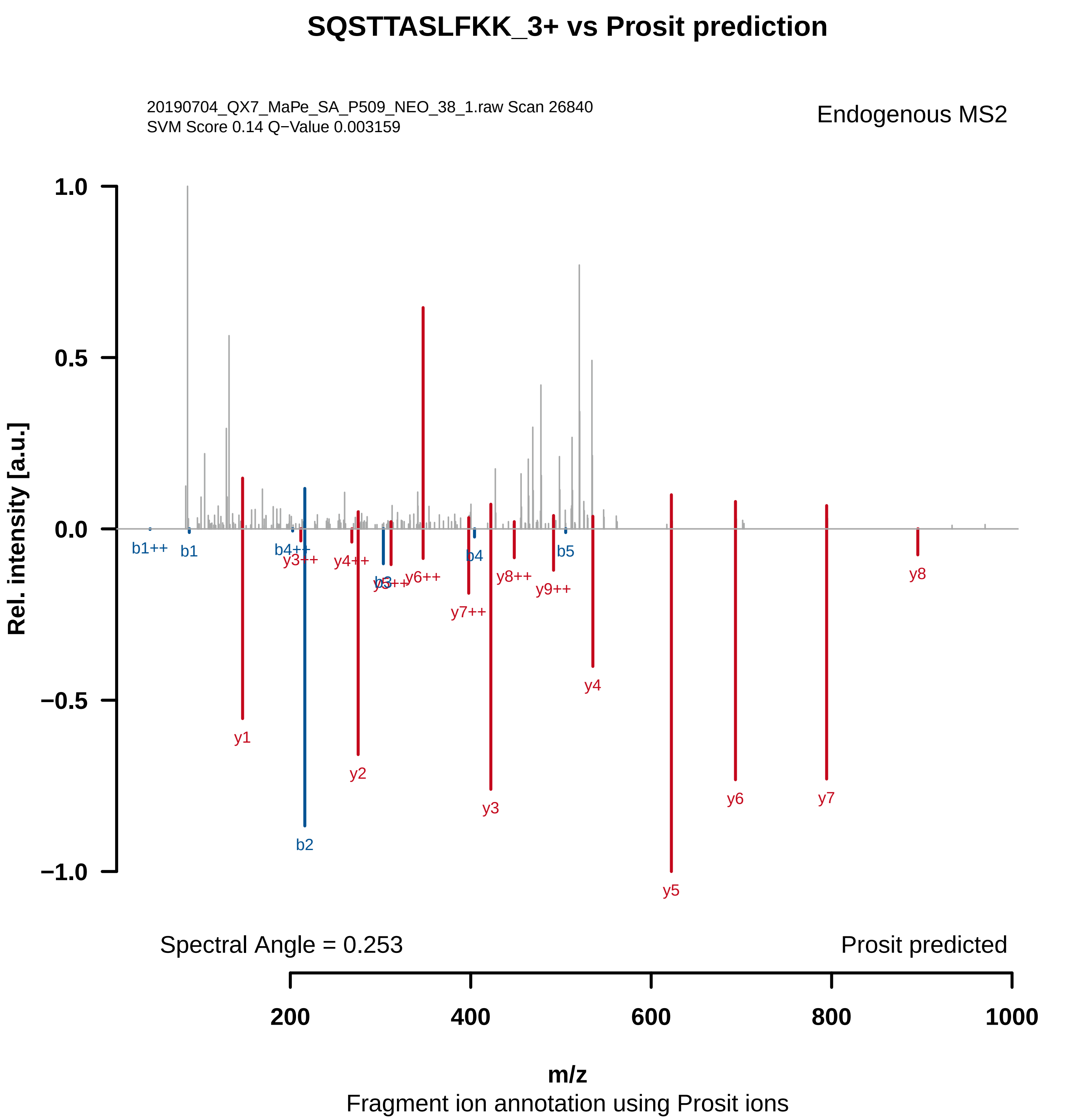
SQSTTASLFKK_3+ vs Prosit prediction
20190704_QX7_MaPe_SA_P509_NEO_38_1.raw Scan 26840
SVM Score 0.14 Q−Value 0.003159
Endogenous MS2
Rel. intensity [a.u.]
1.0
0.5
0.0
−0.5
−1.0
Spectral Angle = 0.253
Prosit predicted
200
400
600
800
1000
m/z
Fragment ion annotation using Prosit ions

SQSTTASLFKK_2+ vs synthetic peptide

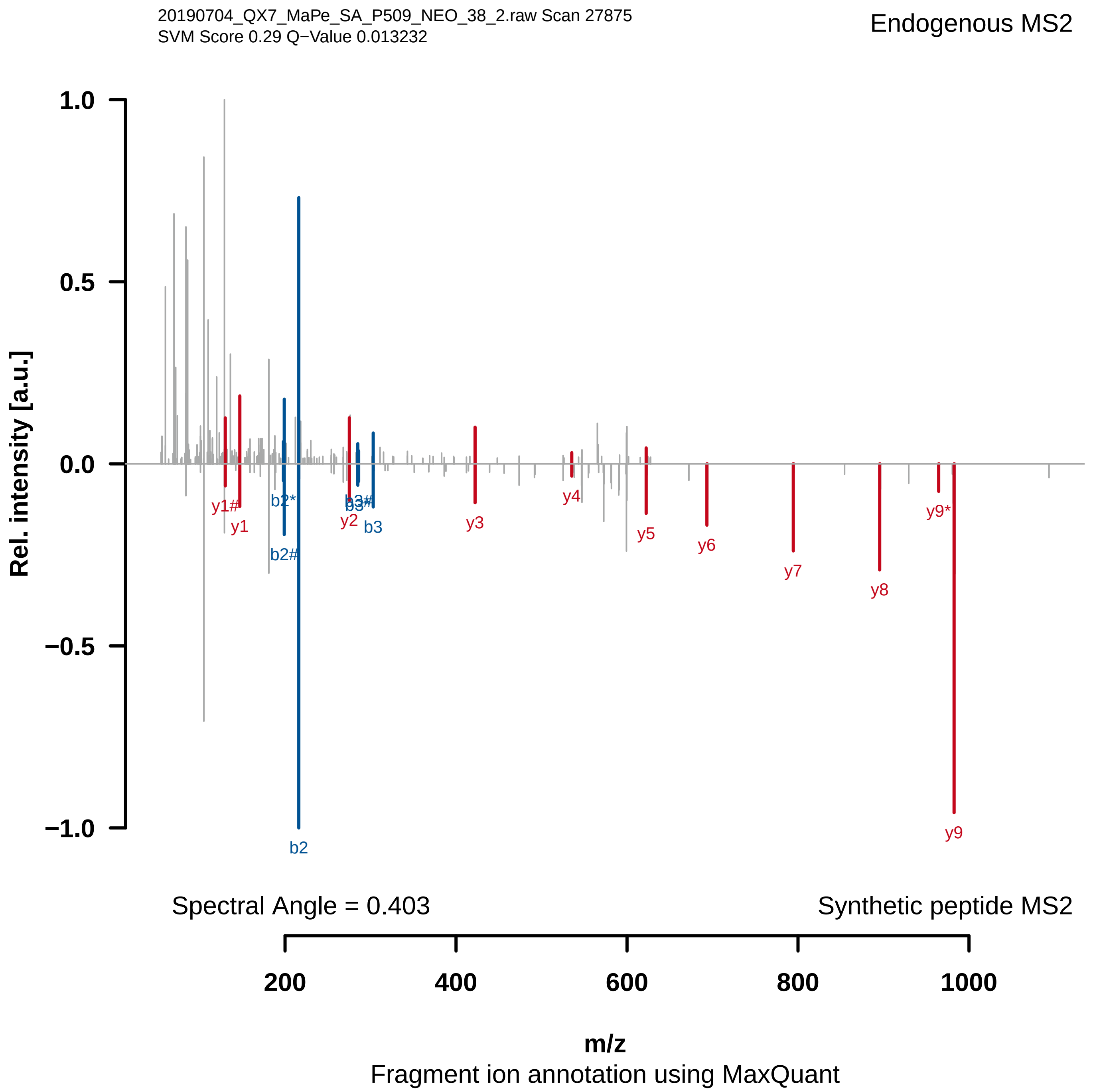

SQSTTASLFKK_2+ vs Prosit prediction

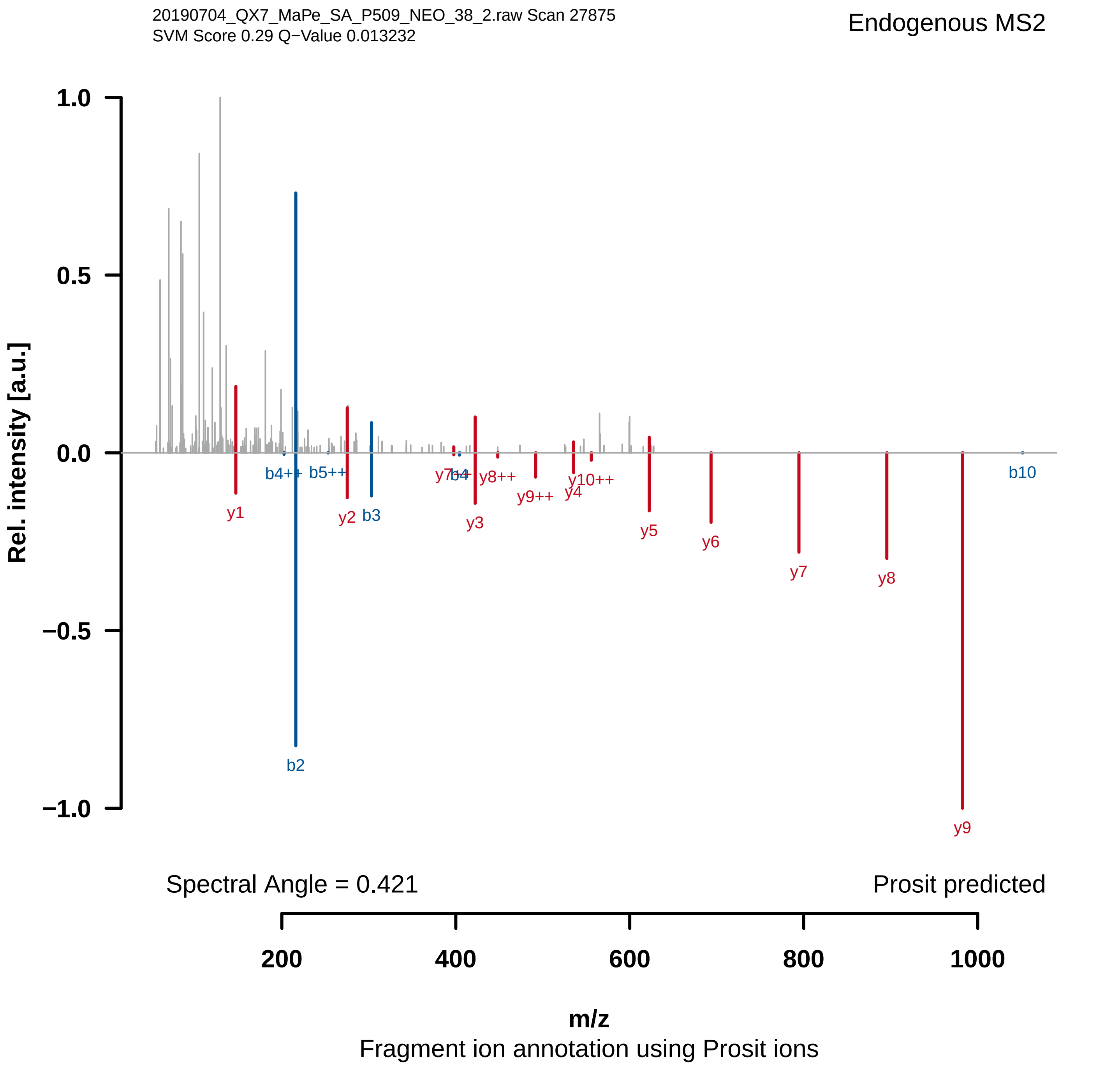

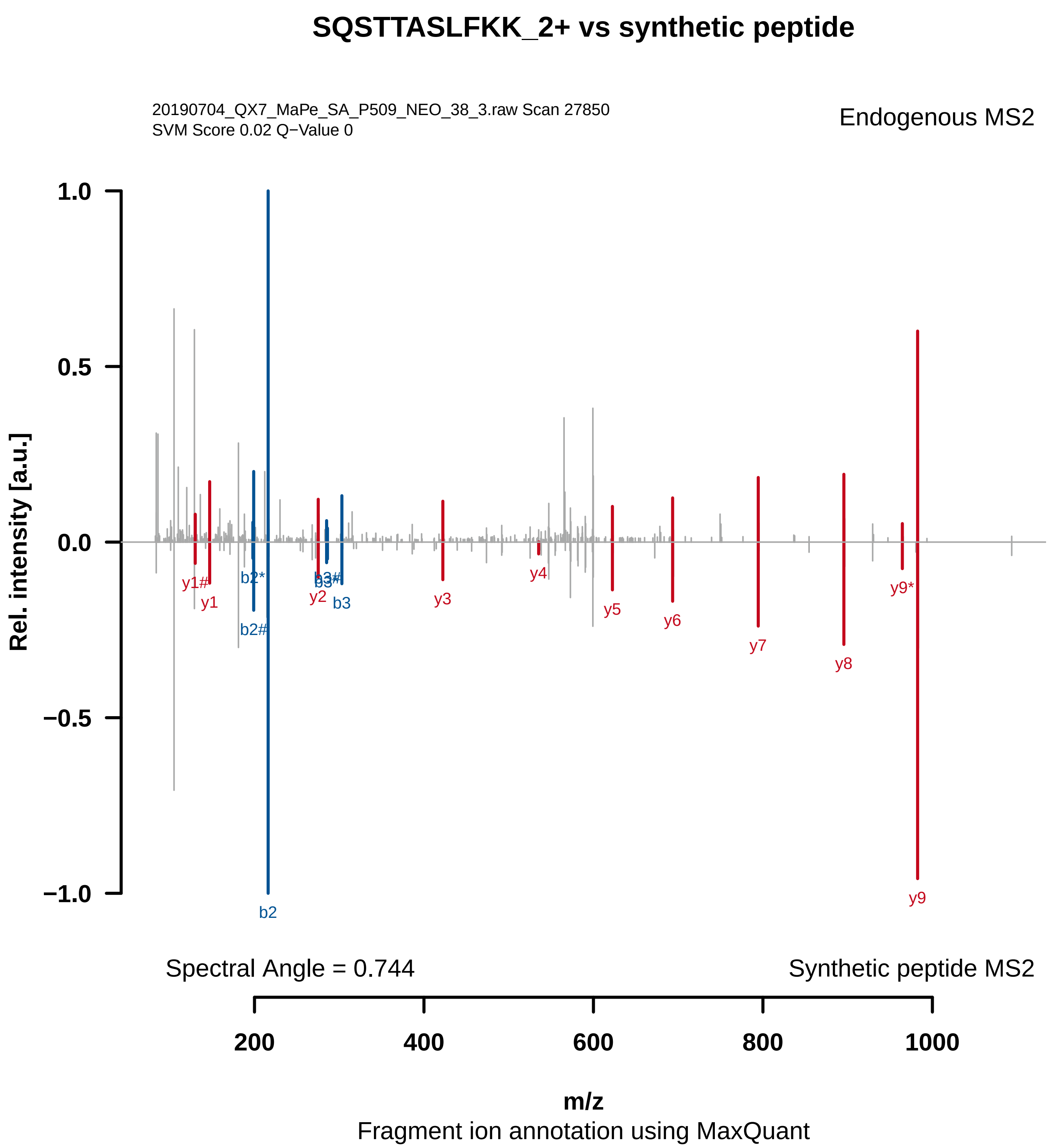
SQSTTASLFKK_2+ vs synthetic peptide
20190704_QX7_MaPe_SA_P509_NEO_38_3.raw Scan 27850
SVM Score 0.02 Q−Value 0
Endogenous MS2
Rel. intensity [a.u.]
1.0
0.5
0.0
−0.5
−1.0
y1#
y1
b2*
b2#
b3#
b3*
y2
b3
y3
y4
y5
y6
y7
y8
y9*
b2
y9
Spectral Angle = 0.744
Synthetic peptide MS2
200
400
600
800
1000
m/z
Fragment ion annotation using MaxQuant

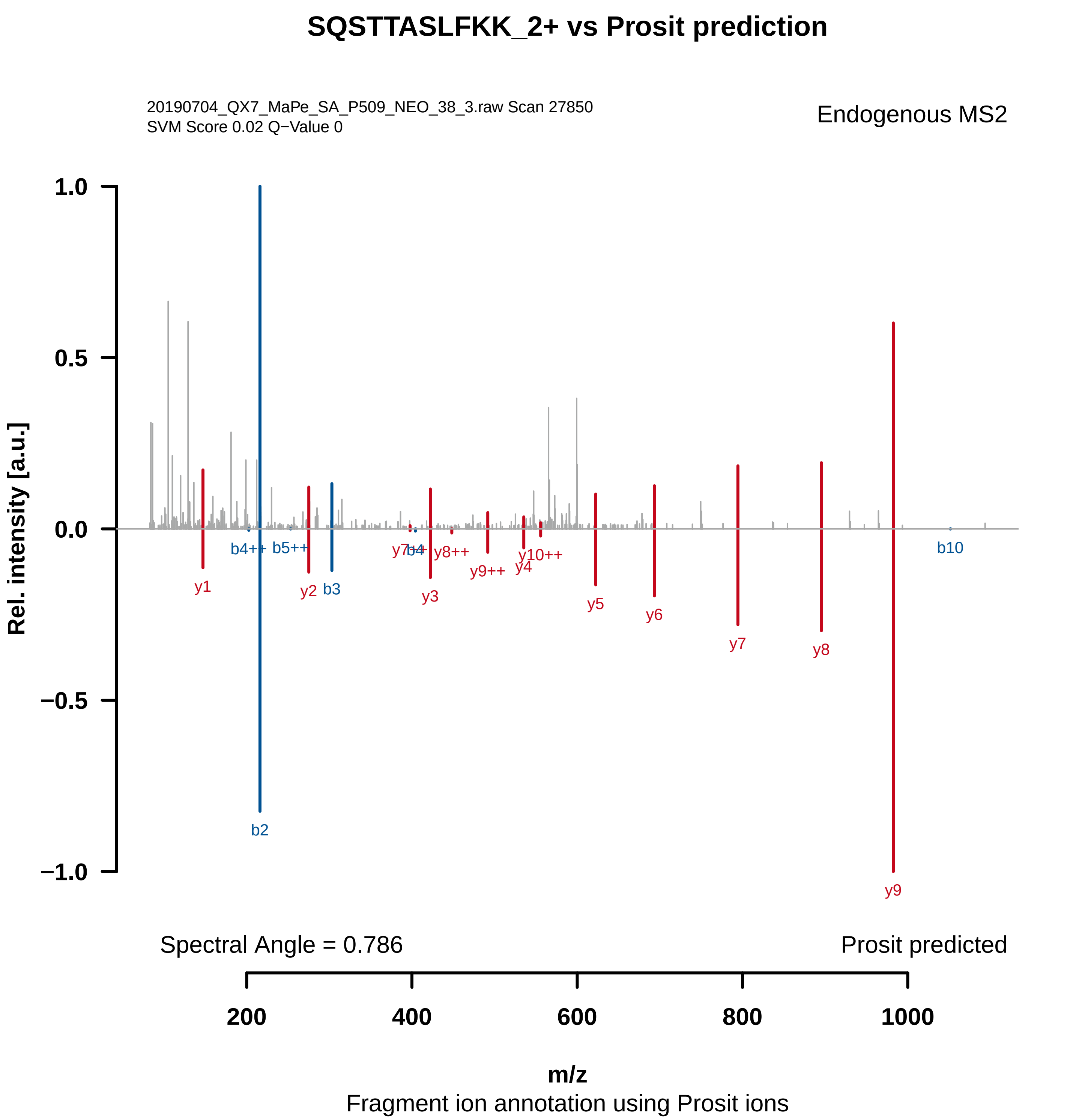
SQSTTASLFKK_2+ vs Prosit prediction
20190704_QX7_MaPe_SA_P509_NEO_38_3.raw Scan 27850
SVM Score 0.02 Q−Value 0
Endogenous MS2
Rel. intensity [a.u.]
1.0
0.5
0.0
−0.5
−1.0
b4++
b5++
y1
y2
b3
y7
b4
y8++
y3
y9++
y4
y10++
y5
y6
y7
y8
b10
b2
y9
Spectral Angle = 0.786
Prosit predicted
200
400
600
800
1000
m/z
Fragment ion annotation using Prosit ions

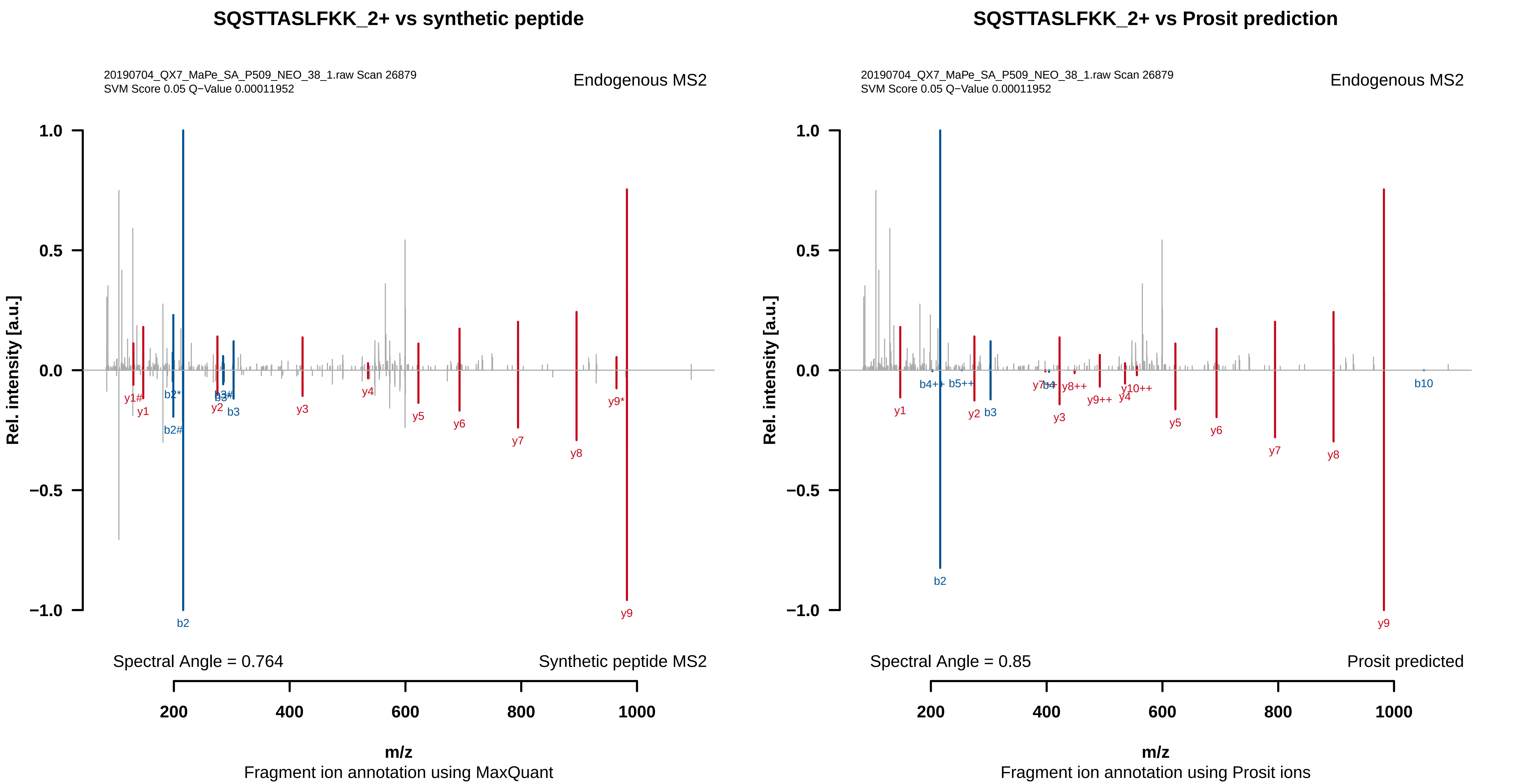

SQSTTASLFKK_2+ vs synthetic peptide
20190704_QX7_MaPe_SA_P509_NEO_38_1.raw Scan 26879
SVM Score 0.05 Q−Value 0.00011952
Endogenous MS2
Rel. intensity [a.u.]
1.0
0.5
0.0
−0.5
−1.0
y1#
y1
b2*
b2#
b2
b3#
b3*
b3
y2
y3
y4
y5
y6
y7
y8
y9*
y9
Spectral Angle = 0.764
Synthetic peptide MS2
200
400
600
800
1000
m/z
Fragment ion annotation using MaxQuant
SQSTTASLFKK_2+ vs Prosit prediction
20190704_QX7_MaPe_SA_P509_NEO_38_1.raw Scan 26879
SVM Score 0.05 Q−Value 0.00011952
Endogenous MS2
Rel. intensity [a.u.]
1.0
0.5
0.0
−0.5
−1.0
y1
b4++
b5++
b2
y2
b3
y7++
b4
y8++
y3
y9++
y4
y10++
y5
y6
y7
y8
y9
b10
Spectral Angle = 0.85
Prosit predicted
200
400
600
800
1000
m/z
Fragment ion annotation using Prosit ions

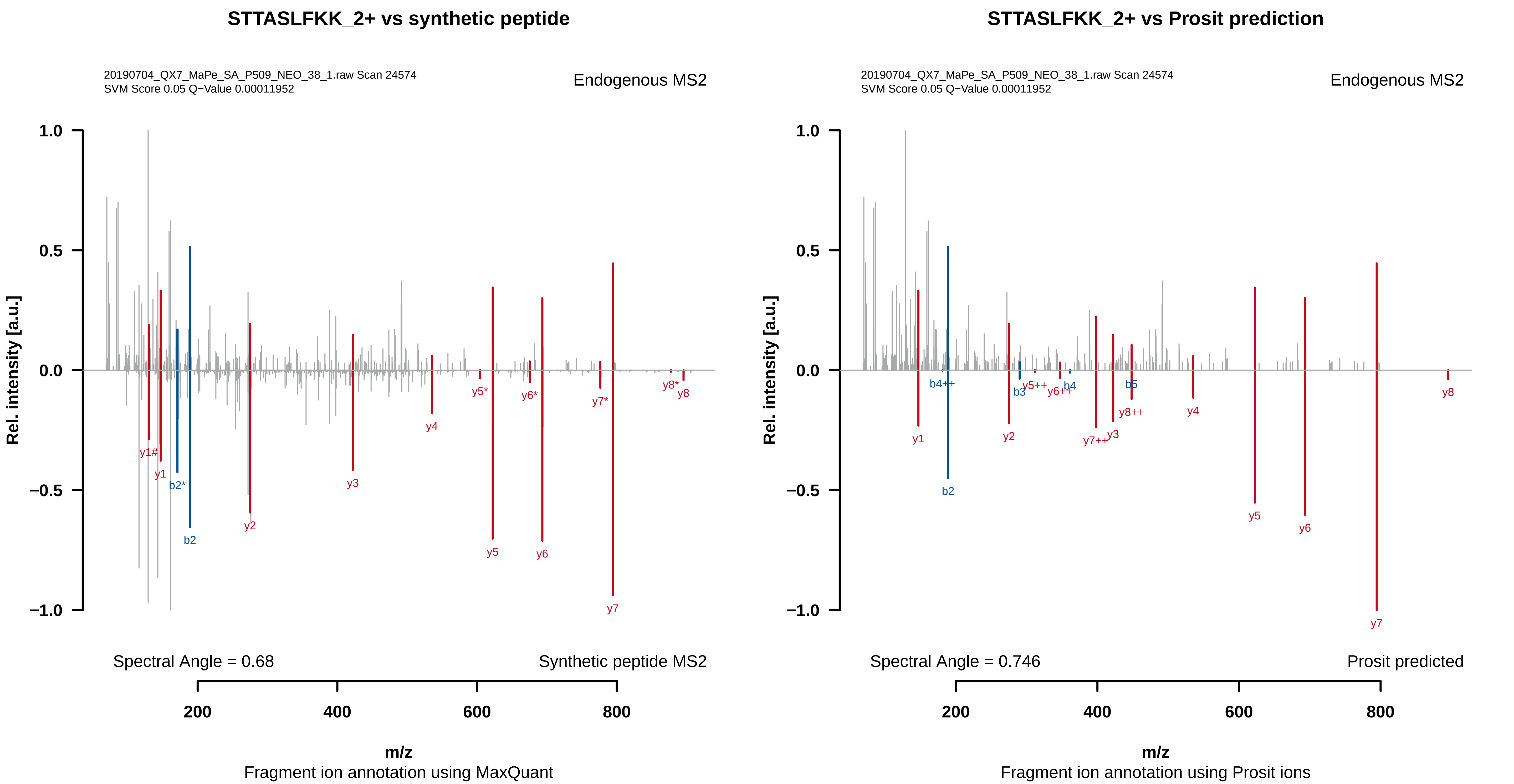

STTASLFKK_2+ vs synthetic peptide
20190704_QX7_MaPe_SA_P509_NEO_38_1.raw Scan 24574
SVM Score 0.05 Q−Value 0.00011952
Endogenous MS2
Rel. intensity [a.u.]
1.0
0.5
0.0
−0.5
−1.0
y1#
y1
b2*
b2
y2
y3
y4
y5*
y5
y6*
y6
y7*
y7
y8*
y8
Spectral Angle = 0.68
Synthetic peptide MS2
200
400
600
800
m/z
Fragment ion annotation using MaxQuant
STTASLFKK_2+ vs Prosit prediction
20190704_QX7_MaPe_SA_P509_NEO_38_1.raw Scan 24574
SVM Score 0.05 Q−Value 0.00011952
Endogenous MS2
Rel. intensity [a.u.]
1.0
0.5
0.0
−0.5
−1.0
y1
b4++
b2
b3
y2
y5++
y6++
b4
y7++
y3
y8++
b5
y4
y5
y6
y7
y8
Spectral Angle = 0.746
Prosit predicted
200
400
600
800
m/z
Fragment ion annotation using Prosit ions

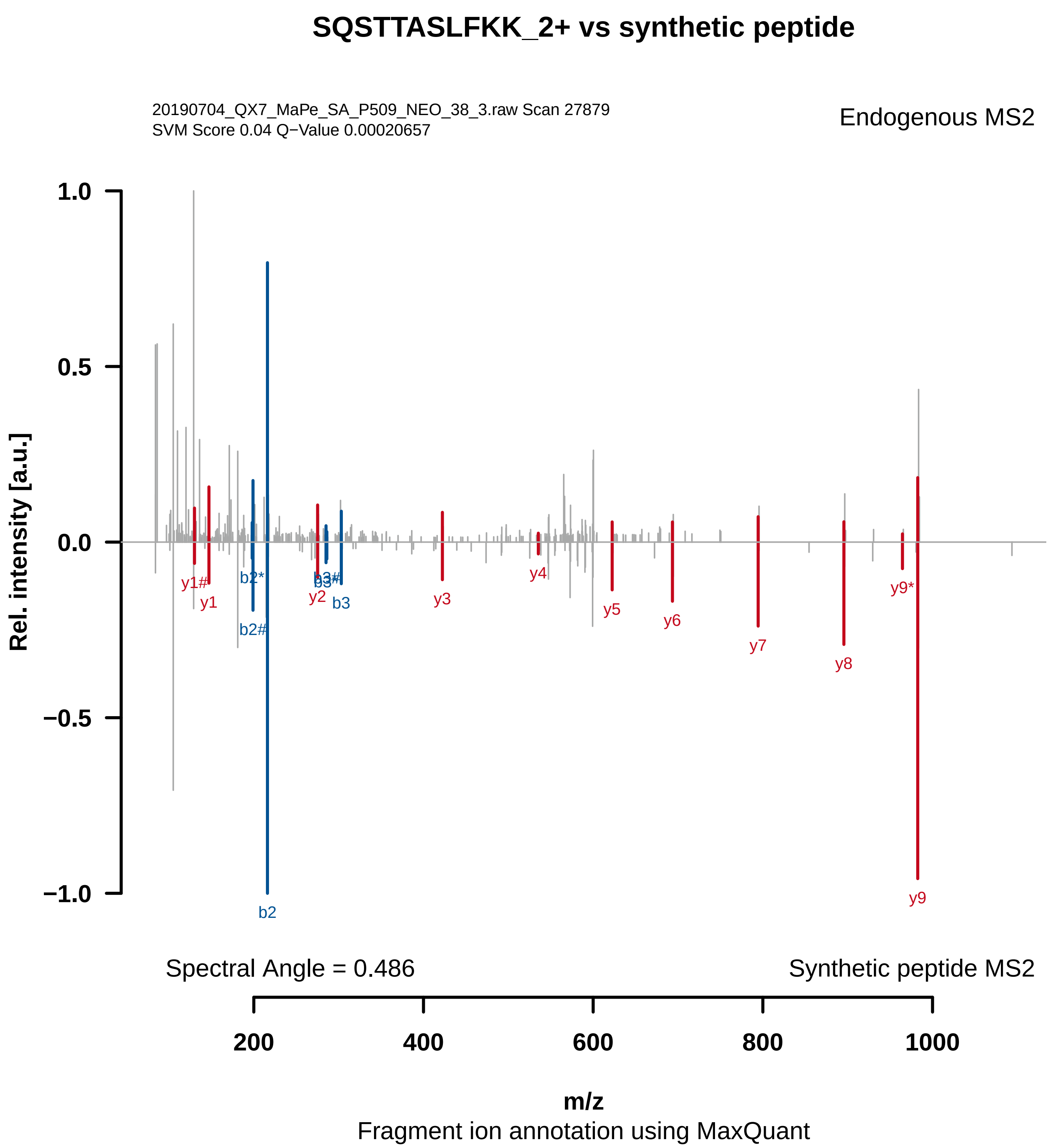
SQSTTASLFKK_2+ vs synthetic peptide
20190704_QX7_MaPe_SA_P509_NEO_38_3.raw Scan 27879
SVM Score 0.04 Q−Value 0.00020657
Endogenous MS2
Rel. intensity [a.u.]
1.0
0.5
0.0
−0.5
−1.0
y1#
y1
b2*
b2#
b2
y2
b3#
b3*
b3
y3
y4
y5
y6
y7
y8
y9*
y9
Spectral Angle = 0.486
Synthetic peptide MS2
200
400
600
800
1000
m/z
Fragment ion annotation using MaxQuant

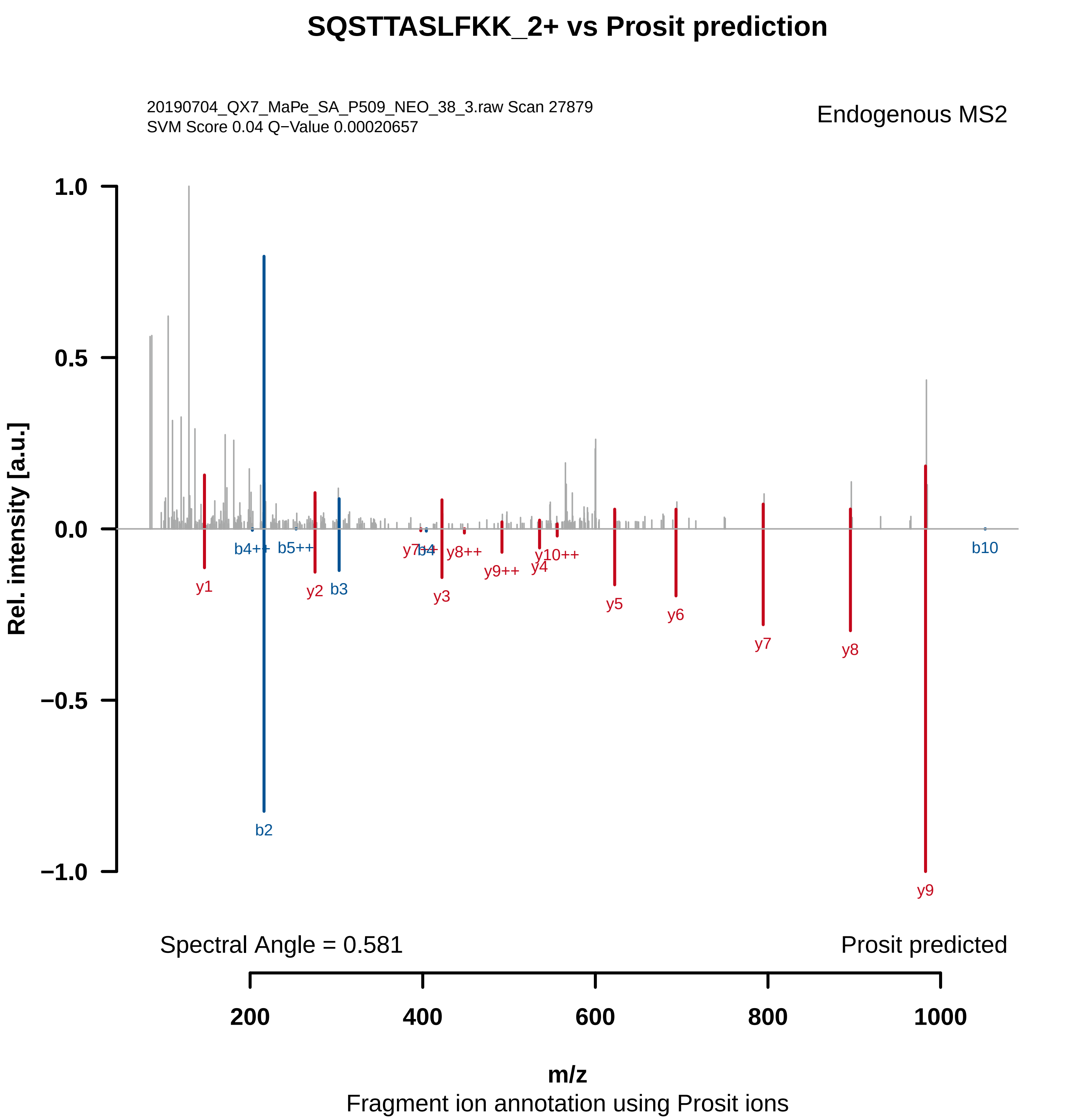
SQSTTASLFKK_2+ vs Prosit prediction
20190704_QX7_MaPe_SA_P509_NEO_38_3.raw Scan 27879
SVM Score 0.04 Q−Value 0.00020657
Endogenous MS2
Rel. intensity [a.u.]
1.0
0.5
0.0
−0.5
−1.0
y1
b4++
b5++
b2
y2
b3
y7++
b4
y3
y8++
y9++
y4
y10++
y5
y6
y7
y8
y9
b10
Spectral Angle = 0.581
Prosit predicted
200
400
600
800
1000
m/z
Fragment ion annotation using Prosit ions

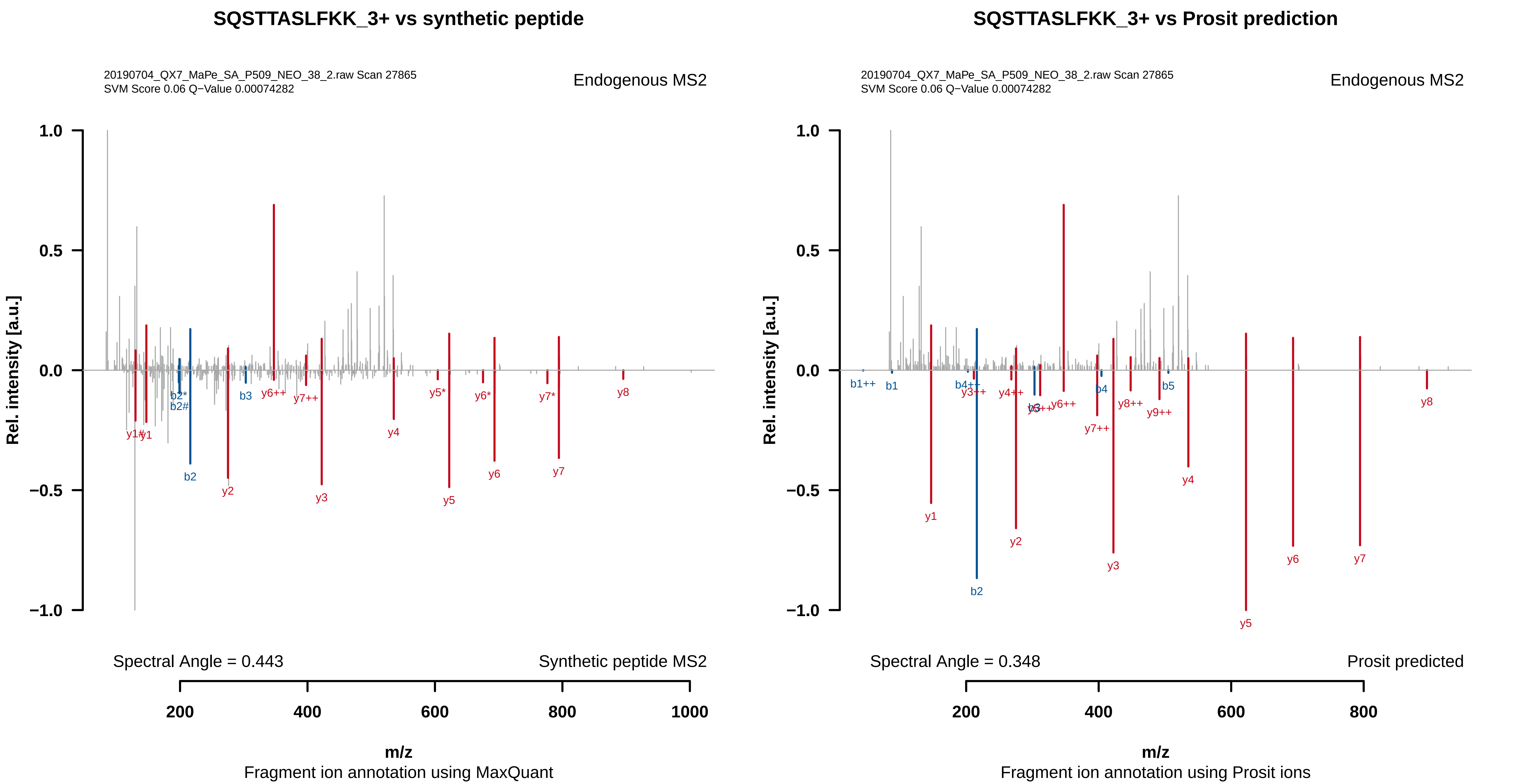
SQSTTASLFKK_3+ vs synthetic peptide
20190704_QX7_MaPe_SA_P509_NEO_38_2.raw Scan 27865
SVM Score 0.06 Q−Value 0.00074282
Endogenous MS2
Rel. intensity [a.u.]
Spectral Angle = 0.443
Synthetic peptide MS2
m/z
Fragment ion annotation using MaxQuant
SQSTTASLFKK_3+ vs Prosit prediction
20190704_QX7_MaPe_SA_P509_NEO_38_2.raw Scan 27865
SVM Score 0.06 Q−Value 0.00074282
Endogenous MS2
Rel. intensity [a.u.]
Spectral Angle = 0.348
Prosit predicted
m/z
Fragment ion annotation using Prosit ions

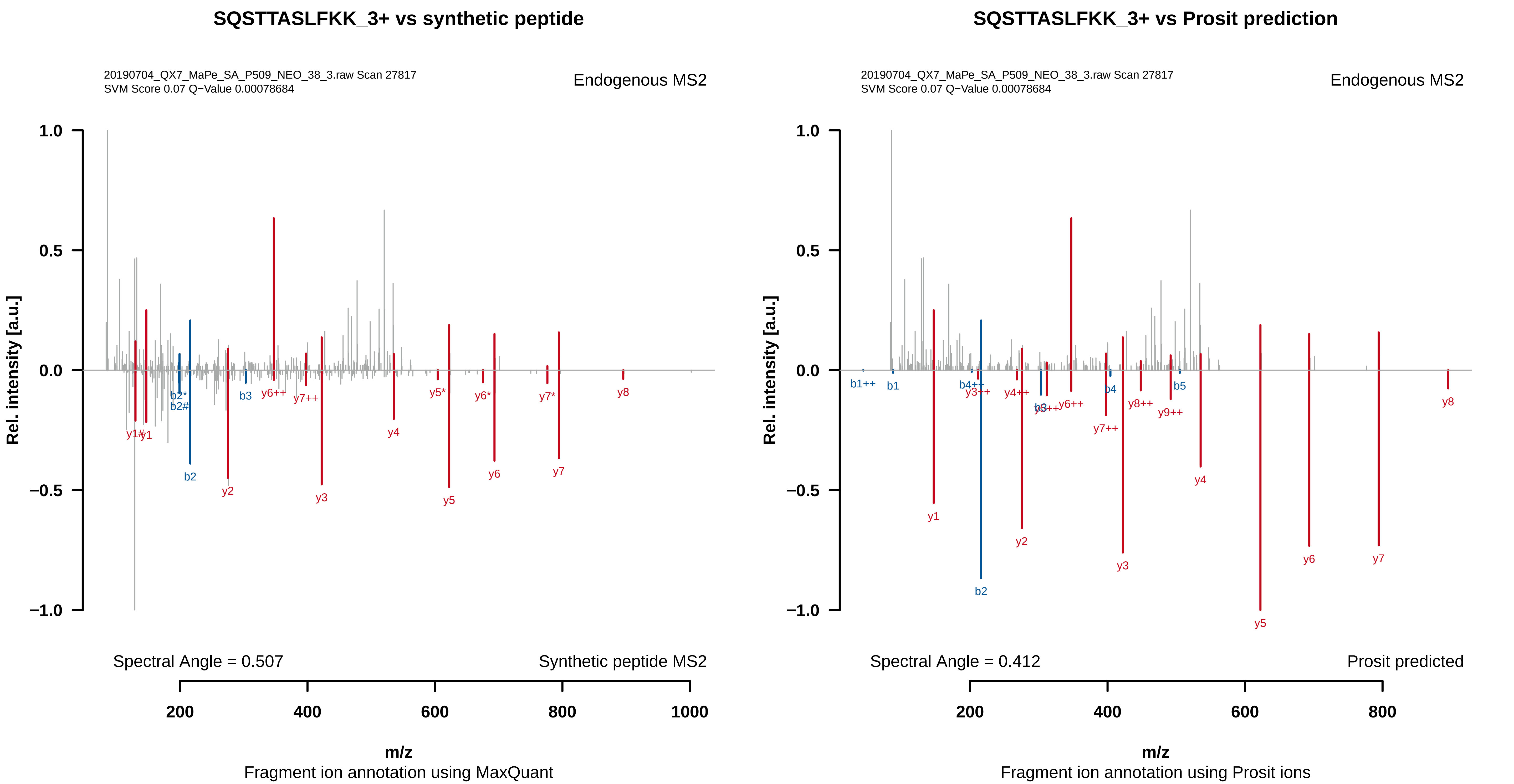
SQSTTASLFKK_3+ vs synthetic peptide
20190704_QX7_MaPe_SA_P509_NEO_38_3.raw Scan 27817
SVM Score 0.07 Q−Value 0.00078684
Endogenous MS2
Rel. intensity [a.u.]
1.0
0.5
0.0
−0.5
−1.0
b2*
b2#
b3
y6++
y7++
y5*
y6*
y7*
y8
y1#
y1
b2
y2
y3
y4
y5
y6
y7
Spectral Angle = 0.507
Synthetic peptide MS2
200
400
600
800
1000
m/z
Fragment ion annotation using MaxQuant
SQSTTASLFKK_3+ vs Prosit prediction
20190704_QX7_MaPe_SA_P509_NEO_38_3.raw Scan 27817
SVM Score 0.07 Q−Value 0.00078684
Endogenous MS2
Rel. intensity [a.u.]
1.0
0.5
0.0
−0.5
−1.0
b1++
b1
b4++
y3++
y4++
b3
y5++
y6++
b4
y7++
y8++
y9++
b5
y4
y8
y1
y2
y3
b2
y5
y6
y7
Spectral Angle = 0.412
Prosit predicted
200
400
600
800
m/z
Fragment ion annotation using Prosit ions

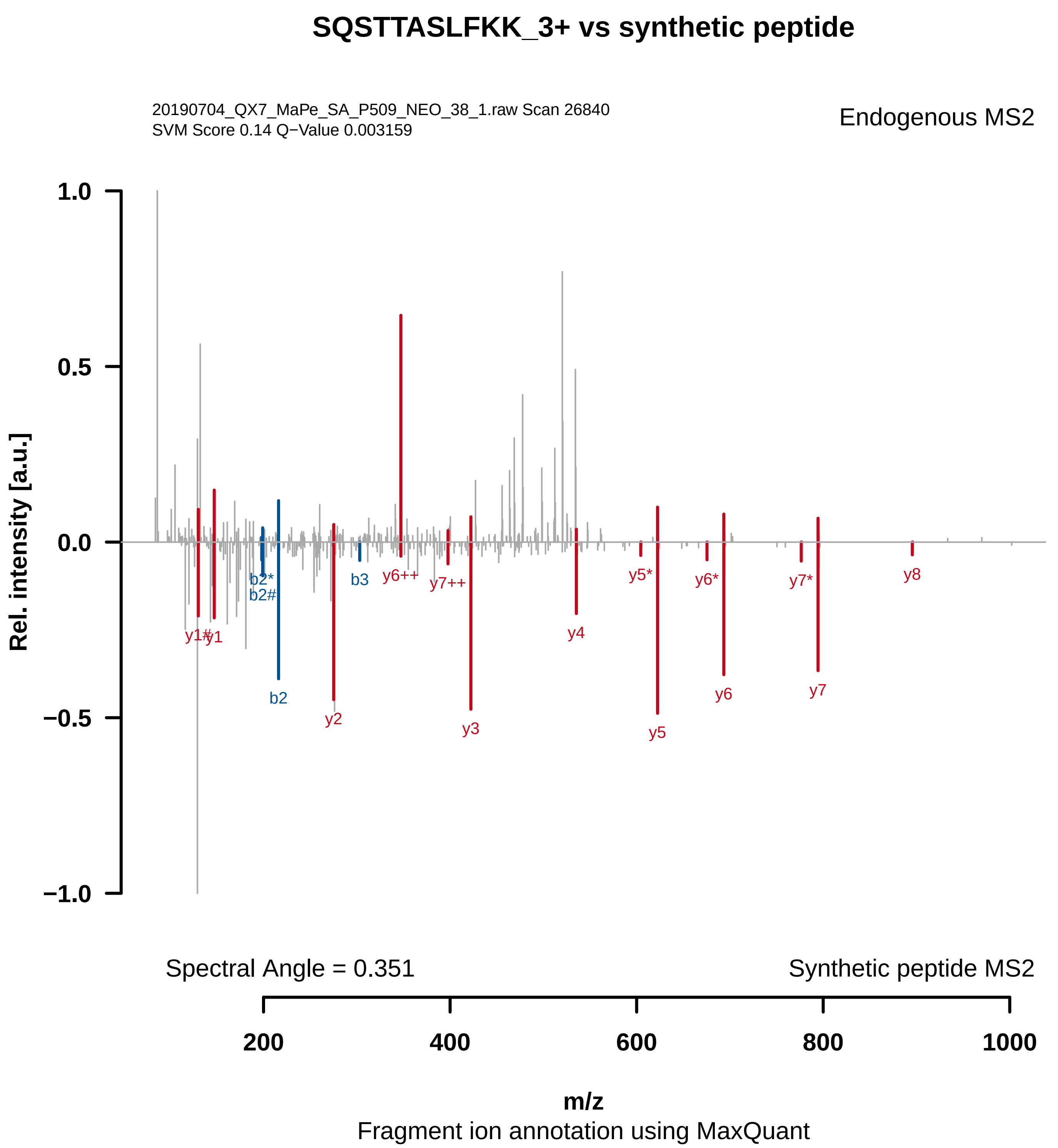
SQSTTASLFKK_3+ vs synthetic peptide
20190704_QX7_MaPe_SA_P509_NEO_38_1.raw Scan 26840
SVM Score 0.14 Q−Value 0.003159
Endogenous MS2
Rel. intensity [a.u.]
1.0
0.5
0.0
−0.5
−1.0
y1#
y1
b2*
b2#
b2
b3
y2
y6++
y7++
y3
y4
y5*
y5
y6*
y6
y7*
y7
y8
Spectral Angle = 0.351
Synthetic peptide MS2
200
400
600
800
1000
m/z
Fragment ion annotation using MaxQuant

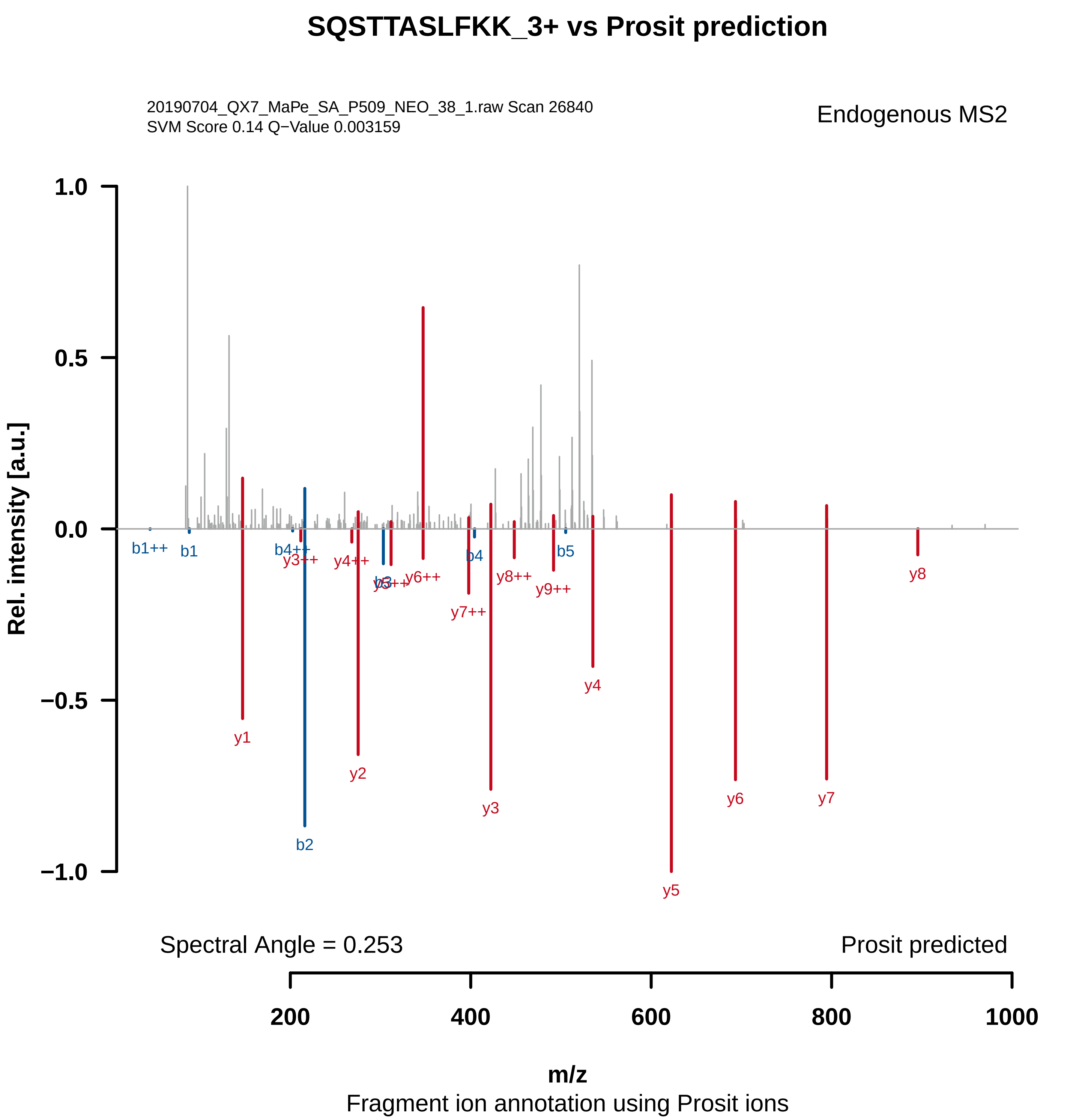
SQSTTASLFKK_3+ vs Prosit prediction
20190704_QX7_MaPe_SA_P509_NEO_38_1.raw Scan 26840
SVM Score 0.14 Q−Value 0.003159
Endogenous MS2
Rel. intensity [a.u.]
1.0
0.5
0.0
−0.5
−1.0
b1++
b1
b4++
y3++
y4++
b3
y5++
y6++
b4
y8++
b5
y9++
y7++
y1
b2
y2
y3
y4
y5
y6
y7
y8
Spectral Angle = 0.253
Prosit predicted
200
400
600
800
1000
m/z
Fragment ion annotation using Prosit ions

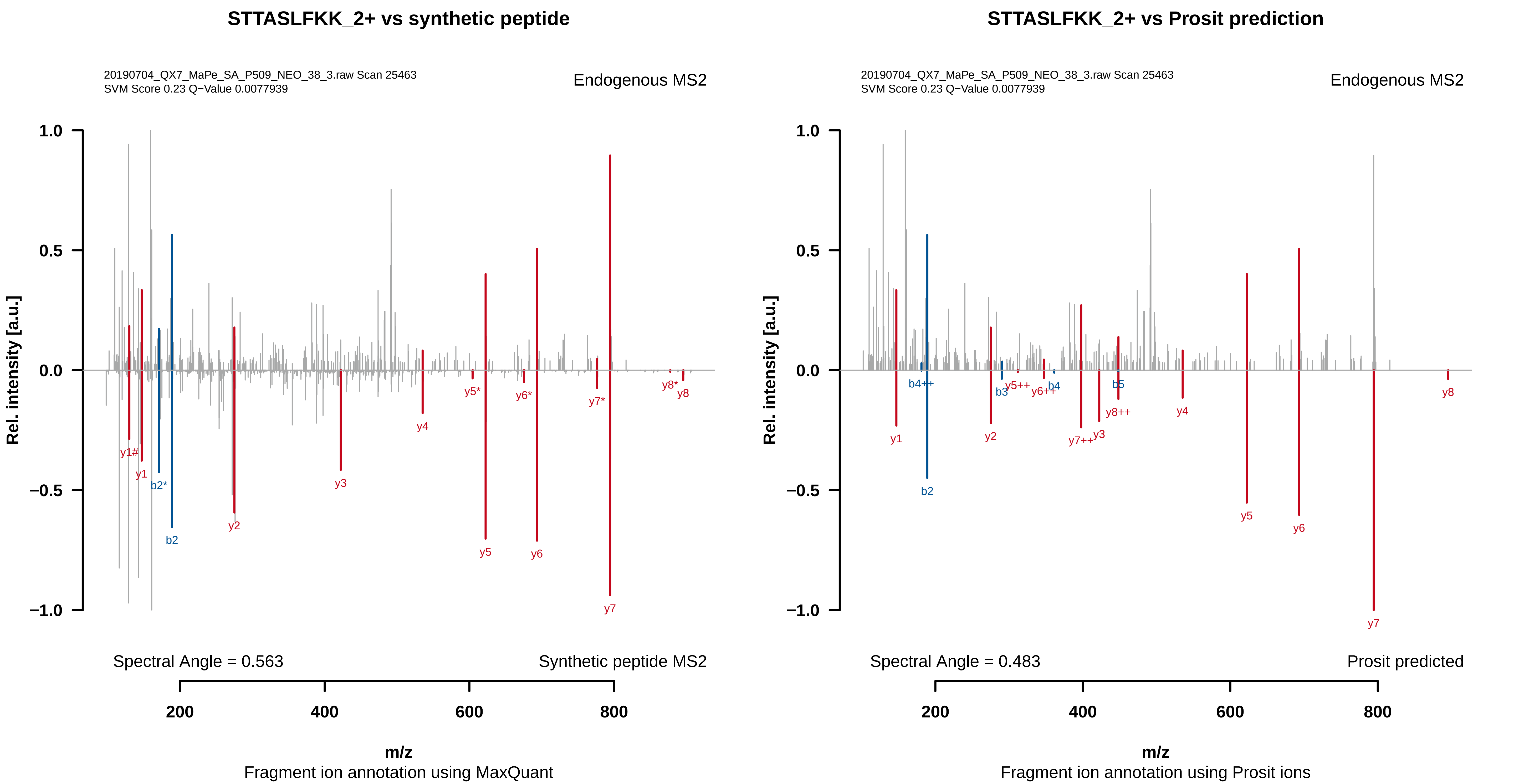
STTASLFKK_2+ vs synthetic peptide
20190704_QX7_MaPe_SA_P509_NEO_38_3.raw Scan 25463
SVM Score 0.23 Q−Value 0.0077939
Endogenous MS2
Rel. intensity [a.u.]
1.0
0.5
0.0
−0.5
−1.0
y1#
y1
b2*
b2
y2
y3
y4
y5*
y5
y6*
y6
y7*
y7
y8*
y8
Spectral Angle = 0.563
Synthetic peptide MS2
200
400
600
800
m/z
Fragment ion annotation using MaxQuant
STTASLFKK_2+ vs Prosit prediction
20190704_QX7_MaPe_SA_P509_NEO_38_3.raw Scan 25463
SVM Score 0.23 Q−Value 0.0077939
Endogenous MS2
Rel. intensity [a.u.]
1.0
0.5
0.0
−0.5
−1.0
b4++
y1
b2
b3
y2
y5++
y6++
b4
y7++
y3
b5
y8++
y4
y5
y6
y7
y8
Spectral Angle = 0.483
Prosit predicted
200
400
600
800
m/z
Fragment ion annotation using Prosit ions

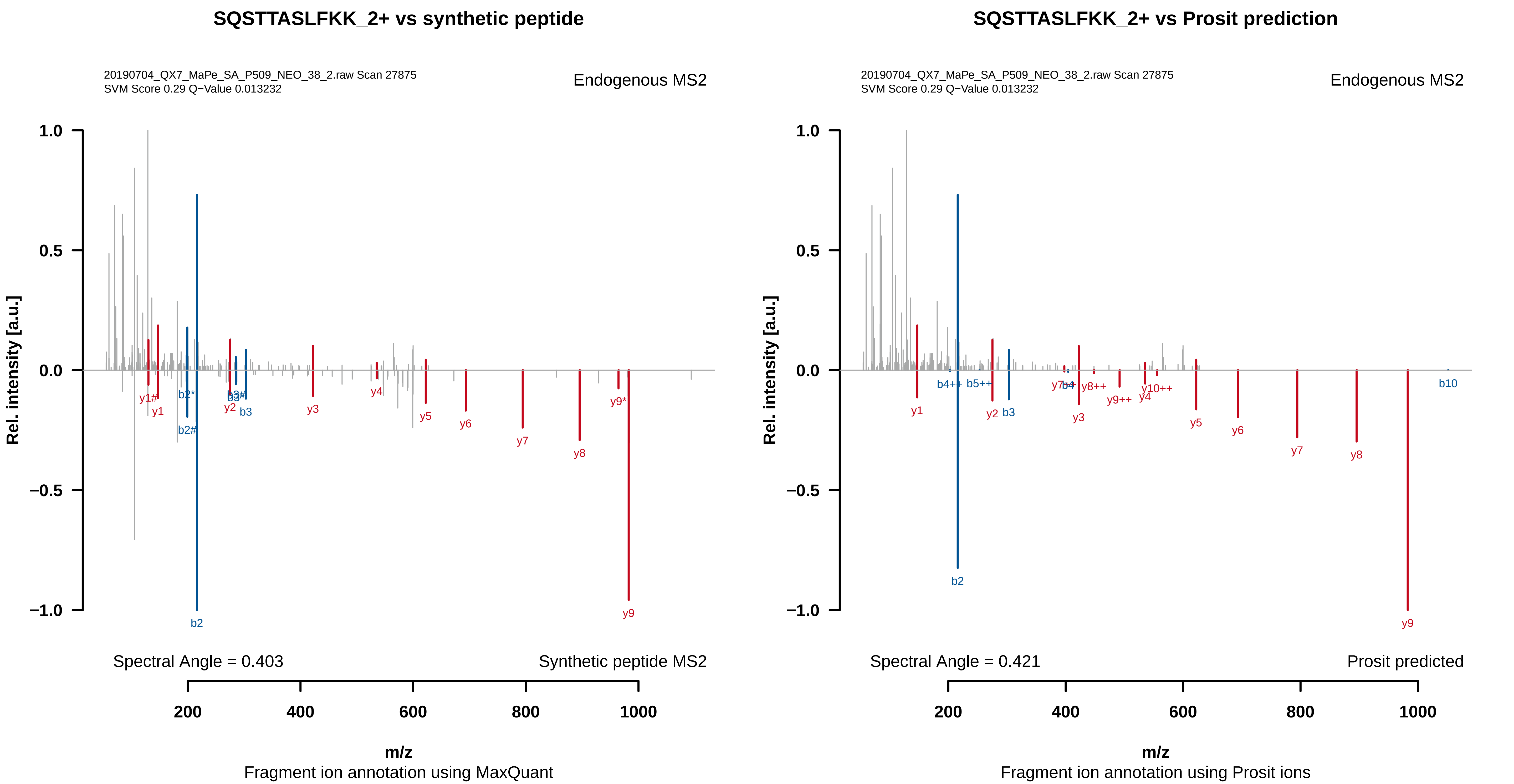
SQSTTASLFKK_2+ vs synthetic peptide
20190704_QX7_MaPe_SA_P509_NEO_38_2.raw Scan 27875
SVM Score 0.29 Q−Value 0.013232
Endogenous MS2
Rel. intensity [a.u.]
Spectral Angle = 0.403
Synthetic peptide MS2
m/z
Fragment ion annotation using MaxQuant
SQSTTASLFKK_2+ vs Prosit prediction
20190704_QX7_MaPe_SA_P509_NEO_38_2.raw Scan 27875
SVM Score 0.29 Q−Value 0.013232
Endogenous MS2
Rel. intensity [a.u.]
Spectral Angle = 0.421
Prosit predicted
m/z
Fragment ion annotation using Prosit ions
